# Design, Synthesis and Biological Evaluation of Highly Potent Simplified Archazolids

**DOI:** 10.1002/cmdc.202000154

**Published:** 2020-06-10

**Authors:** Solenne Rivière, Christin Vielmuth, Christiane Ennenbach, Aliaa Abdelrahman, Carina Lemke, Michael Gütschow, Christa E. Müller, Dirk Menche

**Affiliations:** ^1^ Kekulé-Institut für Organische Chemie und Biochemie Universität Bonn Gerhard-Domagk-Strasse 1 53121 Bonn Germany; ^2^ Pharmazeutische & Medizinische Chemie Pharmazeutisches Institut Universität Bonn An der Immenburg 4 53121 Bonn Germany

**Keywords:** anticancer agents, macrolactonization, macrolides, polyenes, polyketides

## Abstract

The archazolids are potent antiproliferative compounds that have recently emerged as a novel class of promising anticancer agents. Their complex macrolide structures and scarce natural supply make the development of more readily available analogues highly important. Herein, we report the design, synthesis and biological evaluation of four simplified and partially saturated archazolid derivatives. We also reveal important structure‐activity relationship data as well as insights into the pharmacophore of these complex polyketides.

## Graphical abstract

**Analogue design**: Simplified archazolids that retained biological potency were efficiently synthesized using an aldol condensation sequence along with a macrolactonization strategy tot close the 24‐membered ring.. Biological assessment of these new analogues gave insights into the archazolids’ pharmacophore: modifications in the C3–C6 area were well tolerated in the assays with inhibition at μmol range suggesting that further simplifications on the archazolid macrocyle might be allowed

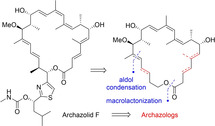

## Introduction

**Figure 1:**
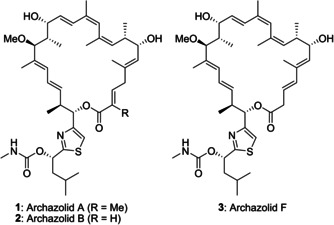
Potent members of the archazolid family.

Extended polyene segments are key structural features of a broad range of complex polyketide macrolide antibiotics. The archazolids A (**1**) and B (**2**, Figure 1) are typical representatives which were first reported in the 1990s by the Höfle group as a novel class of highly potent antiproliferative agents.^1^ A decade later, Sasse *et al*. and Huss *et al*. reported V‐ATPase as a molecular target inhibited by archazolids,^2^, ^3^ and subsequently, the binding site has been defined.^4^, ^5^ In 2011, archazolid F (**3**), was demonstrated to display higher antiproliferative activity making it the most potent member of this family.^6^ In recent years, the archazolids have also been shown to exhibit remarkable inhibitory effects of tumor growth, and based on these studies they have emerged as a promising class of novel anticancer drugs.^7^, ^8^, ^9^, ^10^, ^11^, ^12^ Furthermore, the G protein‐coupled A_3_‐adenosine receptor, the ATP‐gated ion channel receptor P2X3, and human leukocyte elastase have been discovered as further molecular targets of archazolids, which may contribute to their anticancer activities.^13^

The archazolids are 24‐membered macrolactones with eight stereocenters, 7 double bonds and a thiazole side chain. As they are only produced in scarce quantities by nature, there is a need for a synthetic approach to provide sufficient amounts for studies on their mode of action and their target selectivity. So far, one total synthesis of archazolid A was published by us in 2007,^14^ and two total syntheses of archazolid B have been reported by the Trauner group^15^ and our group in 2007 and 2009.^16^ In 2018, we accomplished the total synthesis of archazolid F.^17^ Furthermore, elaborate fragment synthesis of 2,3‐dihydroarchazolid was published by O'Neil *et al*.^18^, ^19^, ^20^

## Design of new simplified archazolid derivatives

**Figure 2:**
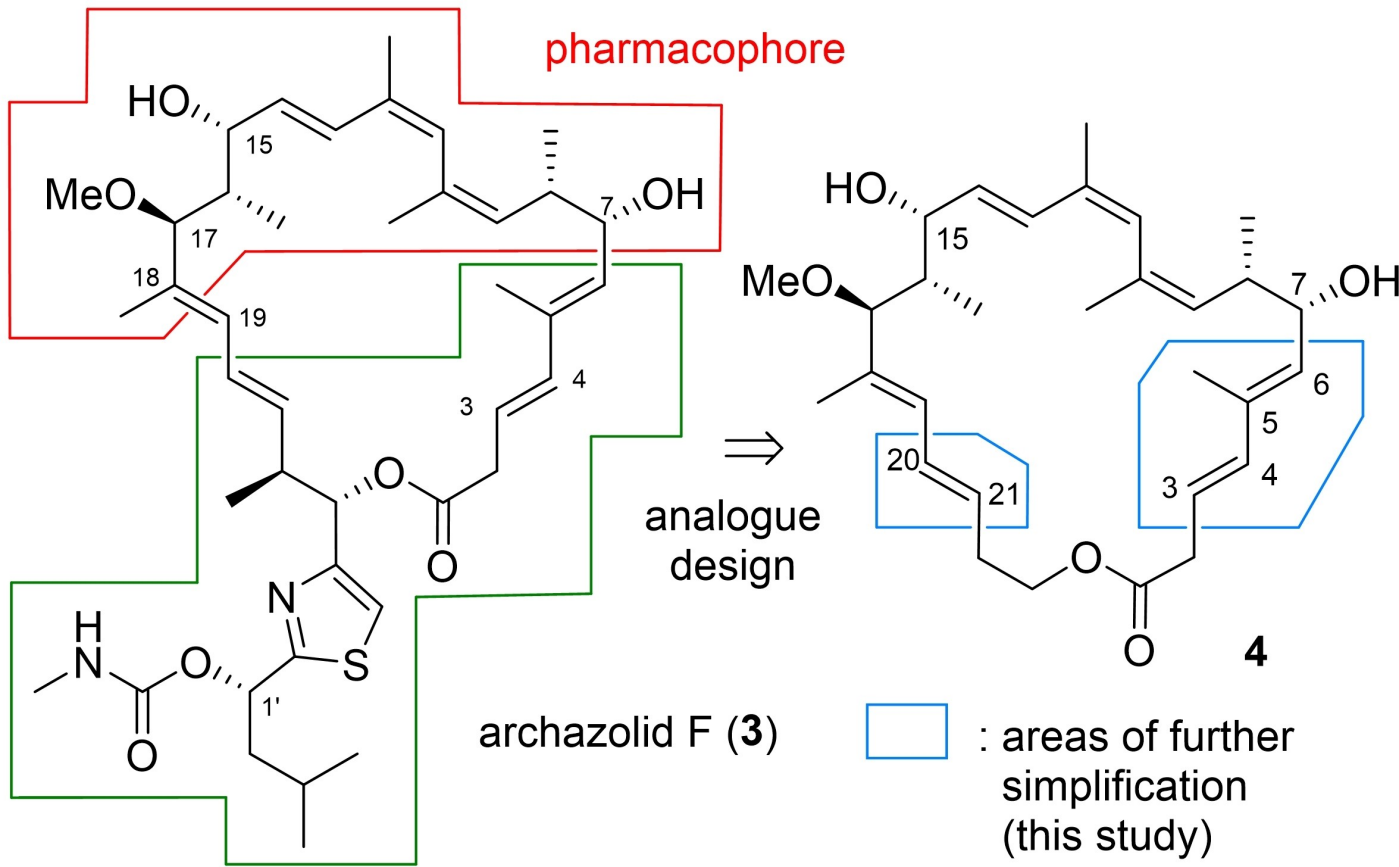
Proposed pharmacophoric area of the archazolids leading to the design of potent archazolog **4** 6 and further simplifications addressed within this study.

Despite various total syntheses, only few SAR studies have been published so far, relying on compounds obtained by chemical derivatization of natural archazolid A^21^ or on acyclic fragments.^21^, ^22^ Initial archazolid derivatizations mainly occurred on the two free hydroxy groups as well as on the carbamate side chain. In detail, modification of either hydroxy function led to a drop in potency,^21^ whereas removal of the carbamate side chain had only a minor effect on biological activity.^22^ Hence, it was proposed that the northern part would be critical for binding, as shown in Figure 2. This hypothesis was further supported by docking calculations and molecular dynamics experiments.^23^ Accordingly, a novel synthetic route towards such macrolides was developed and applied for the total synthesis of archazolid F.^17^ This strategy relied on disconnections of the C18–C19 bond, by an aldol condensation and a ring closing metathesis along the C3–C4 bond. The synthetic methodology route was subsequently used for the total synthesis of a first series of unnatural analogues.^13^ The substantially simplified analogue **4** (Figure 2) was discovered which still exhibited excellent antiproliferative activity towards several mammalian cancer cell lines, even surpassing the activity of natural archazolid F. These results confirmed our previous hypothesis that the archazolids’ binding site is located in the northern, top part of the macrolactone.

**Figure 3:**
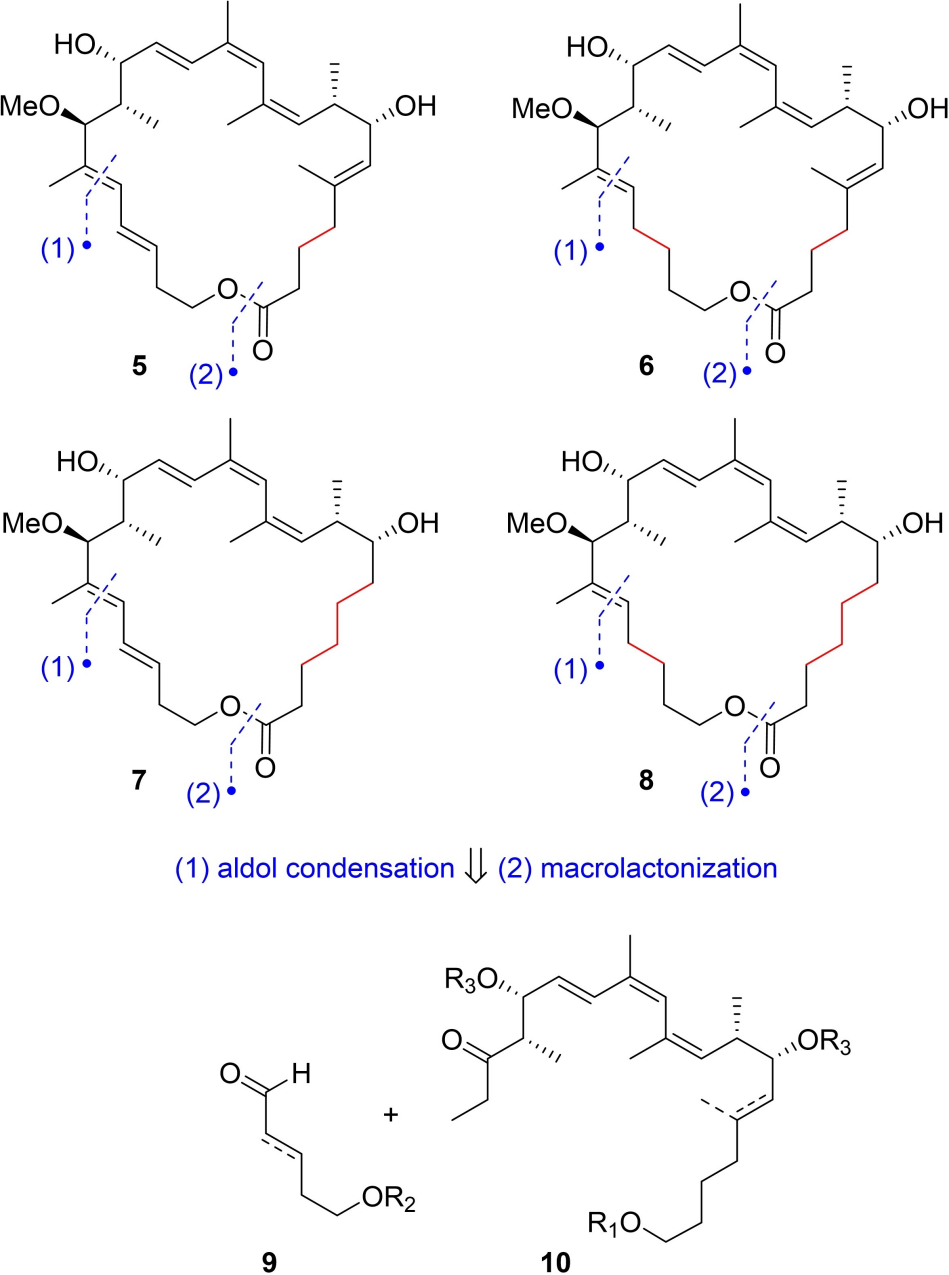
Targeted analogues of this work and their retrosynthetic analysis.

Based on the structure of analogue **4**, a further series of derivatives was devised for this study, focusing on additional simplifications of the southern part. Modifications were gathered around saturations of the three double bonds C3–C4, C5–C6 and C20–C21 as well as the elimination of the C5 methyl group. Loss of these double bonds would introduce more flexibility into the macrocycle and also shorten the synthetic route. Removal of one double bond could indicate its relevance for biological activity. Based on this rationale, the four derivatives **5**–**8** (Figure 3) were envisaged.

## Results and Discussion

The synthesis of these derivatives uses a methodology developed during the total synthesis of archazolid F.^17^ As shown in Figure 3, the implementation of the analogues **5**–**8** was achieved by the combination of two fragments, that is, a main northern subunit of type **10** and various southern segments of type **9**. Following our own precedence,^17^ an aldol‐condensation sequence was planned to forge the 18,19‐double bond, while a novel macrolactonization approach was considered to close the ring.

**Scheme 1:**
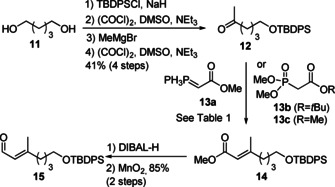
Synthesis of aldehyde **15**.

**Scheme 2:**
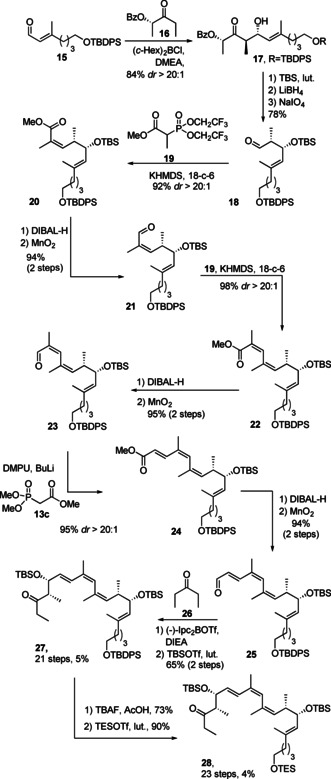
Synthesis of main fragments **27** and **28**.

**Scheme 3:**
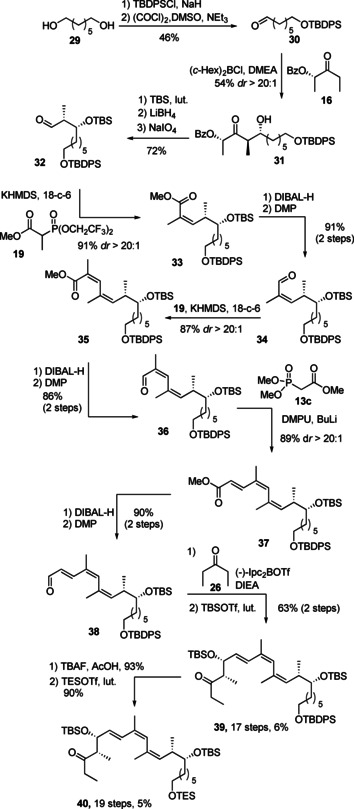
Synthesis of main fragments **39** and **40**.

Schemes 1, 2 and 3 show the synthesis of the main fragments **27**, **28**, **39** and **40** by robust and reliable routes involving aldol and olefination reactions that have previously been established on related systems.^14^, ^16^ As shown in Schemes 2 and 3, we first focused on the preparation of the main fragments **27** and **28**, which were required for analogues **5** and **6**. Their synthesis started with ketone **12** which was obtained in four steps from commercially available pentandiol **11** (Scheme 1). C2 homologation was initially attempted with Wittig ylide **13 a** (Table 1) which was found to be too unreactive to produce ester **14**. On the contrary, Horner‐Wadsworth‐Emmons (HWE) reagents such as **13 b** and **c** were more appropriate. Although rather low yields and selectivities were obtained using NaH or Potassium bis(trimethylsilyl)amide (KHMDS; Table 1), BuLi was found to result in higher degrees of conversion but still low selectivity. The presence of a bulkier R group on the phosphonate was described to increase the selectivity.^24^ However, in our case with phosphonate **13 b**, the *E*/*Z* ratio was only slightly improved from 2 : 1 to 3 : 1. The best conditions involved the use of phosphonate **13 c** and the addition of *N,N′*‐dimethylpropylene urea (DMPU) in combination with BuLi at room temperature with prolongated reaction times overnight, resulting in a high yield (80 %). At this stage, the selectivity of 3 : 1 was accepted as the two isomers were easily separated by column chromatography. Finally, the resulting enoate **14** was converted to aldehyde **15** in two steps. This route proved to be scalable and employed inexpensive starting materials.

**Table 1:** Olefination reactions of ketone **12**.

Reactants	Conditions	Yield^[a]^	*E*/*Z*
12+13a	CH_2_Cl_2_, reflux, 24 h	–^[b]^	–
12+13a	toluene, reflux, 24 h	–^[b]^	–
12+13c	NaH, THF, RT, 24 h	16 %	2 : 1
12+13c	KHMDS, THF, RT, 24 h	36 %	2 : 1
12+13b	*n*BuLi, THF, RT, o/n	52 %	3 : 1
12+13c	DMPU, *n*BuLi, THF, RT, o/n	80 %	3 : 1

[a] Combined yield. [b] No conversion.

As shown in Scheme 2, aldehyde **15** was then subjected to a boron‐mediated Paterson aldol reaction with the (*S*)‐lactate‐derived ketone **16**,^25^ which proceeded with excellent yield and diastereoselectivity (*dr*>20 : 1) towards *β*‐hydroxyketone **17**. After *tert*‐butyldimethylsilyl (TBS) protection, LiBH_4_ reduction and cleavage of the diol with NaIO_4_, aldehyde **18** was obtained. The *Z*/*Z*/*E* triene was then generated using two consecutive Still‐Gennari reactions and an HWE olefination with excellent yield and selectivity. After reduction and oxidation of ester **24**, the required building block **27** was obtained by a *syn*‐boron‐mediated aldol reaction with diethyl ketone **26** 26 and TBS protection. For the synthesis of analogue **5** (see below), the *tert*‐butyldiphenylsilyl (TBDPS) group had to be replaced by a triethylsilane (TES) group. Accordingly, the primary hydroxy group of **27** was selectively liberated in presence of the two secondary TBS groups using tetrabutyl ammonium fluoride (TBAF)/AcOH conditions^27^ and reprotected as a TES ether towards **28**.

The more simplified main fragments **39** and **40** which lack the C2–C3 and C4–C5 double bonds as well as the C5 methyl group as required for archazologs **7** and **8** were prepared in an analogous manner (Scheme 3). In detail, both the corresponding Paterson aldol coupling with derived aldehyde **30**, the two consecutive Still‐Gennari olefinations with aldehydes **32** and **34**, as well as the HWE‐olefination with **36** and the final Ipc‐mediated boron aldol reaction of **38** proceeded with excellent selectivity, giving the required chiral triene building block **39** in an effective and scalable fashion. Likewise, all intermediate interconversions, mainly involving adjustments of the required oxidation states of **31**, **33**, **35**, and **37** could also be carried out in reliable fashions and high yields. The corresponding TES ether **40** was prepared again by the facile deprotection/reprotection sequence.

**Scheme 4:**
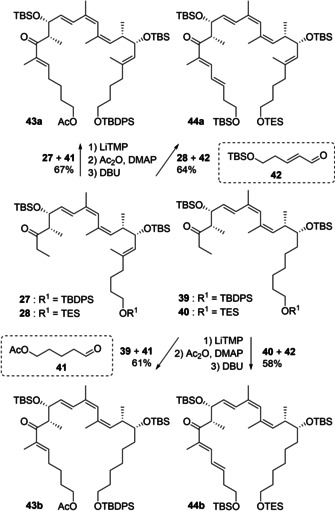
Coupling of the main fragments by an aldol‐condensation sequence.

With these northern fragments in hand, efforts were directed towards the pivotal aldol condensation sequence to access the fully functionalized carbon skeleton of the desired analogues (Scheme 4). The required aldehyde **41** was obtained from the corresponding diol by mono‐acetate protection and Swern oxidation, while **42** was prepared from but‐3‐en‐1‐ol^28^ by cross metathesis with acrolein and TBS protection. Gratifyingly, a three step aldol‐condensation sequence could be implemented, which proceeded with excellent selectivity as well as good yield. In particular, full degrees of conversions of the starting ketones **27**, **28**, **39** and **40** in the initial aldol coupling were obtained with lithium tetramethylpiperidine (LiTMP). Indeed, it was found that LiTMP offers the double benefit of full conversion and facile work‐up in contrast to Ph_2_NLi used in the total synthesis of archazolid F.^17^. Acetate esterification of the aldol products and a 1,8‐Diazabicyclo[5.4. 0]undec‐7‐ene (DBU)‐mediated elimination then afforded the desired unsaturated ketones **43 a**/**b**–**44 a/b**. Excellent *E* selectivity was obtained in the final elimination step by careful temperature control in the initial aldol reaction. Indeed, an increase of the temperature over −30 °C during the enolate formation resulted in an approximately 3 : 1 *E*/*Z* mixture after the elimination step to **43 a** and **43 b**.

**Scheme 5:**
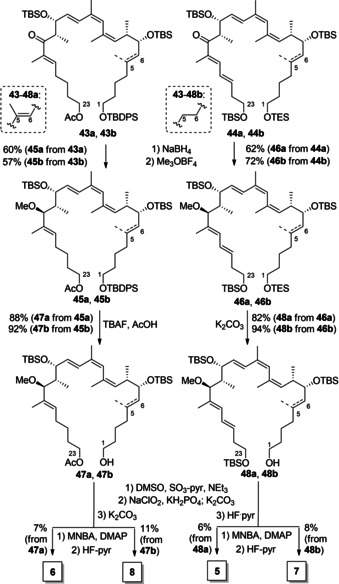
Completion of the synthesis of analogues **5**–**8** by macrolactonization.

As shown in Scheme 5, for completion of the synthesis, ketones **43 a/b** and **44 a/b** were selectively reduced by means of NaBH_4_. This procedure was originally described by the Trauner group^15^ in their total synthesis of archazolid B and had subsequently also been used by us in the preparations of archazolid F^17^ and related analogues.^13^ Gratifyingly, this protocol again proceeded with good selectivity (*dr* 10 : 1) and yields to give, after methylation with Meerwein salt, the corresponding ethers **47 a**/**b** and **48 a**/**b**. The protecting group at the C1 hydroxy group was then selectively removed under TBAF/AcOH conditions for the TBDPS groups of **47 a** and **47 b** and K_2_CO_3_ for the TES groups of **48 a** and **48 b**. The primary alcohols were then oxidized to the carboxylic acids in two steps applying the Parikh‐Doering and Pinnick procedures. The C23 hydroxy protecting groups were selectively removed with K_2_CO_3_ for the acetate groups (Scheme 5, left) and HF‐pyr for the primary TBS groups (right) affording the corresponding alcohols **47 a/b** and **48 a/b**. Deprotection at the C1 hydroxy group as well as the two oxidations to the carboxylic acids proceeded smoothly while deprotection of the C23 positions was less satisfying (40‐50 % yield). The macrocycles were then closed using the Shiina macrolactonization method. Slow addition of the seco acids to a highly diluted solution of 2‐methyl‐6‐nitrobenzoic anhydride (MNBA) and 4‐dimethylaminopyridine (DMAP), pretreated with 4 Å molecular sieves, led to the formation of the macrolactones with high yield (77–86 %), without side products and the need of HPLC . Notably, these cyclizations represent the most efficient methods for macrolide formation of the archazolids reported so far. The reported ring closing methods for the archazolids are so far a HWE macrocyclization (Arch A: 44 %), a Hoye relay ring‐closing metathesis (Arch B: 27 %), a Heck coupling (Arch B: 60 %: diastereomeric mixture) and a RCM reaction (Arch F: 49 %). Finally, global deprotection of the secondary TBS groups successfully afforded the four targeted derivatives **5**–**8**. Similar to the C23 deprotection, removal of the secondary TBS groups was difficult (25‐40 %) and required prolonged reaction times as well as subsequent additions of HF‐pyr to realize full conversion.

Importantly, the choice of protecting groups on the two primary alcohols at C1 and C23 was found to be crucial for the successful synthesis of **5** and **7**. For these two analogues, carrying the C20–C21 double bond, the C23 hydroxy group, prone to elimination during the aldol‐condensation sequence, had to be equipped with a carefully chosen protecting group. The C1 protecting group had to be orthogonally deprotectable with respect to C7, C15 and C23, whereas C23 itself had to be deprotected without affecting the protection of C7 and C15.

As shown in Table 2, several strategies were evaluated. Primary attempts with a benzoic ester functionality (entry 1) as protecting group led to a formation of the C18–C23 triene during the DBU‐mediated elimination. The aldol condensation sequence with PMB as R^2^ (entry 2) led to the desired diene with good yield. Deprotection occurred with 2,3‐dichloro‐5,6‐dicyano‐1,4‐benzoquinone (DDQ); however, only low yields were obtained, and oxidation at C17 was also observed. Attempts to reduce this group at a later stage of the synthesis were also carried out but could only be realized in low yield. The other variable on the molecule was the protecting group at C1. Removal of the TBDPS group to directly introduce the carbonate functionality (entry 3) led to degradation of the ketone during the aldol reaction. Similar degradation was observed with an acetate group as R^1^ (entry 4). The best combination was found to be a TES group as R^1^ and a TBS group as R^2^ (entry 5). Indeed, the TBS group suppressed further elimination along the 22,23‐bond during the aldol‐condensation sequence and the TES group was selectively cleaved in the presence of three TBS groups with high yield. After oxidation at C1, the primary R^2^‐TBS ether could be successfully removed without affecting the two secondary TBS groups using a diluted solution of HF‐pyr.

**Table 2:** Crucial protecting groups choice for the precursors to **5** and **7**.

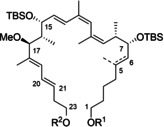			
	Protecting groups	Aldol condensation	R^1^/R^2^ deprotection
1	R1=TBDPS, R^2^=Bz	elimination	/
2	R^1^=TBDPS, R^2^=PMB	61 %	79 %/31 %
3	R^1^=CO_2_Me, R^2^=TBS	degradation	/
4	R^1^=Ac, R^2^=TBS	degradation	/
5	R^1^=TES, R^2^=TBS	60 %	94 %/42 %

All four new analogues **5**–**8** retained antiproliferative activities against 1321 N1 astrocytoma cells in the low‐nanomolar range similar to the parent natural product archazolid F (Table 3). However, they did not reach the sub‐nanomolar potency of archazolog **4**. Macrolactones **5**–**8** also showed similar human P2X3 receptor inhibition as compared to **4**. Our results demonstrate that removal of the (3,4), (5,6) and (20,21) double bonds as well as the C‐5 methyl group are well tolerated with almost no change in activities in these assays. These data confirm and refine our pharmacophore model and demonstrate that the overall structure may be further simplified without loss of biological activity.

**Table 3:** Biological data of novel analogues **5**–**8** in comparison to archazolid F (**3**) and archazolog **4**.

	3	4	5	6	7	8
Growth inhibition of 1321 N1 astrocytoma cells IC_50_±SEM [nM]	4.51±0.51	0.757±0.121	12.2±2.9	19.6±4.0	9.65±1.48	17.4±1.30
Human P2X3 inhibition IC_50_± SEM [μM]	0.438± 0.144	1.31±0.19	2.46±0.46	1.19±0.18	1.02±0.24	1.87±0.03
Affinity for the human adenosine A_3_ receptor *K*_i_±SEM [nM]	859±75	690±39	539±44	436±111	>1000	>1000
HLE inhibition *K*_i_±SEM [μM]	0.830±0.134	5.85±0.16	5.01±0.79	13.3±1.5	5.78±0.65	8.18±1.01

In contrast, the modifications addressed within this study did influence the affinity to the A_3_ adenosine receptor. In detail, the (5,6)‐olefin in combination with the appending methyl group was crucial for receptor interaction, as analogues **7** and **8** lacking this functional pattern were inactive. In contrast, new analogues **5** and **6**, retaining these structural features were still potent and even exhibited slightly better affinity as compared to archazolid F. These results are in agreement with an earlier study^6^ demonstrating that also slight variations in the C2–C3 region had a profound biological effect on this target. In summary, these results suggest that the eastern part of the archazolids is involved in A_3_ adenosine receptor binding. Regarding human leukocyte elastase (HLE), the new archazologs retained moderate inhibitory potency at this enzyme, but were weaker than archazolid F.

## Conclusions

In conclusion, we have reported the design and synthesis of four novel partially saturated archazolid derivatives and their biological evaluation. The design of these derivatives is based on previous SAR studies and pharmacophore analysis suggesting the archazolids’ binding site to be located on the top part of the macrolactone. The modifications were focused on the C3–C4, C5–C6 and C20–C21 double bonds as well as the C5 methyl group. The synthesis relied on a scalable and convenient approach to the northern part utilizing an olefination and aldol methodology as well as a coupling with various southern fragments using a highly stereoselective aldol condensation sequence. We report for the first time the implementation of a macrolactonization strategy to close the archazolid 24‐membered ring without formation of any side product such as dimers. Further insights into the archazolids’ pharmacophore were obtained after biological assessment of these new analogues. Indeed, derivatives **5**–**8** retained potent antiproliferative activities in the nanomolar range, similar to the parent natural product archazolid F but weaker than archazolog **4**. The modifications of these analogues were well tolerated by the P2X3 receptor and HLE as demonstrated in inhibition assays suggesting that further simplifications might be allowed. However, the results of the A_3_‐adenosine receptor binding assays showed that modifications in the C3–C6 area led to a drop in potency suggesting the crucial role of this pattern for receptor interaction. The developed synthetic approach allowed easy access to simplified archazolid derivatives and could be used to further develop this promising novel class of potent anticancer drugs.

## Experimental Section

**General procedures**. All reagents were purchased from commercial suppliers (Sigma‐Aldrich, TCI, Acros, Alfa Aesar) in the highest purity grade available and used without further purification. Anhydrous solvents (CH_2_Cl_2_, Et_2_O, THF, and toluene) were obtained from a solvent drying system MB SPS800 (MBrain) and stored over molecular sieves (4 Å). The reactions in which dry solvents were used were performed under an argon atmosphere in flame‐dried glassware, which had been flushed with argon unless stated otherwise. The reagents were handled using standard Schlenk techniques.

Thin‐layer chromatography monitoring was performed with silica gel 60 F_254_ precoated polyester sheets (0.2 mm silica gel, Macherey‐Nagel) and visualized using UV light and staining with a solution of CAM (1.0 g Ce(SO_4_)_2_, 2.5 g (NH_4_)_6_Mo_7_O_24_, 8 mL conc. H_2_SO_4_ in 100 mL H_2_O) and subsequent heating.

Semipreparative and analytical HPLC analyses were performed on Knauer Wissenschaftliche Gerate GmbH systems. The solvents for HPLC were purchased in HPLC grade. The products were detected by their UV absorption at 230 or 254 nm, respectively. All NMR spectra were recorded on Bruker spectrometers with operating frequencies of 125, 150, 500, 600, and 700 MHz in deuterated solvents obtained from Deutero. Spectra were measured at room temperature unless stated otherwise, and chemical shifts are reported in parts per million relative to (Me)_4_Si and were calibrated to the residual signal of undeuterated solvents.^29^ For full assignment of ^1^H and ^13^C signals of the final products, see the supporting information section. Optical rotations were measured with a PerkinElmer 341 polarimeter in 10 mm cuvette and are uncorrected. High‐resolution mass spectroscopy (HRMS) spectra were recorded on a Thermo LTQ Orbitrab Velos mass spectrometer.

**General method A: Paterson aldol reaction**. To a solution of chlorodicyclohexylborane (1.00 equiv) in Et_2_O at −78 °C, was added DMEA (2.0 equiv) followed by ketone **16** (1.00 equiv) in Et_2_O. The reaction was stirred for 2 h at 0 °C then cooled down again at −78 °C. The aldehyde (1.10 equiv) in Et_2_O was added. The mixture was stirred for 1 h at −78 °C and then stored at −20 °C overnight. The reaction was quenched at 0 °C with MeOH, pH 7 buffer and H_2_O_2_ (2 : 2 : 1) and stirred for 1.5 h at room temperature. After separation of the organic phase, the aqueous phase was extracted with CH_2_Cl_2_. The combined organic layers were dried over MgSO_4_, evaporated *in vacuo* and purified by column chromatography.

**Ketone 17**: Method A with chlorodicyclohexyl borane (10.1 mL, 10.1 mmol), DMEA (1.45 mL, 13.4 mmol) in Et_2_O (55 mL), ketone **16** (1.43 g, 6.69 mmol) in Et_2_O (50 mL) and aldehyde **15** (2.86 g, 7.35 mmol) in Et_2_O (4 mL). Work‐up MeOH (10 mL), buffer (pH 7, 10 mL), H_2_O_2_ (5 mL) and CH_2_Cl_2_ (3×50 mL). Chromatography (SiO_2_, CH/EtOAc, 10 : 1 to 5 : 1) gave **17** (3.23 g, 5.50 mmol, 82 %, *dr*>20 : 1). *R*_f_=0.31 (SiO_2_, CH/EtOAc, 5 : 1); [*α*]20D =+18.0° (*c*=0.44, CHCl_3_); ^1^H NMR (500 MHz, CDCl_3_): δ [ppm]=8.13–8.10 (m, 2H), 7.70–7.67 (m, 4H), 7.60 (ddt, *J*=7.9, 7.0, 1.3 Hz, 1H), 7.49–7.37 (m, 8H), 5.48 (qd, *J*=7.0, 1.6 Hz, 1H), 5.13 (dq, *J*=9.3, 1.3 Hz, 1H), 4.60 (td, *J*=9.0, 4.3 Hz, 1H), 3.68 (t, *J*=5.9 Hz, 2H), 2.89 (dq, *J*=8.6, 7.1 Hz, 1H), 2.02 (d, *J*=4.3 Hz, 2H), 1.70 (d, *J*=1.3 Hz, 3H), 1.59 (dd, *J*=7.0, 1.2 Hz, 3H), 1.56–1.48 (m, 4H), 1.15 (d, *J*=7.1 Hz, 3H), 1.07 (d, *J*=1.5 Hz, 9H); ^13^C NMR (176 MHz, CDCl_3_): δ [ppm]=211.3, 165.9, 140.9, 135.6, 134.1, 133.6, 129.8, 129.6, 128.5, 127.6, 125.1, 75.0, 70.4, 63.7, 60.4, 48.9, 39.3, 32.1, 26.9, 23.9, 21.1, 19.2, 16.8, 15.6, 14.2; HRMS (ESI+) calcd for C_36_H_46_O_5_SiNa^+^ [*M*+Na]^+^: 609.3007; found: 609.3007.

**Ketone 31**: Method A with chlorodicylohexylborane (8.70 mL, 8.70 mmol), DMEA (1.26 mL, 11.6 mmol) in Et_2_O (45 mL), ketone **16** (1.20 g, 5.82 mmol) in Et_2_O (45 mL) and aldehyde **30** (2.63 g, 7.00 mmol) in Et_2_O (3.5 mL). Work‐up MeOH (10 mL), buffer (pH 7, 10 mL), H_2_O_2_ (5 mL) and CH_2_Cl_2_ (3×50 mL). Chromatography (SiO_2_, CH/EtOAc, 10 : 1 to 5 : 1) gave **31** (1.80 g, 3.12 mmol, 54 %, *dr* >20 : 1). *R*_f_=0.34 (SiO_2_, CH/EtOAc, 4 : 1); [*α*]20D =+ 25.2° (*c*=0.31, CHCl_3_); ^1^H NMR (700 MHz, CDCl_3_): δ [ppm]=8.13–8.08 (m, 2H), 7.71–7.66 (m, 4H), 7.63–7.59 (m, 1H), 7.50–7.38 (m, 8H), 5.46 (q, *J*=7.1 Hz, 1H), 3.77 (ddd, *J*=9.7, 7.0, 2.5 Hz, 1H), 3.67 (t, *J*=6.5 Hz, 2H), 2.88 (p, *J*=7.2 Hz, 1H), 1.59 (d, *J*=7.1 Hz, 3H), 1.57–1.55 (m, 2H), 1.52 (tq, *J*=7.9, 2.8, 2.3 Hz, 2H), 1.42–1.31 (m, 6H), 1.29 (d, *J*=7.2 Hz, 3H), 1.06 (s, 9H); ^13^C NMR (176 MHz, CDCl_3_): δ [ppm]=212.1, 165.9, 135.6, 134.2, 133.4, 129.8, 129.5, 129.4, 128.5, 63.9, 60.4, 48.2, 34.5, 32.5, 29.3, 26.9, 25.8, 25.5, 15.9, 14.6; HRMS (ESI+) calcd for C_35_H_46_O_5_SiNa^+^ [*M*+Na]^+^: 597.3307; found: 597.3007.

**General method B: TBS protection, LiBH_4_ reduction and glycol cleavage**: To a stirred solution of *β*‐hydroxyketone (1.00 equiv) in CH_2_Cl_2_ at −78 °C was added 2,6‐lutidine (2.00 equiv) and TBS ⋅ OTf (1.50 equiv). The reaction was stirred for 1.5 h and quenched with a saturated solution of NaHCO_3_ at 0 °C. After separation of the organic layer, the aqueous layer was extracted with CH_2_Cl_2_. The organic layers were combined, dried over MgSO_4_ and evaporated *in vacuo*. The crude product was purified by column chromatography.

To a solution of protected alcohol (1.00 equiv) in THF at −78 °C was added LiBH_4_ (15.0 equiv) in one portion. After stirring 2 h at −78 °C, the mixture was stirred 3 days at room temperature. At 0 °C, water was added followed by careful addition of a saturated solution of NH_4_Cl. The mixture was poured to a mixture of water and Et_2_O (1 : 1). After separation of the organic layer, the aqueous layer was extracted with Et_2_O. The organic layers were combined, dried MgSO_4_ and evaporated *in vacuo*. The residue was purified by column chromatography.

To a solution of diol (1.00 equiv) in dioxane and water (2 : 1) at 0 °C was added NaIO_4_ (2.50 equiv) portionwise. The reaction mixture was vigorously stirred overnight then diluted with CH_2_Cl_2_, and the reaction was quenched with water. After separation of the organic layer, the aqueous layer was extracted with CH_2_Cl_2_. The organic layers were combined, dried over MgSO_4_ and evaporated *in vacuo*. The residue was purified by column chromatography.

**Aldehyde 18**: Method B with *β*‐hydroxyketone (3.23 g, 5.50 mmol), 2,6‐lutidine (1.26 mL,10.9 mmol), TBSOTf (1.88 mL, 8.17 mmol) in CH_2_Cl_2_ (120 mL). Work‐up NaHCO_3_ (80 mL) and CH_2_Cl_2_ (80 mL). Chromatography (SiO_2_, CH/EtOAc, 10 : 1) gave TBS‐protected alcohol (3.64 g, 94 %). Protected alcohol (3.64 g, 5.19 mmol), LiBH_4_ (1.68 g, 77.1 mmol) in THF (120 mL). Work‐up H_2_O (40 mL), NH_4_Cl (5 mL) and Et_2_O/H_2_O (1 : 1, 100 mL). Chromatography (SiO_2_, CH/EtOAc, 4 : 1) gave the diol (3.01 g, 98 %, *dr*=4 : 1). Diol (3.01 g, 5.09 mmol), NaIO_4_ (2.68 g, 12.5 mmol) in dioxane /water (120 mL). Work‐up water (50 mL) and CH_2_Cl_2_ (3×100 mL). Chromatography (SiO_2_, CH/EtOAc, 9 : 1) gave **18** (2.33 g 4.22 mmol, 83 %). *R*_f_=0.65 (SiO_2_, CH/EtOAc, 5 : 1); [*α*]20D =‐17.4° (*c*=0.39, CHCl_3_); ^1^H NMR (700 MHz, CD_2_Cl_2_): δ [ppm]=δ 9.73 (d, *J*=2.9 Hz, 1H), 7.68–7.65 (m, 4H), 7.44–7.41 (m, 2H), 7.38 (ddt, *J*=8.1, 6.7, 1.1 Hz, 4H), 5.16 (dp, *J*=9.1, 1.3 Hz, 1H), 4.58–4.52 (m, 1H), 3.68 (t, *J*=6.0 Hz, 2H), 2.42–2.35 (m, 1H), 2.06–1.97 (m, 2H), 1.65 (d, *J*=1.4 Hz, 3H), 1.60–1.50 (m, 7H), 1.04 (s, 9H), 0.94 (d, *J*=7.0 Hz, 3H), 0.85 (d, *J*=2.7 Hz, 9H), −0.02 (s, 3H), −0.04 (s, 3H); ^13^C NMR (176 MHz, CD_2_Cl_2_): δ [ppm]=204.7, 137.8, 135.5, 134.1, 129.5, 127.6, 126.4, 71.2, 63.7, 53.5, 39.2, 32.2, 26.6, 25.5, 23.9, 19.1, 17.9, 16.5, 10.3, −4.2, −5.4; HRMS (ESI+) calcd for C_33_H_52_O_4_Si_2_Na^+^ [*M*+Na]^+^: 575.3347; found: 575.3347.

**Aldehyde 32**: Method B with *β*‐hydroxyketone (888 mg, 1.54 mmol), 2,6‐lutidine (0.36 mL,3.08 mmol), TBSOTf (0.53 mL, 2.31 mmol) in CH_2_Cl_2_ (50 mL). Work‐up NaHCO_3_ (25 mL), CH_2_Cl_2_ (25 mL). Chromatography (SiO_2_, CH/EtOAc, 10 : 1) gave TBS‐protected alcohol (996 mg, 85 %). Protected alcohol (885 mg, 1.33 mmol), LiBH_4_ (340 mg, 15.7 mmol) in THF (40 mL). Work‐up H_2_O (15 mL), NH_4_Cl (2 mL) and Et_2_O/H_2_O (1 : 1, 40 mL). Chromatography (SiO_2_, CH/EtOAc, 4 : 1) gave the diol (750 mg, quant., *dr*=4 : 1). Diol (750 mg, 1.33 mmol), NaIO_4_ (683 mg, 3.20 mmol) in dioxane /water (30 mL). Work‐up water (20 mL) and CH_2_Cl_2_ (3×20 mL). Chromatography (SiO_2_, CH/EtOAc, 9 : 1) gave **32** (583 mg, 1.07 mmol, 85 %). *R*_f_=0.66 (SiO_2_, CH/EtOAc, 5 : 1); ${\left[\alpha \right]}_{D}^{20}$ = −22.6° (*c*=0.35, CHCl_3_); ^1^H NMR (500 MHz, CDCl_3_): δ [ppm]=9.74 (d, *J*=2.3 Hz, 1H), 7.70–7.63 (m, 4H), 7.47–7.33 (m, 6H), 3.91 (q, *J*=5.5 Hz, 1H), 3.65 (t, *J*=6.4 Hz, 2H), 2.49 (ddd, *J*=7.1, 4.9, 2.3 Hz, 1H), 1.59–1.50 (m, 6H), 1.44 (ddd, *J*=15.4, 9.5, 4.2 Hz, 1H), 1.38–1.23 (m, 7H), 1.07 (d, *J*=7.0 Hz, 3H), 1.04 (s, 9H), 0.88 (s, 9H), 0.06 (d, *J*=4.0 Hz, 6H); ^13^C NMR (125 MHz, CDCl_3_): δ [ppm]=205.2, 135.6, 134.2, 129.5, 127.6, 73.5, 63.9, 51.1, 34.8, 32.5, 29.5, 26.9, 25.8, 24.8, 19.2, 18.1, 10.5, −4.2, −4.7; HRMS (ESI+) calcd for C_34_H_56_O_3_Si_2_K^+^ [*M*+K]^+^: 579.3087; found: 579.3090.

**General method C: Still‐Gennari olefination**. To a solution of [18]crown‐6 (2.30 equiv,) and phosphonate **19** (1.40 equiv) in THF at −78 °C was added KHMDS (1.30 equiv) over 10 min. The reaction was stirred for 30 min then the aldehyde (1.00 equiv) in THF was added dropwise and the reaction was stirred for another 2 h at −78 °C. The reaction was quenched with a saturated solution of NaHCO_3_ at 0 °C. After separation of the organic layer, the aqueous layer was extracted with CH_2_Cl_2_. The organic layers were combined, dried over MgSO_4_, evaporated *in vacuo* and purified by column chromatography.

**Ester 20**: Method C with [18]crown‐6 (2.52 g, 9.55 mmol), **19** (1.93 g, 5.82 mmol), KHMDS (10.8 mL, 8.40 mmol) in THF (100 mL), aldehyde **18** (2.30 g, 4.15 mmol) in THF (4 mL). Work‐up NaHCO_3_ (100 mL) and CH_2_Cl_2_ (240 mL). Chromatography (SiO_2_, CH/EtOAc, 9 : 1) gave **20** (2.38 g, 3.82 mmol, 92 %, *dr* > 20 : 1). *R*_f_=0.56 (SiO_2_, CH/EtOAc, 10 : 1); [*α*]20D =+9.1° (*c*=0.32, CHCl_3_); ^1^H NMR (500 MHz, CDCl_3_): δ [ppm]=7.75–7.66 (m, 4H), 7.49–7.38 (m, 6H), 5.84 (dq, *J*=10.1, 1.4 Hz, 1H), 5.09 (dq, *J*=9.1, 1.3 Hz, 1H), 4.21 (dd, *J*=9.0, 5.9 Hz, 1H), 3.72 (s, 3H), 3.68 (t, *J*=6.0 Hz, 2H), 3.26–3.15 (m, 1H), 1.98 (t, *J*=7.3 Hz, 2H), 1.90 (d, *J*=1.4 Hz, 3H), 1.60 (d, *J*=1.3 Hz, 3H), 1.58–1.48 (m, 4H), 1.07 (s, 9H), 0.96 (dd, *J*=6.9, 2.6 Hz, 3H), 0.88 (s, 9H), −0.02 (s, 3H), −0.04 (s, 3H); ^13^C NMR (125 MHz, CDCl_3_): δ [ppm]=146.0, 135.8, 135.6, 134.1, 129.5, 127.6, 127.3, 126.4, 73.0, 63.7, 51.1, 40.8, 39.3, 32.2, 26.9, 25.8, 24.0, 21.0, 19.2, 18.1, 16.6, 16.1, −4.1, −4.9; HRMS (ESI+) calcd for C_37_H_58_O_4_Si_2_Na^+^ [*M*+Na]^+^: 645.3766; found: 645.3766.

**Ester 22**: Method C with [18]crown‐6 (2.13 g, 8.14 mmol), **19** (1.64 g, 4.96 mmol), KHMDS (9.2 mL, 4.6 mmol) in THF (100 mL), aldehyde **21** (2.12 g, 3.54 mmol) in THF (4 mL). Work‐up NaHCO_3_ (100 mL) and CH_2_Cl_2_ (240 mL). Chromatography (SiO_2_, CH/EtOAc, 9 : 1) gave **22** (2.17 g, 3.27 mmol, 93 %, *dr* > 20 : 1). *R*_f_ *=*0.56 (SiO_2_, CH/EtOAc, 10 : 1); [*α*]20D =+28.1° (*c=*0.31, CHCl_3_); ^1^H NMR (500 MHz, CDCl_3_): δ [ppm]=7.68–7.66 (m, 4H), 7.42–7.36 (m, 6H), 6.41–6.38 (m, 1H), 5.09 (ddt, *J*=11.8, 9.0, 1.4 Hz, 2H), 4.10 (dd, *J*=9.0, 5.9 Hz, 1H), 3.70 (s, 3H), 3.66 (t, *J*=6.1 Hz, 2H), 2.40 (dq, *J*=10.0, 6.5 Hz, 1H), 1.97–1.93 (m, 5H), 1.77–1.74 (m, 3H), 1.58 (d, *J*=1.3 Hz, 3H), 1.55–1.43 (m, 4H), 1.04 (d, *J*=1.5 Hz, 9H), 0.86–0.83 (m, 13H), −0.01 (s, 3H), −0.04 (s, 3H); ^13^C NMR (125 MHz, CDCl_3_): δ [ppm]=169.8, 135.6, 134.1, 133.5, 131.4, 129.5, 127.9, 127.6, 127.3, 73.1, 63.7, 51.4, 40.6, 39.3, 62.2, 26.9, 25.8, 24.0, 22.2, 21.2, 19.2, 18.2, 16.6, 16.0, −4.3, −4.9; HRMS (ESI+) calcd for C_40_H_62_O_4_Si_2_Na^+^ [*M*+Na]^+^: 686.4079; found: 686.4097.

**Ester 33**: Method C with [18]crown‐6 (674 mg, 2.55 mmol), **19** (516 mg, 1.55 mmol), KHMDS (2.9 mL, 1.44 mmol) in THF (20 mL), aldehyde **32** (594 mg, 1.11 mmol) in THF (2 mL). Work‐up NaHCO_3_ (30 mL) and CH_2_Cl_2_ (100 mL). Chromatography (SiO_2_, CH/EtOAc, 9 : 1) gave **33** (610 mg, 1.00 mmol, 91 %, *dr* > 20 : 1). *R*_f_=0.66 (SiO_2_, CH/EtOAc, 5 : 1); [*α*]20D =+5.2° (*c=*0.33, CHCl_3_); ^1^H NMR (700 MHz, CDCl_3_): δ [ppm]=7.69–7.68 (m, 4H), 7.45–7.42 (m, 2H), 7.41–7.38 (m, 4H), 5.94 (dq, *J*=10.1, 1.4 Hz, 1H), 3.73 (s, 3H), 3.66 (t, *J*=6.5 Hz, 2H), 3.55 (td, *J*=6.1, 3.6 Hz, 1H), 3.30 (dqd, *J*=10.4, 6.8, 3.5 Hz, 1H), 1.93 (d, *J*=1.4 Hz, 3H), 1.40–1.18 (m, 10H), 1.06 (s, 9H), 1.00 (d, *J*=6.8 Hz, 3H), 0.92 (s, 9H), 0.07 (s, 3H), 0.06 (s, 3H); ^13^C NMR (176 MHz, CDCl_3_): δ [ppm]=168.5, 144.8, 135.6, 134.2, 129.5, 127.6, 126.6, 75.7, 64.0, 51.2, 38.0, 35.1, 32.6, 29.6, 26.9, 26.0, 25.8, 25.5, 21.1, 19.2, 18.2, 17.0, −4.2, −4.5; HRMS (ESI+) calcd for C_36_H_58_O_4_Si_2_Na^+^ [*M*+Na]^+^: 633.3766, found : 633.3763.

**Ester 35**: Method C with [18]crown‐6 (536 mg, 2.05 mmol), **19** (416 mg, 1.25 mmol), KHMDS (2.3 mL, 1.16 mmol) in THF (20 mL), aldehyde **34** (520 mg, 0.96 mmol) in THF (2 mL). Work‐up NaHCO_3_ (30 mL) and CH_2_Cl_2_ (100 mL). Chromatography (SiO_2_, CH/EtOAc, 9 : 1) gave **35** (510 mg, .078 mmol, 87 %, *dr* > 20 : 1). *R*_f_=0.55 (SiO_2_, CH/EtOAc, 20 : 1); [*α*]20D =+0.9° (*c=*0.22, CHCl_3_); ^1^H NMR (700 MHz, CDCl_3_): δ [ppm]=7.67 (dt, *J*=6.7, 1.5 Hz, 4H), 7.43–7.36 (m, 6H), 6.38–6.36 (m, 1H), 5.16 (dp, *J*=9.9, 1.6 Hz, 1H), 3.70 (s, 3H), 3.65 (t, *J*=6.5 Hz, 2H), 3.44 (dt, *J*=7.0, 4.3 Hz, 1H), 2.44 (dqd, *J*=13.7, 6.8, 3.9 Hz, 1H), 1.97 (d, *J*=1.6 Hz, 3H), 1.79–1.77 (m, 3H), 1.57–1.54 (m, 2H), 1.35–1.29 (m, 4H), 1.28–1.19 (m, 3H), 1.17–1.12 (m, 1H), 1.04 (s, 9H), 0.90–0.88 (m, 12H), 0.01 (d, *J*=3.0 Hz, 6H); ^13^C NMR (176 MHz, CDCl_3_): δ [ppm]=169.4, 136.1, 134.2, 131.9, 129.5, 128.4, 127.6, 75.8, 64.0, 51.4, 35.8, 33.6, 32.6, 29.7, 26.9, 26.0, 25.9, 22.5, 21.1, 19.2, 18.1, 15.9, −4.3, −4.5; HRMS (ESI+) calcd for C_39_H_62_O_4_Si_2_Na^+^ [*M*+Na]^+^: 637.4079; found: 673.4079.

**General method D: Red‐Ox sequence from ester to aldehyde**. To a solution of ester (1.00 equiv) in CH_2_Cl_2_ at −78 °C was added DIBAL‐H (3.00 equiv) dropwise. The mixture was stirred for 1 h and warmed up to 0 °C for 45 min. CH_2_Cl_2_ was added followed by H_2_O_2_, a 3 M aqueous solution of NaOH and H_2_O (1 : 1 : 2.5). After stirring 15 min at room temperature, MgSO_4_ was added and the mixture was stirred an additional 15 min. After filtration, the solvent was removed *in vacuo*.

*D1= With MnO_2_*. The crude product was directly diluted in CH_2_Cl_2_ and MnO_2_ (20.0 equiv) was added. The reaction was stirred overnight at room temperature. The solution was filtered through celite and the solvent was evaporated *in vacuo*. The residue was purified by column chromatography.

**Aldehyde 21**: Method D1 with ester **20** (2.38 g, 3.82 mmol), DIBAL‐H (11.4 mL, 11.4 mmol), in CH_2_Cl_2_ (50 mL). Work‐up CH_2_Cl_2_ (50 mL), H_2_O_2_ (0.45 mL), 3 M NaOH (0.45 ml), H_2_O (1.1 mL). Crude product and MnO_2_ (6.64 g, 76.4 mmol) in CH_2_Cl_2_ (40 mL). Chromatography (SiO_2_, CH/EtOAc, 9 : 1) gave **21** (2.12 g, 3.54 mmol, 94 % over 2 steps). *R*_f_=0.56 (SiO_2_, CH/EtOAc, 10 : 1); [*α*]20D =+11.8° (*c=*0.51, CHCl_3_); ^1^H NMR (500 MHz, CDCl_3_): δ [ppm]=10.04 (d, *J*=0.5 Hz, 1H), 7.69–7.63 (m, 4H), 7.45–7.34 (m, 6H), 6.34 (dq, *J*=10.9, 1.3 Hz, 1H), 5.06 (dq, *J*=9.3, 1.4 Hz, 1H), 4.19–4.13 (m, 1H), 3.66 (t, *J*=6.0 Hz, 2H), 3.17 (dp, *J*=10.7, 6.7 Hz, 1H), 2.02–1.95 (m, 2H), 1.77 (d, *J*=1.4 Hz, 3H), 1.62 (d, *J*=1.3 Hz, 3H), 1.54–1.46 (m, 4H), 1.04 (s, 9H), 1.00 (d, *J*=6.7 Hz, 3H), 0.82 (d, *J*=2.6 Hz, 9H), −0.02 (s, 3H), −0.04 (s, 3H); ^13^C NMR (125 MHz, CDCl_3_): δ [ppm]=192.1, 152.6, 136.0, 135.9, 135.5, 134.1, 129.5, 127.6, 127.0, 73.0, 63.6, 39.3, 38.4, 32.2, 26.9, 25.7, 23.9, 19.2, 18.1, 17.2, 16.8, 16.6, −4.1, −4.9; HRMS (ESI+) calcd for C_36_H_56_O_3_Si_2_Na^+^ [*M*+Na]^+^: 615.3660; found: 615.3664.

**Aldehyde 23**: Method D1 with ester **22** (2.17 g, 3.27 mmol), DIBAL‐H (9.81 mL, 9.81 mmol), in CH_2_Cl_2_ (50 mL). Work‐up CH_2_Cl_2_ (50 mL), H_2_O_2_ (0.40 mL), 3 M NaOH (0.40 ml), H_2_O (1.0 mL). Crude product and MnO_2_ (5.69 g, 65.4 mmol) in CH_2_Cl_2_ (40 mL). Chromatography (SiO_2_, CH/EtOAc, 9 : 1) gave **23** (1.96 g, 3.09 mmol, 95 % over 2 steps). *R*_f_=0.61 (SiO_2_, CH/EtOAc, 20 : 1); [*α*]20D =+11.3° (*c=*0.77, CHCl_3_); ^1^H NMR (500 MHz, CDCl_3_): δ [ppm]=9.90 (s, 1H), 7.70–7.64 (m, 5H), 7.44–7.35 (m, 7H), 6.92 (dd, *J*=2.3, 1.2 Hz, 1H), 5.40 (dq, *J*=10.2, 1.4 Hz, 1H), 5.03 (dq, *J*=9.0, 1.3 Hz, 1H), 4.09 (dd, *J*=8.9, 6.2 Hz, 1H), 3.66 (t, *J*=6.1 Hz, 2H), 2.35–2.27 (m, 1H), 1.98–1.94 (m, 2H), 1.88 (q, *J*=2.1, 1.6 Hz, 2H), 1.81 (d, *J*=1.4 Hz, 2H), 1.56 (d, *J*=1.3 Hz, 2H), 1.53–1.45 (m, 3H), 1.05–1.04 (m, 9H), 0.87–0.83 (m, 12H) −0.01 (s, 3H), −0.04 (s, 3H); ^13^C NMR (125 MHz, CDCl_3_): δ [ppm]=193.4, 147.0, 136.2, 135.8, 135.6, 134.1, 129.5, 127.6, 127.4, 73.2, 63.7, 40.9, 39.3, 32.2, 26.9, 25.8, 25.0, 24.0, 19.2, 18.1, 16.6, 16.3, 15.9, −4.2, −4.9; HRMS (ESI+) calcd for C_39_H_60_O_3_Si_2_Na^+^ [*M*+Na]^+^: 655.3973; found: 655.3973.

**Aldehyde 25**: Method D1 with ester **24** (582 mg, 0.84 mmol), DIBAL‐H (2.50 mL, 2.50 mmol), in CH_2_Cl_2_ (10 mL). Work‐up CH_2_Cl_2_ (20 mL), H_2_O_2_ (0.1 mL), 3 M NaOH (0.1 ml), H_2_O (0.2 mL). Crude product and MnO_2_ (1.46 g, 16.8 mmol) in CH_2_Cl_2_ (6 mL). Chromatography (SiO_2_, CH/EtOAc, 9 : 1) gave **25** (540 mg, 0.82 mmol, 98 % over 2 steps). *R*_f_=0.50 (SiO_2_, CH/EtOAc, 10 : 1); [*α*]20D =+17.0° (*c=*0.37, CHCl_3_); ^1^H NMR (700 MHz, CDCl_3_): δ [ppm]=9.61 (d, *J*=7.9 Hz, 1H), 7.70–7.68 (m, 5H), 7.53 (dd, *J*=15.7, 0.8 Hz, 1H), 7.45–7.43 (m, 2H), 7.41–7.38 (m, 5H), 6.29 (dd, *J*=2.2, 1.2 Hz, 1H), 6.18 (ddt, *J*=15.7, 7.8, 0.7 Hz, 1H), 5.32 (dt, *J*=10.2, 1.5 Hz, 1H), 5.08–5.05 (m, 1H), 4.10 (dd, *J*=8.9, 6.2 Hz, 1H), 3.68 (t, *J*=6.1 Hz, 2H), 2.29 (dp, *J*=10.3, 6.8 Hz, 1H), 1.97 (t, *J*=7.4 Hz, 2H), 1.95 (d, *J*=1.3 Hz, 3H), 1.86–1.85 (m, 3H), 1.59 (dd, *J*=1.3, 0.7 Hz, 3H), 1.57–1.54 (m, 2H), 1.49 (qd, *J*=7.1, 3.4 Hz, 2H), 1.06 (d, *J*=0.6 Hz, 10H), 0.87 (d, *J*=0.6 Hz, 12H); ^13^C NMR (176 MHz, CD_2_Cl_2_): δ [ppm]=194.0, 150.8, 139.9, 135.8, 135.5, 134.8, 134.2, 131.7, 131.2, 129.5, 128.9, 127.6, 127.3, 73.1, 63.8, 40.7, 39.3, 32.2, 26.6, 25.6, 24.1, 24.0, 19.4, 19.1, 18.0, 16.4, 15.6, −4.5, −5.2; HRMS (ESI+) calcd for C_41_H_62_O_3_Si_2_Na^+^ [*M*+Na]^+^: 681.4130; found: 681.4130.

*D2= With DMP*. DMP (1.20 eq) was added to a solution of crude alcohol in DCM at 0 °C. The mixture was stirred for 1 to 3 h at room temperature and quenched with a saturated solution of NaHCO_3_/Na_2_S_3_O_3_ (2 : 1). After separation of the organic layer, the aqueous layer was extracted with DCM. The combined organic layers were dried over MgSO_4_, evaporated *in vacuo* and purified by column chromatography.

**Aldehyde 34**: Method D2 with ester **33** (620 mg, 1.01 mmol), DIBAL‐H (3.00 mL, 3.00 mmol), in CH_2_Cl_2_ (10 mL). Work‐up CH_2_Cl_2_ (20 mL), H_2_O_2_ (0.12 mL), 3 M NaOH (0.12 ml), H_2_O (0.30 mL). Crude product and DMP (517 mg, 1.21 mmol) in CH_2_Cl_2_ (10 mL). Work‐up NaHCO_3_/Na_2_S_3_O_3_ (30 mL) and DCM (60 mL). Chromatography (SiO_2_, CH/EtOAc, 9 : 1) gave **34** (525 mg, 0.96 mmol, 90 % over 2 steps). *R*_f_=0.52 (SiO_2_, CH/EtOAc, 20 : 1); [*α*]20D =+8.8° (*c=*0.26, CHCl_3_); ^1^H NMR (700 MHz, CDCl_3_): δ [ppm]=10.08 (d, *J*=0.5 Hz, 1H), 7.68–7.65 (m, 4H), 7.43–7.40 (m, 2H), 7.39–7.36 (m, 4H), 6.45 (dq, *J*=10.8, 1.3 Hz, 1H), 3.64 (t, *J*=6.4 Hz, 2H), 3.55 (td, *J*=5.7, 4.6 Hz, 1H), 3.33–3.27 (m, 1H), 1.79 (d, *J*=1.3 Hz, 3H), 1.56–1.53 (m, 4H), 1.46 (ddt, *J*=13.7, 10.4, 5.0 Hz, 1H), 1.40–1.31 (m, 3H), 1.30–1.21 (m, 4H), 1.06 (d, *J*=6.8 Hz, 3H), 1.04 (s, 9H), 0.88 (s, 9H); ^13^C NMR (176 MHz, CDCl_3_): δ [ppm]=191.6, 152.1, 135.6, 134.2, 129.5, 127.6, 75.6, 63.9, 35.6, 34.9, 32.5, 29.6, 26.9, 25.9, 25.8, 24.8, 19.2, 18.6, 18.1, 16.7, −4.2, −4.4; HRMS (ESI+) calcd for C_35_H_56_O_3_Si_2_Na^+^ [*M*+Na]^+^: 603.3660; found: 603.3663.

**Aldehyde 36**: Method D2 with ester **35** (507 mg, 0.78 mmol), DIBAL‐H (2.33 mL, 2.33 mmol), in CH_2_Cl_2_ (12 mL). Work‐up CH_2_Cl_2_ (15 mL), H_2_O_2_ (0.10 mL), 3 M NaOH (0.10 ml), H_2_O (0.20 mL). Crude product and DMP (396 mg, 0.93 mmol) in CH_2_Cl_2_ (10 mL). Work‐up NaHCO_3_/Na_2_S_3_O_3_ (15 mL) and DCM (45 mL). Chromatography (SiO_2_, CH/EtOAc, 9 : 1) gave **36** (416 mg, 0.67 mmol, 86 % over 2 steps). *R*_f_=0.52 (SiO_2_, CH/EtOAc, 20 : 1); [*α*]20D =+6.2° (*c=*0.26, CHCl_3_); ^1^H NMR (700 MHz, CDCl_3_): δ [ppm]=9.89 (s, 1H), 7.68–7.65 (m, 4H), 7.42–7.36 (m, 6H), 6.94 (dd, *J*=2.5, 1.3 Hz, 1H), 5.44 (dt, *J*=10.3, 1.5 Hz, 1H), 3.65 (t, *J*=6.5 Hz, 2H), 3.46–3.38 (m, 1H), 2.40 (ddd, *J*=10.5, 6.9, 3.9 Hz, 1H), 1.90 (dd, *J*=1.4, 0.8 Hz, 3H), 1.83 (d, *J*=1.5 Hz, 3H), 1.38–1.20 (m, 9H), 1.04 (s, 9H), 0.91 (d, *J*=6.8 Hz, 3H), 0.88 (s, 9H), −0.00 (d, *J*=7.1 Hz, 6H); ^13^C NMR (176 MHz, CDCl_3_): δ [ppm]=193.1, 146.8, 136.5, 135.6, 135.3, 134.2, 129.8, 129.5, 127.6, 75.8, 64.0, 38.5, 33.9, 32.6, 29.6, 25.9, 25.6, 25.1, 19.2, 18.1, 16.2, 15.8, −4.3, −4.5; HRMS (ESI+) calcd for C_38_H_60_O_3_Si_2_Na^+^ [*M*+Na]^+^: 643.3973; found: 643.3973.

**Aldehyde 38**: Method D2 with ester **37** (120 mg, 0.18 mmol), DIBAL‐H (.053 mL, 0.53 mmol), in CH_2_Cl_2_ (7 mL). Work‐up CH_2_Cl_2_ (15 mL), H_2_O_2_ (0.08 mL), 3 M NaOH (0.08 ml), H_2_O (0.15 mL). Crude product and DMP (90 mg, 0.21 mmol) in CH_2_Cl_2_ (4 mL). Work‐up NaHCO_3_/Na_2_S_3_O_3_ (12 mL) and DCM (30 mL) Chromatography (SiO_2_, CH/EtOAc, 20 : 1) gave **38** (416 mg, 0.67 mmol, 90 % over 2 steps). *R*_f_=0.52 (SiO_2_, CH/EtOAc, 20 : 1); [*α*]20D =+3.3° (*c=*0.24, CHCl_3_); ^1^H NMR (700 MHz, CDCl_3_): δ [ppm]=9.61 (dd, *J*=7.8, 5.5 Hz, 1H), 7.69–7.64 (m, 4H), 7.50–7.43 (m, 1H), 7.42–7.34 (m, 6H), 6.27 (s, 1H), 6.16 (dd, *J*=15.7, 7.8 Hz, 1H), 5.35 (dt, *J*=10.2, 1.5 Hz, 1H), 3.64 (t, *J*=6.4 Hz, 2H), 3.40 (dd, *J*=6.4, 4.0 Hz, 1H), 2.34 (ddd, *J*=10.5, 6.9, 3.8 Hz, 1H), 1.94 (d, *J*=1.4 Hz, 2H), 1.87–1.81 (m, 2H), 1.26 (dt, *J*=21.0, 11.2 Hz, 8H), 1.04 (s, 9H), 0.91 (d, *J*=6.8 Hz, 3H), 0.88 (d, *J*=2.7 Hz, 9H), 0.04—0.06 (m, 6H); ^13^C NMR (176 MHz, CDCl_3_): δ [ppm]=194.2, 150.7, 139.9, 135.6, 134.2, 134.0, 132.0, 131.5, 129.5, 129.1, 127.6, 75.9, 64.0, 38.5, 33.7, 32.6, 29.6, 26.9, 25.9, 24.5, 19.6, 19.2, 18.1, 15.8, −4.3, −4.6; HRMS (ESI+) calcd for C_40_H_62_O_3_Si_2_Na^+^ [*M*+Na]^+^: 669.4130; found: 669.4130.

**General method E: HWE olefination**. To a solution of trimethyl phosphonoacetate **13 c** (1.50 equiv) and DMPU (1.50 equiv) in THF at 0 °C was added *n*BuLi (1.40 equiv). The mixture was stirred for 30 min then the aldehyde (1.00 equiv) in THF was added dropwise. After stirring for 2 h at 0 °C, the reaction was stirred overnight at room temperature. The reaction was quenched with buffer pH 7 and H_2_O at 0 °C. After separation of the organic layer, the aqueous layer was extracted with Et_2_O. The organic layers were combined, dried over MgSO_4_, evaporated *in vacuo* and purified by column chromatography.

**Ester 24**: Method E with **13 c** (0.75 mL, 4.64 mmol), DMPU (0.56 mL, 4.64 mmol, *n*BuLi (2.7 mL, 4.33 mmol) and aldehyde **23** (1.96 g, 3.09 mmol) in THF (80 mL). Work‐up at pH 7 (50 mL) and Et_2_O (300 mL). Chromatography (SiO_2_, CH/EtOAc, 9 : 1) gave **24** (2.03 g, 2.94 mmol, 95 %). *R*_f_=0.53 (SiO_2_, CH/EtOAc, 20 : 1); [*α*]20D =+39.3° (*c=*0.41, CHCl_3_); ^1^H NMR (500 MHz, CDCl_3_): δ [ppm]=7.68–7.62 (m, 5H), 7.41–7.35 (m, 6H), 6.17 (td, *J*=1.5, 0.8 Hz, 1H), 5.86 (dd, *J*=15.8, 0.7 Hz, 1H), 5.23–5.17 (m, 1H), 5.05 (dq, *J=*9.2, 1.3 Hz, 1H), 4.08 (dd, *J*=9.0, 5.8 Hz, 1H), 3.74 (s, 3H), 3.66 (td, *J*=6.0, 2.5 Hz, 3H), 2.30–2.22 (m, 1H), 1.98–1.93 (m, 2H), 1.89 (d, *J*=1.4 Hz, 3H), 1.80 (dd, *J*=1.4, 0.7 Hz, 3H), 1.57 (d, *J*=1.3 Hz, 3H), 1.54–1.46 (m, 5H), 1.04 (d, *J*=2.0 Hz, 11H), 0.88–0.86 (m, 3H), 0.86–0.82 (m, 9H), −0.06 (s, 5H); ^13^C NMR (125 MHz, CDCl_3_): δ [ppm]=167.8, 143.3, 138.3, 135.5, 134.5, 134.1, 131.3, 131.2, 129.5, 127.6, 127.1, 117.8, 72.9, 63.7, 51.4, 40.8, 39.3, 62.2, 26.8, 25.8, 24.5, 24.0, 19.8, 19.2, 18.1, 16.6, 15.5, −4.3, −4.9; HRMS (ESI+) calcd for C_42_H_64_O_4_Si_2_Na^+^ [*M*+Na]^+^: 711.4235, found : 711.4238.

**Ester 37**: Method E with **13 c** (0.16 mL, 1.00 mmol), DMPU (0.12 mL, 1.00 mmol, *n*BuLi (0.58 mL, 0.94 mmol) and aldehyde **36** (416 mg, 0.67 mmol) in THF (15 mL). Work‐up at pH 7 (15 mL), Et_2_O (60 mL). Chromatography (SiO_2_, CH/EtOAc, 9 : 1) gave **37** (416 mg, 0.67 mmol, 89 %). *R*_f_=0.52 (SiO_2_, CH/EtOAc, 20 : 1); [*α*]20D =+40.4° (*c=*0.26, CHCl_3_); ^1^H NMR (700 MHz, CDCl_3_): δ [ppm]=7.68–7.65 (m, 4H), 7.61 (dd, *J*=15.8, 0.7 Hz, 1H), 7.43–7.36 (m, 6H), 6.15 (d, *J*=1.9 Hz, 1H), 5.87 (dd, *J*=15.8, 0.7 Hz, 1H), 5.29 (dt, *J*=10.3, 1.4 Hz, 1H), 3.74 (s, 3H), 3.64 (t, J=6.5 Hz, 2H), 3.39 (ddd, J=6.9, 4.8, 3.5 Hz, 1H), 2.32 (ddd, *J*=10.4, 6.9, 3.7 Hz, 1H), 1.89 (d, *J*=1.4 Hz, 3H), 1.81 (dd, *J*=1.4, 0.7 Hz, 3H), 1.59–1.54 (m, 2H), 1.38–1.18 (m, 9H), 1.04 (d, *J*=1.7 Hz, 9H), 0.92 (d, *J*=6.8 Hz, 3H), 0.87 (s, 9H), −0.02 (s, 3H), −0.03 (s, 3H); ^13^C NMR (176 MHz, CDCl_3_): δ [ppm]=167.7, 142.2, 138.0, 135.6, 134.2, 133.3, 131.6, 129.5, 127.6, 118.0, 75.9, 64.0, 51.5, 38.5, 33.5, 32.6, 29.6, 26.9, 26.0, 25.9, 24.6, 19.6, 19.2, 18.1, 15.5, −4.4, −4.6; HRMS (ESI+) calcd for C_41_H_64_O_4_Si_2_Na^+^ [*M*+Na]^+^: 699.4235; found: 699.4235.

**General method F: Ipc boron mediated aldol reaction and TBS protection**. (−)‐Ipc_2_BH (1.00 equiv) was dissolved in anhydrous hexane and cooled down at 0 °C. Triflic acid (1.00 equiv) was added dropwise and the mixture was stirred at room temperature until no Ipc_2_BH crystals were seen to afford a stock solution of triflate of 1.9 M. The stock solution (1.30 equiv) was diluted in CH_2_Cl_2_ and cooled down to −78 °C. DIEA (3.00 equiv) was added dropwise followed by diethylketone **26** (1.40 equiv). The reaction mixture was stirred for 3 h at this temperature. Then the aldehyde (1.00 equiv) in CH_2_Cl_2_ was added, the reaction was stirred for 1 h at −78 °C and stored overnight at −20 °C. Buffer (pH 7), MeOH and H_2_O_2_ (2 : 2 : 1) were added, and the solution was stirred for 1 h at room temperature. After separation of the organic layer, the aqueous layer was extracted with CH_2_Cl_2_. The organic layers were combined, dried over MgSO_4_, evaporated *in vacuo* and purified by column chromatography.

To a stirred solution of *β*‐hydroxyketone (1.00 equiv) in CH_2_Cl_2_ at −78 °C was added 2,6‐lutidine (2.00 equiv) and TBSOTf (1.50 equiv). The reaction was stirred for 1.5 h and quenched with a saturated solution of NaHCO_3_ at 0 °C. After separation of the organic layer, the aqueous layer was extracted with CH_2_Cl_2_. The organic layers were combined, dried over MgSO_4_ and evaporated *in vacuo*. The crude product was purified by column chromatography.

**Ketone 27**: Method F with TfOH (336 μL, 3.81 mmol), Ipc_2_BH (1.09 g, 3.81 mmol) in hexane (0.88 mL). Triflate stock solution (0.55 mL, 1.05 mmol), DIEA (360 μL, 2.10 mmol), diethylketone **26** (100 μL, 0.98 mmol) and aldehyde **25** (460 mg, 0.70 mmol) in CH_2_Cl_2_ (8 mL). Work‐up at pH 7 buffer (4 mL), MeOH (4 mL), H_2_O_2_ (2 mL) and CH_2_Cl_2_ (30 mL). Chromatography (SiO_2_, CH/EtOAc, 30 : 1) gave the corresponding *β*‐hydroxyketone (310 mg, 0.42 mmol, 61 %, *dr* = 10:1). The *β*‐hydroxyketone (370 mg, 0.50 mmol), 2,6‐lutidine (0.11 mL, 1.00 mmol) and TBS ⋅ OTf (0.17 mL, 0.75 mmol) in CH_2_Cl_2_ (8 mL). Work‐up NaHCO_3_ (10 mL) and CH_2_Cl_2_ (30 mL). Chromatography (SiO_2_, CH/EtOAc, 30 : 1) gave **27** (383 mg, 0.44 mmol, 90 %). *R*_f_=0.18 (SiO_2_, CH/EtOAc, 10 : 1); [*α*]20D =+56.2° (*c=*0.34, CHCl_3_); ^1^H NMR (700 MHz, CD_2_Cl_2_): δ [ppm]=7.68–7.66 (m, 4H), 7.43–7.41 (m, 2H), 7.38 (ddt, *J*=8.2, 6.7, 1.2 Hz, 4H), 6.44–6.39 (m, 1H), 5.93–5.91 (m, 1H), 5.60–5.54 (m, 1H), 5.11 (dq, *J*=9.7, 1.5 Hz, 1H), 5.08 (dp, *J*=9.0, 1.2 Hz, 1H), 4.35 (ddd, *J*=6.9, 5.8, 1.2 Hz, 0H), 4.31 (ddd, *J*=7.7, 5.9, 1.0 Hz, 1H), 4.14–4.10 (m, 1H), 3.68 (t, *J*=6.2 Hz, 2H), 2.70 (qd, *J*=6.9, 5.7 Hz, 1H), 2.53–2.38 (m, 2H), 2.37–2.31 (m, 1H), 2.00–1.96 (m, 2H), 1.84–1.81 (m, 3H), 1.78–1.76 (m, 3H), 1.58 (d, *J*=1.4 Hz, 2H), 1.57–1.54 (m, 2H), 1.50 (ddd, *J*=8.5, 6.7, 4.7 Hz, 2H), 1.04–1.02 (m, 12H), 0.95 (t, *J*=7.2 Hz, 3H), 0.87 (s, 9H), 0.85 (d, *J*=4.4 Hz, 12H), 0.03 (s, 3H), −0.01 (d, *J*=4.4 Hz, 6H), −0.03—0.04 (m, 3H); ^13^C NMR (176 MHz, CDCl_3_): δ [ppm]=212.6, 135.5, 135.4, 134.2, 132.7, 132.1, 131.9, 130.6, 130.4, 129.7, 129.5, 127.6, 127.2, 76.0, 72.9, 63.8, 52.9, 40.5, 39.3, 36.5, 32.2, 26.6, 25.7, 25.6, 24.5, 24.0, 20.1, 19.1, 18.0, 19.7, 16.4, 15.4, 12.1, 7.2, −4.3, −4.6, −5.1, −5.2; HRMS (ESI+) calcd for C_52_H_88_O_4_Si_3_Na^+^ [*M*+Na]^+^: 881.5726; found: 881.5726.

**Ketone 39**: Method F with TfOH (167 μL, 1.93 mmol), Ipc_2_BH (545 mg, 1.93 mmol) in hexane (0.44 mL). Triflate solution (0.22 mL, 0.76 mmol), DIEA (145 μL, 0.83 mmol), diethylketone **26** (41 μL, 0.39 mmol) and aldehyde **38** (180 mg, 0.28 mmol) in CH_2_Cl_2_ (4 mL).Work‐up buffer pH 7, (2 mL), MeOH (2 mL), H_2_O_2_ (1 mL) and CH_2_Cl_2_ (30 mL). Chromatography (SiO_2_, CH/EtOAc, 30 : 1) gave the corresponding *β*‐hydroxyketone (132 mg, 0.18 mmol, 64 %, *dr* = 10:1). The *β*‐hydroxyketone (145 mg, 0.20 mmol), 2,6‐lutidine (46 μL, 0.40 mmol) and TBS ⋅ OTf (68 μL, 0.30 mmol) in CH_2_Cl_2_ (3 mL). Work‐up NaHCO_3_ (5 mL) and CH_2_Cl_2_ (15 mL). Chromatography (SiO_2_, CH/EtOAc, 20 : 1) gave **39** (153 mg, 0.18 mmol, 90 %). *R*_f_=0.54 (SiO_2_, CH/EtOAc, 10 : 1); [*α*]20D =+23.0° (*c=*0.31, CHCl_3_); ^1^H NMR (700 MHz, CDCl_3_): δ [ppm]=7.73–7.67 (m, 4H), 7.51–7.36 (m, 6H), 6.39 (d, *J*=15.7 Hz, 1H), 5.89 (s, 1H), 5.59 (dd, *J*=15.8, 7.4 Hz, 1H), 5.17 (d, *J*=9.6 Hz, 1H), 4.34 (t, *J*=6.8 Hz, 1H), 3.67 (t, *J*=6.5 Hz, 2H), 3.42 (s, 1H), 2.72 (p, *J*=6.8 Hz, 1H), 2.49 (dq, *J*=10.4, 7.2 Hz, 3H), 1.84 (d, *J*=1.4 Hz, 3H), 1.58 (d, *J*=7.4 Hz, 2H), 1.40–1.23 (m, 8H), 1.10–1.05 (m, 12H), 1.01 (t, *J*=7.2 Hz, 4H), 0.89 (dd, *J*=2.8, 1.3 Hz, 21H), −0.00—0.03 (m, 12H); ^13^C NMR (176 MHz, CDCl_3_): δ [ppm]=213.3, 135.6, 134.2, 131.8, 130.7, 130.3, 129.6, 129.5, 127.6, 75.9, 75.8, 64.0, 53.0, 38.7, 36.6, 33.0, 32.6, 29.7, 26.9, 26.3, 26.0, 25.9, 24.6, 20.2, 19.2, 18.1, 15.3, 12.5, 7.2, −4.0, −4.4, −4.5, −4.9; HRMS (ESI+) calcd for C_51_H_86_O_4_Si_3_Na^+^ [*M*+Na]^+^: 869.5726; found: 869.5727.

**General method G: TDBPS deprotection and TES protection**. To a solution of TBAF (1.00 equiv) in THF at 0 °C was added AcOH (1.00 equiv) resulting in a 41.5 mM stock solution. To the neat alcohol (1.00 equiv) was added the TBAF stock solution at 0 °C (1.10 equiv). The reaction was stirred for 1 h at this temperature then 30 h at room temperature. The reaction was diluted with Et_2_O and quenched with a saturated solution of NaHCO_3_ at 0 °C. After separation of the organic layer, the aqueous layer was extracted with Et_2_O. The organic layers were combined, dried over MgSO_4_ and evaporated *in vacuo*. The crude product was purified by column chromatography.

To a solution of alcohol (1.00 equiv) in CH_2_Cl_2_ at −78 °C was added 2,6‐lutidine (2.00 equiv) followed by TES ⋅ OTf (1.50 equiv). The reaction mixture was stirred 1 h and quenched with water at 0 °C. After separation of the organic layer, the aqueous layer was extracted with CH_2_Cl_2_. The combined organic layers were dried over MgSO_4_ and evaporated *in vacuo*. The crude product was purified by column chromatography.

**Ketone 28**: Method G with TBAF (830 μL,0.84 mmol), AcOH (48 μL, 0.84 mmol) in THF (10.6 mL).Neat alcohol **27** (340 mg, 0.40 mmol) and stock solution (10.6 mL, 0.44 mmol). Work‐up NaHCO_3_ (10 mL) and Et_2_O (10 mL). Chromatography (SiO_2_, CH/EtOAc, 20 : 1) gave the unprotected alcohol (180 mg, 0.29 mmol, 73 %). Unprotected alcohol (102 mg, 0.16 mmol), 2,6‐lutidine (38 μL, 0.33 mmol), TES ⋅ OTf (56 μL, 0.25 mmol) in CH_2_Cl_2_ (4 mL). Work‐up H_2_O (4 mL) and CH_2_Cl_2_ (15 mL). Chromatography (SiO_2_, CH/EtOAc, 20 : 1) gave **28** (108 mg, 0.15 mmol, 90 %). *R*_f_=0.59 (SiO_2_, CH/EtOAc, 10 : 1); [*α*]20D =+61.0° (*c=*0.29, CHCl_3_); ^1^H NMR (700 MHz, CD_2_Cl_2_): δ [ppm]=6.41 (d, *J*=15.8 Hz, 1H), 5.92 (s, 1H), 5.59–5.55 (m, 1H), 5.12–5.07 (m, 2H), 4.32–4.30 (m, 1H), 4.12 (dd, *J*=8.9, 5.9 Hz, 1H), 3.60 (t, *J*=6.2 Hz, 4H), 2.72–2.68 (m, 1H), 2.53–2.39 (m, 2H), 2.33 (ddd, *J*=16.9, 10.1, 5.0 Hz, 1H), 1.98 (t, *J*=7.1 Hz, 2H), 1.83 (d, *J*=1.1 Hz, 3H), 1.77 (s, 3H), 1.58 (s, 3H), 1.50–1.43 (m, 4H), 1.03 (d, *J*=6.9 Hz, 3H), 0.96 (dt, *J*=14.5, 5.2 Hz, 12H), 0.89 (s, 3H), 0.87 (d, *J*=3.0 Hz, 9H), 0.85–0.84 (m, 9H), 0.58 (dt, *J*=8.0, 5.3 Hz, 6H), 0.03 (s, 3H), −0.01 (s, 6H), −0.03 (s, 6H); ^13^C NMR (700 MHz, CD_2_Cl_2_): δ [ppm]=212.8, 135.5, 132.7, 132.1, 131.9, 130.6, 130.4, 129.7, 127.1, 76.0, 72.9, 62.6, 52.9, 40.5, 39.4, 36.4, 32.6, 25.6, 25.6, 24.5, 24.1, 20.1, 18.0, 17.9, 16.4, 15.4, 13.8, 12.1, 7.2, 6.6, 4.4, −4.3, −4.6, −5.2; HRMS (ESI+) calcd for C_42_H_86_O_4_Si_3_N [*M*+NH_4_]^+^: 752.5859; found: 752.5859.

**Ketone 40**: Method G with TBAF (830 μL,0.84 mmol), AcOH (48 μL, 0.84 mmol in THF (10.6 mL). Neat protected alcohol **39** (340 mg, 0.40 mmol) and stock solution (10.6 mL, 0.44 mmol). Work‐up NaHCO_3_ (10 mL) and Et_2_O (10 mL). Chromatography (SiO_2_, CH/EtOAc, 20 : 1) gave the unprotected alcohol (180 mg, 0.29 mmol, 73 %). Unprotected alcohol (127 mg, 0.32 mmol), 2,6‐lutidine (48 μL, 0.42 mmol), TES ⋅ OTf (78 μL, 0.31 mmol) in CH_2_Cl_2_ (4 mL). Work‐up H_2_O (4 mL) and CH_2_Cl_2_ (15 mL). Chromatography (SiO_2_, CH/EtOAc, 20 : 1) gave **40** (140 mg, 0.19 mmol, 90 %). *R*_f_=0.52 (SiO_2_, CH/EtOAc, 10 : 1); [*α*]20D =+26.1° (*c=*0.62, CHCl_3_); ^1^H NMR (700 MHz, CD_2_Cl_2_): δ [ppm]=6.39 (dt, *J*=15.7, 0.9 Hz, 1H), 5.89 (d, *J*=1.7 Hz, 1H), 5.60–5.56 (m, 1H), 5.19–5.16 (m, 1H), 4.33 (ddd, *J*=7.2, 5.9, 1.0 Hz, 1H), 3.58 (td, *J*=6.7, 1.8 Hz, 2H), 3.43 (td, *J*=6.5, 6.0, 3.8 Hz, 1H), 2.69 (qd, *J*=6.9, 5.7 Hz, 1H), 2.54–2.37 (m, 3H), 1.84–1.81 (m, 2H), 1.78–1.74 (m, 3H), 1.51–1.47 (m, 2H), 1.36–1.15 (m, 8H), 1.04 (d, *J*=6.9 Hz, 3H), 0.95 (td, *J*=7.6, 4.5 Hz, 12H), 0.89–0.88 (m, 3H), 0.88 (d, *J*=2.7 Hz, 9H), 0.87 (s, 9H), 0.59 (q, *J*=8.0 Hz, 6H), 0.05 (s, 3H), −0.01 (s, 6H), −0.04 (s, 3H); ^13^C NMR (700 MHz, CD_2_Cl_2_): δ [ppm]=212.6, 132.4, 132.4, 131.7, 130.8, 130.2, 129.4, 75.9, 75.8, 62.8, 53.8, 53.7, 53.6, 53.6, 53.5, 53.4, 53.3, 53.3, 53.1, 52.9, 38.6, 36.4, 33.1, 33.0, 29.7, 26.2, 25.9, 25.7, 25.7, 25.6, 25.6, 25.6, 24.6, 19.9, 18.0, 17.9, 15.1, 12.1, 7.2, 6.5, 4.4, −4.3, −4.7, −4.8, −5.2; HRMS (ESI+) calcd for C_41_H_82_O_4_Si_3_Na^+^ [*M*+Na]^+^: 745.5413; found: 745.5410.

**General method H: Aldol condensation sequence**. LiTMP stock solution: To a solution of TMP (4.00 equiv) in THF at −78 °C was added *n*BuLi (4.00 equiv). The yellow solution was stirred for 15 min at this temperature and 15 min at 0 °C.

The ketone (1.00 equiv) was diluted in THF and cooled down at −78 °C. LiTMP (2.00 equiv) was added dropwise. The mixture was stirred for 30 min at −78 °C and warmed up to −50 °C for 20 min. The enolate solution was cooled down to −78 °C and the aldehyde (1.50 equiv) was added dropwise. After 2 h, the reaction mixture was diluted with CH_2_Cl_2_ and quenched with a saturated solution of NaHCO_3_ at 0 °C. After separation of the organic layer, the aqueous layer was extracted with CH_2_Cl_2_. The organic layers were combined, dried over MgSO_4_, evaporated *in vacuo* and purified by column chromatography.

The mixture of diastereoisomers was directly diluted in THF, DMAP (5.00 equiv) and Ac_2_O (4.00 equiv) were added at 0 °C. After 30 min, buffer pH 7 was added. After separation of the organic layer, the aqueous layer was extracted with Et_2_O. The organic layers were combined, dried over MgSO_4_, evaporated under vacuum and purified by column chromatography.

The protected alcohol was diluted in THF and DBU (35.0 equiv) was added at room temperature. After one night, the reaction was quenched with buffer (pH 7). After separation of the organic layer, the aqueous layer was extracted with EtOAc. The organic layers were combined, dried over MgSO_4_, evaporated under vacuum and purified by column chromatography.

**Ketone 43 a**: Method H with TMP (32 μL, 0.18 mmol), *n*BuLi (0.12 mL, 0.18 mmol) in THF (0.8 mL). Ketone **27** (40 mg, 47 μmol), LiTMP (0.50 mL, 94 μmol) in THF (1.5 mL) and aldehyde **41** (10 mg, 70 μmol) in THF (0.2 mL). Work‐up NaHCO_3_ (2 mL) and CH_2_Cl_2_ (15 mL). Chromatography (SiO_2_, CH/EtOAc, 30 : 1 to 10 : 1) gave the aldol product (39 mg, 39 μmol, 83 %). Directly used with DMAP (24 mg, 0.19 mmol) and Ac_2_O (15 μL, 0.16 mmol) in THF (2 mL). Work‐up buffer (pH 7, 3 mL) and EtOAc (9 mL). Chromatography (SiO_2_, CH/EtOAc, 20 : 1) gave protected alcohol (35 mg, 33 μmol, 86 %). Directly used with DBU (175 μL, 1.29 mmol) in THF (2 mL). Work‐up buffer (pH 7, 2 mL) and EtOAc (9 mL). Chromatography (SiO_2_, CH/EtOAc, 100 : 1) gave **43 a** (31 mg, 32 μmol, 94 %, 67 % over 3 steps). *R*_f_=0.48 (SiO_2_, CH/EtOAc, 10 : 1). [*α*]20D =+24.7° (*c=*0.58, CHCl_3_); ^1^H NMR (700 MHz, CD_2_Cl_2_): δ [ppm]=7.73–7.69 (m, 4H), 7.48–7.39 (m, 7H), 6.62–6.57 (m, 1H), 6.39 (dt, *J*=15.8, 0.8 Hz, 1H), 5.95 (s, 1H), 5.60–5.54 (m, 1H), 5.13 (dddd, *J*=10.3, 9.1, 2.8, 1.4 Hz, 2H), 4.33–4.24 (m, 1H), 4.16 (ddd, *J*=9.0, 5.9, 1.5 Hz, 1H), 4.09 (td, *J*=6.6, 5.0 Hz, 2H), 3.72 (t, *J*=6.0 Hz, 2H), 3.48–3.37 (m, 1H), 2.46–2.33 (m, 1H), 2.34–2.25 (m, 2H), 2.05 (d, *J*=2.0 Hz, 3H), 2.02 (t, *J*=7.2 Hz, 2H), 1.82 (d, *J*=1.3 Hz, 2H), 1.80 (d, *J*=0.6 Hz, 3H), 1.72 (q, *J*=0.9 Hz, 3H), 1.71–1.66 (m, 2H), 1.62 (d, *J*=1.4 Hz, 3H), 1.57 (s, 15H), 1.11 (dd, *J*=6.8, 2.0 Hz, 3H), 1.08 (s, 8H), 0.94–0.89 (m, 11H), 0.89–0.88 (m, 9H), 0.06 (s, 3H), 0.03 (s, 3H), 0.02 (s, 3H), 0.01 (s, 3H). ^13^C NMR (176 MHz, CD_2_Cl_2_): δ [ppm]=203.6, 170.8, 141.3, 137.9, 135.5, 135.4, 134.2, 132.8, 132.2, 132.0, 131.4, 130.1, 129.5, 129.3, 127.6, 127.2, 76.8, 72.9, 64.0, 63.8, 46.4, 40.6, 39.3, 32.2, 28.6, 28.4, 26.6, 25.6, 25.1, 24.6, 24.0, 20.7, 20.1, 19.6, 18.0, 16.4, 15.5, 14.0, 11.3, −4.2, −4.6, −5.1, −5.2. HRMS (ESI+) calcd for C_59_H_96_O_6_Si_3_Na^+^ [*M*+Na]^+^: 1007.6407; found: 1007.6407.

**Ketone 43 b**: Method H with TMP (120 μL, 0.70 mmol), *n*BuLi (0.28 mL, 0.70 mmol) in THF (2.0 mL). Ketone **39** (150 mg, 176 μmol), LiTMP stock solution (1.20 mL, 0.35 mmol) in THF (3.0 mL) and aldehyde **41** (38 mg, 265 μmol) in THF (0.5 mL). Work‐up NaHCO_3_ (4 mL) and CH_2_Cl_2_ (30 mL). Chromatography (SiO_2_, CH/EtOAc, 30 : 1 to 10 : 1) gave the aldol product (148 mg, 149 μmol, 85 %). Directly used with DMAP (91 mg, 0.75 mmol) and Ac_2_O (56 μL, 0.60 mmol) in THF (5 mL). Work‐up buffer (pH 7, 10 mL) and EtOAc (30 mL). Chromatography (SiO_2_, CH/EtOAc, 20 : 1) gave protected alcohol (135 mg, 130 μmol, 87 %). Directly used with DBU (0.68 mL, 4.57 mmol) in THF (8 mL). Work‐up buffer (pH 7, 10 mL) and EtOAc (30 mL). Chromatography (SiO_2_, CH/EtOAc, 100 : 1) gave **43 b** (105 mg, 108 μmol, 83 %, 61 % over 3 steps). *R*_f_=0.48 (SiO_2_, CH/EtOAc, 10 : 1); [*α*]20D =+10.9° (*c=*0.35, CHCl_3_); ^1^H NMR (700 MHz, CD_2_Cl_2_): δ [ppm]=7.66 (dt, *J=*6.8, 1.5 Hz, 4H), 7.42 (ddt, *J*=8.4, 6.5, 1.5 Hz, 2H), 7.40–7.36 (m, 4H), 6.59–6.53 (m, 1H), 6.33 (dt, *J*=15.8, 0.8 Hz, 1H), 5.91–5.86 (m, 1H), 5.59–5.53 (m, 1H), 5.19–5.16 (m, 1H), 4.28–4.25 (m, 1H), 4.05 (q, J=6.5 Hz, 2H), 3.66 (td, *J*=6.5, 2.2 Hz, 2H), 3.44 (td, *J*=6.6, 5.8, 3.5 Hz, 1H), 3.39 (q, *J*=6.9 Hz, 1H), 2.44–2.37 (m, 1H), 2.30–2.23 (m, 2H), 2.04–1.99 (m, 3H), 1.79–1.77 (m, 3H), 1.76 (t, *J*=1.1 Hz, 3H), 1.70 (p, *J*=1.3 Hz, 2H), 1.68–1.64 (m, 2H), 1.59–1.55 (m, 2H), 1.37–1.21 (m, 8H), 1.10–1.06 (m, 2H), 1.04 (s, 9H), 0.91–0.89 (m, 3H), 0.88 (d, *J*=2.7 Hz, 9H), 0.87 (s, 8H), −0.00—0.03 (m, 12H); ^13^C NMR (176 MHz, CD_2_Cl_2_): δ [ppm]=203.6, 170.8, 141.3, 137.8, 135.5, 134.2, 132.5, 132.4, 131.6, 131.5, 129.9, 129.5, 129.0, 127.5, 76.6, 75.9, 64.0, 63.9, 46.4, 38.6, 33.2, 32.6, 29.6, 28.6, 28.4, 26.6, 26.2, 25.9, 25.7, 25.6, 25.1, 24.6, 20.7, 20.0, 19.1, 18.0, 15.2, 14.0, 11.4, −4.3, −4.7, −4.8, −5.1; HRMS (ESI+) calcd for C_58_H_100_O_6_Si_3_N^+^ [*M*+NH_4_]^+^: 990.6853; found: 990.6853.

**Ketone 44 a**: Method H with TMP (94 μL, 0.28 mmol), *n*BuLi (0.11 mL, 0.28 mmol) in THF (2.0 mL). Ketone **28** (104 mg, 140 μmol), LiTMP stock solution (1.1 mL, 0.28 mmol) in THF (3.0 mL) and aldehyde **42** (45 mg, 211 μmol) in THF (0.5 mL). Work‐up NaHCO_3_ (4 mL) and CH_2_Cl_2_ (30 mL). Chromatography (SiO_2_, CH/EtOAc, 100 : 1 to 20 : 1) gave the aldol product (117 mg, 123 μmol, 88 %). Directly used with DMAP (75 mg, 0.62 mmol) and Ac_2_O (47 μL, 0.49 mmol) in THF (4 mL). Work‐up buffer (pH 7, 5 mL) and EtOAc (20 mL). Chromatography (SiO_2_, CH/EtOAc, 30 : 1) gave protected alcohol (111 mg, 112 μmol, 91 %). Directly used with DBU (0.58 mL, 3.92 mmol) in THF (6 mL). Work‐up buffer (pH 7, 10 mL) and EtOAc (30 mL). Chromatography (SiO_2_, CH/EtOAc, 100 : 1) gave **44 a** (84 mg, 90 μmol, 80 %, 64 % over 3 steps). *R*_f_=0.67 (SiO_2_, CH/EtOAc, 10 : 1); [*α*]20D =‐14.7° (*c=*0.32, CHCl_3_); ^1^H NMR (700 MHz, CD_2_Cl_2_): δ [ppm]=7.03–6.98 (m, 1H), 6.53–6.47 (m, 1H), 6.37–6.33 (d, 1H), 6.17–6.11 (m, 1H), 5.90 (dd, *J*=11.8, 6.9 Hz, 1H), 5.56–5.52 (m, 1H), 5.11–5.06 (m, 1H), 4.26–4.22 (m, 1H), 4.14–4.10 (m, 1H), 3.72–3.70 (m, 2H), 3.60 (t, *J*=6.2 Hz, 2H), 3.42 (dd, *J*=13.8, 6.9 Hz, 1H), 2.41 (q, *J*=6.5 Hz, 2H), 2.32 (ddd, *J*=15.6, 9.5, 4.6 Hz, 1H), 2.00–1.96 (m, 2H), 1.78 (s, 3H), 1.78–1.76 (m, 6H), 1.58 (d, *J*=1.1 Hz, 3H), 1.49–1.44 (m, 4H), 1.09 (d, *J*=6.8 Hz, 1H), 0.96–0.94 (m, 9H), 0.90–0.89 (m, 12H), 0.87–0.86 (m, 9H), 0.85 (d, *J*=1.2 Hz, 9H), 0.60–0.57 (m, 6H), 0.06 (d, *J*=2.2 Hz, 6H), 0.02 (d, *J*=1.4 Hz, 3H), −0.01—0.02 (m, 3H), −0.02—0.03 (m, 3H), −0.04 (d, *J*=1.4 Hz, 3H); ^13^C NMR (176 MHz, CD_2_Cl_2_): δ [ppm]=203.6, 147.7, 139.8, 138.0, 135.6, 135.2, 132.6, 132.3, 131.9, 131.4, 130.1, 129.2, 128.4, 127.2, 76.8, 73.0, 62.6, 62.2, 46.3, 40.6, 39.6, 36.9, 32.7, 25.6, 24.6, 24.3, 20.3, 18.2, 17.7, 16.5, 15.5, 14.2, 11.5, 6.5, 4.3, −4.2, −4.6, −5.1, −5.2, −5.6; HRMS (ESI+) calcd for C_53_H_102_O_5_Si_4_Na^+^ [*M*+Na]^+^: 953 : 6697; found: 953.6697.

**Ketone 44 b**: Method H with TMP (134 μL, 0.40 mmol), *n*BuLi (0.30 mL, 0.40 mmol) in THF (2.0 mL). Ketone **40** (145 mg, 200 μmol), LiTMP stock solution (1.3 mL, 0.40 mmol) in THF (3.0 mL) and aldehyde **42** (65 mg, 300 μmol) in THF (0.5 mL). Work‐up NaHCO_3_ (4 mL) and CH_2_Cl_2_ (30 mL). Chromatography (SiO_2_, CH/EtOAc, 100 : 1 to 50 : 1) gave the aldol product (148 mg, 157 μmol, 78 %). Directly used with DMAP (96 mg, 0.78 mmol) and Ac_2_O (59 μL, 0.63 mmol) in THF (5 mL). Work‐up buffer (pH 7, 5 mL) and EtOAc (20 mL). Chromatography (SiO_2_, CH/EtOAc, 50 : 1) gave protected alcohol (130 mg, 133 μmol, 85 %). Directly used with DBU (0.69 mL, 4.64 mmol) in THF (5 mL). Work‐up buffer (pH 7, 10 mL) and EtOAc (30 mL). Chromatography (SiO_2_, CH/EtOAc, 100 : 1) gave **44 b** (108 mg, 117 μmol, 88 %, 58 % over 3 steps). *R*_f_=0.67 (SiO_2_, CH/EtOAc, 10 : 1); [*α*]20D =‐29.6° (*c=*0.23, CHCl_3_); ^1^H NMR (500 MHz, CD_2_Cl_2_): δ [ppm]=7.07–6.99 (m, 1H), 6.54 (dd, *J*=15.3, 10.7 Hz, 1H), 6.36 (d, *J*=15.8 Hz, 1H), 6.17 (dt, *J*=14.3, 6.9 Hz, 1H), 5.91 (s, 1H), 5.63–5.54 (m, 1H), 5.21 (dt, *J*=9.6, 1.6 Hz, 1H), 4.35–4.27 (m, 1H), 3.75 (t, *J*=6.4 Hz, 2H), 3.62 (td, *J*=6.6, 1.3 Hz, 2H), 3.51–3.37 (m, 2H), 2.45 (q, *J*=6.6 Hz, 3H), 1.83 (d, *J*=1.1 Hz, 3H), 1.82–1.76 (m, 6H), 1.51 (dd, *J*=10.5, 4.0 Hz, 2H), 1.33 (dd, *J*=11.4, 7.3 Hz, 8H), 1.15–1.10 (m, 3H), 1.02–0.96 (m, 9H), 0.93 (s, 12H), 0.91 (t, *J*=2.4 Hz, 17H), 0.62 (qd, *J*=7.9, 0.8 Hz, 6H), 0.09 (s, 6H), 0.06 (s, 3H), 0.05 (s, 3H), −0.01 (s, 3H), −0.04 (s, 3H); ^13^C NMR (125 MHz, CD_2_Cl_2_): δ [ppm]=203.6, 139.7, 138.1, 134.9, 132.5, 132.4, 131.6, 129.8, 128.9, 128.3, 76.5, 75.9, 62.8, 62.8, 62.2, 53.8, 53.6, 53.5, 53.4, 53.2, 53.1, 52.9, 46.3, 38.6, 36.8, 33.1, 32.9, 30.0, 29.7, 26.2, 25.9, 25.7, 25.6, 25.6, 25.4, 24.6, 19.9, 18.1, 18.0, 15.2, 14.1, 11.5, 6.5, 4.3, −4.3, −4.7, −4.8, −4.8, −5.1, −5.6, −5.7; HRMS (ESI+) calcd for C_52_H_102_O_5_Si_4_Na^+^ [*M*+Na]^+^: 941.6679; found: 941.6679.

**General method I: Reduction and methylation at the C18 position**. To a solution of ketone (1.00 equiv) in MeOH and THF at 0 °C was added NaBH_4_ (4.00 equiv) and the solution was warmed up to room temperature. After 3 h, the reaction was diluted with EtOAc and quenched carefully with a saturated solution of NH_4_Cl at 0 °C. After separation of the organic layer, the aqueous layer was extracted with EtOAc. The organic layers were combined, dried over MgSO_4_ and evaporated *in vacuo*. The crude product was purified by column chromatography.

To a solution of alcohol (1.00 equiv) in CH_2_Cl_2_ at 0 °C was added proton sponge (5.50 equiv) followed by Me_3_OBF_4_ (5.00 equiv). The reaction was stirred for 3 to 5 h at 0 °C. After this time, a saturated solution of NaHCO_3_ was added at 0 °C. After separation of the organic layer, the aqueous layer was extracted with CH_2_Cl_2_. The organic layers were combined, dried over MgSO_4_, evaporated *in vacuo* and purified by column chromatography.

**Methyl ether 45 a**: Method I with ketone **43 a** (65 mg, 66 μmol) and NaBH_4_ (5 mg, 132 μmol) in MeOH (3 mL) and THF (1 mL). Work‐up with NH_4_Cl (4 mL) and EtOAc (35 mL). Chromatography (SiO_2_, CH/EtOAc, 60 : 1 to 30 : 1) gave the alcohol (44 mg, 45 μmol, 67 %, *dr*=10 : 1). The alcohol (42 mg, 42 μmol) was used with proton sponge (46 mg, 0.23 mmol) and Me_3_OBF_4_ (31 mg, 0.21 mmol) in CH_2_Cl_2_ (3 mL). Work‐up NaHCO_3_ (3 mL) and CH_2_Cl_2_ (20 mL). Chromatography (SiO_2_, CH/EtOAc, 60 : 1) gave **45 a** (37 mg, 37 μmol, 89 %, 60 % over 2 steps). *R*_f_=0.46 (SiO_2_, CH/EtOAc, 10 : 1); [*α*]20D =+29.8° (*c=*0.48, CHCl_3_); ^1^H NMR (700 MHz, CD_2_Cl_2_): δ [ppm]=7.71–7.70 (m, 4H), 7.47–7.44 (m, 2H), 7.43–7.41 (m, 4H), 6.46 (dt, *J*=15.9, 0.9 Hz, 1H), 5.75 (ddd, *J*=15.9, 6.9, 0.7 Hz, 1H), 5.35–5.33 (m, 1H), 5.12 (dddq, *J*=9.7, 4.3, 3.0, 1.4 Hz, 2H), 4.72 (dt, *J*=7.0, 1.5 Hz, 1H), 4.16 (dd, *J*=9.0, 5.8 Hz, 1H), 4.07 (t, *J*=6.7 Hz, 2H), 3.71 (t, *J*=6.2 Hz, 2H), 3.34 (d, *J*=10.0 Hz, 1H), 3.13 (s, 3H), 2.44–2.38 (m, 1H), 2.18–2.09 (m, 2H), 2.04 (s, 4H), 2.03–1.99 (m, 2H), 1.88 (d, *J*=1.4 Hz, 3H), 1.82 (dt, *J*=2.8, 1.4 Hz, 2H), 1.70–1.64 (m, 3H), 1.62–1.57 (m, 7H), 1.50–1.44 (m, 6H), 1.07 (s, 9H), 0.95 (s, 9H), 0.93–0.92 (m, 3H), 0.88 (d, *J*=2.7 Hz, 10H), 0.67 (dd, *J*=6.9, 2.4 Hz, 3H), 0.08 (d, *J*=4.3 Hz, 3H), 0.02 (s, 6H), 0.01 (s, 3H); ^13^C NMR (176 MHz, CD_2_Cl_2_): δ [ppm]=170.9, 135.5, 135.4, 134.2, 134.0, 133.5, 132.6, 132.5, 132.2, 130.2, 129.5, 129.1, 127.7, 127.6, 127.1, 88.3, 72.8, 71.8, 64.3, 63.8, 55.1, 42.4, 40.5, 32.2, 28.3, 27.1, 16.6, 25.9, 25.7, 25.6, 24.5, 24.0, 20.7, 20.2, 19.1, 18.1, 18.0, 16.3, 15.1, 9.8, 8.9, −4.1, −4.6, −5.2, −5.4; HRMS (ESI+) calcd for C_60_H_100_O_6_Si_3_Na [*M*+Na]^+^: 1023.6720; found: 1023.6720.

**Methyl ether 45 b**: Method I with ketone **43 b** (105 mg, 108 μmol) and NaBH_4_ (8 mg, 216 μmol) in MeOH (5 mL) and THF (2 mL). Work‐up with NH_4_Cl (8 mL) and EtOAc (40 mL). Chromatography (SiO_2_, CH/EtOAc, 60 : 1 to 30 : 1) gave the alcohol (73 mg, 75 μmol, 70 %, *dr*=10 : 1). Directly used with proton sponge (88 mg, 0.41 mmol) and Me_3_OBF_4_ (55 mg, 0.37 mmol) in CH_2_Cl_2_ (4 mL). Work‐up NaHCO_3_ (5 mL) and CH_2_Cl_2_ (30 mL). Chromatography (SiO_2_, CH/EtOAc, 60 : 1) gave **45 b** (60 mg, 61 μmol, 82 %, 57 % over 2 steps). *R*_f_=0.44 (SiO_2_, CH/EtOAc, 10 : 1); [*α*]20D =+4.5° (*c=*0.33, CHCl_3_); ^1^H NMR (500 MHz, CD_2_Cl_2_): δ [ppm]=7.68–7.65 (m, 4H), 7.43–7.35 (m, 6H), 6.39 (d, *J*=15.9 Hz, 1H), 5.80 (s, 1H), 5.69 (dd, *J*=15.9, 6.8 Hz, 1H), 5.29 (t, *J*=6.6 Hz, 1H), 5.13–5.10 (m, 1H), 4.68 (d, *J*=6.8 Hz, 1H), 4.07 (t, *J*=6.6 Hz, 2H), 3.65 (t, *J*=6.5 Hz, 2H), 3.41–3.37 (m, 1H), 3.30 (d, *J*=10.0 Hz, 1H), 3.11 (s, 3H), 2.42–2.38 (m, 1H), 2.09 (dt, *J*=13.9, 6.9 Hz, 2H), 2.05 (s, 3H), 1.83 (d, *J*=1.2 Hz, 3H), 1.78 (s, 3H), 1.64 (dt, *J*=14.7, 6.6 Hz, 2H), 1.59–1.55 (m, 2H), 1.47–1.42 (m, 5H), 1.36–1.20 (m, 8H) 1.04 (s, 9H), 0.92–0.90 (s, 9H), 0.89–0.87 (m, 3H), 0.87–0.86 (m, 9H), 0.64–0.61 (d, *J*=6.9 Hz 2H), 0.03 (d, *J*=2.5 Hz, 3H), −0.01‐(‐0.02) (s, 6H), −0.03 (s, 3H); ^13^C NMR (125 MHz, CD_2_Cl_2_): δ [ppm]=171.2, 135.6, 134.3, 134.2, 133.5, 132.8, 132.6, 131.7, 130.1, 129.5, 128.9, 127.6, 127.5, 88.4, 75.8, 71.6, 64.4, 64.0, 55.4, 42.4, 38.6, 32.7, 32.6, 29.7, 28.3, 27.2, 26.9, 26.3, 25.9, 24.9, 21.0, 20.4, 19.3, 18.2, 18.1, 14.8, 10.0, 9.1, −3.8, −4.5, −4.6, −5.1; HRMS (ESI+) calcd for C_59_H_104_O_6_Si_3_N^+^ [*M*+NH_4_]^+^: 1006.7166; found: 1006.7166.

**Methyl ether 46 a**: Method I with ketone **44 a** (84 mg, 90 μmol) and NaBH_4_ (14 mg, 360 μmol) in MeOH (3 mL) and THF (1 mL). Work‐up with NH_4_Cl (4 mL) and EtOAc (35 mL). Chromatography (SiO_2_, CH/EtOAc, 50 : 1) gave the alcohol (67 mg, 73 μmol, 86 %, *dr*=8 : 1). Directly used with proton sponge (86 mg, 0.40 mmol) and Me_3_OBF_4_ (54 mg, 0.36 mmol) in CH_2_Cl_2_ (4 mL). Work‐up NaHCO_3_ (3 mL) and CH_2_Cl_2_ (20 mL). Chromatography (SiO_2_, CH/EtOAc, 80 : 1) gave **46 a** (50 mg, 53 μmol, 72 %, 62 % over 2 steps). *R*_f_=0.69 (SiO_2_, CH/EtOAc, 10 : 1); [*α*]20D =+15.6° (*c=*0.41, CHCl_3_); ^1^H NMR (700 MHz, CDCl_3_): δ [ppm]=6.45–6.41 (m, 1H), 6.38–6.32 (m, 1H), 5.92 (t, *J*=8.1 Hz, 2H), 5.86 (s, 1H), 5.73–5.62 (m, 2H), 5.08 (ddd, *J*=8.3, 5.1, 1.3 Hz, 2H), 4.69 (d, *J*=7.0 Hz, 1H), 4.15–4.10 (m, 2H), 3.66 (t, *J*=6.6 Hz, 2H), 3.60 (t, *J*=6.1 Hz, 2H), 3.34 (d, *J*=9.9 Hz, 1H), 3.10 (s, 3H), 2.39–2.29 (m, 3H), 1.98 (t, *J*=7.2 Hz, 2H), 1.85–1.84 (m, 3H), 1.79 (s, 3H), 1.57 (d, *J*=1.4 Hz, 6H), 1.50–1.43 (m, 4H), 0.95 (dd, *J*=10.3, 5.5 Hz, 9H), 0.92 (s, 9H), 0.89 (s, 12H), 0.86–0.86 (m, 3H), 0.64–0.62 (m, 3H), 0.59 (q, *J*=8.0 Hz, 6H), 0.05 (d, *J*=1.3 Hz, 3H), 0.05 (s, 6H), −0.01 (s, 3H), −0.01 (s, 3H), −0.04 (s, 3H). ^13^C NMR (176 MHz, CDCl_3_): δ [ppm]=135.4, 134.2, 133.9, 132.5, 132.1, 130.7, 129.6, 129.1, 127.7, 126.9, 88.1, 72.8, 71.7, 62.8, 62.5, 55.3, 42.6, 40.4, 39.3, 36.5, 32.5, 25.7, 25.6, 25.6, 24.5, 24.1, 20.1, 18.1, 18.0, 17.9, 16.4, 15.1, 10.3, 8.7, 6.5, 4.3, −4.1, −4.7, −5.3, −5.4, −5.6; HRMS (ESI+) calcd for C_54_H_110_O_5_Si_3_N^+^ [*M*+NH_4_]^+^: 964.7456; found: 964.7456.

**Methyl ether 46 b**: Method I with ketone **44 b** (78 mg, 85 μmol) and NaBH_4_ (13 mg, 340 μmol) in MeOH (4 mL) and THF (1 mL). Work‐up with NH_4_Cl (4 mL) and EtOAc (35 mL). Chromatography (SiO_2_, CH/EtOAc, 50 : 1) gave the alcohol (67 mg, 73 μmol, 86 %, *dr*=10 : 1). Directly used with proton sponge (86 mg, 0.40 mmol) and Me_3_OBF_4_ (54 mg, 0.36 mmol) in CH_2_Cl_2_ (4 mL). Work‐up NaHCO_3_ (3 mL) and CH_2_Cl_2_ (20 mL). Chromatography (SiO_2_, CH/EtOAc, 80 : 1) gave **46 b** (57 mg, 61 μmol, 84 %, 72 % over 2 steps). *R*_f_=0.69 (SiO_2_, CH/EtOAc, 10 : 1); [*α*]20D =‐7.2° (*c=*0.25, CHCl_3_); ^1^H NMR (700 MHz, CDCl_3_): δ [ppm]=6.41–6.37 (m, 1H), 6.33 (ddt, *J*=15.0, 10.6, 1.3 Hz, 1H), 5.92–5.88 (m, 1H), 5.80 (s, 1H), 5.71–5.64 (m, 2H), 5.13–5.10 (m, 1H), 4.69 (dt, *J*=6.9, 1.6 Hz, 1H), 3.67 (t, *J*=6.7 Hz, 2H), 3.59 (t, *J*=6.8 Hz, 3H), 3.40 (td, *J*=8.7, 7.9, 4.7 Hz, 1H), 3.34 (d, *J*=10.0 Hz, 1H), 3.13 (d, *J*=9.8 Hz, 3H), 2.40 (tt, *J*=10.8, 6.5 Hz, 1H), 2.36–2.32 (m, 2H), 1.83 (d, *J*=1.4 Hz, 3H), 1.78 (q, *J*=1.8, 1.1 Hz, 3H), 1.60 (s, 1H), 1.59–1.57 (m, 3H), 1.53–1.49 (m, 3H), 1.34–1.27 (m, 7H), 1.15 (tt, *J*=9.8, 5.8 Hz, 1H), 0.96 (t, *J*=7.9 Hz, 12H), 0.92 (d, *J*=6.4 Hz, 9H), 0.89 (d, *J*=2.9 Hz, 13H), 0.87–0.86 (m, 9H), 0.63 (d, *J*=6.9 Hz, 2H), 0.60 (t, *J*=8.0 Hz, 6H), 0.05 (s, 6H), 0.04 (s, 3H), −0.01 (s, 3H), –0.02 (s, 3H), −0.03 (s, 3H); ^13^C NMR (176 MHz, CDCl_3_): δ [ppm]=134.2, 134.2, 132.8, 132.6, 131.7, 130.6, 129.7, 129.0, 127.9, 127.5, 88.3, 77.2, 77.2, 77.0, 76.8, 75.8, 71.6, 63.0, 62.9, 55.6, 42.7, 38.7, 36.6, 33.0, 32.7, 29.7, 29.7, 26.3, 26.0, 26.0, 26.0, 26.0, 25.9, 25.9, 24.9, 20.4, 18.4, 18.2, 18.1, 14.8, 10.5, 9.0, 6.8, 4.5, −3.8, −4.5, −4.6, −5.1, −5.2, −5.2; HRMS (ESI+) calcd for C_53_H_106_O_5_Si_4_Na^+^ [*M*+Na]^+^: 934.7117; found: 934.7117.

**General method J: Deprotection at the C1 position**.

*J1 = TBDPS group*. To a solution of TBAF (1.00 equiv) in THF at 0 °C was added AcOH (1.00 equiv) resulting in a 41.5 mM solution stock solution. To the neat TBDPS‐protected alcohol (1.00 equiv) was added the stock solution at 0 °C (1.10 equiv). The reaction was stirred for 1 h at this temperature and 44 h at room temperature. The reaction was diluted with Et_2_O and quenched with a saturated solution of NaHCO_3_ at 0 °C. After separation of the organic layer, the aqueous layer was extracted with Et_2_O. The organic layers were combined, dried over MgSO_4_ and evaporated *in vacuo*. The crude product was purified by column chromatography.

**Alcohol 47 a**: Method J1 with TBAF (415 μL, 0.42 mmol) and AcOH (24 μL, 0.42 mmol) in THF (9.6 mL). Alcohol **45 a** (40 mg, 40 μmol) and TBAF stock solution (1.0 mL, 44 μmol). Work‐up NaHCO_3_ (2 mL) and Et_2_O (20 mL). Chromatography (SiO_2_, CH/EtOAc, 10 : 1 to 5 : 1) gave **47 a** (27 mg, 36 μmol, 88 %). *R*_f_=0.16 (SiO_2_, CH/EtOAc, 10 : 1); [*α*]20D =+34.8° (*c=*0.33, CHCl_3_, 20 °C); ^1^H NMR (700 MHz, CD_2_Cl_2_): δ [ppm]=6.46–6.40 (m, 1H), 5.86 (s, 1H), 5.71 (dddd, *J*=16.0, 7.0, 3.6, 0.7 Hz, 1H), 5.31–5.28 (m, 1H), 5.14–5.05 (m, 2H), 4.71–4.65 (m, 1H), 4.14–4.09 (m, 1H), 4.03 (t, *J*=6.7 Hz, 2H), 3.62–3.57 (m, 2H), 3.30 (d, *J*=10.0 Hz, 1H), 3.10 (d, *J*=2.0 Hz, 3H), 2.36 (dqd, *J*=12.6, 6.7, 6.3, 3.6 Hz, 1H), 2.09 (dp, *J*=18.4, 7.3 Hz, 2H), 2.00 (d, *J*=3.3 Hz, 6H), 1.85–1.83 (m, 3H), 1.82–1.77 (m, 3H), 1.64–1.61 (m, 2H), 1.59–1.57 (m, 3H), 1.47–1.40 (m, 8H), 0.91 (s, 9H), 0.90–0.88 (m, 3H), 0.85 (s, 9H), 0.64 (dd, *J*=6.9, 4.4 Hz, 3H), 0.04 (s„ 3H), −0.01 (s, 6H), −0.03—0.05 (s, 3H); ^13^C NMR (176 MHz, CD_2_Cl_2_): δ [ppm]=170.9, 135.2, 134.1, 133.5, 132.6, 132.5, 132.2, 130.2, 129.0, 127.7, 127.2, 88.3, 72.8, 71.8, 64.3, 62.6, 55.1, 42.4, 40.5, 39.3, 32.5, 28.3, 27.1, 25.9, 25.7, 25.6, 24.5, 23.9, 20.7, 20.2, 18.1, 18.0, 16.4, 15.2, 9.8, 8.9, −4.1, −4.7, −5.2, −5.4; HRMS (ESI+) calcd for C_44_H_82_O_6_Si_2_Na^+^ [*M*+Na]^+^: 785.5548; found: 785.5544.

**Alcohol 47 b**: Method J1 with TBAF (415 μL, 0.42 mmol) and AcOH (24 μL, 0.42 mmol) in THF (9.6 mL). Alcohol **45 b** (58 mg, 59 μmol) and TBAF stock solution (1.6 mL, 65 μmol). Work‐up NaHCO_3_ (2 mL) and Et_2_O (20 mL). Chromatography (SiO_2_, CH/EtOAc, 20 : 1 to 10 : 1) gave **47 b** (41 mg, 54 μmol, 92 %). *R*_f_=0.13 (SiO_2_, CH/EtOAc, 10 : 1); [*α*]20D =‐0.7° (*c=*0.22, CHCl_3_); ^1^H NMR (700 MHz, CD_2_Cl_2_): δ [ppm]=6.46–6.40 (m, 1H), 5.86 (s, 1H), 5.71 (dddd, *J*=16.0, 7.0, 3.6, 0.7 Hz, 1H), 5.31–5.28 (m, 1H), 5.14–5.05 (m, 2H), 4.71–4.65 (m, 1H), 4.14–4.09 (m, 1H), 4.03 (t, *J*=6.7 Hz, 2H), 3.62–3.57 (m, 2H), 3.30 (d, *J*=10.0 Hz, 1H), 3.10 (d, *J*=2.0 Hz, 3H), 2.36 (dqd, *J*=12.6, 6.7, 6.3, 3.6 Hz, 1H), 2.09 (dp, *J*=18.4, 7.3 Hz, 2H), 2.00 (d, *J*=3.3 Hz, 6H), 1.85–1.83 (m, 3H), 1.82–1.77 (m, 3H), 1.64–1.61 (m, 2H), 1.59–1.57 (m, 3H), 1.44 (d, *J*=0.8 Hz, 9H), 0.91 (s, 9H), 0.90–0.88 (m, 3H), 0.85 (s, 9H), 0.64 (dd, *J*=6.9, 4.4 Hz, 3H), 0.05 (s, 3H), −0.01 (s, 6H), −0.04 (s, 3H); ^13^C NMR (176 MHz, CDCl_3_): δ [ppm]=170.9, 134.4, 133.5, 132.8, 132.7, 131.6, 130.2, 128.9, 127.4, 88.3, 75.8, 71.7, 64.3, 62.8, 55.1, 42.5, 38.6, 32.9, 32.6, 29.6, 28.3, 27.1, 26.3, 25.9, 25.8, 25.7, 25.6, 25.4, 20.7, 20.0, 18.1, 17.9, 14.6, 9.7, 8.9, −4.1, −4.8, −4.9, −5.3; HRMS (ESI+) calcd for C_43_H_82_O_6_Si_2_Na^+^ [*M*+Na]^+^: 773.5542; found: 773.5542.

*J2=TES group*. To a solution of TES‐protected alcohol (1.00 equiv) in MeOH was added K_2_CO_3_ (30.0 equiv) at 0 °C. The solution was warmed up to room temperature and stirred overnight. The reaction was quenched with a saturated solution of NaHCO_3_ and diluted with EtOAc. After separation of the organic layer, the aqueous layer was extracted with EtOAc. The combined organic layers were dried over MgSO_4_ and evaporated *in vacuo*. The crude product was purified by column chromatography.

**Alcohol 48 a**: Method J2 with alcohol **46 a** (48 mg, 51 μmol) and K_2_CO_3_ (210 mg, 1.53 μmol) in MeOH (7 mL). Work‐up NaHCO_3_ (10 mL) and EtOAc (40 mL). Chromatography (SiO_2_, CH/EtOAc, 10 : 1) gave **48 a** (35 mg, 42 μmol, 82 %). *R*_f_ *=*0.22 (SiO_2_, CH/EtOAc, 10 : 1); [*α*]20D =+10.4° (*c=*0.25, CHCl_3_); ^1^H NMR (500 MHz, CDCl_3_): δ [ppm]=6.43 (d, *J*=15.9 Hz, 1H), 6.35 (dd, *J*=15.1, 10.8 Hz, 1H), 5.91 (d, *J*=11.2 Hz, 1H), 5.86 (s, 1H), 5.73–5.63 (m, 2H), 5.09 (dd, *J*=12.8, 5.3 Hz, 2H), 4.69 (d, *J*=7.0 Hz, 1H), 4.16–4.10 (m, 1H), 3.66 (t, *J*=6.6 Hz, 2H), 3.60 (t, *J*=11.8 Hz, 2H), 3.34 (d, *J*=9.9 Hz, 1H), 3.10 (s, 3H), 2.39–2.35 (m, 1H), 2.32 (dd, *J*=13.4, 6.7 Hz, 2H), 2.02–1.98 (m, 2H), 1.85 (d, *J*=1.2 Hz, 3H), 1.79 (s, 3H), 1.58 (dd, *J*=5.0, 3.8 Hz, 6H), 1.50–1.43 (m, 4H), 0.92 (s, 9H), 0.88 (d, *J*=1.9 Hz, 9H), 0.86 (d, *J*=3.2 Hz, 3H), 0.85 (s, 9H), 0.64–0.62 (m, 3H), 0.05 (d, *J*=1.5 Hz, 3H), 0.05 (s, 6H), −0.01 (s, 3H), −0.01 (s, 3H), −0.04 (s, 3H). ^13^C NMR (126 MHz, CDCl_3_): δ [ppm]=137.1, 136.2, 135.9, 134.5, 134.4, 134.1, 132.6, 131.6, 131.5, 131.0, 129.7, 129.1, 90.1, 74.7, 73.7, 64.7, 64.5, 57.2, 44.6, 42.3, 41.2, 38.4, 34.6, 27.7, 27.6, 27.6, 27.5, 26.4, 25.8, 22.1, 20.1, 18.3, 17.0, 12.3, 10.7, −2.2, −2.8, −3.3, −3.5, −3.7. HRMS (ESI+) calcd for C_48_H_92_O_5_Si_3_Na^+^ [*M*+Na]^+^: 855.6145; found: 855.6145.

**Alcohol 48 b**: Method J2 with alcohol **46 b** (60 mg, 64 μmol) and K_2_CO_3_ (226 mg, 190 μmol) in MeOH (10 mL). Work‐up NaHCO_3_ (10 mL) and EtOAc (40 mL). Chromatography (SiO_2_, CH/EtOAc, 10 : 1) gave **48 b** (50 mg, 61 μmol, 94 %). *R*_f_=0.10 (SiO_2_, CH/EtOAc, 10 : 1); [*α*]20D =‐11.6° (*c=*0.32, CHCl_3_); ^1^H NMR (700 MHz, CD_2_Cl_2_): δ [ppm]=6.40–6.36 (m, 1H), 6.36–6.32 (m, 1H), 5.94–5.90 (m, 1H), 5.81 (s, 1H), 5.74–5.65 (m, 2H), 5.16–5.12 (m, 1H), 4.70 (dt, *J*=6.9, 1.5 Hz, 1H), 3.66 (t, *J*=6.6 Hz, 2H), 3.58 (td, *J*=6.7, 5.3 Hz, 2H), 3.43 (ddd, *J*=9.8, 7.4, 4.3 Hz, 1H), 3.34 (d, *J*=9.9 Hz, 1H), 3.10 (s, 3H), 2.41 (dqd, *J*=10.6, 6.8, 3.7 Hz, 1H), 2.34–2.29 (m, 2H), 1.84 (d, *J*=1.4 Hz, 3H), 1.80–1.77 (m, 3H), 1.60–1.58 (m, 1H), 1.58–1.56 (m, 3H), 1.52–1.49 (m, 2H), 1.34–1.27 (m, 8H), 0.92 (d, *J*=6.5 Hz, 9H), 0.89 (d, *J*=4.0 Hz, 12H), 0.86 (s, 9H), 0.63–0.61 (m, 3H), 0.06 (s, 3H), 0.05 (s, 6H), −0.01 (s, 3H), −0.01 (s, 3H), −0.02 (s, 3H); ^13^C NMR (176 MHz, CD_2_Cl_2_): δ [ppm]=134.3, 134.2, 132.8, 132.7, 131.6, 130.7, 129.6, 128.9, 127.8, 127.5, 88.2, 75.8, 71.7, 62.8, 62.8, 62.8, 55.3, 53.8, 53.7, 53.6, 53.6, 53.5, 53.4, 53.3, 53.1, 42.7, 38.6, 36.5, 32.9, 32.6, 29.7, 29.6, 26.3, 25.8, 25.8, 25.8, 25.7, 25.7, 25.7, 25.7, 25.7, 25.7, 24.5, 20.0, 18.2, 18.1, 18.0, 18.0, 14.5, 10.3, 8.8, −4.1, −4.8, −4.9, −5.3, −5.6, −5.6; HRMS (ESI+) calcd for C_47_H_96_O_5_Si_3_N^+^ [*M*+NH_4_]^+^: 838.6591; found: 838.6591.

**General method K: C1 Oxidations to carboxylic acid, C23 deprotection, macrolactonization and global deprotection**. To a solution of DMSO (10.0 equiv), sulfur trioxide pyridine complex (3.00 equiv) and DIEA (4.00 equiv) in CH_2_Cl_2_ at 0 °C was added alcohol (1.00 equiv) diluted in CH_2_Cl_2_. The solution was stirred at 0 °C for 1.5 h. After this time the reaction was quenched with aqueous saturated solution of NaHCO_3_ and diluted with CH_2_Cl_2_. After separation of the organic layer, the aqueous layer was extracted with CH_2_Cl_2_. The organic layers were combined, dried over MgSO_4_ and evaporated *in vacuo* until 200 mbar. The crude product was then directly used in the next reaction.

The crude aldehyde was diluted in *tert*‐butanol and 2‐methylbut‐2‐ene (10 : 1) and cooled at 0 °C. A solution of NaClO_2_ (3.20 equiv), KH_2_PO_4_ (4.00 equiv) in H_2_O was added to the reaction mixture. The reaction was stirred for 1 h at room temperature. Saturated aqueous solution of NaCl was added and CH_2_Cl_2_. After separation of the organic layer, the aqueous layer was extracted with CH_2_Cl_2_. The organic layers were combined, dried over MgSO_4_ and evaporated under vacuum.

*K1:C23=Ac*. The crude carboxylic acid was diluted in MeOH and K_2_CO_3_ was added (3.00 equiv). The reaction was stirred for 3 h at room temperature. The reaction was quenched with NaHCO_3_ and diluted with CH_2_Cl_2_. After separation of the organic layer, the aqueous layer was extracted with CH_2_Cl_2_. The combined organic layers were washed with brine, dried over MgSO_4_ and evaporated *in vacuo*. The crude product was purified by column chromatography.

*K2:C23=TBS*: To a solution of THF and pyridine at 0 °C was added HF‐pPyr (70 % HF) resulting in a stock solution. To a solution of carboxylic acid (1.00 equiv) in THF at 0 °C was added the HF‐pyr stock solution. The reaction was stirred for 6 h at 0 °C. The reaction was quenched with a saturated solution of NaHCO_3_ and diluted with CH_2_Cl_2_. After separation of the organic layer, the aqueous layer was extracted with CH_2_Cl_2_. The combined organic layers were washed with brine, dried over MgSO_4_ and evaporated *in vacuo*. The crude product was purified by column chromatography.

MNBA (5.00 equiv), DMAP (7.00 equiv) and 4 Å MS were dried for 1 h under high vacuum before CH_2_Cl_2_ was added. The seco acid was diluted in CH_2_Cl_2_ and added to the solution over 20 h at room temperature. Two hours after completion of the addition, the reaction was quenched at 0 °C with buffer (pH 7). After separation of the organic layer, the aqueous layer was extracted with CH_2_Cl_2_. The combined organic layers were washed with brine, dried over MgSO_4_ and evaporated *in vacuo*. The crude product was purified by column chromatography.

The macrolactone (1.00 equiv) was then diluted in THF and cooled down at 0 °C. Pyridine was added followed by HF‐pyr (70 % HF). After 1 day, the reaction was quenched at 0 °C with buffer (pH 7). After separation of the organic layer, the aqueous layer was extracted with EtOAc. The organic layers were washed with a saturated solution of NaHCO_3_, combined, dried over MgSO_4_ and evaporated *in vacuo*. The crude product was purified by column chromatography.

**Analogue 5**: Method K2 with DMSO (30 μL, 420 mmol), SO_3_‐pyr (20 mg, 126 μmol), DIEA (29 μL, 168 μmol) and alcohol **48 a** (35 mg, 42 μmol) in CH_2_Cl_2_ (3 mL). Work‐up NaHCO_3_ (3 mL) and CH_2_Cl_2_ (20 mL). Crude aldehyde diluted in *tert*‐butanol (2 mL) and 2‐methylbut‐2‐ene (0.2 mL) with NaClO_2_ (12 mg, 134 μmol) and KH_2_PO_4_ (23 mg, 168 μmol) in H_2_O (2 mL). Work‐up NaCl (4 mL) and CH_2_Cl_2_ (20 mL). Crude carboxylic acid and HF^.^‐pyr stock solution (0.34 mL, out of a solution of THF (1.3 mL), pyridine (0.75 mL), HF^.^‐pyr (0.25 mL, 75 % HF)) in THF (0.8 mL). Work‐up NaHCO_3_ (10 mL) and CH_2_Cl_2_ (20 mL). Chromatography (SiO_2_, CH/EtOAc, 3 : 2) gave the corresponding seco acid (6.3 mg, 8.6 μmol, 32 % over 3 steps). Directly used with MNBA (15 mg, 43 μmol) and DMAP (7.3 mg, 60 μmol) in CH_2_Cl_2_ (4 mL). Seco acid diluted in CH_2_Cl_2_ (5 mL). Work‐up buffer (pH 7, 7 mL) and CH_2_Cl_2_ (15 mL). Chromatography (SiO_2_, CH/EtOAc, 50 : 1) gave the macrolactone (5.1 mg, 7.1 μmol, 83 %). Directly used with HF^.^‐pyr (0.3 mL) in THF (0.3 mL) and pyridine (0.3 mL). Work‐up buffer (pH 7, 5 mL) and EtOAC (20 mL). Chromatography (SiO_2_, CH/EtOAc, 5 : 1) gave **5** (1.2 mg, 3.4 μmol, 35 %, 6 % over 5 steps).

*R*_f_=0.45 (SiO_2_, CH/EtOAc, 3 : 1); [*α*]20D =‐33.4° (*c=*0.12, CHCl_3_); ^1^H NMR (700 MHz, CD_2_Cl_2_): δ [ppm]=6.53 (d, *J*=16.0 Hz, 1H), 6.32 (dd, *J*=15.1, 10.9 Hz, 1H), 5.93 (d, *J*=10.7 Hz, 1H), 5.67 (dd, *J*=16.0, 4.8 Hz, 1H), 5.63 (s, 1H), 5.60–5.56 (m, 1H), 5.20–5.17 (m, 1H), 5.01 (dd, *J*=9.0, 1.1 Hz, 1H), 4.40 (d, *J*=4.5 Hz, 1H), 4.39–4.37 (m, 1H), 4.01–3.97 (m, 1H), 3.95 (d, *J*=9.4 Hz, 1H), 3.50 (d, *J*=9.0 Hz, 1H), 3.19 (s, 3H), 2.44 (dt, *J*=12.0, 3.9 Hz, 2H), 2.24 (ddd, *J*=9.9, 8.7, 5.4 Hz, 1H), 2.21–2.19 (m, 2H), 1.95 (td, *J*=9.8, 5.8 Hz, 2H), 1.89 (d, *J*=2.3 Hz, 3H), 1.82 (ddd, *J*=9.1, 7.3, 2.0 Hz, 1H), 1.77 (s, 3H), 1.71 (dd, *J*=6.3, 2.9 Hz, 2H), 1.67 (d, *J*=1.2 Hz, 3H), 1.63 (s, 3H), 0.71 (d, *J*=6.7 Hz, 3H), 0.57 (d, *J*=7.2 Hz, 3H); ^13^C NMR (176 MHz, CDCl_3_): δ [ppm]=173.3, 139.2, 134.6, 133.7, 132.6, 132.0, 131.1, 130.8, 128.9, 128.5, 128.0, 127.9, 126.8, 89.3, 72.9, 72.8, 62.6, 55.9, 40.9, 40.3, 39.2, 34.5, 32.7, 24.3, 23.8, 19.8, 17.1, 16.5, 11.8, 10.6; HRMS (ESI+) calcd for C_30_H_46_O_5_Na^+^ [*M*+Na]^+^: 509.3237; found: 509.3237.

**Analogue 6**: Method K1 with DMSO (25 μL, 354 mmol), SO_3_^.^‐pyr (17 mg, 106 μmol), DIEA (25 μL, 141 μmol) and alcohol **47 a** (27 mg, 35 μmol) in CH_2_Cl_2_ (3 mL). Work‐up NaHCO_3_ (3 mL) and CH_2_Cl_2_ (20 mL). Crude aldehyde in *tert*‐butanol (2 mL) and 2‐methylbut‐2‐ene (0.2 mL) with NaClO_2_ (10 mg, 113 μmol) and KH_2_PO_4_ (19 mg, 141 μmol) in H_2_O (2 mL). Work‐up NaCl (4 mL) and CH_2_Cl_2_ (20 mL). Crude carboxylic acid with K_2_CO_3_ (15 mg, 106 μmol) in MeOH (2.5 mL). Work‐up NaHCO_3_ (5 mL) and CH_2_Cl_2_ (20 mL). Chromatography (SiO_2_, CH/EtOAc, 3 : 2) gave the corresponding seco acid (5 mg, 7 μmol, 20 % over 3 steps). Directly used with MNBA (11 mg, 31 μmol) and DMAP (5.2 mg, 43 μmol) in CH_2_Cl_2_ (3 mL). Seco acid diluted in CH_2_Cl_2_ (4 mL). Work‐up buffer (pH 7, 3 mL) and CH_2_Cl_2_ (15 mL). Chromatography (SiO_2_, CH/EtOAc, 50 : 1) gave the macrolactone (4.3 mg, 6 μmol, 86 %). Directly used with HF‐^.^pyr (0.2 mL) in THF (0.3 mL) and pyridine (0.3 mL). Work‐up buffer (pH 7, 5 mL) and EtOAC (20 mL). Chromatography (SiO_2_, CH/EtOAc, 10 : 1 to 5 : 1) gave **6** (1.2 mg, 2.5 μmol, 41 %, 7 % over 5 steps). *R*_f_=0.37 (SiO_2_, CH/EtOAc, 2 : 1); [*α*]20D =‐10.4° (*c=*0.1, CHCl_3_, 20 °C); ^1^H NMR (700 MHz, CD_2_Cl_2_): δ [ppm]=6.51 (d, *J*=15.9 Hz, 1H), 5.71–5.67 (m, 1H), 5.66 (s, 1H), 5.38–5.35 (m, 1H), 5.18 (d, *J*=1.2 Hz, 1H), 4.99 (dd, *J*=9.1, 1.2 Hz, 1H), 4.32 (s, 1H), 4.09–4.05 (m, 1H), 3.99–3.93 (m, 2H), 3.43 (d, *J*=9.9 Hz, 1H), 3.17 (s, 3H), 2.27–2.18 (m, 5H), 2.07–2.03 (m, 2H), 2.01–1.97 (m, 2H), 1.91 (d, *J*=1.4 Hz, 3H), 1.77 (dd, *J*=1.4, 0.8 Hz, 3H), 1.72–1.68 (m, 2H), 1.65 (d, *J*=1.4 Hz, 3H), 1.59–1.55 (m, 2H), 1.51 (t, *J*=1.2 Hz, 3H), 1.45 (ddd, *J*=10.5, 4.4, 2.9 Hz, 2H), 0.73 (d, *J*=6.7 Hz, 3H), 0.60 (d, *J*=7.1 Hz, 3H); ^13^C NMR (176 MHz, CD_2_Cl_2_): δ [ppm]=173.3, 138.4, 135.0, 132.9, 132.2, 131.9, 130.9, 130.6, 128.8, 18.7, 126.7, 90.1, 73.0, 71.7, 64.1, 55.4, 40.7, 40.4, 38.8, 34.1, 27.8, 26.6, 25.9, 24.3, 23.0, 19.7, 17.0, 16.7, 12.1, 9.8; HRMS (ESI+) calcd for C_30_H_48_O_5_Na^+^ [*M*+Na]^+^: 511.3394; found: 511.3394.

**Analogue 7**: Method K2 with DMSO (41 μL, 523 mmol), SO_3_^.^‐pyr (25 mg, 157 μmol), DIEA (37 μL, 209 μmol) and alcohol **48 b** (43 mg, 52 μmol) in CH_2_Cl_2_ (3 mL). Work‐up NaHCO_3_ (3 mL) and CH_2_Cl_2_ (20 mL). Crude aldehyde in *tert*‐butanol (2 mL) and 2‐methylbut‐2‐ene (0.2 mL) with NaClO_2_ (15 mg, 167 μmol) and KH_2_PO_4_ (29 mg, 209 μmol) in H_2_O (2 mL). Work‐up NaCl (4 mL) and CH_2_Cl_2_ (20 mL). Crude carboxylic acid and HF^.^‐pyr stock solution (0.50 mL, out of a solution of THF (1.3 mL), pyridine (0.75 mL), HF^.^‐pyr (0.25 mL, 75 % HF)) in THF (1.0 mL). Work‐up NaHCO_3_ (10 mL) and CH_2_Cl_2_ (20 mL). Chromatography (SiO_2_, CH/EtOAc, 3 : 2) gave the corresponding seco acid (12 mg, 17 μmol, 42 % over 3 steps). Directly used with MNBA (29 mg, 84 μmol) and DMAP (14 mg, 117 μmol) in CH_2_Cl_2_ (6 mL). Seco acid diluted in CH_2_Cl_2_ (8 mL). Work‐up buffer (pH 7, 10 mL) and CH_2_Cl_2_ (25 mL). Chromatography (SiO_2_, CH/EtOAc, 50 : 1) gave the macrolactone (9.8 mg, 14 μmol, 83 %). Directly used with HF^.^‐pyr (0.5 mL) in THF (0.5 mL) and pyridine (0.5 mL). Work‐up buffer (pH 7, 5 mL) and EtOAC (20 mL). Chromatography (SiO_2_, CH/EtOAc, 10 : 1 to 5 : 1) gave **7** (1.6 mg, 3.4 μmol, 24 %, 8 % over 5 steps). *R*_f_=0.28 (SiO_2_, CH/EtOAc, 2 : 1); [*α*]20D =‐24.7° (*c=*0.15, CHCl_3_); ^1^H NMR (700 MHz, CD_2_Cl_2_): δ [ppm]=6.53 (dd, *J*=16.0, 4.0 Hz, 1H), 6.35 (dd, *J*=15.3, 10.7 Hz, 1H), 5.93 (d, *J*=10.8 Hz, 1H), 5.69 (dd, *J*=16.0, 4.9 Hz, 1H), 5.64 (s, 1H), 5.60 (ddd, *J*=13.8, 9.6, 5.3 Hz, 1H), 5.16 (d, *J*=9.9 Hz, 1H), 4.39 (s, 1H), 4.28 (ddd, *J*=10.8, 8.7, 4.4 Hz, 1H), 4.04 (ddd, *J*=10.1, 6.8, 4.6 Hz, 1H), 3.53 (d, *J*=9.2 Hz, 1H), 3.24 (td, *J*=8.9, 2.4 Hz, 1H), 3.18 (s, 3H), 2.48–2.43 (m, 2H), 2.26–2.15 (m, 3H), 1.89 (d, *J*=2.7 Hz, 3H), 1.87–1.84 (m, 1H), 1.76 (d, *J*=3.1 Hz, 3H), 1.64 (d, *J*=2.9 Hz, 3H), 1.51–1.42 (m, 4H), 1.25–1.13 (m, 4H), 0.80 (d, *J*=6.7 Hz, 3H), 0.55 (d, *J*=7.2 Hz, 3H); ^13^C NMR (176 MHz, CD_2_Cl_2_): δ [ppm]=δ 173.6, 134.4, 133.8, 132.3, 132.0, 131.4, 130.9, 129.2, 128.5, 128.1, 128.0, 89.3, 76.3,73.2, 62.9, 55.9, 40.9, 40.2, 35.2, 33.8, 32.3, 29.9, 26.1, 25.5, 24.5, 19.8, 17.3,11.4, 10.5; HRMS (ESI+) calcd for C_29_H_46_O_5_Na^+^ [*M*+Na]^+^: 497.3237; found: 497.3237.

**Analogue 8**: Method K1 with DMSO (20 μL, 208 mmol), SO_3_^.^‐pyr (13 mg, 84 μmol), DIEA (20 μL, 112 μmol) and alcohol **47 b** (21 mg, 28 μmol) in CH_2_Cl_2_ (3 mL). Work‐up NaHCO_3_ (3 mL) and CH_2_Cl_2_ (20 mL). Crude aldehyde in *tert*‐butanol (2 mL) and 2‐methylbut‐2‐ene (0.2 mL) with NaClO_2_ (8 mg, 89 μmol) and KH_2_PO_4_ (15 mg, 111 μmol) in H_2_O (2 mL). Work‐up NaCl (4 mL) and CH_2_Cl_2_ (20 mL). Crude carboxylic acid with K_2_CO_3_ (11 mg, 84 μmol) in MeOH (2.0 mL). Work‐up NaHCO_3_ (2 mL) and CH_2_Cl_2_ (15 mL). Chromatography (SiO_2_, CH/EtOAc, 3 : 2) gave the corresponding seco acid (9.5 mg, 13 μmol, 46 % over 3 steps). Directly used with MNBA (23 mg, 66 μmol) and DMAP (11 mg, 92 μmol) in CH_2_Cl_2_ (5 mL). Seco acid diluted in CH_2_Cl_2_ (7 mL). Work‐up buffer (pH 7, 3 mL) and CH_2_Cl_2_ (15 mL). Chromatography (SiO_2_, CH/EtOAc, 50 : 1) gave the macrolactone (7.1 mg, 10 μmol, 77 %). Directly used with HF^.^‐pyr (0.3 mL) in THF (0.5 mL) and pyridine (0.5 mL). Work‐up buffer (pH 7, 5 mL) and EtOAC (20 mL). Chromatography (SiO_2_, CH/EtOAc, 10 : 1 to 5 : 1) gave **8** (2.1 mg, 4.4 μmol, 31 %, 11 % over 5 steps). *R*_f_=0.44 (SiO_2_, CH/EtOAc, 2 : 1); [*α*]20D =‐8.0° (*c=*0.20, CHCl_3_); ^1^H NMR (700 MHz, CD_2_Cl_2_): δ [ppm]=6.59 (d, *J*=16.0 Hz, 1H)., 5.71 (ddd, *J*=15.9, 4.6, 0.7 Hz, 1H), 5.64 (s, 1H), 5.37 (ddd, *J*=9.3, 5.4, 1.6 Hz, 1H), 5.18 (dq, *J*=9.9, 1.3 Hz, 1H), 4.46 (s, 1H), 4.11 (dt, *J*=10.8, 6.0 Hz, 1H), 3.99–3.95 (m, 1H), 3.42 (d, *J*=10.0 Hz, 1H), 3.24 (td, *J*=8.8, 8.3, 2.2 Hz, 1H), 3.16 (s, 3H), 2.30 (dt, *J*=14.6, 6.8 Hz, 1H), 2.24–2.18 (m, 3H), 2.05 (dtdd, *J*=14.1, 6.4, 5.1, 1.3 Hz, 1H), 1.89 (d, *J*=1.4 Hz, 3H), 1.87–1.83 (m, 1H), 1.76 (dd, *J*=1.5, 0.8 Hz, 3H), 1.62–1.56 (m, 4H), 1.51 (t, *J*=1.2 Hz, 3H), 1.27–1.18 (m, 8H), 0.81 (d, *J*=6.7 Hz, 3H), 0.57 (d, *J*=7.1 Hz, 3H); ^13^C NMR (176 MHz, CD_2_Cl_2_): δ [ppm]=173.3, 134.3, 132.9, 132.6, 132.0, 131.4, 130.6, 128.4, 127.9, 89.6, 76.7, 72.8, 64.0, 55.4, 40.5, 39.9, 35.1, 34.0, 29.6, 28.1, 26.9, 26.8, 26.0, 25.5, 24.4, 19.7, 17.5, 11.1, 9.7; HRMS (ESI+) calcd for C_29_H_48_O_5_Na^+^ [*M*+Na]^+^: 499.3394; found: 499.3394.

**MTT assays**: The test compounds were investigated at human 1321 N1 astrocytoma cells using the 3‐(4,5‐dimethylthiazol‐2‐yl)‐2,5‐diphenyltetrazolium bromide (MTT) assay in order to assess their cytotoxic effects. Assays were performed as previously described by Baqi *et al*.^30^ In brief, cells were detached from the 175 cm^2^ culture flasks in which they were grown and subsequently counted using a Neubauer haemocytometer. Then, they were resuspended in the growth medium. An aliquot of the cell suspension (180 μL) was added into each well of a 96‐well plate to obtain 1000 cells per well and incubated for 24 h at 37 °C, 5 % CO_2_, and 95 % humidity. The outer wells of the 96‐well plate were filled with 200 μL of phosphate‐buffered saline (PBS) to prevent evaporation of the fluid. After 24 h, stock solutions (10 mM) of the test compounds (archazolids) were prepared in DMSO and diluted with cell culture medium to give tenfold of the final concentrations. Then, test compound solution (20 μL) was added to each well. The final DMSO concentration was 1 %. The cells were incubated in the presence of the appropriate drug for 71 h. Then, 40 μL from a freshly made stock solution of MTT in water (5 mg/mL) was added to each well, and the cells were incubated for 1 h at 37 °C, 5 % CO_2_. After the incubation time, the medium containing MTT was removed, and 100 μL of DMSO was added to each well in order to dissolve the crystals that were formed. The spectrophotometric absorbance was subsequently measured at 570 nm using a FlexStation (3 multimode plate reader, molecular devices) with a filter of 690 nm. The data were analyzed using Microsoft Excel and GraphPad Prism 5. Results were evaluated by comparing the absorbance of the wells containing compound‐treated cells with the absorbance of wells containing 1 % DMSO without any drug (=100 % viability). All experiments were performed in duplicates in at least three separate experiments.

**P2X3 receptor assay**. 1321 N1 astrocytoma cell lines stably expressing the human P2X3 receptor were utilized to determine the compounds’ inhibition of ATP‐induced calcium influx as previously described.^13^, ^31^‐^32^ The agonist concentration used corresponded to ∼80 % of its maximal effect. Full concentration−inhibition curves were determined, and IC_50_ values were calculated using GraphPad Prism. Data are means from at least 3 separate experiments, each performed in duplicates.

**A_3_ adenosine receptor radioligand binding assay**. Membrane preparations of Chinese hamster ovary (CHO) cells expressing human A_3_ARs were obtained as described before.^33^ [^3^H]Phenyl‐8‐ethyl‐4‐methyl‐(8*R*)‐4,5,7,8‐tetrahydro‐1*H*‐imidazo‐[2,1‐*i*]purine‐5‐one ([^3^H]PSB‐11, 53 Ci/mmol) was used as a radioligand (0.5 nM). Nonspecific binding was determined in the presence of 100 μM (*R*)‐*N* ^*6*^‐phenylisopropyladenosine (*R*‐PIA). The competition assays were performed in a total volume of 400 μL in assay buffer (50 mM Tris ⋅ HCl, pH 7.4). Stock solutions of the test compounds were prepared in DMSO; the final DMSO concentration was 1 %. The membrane preparations were preincubated for 20 min with adenosine deaminase 2 U/mL per mg of protein. Incubation was carried out for 60 min at 23 °C. The incubation was terminated by filtration through GF/B glass‐fiber filters using a 48‐channel cell harvester, and filters were washed three times with ice‐cold Tris ⋅ HCl buffer (50 mM, pH 7.4). The filters were transferred into scintillation vials and incubated for 6 h with 2.5 mL of scintillation cocktail (Beckman‐Coulter). Radioactivity was counted in a liquid scintillation counter. At least three separate experiments were performed. Data were analyzed using Graph Pad Prism version 5 (San Diego, CA, USA). For the calculation of *K*_i_ values by nonlinear regression analysis, the Cheng−Prusoff equation and a *K*_D_ value of 4.9 nM for [^3^H]PSB‐11 were used.

**HLE assay**. Assay buffer was 50 mM sodium phosphate buffer (pH 7.8) containing 500 mM NaCl. An enzyme stock of 100 μg/mL was prepared in 100 mM sodium acetate buffer (pH 5.5). A 50 mM stock solution of the chromogenic substrate MeO‐Suc‐Ala‐Ala‐Pro‐Val‐pNA was prepared in DMSO and diluted with assay buffer containing 10 % DMSO to a final concentration of 2 mM. In each cuvette, 890 μL of assay buffer were pipetted followed by 10 μL of DMSO (or inhibitor solution in DMSO) and 50 μL of the substrate dilution. The reaction was started by addition of 50 μL of enzyme solution. The final concentrations were as follows, substrate, 100 μM (=1.85 × *K*_m_); DMSO, 1.5 %; HLE, 100 ng/mL. The progress curves of product formation were followed at 405 nm and 25 °C for 10 min and analyzed by linear regression. IC_50_ values were determined from duplicate measurements by nonlinear regression using the equation *v*_s_=*v*_0_/(1+[I]/IC_50_), where *v*_s_ is the steady‐state rate, *v*_0_ is the rate in the absence of an inhibitor, and [I] is the inhibitor concentration. Standard errors of the mean refer to the nonlinear regression analysis.^34^, ^35^

Full experimental procedures and copies of NMR spectra are available in the Supporting Information.

## Conflict of interest

The authors declare no conflict of interest.

## Supporting information

As a service to our authors and readers, this journal provides supporting information supplied by the authors. Such materials are peer reviewed and may be re‐organized for online delivery, but are not copy‐edited or typeset. Technical support issues arising from supporting information (other than missing files) should be addressed to the authors.

Supplementary
