# Supplementary material for: Design, Synthesis and Biological Evaluation of Highly Potent Simplified Archazolids
Source: ChemMedChem. 2020 Jun 10;15(14):1348–63. doi: 10.1002/cmdc.202000154 (PMC7496434; doi:10.1002/cmdc.202000154)

ChemMedChem

Supporting Information

Design, Synthesis and Biological Evaluation of Highly Potent Simplified Archazolids

Solenne Rivière, Christin Vielmuth, Christiane Ennenbach, Aliaa Abdelrahman, Carina Lemke, Michael Gütschow, Christa E. Müller, and Dirk Menche*© 2020 The Authors. Published by Wiley-VCH Verlag GmbH & Co. KGaA. This is an open access article under the terms of the Creative Commons Attribution License, which permits use, distribution and reproduction in any medium, provided the original work is properly cited.

Contents

1. General methods 2

2. Chemical syntheses and characterization data 3

    2.1 Synthesis of main fragment 27 and 28 3

    2.2 Synthesis of main fragments 39 and 40 15

    2.3 Coupling by an aldol-condensation sequence 24

    2.4 Completion of analogue 5 30

    2.5 Completion of analogue 6 33

    2.6 Completion of analogue 7 37

    2.7 Completion of analogue 8 40

    2.8 Protecting group strategy 44

3. Biological assays 54

    3.1 MTT assay 54

    3.2 Human P2X3 Inhibition assay 54

    3.3 A_3 Adenosine Receptor Radioligand Binding Assay 55

    3.4 Protease Assay 55

4. Copies of NMR spectra 56

1. General methods

Reaction conditions: All reagents were purchased from commercial suppliers (*Sigma-Aldrich*, *TCI*, *AlfaAesar*, *Acros*, ABCR, *Carbolution*) in the highest grade available and used without further purification, unless stated otherwise. Anhydrous solvents (DCM, THF, diethyl ether, MeCN, DMF, toluene) were obtained from a MB-SPS 800 solvent purification system from *MBraun* and stored over molecular sieves (4 Å). Other solvents (*n*-hexane, MeOH, EtOH, $CHCl_3$) were purchased from commercial suppliers (*Sigma-Aldrich*, *AlfaAesar*, *Acros*) in HPLC or analytical reagent grade and used without further purification. Cyclohexane and DCM were purified from high-boiling impurities by distillation over a Vigreux column. Ethyl acetate was distilled over a packed column (1 m).

All reactions with dry solvents were performed under an atmosphere of argon in flame-dried glassware which had been cooled under argon unless stated otherwise. The flasks were equipped with rubber septa and reactants were handled using standard Schlenk techniques. Temperatures above room temperature (20-23 °C) refer to oil bath temperatures which were controlled by a temperature modulator. For cooling, the following baths were used: acetone/dry ice (–78 °C), water/ice (0 °C), MeCN/dry ice (-40°C) for other temperatures below 0 °C a Huber TC100E-F-NR cooler was used.

Reactions were monitored by TLC on silica gel 60 F254 pre-coated polyester plates (0.2 mm SiO_2, *Macherey-Nagel*) and visualized using UV light (λ = 254 nm and 366 nm) and/or staining with a solution of CAM (1.0 g $Ce(SO_4)_2$, 2.5 g $(NH_4)_6Mo_7O_{24}$, 8 mL conc. H_2SO_4 in 100 mL H_2O) and subsequent heating.

Purification methods: For column chromatography, silica gel (pore size 60 Å, 40-63 µm) obtained from Merck or Aldrich was used. Compounds were eluated using the stated mixtures under a positive pressure of nitrogen or air. Solvents for column chromatography were distilled prior to use.

Analytical methods All NMR spectra were recorded at room temperature unless stated otherwise on Bruker spectrometers with operating frequencies of 125 (^{13}C), 150 (^{13}C), 176 (^{13}C), 400 (^{1}H), 500 ($_{1}H$), 600 (^{1}H) and 700 MHz (^{1}H) in deuterated solvents obtained from *Deutero* and *CarlRoth*. Chemical shifts (λ) are reported in ppm relative to tetramethylsilane (λ = 0.00 ppm) and the spectra were calibrated to the residual signal of undeuterated solvents.[1] Data for 1H-NMR spectra are reported as follows: chemical shift (multiplicity, coupling constants, number of hydrogens). Abbreviations used are: s (singlet), d (doublet), t (triplet), q (quartet), quint (quintet), m (multiplet), br (broad).

Mass spectra (MS) and high-resolution-mass spectra (HR-MS) were recorded on Thermo LTQ Orbitrab Velos mass spectrometer.

Optical rotations were measured with a Perkin Elmer 341 in a 10 mm cuvette and are uncorrected.

[1] G. R. Fulmer, A. J. Miller, N. H. Sherden, H. E. Gottlieb, A. Nudelman, B. M. Stoltz, J. E. Bercaw, K. I. Goldberg, *Organometallics* **2010**, 29, 2176–2179.

2. Chemical syntheses and characterization data

2.1 Synthesis of main fragment 27 and 28

Synthesis of ketone 12

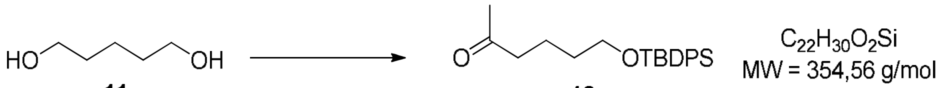

To a solution of 1,5-pentanediol (**11**) (2.00 g, 19.3 mmol, 1.10 eq) in THF (100 mL) at 0 °C was added NaH (60 % in oil, 419 mg, 17.5 mmol, 1.00 eq). The reaction was stirred 30 min at 0 °C and for 1 h at room temperature. TBDPSCl (4.5 mL, 17.5 mmol, 1.00 eq) was added dropwise and the reaction mixture was stirred overnight. The mixture was diluted with Et_2O (100 mL) and quenched with a saturated solution of NH_4Cl (50 mL) and water (50 mL). After separation of the organic layer, the aqueous layer was extracted with Et_2O (3*100 mL). The combined organic layers were dried over $MgSO_4$ and evaporated *in vacuo*. The crude mixture was purified by column chromatography (SiO_2, CH/EtOAc, 4:1) to obtain the desired mono-protected alcohol (2.43 g, 7.10 mmol, 42 %) as a thick colorless oil.

DMSO (2.94 mL, 43.8 mmol, 3.00 eq) was added dropwise to a solution of $(COCl)_2$ (2.52 mL, 29 mmol, 2.00 eq) in DCM (80 mL) at -78 °C. The mixture was stirred 15 min then the mono-protected alcohol (5.0 g, 14.6 mmol, 1.00 eq) in DCM (4 mL) was added over 15 min. After 1 h, NEt_3 (9.8 mL, 73 mmol, 4.00 eq) was added over 10 min. The reaction was stirred for 1 h at -78 °C then warmed up at 0 °C for 30 min. The reaction was quenched with a slow addition of water (100 mL). After separation of the organic layer, the aqueous layer was extracted with DCM (3*100 mL). The combined organic layers were dried over $MgSO_4$ and evaporated *in vacuo.* The crude product was directly used in the next reaction.

To a solution of crude aldehyde (4.70 g, 13.8 mmol, 1.00 eq) in THF (100 mL) at -78 °C was added MeMgBr (3 M in Et_2O, 9.20 mL, 27.6 mmol, 2.00 eq). The reaction was stirred 30 min, diluted with Et_2O (80 mL) and quenched with a cold saturated solution of NH_4Cl (100 mL). After separation of the organic layer, the aqueous layer was extracted with Et_2O (3*100 mL). The organic layers were combined, dried over $MgSO_4$ and evaporated *in vacuo*. The crude product was purified by column chromatography (SiO_2, CH/EtOAc, 9:1) to give the racemic alcohol (3.83 g, 10.6 mmol, 73 % over 2 steps) as a colorless oil.

DMSO (1.87 mL, 25.6 mmol, 2.40 eq) was added dropwise to a solution of $(COCl)_2$ (1.12 mL, 12.8 mmol, 1.20 eq) in DCM (60 mL) at -78 °C. The mixture was stirred 15 min then alcohol (3.83 g, 10.6 mmol, 1.00 eq) in DCM (4 mL) was added over 15 min. After 1 h, NEt_3 (5.81 mL, 42.6 mmol, 4.00 eq) was added over 10 min. The reaction was stirred for 1 h at -78 °C and warmed up to 0 °C for 30 min. The reaction was quenched with a slow addition of water (100 mL). After separation of the organic layer, the aqueous layer was extracted with DCM (3*50 mL). The combined organic layers were dried over $MgSO_4$ and evaporated *in vacuo.* The crude ketone **12** (3.50 g, 9.87 mmol, 93 %) was use without further purification.

***R*$_f$** = 0.75 (SiO_2, CH/EtOAc, 1:1); **^{1}H-NMR** (500 MHz, $CDCl_3$): δ [ppm] = 7.72 – 7.61 (m, 4H), 7.48 – 7.31 (m, 6H), 3.67 (td, *J* = 6.1, 4.7 Hz, 2H), 2.41 (t, *J* = 7.2 Hz, 2H), 2.11 (s, 3H), 1.72 – 1.51 (m, 4H), 1.05 (s, 9H); ^{13}C-NMR (126 MHz, CDCl3): δ [ppm] = 205.1, 135.6, 134.0, 129.6, 127.6, 63.5, 43.4, 31.9, 29.8, 26.9, 20.3, 19.2; **^{13}C NMR** (126 MHz, $CDCl_3$) δ [ppm] = 209.1, 135.6, 134.0, 129.6, 127.6, 63.5, 43.4, 31.9, 29.8, 26.9, 20.3, 19.2, **HRMS (APCI)** calculated for $C_{22}H_{31}O_2Si^+$ $[M+H]^+$: 355.2088, found : 355.2088. The spectroscopic data were in agreement with those previously reported.[2]

Synthesis of ester 14

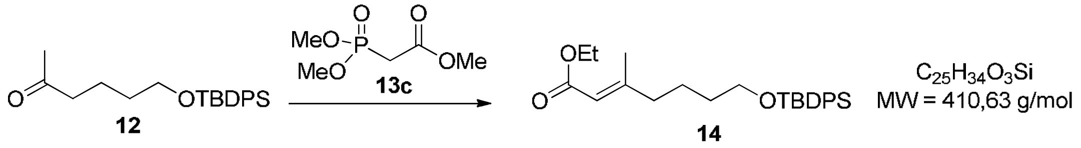

To a solution of trimethyl phosphonoacetate (**13c**) (7.48 mL, 46.2 mmol, 3.00 eq) and DMPU (2.78 mL, 23.1 mmol, 1.50 eq) in THF (100 mL) at 0 °C was added *n*-BuLi (2.5 M in hexane, 17.2 mL, 43.1 mmol, 2.80 eq). The mixture was stirred for 30 min then ketone **12** (5.46 g, 15.4 mmol, 1.00 eq) in THF (4 mL) was added dropwise. After stirring overnight at room temperature, the reaction was quenched with water (100 mL) at 0 °C. After separation of the organic layer, the aqueous layer was extracted with DCM (3*100 mL). The organic layers were combined, dried over $MgSO_4$ and evaporated *in vacuo*. A crude product ^{1}H-NMR showed a 3:1 E/Z mixture. Purification by column chromatography (SiO_2, CH/Et_2O, 50:1 to 45:1) yielded to the desired isomer **14-*E*** (3.73 g, 9.06 mmol, 59 %) as a colorless oil and the *Z* isomer (1.31 g, 3.19 mmol, 21 %).

***R*$_f$** = 0.48 (SiO_2, CH/EtOAc, 10:1); **^{1}H-NMR** (500 MHz, $CDCl_3$): δ [ppm] = 7.69 (dq, *J* = 6.6, 1.7 Hz, 4H), 7.48 – 7.38 (m, 6H), 5.68 (q, *J* = 1.6 Hz, 1H), 3.72 (s, 3H), 3.68 (dt, *J* = 3.9, 1.8 Hz, 2H), 2.16 – 2.12 (m, 5H), 1.63 – 1.57 (m, 4H), 1.08 (s, 9H); **^{13}C-NMR** (125.7 MHz, $CDCl_3$): δ [ppm] = 167.3, 160.4, 135.6, 134.0, 129.6, 127.6, 115.2, 63.5, 50.8, 40.6, 32.0, 26.9, 23.6, 19.2, 18.7; **HRMS (ESI+)** calculated for $C_{25}H_{34}O_2SiNa^+$ $[M+Na]^+$: 433.2149, found : 433.2155.

Synthesis of aldehyde 15

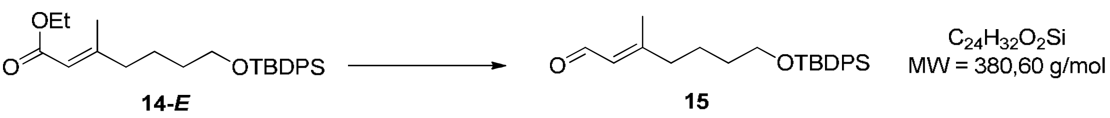

To a solution of ester **14-*E*** (3.73 g, 9.08 mmol, 1.00 eq) in DCM (150 mL) at -78 °C was added DIBALH (1 M in hexane, 27.3 mL, 3.00 eq) over 15 min. The reaction was stirred for 1 h 30 before being diluted with Et_2O (100 mL) and quenched with H_2O (1 mL). After addition of a 3 M aqueous solution of NaOH (1 mL) and an additional H_2O (2.6 mL), the reaction was stirred for 15 min at room temperature. $MgSO_4$ was added and the solution was stirred for another 15 min. The solution was filtered and evaporated *in vacuo* until 150 mL of solvent was left.

MnO_2 was added (15.8 g, 182 mmol, 20.0 eq) to the solution of crude alcohol at room temperature. The reaction was stopped after 3 h and filtered through celite. The solvent was

[2] W. Tu, P. E. Floreancig, *Org. Lett.* **2007**, *9*, 2389–2392.

evaporated *in vacuo* and the crude product was purified by column chromatography (SiO_2, CH/EtOAc, 9:1). Pure product **15** was obtained as a thick colorless oil (2.83 g, 7.43 mmol, 85 %).

***R*$_f$** = 0.30 (SiO_2, CH/EtOAc, 10:1); **^{1}H-NMR** (700 MHz, $CDCl_3$): δ [ppm] = 9.99 (d, *J* = 8.1 Hz, 1H), 7.68 – 7.63 (m, 4H), 7.44 – 7.41 (m, 2H), 7.38 (ddt, *J* = 8.1, 6.6, 1.1 Hz, 4H), 5.89 – 5.84 (m, 1H), 3.68 (q, *J* = 6.0 Hz, 2H), 2.19 (ddd, *J* = 8.1, 7.0, 1.2 Hz, 2H), 2.14 (d, *J* = 1.3 Hz, 3H), 1.63 – 1.54 (m, 4H), 1.05 (s, 9H); **^{13}C-NMR** (176 MHz, $CDCl_3$): δ [ppm] = 191.3, 164.1, 135.6, 133.9, 129.6, 127.7, 127.4, 63.4, 40.3, 31.9, 26.9, 23.4, 19.2, 17.4; **HRMS (ESI+)** calculated for $C_{24}H_{33}O_2Si^+$ $[M+H]^+$: 381,2244 found : 381.2244.

Synthesis of ketone 16

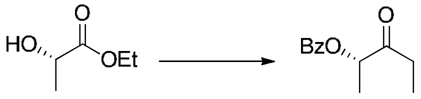

49 **16**

$C_{12}H_{14}O_3$
MW = 206,24 g/mol

To a solution of (*S*)-lactate (**49**) (4.8 mL, 42.3 mmol, 1.00 eq) and *N,O*-dimethylhydroxylamine (8.67 g, 97.5 mmol, 2.10 eq) in THF (100 mL) at -20 °C was added *i*-PrMgCl (2 M in THF, 89 mL, 177 mmol, 4.20 eq) over 30 min. The reaction was then stirred for 30 min and another 30 min at 0 °C. The reaction was quenched with a saturated solution of satured NH_4Cl (200 mL) and water (200 mL). After separation of the organic phase, the aqueous phase was extracted with Et_2O (3*100 mL). The combined organic layers were dried over $MgSO_4$ and evaporated *in vacuo* to give the clean Weinreb amine (4.00 g, 30.0 mmol, 72 %).

To a solution of Weinreb amine (4.00 g, 30.0 mmol, 1.00 eq) in THF (50 mL) was added EtMgBr (30 mL, 90 mmol, 3.00 eq) at 0 °C over 30 min. The reaction was stirred 30 min at room temperature. The reaction was quenched very carefully with a satured NH_4Cl solution (200 mL). After separation of the organic phase, the aqueous phase was extracted with Et_2O (3*100 mL). The combined organic layers were dried over $MgSO_4$ and concentrated to about 100 mL. Then Bz_2O (10.2 g, 45.1 mmol, 1.50 eq), DMAP (0.37 g, 3.01 mmol, 0.10 eq) and NEt_3 (7.70 mL, 57.0 mmol, 1.90 eq) were added and the reaction mixture was stirred overnight at room temperature. The reaction was quenched with ehtylenediamine (2.25 mL, 33.0 mmol, 1.10 eq) and then H_2O (100 mL). After separation of the organic phase, the aqueous phase was extracted with Et_2O (3*100 mL). The combined organic layers were dried over $MgSO_4$ and evaporated *in vacuo.* The crude product was purified by column chromatography (SiO_2, CH/EtOAc, 10:1) to give ketone **16** as colorless oil (2.60 g, 12.6 mmol, 42 %).

***R*$_f$** = 0.44 (SiO_2, CH/EtOAc, 4:1); **^{1}H-NMR** (500 MHz, $CDCl_3$): δ [ppm] = 8.14 – 8.05 (m, 2H), 7.63 – 7.55 (m, 1H), 7.54 – 7.41 (m, 2H), 5.36 (q, *J* = 7.0 Hz, 1H), 2.72 – 2.46 (m, 2H), 1.53 (d, *J* = 7.0 Hz, 3H), 1.10 (t, *J* = 7.3 Hz, 3H); **^{13}C-NMR** (125 MHz, $CDCl_3$): δ [ppm] = 208.6, 166.0, 133.4, 129.8, 129.5, 128.5, 75.2, 31.5, 16.2, 7.3. The spectroscopic data are in agreement with those previously reported.[3]

[3] I. Paterson, D. J. Wallace, C. J. Cowden, *Synthesis.* **1998**, 639–652.

Synthesis of *β*-hydroxyketone 17

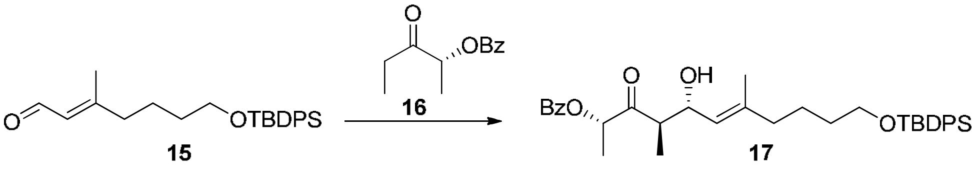

$C_{36}H_{46}O_5Si$
MW = 586,84 g/mol

To a solution of chlorodicyclohexylborane (1 M in hexane, 10.1 mL, 10.1 mmol, 1.50 eq) in Et_2O (50 mL) at -78 °C, was added DMEA (1.45 mL, 13.4 mmol, 2.00 eq) followed by ketone **19** (1.43 g, 6.69 mmol, 1.00 eq). The reaction was stirred for 2 h at 0 °C then cooled down again at -78 °C. Aldehyde **15** (2.86 g, 7.35 mmol, 1.10 eq) in Et_2O (4 mL) was added. The mixture was stirred for 1 h at -78 °C and then stored in the deep freezer (-20 °C) overnight. The reaction was quenched at 0 °C with MeOH (10 mL), pH 7 buffer (10 mL) and H_2O_2 (5 mL) and stirred for 1.5 h at room temperature. After separation of the organic phase, the aqueous phase was extracted with DCM (3*50 mL). The combined organic layers were dried over $MgSO_4$ and evaporated *in vacuo*. The crude product was purified by column chromatography (SiO_2, CH/EtOAc, 10:1 to 5:1) to afford alcohol **17** (3.23 g, 5.50 mmol, 82 %, *dr* > 20:1) as a thick colorless oil.

***R*$_f$** = 0.31 (SiO_2, CH/EtOAc, 5:1); $[\alpha]_D^{20}$ = + 18.0° (*c* = 0.44, $CHCl_3$); **^{1}H-NMR** (500 MHz, $CDCl_3$): δ [ppm] = 8.13 – 8.10 (m, 2H), 7.70 – 7.67 (m, 4H), 7.60 (ddt, *J* = 7.9, 7.0, 1.3 Hz, 1H), 7.49 – 7.37 (m, 8H), 5.48 (qd, *J* = 7.0, 1.6 Hz, 1H), 5.13 (dq, *J* = 9.3, 1.3 Hz, 1H), 4.60 (td, *J* = 9.0, 4.3 Hz, 1H), 3.68 (t, *J* = 5.9 Hz, 2H), 2.89 (dq, *J* = 8.6, 7.1 Hz, 1H), 2.02 (d, *J* = 4.3 Hz, 2H), 1.70 (d, *J* = 1.3 Hz, 3H), 1.59 (dd, *J* = 7.0, 1.2 Hz, 3H), 1.56 – 1.48 (m, 4H), 1.15 (d, *J* = 7.1 Hz, 3H), 1.07 (d, *J* = 1.5 Hz, 9H); **^{13}C-NMR** (176 MHz, $CDCl_3$): δ [ppm] = 211.3, 165.9, 140.9, 135.6, 134.1, 133.6, 129.8, 129.6, 128.5, 127.6, 125.1, 75.0, 70.4, 63.7, 60.4, 48.9, 39.3, 32.1, 26.9, 23.9, 21.1, 19.2, 16.8, 15.6, 14.2; **HRMS (ESI+)** calculated for $C_{36}H_{46}O_5SiNa^+$ $[M+Na]^+$: 609.3007 found : 609.3007.

Synthesis of ketone 50

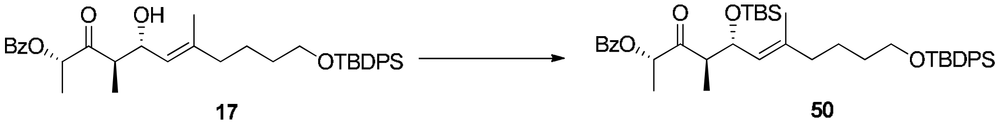

$C_{42}H_{60}O_5Si_2$
MW : 701,11 g/mol

To a stirred solution of alcohol **17** (3.23 g, 5.50 mmol, 1.00 eq) in DCM (120 mL) at -78 °C was added 2,6-lutidine (1.26 mL, 10.9 mmol, 2.00 eq) and TBSOTf (1.88 mL, 8.17 mmol, 1.50 eq). The reaction was stirred 1.5 h and quenched with a saturated solution of $NaHCO_3$ (80 mL) at 0 °C. After separation of the organic layer, the aqueous layer was extracted with DCM (80 mL). The organic layers were combined, dried over $MgSO_4$ and evaporated *in vacuo*. The crude product was purified by column chromatography (SiO_2, CH/EtOAc, 10:1) to give **50** as a thick colorless oil (3.64 g, 5.19 mmol, 94 %).

***R*$_f$** = 0.62 (SiO_2, CH/EtOAc, 5:1); $[\alpha]_D^{20}$ = + 3.1° (*c* = 0.33, $CHCl_3$, 20 °C); **^{1}H-NMR** (700 MHz, $CDCl_3$): δ [ppm] = 8.11 – 8.06 (m, 2H), 7.68 – 7.64 (m, 4H), 7.57 (ddt, *J* = 8.7, 7.3, 1.3 Hz, 1H), 7.47 – 7.43 (m, 2H), 7.43 – 7.39 (m, 2H), 7.39 – 7.35 (m, 4H), 5.42 (qd, *J* = 7.0, 0.9 Hz, 1H), 5.01 (dq, *J* = 9.6, 1.2 Hz, 1H), 4.60 (td, *J* = 9.4, 1.0 Hz, 1H), 3.66 (t, *J* = 6.1 Hz, 2H), 2.85 (ddt, *J* = 9.6, 7.8, 6.7 Hz, 1H), 2.00 – 1.96 (m, 2H), 1.64 (t, *J* = 1.0 Hz, 3H), 1.57 – 1.46 (m, 9H), 1.05 – 1.03 (m, 9H), 1.00 (dd, *J* = 7.1, 1.0 Hz, 3H), 0.82 – 0.80 (m, 9H), -0.01 (s, 6H); **^{13}C-NMR** (176 MHz, $CDCl_3$): δ [ppm] = 209.6, 165.8, 138.0, 135.6,

134.1, 133.2, 129.8, 129.5, 128.4, 127.6, 126.7, 75.4, 71.8, 63.7, 49.6, 39.3, 32.3, 26.9, 25.9, 24.0, 19.2, 18.0, 17.0, 15.1, 14.0, -4.4, -4.9; **HRMS (ESI+)** calculated for $C_{42}H_{60}O_5Si_2Na^+$ $[M+Na]^+$: 723.3871, found : 732.3875.

Synthesis of aldehyde 18

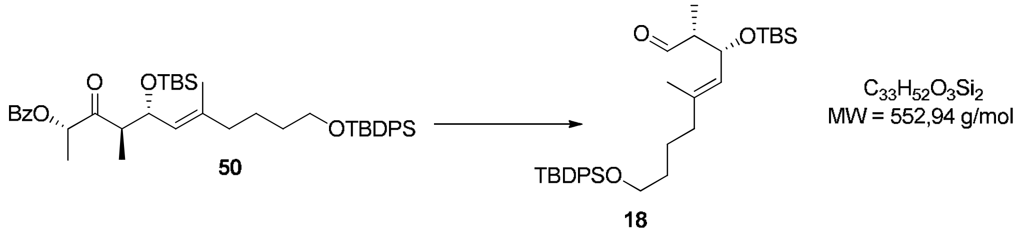

To a solution of protected alcohol (3.64 g, 5.19 mmol, 1.00 eq) in THF (120 mL) at -78 °C was added $LiBH_4$ (1.68 g, 77.1 mmol, 15.0 eq) in one portion. After stirring 2 h at -78 °C, the mixture was stirred 3 days at room temperature. At 0 °C, water (40 mL) was added followed by careful addition of a saturated solution of NH_4Cl (5 mL). The mixture was poured to a mixture of water and Et_2O (1:1, 100 mL). After separation of the organic layer, the aqueous layer was extracted with Et_2O (3*50 mL). The organic layers were combined, dried $MgSO_4$ and evaporated *in vacuo*. Purification by column chromatography (SiO_2, CH/EtOAc, 4:1) yielded to the diol (3.01 g, 5.09 mmol, 98 %, *dr* = 4:1) as a thick colorless oil.

To a solution of diol (3.01 g, 5.09 mmol, 1.00 eq) in dioxane (80 mL) and water (40 mL) at 0 °C was added $NaIO_4$ (2.68 g, 12.5 mmol, 2.50 eq) portionwise. The reaction mixture was vigourously stirred overnight. The reaction was diluted with DCM (80 mL) and quenched with water (50 mL). After separation of the organic layer, the aqueous layer was extracted with DCM (3*100 mL). The organic layers were combined, dried over $MgSO_4$ and evaporated *in vacuo*. After column chromatography (SiO_2, CH/EtOAc, 9:1), aldehyde **18** was obtained (2.33 g, 4.22 mmol, 83 %) as a colorless oil.

***R*f** = 0.65 (SiO_2, CH/EtOAc, 5:1); $[\alpha]_D^{20}$ = - 17.4° (*c* = 0.39, $CHCl_3$); **¹H-NMR** (700 MHz, CD_2Cl_2): δ [ppm] = δ 9.73 (d, *J* = 2.9 Hz, 1H), 7.68 – 7.65 (m, 4H), 7.44 – 7.41 (m, 2H), 7.38 (ddt, *J* = 8.1, 6.7, 1.1 Hz, 4H), 5.16 (dp, *J* = 9.1, 1.3 Hz, 1H), 4.58 – 4.52 (m, 1H), 3.68 (t, *J* = 6.0 Hz, 2H), 2.42 – 2.35 (m, 1H), 2.06 – 1.97 (m, 2H), 1.65 (d, *J* = 1.4 Hz, 3H), 1.60 – 1.50 (m, 7H), 1.04 (s, 9H), 0.94 (d, *J* = 7.0 Hz, 3H), 0.85 (d, *J* = 2.7 Hz, 9H), -0.02 (s, 3H), -0.04 (s, 3H); **¹³C-NMR** (176 MHz, CD_2Cl_2): δ [ppm] = 204.7, 137.8, 135.5, 134.1, 129.5, 127.6, 126.4, 71.2, 63.7, 53.5, 39.2, 32.2, 26.6, 25.5, 23.9, 19.1, 17.9, 16.5, 10.3, -4.2, -5.4; **HRMS (ESI+)** calculated for $C_{33}H_{52}O_4Si_2Na^+$ $[M+Na]^+$: 575.3347, found : 575.3347.

Synthesis of phosphonate 19

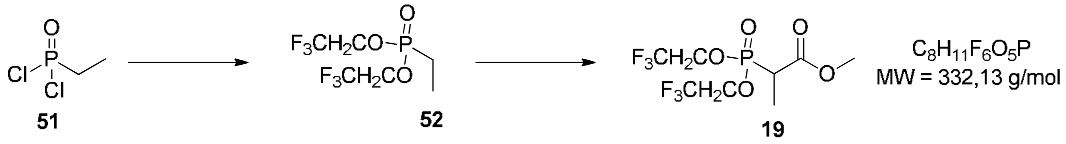

To a solution of trifluoroethanol (15 mL, 0.21 mol, 2.20 eq) and NEt_3 (29 mL, 0.21 mol, 2.20 eq) in THF (300 mL) at 0 °C was added ethylphosphonic dichloride (**51**) (10 mL, 94 mmol, 1.00 eq) over 10 min. The reaction was stirred for 2 h at room temperature and filtered. The solvent was

removed *in vacuo* and the product **52** was purified by distillation (4 mbar, 100 °C) as a colorless oil (17.1 g, 62.0 mmol, 66 %).

To a solution of HMDS (14.8 mL, 71.0 mmol, 2.30 eq) in THF (110 mL) at 0 °C was added *n*-BuLi (2.5 M in hexane, 44.8 mL, 31.0 mmol, 2.30 eq) over 30 min. The solution was stirred for 15 min and then cooled down to -78 °C. A solution of methylchloroformat (2.90 mL, 74.2 mmol, 1.20 eq) and bis(2,2,2-trifluoroethyl) ethylphosphonate **52** (8.50 g, 62.0 mmol, 1.00 eq) in THF (50 mL) was added slowly over 2 h. The reaction mixture was then stirred for 20 min at 0 °C and carefully quenched with 5 M HCl aqueous solution (50 mL). After separation of the organic layer, the aqueous layer was extracted with DCM (3*50 mL). The organic layers were combined, dried over $MgSO_4$ and evaporated *in vacuo*. After column chromatography (SiO_2, CH/EtOAc, 7:3), the desired ester **19** was obtained (5.73 g, 17.2 mmol, 55 %) as a colorless oil.

^{1}H-NMR (500 MHz, $CDCl_3$): δ [ppm] = 4.46 (qd, *J* = 8.9, 8.5, 7.6 Hz, 4H), 3.80 (d, *J* = 0.5 Hz, 3H), 3.23 (dq, *J* = 22.8, 7.4 Hz, 1H), 1.54 (dd, *J* = 19.3, 7.4 Hz, 3H); **^{13}C-NMR** (125 MHz, $CDCl_3$): δ [ppm] =169.1 (d, *J* = 3.1 Hz), 122.3 (ddq, *J* = 2.6, 8.5, 277.3 Hz), 62.7 (ddq, *J* = 1.8, 6.2, 38.9 Hz), 53.2, 30.5 (d, *J* = 141.2 Hz), 11.7 (d, *J* = 6.6 Hz); **^{31}P-NMR** (203 MHz, $CDCl_3$) : δ [ppm] =26.9.The spectroscopic data are in agreement with those previously reported.[123]

Synthesis of ester 19

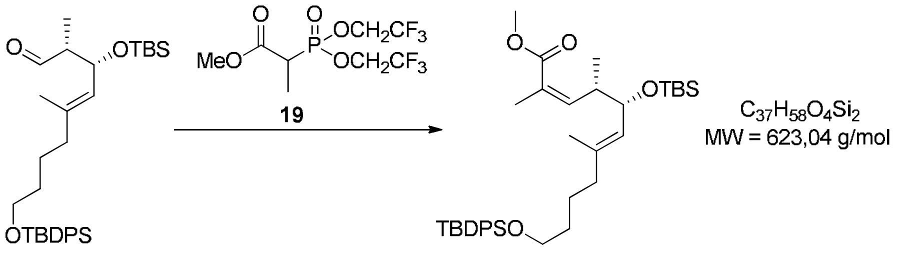

To a solution of 18-c-6 (2.52 g, 9.55 mmol, 2.30 eq) and methyl 2-(bis(2,2,2-trifluoroethoxy)phosphoryl)propanoate **19** (1.93 g, 5.82 mmol, 1.40 eq) in THF (100 mL) at -78 °C was added KHMDS (0.5 M in toluene, 10.8 mL, 5.40 mmol, 1.30 eq) over 10 min. The reaction was stirred of 30 min then aldehyde **18** (2.30 g, 4.15 mmol, 1.00 eq) in THF (4 mL) was added dropwise and the reaction was stirred for another 2 h at -78 °C. The reaction was quenched with a saturated solution of $NaHCO_3$ (100 mL) at 0 °C. After separation of the organic layer, the aqueous layer was extracted with DCM (3*80 mL). The organic layers were combined, dried over $MgSO_4$ and evaporated *in vacuo*. After column chromatography (SiO_2, CH/EtOAc, 9:1), ester **20** was obtained (2.38 g, 3.82 mmol, 92 %, *dr* >20:1,) as a colorless oil.

R_f = 0.56 (SiO_2, CH/EtOAc, 10:1); $[\alpha]_D^{20}$ = + 9.1° (*c* = 0.32, $CHCl_3$); **^{1}H-NMR** (500 MHz, $CDCl_3$): δ [ppm] = 7.75 – 7.66 (m, 4H), 7.49 – 7.38 (m, 6H), 5.84 (dq, *J* = 10.1, 1.4 Hz, 1H), 5.09 (dq, *J* = 9.1, 1.3 Hz, 1H), 4.21 (dd, *J* = 9.0, 5.9 Hz, 1H), 3.72 (s, 3H), 3.68 (t, *J* = 6.0 Hz, 2H), 3.26 – 3.15 (m, 1H), 1.98 (t, *J* = 7.3 Hz, 2H), 1.90 (d, *J* = 1.4 Hz, 3H), 1.60 (d, *J* = 1.3 Hz, 3H), 1.58 – 1.48 (m, 4H), 1.07 (s, 9H), 0.96 (dd, *J* = 6.9, 2.6 Hz, 3H), 0.88 (s, 9H), -0.02 (s, 3H), -0.04 (s, 3H); **^{13}C-NMR** (125 MHz, $CDCl_3$): δ [ppm] = 146.0, 135.8, 135.6, 134.1, 129.5, 127.6, 127.3, 126.4, 73.0, 63.7, 51.1, 40.8, 39.3, 32.2, 26.9, 25.8, 24.0, 21.0, 19.2, 18.1, 16.6, 16.1, -4.1, -4.9; **HRMS (ESI+)** calculated for $C_{37}H_{58}O_4Si_2Na^+$ $[M+Na]^+$: 645.3766, found : 645.3766.

Synthesis of aldehyde 21

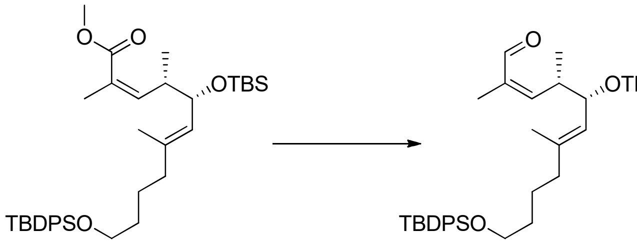

20 **21**

$C_{36}H_{56}O_3Si_2$
MW = 593,01 g/mol

To a solution of ester **20** (2.38 g, 3.82 mmol, 1.00 eq) in DCM (50 mL) at -78°C was added DIBALH (1 M in hexane, 11.4 mL, 11.4 mmol, 3.00 eq) dropwise. The mixture was stirred for 1 h and warmed up to 0 °C for 45 min. DCM (50 mL) was added followed by H_2O (0.45 mL), a 3 M aqueous solution of NaOH (0.45 mL) and H_2O (1.1 mL). After stirring 15 min at room temperature, $MgSO_4$ was added and the mixture was stirred an additionnal 15 min. After filtration, the solvents were removed *in vacuo*.

The crude product was directly diluted in DCM (40 mL) and MnO_2 (6.64 g, 76.4 mmol, 20.0 eq) was added. The reaction was stirred overnight at room temperature. The solution was filtered through celite and the solvent was evaporated *in vacuo*. Purification by column chromatography (SiO_2, CH/EtOAc, 9:1) yielded to aldehyde **21** (2.12 g, 3.54 mmol, 94 % over two steps) as a colorless oil.

***R*$_f$** = 0.56 (SiO_2, CH/EtOAc, 10:1); $[\alpha]_D^{20}$ = + 11.8° (*c* = 0.51, $CHCl_3$); **^{1}H-NMR** (500 MHz, $CDCl_3$): δ [ppm] = 10.04 (d, *J* = 0.5 Hz, 1H), 7.69 – 7.63 (m, 4H), 7.45 – 7.34 (m, 6H), 6.34 (dq, *J* = 10.9, 1.3 Hz, 1H), 5.06 (dq, *J* = 9.3, 1.4 Hz, 1H), 4.19 – 4.13 (m, 1H), 3.66 (t, *J* = 6.0 Hz, 2H), 3.17 (dp, *J* = 10.7, 6.7 Hz, 1H), 2.02 – 1.95 (m, 2H), 1.77 (d, *J* = 1.4 Hz, 3H), 1.62 (d, *J* = 1.3 Hz, 3H), 1.54 – 1.46 (m, 4H), 1.04 (s, 9H), 1.00 (d, *J* = 6.7 Hz, 3H), 0.82 (d, *J* = 2.6 Hz, 9H), -0.02 (s, 3H), -0.04 (s, 3H); **^{13}C-NMR** (125 MHz, $CDCl_3$): δ [ppm] = 192.1, 152.6, 136.0, 135.9, 135.5, 134.1, 129.5, 127.6, 127.0, 73.0, 63.6, 39.3, 38.4, 32.2, 26.9, 25.7, 23.9, 19.2, 18.1, 17.2, 16.8, 16.6, -4.1, -4.9; **HRMS (ESI+)** calculated for $C_{36}H_{56}O_3Si_2Na^+$ $[M+Na]^+$: 615,3660 found : 615.3664.

Synthesis of ester 22

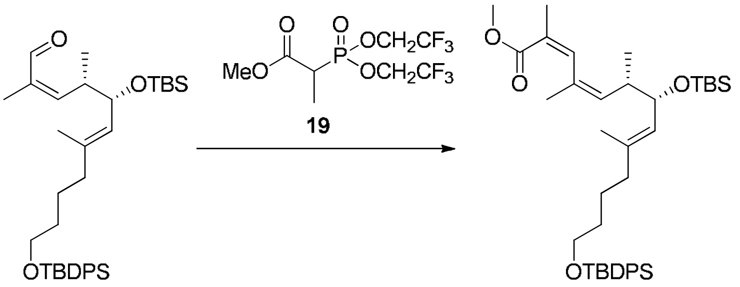

21 **22**

$C_{40}H_{62}O_4Si_2$
MW = 663,10 g/mol

To a solution of 18-c-6 (2.13 g, 8.14 mmol, 2.30 eq) and methyl 2-(bis(2,2,2-trifluoroethoxy)phosphoryl)propanoate **19** (1.64 g, 4.96 mmol, 1.40 eq) in THF (100 mL) at -78 °C was added KHMDS (0.5 M in toluene, 9.2 mL, 4.60 mmol, 1.30 eq) over 10 min. The reaction was stirred of 30 min then aldehyde **21** (2.12 g, 3.54 mmol, 1.00 eq) in THF (4 mL) was added dropwise and the reaction was stirred for another 4 h at -78 °C. The reaction was quenched with a saturated solution of $NaHCO_3$ (80 mL) at 0 °C. After separation of the organic layer, the aqueous layer was extracted with DCM (3*80 mL). The organic layers were combined, dried over $MgSO_4$ and evaporated *in vacuo*. After

column chromatography (SiO_2, CH/EtOAc, 9:1), ester **22** was obtained (2.17 g, 3.27 mmol, 93 %, *dr* >20:1) as a colorless oil.

***R*f** = 0.56 (SiO_2, CH/EtOAc, 10:1); $[\alpha]_D^{20}$ = + 28.1° (*c* = 0.31, $CHCl_3$); **^{1}H-NMR** (500 MHz, $CDCl_3$): δ [ppm] = 7.68 – 7.66 (m, 4H), 7.42 – 7.36 (m, 6H), 6.41 – 6.38 (m, 1H), 5.09 (ddt, *J* = 11.8, 9.0, 1.4 Hz, 2H), 4.10 (dd, *J* = 9.0, 5.9 Hz, 1H), 3.70 (s, 3H), 3.66 (t, *J* = 6.1 Hz, 2H), 2.40 (dq, *J* = 10.0, 6.5 Hz, 1H), 1.97 – 1.93 (m, 5H), 1.77 – 1.74 (m, 3H), 1.58 (d, *J* = 1.3 Hz, 3H), 1.55 – 1.43 (m, 4H), 1.04 (d, *J* = 1.5 Hz, 9H), 0.86 – 0.83 (m, 13H), -0.01 (s, 3H), -0.04 (s, 3H); **^{13}C-NMR** (125 MHz, $CDCl_3$): δ [ppm] = 169.8, 135.6, 134.1, 133.5, 131.4, 129.5, 127.9, 127.6, 127.3, 73.1, 63.7, 51.4, 40.6, 39.3, 62.2, 26.9, 25.8, 24.0, 22.2, 21.2, 19.2, 18.2, 16.6, 16.0, -4.3, -4.9; **HRMS (ESI+)** calculated for $C_{40}H_{62}O_4Si_2Na^+$ $[M+Na]^+$: 686.4079 found : 686.4097.

Synthesis of aldehyde 23

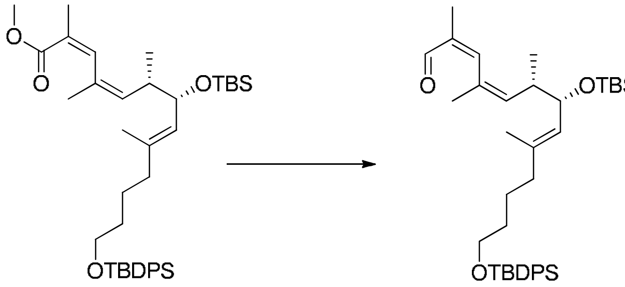

22 23

$C_{39}H_{60}O_3Si_2$
MW = 633,08 g/mol

To a solution of ester **22** (2.17 g, 3.27 mmol, 1.00 eq) in DCM (70 mL) at -78 °C was added DIBALH (1 M in hexane, 9.81 mL, 9.81 mmol, 3.00 eq) dropwise. The mixture was stirred for 1 h and warmed up to 0 °C. After dilution with DCM (50 mL), H_2O (0.4 mL) was added followed by a 3 M aqueous solution of NaOH (0.4 mL) and H_2O (1 mL). After stirring 15 min at room temperature, $MgSO_4$ was added and the mixture was stirred an additionnal 15 min. After filtration, the solvents were removed *in vacuo*.

The crude product was directly diluted in DCM (40 mL) and MnO_2 (5.69 g, 65.4 mmol, 20.0 eq) was added. The reaction was stirred overnight at room temperature. The solution was filtered through celite and the solvent was evaporated *in vacuo*. Purification by column chromatography (SiO_2, CH/EtOAc, 9:1) yielded to aldehyde **23** (1.96 g, 3.09 mmol, 95 % over two steps) as a colorless oil.

***R*f** = 0.61 (SiO_2, CH/EtOAc, 20:1); $[\alpha]_D^{20}$ = + 11.3° (*c* = 0.77, $CHCl_3$); **^{1}H-NMR** (500 MHz, $CDCl_3$): δ [ppm] = 9.90 (s, 1H), 7.70 – 7.64 (m, 5H), 7.44 – 7.35 (m, 7H), 6.92 (dd, *J* = 2.3, 1.2 Hz, 1H), 5.40 (dq, *J* = 10.2, 1.4 Hz, 1H), 5.03 (dq, *J* = 9.0, 1.3 Hz, 1H), 4.09 (dd, *J* = 8.9, 6.2 Hz, 1H), 3.66 (t, *J* = 6.1 Hz, 2H), 2.35 – 2.27 (m, 1H), 1.98 – 1.94 (m, 2H), 1.88 (q, *J* = 2.1, 1.6 Hz, 2H), 1.81 (d, *J* = 1.4 Hz, 2H), 1.56 (d, *J* = 1.3 Hz, 2H), 1.53 – 1.45 (m, 3H), 1.05 – 1.04 (m, 9H), 0.87 – 0.83 (m, 12H) -0.01 (s, 3H), -0.04 (s, 3H); **^{13}C-NMR** (125 MHz, $CDCl_3$): δ [ppm] = 193.4, 147.0, 136.2, 135.8, 135.6, 134.1, 129.5, 127.6, 127.4, 73.2, 63.7, 40.9, 39.3, 32.2, 26.9, 25.8, 25.0, 24.0, 19.2, 18.1, 16.6, 16.3, 15.9, -4.2, -4.9; **HRMS** calculated for $C_{39}H_{60}O_3Si_2Na^+$ $[M+Na]^+$: 655.3973 found : 655.3973.

Synthesis of ester 24

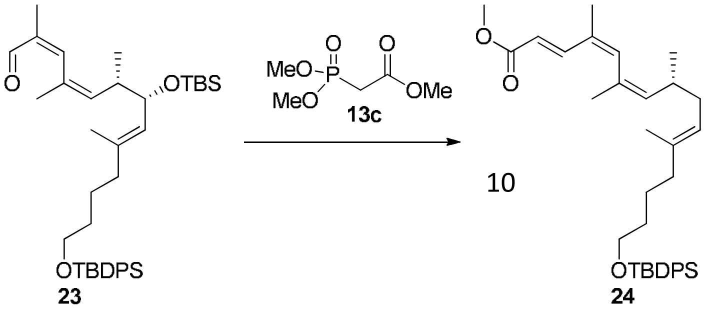

23 24

$C_{42}H_{64}O_4Si_2$
MW = 689,13 g/mol

To a solution of trimethyl phosphonoacetate (**13c**) (0.75 mL, 4.64 mmol, 1.50 eq) and DMPU (0.56 mL, 4.64 mmol, 1.50 eq) in THF (80 mL) at 0 °C was added *n*-BuLi (1.6 M in hexane, 2.7 mL, 4.33 mmol, 1.40 eq). The mixture was stirred for 30 min then aldehyde **23** (1.96 g, 3.09 mmol, 1.00 eq) in THF (3 mL) was added dropwise. After stirring for 2 h at 0 °C, the reaction was stirred overnight at room temperature. The reaction was quenched with buffer pH 7 (15 mL) and H_2O (50 mL) at 0 °C. After separation of the organic layer, the aqueous layer was extracted with Et_2O (3*100 mL). The organic layers were combined, dried over $MgSO_4$ and evaporated *in vacuo*. Purification by column chromatography (SiO_2, CH/EtOAc, 9:1) yielded to ester **24** (2.03 g, 2.94 mmol, 95 %) as a slightly yellow oil.

$\boldsymbol{R}_f$ = 0.53 (SiO_2, CH/EtOAc, 20:1); $[\boldsymbol{\alpha}]_{\boldsymbol{D}}^{\mathbf{20}}$ = + 39.3° (*c* = 0.41, $CHCl_3$); **^{1}H-NMR** (500 MHz, $CDCl_3$): δ [ppm] = 7.68 – 7.62 (m, 5H), 7.41 – 7.35 (m, 6H), 6.17 (td, *J* = 1.5, 0.8 Hz, 1H), 5.86 (dd, *J* = 15.8, 0.7 Hz, 1H), 5.23 – 5.17 (m, 1H), 5.05 (dq, *J* = 9.2, 1.3 Hz, 1H), 4.08 (dd, *J* = 9.0, 5.8 Hz, 1H), 3.74 (s, 3H), 3.66 (td, *J* = 6.0, 2.5 Hz, 3H), 2.30 – 2.22 (m, 1H), 1.98 – 1.93 (m, 2H), 1.89 (d, *J* = 1.4 Hz, 3H), 1.80 (dd, *J* = 1.4, 0.7 Hz, 3H), 1.57 (d, *J* = 1.3 Hz, 3H), 1.54 – 1.46 (m, 5H), 1.04 (d, *J* = 2.0 Hz, 11H), 0.88 – 0.86 (m, 3H), 0.86 – 0.82 (m, 9H), -0.06 (s, 5H); **^{13}C-NMR** (125 MHz, $CDCl_3$): δ [ppm] = 167.8, 143.3, 138.3, 135.5, 134.5, 134.1, 131.3, 131.2, 129.5, 127.6, 127.1, 117.8, 72.9, 63.7, 51.4, 40.8, 39.3, 62.2, 26.8, 25.8, 24.5, 24.0, 19.8, 19.2, 18.1, 16.6, 15.5, -4.3, -4.9; **HRMS (ESI+)** calculated for $C_{42}H_{64}O_4Si_2Na^+$ $[M+Na]^+$: 711.4235, found : 711.4238.

Synthesis of aldehyde 25

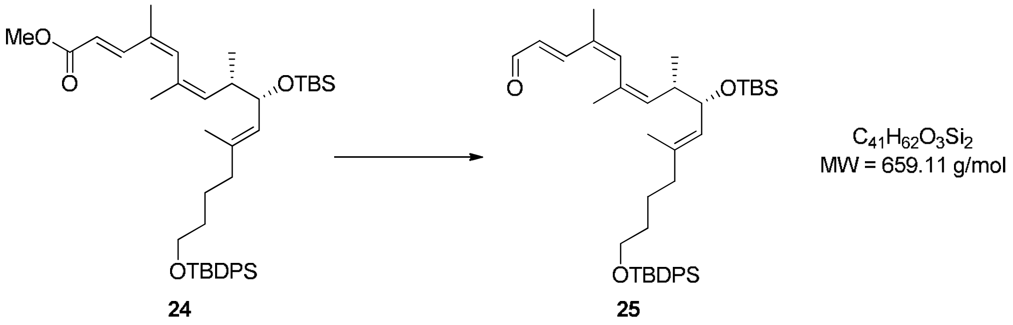

To a solution of ester **24** (582 mg, 0.84 mmol, 1.00 eq) in DCM (15 mL) at -78 °C was added DIBALH (1 M in hexane, 2.5 mL, 2.52 mmol, 3.00 eq) dropwise. The mixture was stirred for 1 h and warmed up to 0 °C. The solution was diluted with DCM (20 mL), H_2O (0.1 mL) followed by a 3 M aqueous solution of NaOH (0.1 mL) and H_2O (0.1 mL) were added. After stirring 15 min at room temperature, $MgSO_4$ was added and the mixture was stirred an additionnal 15 min. After filtration, the solvents were removed *in vacuo*.

The crude product was directly diluted in DCM (6 mL) and MnO_2 (1.46 g, 16.8 mmol, 20.0 eq) was added. The reaction was stirred overnight at room temperature. The solution was filtered through celite and the solvent was evaporated *in vacuo*. Purification by column chromatography (SiO_2, CH/EtOAc, 30:1) yielded to aldehyde **25** (540 mg, 3.09 mmol, 94 % over two steps) as a yellow oil.

***R*~f~** = 0.50 (SiO_2, CH/EtOAc, 10:1); $[\alpha]_D^{20}$ = + 17.0° (*c* = 0.37, $CHCl_3$); **^{1}H-NMR** (700 MHz, $CDCl_3$): δ [ppm] = 9.61 (d, *J* = 7.9 Hz, 1H), 7.70 – 7.68 (m, 5H), 7.53 (dd, *J* = 15.7, 0.8 Hz, 1H), 7.45 – 7.43 (m, 2H), 7.41 – 7.38 (m, 5H), 6.29 (dd, *J* = 2.2, 1.2 Hz, 1H), 6.18 (ddt, *J* = 15.7, 7.8, 0.7 Hz, 1H), 5.32 (dt, *J* = 10.2, 1.5 Hz, 1H), 5.08 – 5.05 (m, 1H), 4.10 (dd, *J* = 8.9, 6.2 Hz, 1H), 3.68 (t, *J* = 6.1 Hz, 2H), 2.29 (dp, *J* = 10.3, 6.8 Hz, 1H), 1.97 (t, *J* = 7.4 Hz, 2H), 1.95 (d, *J* = 1.3 Hz, 3H), 1.86 – 1.85 (m, 3H), 1.59 (dd, *J* = 1.3, 0.7 Hz, 3H), 1.57 – 1.54 (m, 2H), 1.49 (qd, *J* = 7.1, 3.4 Hz, 2H), 1.06 (d, *J* = 0.6 Hz, 10H), 0.87 (d, *J* = 0.6 Hz, 12H); **^{13}C-NMR** (176 MHz, CD_2Cl_2): δ [ppm] = 194.0, 150.8, 139.9, 135.8, 135.5, 134.8, 134.2, 131.7, 131.2, 129.5, 128.9, 127.6, 127.3, 73.1, 63.8, 40.7, 39.3, 32.2, 26.6, 25.6, 24.1, 24.0, 19.4, 19.1, 18.0, 16.4, 15.6, -4.5, -5.2; **HRMS (ESI+)** calculated for $C_{41}H_{62}O_3Si_2Na^+$ $[M+Na]^+$: 681.4130 found : 681.4130.

Synthesis of *β*-hydroxyketone 53

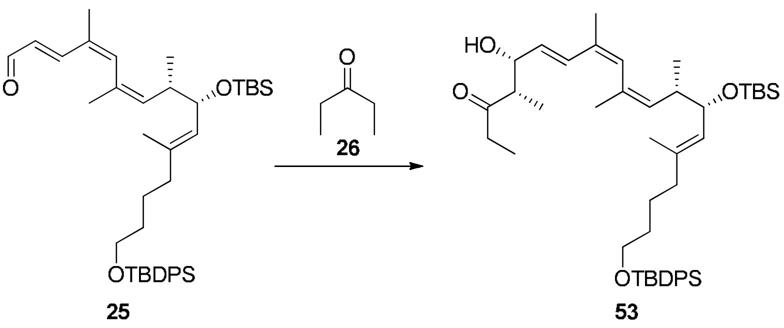

$C_{46}H_{72}O_4Si_2$
MW = 745,25 g/mol

Stock solution: (-)-Ipc_2BH (1.09 g, 3.81 mmol, 1.00 eq) was dissolved in anhydrous hexane (0.88 mL) and cooled down at 0 °C. Triflic acid (336 µL, 3.81 mmol, 1.00 eq) was added dropwise and the mixture was stirred at room temperature until no Ipc_2BH crystals were seen.

The triflate solution (1.9 M, 0.55 mL, 1.05 mmol, 1.30 eq) was diluted in DCM (4 mL) and cooled down to -78 °C. DIEA (360 µL, 2.10 mmol, 3.00 eq) was added dropwise followed by diethylketone (**26**) (100 µL, 0.98 mmol, 1.40 eq). The reaction mixture was stirred for 3 h at this temperature. Then aldehyde **25** (460 mg, 0.70 mmol, 1.00 eq) in DCM (1.5 mL) was added, the reaction was stirred for 1 h at -78 °C and stored in the freezer (-20 °C) overnight. Buffer pH7 (4 mL), MeOH (4 mL) and H_2O_2 (2 mL) were added and the solution was stirred for 1 h at room temperature. After separation of the organic layer, the aqueous layer was extracted with DCM (3*10 mL). The organic layers were combined, dried over $MgSO_4$ and evaporated *in vacuo*. The crude was purified by column chromatograpy (SiO_2, CH/EtOAc 30:1) to give alcohol **53** as a slightly yellow oil (310 mg, 0.42 mmol, 61 %, *dr* = 10.1) and the starting material **25** (90 mg, 0.14 mmol, 20 %, 84 % brsm).

***R*~f~** = 0.18 (SiO_2, CH/EtOAc, 10:1); $[\alpha]_D^{20}$ = + 32.2° (*c* = 0.40, $CHCl_3$); **^{1}H-NMR** (500 MHz, $CDCl_3$): δ [ppm] = δ 7.69 – 7.65 (m, 4H), 7.44 – 7.36 (m, 6H), 6.59 – 6.52 (m, 1H), 5.85 (s, 1H), 5.65 – 5.59 (m, 1H), 5.12 (dq, *J* = 9.9, 1.5 Hz, 1H), 5.06 (ddq, *J* = 9.1, 3.1, 1.4 Hz, 1H), 4.43 (dtd, *J* = 6.5, 3.8, 1.3 Hz, 1H), 4.14 – 4.09 (m, 1H), 3.67 (t, *J* = 6.1 Hz, 2H), 2.67 (qd, *J* = 7.2, 3.9 Hz, 1H), 2.57 – 2.54 (m, 1H), 2.54 – 2.45 (m, 2H), 2.37 – 2.29 (m, 1H), 2.00 – 1.94 (m, 2H), 1.84 (d, *J* = 1.4 Hz, 3H), 1.77 – 1.74 (m, 3H), 1.57 (d, *J* = 1.4 Hz, 3H), 1.09 – 1.07 (m, 3H), 1.03 (s, 9H), 1.00 (t, *J* = 7.2 Hz, 3H), 0.87 – 0.83 (m, 12H), -0.01 (s, 3H), -0.02 – -0.05 (m, 3H); **^{13}C-NMR** (125 MHz, $CDCl_3$): δ [ppm] = 215.2, 135.5, 134.2, 132.6, 131.9, 130.6, 130.1, 129.5, 129.4, 127.6, 127.2, 73.0, 72.9, 63.8, 50.8, 40.5, 39.3,35.3, 32.2, 26.6, 25.6, 24.3, 24.0, 19.9, 19.1, 18.0, 16.4, 15.3, 10.7, 7.3, -4.6, -5.2; **HRMS (ESI+)** calculated for $C_{46}H_{72}O_4Si_2Na^+$ $[M+Na]^+$: 767.4861 found : 767.4867.

Synthesis of ketone 27

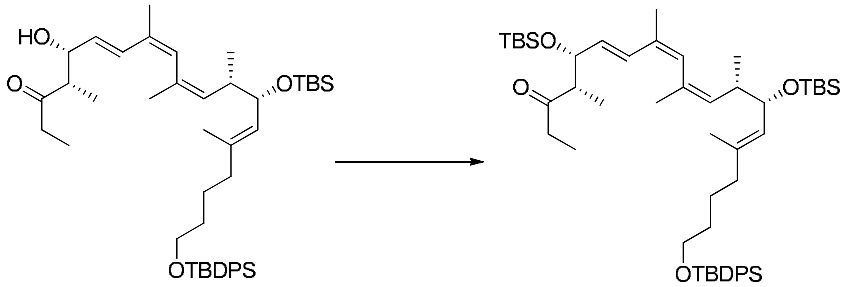

53 **27**

$C_{52}H_{86}O_4Si_3$
MW = 859,51 g/mol

To a stirred solution of *β*-hydroxyketone **53** (370 mg, 0.50 mmol, 1.00 eq) in DCM (8 mL) at -78 °C was added 2,6-lutidine (0.11 mL, 1.00 mmol, 2.00 eq) and TBSOTf (0.17 mL, 0.75 mmol, 1.50 eq). The reaction was stirred for 1.5 h and quenched with a saturated solution of $NaHCO_3$ (10 mL) at 0 °C. After separation of the organic layer, the aqueous layer was extracted with DCM (3*10 mL). The organic layers were combined, dried over $MgSO_4$ and evaporated *in vacuo*. The crude product was purified by column chromatography (SiO_2, CH/EtOAc, 30:1) to give main fragment **27** as a thick colorless oil (383 mg, 0.44 mmol, 90 %).

$\boldsymbol{R}_f$ = 0.18 (SiO_2, CH/EtOAc, 10:1); $[\alpha]_D^{20}$ = + 56.2° (*c* = 0.34, $CHCl_3$); **^{1}H-NMR** (700 MHz, CD_2Cl_2): δ [ppm] = 7.68 – 7.66 (m, 4H), 7.43 – 7.41 (m, 2H), 7.38 (ddt, *J* = 8.2, 6.7, 1.2 Hz, 4H), 6.44 – 6.39 (m, 1H), 5.93 – 5.91 (m, 1H), 5.60 – 5.54 (m, 1H), 5.11 (dq, *J* = 9.7, 1.5 Hz, 1H), 5.08 (dp, *J* = 9.0, 1.2 Hz, 1H), 4.35 (ddd, *J* = 6.9, 5.8, 1.2 Hz, 0H), 4.31 (ddd, *J* = 7.7, 5.9, 1.0 Hz, 1H), 4.14 – 4.10 (m, 1H), 3.68 (t, *J* = 6.2 Hz, 2H), 2.70 (qd, *J* = 6.9, 5.7 Hz, 1H), 2.53 – 2.38 (m, 2H), 2.37 – 2.31 (m, 1H), 2.00 – 1.96 (m, 2H), 1.84 – 1.81 (m, 3H), 1.78 – 1.76 (m, 3H), 1.58 (d, *J* = 1.4 Hz, 2H), 1.57 – 1.54 (m, 2H), 1.50 (ddd, *J* = 8.5, 6.7, 4.7 Hz, 2H), 1.04 – 1.02 (m, 12H), 0.95 (t, *J* = 7.2 Hz, 3H), 0.87 (s, 9H), 0.85 (d, *J* = 4.4 Hz, 12H), 0.03 (s, 3H), -0.01 (d, J = 4.4 Hz, 6H), -0.03 – -0.04 (m, 3H); **^{13}C-NMR** (176 MHz, $CDCl_3$): δ [ppm] = 212.6, 135.5, 135.4, 134.2, 132.7, 132.1, 131.9, 130.6, 130.4, 129.7, 129.5, 127.6, 127.2, 76.0, 72.9, 63.8, 52.9, 40.5, 39.3, 36.5, 32.2, 26.6, 25.7, 25.6, 24.5, 24.0, 20.1, 19.1, 18.0, 19.7, 16.4, 15.4, 12.1, 7.2, -4.3, -4.6, -5.1, -5.2; **HRMS (ESI+)** calculated for $C_{52}H_{88}O_4Si_3Na^+$ $[M+Na]^+$: 881.5726 found : 881.5746.

Synthesis of alcohol 54

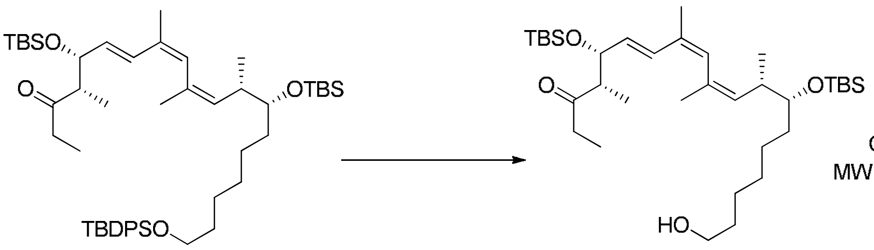

27 **54**

$C_{35}H_{68}O_4Si_2$
MW = 609,10 g/mol

TBAF stock solution : To a solution of TBAF (1 M in THF, 830 µL, 0.84 mmol, 1.00 eq) in THF (10.6 mL) at 0 °C was added AcOH (48 µL, 0.84 mmol, 1.00 eq) resulting in a 41.5 mM solution.

To the neat alcohol **27** (340 mg, 400 µmol, 1.00 eq) was added the TBAF stock solution at 0 °C (10.6 mL, 440 µmol, 1.10 eq). The reaction was stirred for 1 h at this temperature then 30 h at room temperature. The reaction was diluted with Et_2O (10 mL) and quenched with a saturated solution of $NaHCO_3$ (10 mL) at 0 °C. After separation of the organic layer, the aqueous layer was extracted with

Et_2O (3*10 mL). The organic layers were combined, dried over $MgSO_4$ and evaporated *in vacuo*. The crude product was purified by column chromatography (SiO_2, CH/EtOAc, 20:1) to give alcohol **54** as a colorless oil (180 mg, 290 µmol, 73 %).

$\boldsymbol{R_f}$ = 0.13 (SiO_2, CH/EtOAc, 10:1); $[\alpha]_D^{20}$ = - 7.5° (*c* = 0.35, $CHCl_3$); **^{1}H-NMR** (700 MHz, $CDCl_3$): δ [ppm] = δ 6.39 (d, *J* = 15.7 Hz, 1H), 5.89 (s, 1H), 5.59 (dd, *J* = 15.8, 7.4 Hz, 1H), 5.17 (dt, *J* = 9.6, 1.5 Hz, 1H), 4.41 – 4.28 (m, 1H), 3.66 (dd, *J* = 7.2, 6.1 Hz, 2H), 3.47 – 3.38 (m, 1H), 2.74 (q, *J* = 6.7 Hz, 1H), 2.60 – 2.34 (m, 3H), 1.85 (d, *J* = 1.4 Hz, 3H), 1.80 – 1.74 (m, 3H), 1.72– 1.58 (m, 2H), 1.32 (q, *J* = 12.0, 9.8 Hz, 8H), 1.13 – 1.06 (m, 3H), 1.01 (t, *J* = 7.2 Hz, 3H), 0.93 – 0.85 (m, 21H), -0.00 – -0.03 (m, 12H); **^{13}C-NMR** (176 MHz, $CDCl_3$): δ [ppm] = 213.4, 132.4, 132.3, 131.8, 130.7, 130.3, 129.5, 75.9, 75.8, 63.1, 52.9, 38.7, 36.7, 32.9, 32.8, 29.7, 26.2, 25.9, 25.8, 25.6, 24.9, 20.3, 18.1, 18.0, 15.2, 12.5, 7.5, -4.0, -4.4, -4.5, -4.9; **HRMS (ESI+)** calculated for $C_{35}H_{68}O_4Si_2Na^+$ $[M+Na]^+$: 631.4548, found : 631.4548.

Synthesis of *β*-hydroxyketone 28

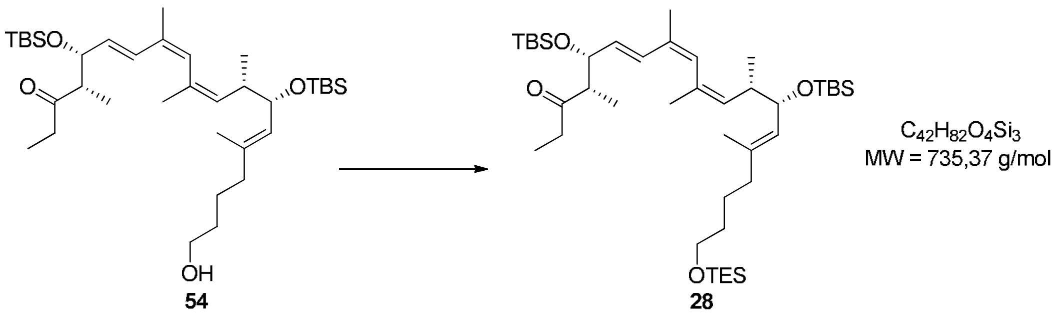

To a solution of the alcohol **52** (102 mg, 0.16 mmol, 1.00 eq) in DCM (4 mL) at -78 °C was added 2,6-lutidine (38 µL, 0.33 mmol, 2.00 eq) followed by TESOTf (56 µL, 0.25 mmol, 1.50 eq). The reaction mixture was stirred 1 h and quenched with water (4 mL) at 0 °C. After separation of the organic layer, the aqueous layer was extracted with DCM (3*5 mL). The combined organic layers were dried over $MgSO_4$ and evaporated *in vacuo*. The crude product was purified by column chromotography (SiO_2, CH/EtOAc 20:1) affording **27** (108 mg, 0.15 mmol, 90 %) as a colorless oil.

$\boldsymbol{R_f}$ = 0.59 (SiO_2, CH/EtOAc, 10:1); $[\alpha]_D^{20}$ = + 61.0° (*c* = 0.29, $CHCl_3$); **^{1}H-NMR** (700 MHz, CD_2Cl_2): δ [ppm] = 6.41 (d, *J* = 15.8 Hz, 1H), 5.92 (s, 1H), 5.59 – 5.55 (m, 1H), 5.12 – 5.07 (m, 2H), 4.32 – 4.30 (m, 1H), 4.12 (dd, *J* = 8.9, 5.9 Hz, 1H), 3.60 (t, *J* = 6.2 Hz, 4H), 2.72 – 2.68 (m, 1H), 2.53 – 2.39 (m, 2H), 2.33 (ddd, *J* = 16.9, 10.1, 5.0 Hz, 1H), 1.98 (t, *J* = 7.1 Hz, 2H), 1.83 (d, *J* = 1.1 Hz, 3H), 1.77 (s, 3H), 1.58 (s, 3H), 1.50 – 1.43 (m, 4H), 1.03 (d, *J* = 6.9 Hz, 3H), 0.96 (dt, *J* = 14.5, 5.2 Hz, 12H), 0.89 (s, 3H), 0.87 (d, *J* = 3.0 Hz, 9H), 0.85 – 0.84 (m, 9H), 0.58 (dt, *J* = 8.0, 5.3 Hz, 6H), 0.03 (s, 3H), -0.01 (s, 6H), -0.03 (s, 6H); **^{13}C-NMR** (700 MHz, CD_2Cl_2): δ [ppm] = 212.8, 135.5, 132.7, 132.1, 131.9, 130.6, 130.4, 129.7, 127.1, 76.0, 72.9, 62.6, 52.9, 40.5, 39.4, 36.4, 32.6, 25.6, 25.6, 24.5, 24.1, 20.1, 18.0, 17.9, 16.4, 15.4, 13.8, 12.1, 7.2, 6.6, 4.4, -4.3, -4.6, -5.2; **HRMS (ESI+)** calculated for $C_{42}H_{86}O_4Si_3N$ $[M+NH_4]^+$: 752.5859, found : 752.5859.

2.2 Synthesis of main fragments 39 and 40

Synthesis of aldehyde 30

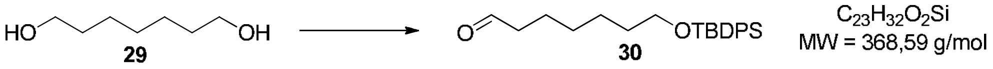

To a solution of 1,7-heptanediol (**29**) (3.70 g, 28.0 mmol, 1.00 eq) in THF (50 mL) at 0 °C, was added NaH (60 % in oil, 940 mg, 28.0 mmol, 1.00 eq) portionwise. The mixture was then stirred for 15 min at 0 °C. TBDPSCl (7.3 mL, 28.0 mmol, 1.00 eq) was added dropwise followed by TBAI (1.03 mg, 0.28 mmol, 0.10 eq). The reaction was stirred overnight at room temperature. The yellow suspension was then diluted with Et_2O (50 mL) and quenched with a saturated solution of NH_4Cl (100 mL). After separation of the organic layer, the aqueous layer was extracted with Et_2O (3*50 mL). The organic layers were combined, dried over $MgSO_4$ and evaporated *in vacuo*. The crude mixture was purified by column chromatography (SiO_2, CH/EtOAc, 4:1) to give the desired mono-protected alcohol as thick colorless oil (5.07 g, 13.7 mmol, 49 %).

DMSO (1 mL, 14.6 mmol, 3.00 eq) was added dropwise to a solution of $(COCl)_2$ (0.84 mL, 9.7 mmol, 2.00 eq) in DCM (60 mL) at -78 °C. The mixture was stirred 15 min then the mono-protected alcohol (1.80 g, 4.86 mmol, 1.00 eq) in DCM (5 mL) was added over 15 min. After 1 h, NEt_3 (2.6 mL, 19.4 mmol, 4.00 eq) was added over 10 min. The reaction was stirred for 30 min at -78 °C then warmed up at 0 °C for 30 min. The reaction was quenched with the slow addition of water (50 mL). After separation of the organic layer, the aqueous layer was extracted with DCM (3*50 mL). The combined organic layers were dried over $MgSO_4$ and evaporated *in vacuo.* Purification by column chromatography (SiO_2, CH/EtOAc, 9:1) gave aldehyde **30** (1.75 g, 0.57 mmol, 98 %).

$\boldsymbol{R}_f$ = 0.65 (SiO_2, CH/EtOAc, 1:1); **^{1}H-NMR** (500 MHz, $CDCl_3$): δ [ppm] = 9.78 (t, *J* = 1.8 Hz, 1H), 7.75 – 7.62 (m, 4H), 7.50 – 7.35 (m, 6H), 3.68 (t, *J* = 6.4 Hz, 2H), 2.42 (td, *J* = 7.4, 1.9 Hz, 2H), 1.70 – 1.52 (m, 4H), 1.47 – 1.30 (m, 4H), 1.07 (s, 9H); **^{13}C-NMR** (125 MHz, $CDCl_3$): δ [ppm] = 202.9, 135.6, 134.1, 129.5, 127.6, 63.8, 43.8, 32.3, 28.9, 26.9, 25.6, 22.1, 19.2. The spectroscopic data were in agreement with those previously reported.[4]

Synthesis of *β*-hydroxyketone 31

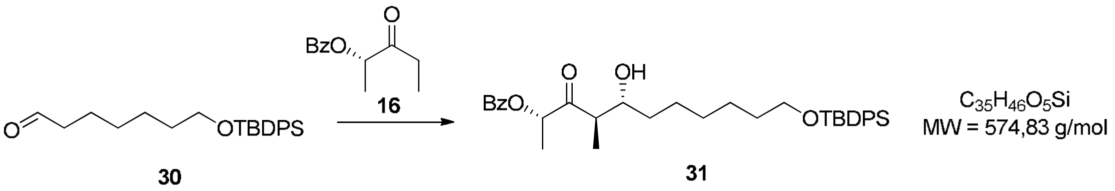

To a solution of chlorodicyclohexylborane (1 M in hexane; 8.70 mL, 8.70 mmol, 1.50 eq) in Et_2O (45 mL) at -78 °C, was added DMEA (1.26 mL, 11.6 mmol, 2.00 eq) followed by ketone **16** (1.20 g, 5.82 mmol, 1.00 eq). The reaction was stirred for 2 h at 0 °C and cooled down at -78 °C. Aldehyde **30**, stirred for 2 h over 3 Å MS, (2.63 g, 7.00 mmol, 1.20 eq) was added. The mixture was stirred for 1 h at -78 °C and then stored in the deep freezer (-20 °C) overnight. The reaction was quenched at 0 °C with MeOH (15 mL), pH 7 buffer (15 mL) and H_2O_2 (7 mL) and stirred for 1 h at room temperature. After separation of the organic phase, the aqueous phase was extracted with DCM (3*20 mL). The combined organic layers were dried over $MgSO_4$ and evaporated *in vacuo*. The crude product was

[4] G. Kumaraswamy, G. Ramakrishna, B. Sridhar, *Tetrahedron Lett.* **2011**, *52*, 1778–1782.

purified by flash column chromatography (SiO_2, CH/EtOAc, 10:1) to give the desired product **31** (1.80 g, 3.12 mmol, *dr* >20:1, 54 %) as a thick colorless oil.

***R*f** = 0.34 (SiO_2, CH/EtOAc, 4:1); $[\alpha]_D^{20}$ = + 25.2° (*c* = 0.31, $CHCl_3$); **^{1}H-NMR** (700 MHz, $CDCl_3$): δ [ppm] = 8.13 – 8.08 (m, 2H), 7.71 – 7.66 (m, 4H), 7.63 – 7.59 (m, 1H), 7.50 – 7.38 (m, 8H), 5.46 (q, *J* = 7.1 Hz, 1H), 3.77 (ddd, *J* = 9.7, 7.0, 2.5 Hz, 1H), 3.67 (t, *J* = 6.5 Hz, 2H), 2.88 (p, *J* = 7.2 Hz, 1H), 1.59 (d, *J* = 7.1 Hz, 3H), 1.57-1.55 (m, 2H), 1.52 (tq, *J* = 7.9, 2.8, 2.3 Hz, 2H), 1.42 – 1.31 (m, 6H), 1.29 (d, *J* = 7.2 Hz, 3H), 1.06 (s, 9H); **^{13}C-NMR** (176 MHz, $CDCl_3$): δ [ppm] = 212.1, 165.9, 135.6, 134.2, 133.4, 129.8, 129.5, 129.4, 128.5, 63.9, 60.4, 48.2, 34.5, 32.5, 29.3, 26.9, 25.8, 25.5, 15.9, 14.6; **HRMS (ESI+)** calculated for $C_{35}H_{46}O_5SiNa^+$ $[M+Na]^+$: 597.3307, found : 597.3007.

Synthesis of protected ketone 55

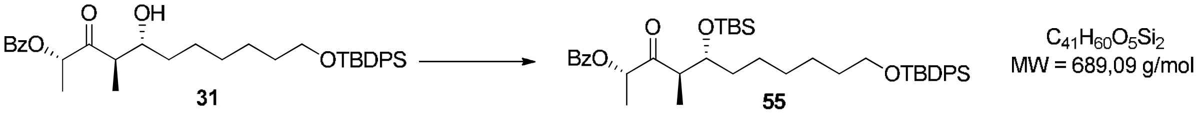

To a stirred solution of alcohol **31** (888 mg, 1.54 mmol, 1.00 eq) in DCM (50 mL) at -78 °C was added 2,6-lutidine (0.36 mL, 3.08 mmol, 2.00 eq) and TBSOTf (0.53 mL, 2.31 mmol, 1.50 eq). The reaction was stirred 1 h 30 then diluted with DCM (50 mL) and quenched with water (50 mL). After separation of the organic layer, the aqueous layer was extracted with DCM (3*50 mL). The organic layers were combined, dried over $MgSO_4$ and evaporated *in vacuo*. The crude product was purified by column chromatography (SiO_2, CH/EtOAc, 10:1) to give **55** as a thick colorless oil (996 mg, 1.33 mmol, 85 %).

***R*f** = 0.63 (SiO_2, CH/EtOAc, 5:1); $[\alpha]_D^{20}$ = - 7.6° (*c* = 0.29, $CHCl_3$); **^{1}H-NMR** (500 MHz, $CDCl_3$): δ [ppm] = 8.15 – 8.04 (m, 2H), 7.71 – 7.58 (m, 4H), 7.66 – 7.53 (m, 1H), 7.51 – 7.34 (m, 8H), 5.42 (q, *J* = 7.0 Hz, 1H), 4.03 (dt, *J* = 8.0, 3.9 Hz, 1H), 3.65 (t, *J* = 6.4 Hz, 2H), 3.03 (dq, *J* = 8.2, 7.1 Hz, 1H), 1.58-1.51 (m, 4H), 1.51 dt, *J* = 6.4 Hz, 3H), 1.38 – 1.31 (m, 2H), 1.31 – 1.21 (m, 4H), 1.09 (d, *J* = 7.0 Hz, 3H), 1.05 (s, 9H), 0.83 (s, 9H); **^{13}C-NMR** (125 MHz, $CDCl_3$): δ [ppm] = 209.3, 165.8, 135.6, 134.2, 133.2, 129.8, 129.7, 128.4, 127.6, 74.9, 72.8, 63.9, 47.1, 33.2, 32.6, 29.7, 26.9, 25.9, 22.9, 19.2, 18.0, 15.5, 13.2, -4.7; **HRMS (ESI+)** calculated for $C_{41}H_{60}O_5Si_2Na^+$ $[M+Na]^+$: 711.3871, found : 711.3871.

Synthesis of aldehyde 32

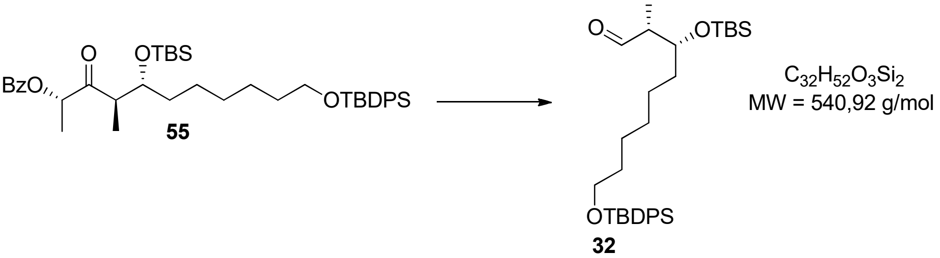

To a solution of $LiBH_4$ (340 mg, 15.7 mmol, 12.0 eq) in THF (40 mL) at -78 °C was added ketone **55** (885 mg, 1.33 mmol, 1.00 eq) in THF (10 mL). The reaction was stirred three days at room temperature. At 0 °C, water (40 mL) was added followed by careful addition of a saturated solution of NH_4Cl (5 mL). The mixture was poured into a mixture of water and Et_2O (100 mL, 1:1). After separation of the organic layer, the aqueous layer was extracted with Et_2O (3*50 mL). The organic

layers were combined, dried $MgSO_4$ and evaporated *in vacuo*. Purification by column chromatography (SiO_2, CH/EtOAc, 4:1) yielded to the diol (750 mg, 1.33 mmol, quant, *dr* = 4:1) as a colorless oil.

To a solution of diol (750 mg, 1.33 mmol, 1.00 eq) in dioxane (20 mL) and water (10 mL) at 0 °C was added $NaIO_4$ (683 mg, 3.20 mmol, 2.50 eq) portionwise. The reaction mixture was stirred vigorously overnight, diluted with DCM (20 mL) and quenched with water (20 mL). After separation of the organic layer, the aqueous layer was extracted with DCM (3*30 mL). The organic layers were combined, dried over $MgSO_4$ and evaporated *in vacuo*. After purification by column chromatography (SiO_2, CH/EtOAc, 9:1), aldehyde **32** was obtained (583 mg, 1.07 mmol, 85 %) as a colorless oil.

R_f = 0.66 (SiO_2, CH/EtOAc, 5:1); $[\alpha]_D^{20}$ = -22.6° (*c* = 0.35, $CHCl_3$); **^{1}H-NMR** (500 MHz, $CDCl_3$): δ [ppm] = 9.74 (d, *J* = 2.3 Hz, 1H), 7.70 – 7.63 (m, 4H), 7.47 – 7.33 (m, 6H), 3.91 (q, *J* = 5.5 Hz, 1H), 3.65 (t, *J* = 6.4 Hz, 2H), 2.49 (ddd, *J* = 7.1, 4.9, 2.3 Hz, 1H), 1.59 – 1.50 (m, 6H), 1.44 (ddd, *J* = 15.4, 9.5, 4.2 Hz, 1H), 1.38 – 1.23 (m, 7H), 1.07 (d, *J* = 7.0 Hz, 3H), 1.04 (s, 9H), 0.88 (s, 9H), 0.06 (d, *J* = 4.0 Hz, 6H); **^{13}C-NMR** (125 MHz, $CDCl_3$): δ [ppm] = 205.2, 135.6, 134.2, 129.5, 127.6, 73.5, 63.9, 51.1, 34.8, 32.5, 29.5, 26.9, 25.8, 24.8, 19.2, 18.1, 10.5, -4.2, -4.7; **HRMS (ESI+)** calculated for $C_{34}H_{56}O_3Si_2K^+$ $[M+K]^+$: 579.3087, found : 579.3090.

Synthesis of ester 33

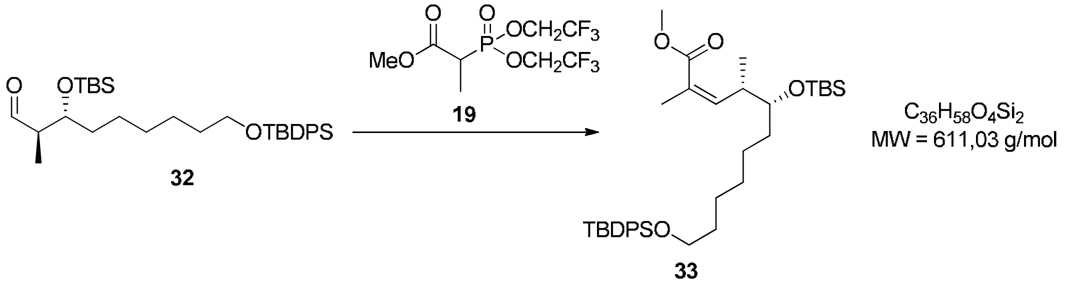

To a solution of 18-c-6 (674 mg, 2.55 mmol, 2.30 eq) and methyl 2-(bis(2,2,2-trifluoroethoxy)phosphoryl)propanoate **19** (516 mg, 1.55 mmol, 1.40 eq) in THF (20 mL) at -78 °C was added KHMDS (0.5 M in toluene, 2.9 mL, 1.44 mmol, 1.30 eq). The reaction was stirred of 30 min. Aldehyde **32** (594 mg, 1.11 mmol, 1.00 eq) in THF (2 mL) was added dropwise and the reaction was stirred for another 2 h at -78 °C. The reaction was quenched with a saturated solution of $NaHCO_3$ (30 mL) at 0 °C. After separation of the organic layer, the aqueous layer was extracted with DCM (3*30 mL). The organic layers were combined, dried over $MgSO_4$ and evaporated *in vacuo*. After column chromatography (SiO_2, CH/EtOAc, 9:1), ester **33** was obtained (610 mg, 1.00 mmol, 91 %) as a colorless oil.

R_f = 0.66 (SiO_2, CH/EtOAc, 5:1); $[\alpha]_D^{20}$ = + 5.2° (*c* = 0.33, $CHCl_3$); **^{1}H-NMR** (700 MHz, $CDCl_3$): δ [ppm] = 7.69 – 7.68 (m, 4H), 7.45 – 7.42 (m, 2H), 7.41 – 7.38 (m, 4H), 5.94 (dq, *J* = 10.1, 1.4 Hz, 1H), 3.73 (s, 3H), 3.66 (t, *J* = 6.5 Hz, 2H), 3.55 (td, *J* = 6.1, 3.6 Hz, 1H), 3.30 (dqd, *J* = 10.4, 6.8, 3.5 Hz, 1H), 1.93 (d, *J* = 1.4 Hz, 3H), 1.40 – 1.18 (m, 10H), 1.06 (s, 9H), 1.00 (d, *J* = 6.8 Hz, 3H), 0.92 (s, 9H), 0.07 (s, 3H), 0.06 (s, 3H); **^{13}C-NMR** (176 MHz, $CDCl_3$): δ [ppm] = 168.5, 144.8, 135.6, 134.2, 129.5, 127.6, 126.6, 75.7, 64.0, 51.2, 38.0, 35.1, 32.6, 29.6, 26.9, 26.0, 25.8, 25.5, 21.1, 19.2, 18.2, 17.0, -4.2, -4.5; **HRMS (ESI+)** calculated for $C_{36}H_{58}O_4Si_2Na^+$ $[M+Na]^+$: 633.3766, found : 633.3763.

Synthesis of aldehyde 34

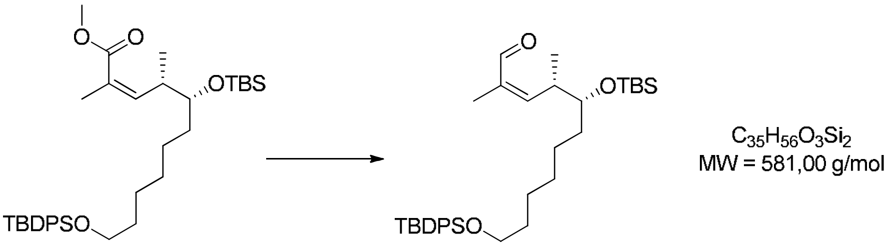

33 **34**

To a solution of ester **33** (620 mg, 1.01 mmol, 1.00 eq) in DCM (15 mL) at -78 °C was added DIBALH (1 M in hexane, 3.0 mL, 3.04 mmol, 3.00 eq) dropwise. The mixture was stirred for 1 h and warmed up to 0 °C. The reaction was diluted with DCM (15 mL), H_2O (0.12 mL) was added followed by 3 M NaOH aqueous solution (0.12 mL) and H_2O (0.3 mL). After stirring 15 min at room temperature, $MgSO_4$ was added and the mixture was stirred an additionnal 15 min. After filtration, the solvents were removed *in vacuo*. The crude product was directly use in the next step.

DMP (517 mg, 1.21 mmol, 1.20 eq) was added to a solution of crude alcohol in DCM (10 mL) at 0 °C. The mixture was stirred for 1 h at room temperature then diluted with DCM (10 mL) and quenched with saturated solution of $NaHCO_3/Na_2S_2O_3$ (30 mL, 2:1). After separation of the organic layer, the aqueous layer was extracted with DCM (3*20 mL). The combined organic layers were dried over $MgSO_4$ and evaporated *in vacuo*. Purification by column chromatography (SiO_2, CH/EtOAc, 9:1) yielded to the aldehyde **34** (525 mg, 0.96 mmol, 90 % over 2 steps) as a colorless oil.

$\boldsymbol{R}_f$ = 0.52 (SiO_2, CH/EtOAc, 20:1); $[\alpha]_D^{20}$ = + 8.8° (*c* = 0.26, $CHCl_3$); **^{1}H-NMR** (700 MHz, $CDCl_3$): δ [ppm] = 10.08 (d, *J* = 0.5 Hz, 1H), 7.68 – 7.65 (m, 4H), 7.43 – 7.40 (m, 2H), 7.39 – 7.36 (m, 4H), 6.45 (dq, *J* = 10.8, 1.3 Hz, 1H), 3.64 (t, *J* = 6.4 Hz, 2H), 3.55 (td, *J* = 5.7, 4.6 Hz, 1H), 3.33 – 3.27 (m, 1H), 1.79 (d, *J* = 1.3 Hz, 3H), 1.56 – 1.53 (m, 4H), 1.46 (ddt, *J* = 13.7, 10.4, 5.0 Hz, 1H), 1.40 – 1.31 (m, 3H), 1.30 – 1.21 (m, 4H), 1.06 (d, *J* = 6.8 Hz, 3H), 1.04 (s, 9H), 0.88 (s, 9H); **^{13}C-NMR** (176 MHz, $CDCl_3$): δ [ppm] =191.6, 152.1, 135.6, 134.2, 129.5, 127.6, 75.6, 63.9, 35.6, 34.9, 32.5, 29.6, 26.9, 25.9, 25.8, 24.8, 19.2, 18.6, 18.1, 16.7, -4.2, -4.4; **HRMS (ESI+)** calculated for $C_{35}H_{56}O_3Si_2Na^+$ $[M+Na]^+$: 603.3660, found : 603.3663.

Synthesis of ester 35

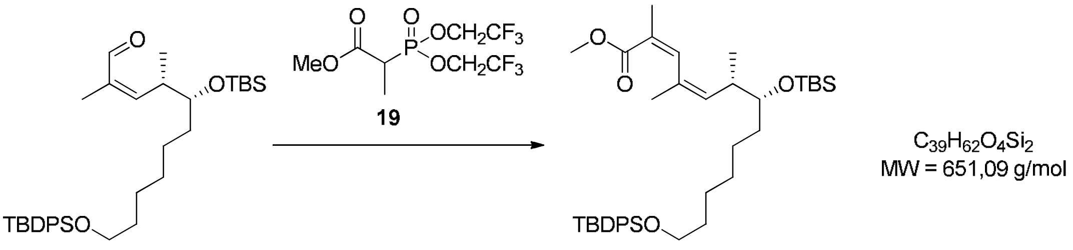

34 **35**

To a solution of 18-c-6 (536 mg, 2.05 mmol, 2.30 eq) and methyl 2-(bis(2,2,2-trifluoroethoxy)phosphoryl)propanoate **19** (416 mg, 1.25 mmol, 1.40 eq) in THF (20 mL) at -78 °C was added KHMDS (0.5 M in toluene, 2.3 mL, 1.16 mmol, 1.30 eq). The reaction was stirred of 30 min and aldehyde **34** (520 mg, 0.96 mmol, 1.00 eq) in THF (2 mL) was added dropwise. The reaction was

stirred for 4 h at -78 °C. The reaction was quenched with a saturated solution of $NaHCO_3$ (20 mL) at 0 °C. After separation of the organic layer, the aqueous layer was extracted with DCM (3*20 mL). The organic layers were combined, dried over $MgSO_4$ and evaporated *in vacuo*. After column chromatography (SiO_2, CH/EtOAc, 9:1), ester **35** was obtained (510 mg, 0.78 mmol, 87 %) as a colorless oil.

R_f = 0.55 (SiO_2, CH/EtOAc, 20:1); $[\alpha]_D^{20}$ = + 0.9 ° (*c* = 0.22, $CHCl_3$); **^{1}H-NMR** (700 MHz, $CDCl_3$): δ [ppm] = 7.67 (dt, *J* = 6.7, 1.5 Hz, 4H), 7.43 – 7.36 (m, 6H), 6.38 – 6.36 (m, 1H), 5.16 (dp, *J* = 9.9, 1.6 Hz, 1H), 3.70 (s, 3H), 3.65 (t, *J* = 6.5 Hz, 2H), 3.44 (dt, *J* = 7.0, 4.3 Hz, 1H), 2.44 (dqd, *J* = 13.7, 6.8, 3.9 Hz, 1H), 1.97 (d, *J* = 1.6 Hz, 3H), 1.79 – 1.77 (m, 3H), 1.57 – 1.54 (m, 2H), 1.35 – 1.29 (m, 4H), 1.28 – 1.19 (m, 3H), 1.17 – 1.12 (m, 1H), 1.04 (s, 9H), 0.90 – 0.88 (m, 12H), 0.01 (d, J = 3.0 Hz, 6H); **^{13}C-NMR** (176 MHz, $CDCl_3$): δ [ppm] = 169.4, 136.1, 134.2, 131.9, 129.5, 128.4, 127.6, 75.8, 64.0, 51.4, 35.8, 33.6, 32.6, 29.7, 26.9, 26.0, 25.9, 22.5, 21.1, 19.2, 18.1, 15.9, -4.3, -4.5; **HRMS (ESI+)** calculated for $C_{39}H_{62}O_4Si_2Na^+$ $[M+Na]^+$: 637.4079, found : 673.4079.

Synthesis of aldehyde 36

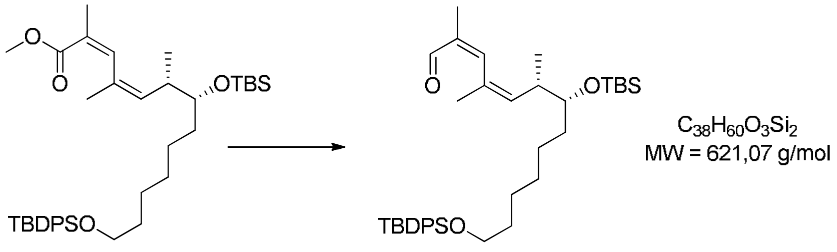

35 **36**

To a solution of ester **35** (507 mg, 0.78 mmol, 1.00 eq) in DCM (12 mL) at -78 °C was added DIBALH (1 M in hexane, 2.33 mL, 2.33 mmol, 3.00 eq) dropwise. The mixture was stirred for 1 h and warmed up to 0 °C. The reaction was diluted with DCM (12 mL), H_2O (0.09 mL) was added followed by 3 M NaOH (0.09 mL) and H_2O (0.23 mL). After stirring 15 min at room temperature, $MgSO_4$ was added and the mixture was stirred an additionnal 15 min. After filtration, the solvents were removed *in vacuo*. The crude product was directly use in the next step.

DMP (396 mg, 0.93 mmol, 1.20 eq) was added to a solution of crude alcohol in DCM (10 mL) at 0 °C. The mixture was stirred for 1 h at room temperature then diluted with DCM and quenched with saturated solutions of $NaHCO_3/Na_2S_2O_3$ (15 mL, 2:1). After separation of the organic layer, the aqueous layer was extracted with DCM (3*15 mL). The combined organic layers were dried over $MgSO_4$ and evaporated *in vacuo*. Purification by column chromatography (SiO_2, CH/EtOAc, 9:1) yielded to the aldehyde **36** (416 mg, 0.67 mmol, 86 % over 2 steps) as a colorless oil.

R_f = 0.52 (SiO_2, CH/EtOAc, 20:1); $[\alpha]_D^{20}$ = + 6.2° (*c* = 0.26, $CHCl_3$); **^{1}H-NMR** (700 MHz, $CDCl_3$): δ [ppm] = 9.89 (s, 1H), 7.68 – 7.65 (m, 4H), 7.42 – 7.36 (m, 6H), 6.94 (dd, *J* = 2.5, 1.3 Hz, 1H), 5.44 (dt, *J* = 10.3, 1.5 Hz, 1H), 3.65 (t, *J* = 6.5 Hz, 2H), 3.46 – 3.38 (m, 1H), 2.40 (ddd, *J* = 10.5, 6.9, 3.9 Hz, 1H), 1.90 (dd, *J* = 1.4, 0.8 Hz, 3H), 1.83 (d, *J* = 1.5 Hz, 3H), 1.38 – 1.20 (m, 9H), 1.04 (s, 9H), 0.91 (d, *J* = 6.8 Hz, 3H), 0.88 (s, 9H), -0.00 (d, *J* = 7.1 Hz, 6H); **^{13}C-NMR** (176 MHz, $CDCl_3$): δ [ppm] = 193.1, 146.8, 136.5, 135.6, 135.3, 134.2, 129.8, 129.5, 127.6, 75.8, 64.0, 38.5, 33.9, 32.6, 29.6, 25.9, 25.6, 25.1, 19.2, 18.1, 16.2, 15.8, -4.3, -4.5; **HRMS (ESI+)** calculated for $C_{38}H_{60}O_3Si_2Na^+$ $[M+Na]^+$: 643.3973, found : 643.3973.

Synthesis of ester 37

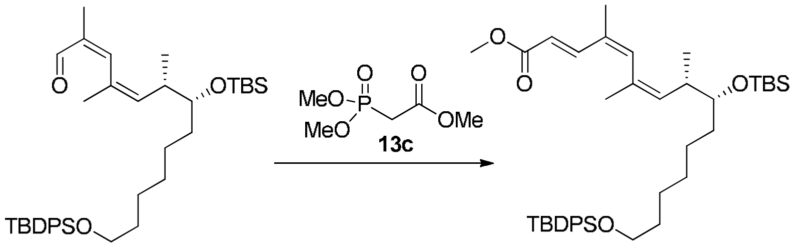

36 **37**

$C_{41}H_{64}O_4Si_2$
MW = 677,13 g/mol

To a solution of trimethyl phosphonoacetate (**13c**) (0.16 mL, 1.00 mmol, 1.50 eq) and DMPU (0.12 mL, 1.00 mmol, 1.50 eq) in THF (15 mL) at 0 °C was added *n*-BuLi (1.6 M in hexane, 0.58 mL, 0.94 mmol, 1.40 eq). The mixture was stirred for 30 min and aldehyde **36** (416 mg, 0.67 mmol, 1.00 eq) in THF (1.5 mL) was added dropwise. After stirring for 2 h at 0 °C, the reaction was stirred overnight at room temperature. The reaction was quenched with buffer ph7 (15 mL) at 0 °C. After separation of the organic layer, the aqueous layer was extracted with EtOAc (3*20 mL). The organic layers were combined, dried over $MgSO_4$ and evaporated *in vacuo*. Purification by column chromatography (SiO_2, CH/EtOAc, 9:1) yielded to ester **37** (416 mg, 0.67 mmol, 89 %) as a colorless oil.

***R*$_f$** = 0.52 (SiO_2, CH/EtOAc, 20:1); $[\alpha]_D^{20}$ = + 40.4° (*c* = 0.26, $CHCl_3$); **^{1}H-NMR** (700 MHz, $CDCl_3$): δ [ppm] = 7.68 – 7.65 (m, 4H), 7.61 (dd, *J* = 15.8, 0.7 Hz, 1H), 7.43 – 7.36 (m, 6H), 6.15 (d, *J* = 1.9 Hz, 1H), 5.87 (dd, *J* = 15.8, 0.7 Hz, 1H), 5.29 (dt, *J* = 10.3, 1.4 Hz, 1H), 3.74 (s, 3H), 3.64 (t, *J* = 6.5 Hz, 2H), 3.39 (ddd, *J* = 6.9, 4.8, 3.5 Hz, 1H), 2.32 (ddd, *J* = 10.4, 6.9, 3.7 Hz, 1H), 1.89 (d, *J* = 1.4 Hz, 3H), 1.81 (dd, *J* = 1.4, 0.7 Hz, 3H), 1.59 – 1.54 (m, 2H), 1.38 – 1.18 (m, 9H), 1.04 (d, *J* = 1.7 Hz, 9H), 0.92 (d, *J* = 6.8 Hz, 3H), 0.87 (s, 9H), -0.02 (s, 3H), -0.03 (s, 3H); **^{13}C-NMR** (176 MHz, $CDCl_3$): δ [ppm] = 167.7, 142.2, 138.0, 135.6, 134.2, 133.3, 131.6, 129.5, 127.6, 118.0, 75.9, 64.0, 51.5, 38.5, 33.5, 32.6, 29.6, 26.9, 26.0, 25.9, 24.6, 19.6, 19.2, 18.1, 15.5, -4.4, -4.6; **HRMS (ESI+)** calculated for $C_{41}H_{64}O_4Si_2Na^+$ $[M+Na]^+$: 699.4235, found : 699.4235.

Synthesis of aldehyde 38

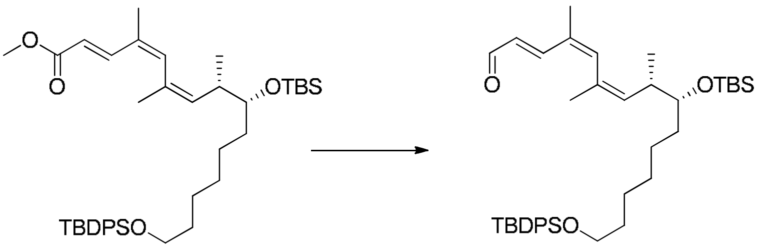

37 **38**

$C_{40}H_{62}O_3Si_2$
MW = 647,10 g/mol

To a solution of ester **37** (120 mg, 0.18 mmol, 1.00 eq) in DCM (10 mL) at -78 °C was added DIBALH (1 M in hexane, 0.53 mL, 0.53 mmol, 3.00 eq) dropwise. The mixture was stirred for 1 h and warmed up to 0 °C. The reaction was diluted with DCM (10 mL), H_2O (0.09 mL) was added followed by aqueous solution of 3 M NaOH aqueous solution (0.09 mL) and H_2O (0.23 mL). After stirring 15 min at room temperature, $MgSO_4$ was added and the mixture was stirred an additionnal 15 min.

After filtration, the solvents were removed *in vacuo*. The crude product was directly use in the next step.

DMP (90 mg, 0.21 mmol, 1.20 eq) was added to a solution of crude alcohol in DCM (4 mL) at 0 °C. The mixture was stirred for 1 h at room temperature then diluted with DCM (4 mL) and quenched with saturated solutions of $NaHCO_3/Na_2S_2O_3$ (12 mL, 2:1). After separation of the organic layer, the aqueous layer was extracted with DCM (3*10 mL). The combined organic layers were dried over $MgSO_4$ and evaporated *in vacuo*. Purification by column chromatography (SiO_2, CH/EtOAc, 20:1) yielded to the aldehyde **38** (105 mg, 0.16 mmol, 90 % over 2 steps) as a yellow oil.

***R*f** = 0.52 (SiO_2, CH/EtOAc, 20:1); $[\alpha]_D^{20}$ = + 3.3° (*c* = 0.24, $CHCl_3$); **^{1}H-NMR** (700 MHz, $CDCl_3$): δ [ppm] = 9.61 (dd, *J* = 7.8, 5.5 Hz, 1H), 7.69 – 7.64 (m, 4H), 7.50 – 7.43 (m, 1H), 7.42 – 7.34 (m, 6H), 6.27 (s, 1H), 6.16 (dd, *J* = 15.7, 7.8 Hz, 1H), 5.35 (dt, *J* = 10.2, 1.5 Hz, 1H), 3.64 (t, *J* = 6.4 Hz, 2H), 3.40 (dd, *J* = 6.4, 4.0 Hz, 1H), 2.34 (ddd, *J* = 10.5, 6.9, 3.8 Hz, 1H), 1.94 (d, *J* = 1.4 Hz, 2H), 1.87 – 1.81 (m, 2H), 1.26 (dt, *J* = 21.0, 11.2 Hz, 8H), 1.04 (s, 9H), 0.91 (d, *J* = 6.8 Hz, 3H), 0.88 (d, *J* = 2.7 Hz, 9H), 0.04 – -0.06 (m, 6H); **^{13}C-NMR** (176 MHz, $CDCl_3$): δ [ppm] = 194.2, 150.7, 139.9, 135.6, 134.2, 134.0, 132.0, 131.5, 129.5, 129.1, 127.6, 75.9, 64.0, 38.5, 33.7, 32.6, 29.6, 26.9, 25.9, 24.5, 19.6, 19.2, 18.1, 15.8, -4.3, -4.6; **HRMS (ESI+)** calculated for $C_{40}H_{62}O_3Si_2Na^+$ $[M+Na]^+$: 669.4130, found : 669.4130.

Synthesis of *β*-hydroxyketone 56

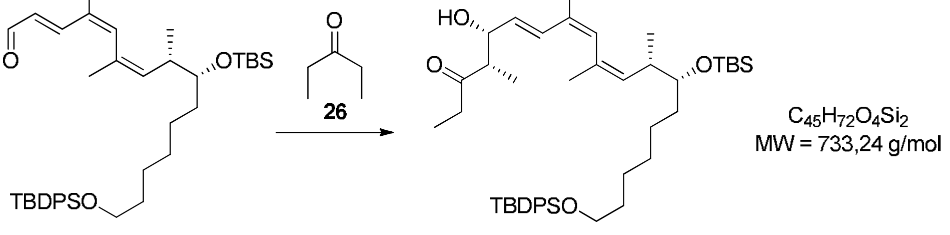

Stock solution: (-)-Ipc_2BH (545 mg, 1.93 mmol, 1.00 eq) was dissolved in anhydrous hexane (0.44 mL) and cooled down at 0°C. Triflic acid (167 µL, 1.93 mmol, 1.00 eq) was added and the mixture was stirred at room temperature until no Ipc_2BH cristals were seen.

The triflate solution (0.22 mL, 0.76 mmol, 1.30 eq) was diluted with anhydrous DCM (4 mL) and cooled down to -78 °C. DIEA (145 µL, 0.83 mmol, 3.00 eq) was added dropwise followed by diethylketone (**26**) (41 µL, 0.39 mmol, 1.40 eq). The reaction mixture was stirred for 3 h at this temperature. Aldehyde **38** (180 mg, 0.28 mmol, 1.00 eq) in DCM (2 mL) was added, the reaction was stirred for 1 h at -78 °C and stored in the freezer (-20 °C) overnight. Buffer pH7 (2 mL), MeOH (2 mL) and H_2O_2 (0.8 mL) were added and stirred for 1 h at room temperature. After separation of the organic layer, the aqueous layer was extracted with DCM (3*5 mL). The organic layers were combined, dried over $MgSO_4$ and evaporated *in vacuo*. The crude was purified by column chromatograpy (SiO_2, CH/EtOAc, 30:1) to give the starting material back (25 mg, 38 µmol, 14 %) and the desired product **56** as a colorless oil (132 mg, 0.18 mmol, 64 %, 78 % brsm., *dr* = 10.1).

***R*f** = 0.36 (SiO_2, CH/EtOAc, 10:1); $[\alpha]_D^{20}$ = + 2.2° (*c* = 0.12, $CHCl_3$); **^{1}H-NMR** (700 MHz, $CDCl_3$): δ [ppm] = 7.68 – 7.65 (m, 4H), 7.43 – 7.40 (m, 2H), 7.39 – 7.36 (m, 4H), 6.52 (dt, *J* = 15.9, 0.9 Hz, 1H), 5.82 (s, 1H), 5.64 (ddd, *J* = 15.8, 6.8, 0.7 Hz, 1H), 5.16 (dp, *J* = 9.9, 1.4 Hz, 1H), 4.47 (dtd, *J* = 6.9, 3.5, 1.3 Hz, 1H), 3.65 (t, *J* = 6.5 Hz, 2H), 3.39 (dd, *J* = 7.0, 3.9 Hz, 1H), 2.69 (tdd, *J* = 7.3, 5.5, 3.0 Hz, 1H), 2.66 – 2.64

(m, 1H), 2.57 – 2.46 (m, 2H), 2.37 (dqd, *J* = 10.4, 6.8, 3.8 Hz, 1H), 1.84 (d, *J* = 1.4 Hz, 3H), 1.77 – 1.75 (m, 3H), 1.35 – 1.28 (m, 5H), 1.24 – 1.19 (m, 2H), 1.14 – 1.12 (m, 3H), 1.06 – 1.03 (m, 13H), 0.90 (d, *J* = 6.8 Hz, 3H), 0.87 (s, 9H), -0.00 – -0.03 (m, 6H); **^{13}C-NMR** (176 MHz, $CDCl_3$): δ [ppm] = 215.7, 135.6, 134.2, 132.3, 132.0, 131.6, 130.8, 130.4, 129.5, 129.1, 127.6, 76.0, 73.1, 64.0, 50.7, 38.5, 35.4, 33.1, 32.6, 29.6, 26.9, 26.2, 25.9, 24.6, 20.1, 19.2, 18.1, 15.2, 10.9, 7.6, -4.4., -4.6; **HRMS (ESI+)** calculated for $C_{45}H_{72}O_4Si_2Na^+$ $[M+Na]^+$: 755.4861, found : 755.4879.

Synthesis of main fragment 39

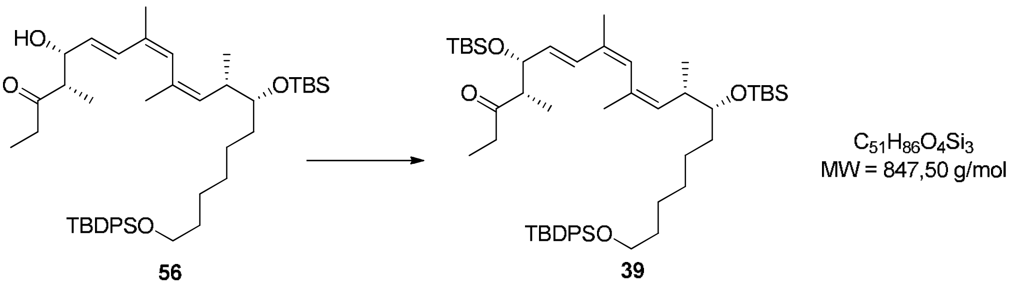

To a stirred solution of alcohol **56** (145 mg, 0.20 mmol, 1.00 eq) in DCM (3 mL) at -78 °C was added 2,6-lutidine (46 µL, 0.40 mmol, 2.00 eq) and TBSOTf (68 µL, 0.30 mmol, 1.50 eq). The reaction was stirred 1 h 30 at -78 °C. The reaction was diluted with DCM (5 mL) at 0 °C and quenched with water (5 mL). After separation of the organic layer, the aqueous layer was extracted with DCM (3*5 mL). The organic layers were combined, dried over $MgSO_4$ and evaporated *in vacuo*. The crude product was purified by column chromatography (SiO_2, CH/EtOAc, 20:1) to give product **39** as a thick colorless oil (153 mg, 0.18 mmol, 90 %).

$\boldsymbol{R}_f$ = 0.54 (SiO_2, CH/EtOAc, 10:1); $[\boldsymbol{\alpha}]_{\boldsymbol{D}}^{\mathbf{20}}$ = + 23.0° (*c* = 0.31, $CHCl_3$); **^{1}H-NMR** (700 MHz, $CDCl_3$): δ [ppm] = 7.73 – 7.67 (m, 4H), 7.51 – 7.36 (m, 6H), 6.39 (d, *J* = 15.7 Hz, 1H), 5.89 (s, 1H), 5.59 (dd, *J* = 15.8, 7.4 Hz, 1H), 5.17 (d, *J* = 9.6 Hz, 1H), 4.34 (t, *J* = 6.8 Hz, 1H), 3.67 (t, *J* = 6.5 Hz, 2H), 3.42 (s, 1H), 2.72 (p, *J* = 6.8 Hz, 1H), 2.49 (dq, *J* = 10.4, 7.2 Hz, 3H), 1.84 (d, *J* = 1.4 Hz, 3H), 1.58 (d, *J* = 7.4 Hz, 2H), 1.40 – 1.23 (m, 8H), 1.10 – 1.05 (m, 12H), 1.01 (t, *J* = 7.2 Hz, 4H), 0.89 (dd, *J* = 2.8, 1.3 Hz, 21H), -0.00 – -0.03 (m, 12H); **^{13}C-NMR** (176 MHz, $CDCl_3$): δ [ppm] = 213.3, 135.6, 134.2, 131.8, 130.7, 130.3, 129.6, 129.5, 127.6, 75.9, 75.8, 64.0, 53.0, 38.7, 36.6, 33.0, 32.6, 29.7, 26.9, 26.3, 26.0, 25.9, 24.6, 20.2, 19.2, 18.1, 15.3, 12.5, 7.2, -4.0, -4.4, -4.5, -4.9; **HRMS (ESI+)** calculated for $C_{51}H_{86}O_4Si_3Na^+$ $[M+Na]^+$: 869.5726, found : 869.5727.

Synthesis of alcohol 57

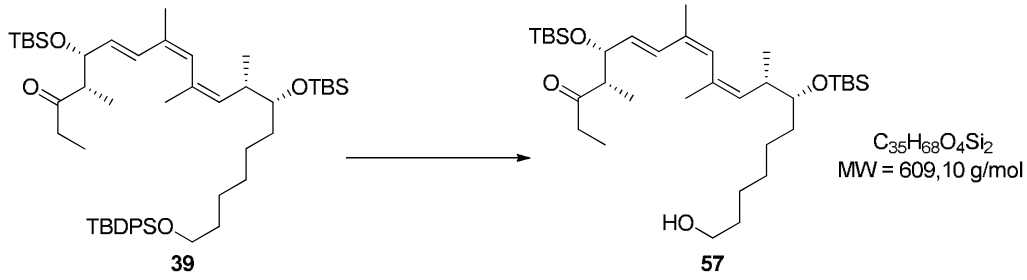

TBAF stock solution : To a solution of TBAF (1 M in THF, 830 µL, 0.84 mmol, 1.00 eq) in THF (10.6 mL) at 0 °C was added AcOH (48 µL, 0.84 mmol, 1.00 eq) resulting in a 41.5 mM solution.

To the neat alcohol **39** (340 mg, 400 µmol, 1.00 eq) was added the TBAF stock solution at 0 °C (10.6 mL, 440 µmol, 1.10 eq). The reaction was stirred for 1 h at this temperature then 30 h at room temperature. The reaction was diluted with Et_2O (10 mL) and quenched with a saturated solution of $NaHCO_3$ (10 mL) at 0 °C. After separation of the organic layer, the aqueous layer was extracted with Et_2O (3*10 mL). The organic layers were combined, dried over $MgSO_4$ and evaporated *in vacuo*. The crude product was purified by column chromatography (SiO_2, CH/EtOAc, 20:1) to give alcohol **57** as a colorless oil (180 mg, 290 µmol, 73 %).

$\boldsymbol{R}_f$ = 0.13 (SiO_2, CH/EtOAc, 10:1); $[\boldsymbol{\alpha}]_{\boldsymbol{D}}^{\boldsymbol{20}}$ = - 7.5° (*c* = 0.35, $CHCl_3$); **^{1}H-NMR** (700 MHz, $CDCl_3$): δ [ppm] = δ 6.39 (d, *J* = 15.7 Hz, 1H), 5.89 (s, 1H), 5.59 (dd, *J* = 15.8, 7.4 Hz, 1H), 5.17 (dt, *J* = 9.6, 1.5 Hz, 1H), 4.41 – 4.28 (m, 1H), 3.66 (dd, *J* = 7.2, 6.1 Hz, 2H), 3.47 – 3.38 (m, 1H), 2.74 (q, *J* = 6.7 Hz, 1H), 2.60 – 2.34 (m, 3H), 1.85 (d, *J* = 1.4 Hz, 3H), 1.80 – 1.74 (m, 3H), 1.72– 1.58 (m, 2H), 1.32 (q, *J* = 12.0, 9.8 Hz, 8H), 1.13 – 1.06 (m, 3H), 1.01 (t, *J* = 7.2 Hz, 3H), 0.93 – 0.85 (m, 21H), -0.00 – -0.03 (m, 12H); **^{13}C-NMR** (176 MHz, $CDCl_3$): δ [ppm] = 213.4, 132.4, 132.3, 131.8, 130.7, 130.3, 129.5, 75.9, 75.8, 63.1, 52.9, 38.7, 36.7, 32.9, 32.8, 29.7, 26.2, 25.9, 25.8, 25.6, 24.9, 20.3, 18.1, 18.0, 15.2, 12.5, 7.5, -4.0, -4.4, -4.5, -4.9; **HRMS (ESI+)** calculated for $C_{35}H_{68}O_4Si_2Na^+$ $[M+Na]^+$: 631.4548, found : 631.4548.

Synthesis of main fragment 40

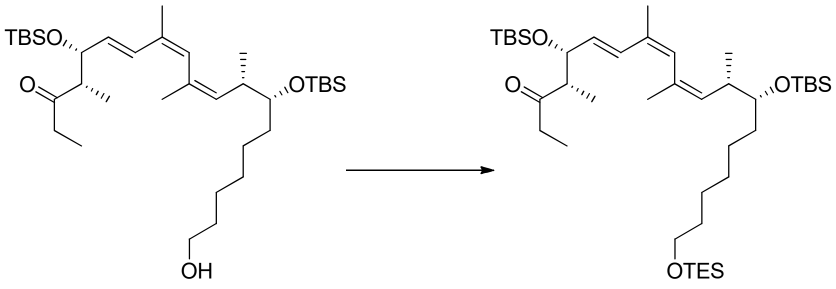

57 **40**

$C_{41}H_{82}O_4Si_3$
MW = 723,36 g/mol

To a solution of alcohol **57** (127 mg, 0.32 mmol, 1.00 eq) in DCM (4 mL) at -78 °C was added 2,6-lutidine (48 µL, 0.42 mmol, 2.00 eq) followed by TESOTf (78 µL, 0.31 mmol, 1.50 eq). The reaction mixture was stirred for 1 h and quenched with water (4 mL) at 0 °C. After separation of the organic layer, the aqueous phase was extracted with DCM (3*5 mL). The combined organic layers were dried over $MgSO_4$ and evaporated *in vacuo*. The crude product was purified by column chromotography (SiO_2, CH/EtOAc 20:1) affording **40** (140 mg, 0.19 mmol, 90 %) as a colorless oil.

$\boldsymbol{R}_f$ = 0.52 (SiO_2, CH/EtOAc, 10:1); $[\boldsymbol{\alpha}]_{\boldsymbol{D}}^{\boldsymbol{20}}$ = + 26.1° (*c* = 0.62, $CHCl_3$); **^{1}H-NMR** (700 MHz, CD_2Cl_2): δ [ppm] = 6.39 (dt, *J* = 15.7, 0.9 Hz, 1H), 5.89 (d, *J* = 1.7 Hz, 1H), 5.60 – 5.56 (m, 1H), 5.19 – 5.16 (m, 1H), 4.33 (ddd, *J* = 7.2, 5.9, 1.0 Hz, 1H), 3.58 (td, *J* = 6.7, 1.8 Hz, 2H), 3.43 (td, *J* = 6.5, 6.0, 3.8 Hz, 1H), 2.69 (qd, *J* = 6.9, 5.7 Hz, 1H), 2.54 – 2.37 (m, 3H), 1.84 – 1.81 (m, 2H), 1.78 – 1.74 (m, 3H), 1.51 – 1.47 (m, 2H), 1.36 – 1.15 (m, 8H), 1.04 (d, *J* = 6.9 Hz, 3H), 0.95 (td, *J* = 7.6, 4.5 Hz, 12H), 0.89 – 0.88 (m, 3H), 0.88 (d, *J* = 2.7 Hz, 9H), 0.87 (s, 9H), 0.59 (q, *J* = 8.0 Hz, 6H), 0.05 (s, 3H), -0.01 (s, 6H), -0.04 (s, 3H); **^{13}C-NMR** (700 MHz, CD_2Cl_2): δ [ppm] = 212.6, 132.4, 132.4, 131.7, 130.8, 130.2, 129.4, 75.9, 75.8, 62.8, 53.8, 53.7, 53.6, 53.6, 53.5, 53.4, 53.3, 53.3, 53.1, 52.9, 38.6, 36.4, 33.1, 33.0, 29.7, 26.2, 25.9, 25.7, 25.7, 25.6, 25.6, 25.6, 24.6, 19.9, 18.0, 17.9, 15.1, 12.1, 7.2, 6.5, 4.4, -4.3, -4.7, -4.8, -5.2; **HRMS (ESI+)** calculated for $C_{41}H_{82}O_4Si_3Na^+$ $[M+Na]^+$: 745.5413, found : 745.5410.

2.3 Coupling by an aldol-condensation sequence

Synthesis of mono-protected alcohol 58

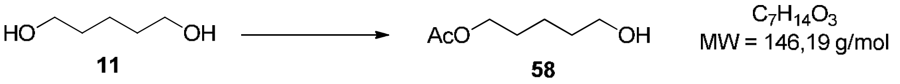

To a solution of 1,5-pentanediol (**11**) (600 mg, 5.76 mmol, 1.00 eq) in DCM (6 mL) at 0 °C was added pyridine (0.62 mL, 7.49 mmol, 1.30 eq), DMAP (84 mg, 0.70 mmol, 0.12 eq) and Ac_2O (0.54 mL, 5.76 mmol, 1.00 eq). The reaction mixture was stirred for 1 h at 0 °C before being diluted with DCM (10 mL) and quenched with H_2O (10 mL). After separation of the organic layer, the aqueous layer was extracted with DCM (3*10 mL). The organic layers were combined and washed with an aqueous solution of 1 M HCl (10 mL). The organic layer was dried over $MgSO_4$ and evaporated *in vacuo*. The crude product was purified by column chromatography (SiO_2, CH/EtOAc, 2:1) to give the monoprotected alcohol **58** (326 mg, 2.23 mmol, 39 %).

***R*f** = 0.40 (SiO_2, CH/EtOAc, 1:1); **^{1}H-NMR** (500 MHz, $CDCl_3$): δ [ppm] = 4.07 (t, *J* = 6.6 Hz, 2H), 3.66 (t, *J* = 6.5 Hz, 2H), 2.04 (s, 3H), 1.69 – 1.57 (m, 4H), 1.47 – 1.40 (m, 2H); **^{13}C-NMR** (125 MHz, $CDCl_3$): δ [ppm] = 171.6, 64.8, 63.1, 32.7, 28.8, 22.6, 21.4; **HRMS (ESI+)** calculated for $C_7H_{15}O_3^+$ $[M+H]^+$: 147.1016 found : 147.1016.

Synthesis of aldehyde 41

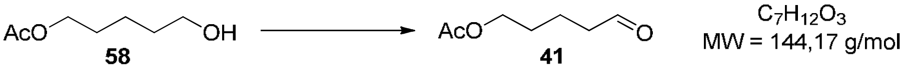

DMSO (0.45 mL, 6.36 mmol, 3.00 eq) was added dropwise to a solution of $(COCl)_2$ (0.36 mL, 4.2 mmol, 2.00 eq) in DCM (10 mL) at -78 °C. The mixture was stirred 15 min then alcohol **58** (310 mg, 2.12 mmol, 1.00 eq) in DCM (1 mL) was added dropwise. After 30 min, NEt_3 (1.15 mL, 8.48 mmol, 4.00 eq) was added dropwise. The reaction was stirred for 20 min at -78 °C then warmed up to 0 °C for 30 min. The reaction was quenched with the addition of water (10 mL). After separation of the organic layer, the aqueous layer was extracted with DCM (3*10 mL). The combined organic layers were dried over $MgSO_4$ and evaporated *in vacuo.* Purification by column chromatography (SiO_2, CH/EtOAc, 5:1) gave aldehyde **41** as a colorless oil (290 mg, 2.01 mmol, 95 %).

***R*f** = 0.54 (SiO_2, CH/EtOAc, 1:1); **^{1}H-NMR** (700 MHz, $CDCl_3$): δ [ppm] = 9.78 (q, *J* = 1.5 Hz, 1H), 4.07 (td, *J* = 6.2, 1.6 Hz, 2H), 2.49 (td, *J* = 7.0, 1.5 Hz, 2H), 2.04 (d, *J* = 1.5 Hz, 3H), 1.74 – 1.63 (m, 4H); **^{13}C-NMR** (176 MHz, $CDCl_3$): δ [ppm] = 201.9, 171.1, 63.9, 43.3, 28.0, 20.9, 18.6; **HRMS (ESI+)** calculated for $C_7H_{13}O_3^+$ $[M+H]^+$: 145.0859, found : 145.0859.

Synthesis of alcohol 60

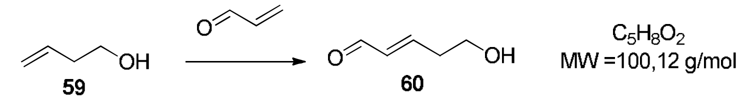

To a solution of but-3-en-1-ol (**59**) (0.18 mL, 2.08 mmol, 1.00 eq) and freshly distilled acrolein (0.42 mL, 6.24 mmol, 3.00 eq) in DCM (3 mL) was added Grubbs II catalyst (10 mg, 17 µmol, 0.10 eq). The mixture was stirred over two nights at room temperature. Solvent was carefully evaporated and

the crude product was directly purified by column chromatography (SiO_2, Pentane/Et_2O, 1:1 to 100 % Et_2O) to give product **60** as a yellow volatile oil (167 mg, 1.67 mmol, 81 %).

***R*$_f$** = 0.23 (SiO_2, Et_2O); **^{1}H-NMR** (700 MHz, $CDCl_3$): δ [ppm] = 9.51 (d, *J* = 7.9 Hz, 1H), 6.89 (dt, *J* = 15.7, 6.9 Hz, 1H), 6.17 (ddt, *J* = 15.7, 7.9, 1.5 Hz, 1H), 3.80 (td, *J* = 6.1, 5.1 Hz, 2H), 2.58 (dtd, *J* = 6.8, 6.2, 1.5 Hz, 2H), 1.60 (t, *J* = 5.3 Hz, 1H); **^{13}C-NMR** (176 MHz, $CDCl_3$): δ [ppm] = 193.7, 154.9, 134.4, 60.7, 35.8.The mass was not found was common MS techniques (EI, ESI, APCI, MALDI).The spectroscopic data are in agreement with those previously reported.[5]

Synthesis of aldehyde 42

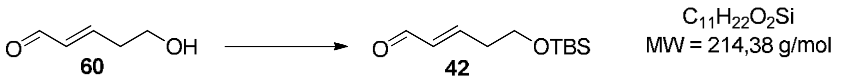

To a solution of alcohol **60** (70 mg, 0.70 mmol, 1.00 eq) in DCM (4 mL) at -78 °C was added 2,6 lutidine (0.12 mL, 1.05 mmol, 1.50 eq) and TBSOTf (0.2 mL, 0.91 mmol, 1.30 eq). The reaction was stirred for 1 h then diluted with DCM (5 mL) and quenched with water (5 mL). After separation of the organic layer, the aqueous layer was extracted with DCM (3*10 mL). The organic layers were combined, dried over $MgSO_4$ and evaporated *in vacuo*. The crude product was purified by column chromatography (SiO_2, CH/EtOAc, 10:1) to give aldehyde **42** as a thick colorless oil (92 mg, 0.43 mmol, 61 %).

***R*$_f$** = 0.65 (SiO_2, CH/EtOAc, 2:1); **^{1}H-NMR** (500 MHz, $CDCl_3$): δ [ppm] = 9.54 (d, *J* = 7.8 Hz, 1H), 6.91 (dt, *J* = 15.7, 6.9 Hz, 1H), 6.20 (ddt, *J* = 15.7, 7.9, 1.5 Hz, 1H), 3.81 (t, *J* = 6.2 Hz, 2H), 2.57 (qd, *J* = 6.3, 1.5 Hz, 2H), 0.92 (s, 9H), 0.09 (s, 6H); **^{13}C-NMR** (125 MHz, $CDCl_3$): δ [ppm] = 194.0, 155.6, 134.3, 61.2, 36.1, 25.9, 18.3, -5.3; **HRMS (ESI+)** calculated for $C_{11}H_{23}O_2Si^+$ $[M+H]^+$: 215:1462, found : 215.1464.

Synthesis of ketone 43a

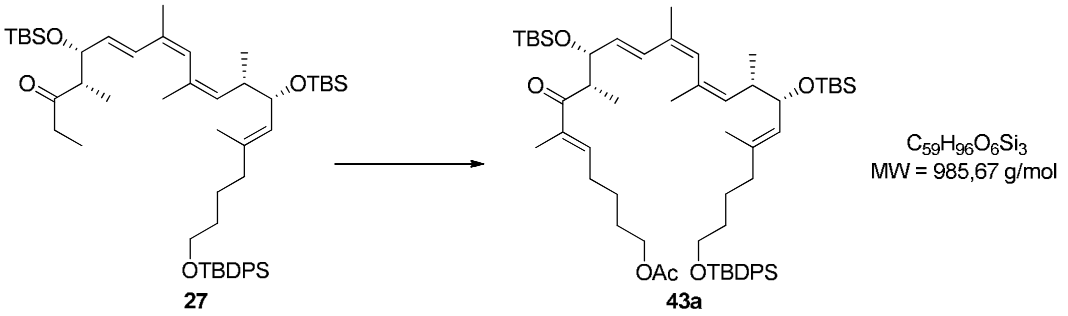

Preparation of LiTMP : To a solution of TMP (32 µL, 0.18 mmol, 4.00 eq) in THF (0.8 mL) at -78 °C was added *n*-BuLi (1.6 M in hexane, 0.12 mL, 0.18 mmol, 4.00 eq). The yellow solution was stirred for 15 min at this temperature and 15 min at 0 °C.

Ketone **27** (40 mg, 47 µmol, 1.00 eq) was diluted in THF (1.5 mL) and dried over 3Å MS for 30 min. The solution was then cooled down at -78 °C, LiTMP (0.50 mL, 94 µmol, 2.00 eq) was added dropwise and the mixture was stirred for 30 min at -78 °C and warmed up to -50 °C for 20 min. The enolate solution was cooled down at -78 °C and aldehyde **41** (10 mg, 70 µmol, 1.50 eq, dried over 3Å MS in 0.2 mL THF) was added dropwise. After 2 h, the reaction mixture was diluted with DCM (3 mL) and quenched with a saturated solution of $NaHCO_3$ (1.5 mL) at 0 °C. After separation of the organic

[5] W. Zhang, J. Bah, A. Wohlfarth, J. Franzén, *Chem. Eur. J.* **2011**, *17*, 13814–13824.

layer, the aqueous layer was extracted with DCM (3*5 mL). The organic layers were combined, dried over $MgSO_4$ and evaporated *in vacuo*. The crude product was purified by column chromatography (SiO_2, CH/EtOAc, 30:1 to 10:1) give the aldol product (39 mg, 39 µmol, 83 %).

The aldol product was directly diluted in THF (2 mL), DMAP (47 mg, 0.39 mmol, 10.0 eq) and Ac_2O (3 µL, 0.35 mmol, 9.00 eq) were added at 0 °C. After 30 min, buffer pH 7 (3 mL) was added. After separation of the organic layer, the aqueous layer was extracted with Et_2O (3*3 mL). The organic layers were combined, dried over $MgSO_4$ and evaporated under vacuum. The crude product was purified by column chromatography (SiO_2, CH/EtOAc, 20:1) to give the protected alcohol (35 mg, 33.4 µmol, 86 %).

The protected alcohol was diluted in THF (2 mL) and DBU (175 µL, 1.27 mmol, 35.0 eq) was added. After two days, the reaction was quenched with buffer ph7 (2 mL). After separation of the organic layer, the aqueous layer was extracted with Et_2O (3*3 mL). The organic layers were combined, dried over $MgSO_4$ and evaporated under vacuum. The crude product was purified by column chromatography (SiO_2, CH/EtOAc, 100:1) to give the desired product **43a** as a colorless oil (31 mg, 31.5 µmol, 67 % over three steps).

***R*f** = 0.48 (SiO_2, CH/EtOAc, 10:1). $[\alpha]_D^{20}$ = + 24.7° (*c* = 0.58, $CHCl_3$); **^{1}H-NMR** (700 MHz, CD_2Cl_2): δ [ppm] = 7.73 – 7.69 (m, 4H), 7.48 – 7.39 (m, 7H), 6.62 – 6.57 (m, 1H), 6.39 (dt, *J* = 15.8, 0.8 Hz, 1H), 5.95 (s, 1H), 5.60 – 5.54 (m, 1H), 5.13 (dddd, *J* = 10.3, 9.1, 2.8, 1.4 Hz, 2H), 4.33 – 4.24 (m, 1H), 4.16 (ddd, *J* = 9.0, 5.9, 1.5 Hz, 1H), 4.09 (td, *J* = 6.6, 5.0 Hz, 2H), 3.72 (t, *J* = 6.0 Hz, 2H), 3.48 – 3.37 (m, 1H), 2.46 – 2.33 (m, 1H), 2.34 – 2.25 (m, 2H), 2.05 (d, *J* = 2.0 Hz, 3H), 2.02 (t, *J* = 7.2 Hz, 2H), 1.82 (d, *J* = 1.3 Hz, 2H), 1.80 (d, *J* = 0.6 Hz, 3H), 1.72 (q, *J* = 0.9 Hz, 3H), 1.71 – 1.66 (m, 2H), 1.62 (d, *J* = 1.4 Hz, 3H), 1.57 (s, 15H), 1.11 (dd, *J* = 6.8, 2.0 Hz, 3H), 1.08 (s, 8H), 0.94 – 0.89 (m, 11H), 0.89 – 0.88 (m, 9H), 0.06 (s, 3H), 0.03 (s, 3H), 0.02 (s, 3H), 0.01 (s, 3H). **^{13}C-NMR** (176 MHz, CD_2Cl_2): δ [ppm] = 203.6, 170.8, 141.3, 137.9, 135.5, 135.4, 134.2, 132.8, 132.2, 132.0, 131.4, 130.1, 129.5, 129.3, 127.6, 127.2, 76.8, 72.9, 64.0, 63.8, 46.4, 40.6, 39.3, 32.2, 28.6, 28.4, 26.6, 25.6, 25.1, 24.6, 24.0, 20.7, 20.1, 19.6, 18.0, 16.4, 15.5, 14.0, 11.3, -4.2, -4.6, -5.1, -5.2. **HRMS (ESI+)** calculated for $C_{59}H_{96}O_6Si_3Na^+$ $[M+Na]^+$: 1007.6407 found : 1007.6407.

Synthesis of ketone 43b

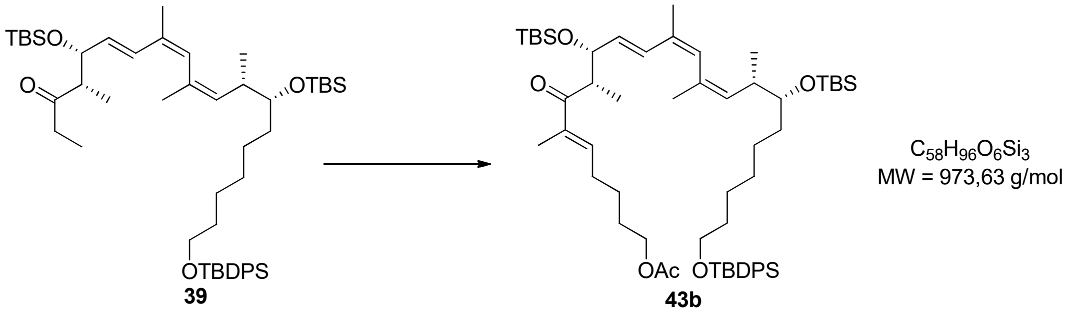

39 **43b**

Preparation of LiTMP : To a solution of TMP (120 µL, 0.70 mmol, 4.00 eq) in THF (2 mL) at -78 °C was added *n*-BuLi (2.5 M in hexane, 0.28 mL, 0.70 mmol, 4.00 eq). The yellow solution was stirred for 15 min at this temperature and 15 min at 0 °C.

Ketone **39** (150 mg, 176 µmol, 1.00 eq) was diluted in THF (3 mL) and dried over 3Å MS for 30 min. The solution was then cooled down at -78 °C, LiTMP (1.2 mL, 0.35 mmol, 2.00 eq) was added dropwise and the mixture was stirred for 30 min at -78 °C and warmed up to -50 °C and for 20 min. The enolate solution was then cooled down at -78 °C and aldehyde **41** (38 mg, 265 µmol, 1.50 eq,

dried over 3Å MS in 0.2 mL THF) was added dropwise. After 2 h, the reaction mixture was diluted with DCM (4 mL) and quenched with a saturated solution of $NaHCO_3$ (4 mL) at 0 °C. After separation of the organic layer, the aqueous layer was extracted with DCM (3*10 mL). The organic layers were combined, dried over $MgSO_4$ and evaporated *in vacuo*. The crude product was purified by column chromatography (SiO_2, CH/EtOAc, 30:1 to 10:1) give the aldol product (148 mg, 149 µmol, 85 %) and the starting material (13.5 mg, 16 µmol, 9 %, 94 % brsm).

The mixture was directly diluted in THF (5 mL), DMAP (182 mg, 0.15 mmol, 10.0 eq) and Ac_2O (126 µL, 1.34 mmol, 9.00 eq) were added at 0 °C. After 30 min, buffer pH 7 (5 mL) was added and the organic layer was separated. The aqueous layer was extracted with Et_2O (3*5 mL), the organic layers were combined, dried over $MgSO_4$ and evaporated under vacuum. The crude product was purified by column chromatography (SiO_2, CH/EtOAc, 20:1) give the protected alcohol (135 mg, 130 µmol, 87 %).

The protected alcohol was then diluted in THF (8 mL) and DBU (0.68 mL, 4.57 mmol, 35.0 eq) was added at room temperature. After two days of stirring, the reaction was quenched with buffer pH7 (10 mL) and the aqueous layer was extracted with Et_2O (3*10 mL). The organic layers were combined, dried over $MgSO_4$ and evaporated under vacuum. The crude product was purified by column chromatography (SiO_2, CH/EtOAc, 50:1) to give ketone **43b** as a colorless oil (105 mg, 108 µmol, 61 % over three steps).

***R*f** = 0.48 (SiO_2, CH/EtOAc, 10:1); $[\alpha]_D^{20}$ = + 10.9° (*c* = 0.35, $CHCl_3$); **^{1}H-NMR** (700 MHz, CD_2Cl_2): δ [ppm] = 7.66 (dt, *J* = 6.8, 1.5 Hz, 4H), 7.42 (ddt, *J* = 8.4, 6.5, 1.5 Hz, 2H), 7.40 – 7.36 (m, 4H), 6.59 – 6.53 (m, 1H), 6.33 (dt, *J* = 15.8, 0.8 Hz, 1H), 5.91 – 5.86 (m, 1H), 5.59 – 5.53 (m, 1H), 5.19 – 5.16 (m, 1H), 4.28 – 4.25 (m, 1H), 4.05 (q, *J* = 6.5 Hz, 2H), 3.66 (td, *J* = 6.5, 2.2 Hz, 2H), 3.44 (td, *J* = 6.6, 5.8, 3.5 Hz, 1H), 3.39 (q, *J* = 6.9 Hz, 1H), 2.44 – 2.37 (m, 1H), 2.30 – 2.23 (m, 2H), 2.04 – 1.99 (m, 3H), 1.79 – 1.77 (m, 3H), 1.76 (t, *J* = 1.1 Hz, 3H), 1.70 (p, *J* = 1.3 Hz, 2H), 1.68 – 1.64 (m, 2H), 1.59 – 1.55 (m, 2H), 1.37 – 1.21 (m, 8H), 1.10 – 1.06 (m, 2H), 1.04 (s, 9H), 0.91 – 0.89 (m, 3H), 0.88 (d, *J* = 2.7 Hz, 9H), 0.87 (s, 8H), -0.00 – -0.03 (m, 12H); **^{13}C-NMR** (176 MHz, CD_2Cl_2): δ [ppm] = 203.6, 170.8, 141.3, 137.8, 135.5, 134.2, 132.5, 132.4, 131.6, 131.5, 129.9, 129.5, 129.0, 127.5, 76.6, 75.9, 64.0, 63.9, 46.4, 38.6, 33.2, 32.6, 29.6, 28.6, 28.4, 26.6, 26.2, 25.9, 25.7, 25.6, 25.1, 24.6, 20.7, 20.0, 19.1, 18.0, 15.2, 14.0, 11.4, -4.3, -4.7, -4.8, -5.1; **HRMS (ESI+)** calculated for $C_{58}H_{100}O_6Si_3N^+$ $[M+NH_4]^+$: 990.6853, found 990.6853.

Synthesis of ketone 44a

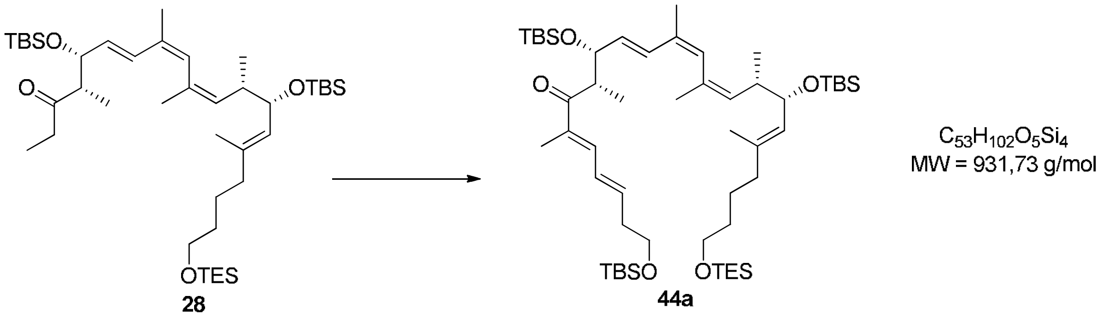

28 **44a**

Preparation of LiTMP: To a solution of TMP (94 µL, 0.28 mmol, 4.00 eq) in THF (2 mL) at -78 °C was added *n*-BuLi (2.5 M, 0.11 mL, 0.28 mmol, 4.00 eq). The yellow solution was stirred for 15 min at this temperature and 15 min at 0 °C.

Ketone **28** (104 mg, 140 µmol, 1.00 eq) was diluted in THF (3 mL). The solution was cooled down at -78 °C and LiTMP (1.1 mL, 280 µmol, 2.00 eq) was added dropwise. The mixture was stirred for 30 min at -78 °C and warmed up to -50 °C for 20 min. The enolate solution was cooled down again

at -78 °C and aldehyde **42** (45 mg, 211 µmol, 1.50 eq) was added dropwise. After 2 h, the reaction mixture was diluted with DCM (5 mL) and quenched with a saturated solution of $NaHCO_3$ (4 mL) at 0 °C. After separation of the organic layer, the aqueous layer was extracted with DCM (3*10 mL). The organic layers were combined, dried over $MgSO_4$ and evaporated *in vacuo*. The crude product was purified by column chromatography (SiO_2, CH/EtOAc, 100:1 to 20:1) give the aldol product (117 mg, 123 µmol, 88 %).

The mixture was directly diluted in THF (4 mL), DMAP (84 mg, 0.71 mmol, 5.00 eq) and Ac_2O (54 µL, 0.57 mmol, 4.00 eq) were added at 0 °C. After 30 min, buffer pH 7 (5 mL) was added. After separation of the organic layer, the aqueous layer was extracted with Et_2O (3*5 mL). The organic layers were combined, dried over $MgSO_4$ and evaporated under vacuum. The crude product was purified by column chromatography (SiO_2, CH/EtOAc, 30:1) to give the protected alcohol (111 mg, 112 µmol, 91 %).

The protected alcohol was diluted in THF (4 mL) and DBU (0.58 mL, 3.92 mmol, 35.0 eq) was added. After one night, the reaction was quenched with buffer pH7 (10 mL). After separation of the organic layer, the aqueous layer was extracted with EtOAc (3*5 mL). The organic layers were combined, dried over $MgSO_4$ and evaporated under vacuum. The crude product was purified by column chromatography (SiO_2, CH/EtOAc, 50:1) affording ketone **44a** as a colorless oil (84 mg, 90 µmol, 64 % over three steps).

***R*f** = 0.67 (SiO_2, CH/EtOAc, 10:1); $[\boldsymbol{\alpha}]_{\boldsymbol{D}}^{\mathbf{20}}$ = - 14.7° (*c* = 0.32, $CHCl_3$); **^{1}H-NMR** (700 MHz, CD_2Cl_2): δ [ppm] = 7.03 – 6.98 (m, 1H), 6.53 – 6.47 (m, 1H), 6.37 – 6.33 (d, 1H), 6.17 – 6.11 (m, 1H), 5.90 (dd, *J* = 11.8, 6.9 Hz, 1H), 5.56 – 5.52 (m, 1H), 5.11 – 5.06 (m, 1H), 4.26 – 4.22 (m, 1H), 4.14 – 4.10 (m, 1H), 3.72 – 3.70 (m, 2H), 3.60 (t, *J* = 6.2 Hz, 2H), 3.42 (dd, *J* = 13.8, 6.9 Hz, 1H), 2.41 (q, *J* = 6.5 Hz, 2H), 2.32 (ddd, *J* = 15.6, 9.5, 4.6 Hz, 1H), 2.00 – 1.96 (m, 2H), 1.78 (s, 3H), 1.78 – 1.76 (m, 6H), 1.58 (d, *J* = 1.1 Hz, 3H), 1.49 – 1.44 (m, 4H), 1.09 (d, *J* = 6.8 Hz, 1H), 0.96 – 0.94 (m, 9H), 0.90 – 0.89 (m, 12H), 0.87 – 0.86 (m, 9H), 0.85 (d, *J* = 1.2 Hz, 9H), 0.60 – 0.57 (m, 6H), 0.06 (d, *J* = 2.2 Hz, 6H), 0.02 (d, *J* = 1.4 Hz, 3H), -0.01 – -0.02 (m, 3H), -0.02 – -0.03 (m, 3H), -0.04 (d, *J* = 1.4 Hz, 3H); **^{13}C-NMR** (176 MHz, CD_2Cl_2): δ [ppm] = 203.6, 147.7, 139.8, 138.0, 135.6, 135.2, 132.6, 132.3, 131.9, 131.4, 130.1, 129.2, 128.4, 127.2, 76.8, 73.0, 62.6, 62.2, 46.3, 40.6, 39.6, 36.9, 32.7, 25.6, 24.6, 24.3, 20.3, 18.2, 17.7, 16.5, 15.5, 14.2, 11.5, 6.5, 4.3, -4.2, -4.6, -5.1, -5.2, -5.6; **HRMS (ESI+)** calculated for $C_{53}H_{102}O_5Si_4Na^+$ $[M+Na]^+$: 953:6697, found 953.6697.

Synthesis of ketone 44b

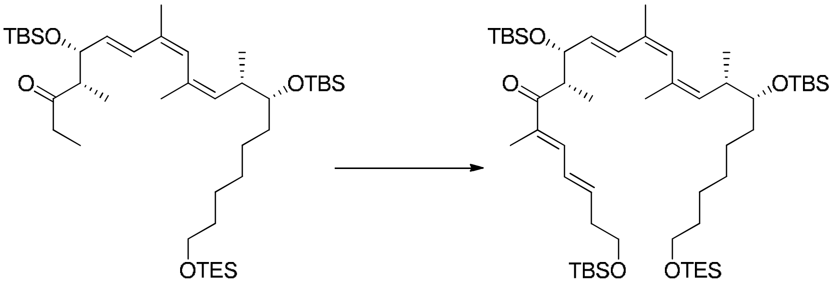

40 44b

$C_{52}H_{102}O_5Si_4$
MW = 919,72 g/mol

Preparation of LiTMP: To a solution of TMP (134 µL, 0.4 mmol, 4.00 eq) in THF (2 mL) at -78 °C was added *n*-BuLi (2.5 M in hexane, 0.30 mL, 0.4 mmol, 4.00 eq). The yellow solution was stirred for 15 min at this temperature and 15 min at 0 °C.

Ketone **40** (145 mg, 200 µmol, 1.00 eq) was diluted in THF (3 mL) and dried over 3Å MS for 30 min. The solution was then cooled down at -78 °C, LiTMP (1.3 mL, 0.4 mmol, 2.00 eq) was added dropwise and the mixture was stirred for 30 min at -78 °C and warmed up to -50 °C for 20 min. The enolate solution was then cooled down at -78 °C and aldehyde **42** (65 mg, 300 µmol, 1.50 eq) was added dropwise. After 2 h, the reaction mixture was diluted with DCM (5 mL) and quenched with $NaHCO_3$ (4 mL) at 0 °C. After separation of the organic layer, the aqueous layer was extracted with DCM (3*10 mL). The organic layers were combined, dried over $MgSO_4$ and evaporated *in vacuo*. The crude product was purified by column chromatography (SiO_2, CH/EtOAc, 100:1 to 50:1) give the aldol product (148 mg, 157 µmol, 78 %).

The aldol product was directly diluted in THF (5 mL), DMAP (192 mg, 0.157 mmol, 10.0 eq) followed by Ac_2O (134 µL, 1.42 mmol, 9.00 eq) were added at 0 °C. After 30 min, buffer pH 7 (5 mL) was added. After separation of the organic layer, the aqueous layer was extracted with Et_2O (3*5 mL). The organic layers were combined, dried over $MgSO_4$ and evaporated under vacuum. The crude product was purified by column chromatography (SiO_2, CH/EtOAc, 50:1) give the protected alcohol (130 mg, 133 µmol, 85 %).

The protected alcohol was diluted in THF (5 mL) and DBU (0.69 mL, 4.64 mmol, 35.0 eq) was added at room temperature. After one night of stirring, the reaction was quenched with pH7 buffer (10 mL). After the separation of the organic layer, the aqueous layer was extracted with EtOAc (3*5 mL). The organic layers were combined, dried over $MgSO_4$ and evaporated under vacuum. The crude product was purified by column chromatography (SiO_2, CH/EtOAc, 50:1) to give ketone **44b** as a colorless oil (108 mg, 117 µmol, 58 % over three steps).

***R*f** = 0.67 (SiO_2, CH/EtOAc, 10:1); $[\alpha]_D^{20}$ = - 29.6° (*c* = 0.23, $CHCl_3$); **^{1}H-NMR** (500 MHz, CD_2Cl_2): δ [ppm] = 7.07 – 6.99 (m, 1H), 6.54 (dd, *J* = 15.3, 10.7 Hz, 1H), 6.36 (d, *J* = 15.8 Hz, 1H), 6.17 (dt, *J* = 14.3, 6.9 Hz, 1H), 5.91 (s, 1H), 5.63 – 5.54 (m, 1H), 5.21 (dt, *J* = 9.6, 1.6 Hz, 1H), 4.35 – 4.27 (m, 1H), 3.75 (t, *J* = 6.4 Hz, 2H), 3.62 (td, *J* = 6.6, 1.3 Hz, 2H), 3.51 – 3.37 (m, 2H), 2.45 (q, *J* = 6.6 Hz, 3H), 1.83 (d, *J* = 1.1 Hz, 3H), 1.82 – 1.76 (m, 6H), 1.51 (dd, *J* = 10.5, 4.0 Hz, 2H), 1.33 (dd, *J* = 11.4, 7.3 Hz, 8H), 1.15 – 1.10 (m, 3H), 1.02 – 0.96 (m, 9H), 0.93 (s, 12H), 0.91 (t, *J* = 2.4 Hz, 17H), 0.62 (qd, *J* = 7.9, 0.8 Hz, 6H), 0.09 (s, 6H), 0.06 (s, 3H), 0.05 (s, 3H), -0.01 (s, 3H), -0.04 (s, 3H); **^{13}C-NMR** (125 MHz, CD_2Cl_2): δ [ppm] = 203.6, 139.7, 138.1, 134.9, 132.5, 132.4, 131.6, 129.8, 128.9, 128.3, 76.5, 75.9, 62.8, 62.8, 62.2, 53.8, 53.6, 53.5, 53.4, 53.2, 53.1, 52.9, 46.3, 38.6, 36.8, 33.1, 32.9, 30.0, 29.7, 26.2, 25.9, 25.7, 25.6, 25.6, 25.4, 24.6, 19.9, 18.1, 18.0, 15.2, 14.1, 11.5, 6.5, 4.3, -4.3, -4.7, -4.8, -4.8, -5.1, -5.6, -5.7; **HRMS (ESI+)** calculated for $C_{52}H_{102}O_5Si_4Na^+$ $[M+Na]^+$: 941.6679, found 941.6679.

2.4 Completion of analogue 5

Synthesis of methyl ether 46a

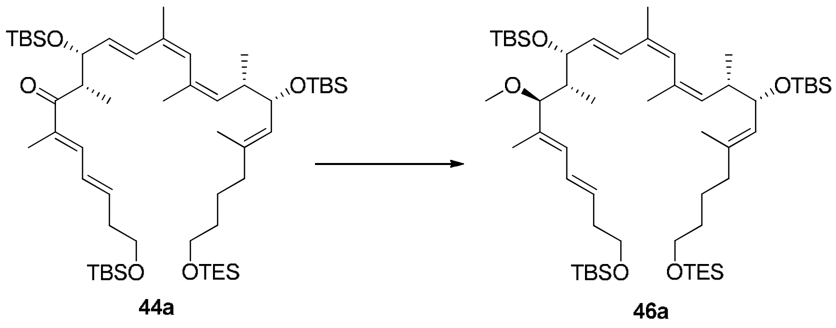

$C_{54}H_{106}O_5Si_4$
MW = 947,78 g/mol

To a solution of ketone **44a** (84 mg, 90 µmol, 1.00 eq) in MeOH (3 mL) and THF (1 mL) at 0 °C was added $NaBH_4$ (123.6 mg, 360 µmol, 4.00 eq) and the solution was warmed up to room temperature. After 3 h, the reaction was diluted with EtOAc (5 mL) and quenched carefully with a saturated solution of NH_4Cl (4 mL) at 0 °C. After separation of the organic layer, the aqueous layer was extracted with EtOAc (3* 10 mL). The organic layers were combined, dried over $MgSO_4$ and evaporated *in vacuo*. The crude product was purified by column chromatography (SiO_2, CH/EtOAc, 50:1) to give the alcohol as a thick oil (67 mg, 73 µmol, 86 %, *dr* = 8:1).

To a solution of alcohol (67 mg, 73 µmol, 1.00 eq) in DCM (4 mL) at 0 °C was added proton sponge (86 mg, 0.40 mmol, 5.50 eq) followed by MeO_3BF_4 (54 mg, 0.36 mmol, 5.00 eq). The reaction was stirred for 4.5 h at 0 °C. After this time, a saturated solution of $NaHCO_3$ (2.5 mL) was added at 0 °C. After separation of the organic layer, the aqueous layer was extracted with DCM (3*5 mL). The organic layers were combined, dried over $MgSO_4$ and evaporated *in vacuo*. The crude product was purified by column chromatography (SiO_2, CH/EtOAc, 80:1) to give the methyl ether **46a** (50 mg, 53 µmol, 72 %).

$\boldsymbol{R}_f$ = 0.69 (SiO_2, CH/EtOAc, 10:1); $[\alpha]_D^{20}$ = + 15.6° (*c* = 0.41, $CHCl_3$); **^{1}H-NMR** (700 MHz, $CDCl_3$): δ [ppm] = 6.45 – 6.41 (m, 1H), 6.38 – 6.32 (m, 1H), 5.92 (t, *J* = 8.1 Hz, 2H), 5.86 (s, 1H), 5.73 – 5.62 (m, 2H), 5.08 (ddd, *J* = 8.3, 5.1, 1.3 Hz, 2H), 4.69 (d, *J* = 7.0 Hz, 1H), 4.15 – 4.10 (m, 2H), 3.66 (t, *J* = 6.6 Hz, 2H), 3.60 (t, *J* = 6.1 Hz, 2H), 3.34 (d, *J* = 9.9 Hz, 1H), 3.10 (s, 3H), 2.39 – 2.29 (m, 3H), 1.98 (t, *J* = 7.2 Hz, 2H), 1.85 – 1.84 (m, 3H), 1.79 (s, 3H), 1.57 (d, *J* = 1.4 Hz, 6H), 1.50 – 1.43 (m, 4H), 0.95 (dd, *J* = 10.3, 5.5 Hz, 9H), 0.92 (s, 9H), 0.89 (s, 12H), 0.86 – 0.86 (m, 3H), 0.64 – 0.62 (m, 3H), 0.59 (q, *J* = 8.0 Hz, 6H), 0.05 (d, *J* = 1.3 Hz, 3H), 0.05 (s, 6H), -0.01 (s, 3H), -0.01 (s, 3H), -0.04 (s, 3H). **^{13}C-NMR** (176 MHz, $CDCl_3$): δ [ppm] = 135.4, 134.2, 133.9, 132.5, 132.1, 130.7, 129.6, 129.1, 127.7, 126.9, 88.1, 72.8, 71.7, 62.8, 62.5, 55.3, 42.6, 40.4, 39.3, 36.5, 32.5, 25.7, 25.6, 25.6, 24.5, 24.1, 20.1, 18.1, 18.0, 17.9, 16.4, 15.1, 10.3, 8.7, 6.5, 4.3, -4.1, -4.7, -5.3, -5.4, -5.6; **HRMS (ESI+)** calculated for $C_{54}H_{110}O_5Si_3N^+$ $[M+NH_4]^+$: 964.7456, found 964.7456.

Synthesis of alcohol 48a

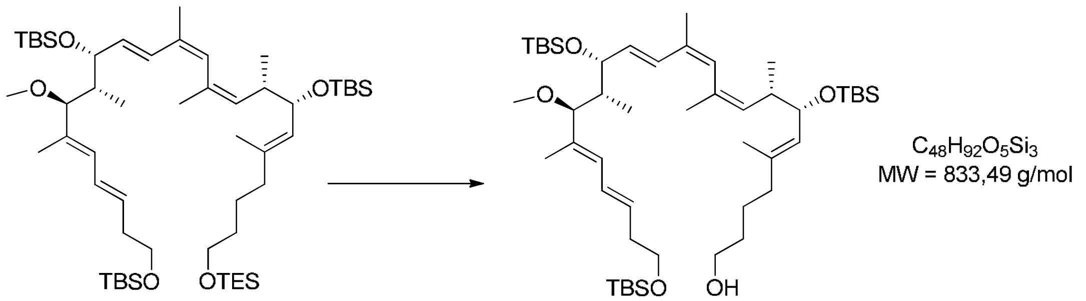

46a → **48a**

To a solution of compound **46a** (48 mg, 51 µmol, 1.00 eq) in MeOH (7 mL) was added K_2CO_3 (210 mg, 1.53 mmol, 30.0 eq) at 0 °C. The solution was warmed up to room temperature and stirred overnight. The reaction was quenched with a saturated solution of $NaHCO_3$ (10 mL) and diluted with EtOAc (10 mL). After separation of the organic layer, the aqueous layer was extracted with EtOAc (3*10 mL). The combined organic layers were dried over $MgSO_4$ and evaporated *in vacuo.* The crude product was purified by column chromatography (SiO_2, CH/EtOAc, 15:1) to give alcohol **48a** (35 mg, 42 µmol, 82 %).

***R*f** = 0.22 (SiO_2, CH/EtOAc, 10:1); $[\alpha]_D^{20}$ = + 10.4° (*c* = 0.25, $CHCl_3$); **^{1}H-NMR** (500 MHz, $CDCl_3$): δ [ppm] = 6.43 (d, *J* = 15.9 Hz, 1H), 6.35 (dd, *J* = 15.1, 10.8 Hz, 1H), 5.91 (d, *J* = 11.2 Hz, 1H), 5.86 (s, 1H), 5.73 – 5.63 (m, 2H), 5.09 (dd, *J* = 12.8, 5.3 Hz, 2H), 4.69 (d, *J* = 7.0 Hz, 1H), 4.16 – 4.10 (m, 1H), 3.66 (t, *J* = 6.6 Hz, 2H), 3.60 (t, *J* = 11.8 Hz, 2H), 3.34 (d, *J* = 9.9 Hz, 1H), 3.10 (s, 3H), 2.39 – 2.35 (m, 1H), 2.32 (dd, *J* = 13.4, 6.7 Hz, 2H), 2.02 – 1.98 (m, 2H), 1.85 (d, *J* = 1.2 Hz, 3H), 1.79 (s, 3H), 1.58 (dd, *J* = 5.0, 3.8 Hz, 6H), 1.50 – 1.43 (m, 4H), 0.92 (s, 9H), 0.88 (d, *J* = 1.9 Hz, 9H), 0.86 (d, *J* = 3.2 Hz, 3H), 0.85 (s, 9H), 0.64 – 0.62 (m, 3H), 0.05 (d, *J* = 1.5 Hz, 3H), 0.05 (s, 6H), -0.01 (s, 3H), -0.01 (s, 3H), -0.04 (s, 3H). **^{13}C-NMR** (126 MHz, $CDCl_3$): δ [ppm] = 137.1, 136.2, 135.9, 134.5, 134.4, 134.1, 132.6, 131.6, 131.5, 131.0, 129.7, 129.1, 90.1, 74.7, 73.7, 64.7, 64.5, 57.2, 44.6, 42.3, 41.2, 38.4, 34.6, 27.7, 27.6, 27.6, 27.5, 26.4, 25.8, 22.1, 20.1, 18.3, 17.0, 12.3, 10.7, -2.2, -2.8, -3.3, -3.5, -3.7. **HRMS (ESI+)** calculated for $C_{48}H_{92}O_5Si_3Na^+$ $[M+Na]^+$: 855.6145, found 855.6145.

Synthesis of analogue 5

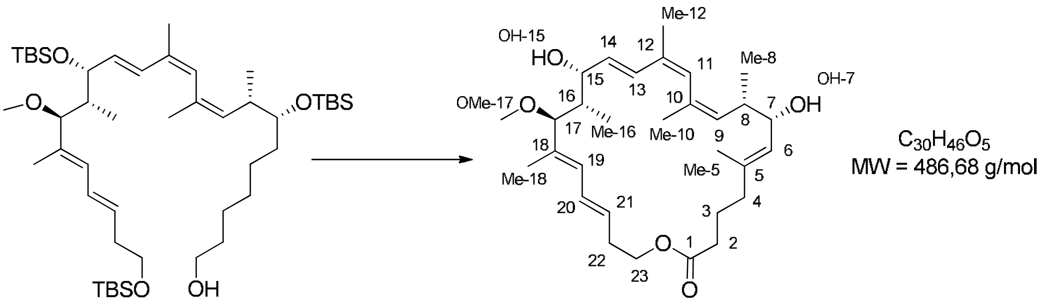

48a → **5**

To a solution of DMSO (30 µL, 420 µmol, 10.0 eq), sulfur trioxide pyridine complex (20 mg, 126 µmol, 3.00 eq) and DIEA (29 µL, 168 µmol, 4.00 eq) in DCM (3 mL) at 0 °C was added alcohol **48a** (35 mg, 42 µmol, 1.00 eq). The solution was stirred at 0 °C for 1.5 h. After this time the reaction was quenched with aqueous saturated solution of $NaHCO_3$ (3 mL) and diluted with DCM (3 mL). After separation of the organic layer, the aqueous layer was extracted with DCM (3*5 mL).The organic layers were combined, dried over $MgSO_4$ and evaporated *in vacuo*.

The crude aldehyde was diluted in *tert*-butanol (2 mL) and 2-methylbut-2-ene (0.2 mL) and cooled at 0 °C. A solution of $NaClO_2$ (12 mg, 134 µmol, 3.20 eq), KH_2PO_4 (23 mg, 168 µmol, 4.00 eq) in H_2O (2 mL) was added to the solution and the reaction was stirred for 1 h at room temperature. Saturated aqueous solution of NaCl (4 mL) was added followed by DCM (4 mL). After separation of the organic layer, the aqueous layer was extracted with DCM (3*5 mL). The organic layers were combined, dried over $MgSO_4$ and evaporated *in vacuo*. The crude product was purified by column chromatography (SiO_2, CH/EtOAc, 10:1) to give the carboxylic acid (23 mg, 27 µmol, 64 % over two steps).

HF-pyridine stock solution: To a solution of THF (1.3 mL) and pyridine (0.75 mL) at 0 °C was added HF-pyr (0.25 mL, 70 % HF).

To a solution of carboxylic acid (23 mg, 27 µmol, 1.00 eq) in THF (0.8 mL) at 0 °C was added the HF-pyr stock solution (0.34 mL). The reaction was stirred for 6 h at 0 °C. The reaction was quenched with a saturated solution of $NaHCO_3$ (10 mL) and diluted with DCM (3 mL). After separation of the organic layer, the aqueous layer was extracted with DCM (3*5 mL). The combined organic layers were washed with brine (5 mL), dried over $MgSO_4$ and evaporated *in vacuo.* The crude product was purified by column chromatography (SiO_2, CH/EtOAc, 3:2) to give the precursor for Shiina esterification (6.3 mg, 8.6 µmol, 32 %).

MNBA (15 mg, 43 µmol, 5.00 eq), DMAP (7.3 mg, 60 µmol, 7.00 eq) and 3Å MS were dried for 1 h under high vacuum before DCM was added (4 mL). The seco acid (6.3 mg, 8.6 µmol, 1.00 eq) was diluted in DCM (5 mL) and added to the solution over 20 h. Two hours after completion of the addition, the reaction was quenched at 0 °C with pH7 Buffer (3 mL). After separation of the organic layer, the aqueous layer was extracted with DCM (3*5 mL). The combined organic layers were washed with brine (5 mL), dried over $MgSO_4$ and evaporated *in vacuo.* The crude product was purified by column chromatography (SiO_2, CH/EtOAc, 50:1) to give the desired macrolactone (5.1 mg, 7.1 µmol, 83 %).

The macrolactone (5.1 mg, 7.1 µmol, 1.00 eq) was diluted in THF (0.3 mL) and cooled down at 0 °C. Pyridine (0.3 mL) was added followed by HF-pyr (70 % HF, 0.3 mL). After two days the reaction was quenched at 0 °C with pH7 buffer (5 mL). After separation of the organic layer, the aqueous layer was extracted with EtOAc (3*5 mL).The organic layers were combined, dried over $MgSO_4$ and evaporated *in vacuo*. The crude product was purified by column chromatography (SiO_2, CH/EtOAc, 5:1 to 3:1) to give **5** as a white solid (1.2 mg, 3.4 µmol, 35 %).

R_f = 0.45 (SiO_2, CH/EtOAc, 3:1); $[\alpha]_D^{20}$ = - 33.4° (*c* = 0.12, $CHCl_3$); **^{1}H-NMR** (700 MHz, CD_2Cl_2): δ [ppm] = 6.53 (d, *J* = 16.0 Hz, 1H, **H-13**), 6.32 (dd, *J* = 15.1, 10.9 Hz, 1H, **H-20**), 5.93 (d, *J* = 10.7 Hz, 1H, **H-19**), 5.67 (dd, *J* = 16.0, 4.8 Hz, 1H, **H-14**), 5.63 (s, 1H, **H-11**), 5.60 – 5.56 (m, 1H, **H-21**), 5.20 – 5.17 (m, 1H, **H-9**), 5.01 (dd, *J* = 9.0, 1.1 Hz, 1H, **H-6**), 4.40 (d, *J* = 4.5 Hz, 1H, **H-15**), 4.39 – 4.37 (m, 1H, **1H-23**), 4.01 – 3.97 (m, 1H, **1H-23**), 3.95 (d, *J* = 9.4 Hz, 1H, **H-7**), 3.50 (d, *J* = 9.0 Hz, 1H, **H-17**), 3.19 (s, 3H, **OMe-17**), 2.44 (dt, *J* = 12.0, 3.9 Hz, 2H, **22**), 2.24 (ddd, *J* = 9.9, 8.7, 5.4 Hz, 1H, **H-8**), 2.21 – 2.19 (m, 2H, **2**), 1.95 (td, *J* = 9.8, 5.8 Hz, 2H, **4**), 1.89 (d, *J* = 2.3 Hz, 3H, **Me-12**), 1.82 (ddd, *J* = 9.1, 7.3, 2.0 Hz, 1H, **H-16**), 1.77 (s, 3H, **Me-10**), 1.71 (dd, *J* = 6.3, 2.9 Hz, 2H, **3**), 1.67 (d, *J* = 1.2 Hz, 3H, **Me-5**), 1.63 (s, 3H, **Me-18**), 0.71 (d, *J* = 6.7 Hz, 3H, **Me-8**), 0.57 (d, *J* = 7.2 Hz, 3H, **Me-16**); **^{13}C-NMR** (176 MHz, $CDCl_3$): δ [ppm] = 173.3 (**C-1**), 139.2 (**C-5**), 134.6 (**C-10**), 133.7 (**C-18**), 132.6 (**C-14**), 132.0 (**C-12**), 131.1 (**C-9**), 130.8 (**C-21**), 128.9 (**C-19**), 128.5 (**C-11**), 128.0 (**C-20**), 127.9 (**C-13**), 126.8 (**C-6**), 89.3 (**C-17**), 72.9 (**C-7**), 72.8 (**C-15**), 62.6 (**C-**

23), 55.9 (**OMe-17**), 40.9 (**C-16**), 40.3 (**C-8**), 39.2 (**C-4**), 34.5 (**C-2**), 32.7 (**C-22**), 24.3 (**Me-10**), 23.8 (**C-3**), 19.8 (**Me-12**), 17.1 (**Me-8**), 16.5 (**Me-18**), 11.8 (**Me-16**), 10.6 (**Me-5**); **HRMS (ESI+)** calculated for $C_{30}H_{46}O_5Na^+$ $[M+Na]^+$: 509.3237, found 509.3237.

2.5 Completion of analogue 6

Synthesis of methyl ether 45a

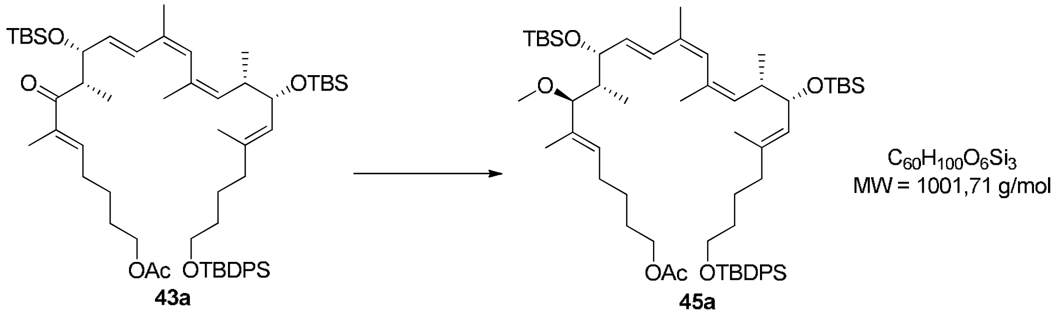

To a solution of ketone **43a** (65 mg, 66 µmol, 1.00 eq) in MeOH (3 mL) and THF (1 mL) at 0 °C was added $NaBH_4$ (5 mg, 132 µmol, 2.00 eq) and the solution was warmed up to room temperature. After 2h, more $NaBH_4$ (5 mg, 132 µmol, 2.00 eq) was added. After 3 h, the reaction was diluted with EtOAc (5 mL) and quenched with a saturated solution of NH_4Cl (4 mL) at 0 °C. After separation of the organic layer, the aqueous layer was extracted with EtOAc (3* 10 mL). The organic layers were combined, dried over $MgSO_4$ and evaporated *in vacuo*. The crude product was purified by column chromatography (SiO_2, CH/EtOAc, 60:1 to 30:1) to give the alcohol as a thick oil (44 mg, 44.5 µmol, 67 %, *dr* = 10:1).

To a solution of alcohol (41 mg, 41.5 µmol, 1.00 eq) in DCM (2.5 mL) at 0 °C was added proton sponge (46 mg, 0.23 mmol, 5.50 eq) followed by MeO_3BF_4 (31 mg, 0.21 mmol, 5.00 eq). The reaction was stirred for 3 h at 0 °C. After this time, a saturated solution of $NaHCO_3$ (2.5 mL) was added at 0 °C. After separation of the organic layer, the aqueous layer was extracted with DCM (3*5 mL). The organic layers were combined, dried over $MgSO_4$ and evaporated *in vacuo*. The crude product was purified by column chromatography (SiO_2, CH/EtOAc, 60:1) to give methyl ether **45a** (37 mg, 37 µmol, 89 %).

$\boldsymbol{R}_f$ = 0.46 (SiO_2, CH/EtOAc, 10:1); $[\alpha]_D^{20}$ = + 29.8° (*c* = 0.48, $CHCl_3$); **^{1}H-NMR** (700 MHz, CD_2Cl_2): δ [ppm] = 7.71 – 7.70 (m, 4H), 7.47 – 7.44 (m, 2H), 7.43 – 7.41 (m, 4H), 6.46 (dt, *J* = 15.9, 0.9 Hz, 1H), 5.75 (ddd, *J* = 15.9, 6.9, 0.7 Hz, 1H), 5.35 – 5.33 (m, 1H), 5.12 (dddq, *J* = 9.7, 4.3, 3.0, 1.4 Hz, 2H), 4.72 (dt, *J* = 7.0, 1.5 Hz, 1H), 4.16 (dd, *J* = 9.0, 5.8 Hz, 1H), 4.07 (t, *J* = 6.7 Hz, 2H), 3.71 (t, *J* = 6.2 Hz, 2H), 3.34 (d, *J* = 10.0 Hz, 1H), 3.13 (s, 3H), 2.44 – 2.38 (m, 1H), 2.18 – 2.09 (m, 2H), 2.04 (s, 4H), 2.03 – 1.99 (m, 2H), 1.88 (d, *J* = 1.4 Hz, 3H), 1.82 (dt, *J* = 2.8, 1.4 Hz, 2H), 1.70 – 1.64 (m, 3H), 1.62 – 1.57 (m, 7H), 1.50 – 1.44 (m, 6H), 1.07 (s, 9H), 0.95 (s, 9H), 0.93 – 0.92 (m, 3H), 0.88 (d, *J* = 2.7 Hz, 10H), 0.67 (dd, **J** = 6.9, 2.4 Hz, 3H), 0.08 (d, *J* = 4.3 Hz, 3H), 0.02 (s, 6H), 0.01 (s, 3H); **^{13}C-NMR** (176 MHz, CD_2Cl_2): δ [ppm] = 170.9, 135.5, 135.4, 134.2, 134.0, 133.5, 132.6, 132.5, 132.2, 130.2, 129.5, 129.1, 127.7, 127.6, 127.1, 88.3, 72.8, 71.8, 64.3, 63.8, 55.1, 42.4, 40.5, 32.2, 28.3, 27.1, 16.6, 25.9, 25.7, 25.6, 24.5, 24.0, 20.7, 20.2, 19.1, 18.1, 18.0, 16.3, 15.1, 9.8, 8.9, -4.1, -4.6, -5.2, -5.4; **HRMS (ESI+)** calculated for $C_{60}H_{100}O_6Si_3Na$ $[M+Na]^+$: 1023.6720 found : 1023.6720.

Synthesis of alcohol 47a

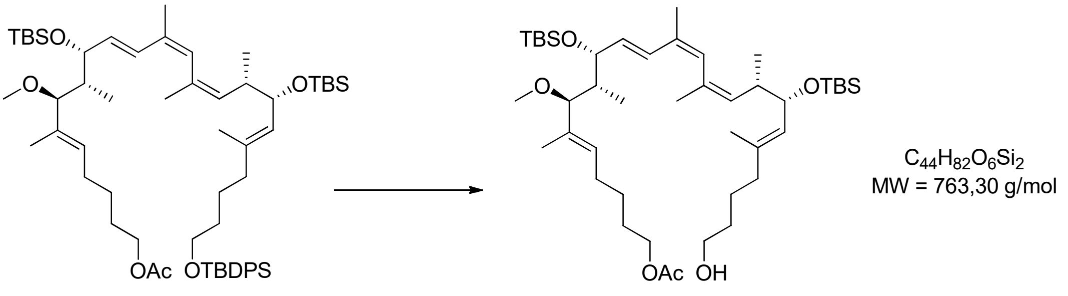

TBAF stock solution : To a solution of TBAF (1 M in THF, 415 µL, 0.42 mmol, 1.00 eq) in THF (9.56 mL) at 0 °C was added AcOH (24 µL, 0.42 mmol, 1.00 eq) resulting in a 41.5 mM solution.

To the neat alcohol **45a** (40 mg, 40 µmol, 1.00 eq) was added the stock solution at 0 °C (1.06 mL, 43.9 µmol, 1.10 eq). The reaction was stirred for 1 h at this temperature and 44 h at room temperature. The reaction was diluted with Et_2O (3 mL) and quenched with a saturated solution of $NaHCO_3$ (2 mL) at 0 °C. After separation of the organic layer, the aqueous layer was extracted with Et_2O (3*5 mL). The organic layers were combined, dried over $MgSO_4$ and evaporated *in vacuo*. The crude product was purified by column chromatography (SiO_2, CH/EtOAc, 10:1 to 5:1) to give the desired product **47a** as a colorless oil (27 mg, 35.4 µmol, 88 %).

$\boldsymbol{R}_f$ = 0.16 (SiO_2, CH/EtOAc, 10:1); $[\boldsymbol{\alpha}]_{\boldsymbol{D}}^{\boldsymbol{20}}$ = + 34.8° (*c* = 0.33, $CHCl_3$, 20 °C); **^{1}H-NMR** (700 MHz, CD_2Cl_2): δ [ppm] = 6.46 – 6.40 (m, 1H), 5.86 (s, 1H), 5.71 (dddd, *J* = 16.0, 7.0, 3.6, 0.7 Hz, 1H), 5.31 – 5.28 (m, 1H), 5.14 – 5.05 (m, 2H), 4.71 – 4.65 (m, 1H), 4.14 – 4.09 (m, 1H), 4.03 (t, *J* = 6.7 Hz, 2H), 3.62 – 3.57 (m, 2H), 3.30 (d, *J* = 10.0 Hz, 1H), 3.10 (d, *J* = 2.0 Hz, 3H), 2.36 (dqd, *J* = 12.6, 6.7, 6.3, 3.6 Hz, 1H), 2.09 (dp, *J* = 18.4, 7.3 Hz, 2H), 2.00 (d, *J* = 3.3 Hz, 6H), 1.85 – 1.83 (m, 3H), 1.82 – 1.77 (m, 3H), 1.64 – 1.61 (m, 2H), 1.59 – 1.57 (m, 3H), 1.47 – 1.40 (m, 8H), 0.91 (s, 9H), 0.90 – 0.88 (m, 3H), 0.85 (s, 9H), 0.64 (dd, *J* = 6.9, 4.4 Hz, 3H), 0.04 (s, 3H), -0.01 (s, 6H), -0.03 – -0.05 (s, 3H); **^{13}C-NMR** (176 MHz, CD_2Cl_2): δ [ppm] = 170.9, 135.2, 134.1, 133.5, 132.6, 132.5, 132.2, 130.2, 129.0, 127.7, 127.2, 88.3, 72.8, 71.8, 64.3, 62.6, 55.1, 42.4, 40.5, 39.3, 32.5, 28.3, 27.1, 25.9, 25.7, 25.6, 24.5, 23.9, 20.7, 20.2, 18.1, 18.0, 16.4, 15.2, 9.8, 8.9, -4.1, -4.7, -5.2, -5.4; **HRMS (ESI+)** calculated for $C_{44}H_{82}O_6Si_2Na^+$ $[M+Na]^+$: 785.5542 found : 785.5564.

Synthesis of analogue 6

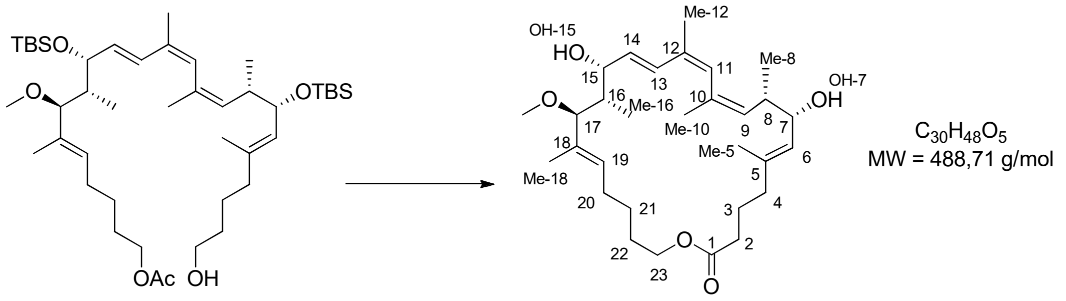

To a solution of DMSO (25 µL, 354 µmol, 10.0 eq), sulfur trioxide pyridine complex (17 mg, 106 µmol, 3.00 eq) and DIEA (25 µL, 141 µmol, 4.00 eq) in DCM (3 mL) at 0 °C was added alcohol **47a**

(27 mg, 35 µmol, 1.00 eq). The solution was stirred at 0 °C for 1.5 h. After this time the reaction was quenched with aqueous saturated solution of $NaHCO_3$ (3 mL) and diluted with DCM (3 mL). After separation of the organic layer, the aqueous layer was extracted 3 times with DCM (5 mL).The organic layers were combined, dried over $MgSO_4$ and evaporated *in vacuo* until 200 mbar. The crude product was then directly used in the next reaction.

The crude aldehyde was diluted in *tert*-butanol (2.5 mL) and 2-methylbut-2-ene (0.15 mL) and cooled at 0 °C. A solution of $NaClO_2$ (10.2 mg, 113 µmol, 3.20 eq), KH_2PO_4 (19 mg, 141 µmol, 4.00 eq) in H_2O (2.5 mL) was added to the reaction mixture and the reaction was stirred for 1 h at room temperature. Saturated aqueous solution of NaCl (4 mL) was added and DCM (4 mL). After separation of the organic layer, the aqueous layer was extracted with DCM (3*5 mL). The organic layers were combined, dried over $MgSO_4$ and evaporated *in vacuo*.

The crude carboxylic acid was diluted in MeOH (2.5 mL) and K_2CO_3 was added (14.7 mg, 106 µmol, 3.00 eq). The reaction was stirred for 3 h at room temperature. The reaction was quenched with $NaHCO_3$ (2 mL) and diluted with DCM (3 mL). After separation of the organic layer, the aqueous layer was extracted with DCM (3*5 mL). The combined organic layers were washed with brine (5 mL), dried over $MgSO_4$ and evaporated *in vacuo.* The crude product was purified by column chromatography (SiO_2, CH/EtOAc, 3:2) to give the precursor for Shiina esterification (5 mg, 7 µmol, 20 % over 3 steps).

MNBA (10.5 mg, 30.6 µmol, 5.00 eq), DMAP (5.2 mg, 43 µmol, 7.00 eq) and 3Å MS were dried for 1 h under high vacuum before DCM was added (3 mL). The seco acid was diluted in DCM (4 mL) and added to the solution over 20 h. Two hours after completion of the addition, the reaction was quenched at 0 °C with pH7 Buffer (3 mL). After separation of the organic layer, the aqueous layer was extracted with DCM (3*5 mL). The combined organic layers were washed with brine (5 mL), dried over $MgSO_4$ and evaporated *in vacuo.* The crude product was purified by column chromatography (SiO_2, CH/EtOAc, 50:1) to give the desired macrolactone (4.3 mg, 6 µmol, 86 %).

The macrolactone (4.3 mg, 6 µmol, 1.00 eq) was then diluted in THF (0.3 mL) and cooled down at 0 °C. Pyridine (0.3 mL) was added followed by HF-pyr (70 % HF, 0.2 mL). After 1 day the reaction was quenched at 0 °C with pH7 Buffer (5 mL). After separation of the organic layer, the aqueous layer was extracted with EtOAc (3*5 mL). The organic layers were washed with a saturated solution of $NaHCO_3$ (15 mL), combined, dried over $MgSO_4$ and evaporated *in vacuo*. The crude product was purified by column chromatography (SiO_2, CH/EtOAc, 10: 1 to 5:1) to afford **6** (1.2 mg, 2.46 µmol, 41 %).

***R*$_f$** = 0.37 (SiO_2, CH/EtOAc, 2:1); $[\alpha]_D^{20}$ = - 10.4° (*c* = 0.1, $CHCl_3$, 20 °C); **^{1}H-NMR** (700 MHz, CD_2Cl_2): δ [ppm] = 6.51 (d, *J* = 15.9 Hz, 1H, **H-13**), 5.71 – 5.67 (m, 1H, **H-14**), 5.66 (s, 1H, **H-11**), 5.38 – 5.35 (m, 1H, **H-19**), 5.18 (d, *J* = 1.2 Hz, 1H, **H-9**), 4.99 (dd, *J* = 9.1, 1.2 Hz, 1H, **H-6**), 4.32 (s, 1H, **H-15**), 4.09 – 4.05 (m, 1H, **1H-23**), 3.99 – 3.93 (m, 2H, **1H-23+H-7**), 3.43 (d, J = 9.9 Hz, 1H, **H-17**), 3.17 (s, 3H, **OMe-17**), 2.27 – 2.18 (m, 5H, **2+20+H-8**), 2.07 – 2.03 (m, 2H, **H-16**), 2.01 – 1.97 (m, 2H, **4**), 1.91 (d, J = 1.4 Hz, 3H, **Me-12**), 1.77 (dd, J = 1.4, 0.8 Hz, 3H, **Me-10**), 1.72 – 1.68 (m, 2H, **3**), 1.65 (d, J = 1.4 Hz, 3H, **Me-5**), 1.59 – 1.55 (m, 2H, **22**), 1.51 (t, J = 1.2 Hz, 3H, **Me-18**), 1.45 (ddd, J = 10.5, 4.4, 2.9 Hz, 2H, **21**), 0.73 (d, J = 6.7 Hz, 3H, **Me-9**), 0.60 (d, J = 7.1 Hz, 3H, **Me-16**); **^{13}C-NMR** (176 MHz, CD_2Cl_2): δ [ppm] = 173.3 (**C-1**), 138.4 (**C-5**), 135.0, 132.9, 132.2, 131.9 (**C-14**)130.9 (**C-9**), 130.6 (**C-19**), 128.8 (**C-11**), 18.7 (**C-13**) 126.7 (**C-6**), 90.1, (**C-17**), 73.0 (**C-15**), 71.7 (**C-7**), 64.1 (**C-23**), 55.4 (**OMe-17**), 40.7 (**C-8**), 40.4 (**C-16**), 38.8 (**C-4**), 34.1 (**C-2**), 27.8 (**C-22**), 26.6 (**C-20**), 25.9 (**C-21**), 24.3 (**Me-10**), 23.0 (**C-3**), 19.7 (**Me-12**),

17.0 (**Me-8**), 16.7 (**Me-5**), 12.1 (**Me-16**), 9.8 (**Me-18**); **HRMS (ESI+)** calculated for $C_{30}H_{48}O_5Na^+$ $[M+Na]^+$: 511.3394, found : 511.3394.

2.6 Completion of analogue 7

Synthesis of methyl ether 46b

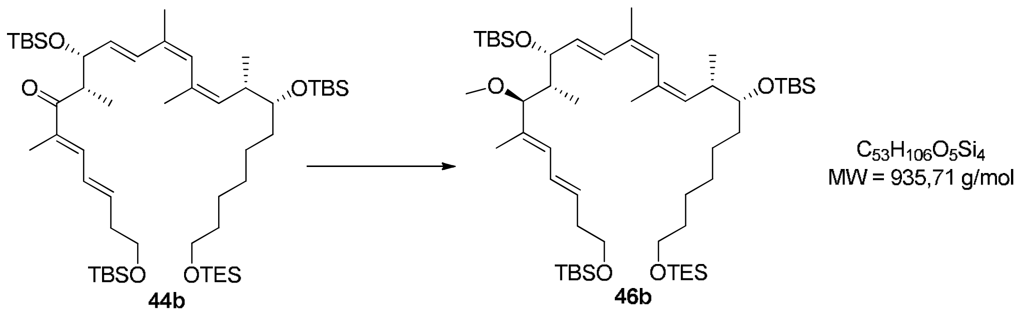

To a solution of ketone **44b** (78 mg, 85 µmol, 1.00 eq) in MeOH (3.5 mL) and THF (1 mL) at 0 °C was added $NaBH_4$ (12.8 mg, 340 µmol, 4.00 eq) and the solution was warmed up to room temperature. After 2 h, more $NaBH_4$ (12.8 mg, 340 µmol, 4.00 eq) was added. After 3 h, the reaction was diluted with EtOAc (5 mL) and quenched carefully with NH_4Cl (4 mL) at 0 °C. After separation of the organic layer, the aqueous layer was extracted with EtOAc (3*10 mL). The organic layers were combined, dried over $MgSO_4$ and evaporated *in vacuo*. The crude product was purified by column chromatography (SiO_2, CH/EtOAc, 50:1) to give the alcohol as a thick oil (67 mg, 73 µmol, 86 %, *dr* > 10:1).

To a solution of alcohol (67 mg, 73 µmol, 1.00 eq) in DCM (4 mL) at 0 °C was added proton sponge (86 mg, 0.4 mmol, 5.50 eq) followed by MeO_3BF_4 (54 mg, 0.63 mmol, 5.00 eq). The reaction was stirred for 5 h at 0 °C. After this time, a saturated solution of $NaHCO_3$ (2.5 mL) was added at 0 °C. After separation of the organic layer, the aqueous layer was extracted with DCM (3*5 mL). The organic layers were combined, dried over $MgSO_4$ and evaporated *in vacuo*. The crude product was purified by column chromatography (SiO_2, CH/EtOAc, 80:1) to give the methyl ether **46b** (57 mg, 61 µmol, 84 %).

***R*f** = 0.69 (SiO_2, CH/EtOAc, 10:1); $[\alpha]_D^{20}$ = - 7.2° (*c* = 0.25, $CHCl_3$); **^{1}H-NMR** (700 MHz, $CDCl_3$): δ [ppm] = 6.41 – 6.37 (m, 1H), 6.33 (ddt, *J* = 15.0, 10.6, 1.3 Hz, 1H), 5.92 – 5.88 (m, 1H), 5.80 (s, 1H), 5.71 – 5.64 (m, 2H), 5.13 – 5.10 (m, 1H), 4.69 (dt, *J* = 6.9, 1.6 Hz, 1H), 3.67 (t, *J* = 6.7 Hz, 2H), 3.59 (t, *J* = 6.8 Hz, 3H), 3.40 (td, *J* = 8.7, 7.9, 4.7 Hz, 1H), 3.34 (d, *J* = 10.0 Hz, 1H), 3.13 (d, *J* = 9.8 Hz, 3H), 2.40 (tt, *J* = 10.8, 6.5 Hz, 1H), 2.36 – 2.32 (m, 2H), 1.83 (d, *J* = 1.4 Hz, 3H), 1.78 (q, *J* = 1.8, 1.1 Hz, 3H), 1.60 (s, 1H), 1.59 – 1.57 (m, 3H), 1.53 – 1.49 (m, 3H), 1.34 – 1.27 (m, 7H), 1.15 (tt, *J* = 9.8, 5.8 Hz, 1H), 0.96 (t, *J* = 7.9 Hz, 12H), 0.92 (d, *J* = 6.4 Hz, 9H), 0.89 (d, *J* = 2.9 Hz, 13H), 0.87 – 0.86 (m, 9H), 0.63 (d, *J* = 6.9 Hz, 2H), 0.60 (t, *J* = 8.0 Hz, 6H), 0.05 (s, 6H), 0.04 (s, 3H), -0.01 (s, 3H), --0.02 (s, 3H), -0.03 (s, 3H); **^{13}C-NMR** (176 MHz, $CDCl_3$): δ [ppm] = 134.2, 134.2, 132.8, 132.6, 131.7, 130.6, 129.7, 129.0, 127.9, 127.5, 88.3, 77.2, 77.2, 77.0, 76.8, 75.8, 71.6, 63.0, 62.9, 55.6, 42.7, 38.7, 36.6, 33.0, 32.7, 29.7, 29.7, 26.3, 26.0, 26.0, 26.0, 26.0, 25.9, 25.9, 24.9, 20.4, 18.4, 18.2, 18.1, 14.8, 10.5, 9.0, 6.8, 4.5, -3.8, -4.5, -4.6, -5.1, -5.2, -5.2; **HRMS (ESI+)** calculated for $C_{53}H_{106}O_5Si_4Na^+$ $[M+Na]^+$: 934.7117, found 934.7117.

Synthesis of alcohol 48b

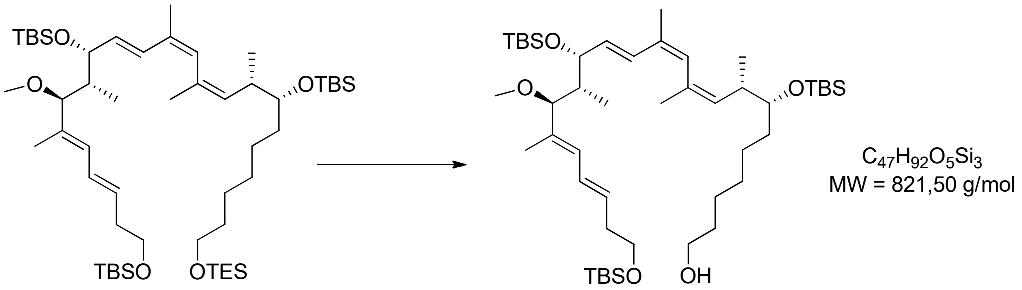

46b **48b**

To a solution of **46b** (60 mg, 64.1 µmol, 1.00 eq) in MeOH (10 mL) was added K_2CO_3 (266 mg, 1.9 mmol, 30.0 eq) at 0 °C. The solution was warmed up to room temperature and stirred overnight. The reaction was quenched with a saturated solution of $NaHCO_3$ (10 mL) and diluted with EtOAc (10 mL). After separation of the organic layer, the aqueous layer was extracted with EtOAc (3*10 mL). The combined organic layers were dried over $MgSO_4$ and evaporated *in vacuo.* The crude product was purified by column chromatography (SiO_2, CH/EtOAc, 10:1) to give the alcohol **48b** (49.7 mg, 60.5 µmol, 94 %).

***R*f** = 0.09 (SiO_2, CH/EtOAc, 10:1); $[\alpha]_D^{20}$ = - 11.6° (*c* = 0.32, $CHCl_3$); **^{1}H-NMR** (700 MHz, CD_2Cl_2): δ [ppm] = 6.40 – 6.36 (m, 1H), 6.36 – 6.32 (m, 1H), 5.94 – 5.90 (m, 1H), 5.81 (s, 1H), 5.74 – 5.65 (m, 2H), 5.16 – 5.12 (m, 1H), 4.70 (dt, *J* = 6.9, 1.5 Hz, 1H), 3.66 (t, *J* = 6.6 Hz, 2H), 3.58 (td, *J* = 6.7, 5.3 Hz, 2H), 3.43 (ddd, *J* = 9.8, 7.4, 4.3 Hz, 1H), 3.34 (d, *J* = 9.9 Hz, 1H), 3.10 (s, 3H), 2.41 (dqd, *J* = 10.6, 6.8, 3.7 Hz, 1H), 2.34 – 2.29 (m, 2H), 1.84 (d, *J* = 1.4 Hz, 3H), 1.80 – 1.77 (m, 3H), 1.60 – 1.58 (m, 1H), 1.58 – 1.56 (m, 3H), 1.52 – 1.49 (m, 2H), 1.34 – 1.27 (m, 8H), 0.92 (d, *J* = 6.5 Hz, 9H), 0.89 (d, *J* = 4.0 Hz, 12H), 0.86 (s, 9H), 0.63 – 0.61 (m, 3H), 0.06 (s, 3H), 0.05 (s, 6H), -0.01 (s, 3H), -0.01 (s, 3H), -0.02 (s, 3H); **^{13}C-NMR** (176 MHz, CD_2Cl_2): δ [ppm] = 134.3, 134.2, 132.8, 132.7, 131.6, 130.7, 129.6, 128.9, 127.8, 127.5, 88.2, 75.8, 71.7, 62.8, 62.8, 62.8, 55.3, 53.8, 53.7, 53.6, 53.6, 53.5, 53.4, 53.3, 53.1, 42.7, 38.6, 36.5, 32.9, 32.6, 29.7, 29.6, 26.3, 25.8, 25.8, 25.8, 25.7, 25.7, 25.7, 25.7, 25.7, 25.7, 24.5, 20.0, 18.2, 18.1, 18.0, 18.0, 14.5, 10.3, 8.8, -4.1, -4.8, -4.9, -5.3, -5.6, -5.6; **HRMS** calculated for $C_{47}H_{96}O_5Si_3N^+$ $[M+NH_4]^+$: 838.6591, found 838.6591.

Synthesis of analogue 7

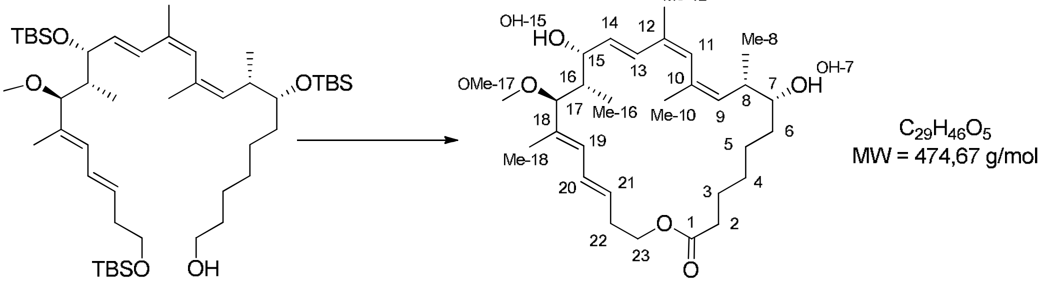

48b **7**

To a solution of DMSO (41 µL, 523 µmol, 10.0 eq), sulfur trioxide pyridine complex (25 mg, 157 µmol, 3.00 eq) and DIEA (37 µL, 209 µmol, 4.00 eq) in DCM (4 mL) at 0 °C was added alcohol **48b** (43 mg, 52.3 µmol, 1.00 eq). The solution was stirred at 0 °C for 1.5 h. After this time the reaction

was quenched with aqueous saturated solution of $NaHCO_3$ (3 mL) and diluted with DCM (3 mL). After separation of the organic layer, the aqueous layer was extracted with DCM (3*5 mL).The organic layers were combined, dried over $MgSO_4$ and evaporated *in vacuo*.

The crude aldehyde was diluted in *tert*-butanol (2 mL) and 2-methylbut-2-ene (0.2 mL) and cooled at 0 °C. A solution of $NaClO_2$ (15 mg, 167 µmol, 3.20 eq), KH_2PO_4 (28.5 mg, 209 µmol, 4.00 eq) in H_2O (2 mL) was added to the solution and the reaction was stirred for 1 h at room temperature. Saturated aqueous solution of NaCl (4 mL) was added followed by DCM (4 mL). After separation of the organic layer, the aqueous layer was extracted with DCM (3*5 mL). The organic layers were combined, dried over $MgSO_4$ and evaporated *in vacuo*. The crude product was purified by column chromatography (SiO_2, CH/EtOAc, 10:1) to give the carboxylic acid (33.4 mg, 40 µmol, 76 % over two steps).

HF-pyridine stock solution: To a solution of THF (1.3 mL) and pyridine (0.75 mL) at 0 °C was added HF-pyr (0.25 mL, 70 % HF).

To a solution of carboxylic acid (33 mg, 40 µmol, 1.00 eq) in THF (1 mL) at 0 °C was added HF-pyr stock solution (0.5 mL). The reaction was then stirred for 6 h at 0 °C. The reaction was quenched with a saturated solution of $NaHCO_3$ (10 mL) and diluted with DCM (3 mL). After separation of the organic layer, the aqueous layer was extracted with DCM (3*5 mL). The combined organic layers were washed with brine (5 mL), dried over $MgSO_4$ and evaporated *in vacuo.* The crude product was purified by column chromatography (SiO_2, CH/EtOAc, 3:2) to give the precursor for Shiina esterification (12.1 mg, 17 µmol, 42 %).

MNBA (29 mg, 84 µmol, 5.00 eq), DMAP (14 mg, 117 µmol, 7.00 eq) and 3Å MS were dried for 1 h under high vacuum before DCM was added (6 mL). The seco acid was diluted in DCM (8 mL) and added to the solution over 20 h. Two hours after completion of the addition, the reaction was quenched at 0 °C with pH7 Buffer (3 mL). After separation of the organic layer, the aqueous layer was extracted with DCM (3*5 mL). The combined organic layers were washed with brine (5 mL), dried over $MgSO_4$ and evaporated *in vacuo.* The crude product was purified by column chromatography (SiO_2, CH/EtOAc, 50:1) to give the desired macrolactone (9.8 mg, 14 µmol, 83 %).

The macrolactone (9.2 mg, 14 µmol, 1 eq) was diluted in THF (0.5 mL) and cooled down at 0 °C. Pyridine (0.5 mL) was added followed by HF-pyr (70 % HF, 0.5 mL). After two days at room temperature, the reaction was quenched at 0 °C with Buffer pH7 (5 mL). After separation of the organic layer, the aqueous layer was extracted with EtOAc (3*5 mL). The organic layers were combined, dried over $MgSO_4$ and evaporated *in vacuo*. The crude product was purified by column chromatography (SiO_2, CH/EtOAc, 10: 1 to 5:1) to give **221** as a white solid (1.6 mg, 3.4 µmol, 24 %).

R_f = 0.28 (SiO_2, CH/EtOAc, 2:1); $[\alpha]_D^{20}$ = - 24.7° (*c* = 0.15, $CHCl_3$); <u>^{1}H-NMR</u> (700 MHz, CD_2Cl_2): δ [ppm] = 6.53 (dd, *J* = 16.0, 4.0 Hz, 1H, **H-13**), 6.35 (dd, *J* = 15.3, 10.7 Hz, 1H, **H-20**), 5.93 (d, *J* = 10.8 Hz, 1H, **H-19**), 5.69 (dd, *J* = 16.0, 4.9 Hz, 1H, **H-14**), 5.64 (s, 1H, **H-11**), 5.60 (ddd, *J* = 13.8, 9.6, 5.3 Hz, 1H, **H-21**), 5.16 (d, *J* = 9.9 Hz, 1H, **H-9**), 4.39 (s, 1H, **H-15**), 4.28 (ddd, *J* = 10.8, 8.7, 4.4 Hz, 1H, **1H-23**), 4.04 (ddd, *J* = 10.1, 6.8, 4.6 Hz, 1H, **1H-23**), 3.53 (d, *J* = 9.2 Hz, 1H, **H-17**), 3.24 (td, *J* = 8.9, 2.4 Hz, 1H, **H-8**), 3.18 (s, 3H, **OMe-17**), 2.48 – 2.43 (m, 2H, **22**), 2.26 – 2.15 (m, 3H, **2+H-8**), 1.89 (d, *J* = 2.7 Hz, 3H, **Me-12**), 1.87 – 1.84 (m, 1H, **H-16**), 1.76 (d, *J* = 3.1 Hz, 3H, **Me-10**), 1.64 (d, *J* = 2.9 Hz, 3H, **Me-18**), 1.51 – 1.42 (m, 4H, **3+4**), 1.25 – 1.13 (m, 4H, **5+6**), 0.80 (d, *J* = 6.7 Hz, 3H, **Me-8**), 0.55 (d, *J* = 7.2 Hz, 3H, **Me-16**); <u>^{13}C-NMR</u> (176 MHz, CD_2Cl_2): δ [ppm] = δ 173.6(**C-1**), 134.4 (**C-10**), 133.8 (**C-18**), 132.3 (**C-14**), 132.0 (**C-12**), 131.4 (**C-9**), 130.9 (**C-21**), 129.2 (**C-19**), 128.5 (**C-11**), 128.1 (**C-20**), 128.0 (**C-13**), 89.3 (**C-17**), 76.3 (**C-7**),73.2 (**C-15**), 62.9 (**C-23**), 55.9 (**OMe-17**), 40.9 (**C-16**), 40.2 (**C-8**), 35.2 (**C-2**), 33.8 (**C-6**), 32.3 (**C-22**),

29.9 (**C-4**), 26.1 (**C-3**), 25.5 (**C-5**), 24.5 (**Me-10**), 19.8 (**Me-12**), 17.3 (**Me-8**),11.4 (**Me-16**) 10.5 (**Me-18**); **HRMS (ESI+)** calculated for $C_{29}H_{46}O_5Na^+$ [M+Na]$^+$: 497.3237, found : 497.3237.

2.7 Completion of analogue 8

Synthesis of alcohol methyl ether 45b

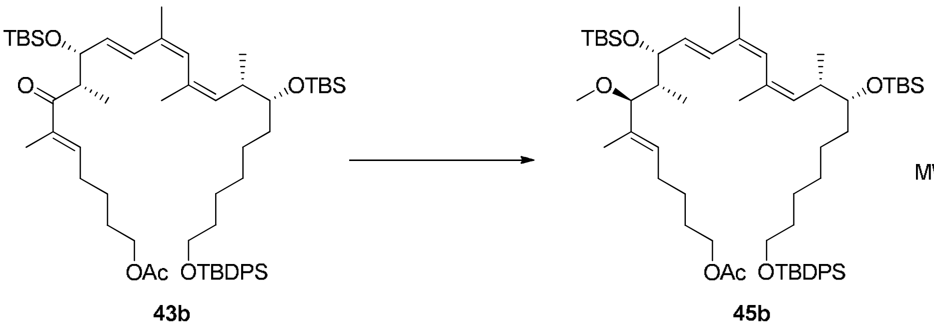

$C_{59}H_{100}O_6Si_3$
MW = 989,69 g/mol

To a solution of ketone **43b** (105 mg, 108 µmol, 1.00 eq) in MeOH (3 mL) and THF (1.5 mL) at 0 °C was added $NaBH_4$ (8 mg, 216 µmol, 2.00 eq) and the solution was warmed up to room temperature. After 2 h, more $NaBH_4$ (8 mg, 216 µmol, 2.00 eq) was added. After 3 h, the reaction was diluted with EtOAc (5 mL) and quenched with NH_4Cl (4 mL) at 0 °C. After separation of the organic layer, the aqueous layer was extracted with EtOAc (3* 10 mL). The organic layers were combined, dried over $MgSO_4$ and evaporated *in vacuo*. The crude product was purified by column chromatography (SiO_2, CH/EtOAc, 60:1 to 30:1) to give alcohol as a thick oil (73 mg, 75 µmol, 70 %, *dr* = 10:1,).

To a solution of alcohol (73 mg, 74 µmol, 1.00 eq) in DCM (4 mL) at 0 °C was added proton sponge (88 mg, 0.41 mmol, 5.50 eq) followed by MeO_3BF_4 (55 mg, 0.37 mmol, 5.00 eq). The reaction was stirred for 3 h at 0 °C. After this time, a saturated solution of $NaHCO_3$ (4 mL) was added at 0 °C. After separation of the organic layer, the aqueous layer was extracted with DCM (3*5 mL). The organic layers were combined, dried over $MgSO_4$ and evaporated *in vacuo*. The crude product was purified by column chromatography (SiO_2, CH/EtOAc, 60:1) to give methyl ether **45b** (60 mg, 61 µmol, 82 %).

***R*$_f$** = 0.44 (SiO_2, CH/EtOAc, 10:1); $[\alpha]_D^{20}$ = + 4.5° (*c* = 0.33, $CHCl_3$); **^{1}H-NMR** (500 MHz, CD_2Cl_2): δ [ppm] = 7.68 – 7.65 (m, 4H), 7.43 – 7.35 (m, 6H), 6.39 (d, *J* = 15.9 Hz, 1H), 5.80 (s, 1H), 5.69 (dd, *J* = 15.9, 6.8 Hz, 1H), 5.29 (t, *J* = 6.6 Hz, 1H), 5.13 – 5.10 (m, 1H), 4.68 (d, *J* = 6.8 Hz, 1H), 4.07 (t, *J* = 6.6 Hz, 2H), 3.65 (t, *J* = 6.5 Hz, 2H), 3.41 – 3.37 (m, 1H), 3.30 (d, *J* = 10.0 Hz, 1H), 3.11 (s, 3H), 2.42 – 2.38 (m, 1H), 2.09 (dt, *J* = 13.9, 6.9 Hz, 2H), 2.05 (s, 3H), 1.83 (d, *J* = 1.2 Hz, 3H), 1.78 (s, 3H), 1.64 (dt, *J* = 14.7, 6.6 Hz, 2H), 1.59-1.55 (m, 2H), 1.47 – 1.42 (m, 5H), 1.36-1.20 (m, 8H) 1.04 (s, 9H), 0.92 – 0.90 (s, 9H), 0.89 – 0.87 (m, 3H), 0.87 – 0.86 (m, 9H), 0.64 – 0.61 (d, *J* = 6.9 Hz 2H), 0.03 (d, *J* = 2.5 Hz, 3H), -0.01-(-0.02) (s, 6H), -0.03 (s, 3H); **^{13}C-NMR** (125 MHz, CD_2Cl_2): δ [ppm] = 171.2, 135.6, 134.3, 134.2, 133.5, 132.8, 132.6, 131.7, 130.1, 129.5, 128.9, 127.6, 127.5, 88.4, 75.8, 71.6, 64.4, 64.0, 55.4, 42.4, 38.6, 32.7, 32.6, 29.7, 28.3, 27.2, 26.9, 26.3, 25.9, 24.9, 21.0, 20.4, 19.3, 18.2, 18.1, 14.8, 10.0, 9.1, -3.8, -4.5, -4.6, -5.1; **HRMS (ESI+)** calculated for $C_{59}H_{104}O_6Si_3N^+$ [M+NH$_4$]$^+$: 1006.7166 found : 1006.7166.

Synthesis of alcohol 47b

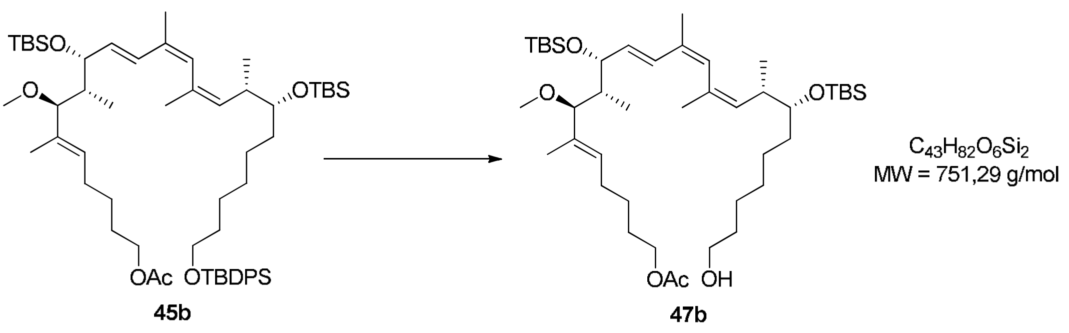

TBAF stock solution : To a solution of TBAF (1 M in THF, 415 µL, 0.42 mmol, 1.00 eq) in THF (9.56 mL) at 0 °C was added AcOH (24 µL, 0.42 mmol, 1.00 eq) resulting in a 41.5 mM solution.

To the alcohol **45b** (58 mg, 59 µmol, 1.00 eq) was added the stock solution at 0 °C (1.6 mL, 65 µmol, 1.10 eq). The reaction was stirred for 1 h at this temperature then 30 h at room temperature. The reaction was diluted with Et_2O (3 mL) and quenched with $NaHCO_3$ (2 mL) at 0 °C. After separation of the organic layer, the aqueous layer was extracted with Et_2O (3*5 mL). The organic layers were combined, dried over $MgSO_4$ and evaporated *in vacuo*. The crude product was purified by column chromatography (SiO_2, CH/EtOAc, 20:1 to 10: 1) to give the desired product **47b** as a colorless oil (41 mg, 54 µmol, 92 %).

$\boldsymbol{R}_f$ = 0.13 (SiO_2, CH/EtOAc, 10:1); $[\boldsymbol{\alpha}]_{\boldsymbol{D}}^{\boldsymbol{20}}$ = - 0.7° (*c* = 0.22, $CHCl_3$); **^{1}H-NMR** (700 MHz, CD_2Cl_2): δ [ppm] = 6.46 – 6.40 (m, 1H), 5.86 (s, 1H), 5.71 (dddd, *J* = 16.0, 7.0, 3.6, 0.7 Hz, 1H), 5.31 – 5.28 (m, 1H), 5.14 – 5.05 (m, 2H), 4.71 – 4.65 (m, 1H), 4.14 – 4.09 (m, 1H), 4.03 (t, *J* = 6.7 Hz, 2H), 3.62 – 3.57 (m, 2H), 3.30 (d, *J* = 10.0 Hz, 1H), 3.10 (d, *J* = 2.0 Hz, 3H), 2.36 (dqd, *J* = 12.6, 6.7, 6.3, 3.6 Hz, 1H), 2.09 (dp, *J* = 18.4, 7.3 Hz, 2H), 2.00 (d, *J* = 3.3 Hz, 6H), 1.85 – 1.83 (m, 3H), 1.82 – 1.77 (m, 3H), 1.64 – 1.61 (m, 2H), 1.59 – 1.57 (m, 3H), 1.44 (d, *J* = 0.8 Hz, 9H), 0.91 (s, 9H), 0.90 – 0.88 (m, 3H), 0.85 (s, 9H), 0.64 (dd, *J* = 6.9, 4.4 Hz, 3H), 0.05 (s, 3H), -0.01 (s, 6H), -0.04 (s, 3H); **^{13}C-NMR** (176 MHz, $CDCl_3$): δ [ppm] = 170.9, 134.4, 133.5, 132.8, 132.7, 131.6, 130.2, 128.9, 127.4, 88.3, 75.8, 71.7, 64.3, 62.8, 55.1, 42.5, 38.6, 32.9, 32.6, 29.6, 28.3, 27.1, 26.3, 25.9, 25.8, 25.7, 25.6, 25.4, 20.7, 20.0, 18.1, 17.9, 14.6, 9.7, 8.9, -4.1, -4.8, -4.9, -5.3; **HRMS (ESI+)** calculated for $C_{43}H_{82}O_6Si_2Na^+$ $[M+Na]^+$: 773.5542, found : 773.5542.

Synthesis of analogue 8

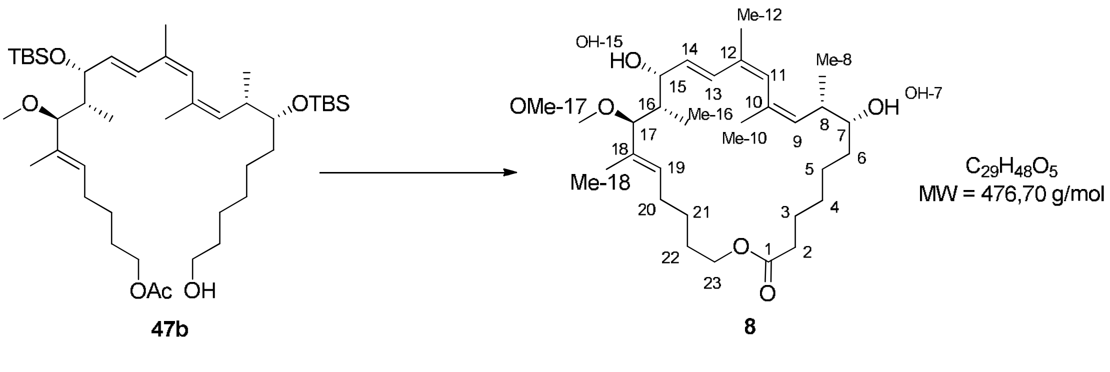

To a solution of DMSO (20 µL, 208 µmol, 10.0 eq), sulfur trioxide pyridine complex (13 mg, 84 µmol, 3.00 eq) and DIEA (20 µL, 112 µmol, 4.00 eq) in DCM (3 mL) at 0 °C was added alcohol **47b** (21 mg, 28 µmol, 1.00 eq). The solution was stirred at 0 °C for 1.5 h. After this time the reaction was quenched with a saturated solution of $NaHCO_3$ (3 mL) and diluted with DCM (3 mL). After separation of the organic layer, the aqueous layer was extracted with DCM (3*5 mL).The organic layers were combined, dried over $MgSO_4$ and evaporated *in vacuo* until 200 mbar. The crude product was then directly used in the next reaction.

The crude aldehyde was diluted in *tert*-butanol (2 mL) and 2-methylbut-2-ene (0.1 mL) and cooled at 0 °C. $NaClO_2$ (8 mg, 89 µmol, 3.20 eq), KH_2PO_4 (15 mg, 111 µmol, 4.00 eq) in H_2O (2 mL) were added to the solution and the reaction was stirred for 1 h at room temperature. Saturated aqueous solution of NaCl (2 mL) was added and diluted with DCM (2 mL). After the separation of the organic layer, the aqueous layer was extracted with DCM (3*5 mL), the organic layers were combined, dried over $MgSO_4$ and evaporated *in vacuo*.

The crude carboxylic acid was diluted in MeOH (2 mL) and K_2CO_3 was added (11.2 mg, 84 µmol, 3.00 eq). The reaction was stirred for 3 h at room temperature. The reaction was quenched with a saturated solution of $NaHCO_3$ (2 mL) and diluted with DCM (3 mL). After separation of the organic layer, the aqueous layer was extracted with DCM (3*5 mL). The combined organic layers were washed with brine (5 mL), dried over $MgSO_4$ and evaporated *in vacuo.* The crude product was purified by column chromatography (SiO_2, CH/EtOAc, 3:2) to give the precursor for Shiina esterification (9.5 mg, 13 µmol, 46 % over three steps).

MNBA (22.6 mg, 66 µmol, 5.00 eq), DMAP (11 mg, 92 µmol, 7.00 eq) and 3Å MS were dried for 1 h under high vacuum before DCM was added (5 mL). The seco acid was diluted in DCM (7 mL) and added to the solution over 20 h. Two hours after completion of the addition, the reaction was quenched at 0 °C with buffer pH7 (3 mL). After separation of the organic layer, the aqueous layer was extracted with DCM (3*5 mL). The combined organic layers were washed with brine (5 mL), dried over $MgSO_4$ and evaporated *in vacuo.* The crude product was purified by column chromatography (SiO_2, CH/EtOAc, 50:1) to give the desired macrolactone (7 mg, 10 µmol, 77 %).

The macrolactone (10.2 mg, 14.2 µmol, 1.00 eq) was then diluted in THF (0.5 mL) and cooled down at 0 °C. Pyridine (0.5 mL) was added followed by HF-pyr (70 % HF, 0.3 mL). After 1 day the reaction was quenched at 0°C with buffer pH7 (5 mL). After separation of the organic layer, the aqueous layer was extracted with EtOAc (3*5 mL). The organic layers were combined, dried over $MgSO_4$ and evaporated *in vacuo*. The crude product was purified by column chromatography (SiO_2, CH/EtOAc, 10: 1 to 5:1) to give **8** as a thick colorless oil (2.1 mg, 4.4 µmol, 31 %).

***R*$_f$** = 0.44 (SiO_2, CH/EtOAc, 2:1); $[\alpha]_D^{20}$ = - 8.0° (*c* = 0.20, $CHCl_3$); **^{1}H-NMR** (700 MHz, CD_2Cl_2): δ [ppm] = 6.59 (d, *J* = 16.0 Hz, 1H, **H-13**)., 5.71 (ddd, *J* = 15.9, 4.6, 0.7 Hz, 1H, **H-14**), 5.64 (s, 1H, **H-11**), 5.37 (ddd, *J* = 9.3, 5.4, 1.6 Hz, 1H, **H-19**), 5.18 (dq, *J* = 9.9, 1.3 Hz, 1H, **H-9**), 4.46 (s, 1H, **H-15**), 4.11 (dt, *J* = 10.8, 6.0 Hz, 1H, **1H-23**), 3.99 – 3.95 (m, 1H, **1H-23**), 3.42 (d, *J* = 10.0 Hz, 1H, **H-17**), 3.24 (td, *J* = 8.8, 8.3, 2.2 Hz, 1H, **H-7**), 3.16 (s, 3H, **OMe-17**), 2.30 (dt, *J* = 14.6, 6.8 Hz, 1H, **H-8**), 2.24 – 2.18 (m, 3H, **2+1H-20**), 2.05 (dtdd, *J* = 14.1, 6.4, 5.1, 1.3 Hz, 1H, **1H-20**), 1.89 (d, *J* = 1.4 Hz, 3H, **Me-12**), 1.87 – 1.83 (m, 1H, **H-16**), 1.76 (dd, *J* = 1.5, 0.8 Hz, 3H, **Me-10**), 1.62 – 1.56 (m, 4H, **3+4**), 1.51 (t, *J* = 1.2 Hz, 3H, **Me-18**), 1.27 – 1.18 (m, 8H, **5+6+21+22**), 0.81 (d, *J* = 6.7 Hz, 3H, **Me-8**), 0.57 (d, *J* = 7.1 Hz, 3H, **Me-16**); **^{13}C-NMR** (176 MHz, CD_2Cl_2): δ [ppm] = 173.3 (**C-1**), 134.3 (Cq), 132.9 (Cq), 132.6 (**C-14**), 132.0 (Cq), 131.4 (**C-9**), 130.6 (**C-19**), 128.4 (**C-11**), 127.9 (**C-13**), 89.6 (C-**17**), 76.7 (**C-7**), 72.8 (**C-15**), 64.0 (C-**23**), 55.4 (**OMe-17**), 40.5 (**C-16**), 39.9 (**C-8**), 35.1 (**C-2**), 34.0 (**C-6**), 29.6 (**C-22**), 28.1 (**C-4**), 26.9 (**C-5**), 26.8 (**C-20**), 26.0

(**C-3**), 25.5 (C-**21**), 24.4 (**Me-10**), 19.7 (**Me-12**), 17.5 (**Me-8**), 11.1 (**Me-16**), 9.7 (**Me-18**); **HRMS (ESI+)** calculated for $C_{29}H_{48}O_5Na^+$ $[M+Na]^+$: 499.3394, found : 499.3394.

2.8 Protecting group strategy

- *2.8.1 Ester functionality as R_2*

Synthesis of aldehyde 61

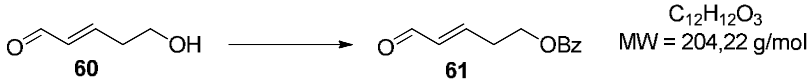

To a solution of alcohol **60** (16 mg, 0.16 mmol, 1.00 eq) in DCM (0.8 mL) at 0 °C was added BzCl (29 µL, 0.25 mmol, 1.50 eq), NEt_3 (44 µL, 0.33 mmol, 2.00 eq) and DMAP (2 mg, 0.02 mmol, 0.10 eq). The solution was stirred for 30 min before begin diluted with DCM (2 mL) and quenched with a solution of saturated aqueous NH_4Cl (2 mL). After separation of the organic layer, the aqueous layer was extracted with DCM (3*5 mL). The organic layers were combined, dried over $MgSO_4$ and evaporated *in vacuo*. The crude product was purified by column chromatography (SiO_2, CH/EtOAc, 3:1) to obtain aldehyde **61** as a colorless oil (17 mg, 83 µmol, 52 %).

***R*$_f$** = 0.50 (SiO_2, CH/EtOAc, 1:1); **^{1}H-NMR** (400 MHz, $CDCl_3$): δ [ppm] = 9.55 (d, *J* = 7.7 Hz, 1H), 8.11 – 7.99 (m, 2H), 7.64 – 7.54 (m, 1H), 7.45 (ddt, *J* = 8.3, 6.7, 1.2 Hz, 2H), 6.90 (dt, *J* = 15.7, 6.7 Hz, 1H), 6.33 – 6.22 (m, 1H), 4.51 (t, *J* = 6.3 Hz, 2H), 2.83 (qd, *J* = 6.4, 1.5 Hz, 2H); **^{13}C-NMR** (76 MHz, $CDCl_3$): δ [ppm] = 193.6, 166.4, 153:1, 134.8, 133.2, 129.8, 129.6, 128.5, 62.4, 32.1. The mass was not found was common MS techniques (EI, ESI, APCI, MALDI).

Synthesis of ketone 62

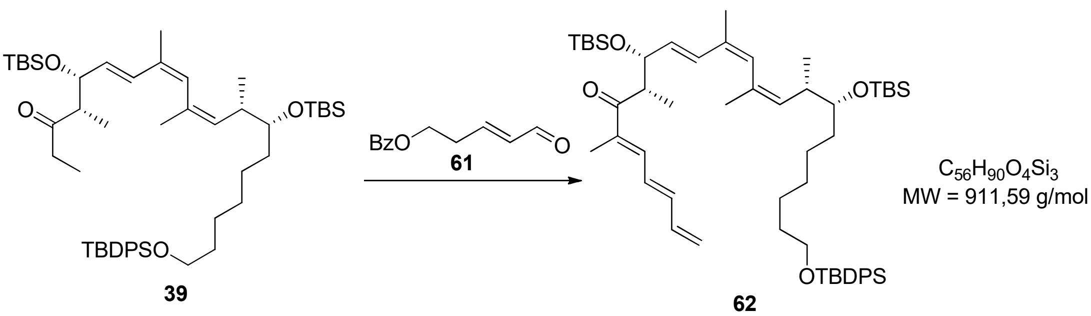

Stock solution: To a solution of TMP (24 µL, 0.14 mmol, 4.00 eq) in THF (0.5 mL) at -78 °C was added *n*-BuLi (1.6 M in hexane, 88 µL, 0.14 mmol, 4.00 eq). The yellow solution was then stirred for 30min at 0 °C.

Ketone **39** (30 mg, 35 µmol, 1.00 eq) dried 30 min over 3Å MS was diluted in THF (0.8 mL). The solution was then cooled down at -78 °C, the LiTMP solution (0.3 mL, 71 µmol, 2.00 eq) was added dropwise.The mixture was stirred for 45 min at -78 °C and 20 min at – 50 °C. The enolate solution was then cooled down at -78 °C and aldehyde **61** (11 mg, 53 µmol, 1.50 eq) in THF (0.4 mL) was added dropwise. After 1.5 h, the reaction mixture was warmed up to 0 °C, diluted with Et_2O (2 mL) and quenched with a saturated $NaHCO_3$ solution (2 mL). After separation of the organic layer, the aqueous layer was extracted with Et_2O (3*3 mL). The organic layers were combined, dried over $MgSO_4$ and evaporated *in vacuo*. The crude product was purified by column chromatography (SiO_2, CH/EtOAc, 20:1 to 10:1) to give a mixture of diastereoisomers (25 mg, 23.8 µmol, 68 %) and starting material ketone (8 mg, 9.4 µmol, 27 %, 95 % brsm).

The mixture of diastereoisomers (25 mg, 23.8 µmol, 1.00 eq) was diluted in THF (2 mL), DMAP (30 mg, 237 µmol, 10.0 eq) and Ac_2O (20 µL, 214 µmol, 9.00 eq) were added at 0 °C. After 1 h,

DBU (71 µL, 475 µmol, 20.0 eq) was added. The reaction mixture was stirred overnight at room temperature. The reaction was quenched with buffer pH7 (5 mL), the aqueous layer was extracted with Et_2O (3*5 mL). The organic layers were combined, dried over $MgSO_4$ and evaporated *in vacuo*. The crude product was purified by column chromatography (SiO_2, CH/EtOAc, 20:1) to give the double eliminated product **62** as a colorless oil (18 mg, 19.7 µmol, 56 % over three steps).

***R*$_f$** = 0.87 (SiO_2, CH/EtOAc, 10:1); $[\alpha]_D^{20}$ = + 9.8° (*c* = 0.56, $CHCl_3$); **^{1}H-NMR** (700 MHz, $CDCl_3$): δ [ppm] = 7.68 – 7.65 (m, 4H), 7.41 (ddt, *J* = 6.4, 4.8, 1.4 Hz, 2H), 7.37 (dd, *J* = 11.1, 4.3 Hz, 6H), 7.04 – 7.01 (m, 1H), 6.60 – 6.52 (m, 1H), 6.51 – 6.45 (m, 2H), 6.33 (d, *J* = 10.6 Hz, 1H), 5.85 (s, 1H), 5.54 (dd, *J* = 10.1, 5.6 Hz, 1H), 5.42 – 5.39 (m, 1H), 5.30 (dd, *J* = 7.8, 3.0 Hz, 1H), 5.16 – 5.13 (m, 1H), 4.27 (t, *J* = 7.5 Hz, 1H), 3.66 – 3.63 (m, 2H), 3.44 – 3.36 (m, 2H), 2.39 – 2.34 (m, 1H), 1.85 (d, *J* = 1.1 Hz, 3H), 1.75 (t, *J* = 4.5 Hz, 6H), 1.53 (dd, *J* = 9.2, 4.4 Hz, 2H), 1.36 – 1.23 (m, 8H), 1.13 (dd, *J* = 6.7, 2.1 Hz, 3H), 1.04 (s, 9H), 0.91 – 0.89 (m, 3H), 0.89 – 0.86 (m, 18H), 0.02 (s, 3H), -0.01 (d, *J* = 1.4 Hz, 3H), -0.02 (s, 3H), -0.02 (s, 3H); **^{13}C-NMR** (176 MHz, $CDCl_3$): δ [ppm] = 203.8, 139.9, 137.6, 137.1, 136.6, 135.6, 134.2, 132.5, 132.4, 131.7, 131.4, 130.1, 129.5, 129.4, 129.1, 128.9, 127.6, 120.9, 76.4, 75.8, 64.0, 46.6, 38.7, 33.1, 32.6, 29.7, 26.8, 26.3, 26.0, 25.9 24.9, 20.2, 19.2, 18.1, 18.1, 15.4, 14.6, 11.9, -4.0, -4.4, -4.45, -4.8; **HRMS (ESI+)** calculated for $C_{56}H_{30}O_4Si_3Na^+$ $[M+Na]^+$: 933.6039, found : 933.6039.

- *2.8.2 PMB as R_2*

Synthesis of aldehyde 63

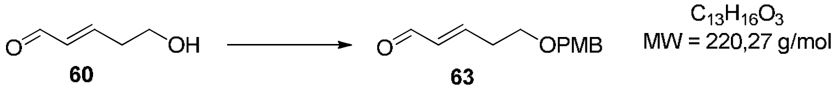

To a solution of alcohol **60** (41 mg, 0.40 mmol, 1.00 eq) and PMB acetimidate (173 mg, 0.61 mmol, 1.50 eq) in DCM (3 mL) was added CSA (9.5 mg, 0.04 mmol, 0.10 eq) at room temperature. The reaction was stirred overnight. The solution was filtered throught celite and the filtrate was washed with a saturated solution of $NaHCO_3$ (2*5 mL). The organic layer was dried over $MgSO_4$ and evaporated *in vacuo*. The crude product was purified by column chromatography (SiO_2, CH/EtOAc, 5:1) to obtain aldehyde **63** (60 mg, 0.30 mmol, 75 %) as a thick colorless oil.

***R*$_f$** = 0.47 (SiO_2, CH/EtOAc, 1:1); **^{1}H-NMR** (700 MHz, $CDCl_3$): δ [ppm] = 9.51 (d, *J* = 7.9 Hz, 1H), 7.26 – 7.24 (m, 2H), 6.90 – 6.85 (m, 3H), 6.17 (ddt, *J* = 15.7, 7.9, 1.5 Hz, 1H), 4.46 (s, 2H), 3.81 (s, 3H), 3.61 (t, *J* = 6.2 Hz, 2H), 2.62 (qd, *J* = 6.3, 1.5 Hz, 2H); **^{13}C-NMR** (176 MHz, $CDCl_3$): δ [ppm] = 194.0, 159.4, 155.2, 134.2, 130.0, 129.4, 113.9, 72.8, 67.6, 55.3, 33.1; **HRMS (ESI+)** calculated for $C_{13}H_{17}O_3^+$ $[M+H]^+$: 221.1172, found : 221.1178.

Synthesis of ketone 64

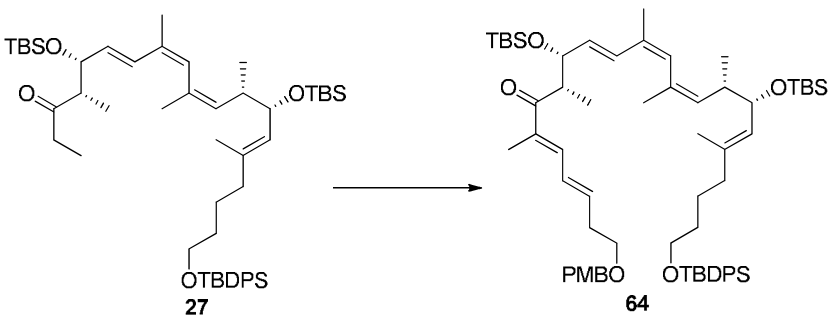

$C_{65}H_{100}O_6Si_3$
MW = 1061,76 g/mol

Preparation of LiTMP: To a solution of TMP (90 µL, 0.53 mmol, 2.00 eq) in THF (2 mL) at -78 °C was added *n*-BuLi (1.6 M in hexane, 0.33 mL, 0.53 mmol, 2.00 eq). The yellow solution was stirred for 15 min at this temperature and 15 min at 0 °C.

Ketone **27** (230 mg, 0.27 mmol, 1.00 eq) was diluted in THF (4 mL) and dried over 3Å MS for 30 min. The solution was then cooled down at -78 °C, LiTMP (2.3 mL, 0.54 mmol, 2.00 eq) was added dropwise and the mixture was stirred for 30 min at -78 °C and warmed up to -50 °C for 20 min. The enolate solution was cooled down at -78 °C and aldehyde **63** (88 mg, 0.40 mmol, 1.50 eq, dried over 3Å MS in 0.4 mL THF) was added dropwise. After 2 h, the reaction mixture was diluted with DCM (10 mL) and quenched with a saturated solution of $NaHCO_3$ (10 mL) at 0 °C. After the separation of the organic layer, the aqueous layer was extracted with DCM (3*15 mL). The organic layers were combined, dried over $MgSO_4$ and evaporated *in vacuo*. The crude product was purified by column chromatography (SiO_2, CH/EtOAc, 30:1 to 10:1) give the aldol product (262 mg, 0.25 mmol, 92 %).

The aldol product was diluted in THF (10 mL), DMAP (296 mg, 2.42 mmol, 10.0 eq) and Ac_2O (0.2 mL, 2.20 mmol, 9.00 eq) were added at 0 °C. After 30 min, buffer pH 7 (10 mL) was added. After separation of the organic layer, the aqueous layer was extracted with Et_2O (3*10 mL). The organic layers were combined, dried over $MgSO_4$ and evaporated under vacuum. The crude product was purified by column chromatography (SiO_2, CH/EtOAc, 20:1) give the protected alcohol (232 mg, 0.21 mmol, 86 %).

The crude mixture was diluted in THF (10 mL) and DBU (1.0 mL, 7.24 mmol, 35.0 eq) was added at room temperature. After two days, the reaction was quenched with pH7 buffer (6 mL). After separation of the organic layer, the aqueous layer was extracted with Et_2O (3*10 mL). The organic layers were combined, dried over $MgSO_4$ and evaporated under vacuum. The crude product was purified by column chromatography (SiO_2, CH/EtOAc, 100:1) to give the desired product **64** as a colorless oil (176 mg, 0.17 mmol, 61 % over three steps).

$\boldsymbol{R}_f$ = 0.48 (SiO_2, CH/EtOAc, 10:1); $[\boldsymbol{\alpha}]_{\boldsymbol{D}}^{\boldsymbol{20}}$ = - 3.0° (*c* = 0.23, $CHCl_3$); **^{1}H-NMR** (700 MHz, CD_2Cl_2): δ [ppm] = 7.68 – 7.65 (m, 4H), 7.43 – 7.36 (m, 6H), 7.27 – 7.23 (m, 2H), 7.00 (d, *J* = 10.8 Hz, 1H), 6.89 – 6.84 (m, 2H), 6.50 (ddt, *J* = 15.1, 10.8, 1.5 Hz, 1H), 6.41 – 6.31 (m, 1H), 6.14 (dt, *J* = 14.6, 7.0 Hz, 1H), 5.90 (s, 1H), 5.57 – 5.51 (m, 1H), 5.12 – 5.06 (m, 2H), 4.43 (d, *J* = 2.3 Hz, 2H), 4.26 – 4.19 (m, 1H), 4.12 (ddd, *J* = 9.0, 6.0, 2.6 Hz, 1H), 3.78 (s, 3H), 3.68 (t, *J* = 6.1 Hz, 2H), 3.56 – 3.52 (m, 2H), 3.43 (q, *J* = 7.0 Hz, 1H), 2.49 (dt, *J* = 7.5, 6.1 Hz, 2H), 2.37 – 2.29 (m, 1H), 1.98 (t, *J* = 7.3 Hz, 2H), 1.79 (s, 3H), 1.77 – 1.73 (m, 6H), 1.58 (d, *J* = 1.4 Hz, 3H), 1.08 (dd, *J* = 6.8, 2.0 Hz, 3H), 1.04 (s, 9H), 0.89 – 0.86 (m, 12H), 0.85 (d, *J* = 1.7 Hz, 9H), 0.02 (s, 3H), -0.01 (s, 3H), -0.02 (s, 3H), -0.04 (s, 3H); **^{13}C-NMR** (176 MHz, CD_2Cl_2): δ [ppm] = 203.6, 159.2, 139.6, 138.1, 135.5, 135.4, 135.2, 134.2, 132.7, 132.2, 132.0, 131.4, 130.6,

130.1, 129.5, 129.2, 128.2, 127.6, 127.2, 113.6, 76.8, 72.9, 72.5, 69.0, 63.8, 55.2, 46.3, 40.6, 39.3, 33.8, 32.2, 26.6, 25.6, 24.6, 24.0, 20.1, 19.1, 18.0, 16.4, 15.5, 14.2, 11.5, -4.2, -4.6, -5.1, -5.2; **HRMS (ESI+)** calculated for $C_{65}H_{100}O_6Si_3Na^+$ $[M+Na]^+$: 1083.6720 found : 1083.6720.

Synthesis of methyl ether 65

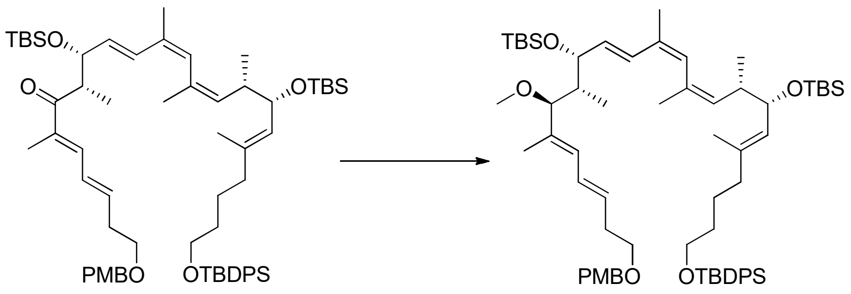

$C_{66}H_{104}O_6Si_3$
MW = 1077,78 g/mol

To a solution of ketone **64** (176 mg, 0.17 mmol, 1.00 eq) in MeOH (5 mL) and THF (1 mL) at 0 °C was added $NaBH_4$ (25 mg, 0.66 mmol, 4.00 eq). The solution was warmed up to room temperature and after 2 h, more $NaBH_4$ (12.5 mg, 0.33 mmol, 2.00 eq) was added. After 3 h, the reaction was diluted with EtOAc (10 mL) and quenched with a saturated solution of NH_4Cl (8 mL) at 0 °C. After separation of the organic layer, the aqueous layer was extracted with EtOAc (3*15 mL). The organic layers were combined, dried over $MgSO_4$ and evaporated *in vacuo*. The crude product was purified by column chromatography (SiO_2, CH/EtOAc, 60:1 to 30:1) to give the alcohol as a thick oil (157 mg, 0.15 mmol, 89 %, *dr* > 10:1).

To a solution of alcohol (153 mg, 0.14 mmol, 1.00 eq) in DCM (8 mL) at 0 °C was added proton sponge (169 mg, 0.79 mmol, 5.50 eq) followed by $MeOBF_4$ (106 mg, 0.72 mmol, 5.00 eq). The reaction was stirred for 3 h at 0 °C and 2 h at room temperature. After this time, a saturated solution of $NaHCO_3$ (10 mL) was added at 0 °C. After separation of the organic layer, the aqueous layer was extracted with DCM (3*15 mL). The organic layers were combined, dried over $MgSO_4$ and evaporated *in vacuo*. The crude product was purified by column chromatography (SiO_2, CH/EtOAc, 60:1) to give the methyl ether **65** (135 mg, 125 µmol, 87 %).

$\boldsymbol{R}_f$ = 0.51 (SiO_2, CH/EtOAc, 10:1); $[\alpha]_D^{20}$ = + 23.7° (*c* = 0.27, $CHCl_3$); **^{1}H-NMR** (500 MHz, CD_2Cl_2): δ [ppm] = 7.72 – 7.65 (m, 5H), 7.45 – 7.36 (m, 7H), 7.28 – 7.22 (m, 2H), 6.90 – 6.84 (m, 2H), 6.46 – 6.40 (m, 1H), 6.36 (ddt, *J* = 15.2, 10.8, 1.5 Hz, 1H), 5.93 – 5.89 (m, 1H), 5.86 (s, 1H), 5.74 – 5.66 (m, 2H), 5.08 (ddd, *J* = 9.8, 2.6, 1.2 Hz, 2H), 4.69 (dt, *J* = 7.0, 1.5 Hz, 1H), 4.42 (s, 2H), 4.12 (dd, *J* = 9.0, 5.8 Hz, 1H), 3.78 (s, 3H), 3.67 (t, *J* = 6.1 Hz, 2H), 3.49 (t, *J* = 6.7 Hz, 2H), 3.34 (d, *J* = 9.9 Hz, 1H), 3.10 (s, 3H), 2.44 – 2.31 (m, 4H), 1.99 – 1.95 (m, 2H), 1.84 (d, *J* = 1.3 Hz, 2H), 1.78 (dd, *J* = 1.4, 0.7 Hz, 3H), 1.59 – 1.55 (m, 7H), 1.53 – 1.44 (m, 4H), 1.03 (s, 10H), 0.93 – 0.90 (m, 9H), 0.87 – 0.85 (m, 3H), 0.85 – 0.83 (m, 9H), 0.63 (dd, *J* = 7.0, 3.1 Hz, 3H), 0.06 – 0.03 (m, 3H), 0.01 – -0.02 (m, 6H), -0.04 (d, *J* = 4.2 Hz, 3H); **^{13}C-NMR** (125 MHz, CD_2Cl_2): δ [ppm] = 159.2, 135.5, 135.4, 134.4, 134.1, 133.9, 132.6, 132.1, 130.8, 130.7, 129.5, 129.2, 129.1, 127.8, 127.6, 127.5, 127.1, 113.6, 88.2, 72.8, 72.4, 71.7, 69.6, 63.8, 55.4, 55.2, 42.6, 40.5, 39.3, 33.4, 32.2, 26.6, 25.7, 25.6, 24.5, 24.0, 20.2, 19.1, 18.1, 18.0, 16.4, 15.1, 10.3, 8.8, -4.0, -4.6, -5.2, -5.4; **HRMS (ESI+)** calculated for $C_{66}H_{104}O_6Si_3Na^+$ $[M+Na]^+$:1099.7033 found : 1099.7033.

Synthesis of alcohol 66

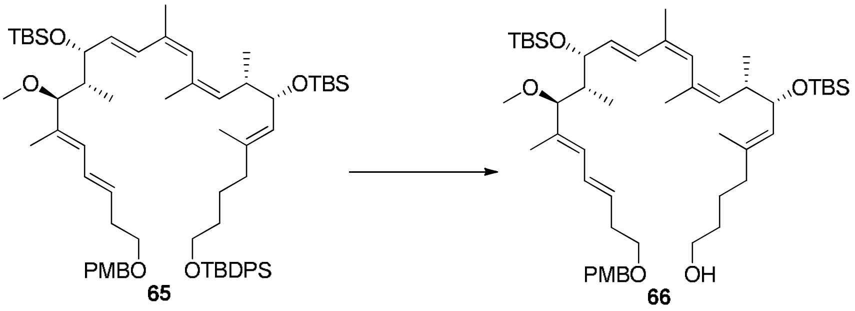

$C_{50}H_{86}O_6Si_2$
MW = 839,40 g/mol

TBAF stock solution : To a solution of TBAF (1 M in THF, 415 µL, 0.42 mmol, 1.00 eq) in THF (9.56 mL) at 0 °C was added AcOH (24 µL, 0.42 mmol, 1.00 eq) resulting in a 41.5 mM solution.

To the neat alcohol **65** (38 mg, 38 µmol, 1.00 eq) was added the stock solution at 0 °C (1.1 mL, 45 µmol, 1.10 eq). The reaction was stirred for 1 h at this temperature then 44 h at room temperature. The reaction was diluted with Et_2O (3 mL) and quenched with a saturated solution of $NaHCO_3$ (2 mL) at 0 °C. After separation of the organic layer, the aqueous layer was extracted with Et_2O (3*10 mL). The organic layers were combined, dried over $MgSO_4$ and evaporated *in vacuo*. The crude product was purified by column chromatography (SiO_2, CH/EtOAc, 15:1 to 5:1) to give alcohol **66** as a colorless oil (25 mg, 30 µmol, 79 %).

$\boldsymbol{R}_f$ = 0.16 (SiO_2, CH/EtOAc, 10:1); $[\boldsymbol{\alpha}]_{\boldsymbol{D}}^{\boldsymbol{20}}$ = + 20.3° (*c* = 0.31, $CHCl_3$); **^{1}H-NMR** (500 MHz, CD_2Cl_2): δ [ppm] = 7.26 – 7.23 (m, 2H), 6.88 – 6.84 (m, 2H), 6.45 – 6.40 (m, 1H), 6.36 (ddt, *J* = 15.1, 10.7, 1.4 Hz, 1H), 5.94 – 5.89 (m, 1H), 5.85 (s, 1H), 5.74 – 5.65 (m, 2H), 5.12 – 5.05 (m, 2H), 4.68 (dt, *J* = 7.0, 1.5 Hz, 1H), 4.42 (s, 2H), 4.14 – 4.10 (m, 1H), 3.78 (s, 3H), 3.63 – 3.56 (m, 2H), 3.34 (d, *J* = 9.9 Hz, 1H), 3.10 (d, *J* = 3.3 Hz, 3H), 2.44 – 2.31 (m, 3H), 2.02 – 1.97 (m, 2H), 1.85 – 1.82 (m, 3H), 1.81 – 1.77 (m, 3H), 1.58 (d, *J* = 1.4 Hz, 3H), 1.57 – 1.55 (m, 3H), 1.52 – 1.43 (m, 4H), 0.94 – 0.89 (m, 9H), 0.87 – 0.82 (m, 13H), 0.63 (dd, *J* = 7.0, 2.9 Hz, 3H), 0.06 – 0.03 (m, 3H), 0.01 – -0.02 (m, 6H), -0.04 (m, 3H); **^{13}C-NMR** (125 MHz, CD_2Cl_2): δ [ppm] = 159.2, 135.2, 134.4, 133.9, 132.6, 132.5, 132.2, 130.8, 129.5, 129.2, 129.1, 127.8, 127.5, 127.2, 113.6, 88.2, 72.8, 72.4, 71.8, 69.6, 62.6, 55.4, 55.2, 42.7, 40.4, 39.3, 33.4, 32.4, 25.7, 25.6, 24.5, 23.9, 20.2, 18.1, 18.0, 16.4, 15.2, 10.3, 8.8, -4.0, -4.7, -5.2, -5.4; **HRMS** calculated for $C_{50}H_{96}O_6Si_2Na^+$ $[M+Na]^+$: 861.5855 found : 861.5855.

Synthesis of methyl ester 67

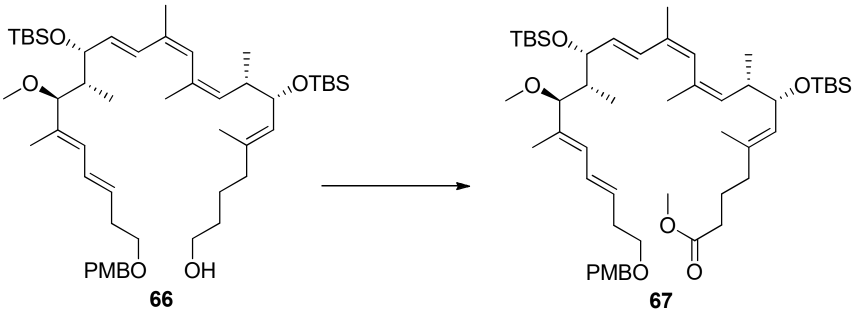

$C_{51}H_{86}O_7Si_2$
MW = 867,41 g/mol

To a solution of alcohol **66** (60 mg, 98 µmol, 1.00 eq) in DCM (4 mL) at 0 °C was added DMP (23 mg, 54 µmol, 1.30 eq). The solution was warmed up to room temperature and stirred for 2 h.

After this time the reaction was cooled to 0 °C again, diluted with Et_2O (6 mL) and filtered through celite. The solvents were evaporated and this sequence was repeated two times.

The crude aldehyde was then diluted in *tert*-butanol (2 mL) and 2-methylbut-2-ene (2 mL) and cooled at 0 °C. $NaClO_2$ (28.5 mg, 310 µmol, 3.20 eq), KH_2PO_4 (53.6 mg, 390 µmol, 4.00 eq) and H_2O (20 µL) were added, the reaction was stirred for 1 h at room temperature. Saturated solution of NaCl (4 mL) was added followed by Et_2O (5 mL). After separation of the organic layer, the aqueous layer was extracted with Et_2O (3*5 mL). The organic layers were combined, dried over $MgSO_4$ and evaporated *in vacuo*.

The crude carboxylic acid was diluted in MeOH (2 mL) and cooled down to 0 °C. $TMSCHN_2$ (0.15 mL, 305 µmol, 4.00 eq) was added dropwise and the reaction was stirred for 20 min. Then AcOH was added until the solution became colorless. Volatiles were evaporated and the crude product was directly purified by column chromatography (SiO_2, CH/EtOAc, 30:1) to give the desired methyl ester **67** (47.5 mg, 74.5 µmol, 76 %).

R**f** = 0.42 (SiO_2, CH/EtOAc, 20:1). $[\alpha]_D^{20}$ = + 10.7° (*c* = 0.74, $CHCl_3$); **^{1}H-NMR** (700 MHz, CD_2Cl_2): δ [ppm] = 7.26 – 7.24 (m, 2H), 6.87 – 6.85 (m, 2H), 6.43 (dt, *J* = 15.9, 1.0 Hz, 1H), 6.38 – 6.34 (m, 1H), 5.92 (dd, *J* = 10.7, 1.6 Hz, 1H), 5.86 (s, 1H), 5.75 – 5.66 (m, 2H), 5.09 (dddt, *J* = 14.0, 8.3, 2.9, 1.3 Hz, 2H), 4.69 (dt, *J* = 7.1, 1.6 Hz, 1H), 4.42 (s, 2H), 4.12 (ddd, *J* = 8.3, 5.5, 2.7 Hz, 1H), 3.79 (s, 3H), 3.63 (s, 3H), 3.50 (t, *J* = 6.6 Hz, 3H), 3.35 (d, *J* = 9.9 Hz, 1H), 3.11 – 3.10 (m, 3H), 2.43 – 2.39 (m, 3H), 2.39 – 2.35 (m, 1H), 2.26 (td, *J* = 7.6, 3.6 Hz, 2H), 2.02 – 1.98 (m, 2H), 1.85 (dd, *J* = 4.6, 1.5 Hz, 3H), 1.80 – 1.77 (m, 3H), 1.75 – 1.72 (m, 3H), 1.59 – 1.58 (m, 4H), 1.57 (d, *J* = 1.3 Hz, 3H), 0.92 (s, 9H), 0.86 (d, *J* = 6.8 Hz, 3H), 0.85 (s, 9H), 0.64 – 0.62 (m, 3H), 0.05 (s, 3H), -0.01 (s, 3H), -0.01 (s, 3H), -0.04 (S, 3H); **^{13}C-NMR** (176 MHz, CD_2Cl_2): δ [ppm] = 173.8, 159.2, 134.4, 134.4, 133.9, 132.6, 132.4, 132.3, 130.8, 130.7, 129.5, 129.2, 129.2, 129.1, 127.8, 127.8, 127.5, 113.6, 88.2, 72.7, 72.4, 71.8, 69.6, 55.4, 55.2, 53.8, 53.7, 53.6, 53.6, 53.5, 53.4, 53.3, 53.1, 51.2, 42.7, 40.4, 38.8, 33.4, 33.3, 25.8, 25.7, 25.6, 25.6, 24.5, 23.0, 20.1, 18.1, 18.0, 16.3, 15.1, 10.3, 8.8, -4.1, -4.7, -5.2, -5.4; **HRMS (ESI+)** calculated for $C_{51}H_{90}O_7Si_2N^+$ $[M+NH_4]^+$: 884.6250, found : 884.6250.

Synthesis of alcohol 68

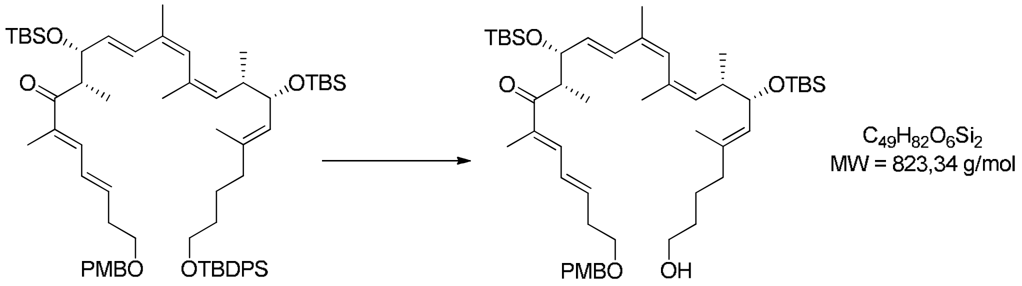

67 **68**

TBAF stock solution : To a solution of TBAF (1 M in THF, 415 µL, 0.42 mmol, 1.00 eq) in THF (9.56 mL) at 0 °C was added AcOH (24 µL, 0.42 mmol, 1.00 eq) resulting in a 41.5 mM solution.

To the neat protected alcohol **67** (78 mg, 73 µmol, 1.00 eq) was added the stock solution at 0 °C (1.9 mL, 81 µmol, 1.10 eq). The reaction was stirred for 1 h at this temperature then 44 h at room temperature. The reaction was diluted with Et_2O (3 mL) and quenched with a saturated solution of $NaHCO_3$ (2 mL) at 0 °C. After separation of the organic layer, the aqueous layer was extracted with Et_2O (3*10 mL). The organic layers were combined, dried over $MgSO_4$ and evaporated *in vacuo*. The

crude product was purified by column chromatography (SiO_2, CH/EtOAc, 20:1 to 5:1) to give alcohol **68** as yellowish oil (51 mg, 62 µmol, 85 %).

***R*f** = 0.16 (SiO_2, CH/EtOAc, 10:1); $[\alpha]_D^{20}$ = -14.9° (*c* = 0.35, $CHCl_3$); **^{1}H-NMR** (500 MHz, CD_2Cl_2): δ [ppm] = 7.28 – 7.22 (m, 2H), 7.00 (d, *J* = 10.6 Hz, 1H), 6.91 – 6.83 (m, 2H), 6.50 (ddt, *J* = 15.1, 10.8, 1.5 Hz, 1H), 6.34 (dt, *J* = 15.7, 0.8 Hz, 1H), 6.20 – 6.07 (m, 1H), 5.90 (d, *J* = 10.4 Hz, 1H), 5.58 – 5.51 (m, 1H), 5.09 (ddq, *J* = 8.9, 4.1, 1.5 Hz, 2H), 4.43 (d, *J* = 1.8 Hz, 2H), 4.30 – 4.20 (m, 1H), 4.15 – 4.09 (m, 1H), 3.78 (d, *J* = 1.0 Hz, 3H), 3.63 – 3.57 (m, 2H), 3.56 – 3.52 (m, 2H), 3.46 – 3.37 (m, 1H), 2.53 – 2.47 (m, 2H), 2.37 – 2.28 (m, 1H), 2.02 – 1.97 (m, 2H), 1.82 – 1.70 (m, 8H), 1.59 – 1.55 (m, 3H), 1.09 (dd, *J* = 6.8, 4.8 Hz, 3H), 0.89 – 0.87 (m, 3H), 0.86 (s, 8H), 0.85 – 0.84 (m, 9H), 0.02 (s, 3H), -0.01 (s, 3H), -0.03 (s, 3H), -0.04 (s, 3H); **^{13}C-NMR** (125 MHz, CD_2Cl_2): δ [ppm] = 204.3, 159.8, 140.2, 138.7, 135.8, 135.7, 133.2, 132.8, 132.6, 132.0, 131.2, 130.6, 129.8, 129.8, 128.7, 128.0, 114.2, 77.3, 73.5, 73.1, 69.6, 69.6, 63.2, 55.8, 54.4, 54.2, 54.1, 54.0, 53.8, 53.6, 46.9, 41.1, 39.9, 34.4, 33.0, 26.2, 26.2, 25.2, 24.5, 20.7, 18.6, 18.6, 17.0, 16.1, 14.8, 12.1, -3.7, -4.0, -4.5, -4.6; **HRMS (ESI+)** calculated for $C_{49}H_{86}O_6Si_2Na^+$ $[M+Na]^+$: 845.5542, found : 845.5542.

Synthesis of methyl ester 69

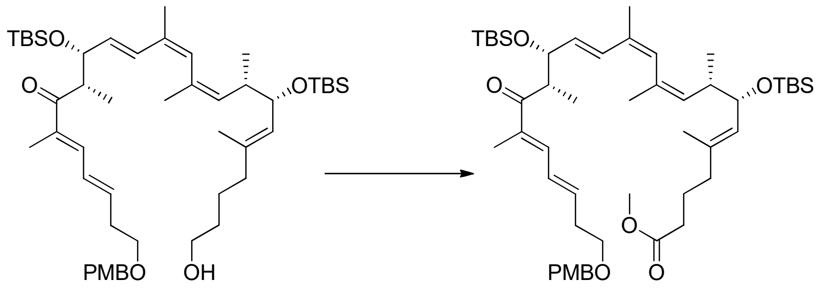

$C_{50}H_{82}O_7Si_2$
MW = 851,37 g/mol

To a solution of DMSO (43 µL, 607 µmol, 10.0 eq), sulfur trioxide pyridine complex (29 mg, 182 µmol, 3.00 eq) and DIEA (42 µL, 243 µmol, 4.00 eq) in DCM (4 mL) at 0 °C was added alcohol **68** (50 mg, 60 µmol, 1.00 eq). The solution was stirred at 0 °C for 1.5 h. After this time the reaction was quenched with aqueous saturated solution of $NaHCO_3$ (3 mL) and diluted with DCM (3 mL). After separation of the organic layer, the aqueous layer was extracted with DCM (3*5 mL).The organic layers were combined, dried over $MgSO_4$ and evaporated *in vacuo* until 200 mbar.

The crude aldehyde was diluted in *tert*-butanol (2 mL) and 2-methylbut-2-ene (0.1 mL) and cooled at 0 °C. A solution of $NaClO_2$ (15 mg, 168 µmol, 3.20 eq), KH_2PO_4 (28 mg, 209 µmol, 4.00 eq) in H_2O (2 mL) was added to the solution. The reaction was stirred for 1 h at room temperature. Saturated solution of NaCl (5 mL) was added followed by Et_2O (5 mL). After separation of the organic layer, the aqueous layer was extracted with Et_2O (3*5 mL). The organic layers were combined, dried over $MgSO_4$ and evaporated *in vacuo*.

The crude carboxylic acid was diluted in MeOH (2 mL) and cooled down to 0 °C. $TMSCHN_2$ (0.13 mL, 260 µmol, 5.00 eq) was added dropwise and the reaction was stirred for 20 min. Then AcOH was added until the solution became colorless. Volatiles were evaporated and the crude product was directly purified by column chromatography (SiO_2, CH/EtOAc, 30:1 to 20:1) to give methyl ester **69** (28 mg, 33 µmol, 54 %).

***R*f** = 0.41 (SiO_2, CH/EtOAc, 10:1); $[\alpha]_D^{25}$ = -2.6° (*c* = 0.23, $CHCl_3$); **^{1}H-NMR** (500 MHz, $CDCl_3$): δ [ppm] = 7.26 – 7.23 (m, 2H), 7.00 (d, *J* = 10.7 Hz, 1H), 6.86 (dd, *J* = 6.8, 4.8 Hz, 2H), 6.54 – 6.47 (m, 1H), 6.36 –

6.33 (m, 1H), 6.18 – 6.11 (m, 1H), 5.89 (s, 1H), 5.54 (dd, *J* = 15.7, 7.7 Hz, 1H), 5.12 – 5.07 (m, 2H), 4.43 (s, 2H), 4.23 (dd, *J* = 9.6, 4.9 Hz, 8H), 4.14 – 4.09 (m, 2H), 3.79 (s, 3H), 3.63 (s, 3H), 3.55 (t, *J* = 6.5 Hz, 2H), 3.43 (dt, *J* = 13.8, 6.9 Hz, 1H), 2.50 (q, *J* = 6.6 Hz, 2H), 2.33 (ddd, *J* = 9.7, 6.4, 2.8 Hz, 1H), 2.26 (dd, *J* = 10.4, 4.7 Hz, 2H), 2.00 (dd, *J* = 8.3, 6.5 Hz, 2H), 1.78 (d, *J* = 3.0 Hz, 3H), 1.77 (d, *J* = 1.4 Hz, 6H), 1.73 – 1.69 (m, 2H), 1.58 (d, *J* = 2.2 Hz, 3H), 1.08 (d, *J* = 6.7 Hz, 3H), 0.87 – 0.86 (m, 9H), 0.85 (q, *J* = 2.1 Hz, 12H), 0.02 (s, 3H), -0.01 (s, 3H), -0.02 (s, 3H), -0.04 (s, 3H); **^{13}C-NMR** (125 MHz, $CDCl_3$): δ [ppm] = 203.6, 173.8, 159.2, 139.6, 138.1, 135.2, 134.4, 132.5, 132.3, 132.2, 131.5, 130.6, 130.0, 129.6, 129.2, 128.2, 128.0, 120.6, 115.1, 113.6, 76.8, 72.8, 72.5, 69.0, 55.2, 53.8, 53.6, 53.5, 53.4, 53.2, 53.0, 51.2, 46.3, 40.5, 38.8, 33.8, 33.3, 25.6, 25.6, 25.6, 25.4, 24.6, 23.0, 20.1, 18.0, 18.0, 16.3, 15.5, 14.2, 11.5, 11.5, -4.3, -4.7, -5.1, -5.2; **HRMS (ESI+)** calculated for $C_{50}H_{82}O_7Si_2Na^+$ $[M+Na]^+$: 873.5491, found : 879.5491.

Synthesis of alcohol 70

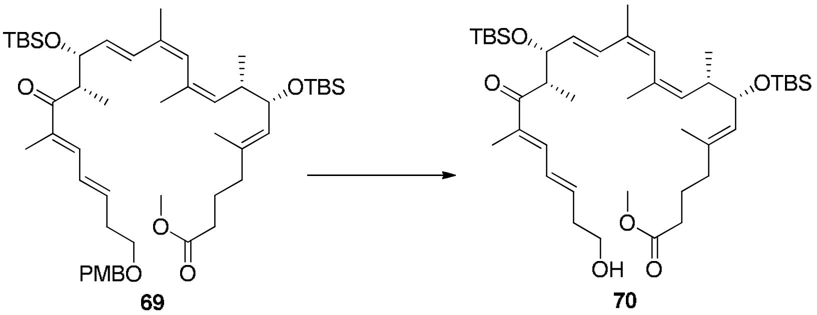

$C_{42}H_{74}O_6Si_2$
MW = 731,22 g/mol

To a solution of **69** (15 mg, 17.6 µmol, 1.00 eq) in DCM/pH7 Buffer (10:1) was added DDQ (8 mg, 35.2 µmol, 2.00 eq). The reaction was stirred for 3 h at room temperature. After this time the reaction was quenched with aqueous saturated solution of $NaHCO_3$ (3 mL) and diluted with DCM (3 mL). After separation of the organic layer, the aqueous layer was extracted with DCM (3*5 mL).The organic layers were combined, dried over $MgSO_4$ and evaporated *in vacuo*. The crude product was purified by column chromatography (SiO_2, CH/EtOAc, 20:1 to 5:1) to give alcohol **70** as yellowish oil (4 mg, 5.5 µmol, 31 %).

$\boldsymbol{R}_f$ = 0.15 (SiO_2, CH/EtOAc, 5:1); $[\boldsymbol{\alpha}]_{\boldsymbol{D}}^{\mathbf{20}}$ = - 18.3° (*c* = 0.46, $CHCl_3$); **^{1}H-NMR** (700 MHz, CD_2Cl_2): δ [ppm] = 7.00 (d, *J* = 10.5 Hz, 1H), 6.53 (ddt, *J* = 15.1, 10.9, 1.4 Hz, 1H), 6.35 (d, *J* = 16.0 Hz, 1H), 6.12 (dt, *J* = 14.8, 7.2 Hz, 1H), 5.89 (d, *J* = 1.7 Hz, 1H), 5.57 – 5.52 (m, 1H), 5.09 (dq, *J* = 10.3, 1.4 Hz, 2H), 4.24 (td, *J* = 7.3, 1.0 Hz, 1H), 4.13 – 4.10 (m, 1H), 3.71 (t, *J* = 6.4 Hz, 2H), 3.63 (d, *J* = 1.9 Hz, 3H), 3.44 – 3.41 (m, 1H), 2.49 – 2.44 (m, 2H), 2.36 – 2.31 (m, 1H), 2.27 (td, *J* = 7.9, 2.7 Hz, 2H), 2.02 – 1.98 (m, 2H), 1.79 (d, *J* = 1.2 Hz, 3H), 1.78 – 1.76 (m, 5H), 1.73 – 1.70 (m, 2H), 1.58 (d, *J* = 1.4 Hz, 3H), 1.10 – 1.07 (m, 3H), 0.87 (s, 9H), 0.85 – 0.84 (m, 12H), -0.01 (s, 3H), -0.03 (s, 3H), -0.04 (s, 3H); **^{13}C-NMR** (176 MHz, $CDCl_3$): δ [ppm] = 203.6, 173.9, 138.9, 137.8, 135.4, 134.4, 132.5, 132.3, 131.4, 130.0, 129.2, 128.9, 128.0, 76.7, 72.8, 61.6, 53.8, 53.8, 53.7, 53.6, 53.6, 53.5, 53.4, 53.3, 53.1, 51.2, 46.4, 40.5, 38.8, 36.7, 33.3, 25.6, 25.6, 24.6, 23.0, 20.1, 18.0, 18.0, 16.3, 15.5, 14.1, 11.6, -4.2, -4.7, -5.1, -5.2; **HRMS (ESI+)** calculated for $C_{42}H_{74}O_6Si_3Na^+$ $[M+Na]^+$: 753.4916 found 753.4915 .

- *2.8.3 Esters as R_1*

Synthesis of methyl ester 71

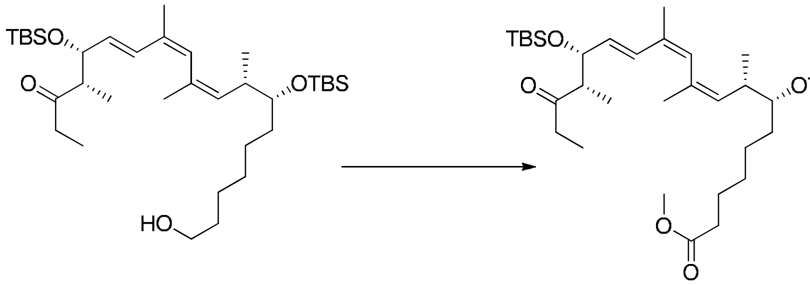

$C_{36}H_{68}O_5Si_2$
MW = 637,11 g/mol

57 **71**

To a solution of alcohol **57** (60 mg, 98 µmol, 1.00 eq) in DCM (4 mL) at 0 °C was added DMP (23 mg, 54 µmol, 1.30 eq). The solution was warmed up to room temperature and stirred for 2 h. After this time the reaction was cooled to 0 °C again, diluted with Et_2O (4 mL) and filtered through celite. The solvents were evaporated and this sequence was repeated two times.

The crude aldehyde was diluted in *tert*-butanol (2 mL) and 2-methylbut-2-ene (2 mL) and cooled at 0 °C. A solution of $NaClO_2$ (28.5 mg, 310 µmol, 3.20 eq), KH_2PO_4 (53.6 mg, 390 µmol, 4.00 eq) in H_2O (2 mL) was added. The reaction was stirred for 1 h at room temperature. Saturated solution of NaCl (3 mL) was added followed by Et_2O (3 mL). After the separation of the organic layer, the aqueous layer was extracted with Et_2O (3*3 mL). The organic layers were combined, dried over $MgSO_4$ and evaporated *in vacuo*.

The crude carboxylic acid was diluted in MeOH (2 mL) and cooled down to 0 °C. $TMSCHN_2$ (0.15 mL, 305 µmol, 4.00 eq) was added dropwise and the reaction was stirred for 20 min. Then AcOH was added until the solution became colorless. Volatiles were evaporated and the crude product was directly purified by column chromatography (SiO_2, CH/EtOAc 30:1) to give methyl ester **71** (47.5 mg, 74.5 µmol, 76 %).

$\boldsymbol{R_f}$ = 0.42 (SiO_2, CH/EtOAc, 20:1). $[\alpha]_D^{20}$ = + 11.0° (*c* = 0.48, $CHCl_3$); **^{1}H-NMR** (700 MHz, $CDCl_3$): δ [ppm] = δ 6.39 (d, *J* = 15.7 Hz, 1H), 5.88 (s, 1H), 5.59 (dd, *J* = 15.7, 7.4 Hz, 1H), 5.23 – 5.10 (m, 1H), 4.34 (t, *J* = 6.8 Hz, 1H), 3.69 (s, 4H), 3.47 – 3.38 (m, 1H), 2.72 (dd, *J* = 7.1, 6.1 Hz, 1H), 2.60 – 2.36 (m, 4H), 2.32 (t, *J* = 7.6 Hz, 3H), 1.85 (d, *J* = 1.3 Hz, 3H), 1.79 (d, *J* = 1.2 Hz, 3H), 1.63 (t, *J* = 7.4 Hz, 2H), 1.37 – 1.28 (m, 6H), 1.08 (d, *J* = 6.9 Hz, 3H), 1.05 – 0.98 (m, 3H), 0.89 (s, 22H), -0.00 – -0.03 (m, 12H); **^{13}C-NMR** (176 MHz, $CDCl_3$): δ [ppm] = 213.3, 174.3, 132.5, 132.4, 131.7, 130.7, 130.3, 129.5, 75.9, 75.7, 52.9, 51.4, 38.7, 36.7, 34.1, 32.8, 29.4, 25.9, 25.8, 25.6, 25.0, 24.9, 20.2, 18.1, 18.0, 15.1, 12.5, 7.5, -4.0, -4.4, -4.5, -4.9; **HRMS (ESI+)** calculated for $C_{36}H_{68}O_5Si_2Na^+$ $[M+Na]^+$: 659.4497, found : 659.4497.

Synthesis of ester 72

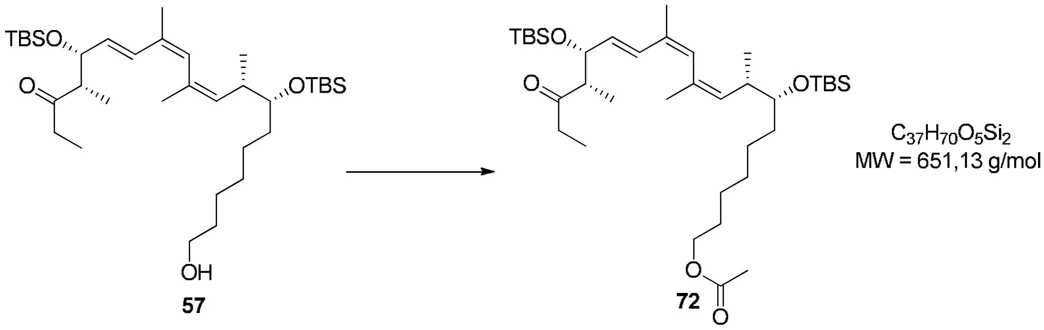

Alcohol **57** (23 mg, 37.8 µmol, 1.00 eq) was diluted in THF (2 mL), DMAP (14 mg, 113 µmol, 3.00 eq) and Ac_2O (7.5 µL, 75.5 µmol, 2.00 eq) were added at 0 °C. After 30 min, buffer pH 7 (3 mL) was added. After the separation of the organic layer, the aqueous layer was extracted with Et_2O (3*3 mL). The organic layers were combined, dried over $MgSO_4$ and evaporated under vacuum. The crude product was purified by column chromatography (SiO_2, CH/EtOAc, 20:1) affording protected alcohol **72** (23 mg, 35.3 µmol, 93 %).

***R*$_f$** = 0.13 (SiO_2, CH/EtOAc, 10:1); $[\alpha]_D^{20}$ = + 32.3° (*c* = 0.27, $CHCl_3$); **^{1}H-NMR** (700 MHz, CD_2Cl_2): δ [ppm] = 6.38 (dt, *J* = 15.8, 0.9 Hz, 1H), 5.92 – 5.87 (m, 1H), 5.62 – 5.55 (m, 1H), 5.18 (dq, *J* = 9.7, 1.5 Hz, 1H), 4.33 (ddd, *J* = 7.2, 5.9, 1.0 Hz, 1H), 4.01 (td, *J* = 6.8, 1.7 Hz, 2H), 3.44 (td, *J* = 6.5, 5.9, 3.8 Hz, 1H), 2.73 – 2.68 (m, 1H), 2.53 – 2.37 (m, 3H), 2.00 (s, 3H), 1.83 (d, *J* = 1.4 Hz, 2H), 1.79 – 1.77 (m, 2H), 1.63 – 1.57 (m, 2H), 1.37 – 1.25 (m, 8H), 1.04 (d, *J* = 7.0 Hz, 2H), 0.97 – 0.95 (m, 3H), 0.90 – 0.89 (m, 3H), 0.88 (d, *J* = 2.5 Hz, 9H), 0.87 (s, 8H)), 0.05 (s, 3H), -0.01 (s, 6H), -0.04 (s, 3H); **^{13}C-NMR** (176 MHz, CD_2Cl_2): δ [ppm] = 212.6, 170.9, 132.5, 132.4, 131.6, 130.8, 130.2, 129.4, 75.9, 75.8, 64.4, 53.8, 53.7, 53.6, 53.6, 53.5, 53.4, 53.3, 53.1, 52.9, 38.6, 36.4, 33.0, 29.5, 28.6, 26.1, 26.0, 25.7, 25.7, 25.6, 25.6, 25.6, 24.6, 20.7, 19.9, 18.0, 18.0, 15.1, 12.1, 7.2, -4.3, -4.7, -4.8, -5.2; **HRMS (ESI+)** calculated for $C_{37}H_{70}O_5Si_2Na^+$ $[M+Na]^+$: 673.4654, found : 674.4657.

3. Biological assays

3.1 MTT assay

Table 1: Antiproliferative activity of test compounds in 1321N1 astrocytoma cells determined by the MTT assay.

Compound	Growth inhibition<br>IC_{50} ± SEM (nM), n=3, duplicates (or % growth inhibition at 10 µM)
Analogue **5**	12.2 ± 2.9
Analogue **6**	19.6 ± 4.0
Analogue **7**	9.65 ± 1.48
Analogue **8**	17.4 ± 1.30

3.2 Human P2X3 Inhibition assay

Table 2: Antagonistic activity of archazolog derivatives at the human P2X3 receptor expressed in 1321N1 astrocytoma cells

Compound	Human P2X3 inhibition<br>IC_{50} ± SEM (nM)
Analogue **5**	2.46 ± 0.46
Analogue **6**	1.19 ± 0.18
Analogue **7**	1.02 ± 0.24
Analogue **8**	1.87 ± 0.03

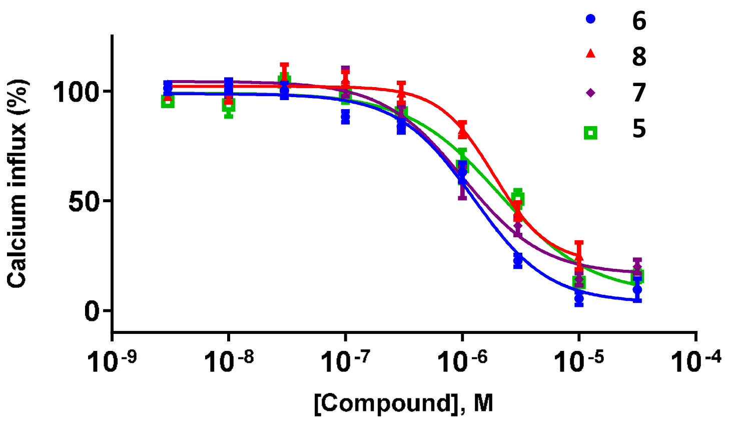

Figure 1: Concentration–response curves of selected antagonists at the human P2X3 receptor expressed in 1321N1 astrocytoma cells. Cells were preincubated for 30 min with test compound and subsequently activated with ATP at a concentration approximately corresponding to its EC_{80} value (60 nM).

3.3 A_3 Adenosine Receptor Radioligand Binding Assay

Table 3: A_3 Adenosine receptor (human recombinant) vs. [^{3}H]PSB 11

Compound	K*i* ± SEM (nM) (n=3)
Analogue **5**	539 ± 44
Analogue **6**	436 ± 111
Analogue **7**	>1000
Analogue **8**	>1000

3.4 Protease Assay

Table 4: Human leukocyte elastase inhibition. IC_{50} values were determined from ten different inhibitor concentrations in duplicates. Standard errors refer to the non-linear regression analysis.

Compound	K_i ± SEM (µM)
Analogue **5**	5.01 ± 0.79
Analogue **6**	13.3 ± 1.5
Analogue **7**	5.78 ± 0.65
Analogue **8**	8.18 ± 1.01

4. Copies of NMR spectra

Nucleus: 1H
Frequency: 499.13 MHz
Solvent: CDCl3
Temperature: 298.0 K

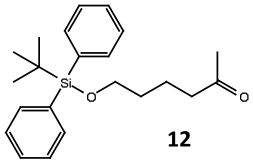

12

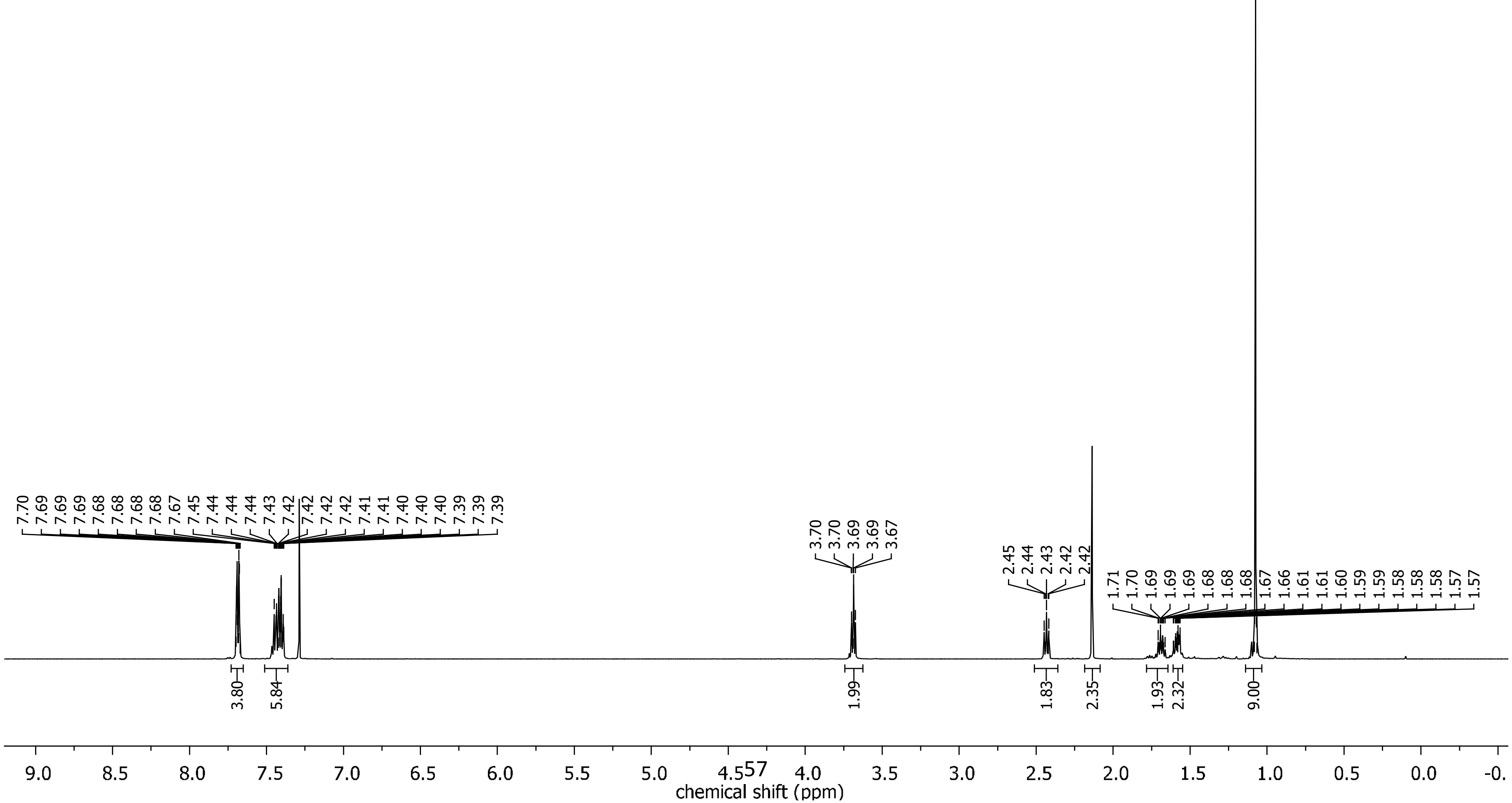

Nucleus: 13C
Frequency: 125.51 MHz
Solvent: CDCl3
Temperature: 298.0 K

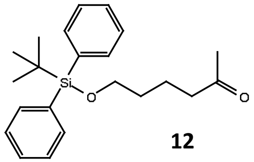

12

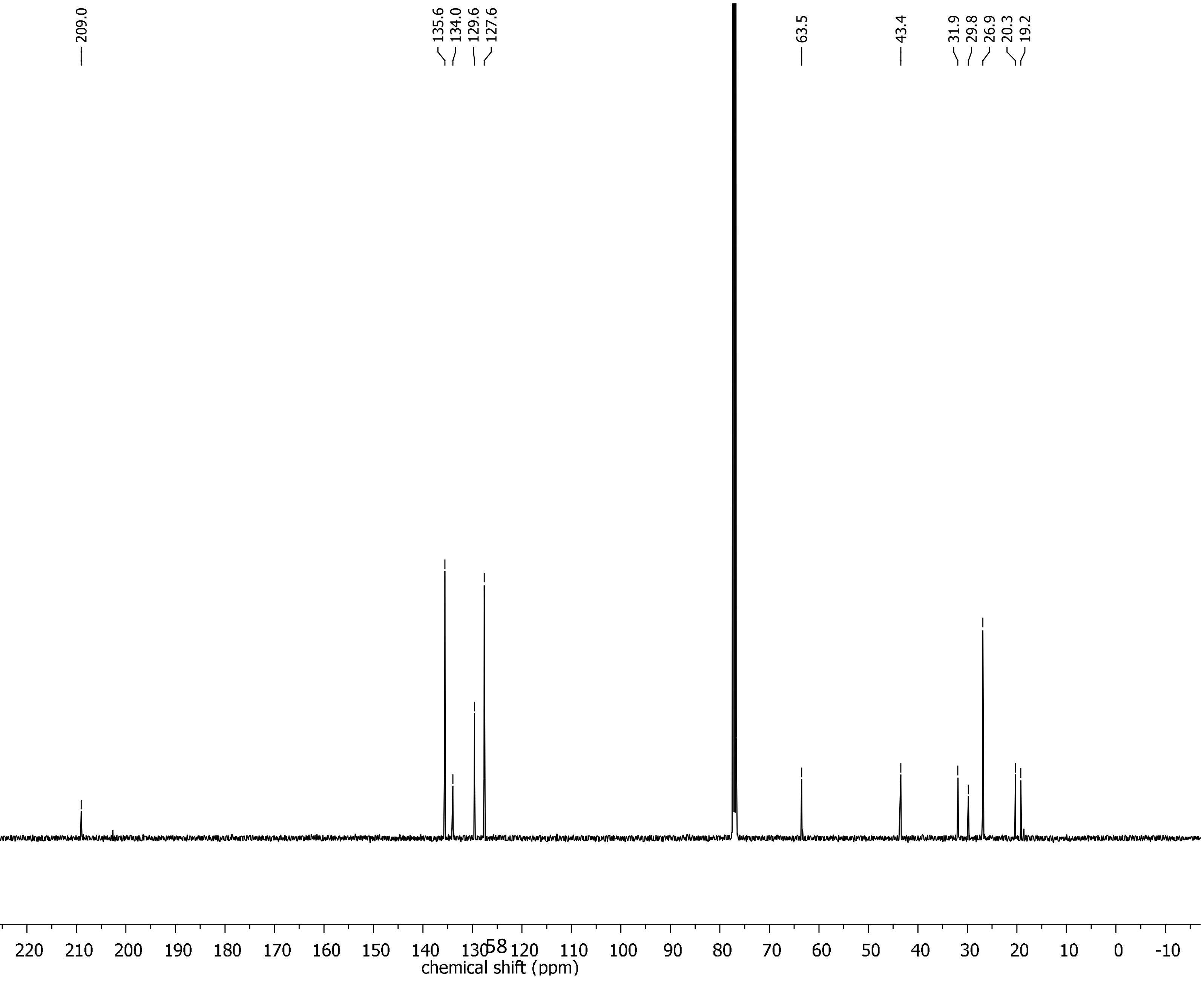

Nucleus: 1H
Frequency: 499.13 MHz
Solvent: CDCl3
Temperature: 297.9 K

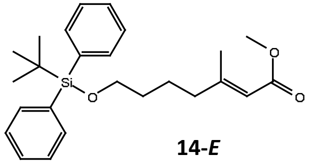

14-*E*

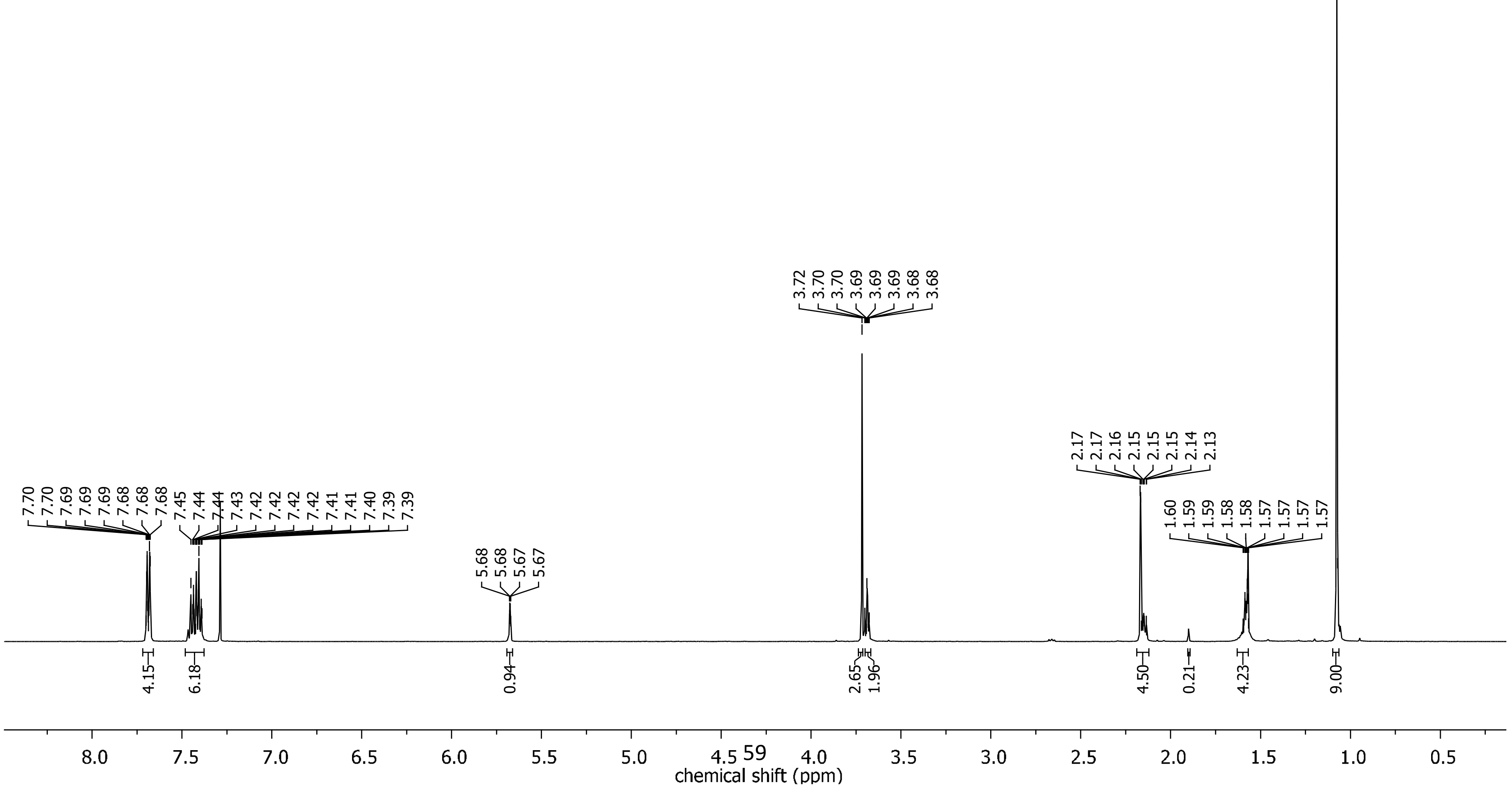

Nucleus: 13C
Frequency: 125.51 MHz
Solvent: CDCl3
Temperature: 298.0 K

14-*E*

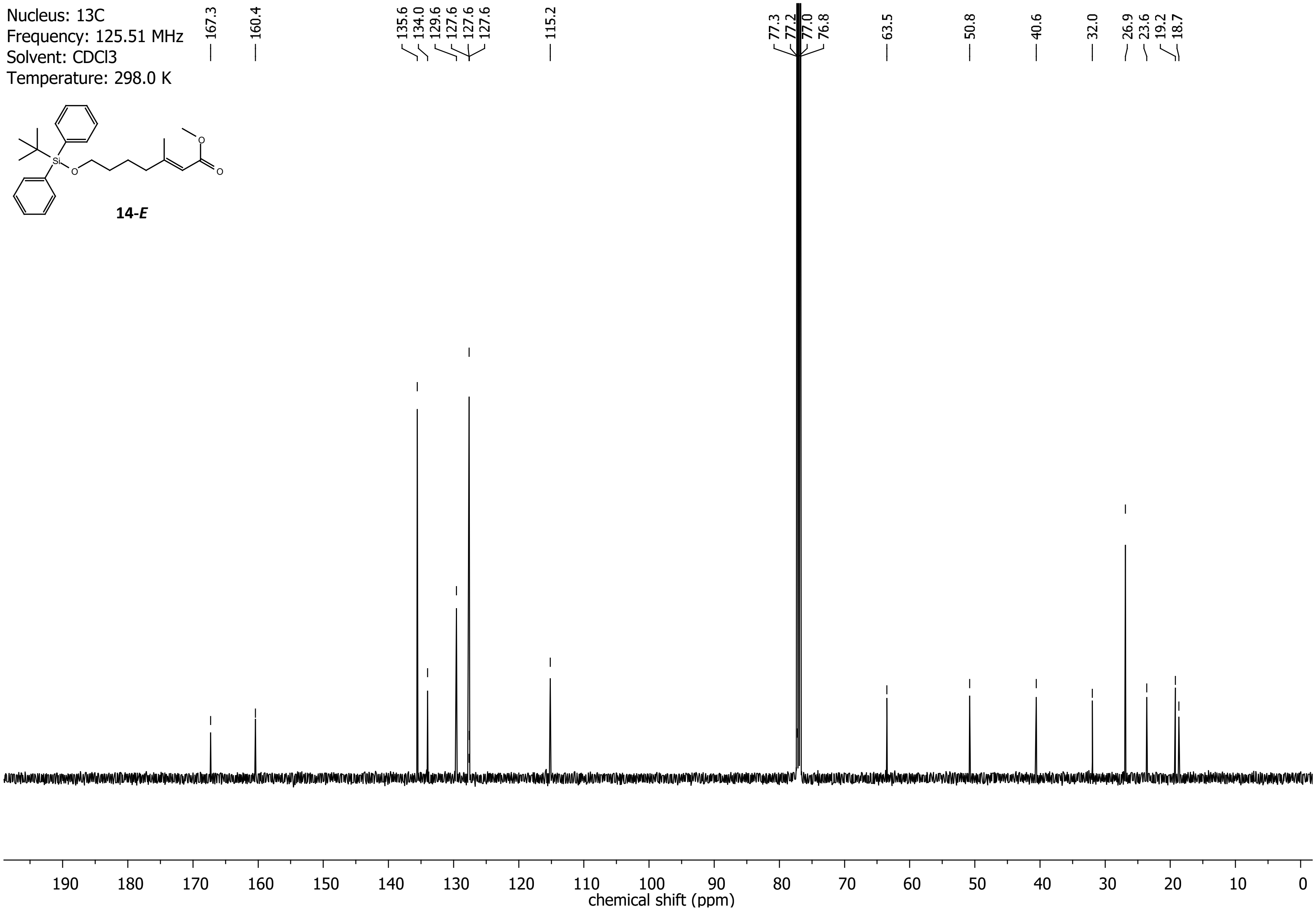

Nucleus: 1H
Frequency: 700.41 MHz
Solvent: CDCl3
Temperature: 298.0 K

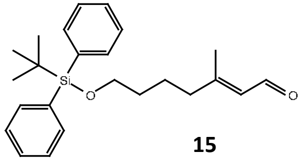

15

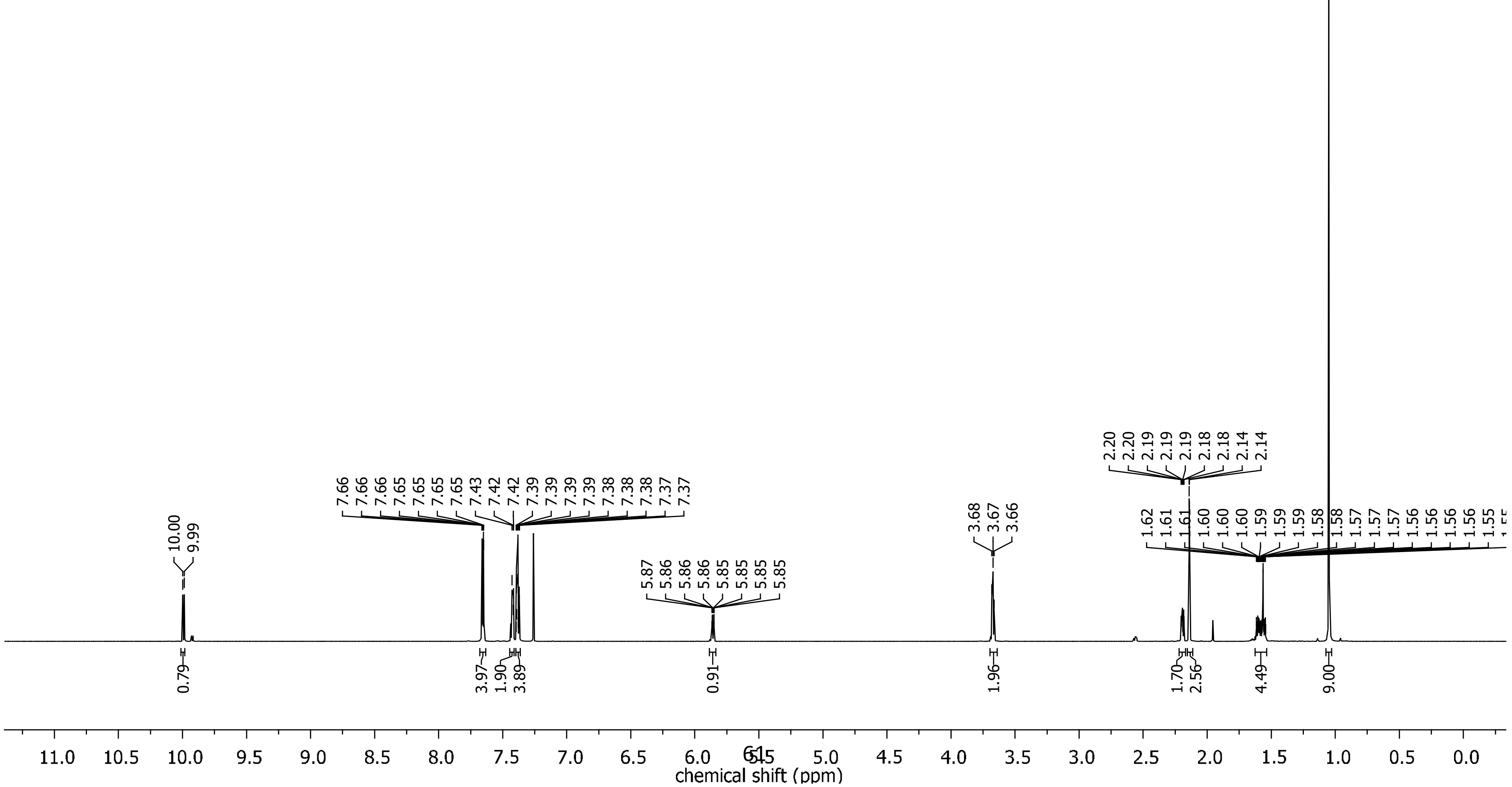

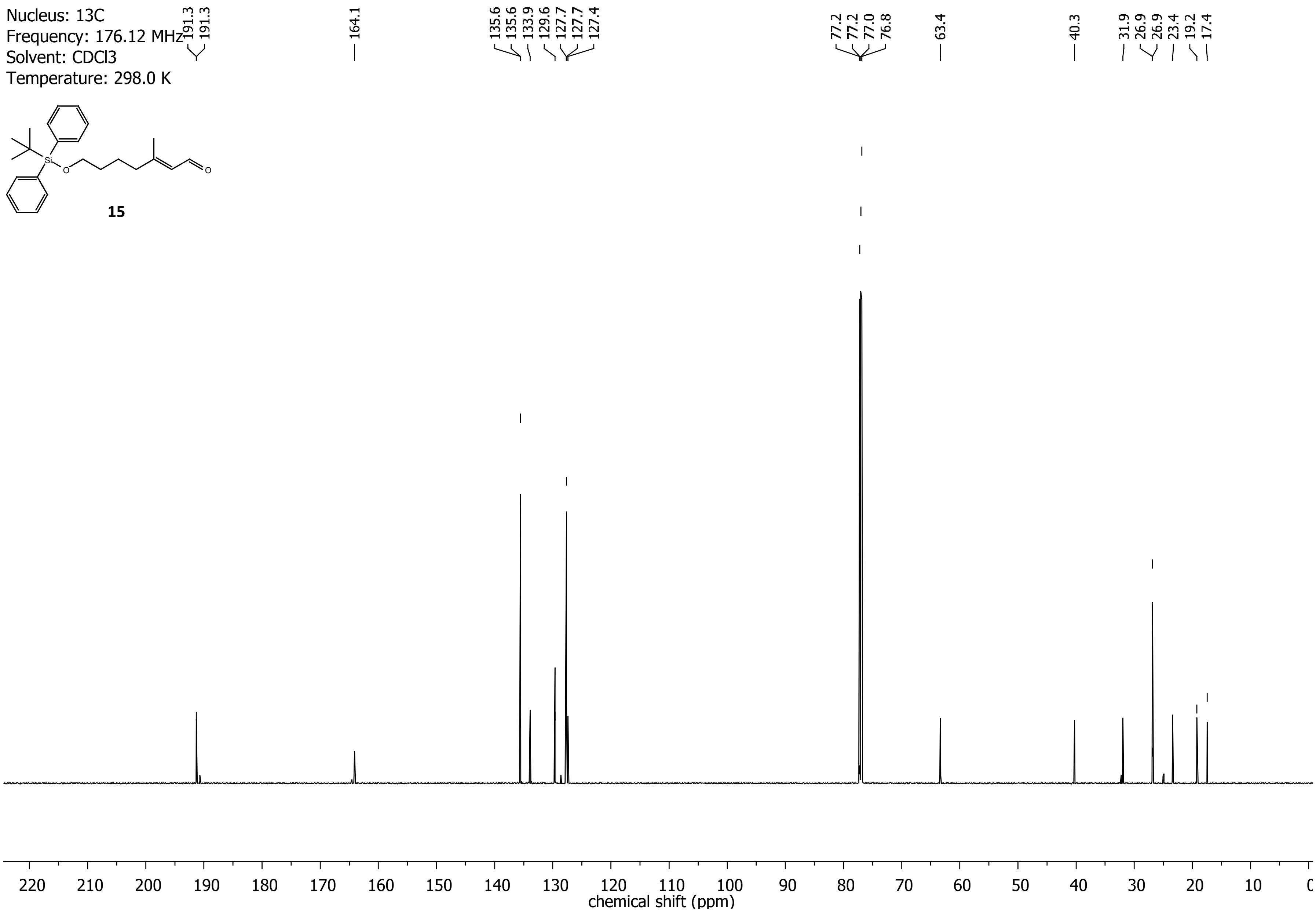
Nucleus: 13C
Frequency: 176.12 MHz
Solvent: CDCl3
Temperature: 298.0 K
191.3
191.3
164.1
135.6
135.6
133.9
129.6
127.7
127.7
127.4
77.2
77.2
77.0
76.8
63.4
40.3
31.9
26.9
26.9
23.4
19.2
17.4
220 210 200 190 180 170 160 150 140 130 120 110 100 90 80 70 60 50 40 30 20 10
chemical shift (ppm)

15

Nucleus: 1H
Frequency: 499.13 MHz
Solvent: CDCl3
Temperature: 297.9 K

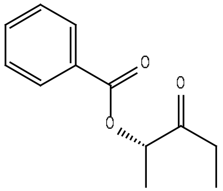

16

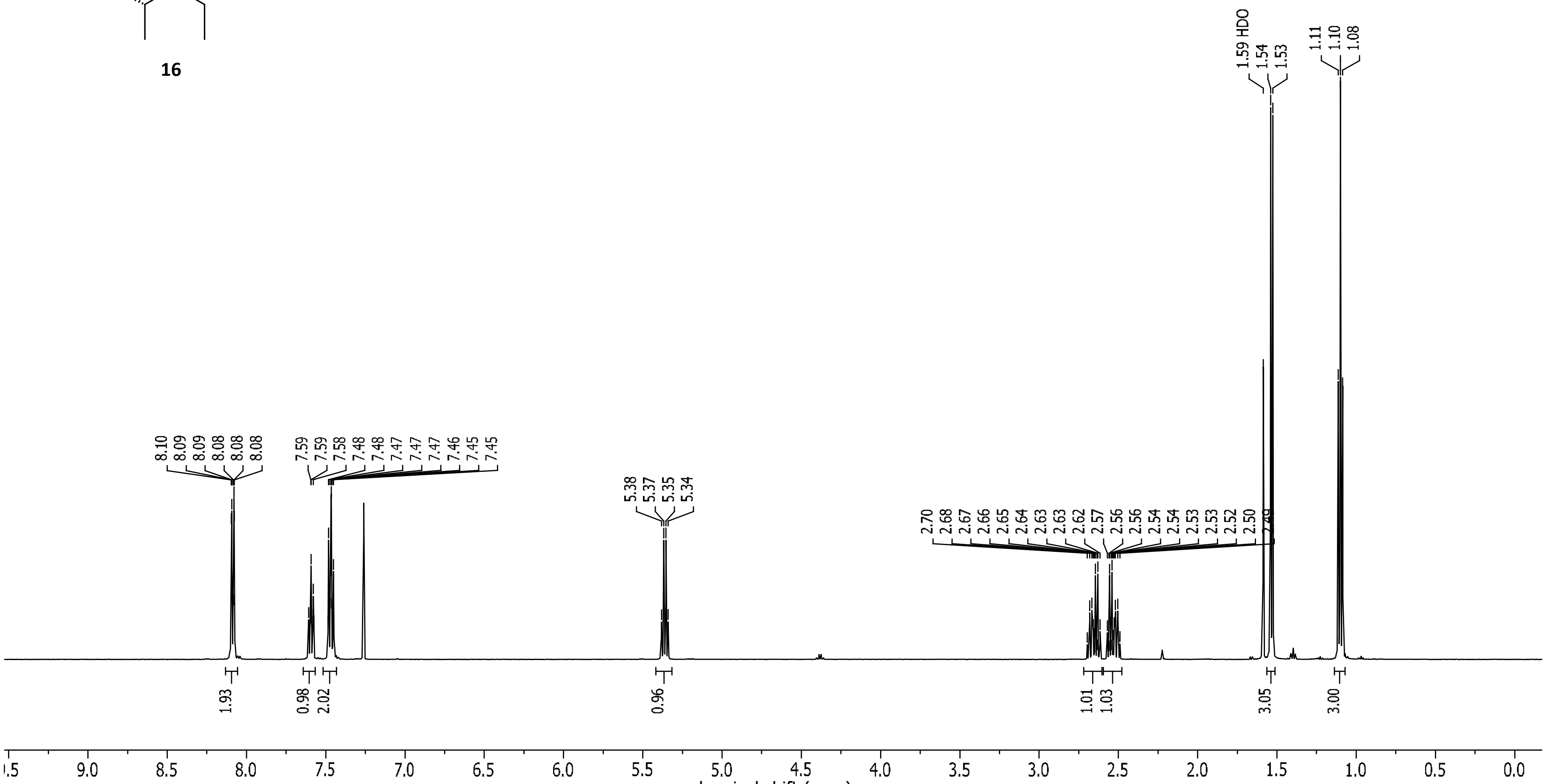

Nucleus: 13C
Frequency: 125.51 MHz
Solvent: CDCl3
Temperature: 298.4 K

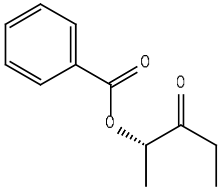

16

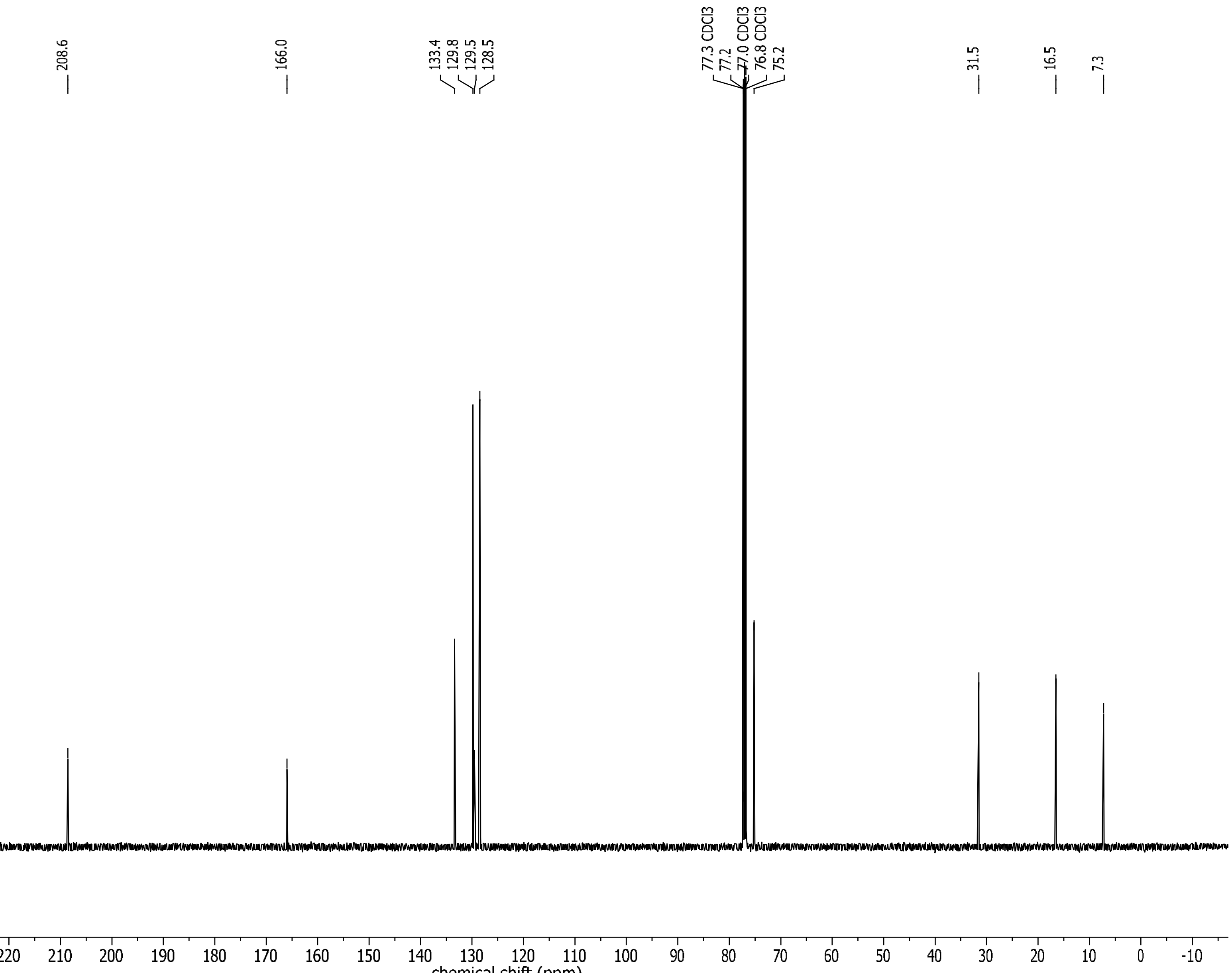

Nucleus: 1H
Frequency: 700.41 MHz
Solvent: CDCl3
Temperature: 298.0 K

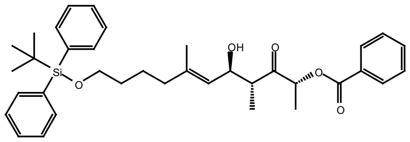

17

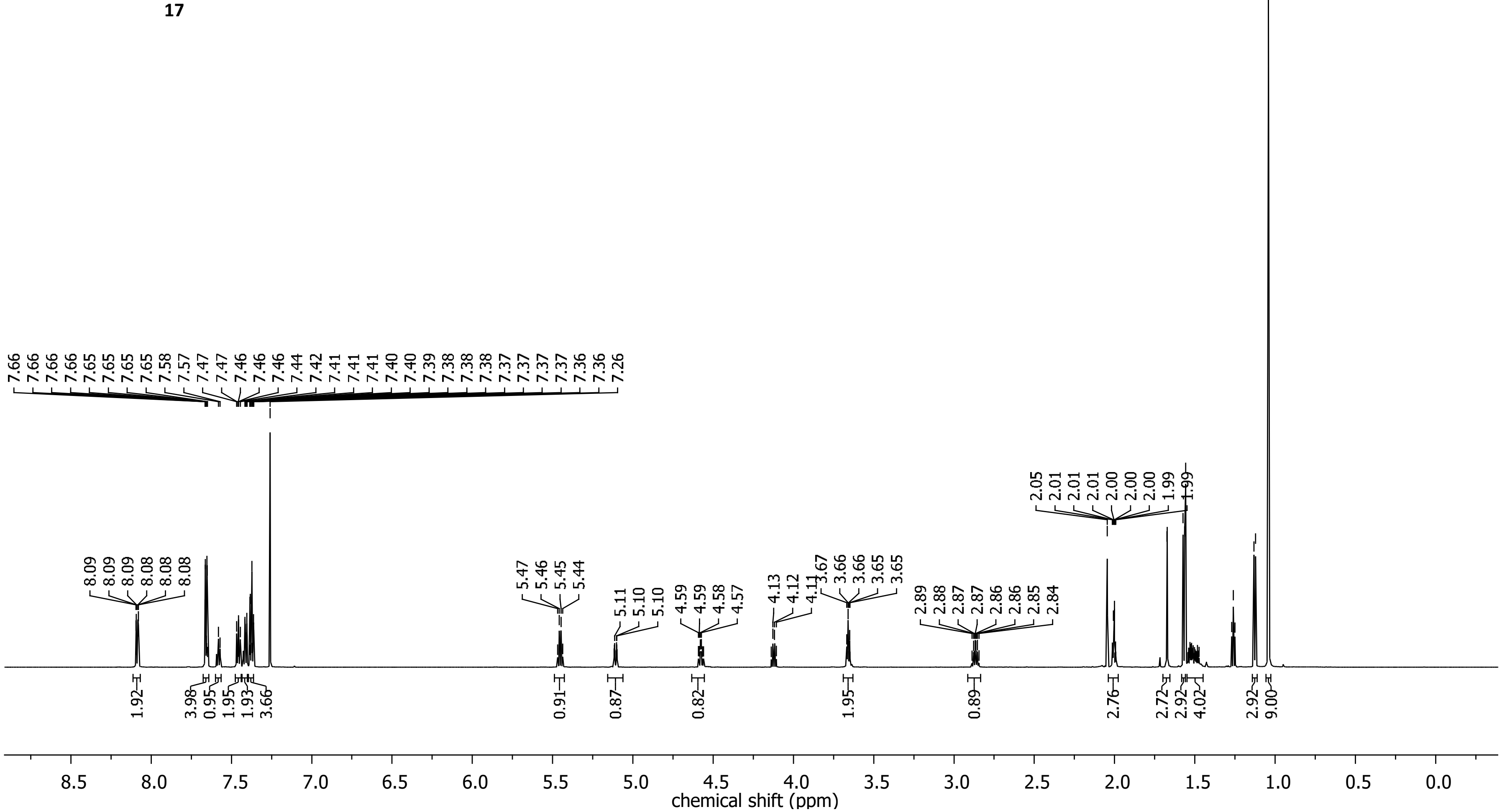

Nucleus: 13C
Frequency: 176.12 MHz
Solvent: CDCl3
Temperature: 298.0 K

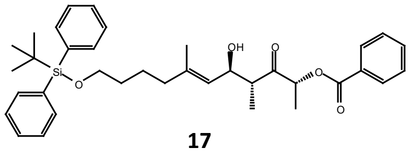

17

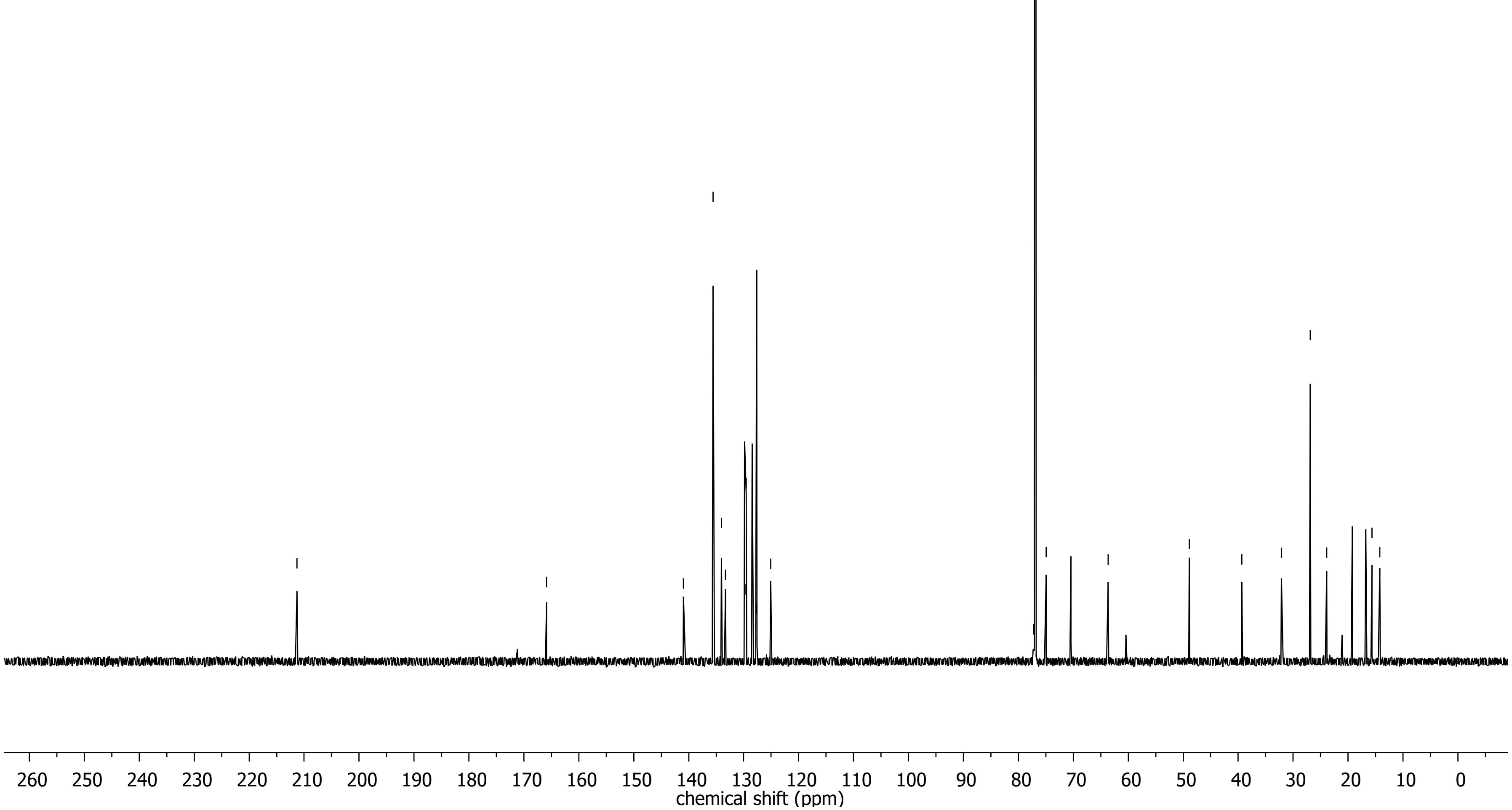

Nucleus: 1H
Frequency: 700.41 MHz
Solvent: CDCl3
Temperature: 298.0 K

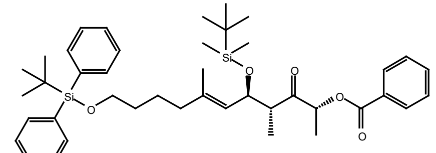

50

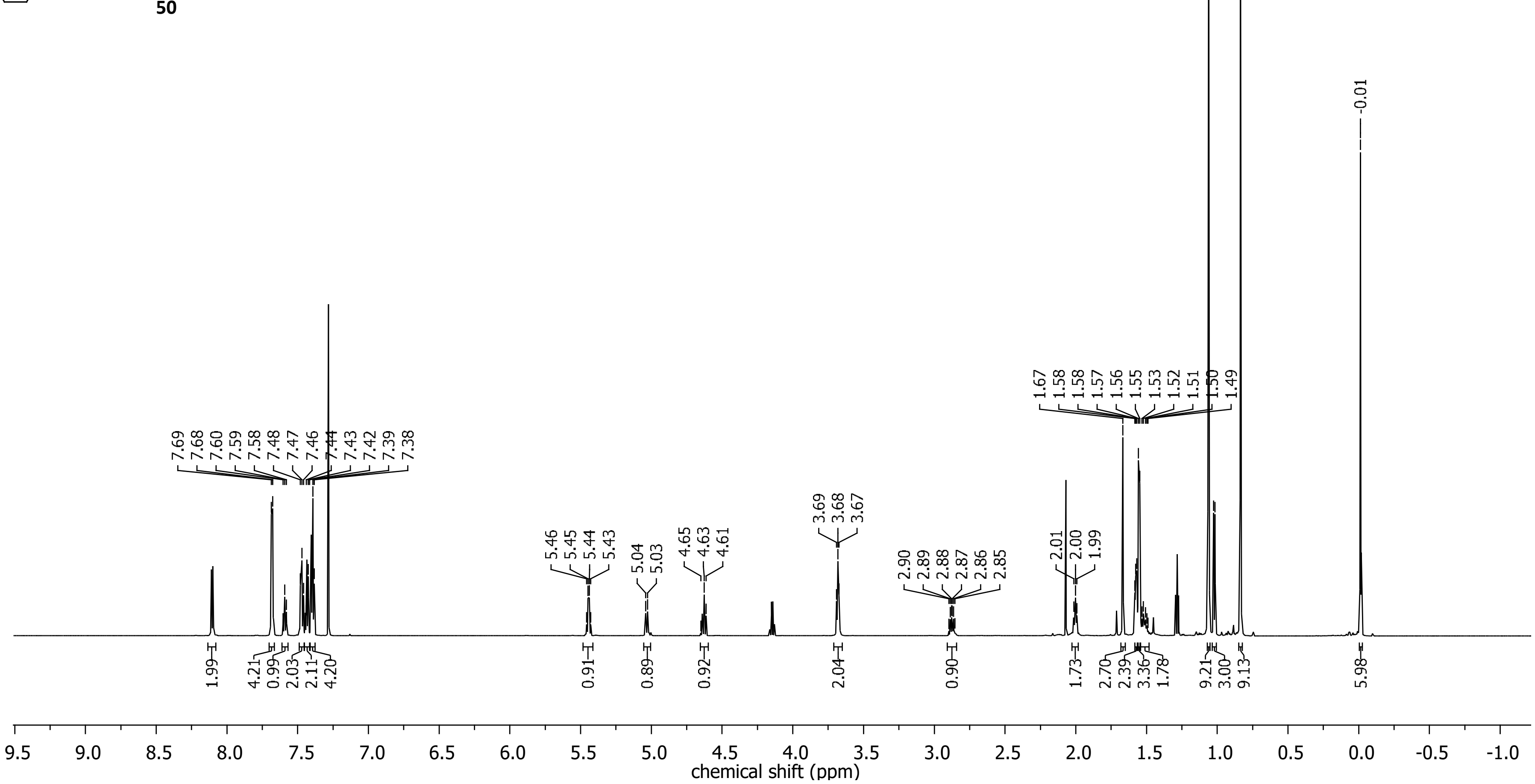

Nucleus: 13C
Frequency: 176.12 MHz
Solvent: CDCl3
Temperature: 298.0 K

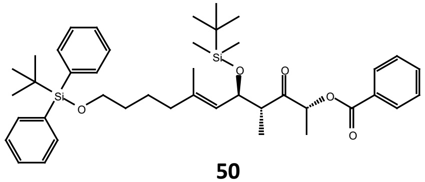

50

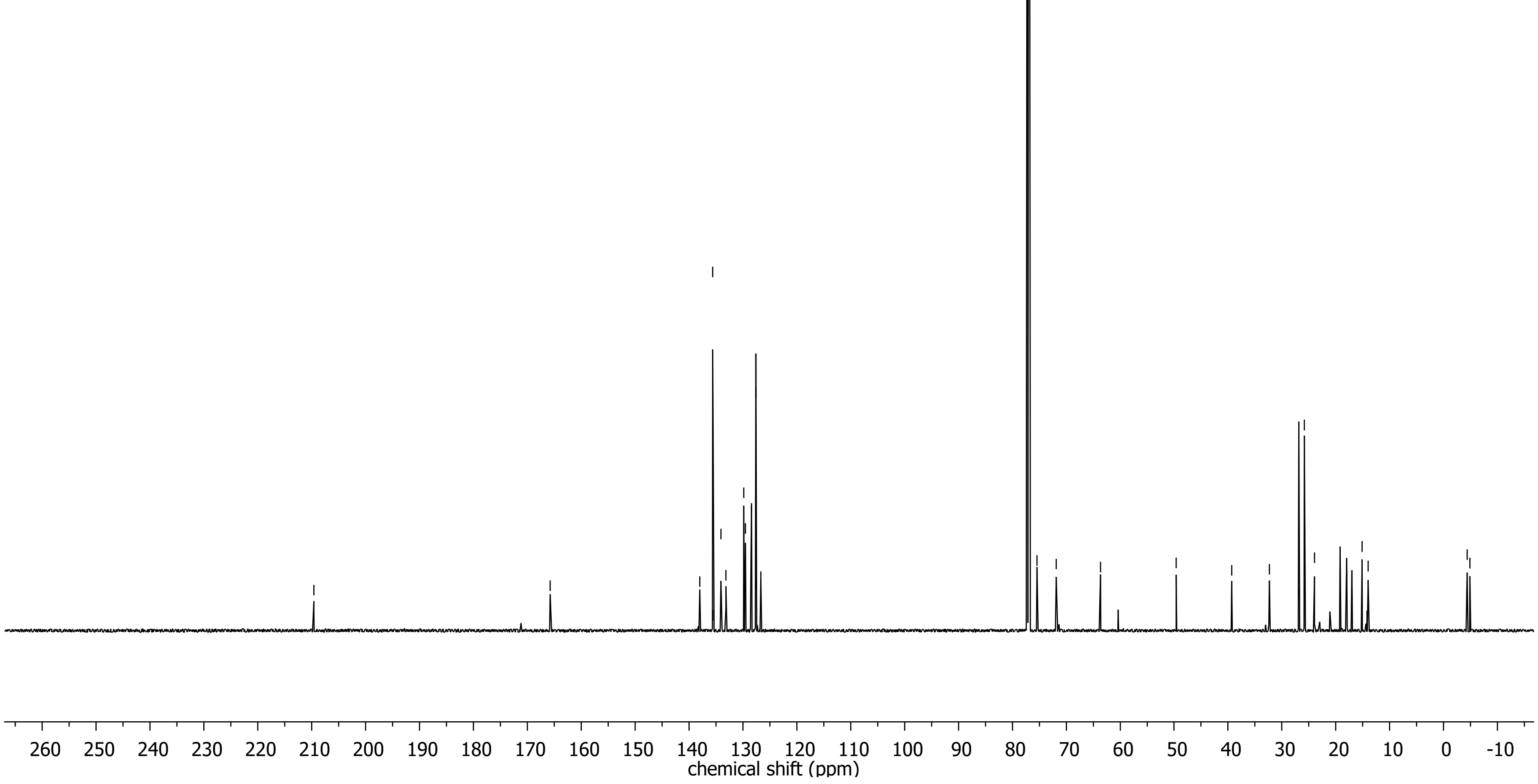

Nucleus: 1H
Frequency: 700.41 MHz
Solvent: CD2Cl2
Temperature: 297.0 K

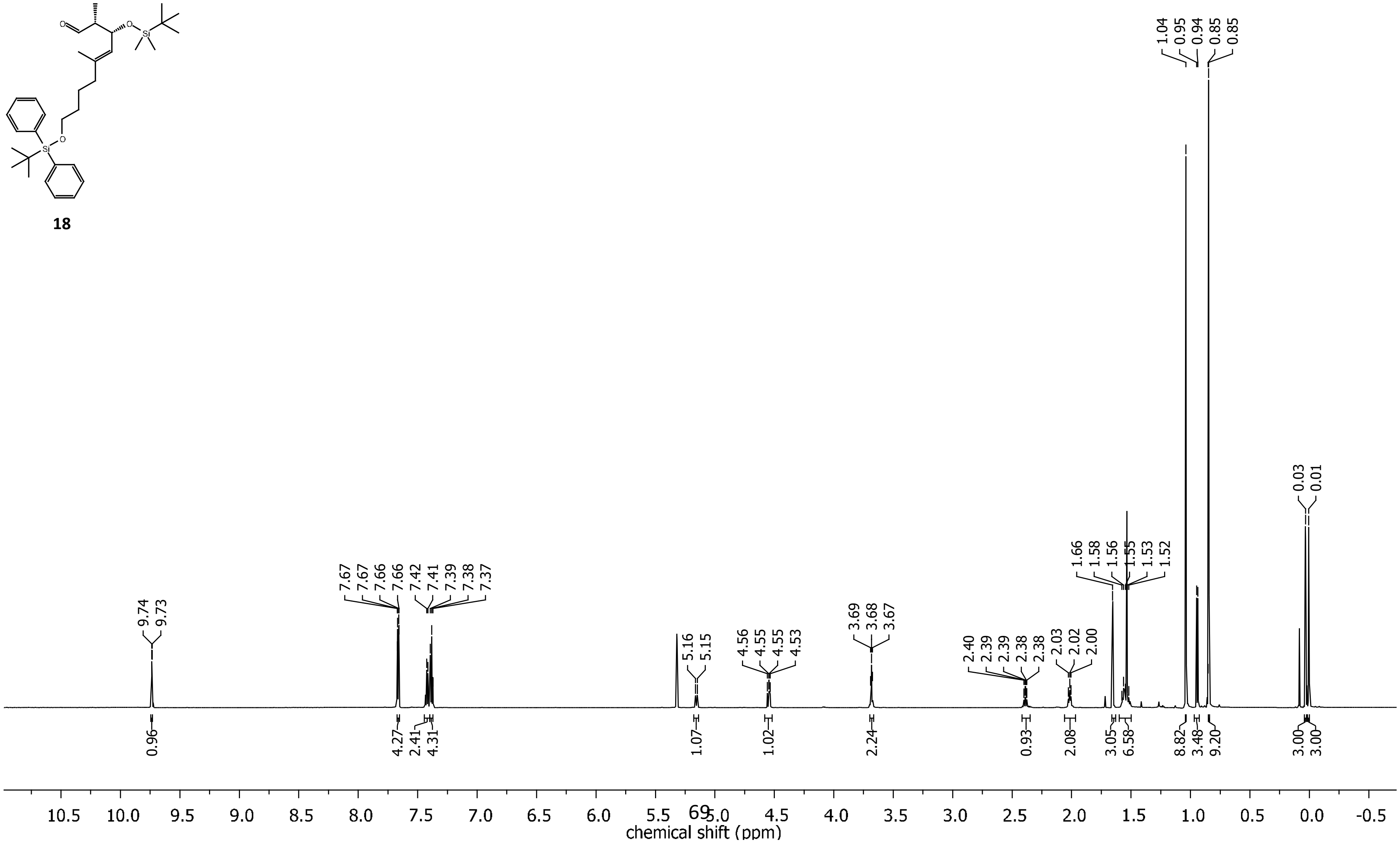

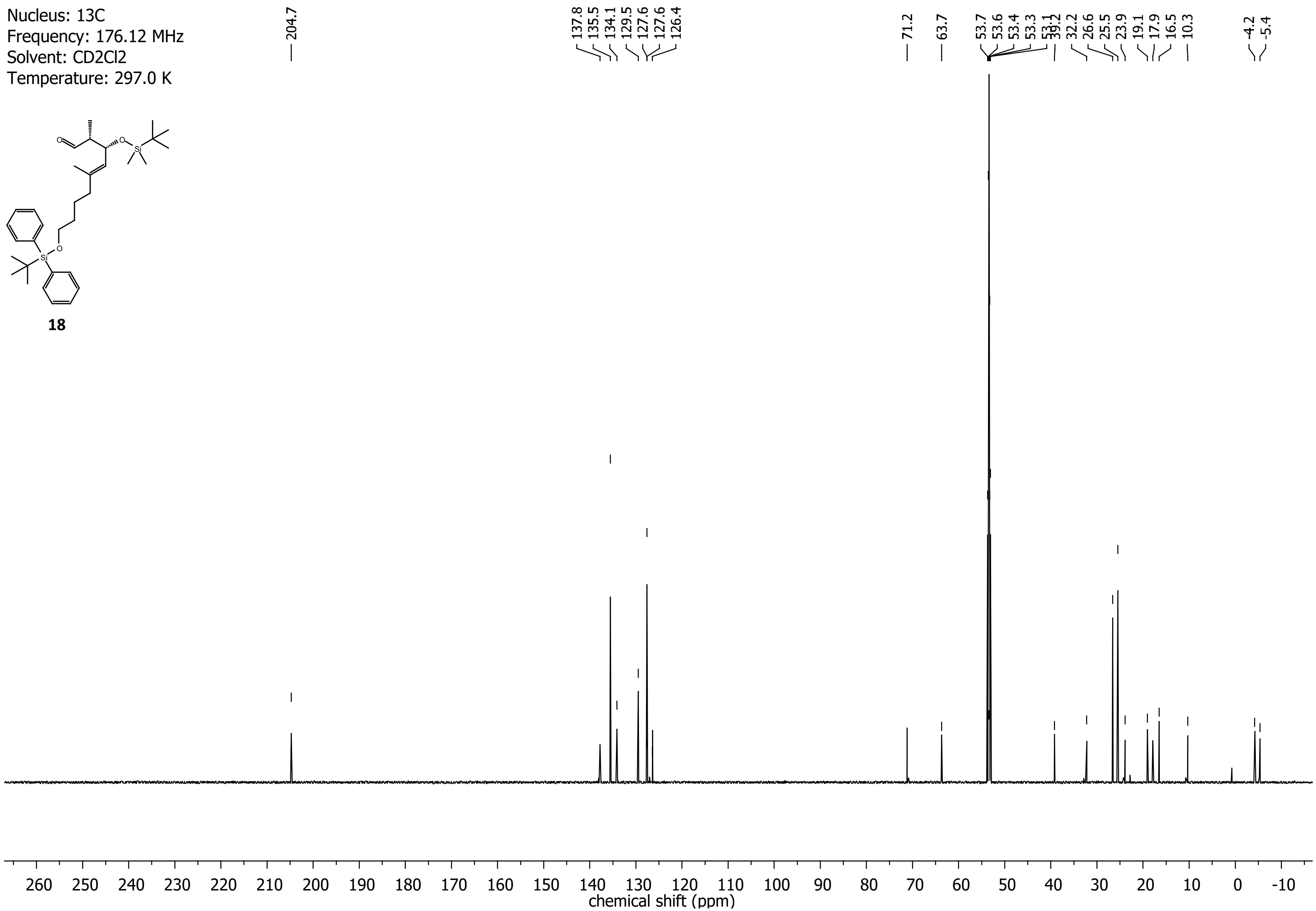
Nucleus: 13C
Frequency: 176.12 MHz
Solvent: CD2Cl2
Temperature: 297.0 K
18
204.7
137.8
135.5
134.1
129.5
127.6
127.6
126.4
71.2
63.7
53.7
53.6
53.4
53.3
53.1
39.2
32.2
26.6
25.5
23.9
19.1
17.9
16.5
10.3
-4.2
-5.4
260 250 240 230 220 210 200 190 180 170 160 150 140 130 120 110 100 90 80 70 60 50 40 30 20 10 0 -10
chemical shift (ppm)

Nucleus: 1H
Frequency: 300.13 MHz
Solvent: CDCl3
Temperature: 298.0 K

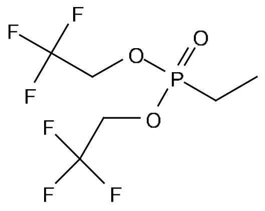

52

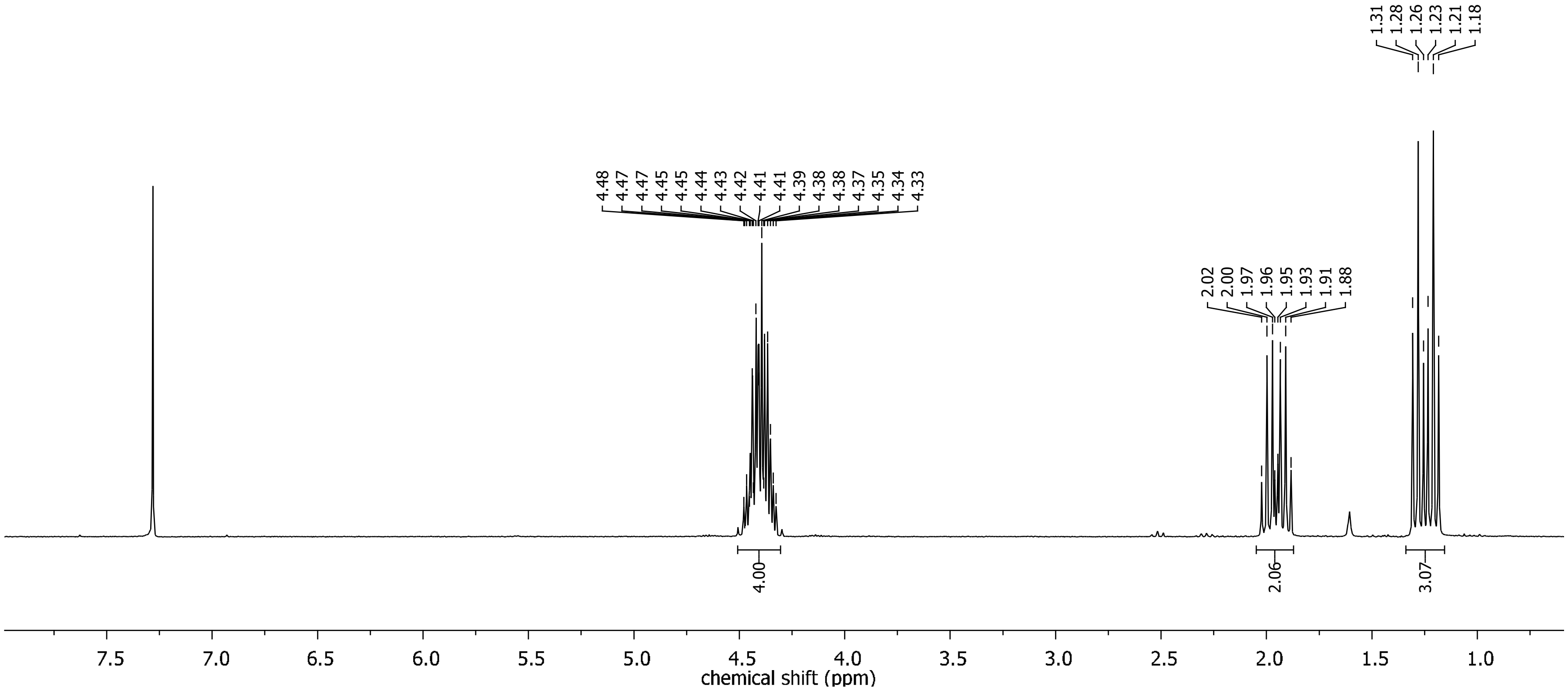

Nucleus: 13C
Frequency: 125.51 MHz
Solvent: CDCl3
Temperature: 297.9 K

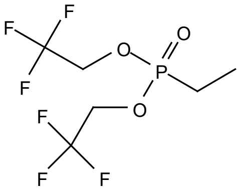

52

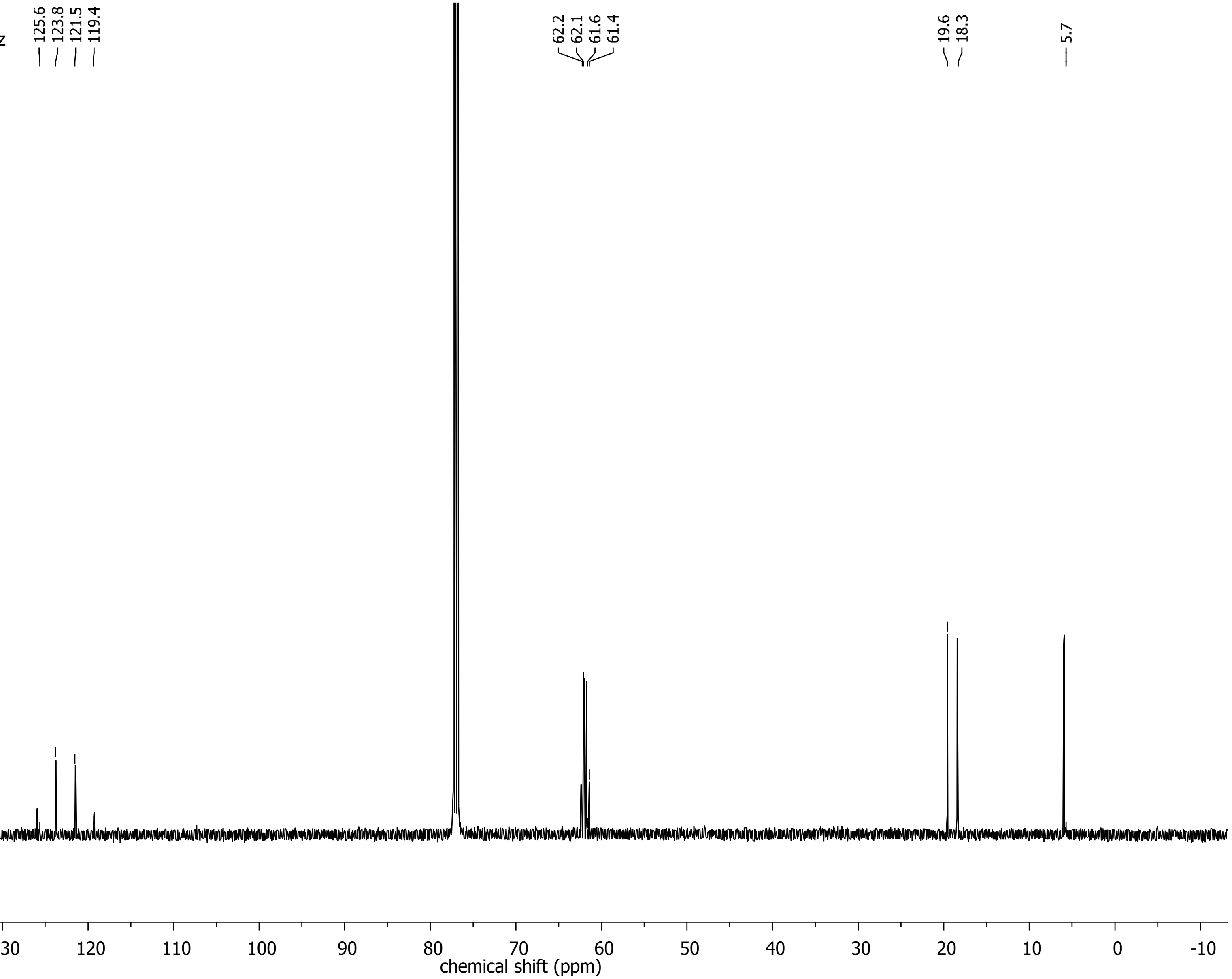

Nucleus: 31P
Frequency: 202.05 MHz
Solvent: CDCl3
Temperature: 297.9 K

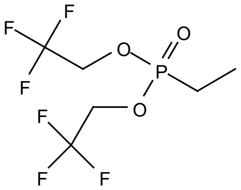

52

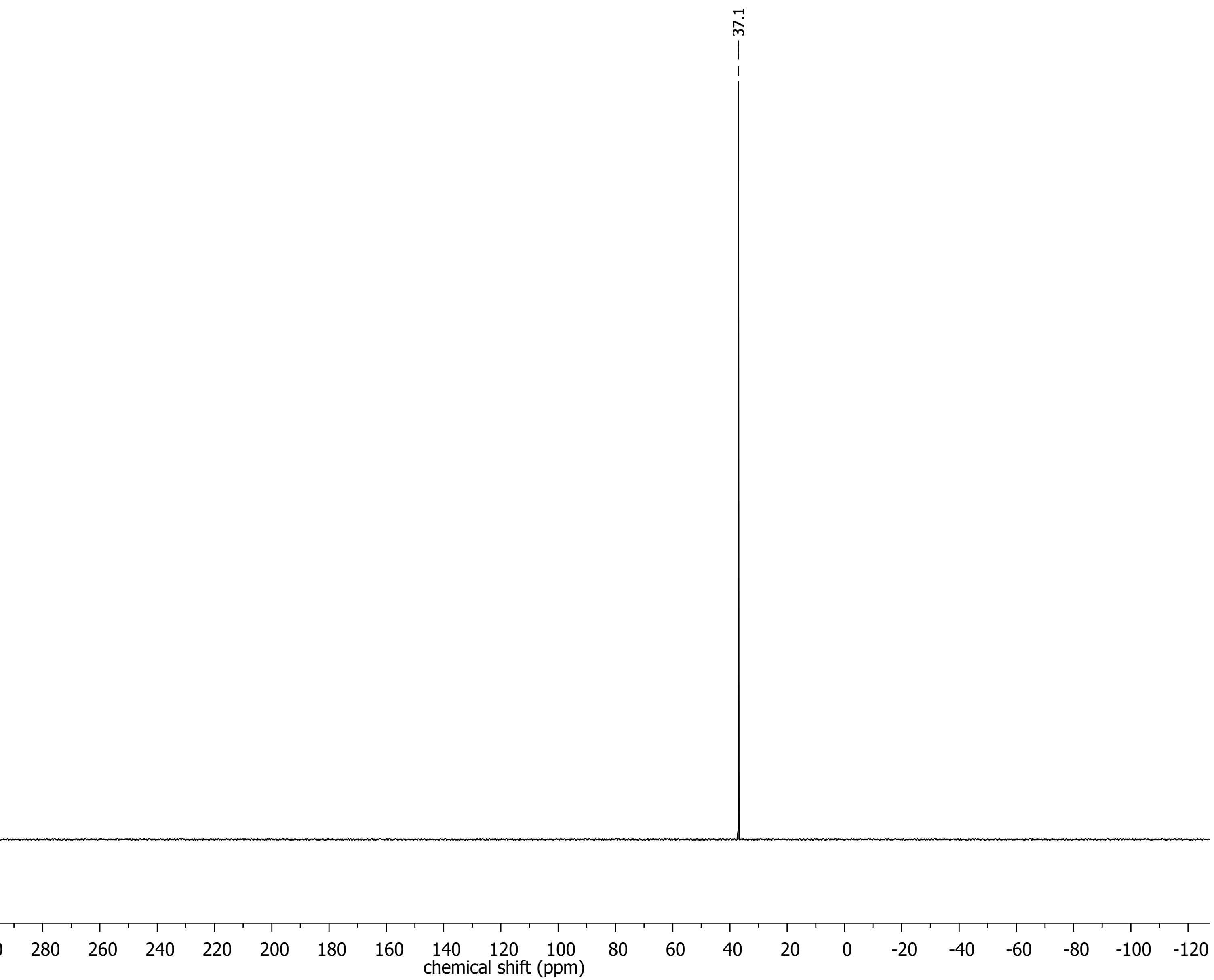

Nucleus: 1H
Frequency: 400.13 MHz
Solvent: CDCl3
Temperature: 298.0 K

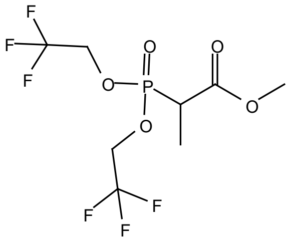

19

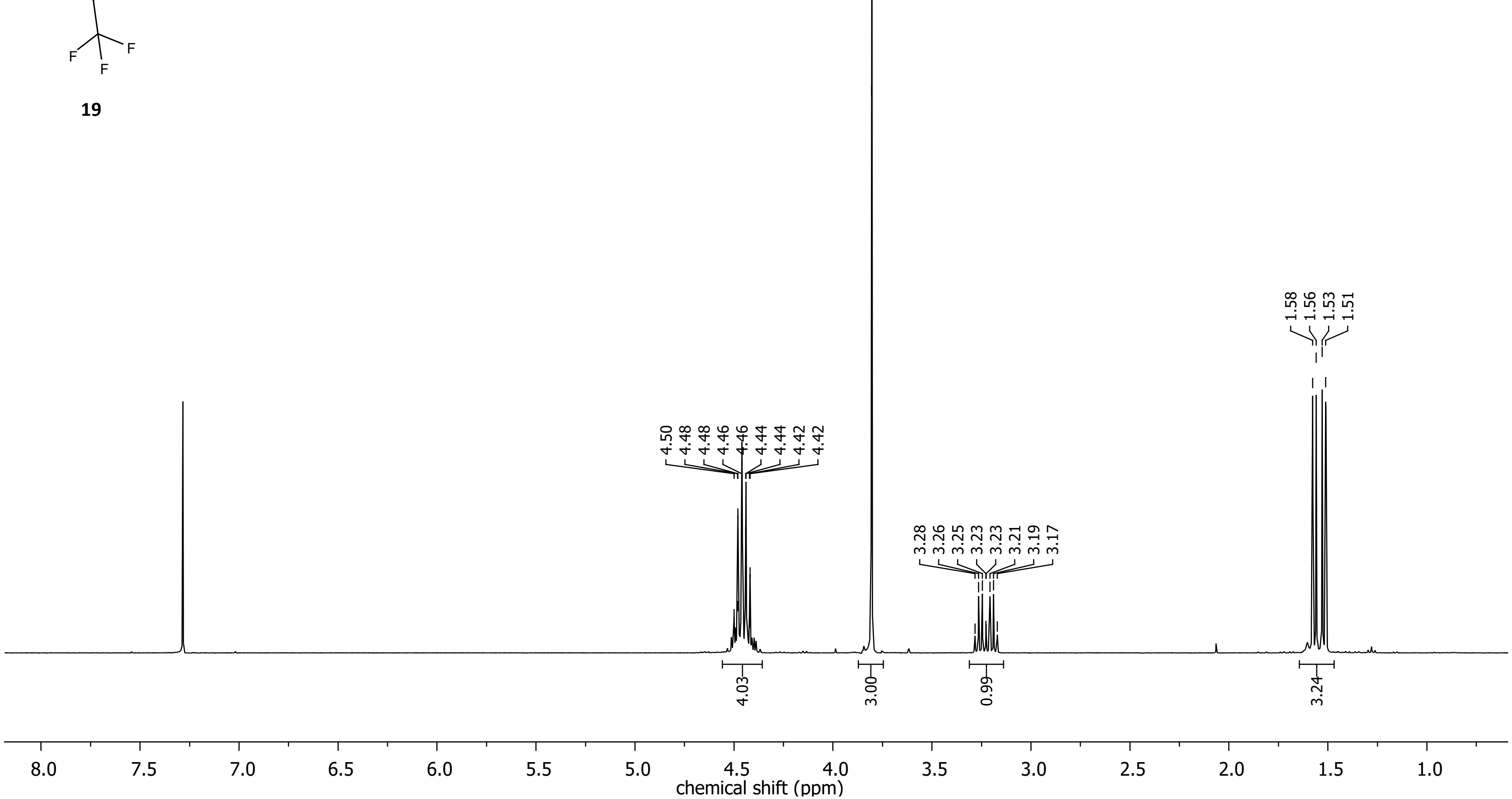

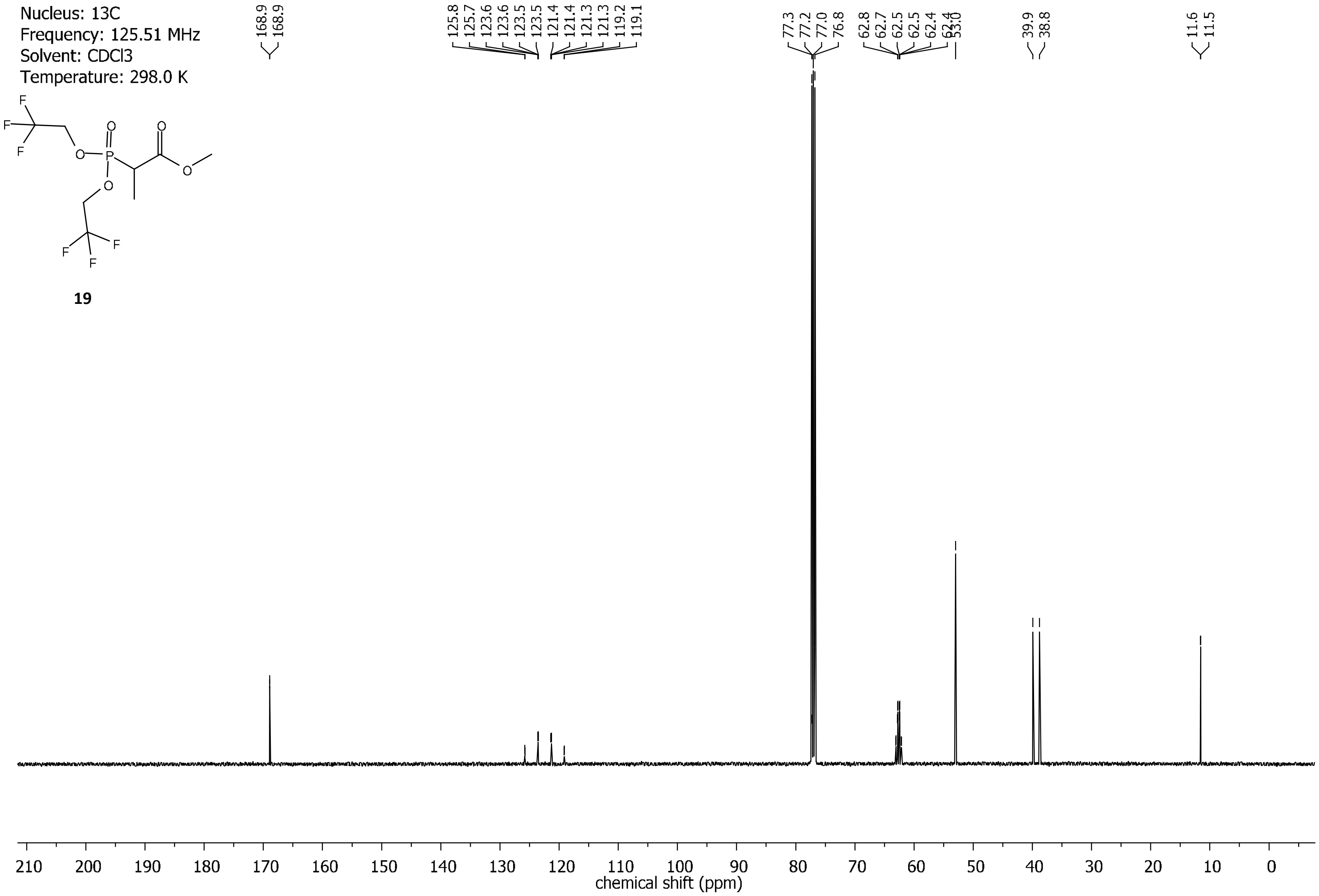
Nucleus: 13C
Frequency: 125.51 MHz
Solvent: CDCl3
Temperature: 298.0 K
168.9
168.9
125.8
125.7
123.6
123.6
123.5
123.5
121.4
121.4
121.3
121.3
119.2
119.1
77.3
77.2
77.0
76.8
62.8
62.7
62.5
62.5
62.4
62.4
53.0
39.9
38.8
11.6
11.5
210 200 190 180 170 160 150 140 130 120 110 100 90 80 70 60 50 40 30 20 10 0
chemical shift (ppm)

F
F
F
O
P
O
O
O
O
F
F
F

19

Nucleus: 31P
Frequency: 202.05 MHz
Solvent: CDCl3
Temperature: 297.9 K

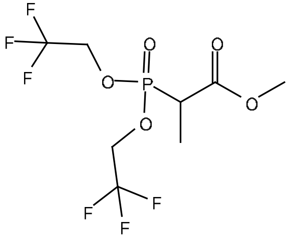

19

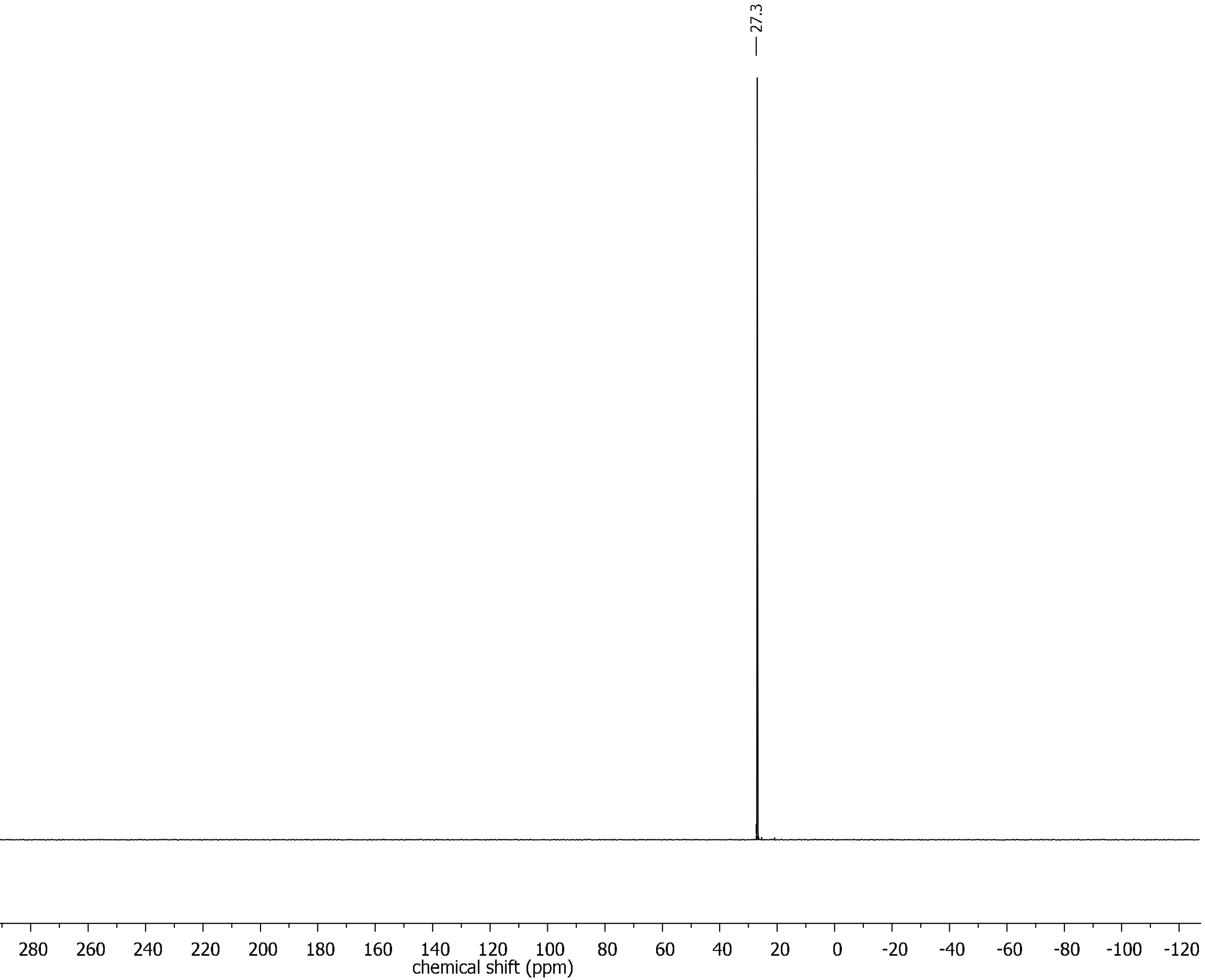

Nucleus: 1H
Frequency: 500.14 MHz
Solvent: CDCl3
Temperature: 298.0 K

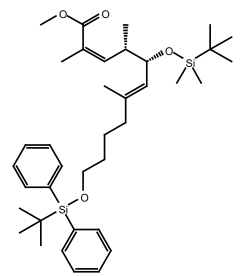

20

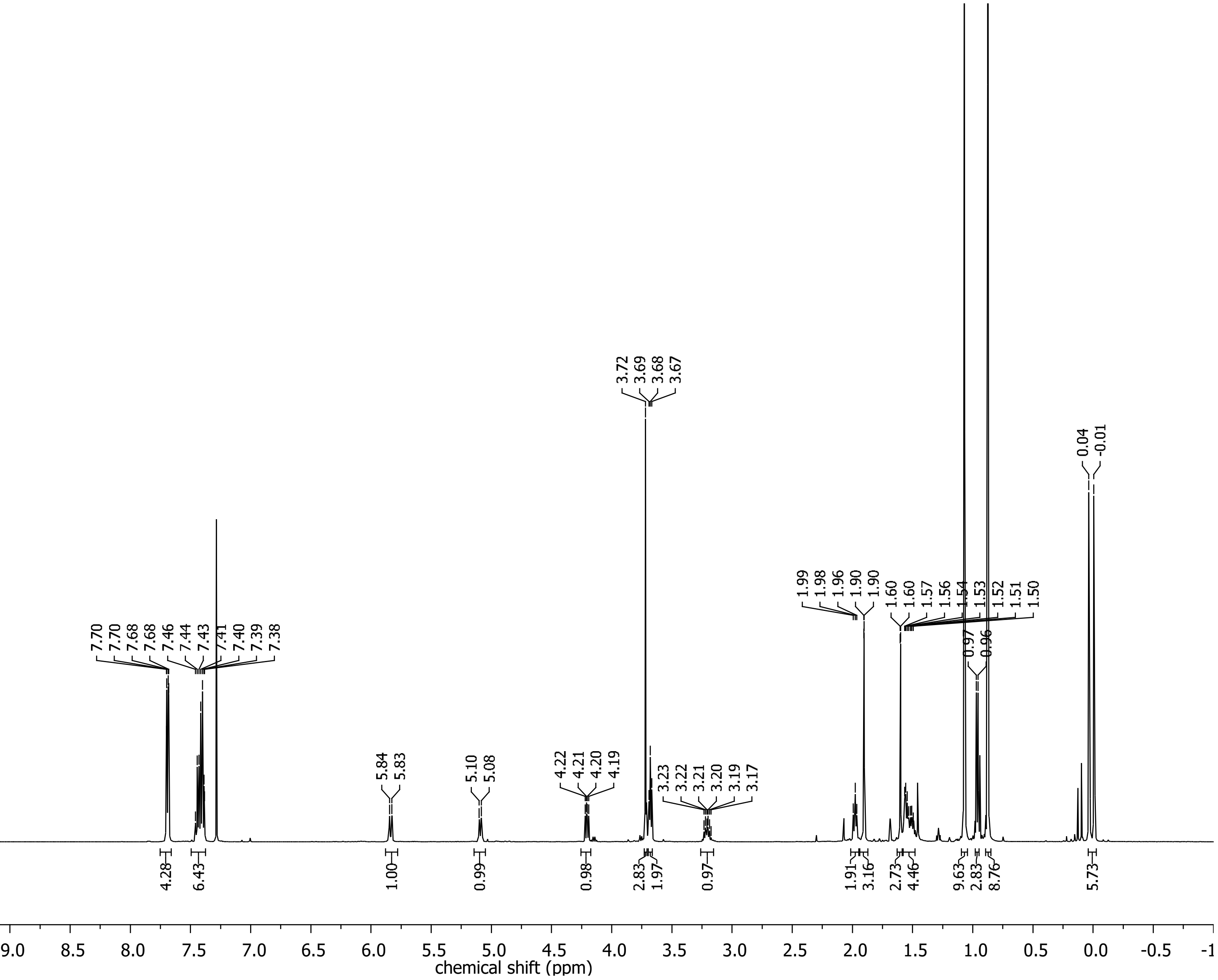

Nucleus: 13C
Frequency: 125.76 MHz
Solvent: CDCl3
Temperature: 298.0 K

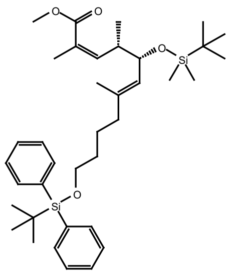

20

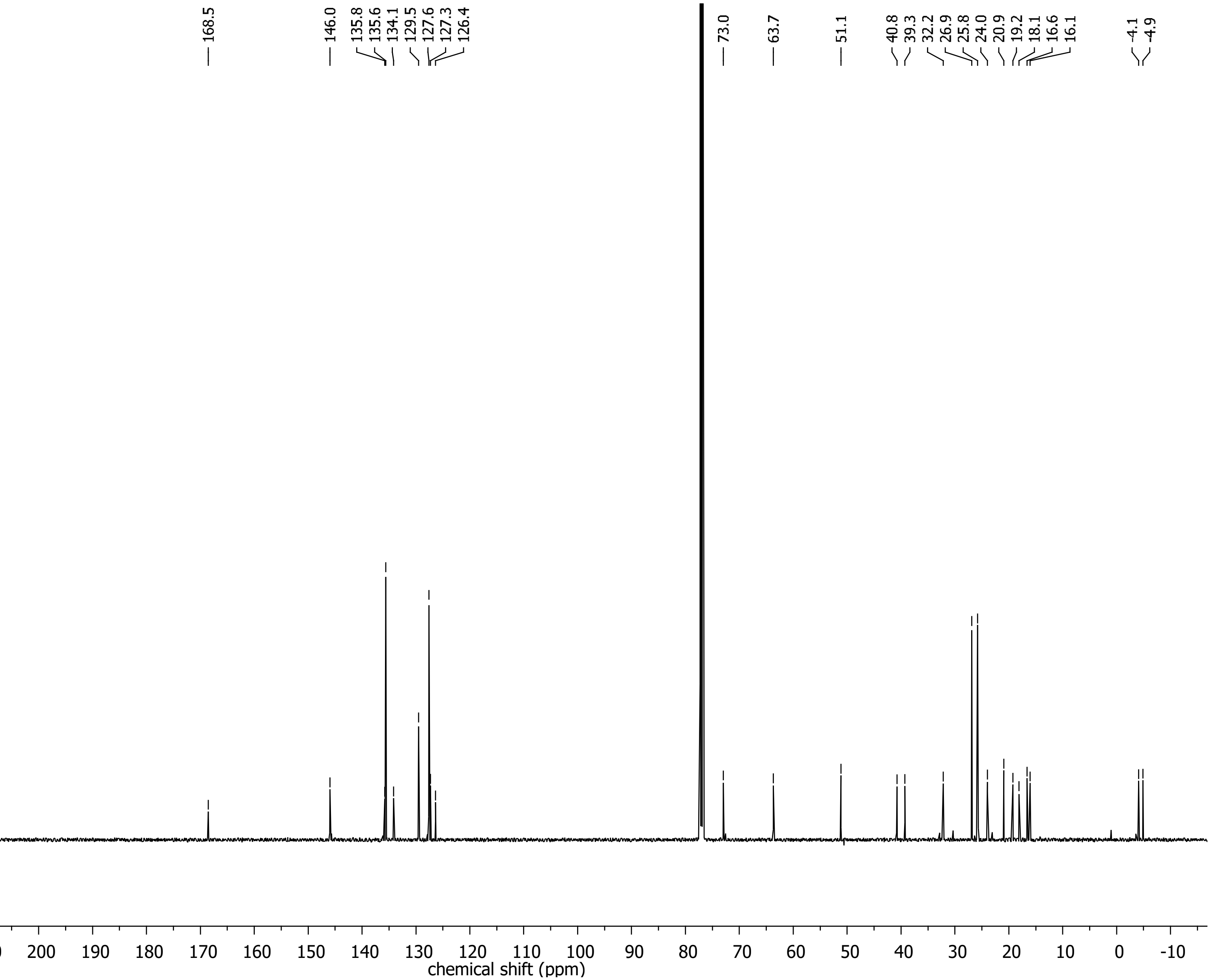

Nucleus: 1H
Frequency: 500.14 MHz
Solvent: CDCl3
Temperature: 298.0 K

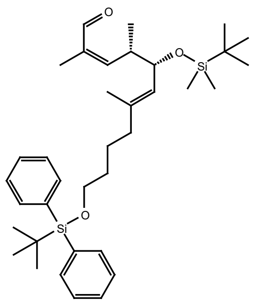

21

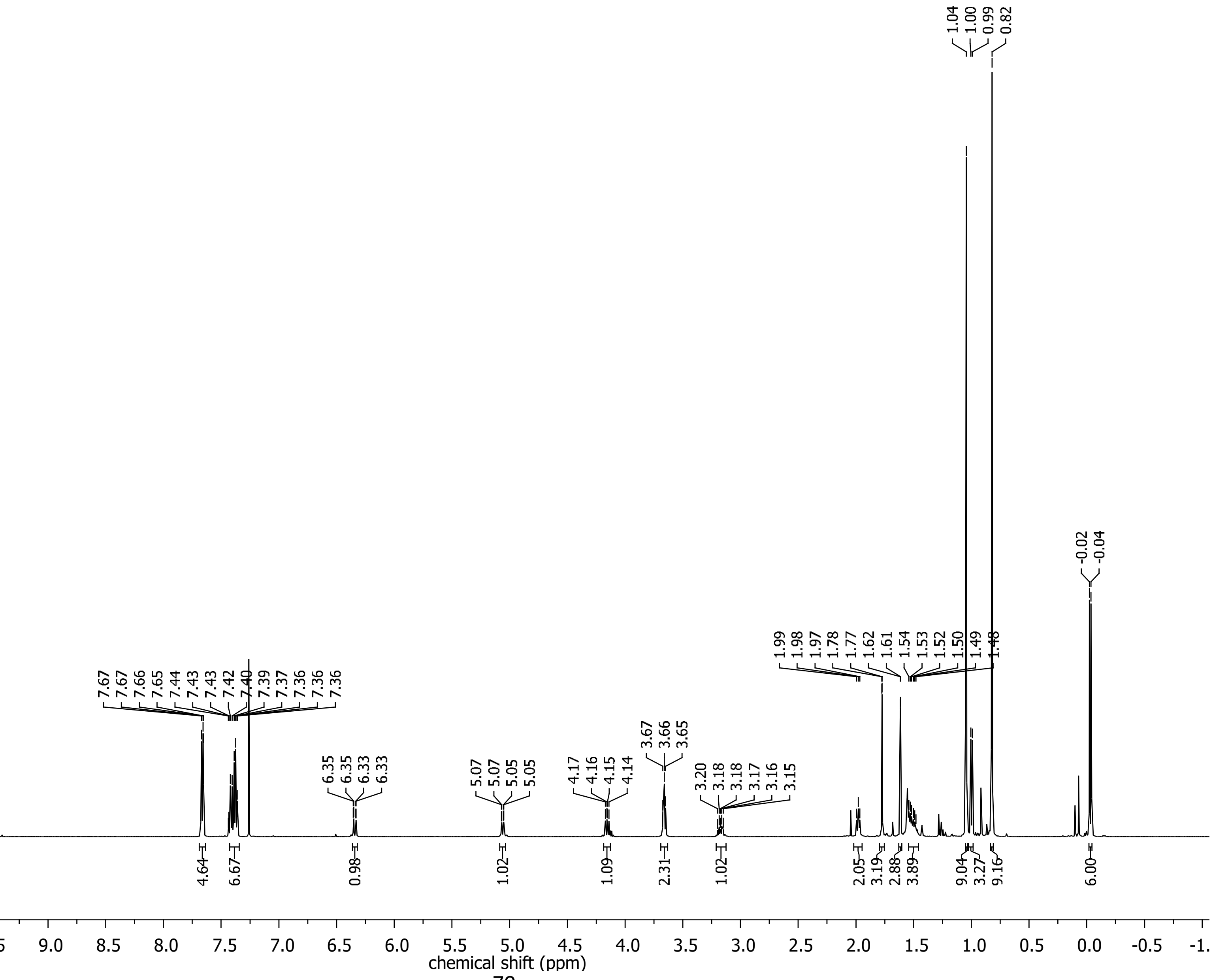

Nucleus: 13C
Frequency: 125.76 MHz
Solvent: CDCl3
Temperature: 298.0 K

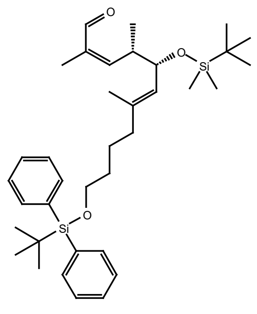

21

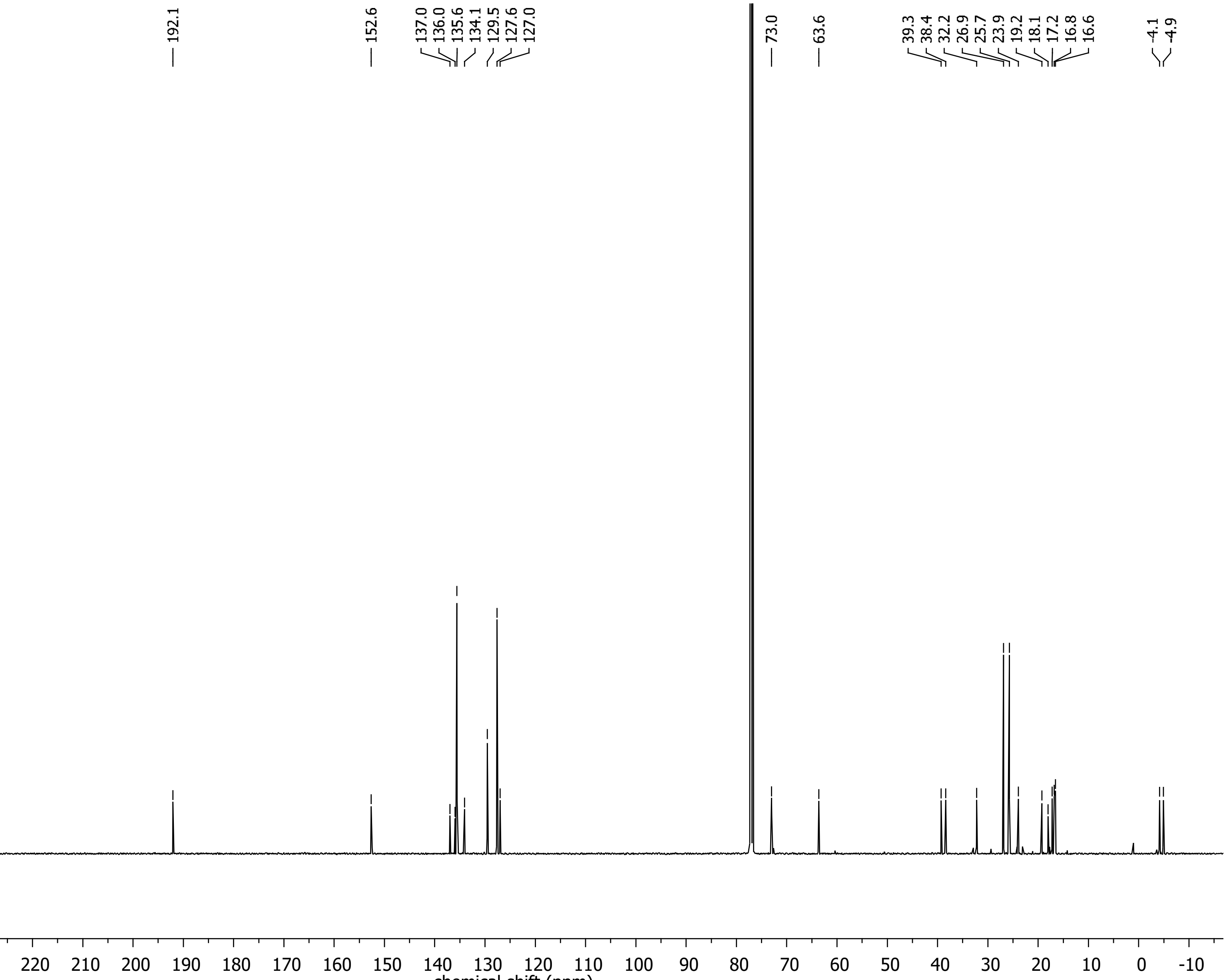

Nucleus: 1H
Frequency: 500.14 MHz
Solvent: CDCl3
Temperature: 298.0 K

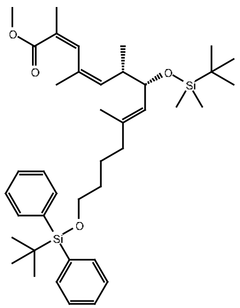

22

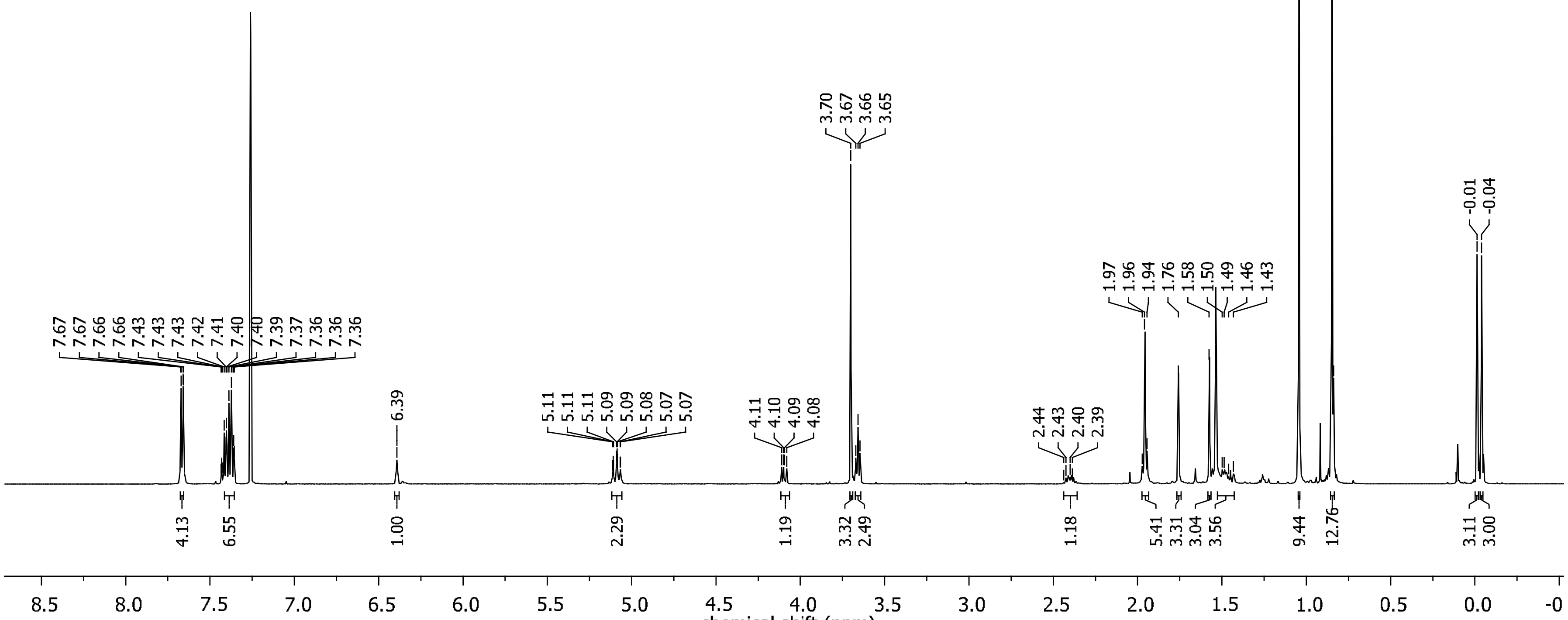

Nucleus: 13C
Frequency: 125.76 MHz
Solvent: CDCl3
Temperature: 298.0 K

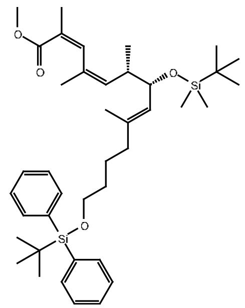

22

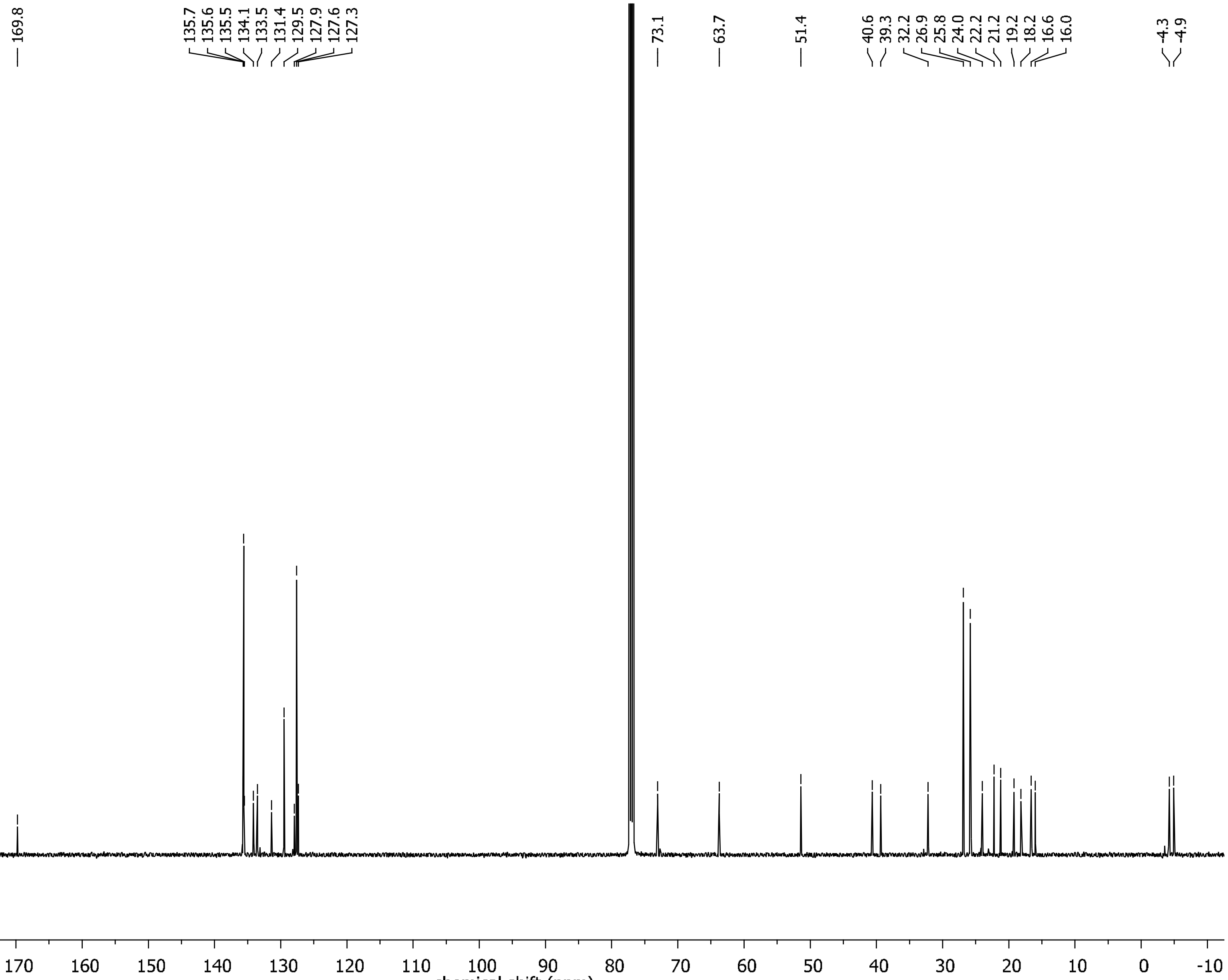

Nucleus: 1H
Frequency: 500.14 MHz
Solvent: CDCl3
Temperature: 298.0 K

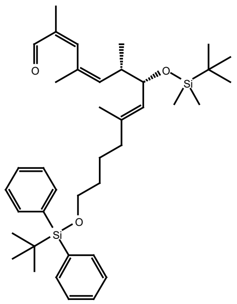

23

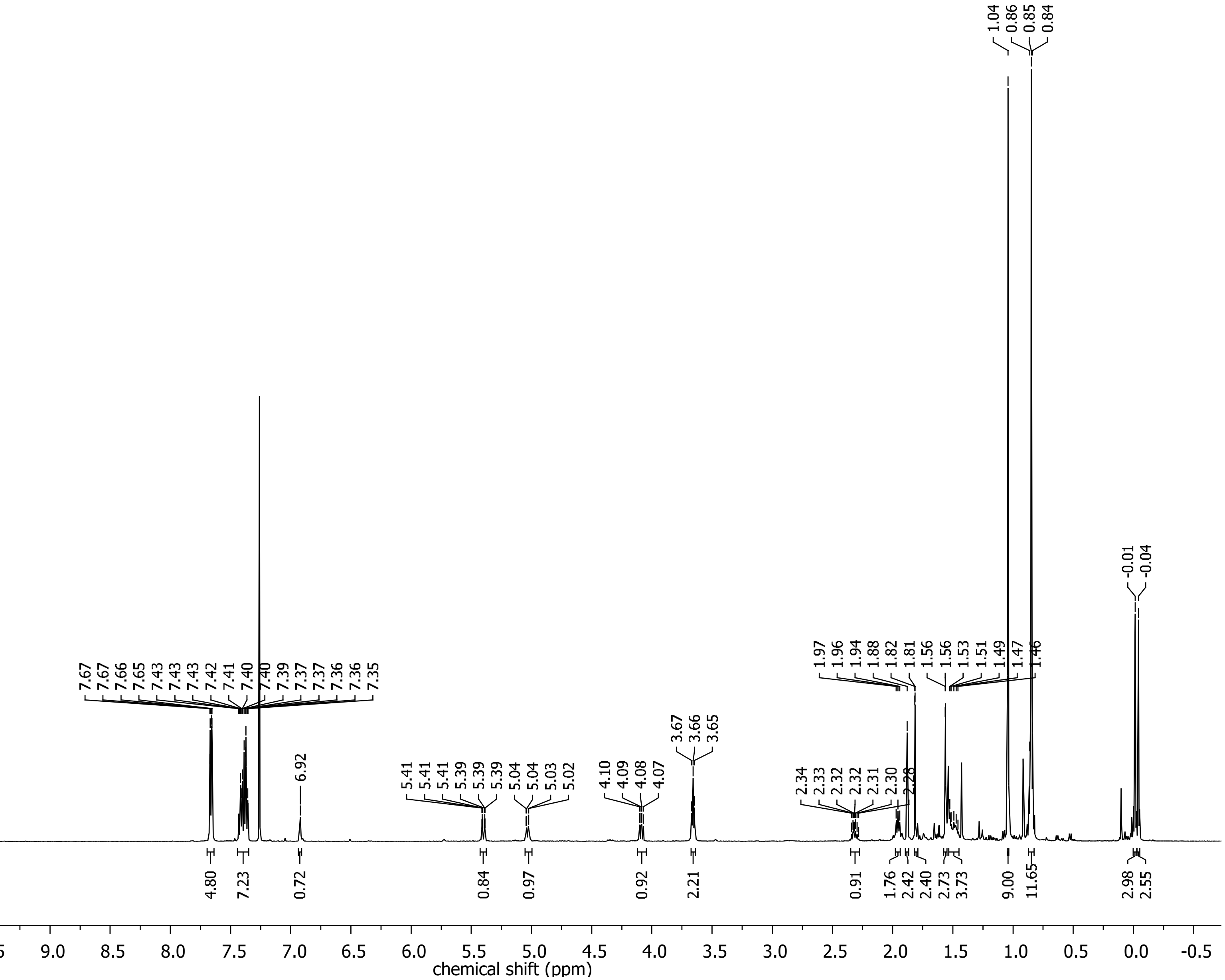

Nucleus: 13C
Frequency: 125.76 MHz
Solvent: CDCl3
Temperature: 298.0 K

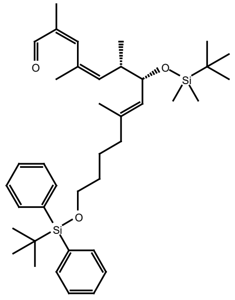

23

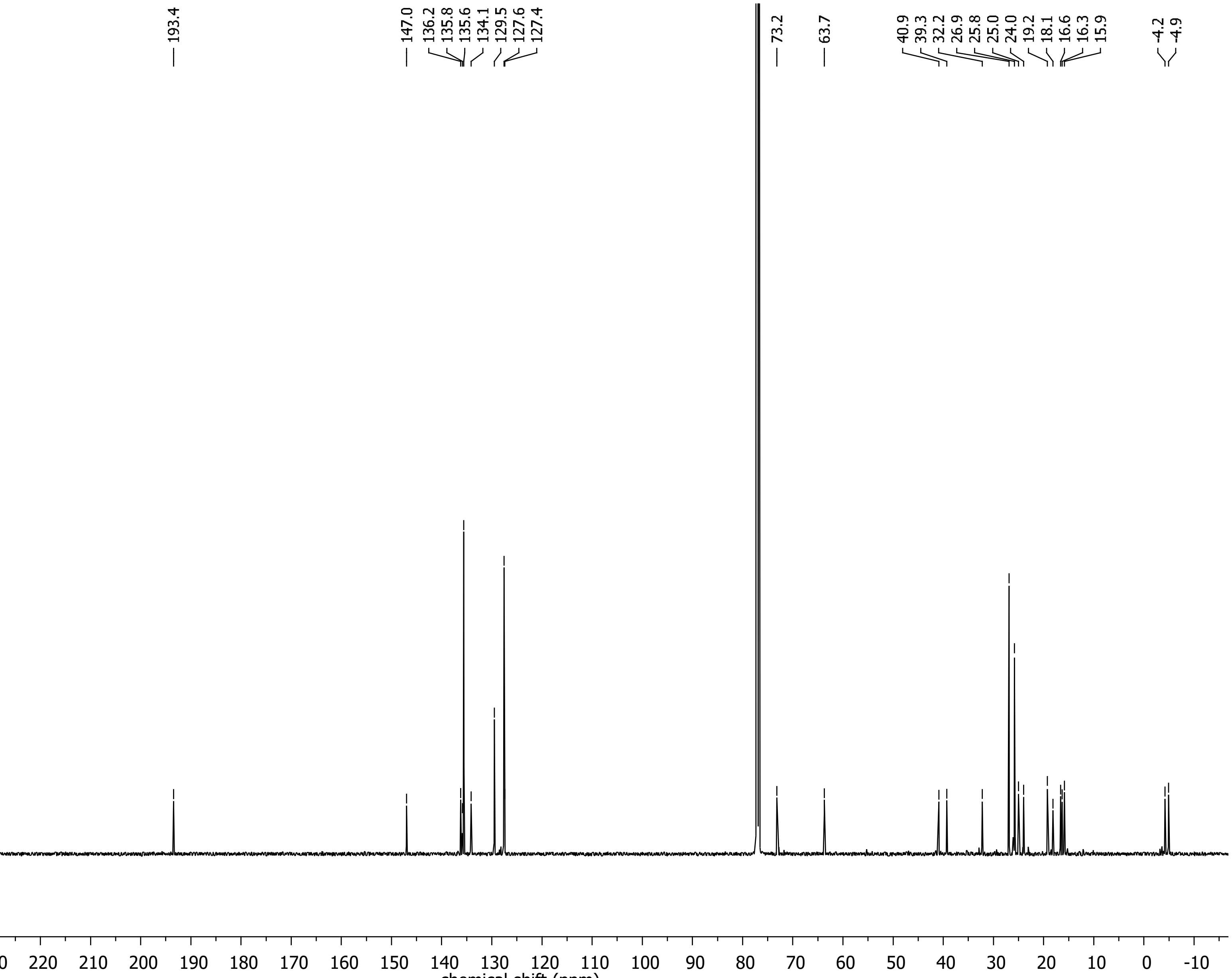

Nucleus: 1H
Frequency: 500.14 MHz
Solvent: CDCl3
Temperature: 298.0 K

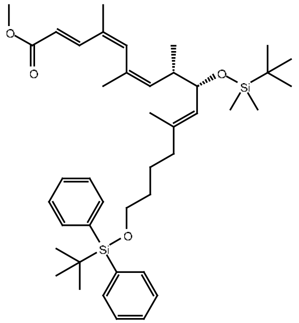

24

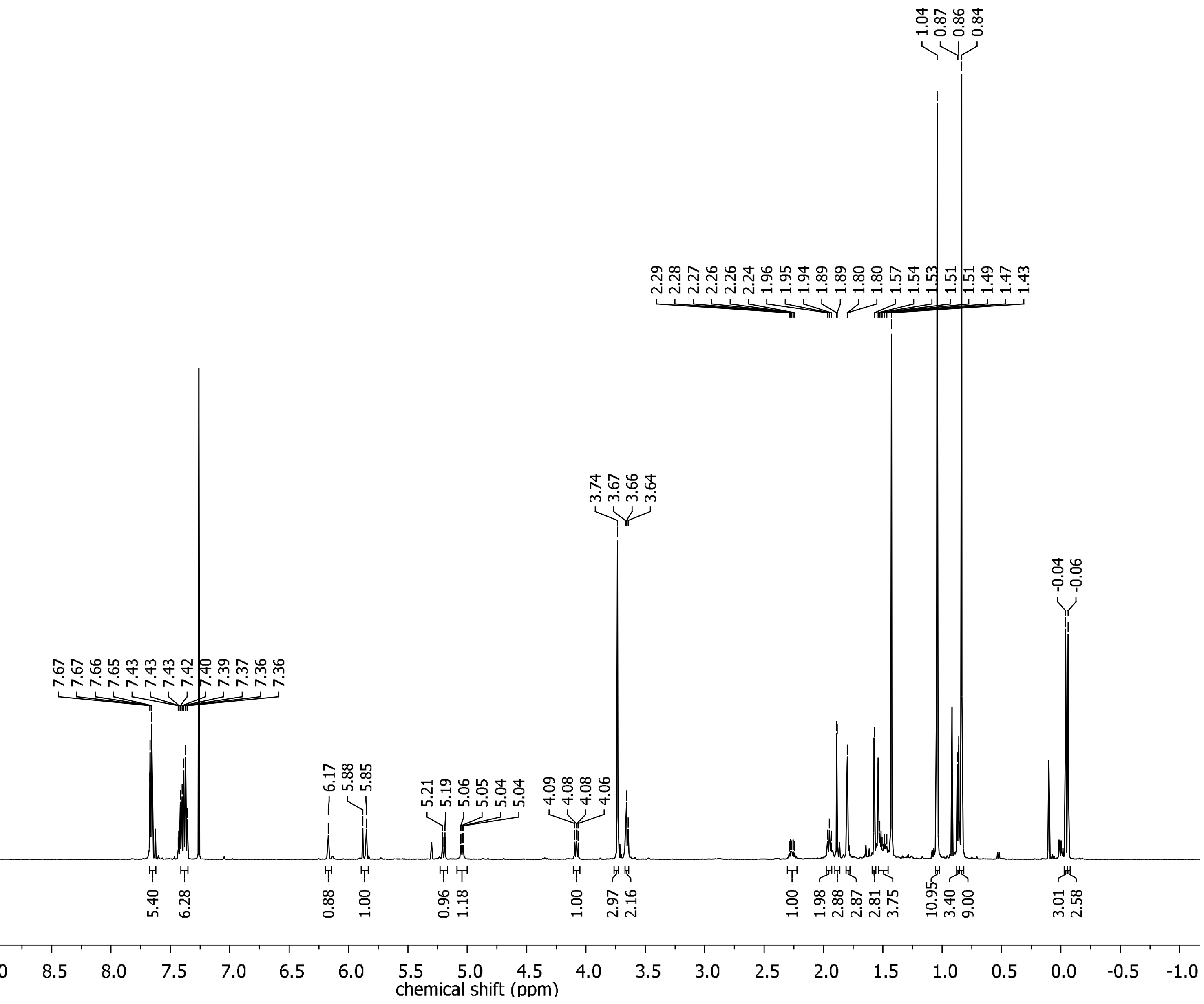

Nucleus: 13C
Frequency: 125.76 MHz
Solvent: CDCl3
Temperature: 298.0 K

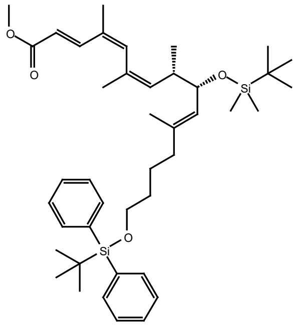

24

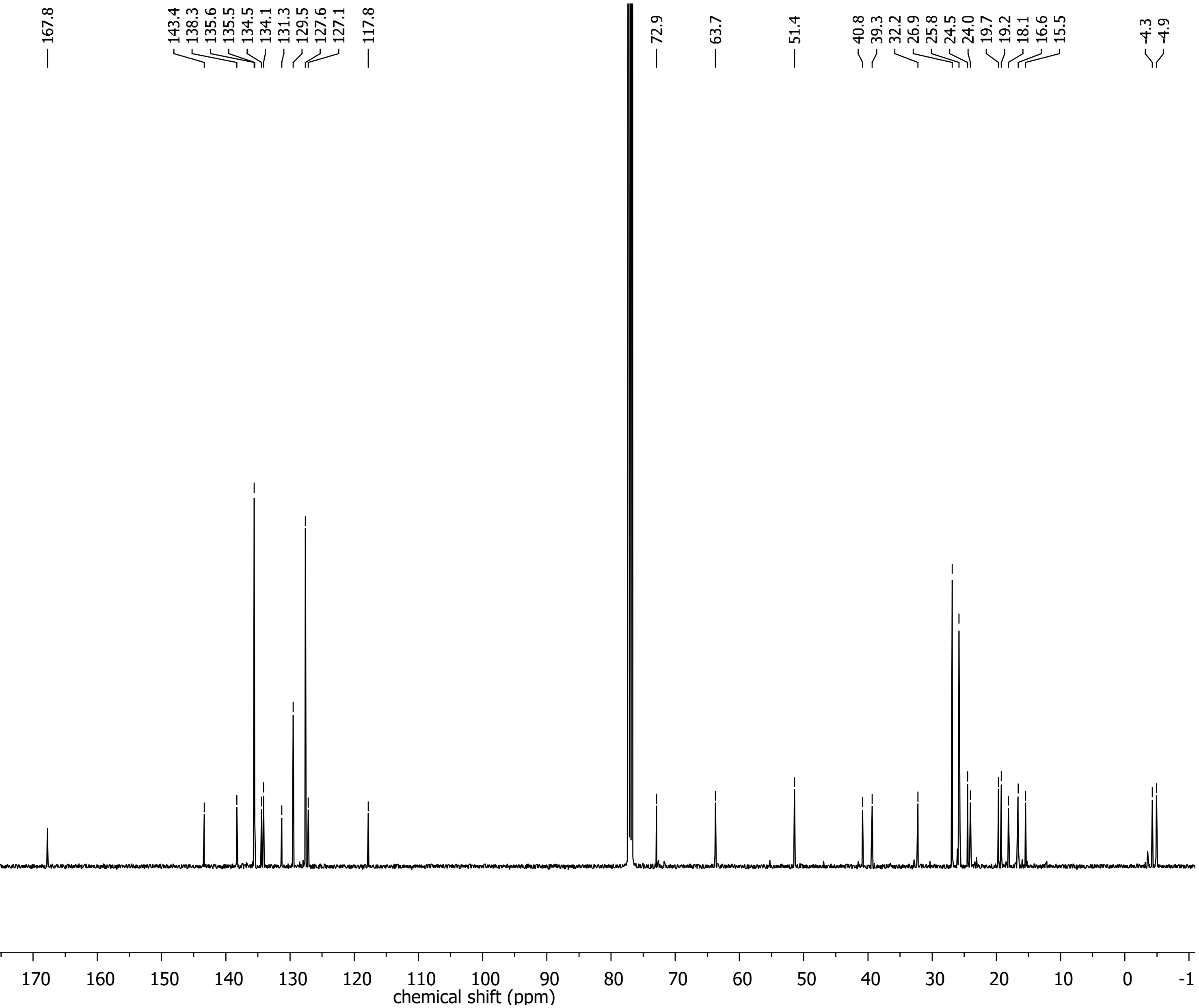

Nucleus: 1H
Frequency: 700.41 MHz
Solvent: CD2Cl2
Temperature: 298.0 K

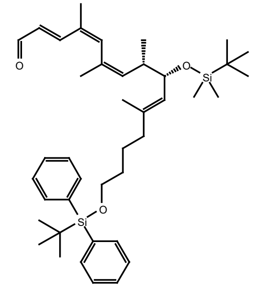

25

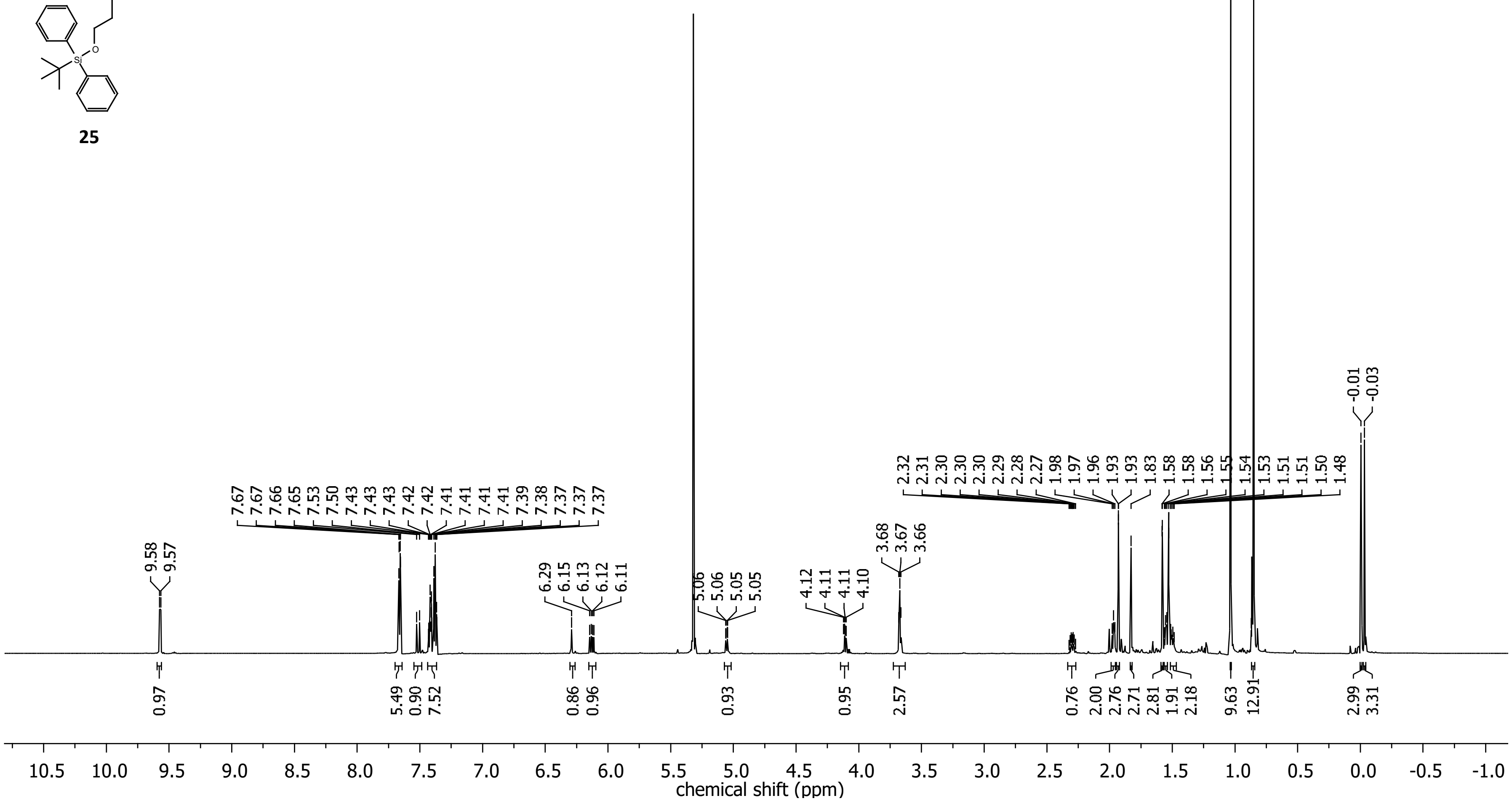

Nucleus: 13C
Frequency: 176.12 MHz
Solvent: CD2Cl2
Temperature: 298.0 K

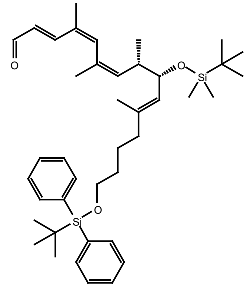

25

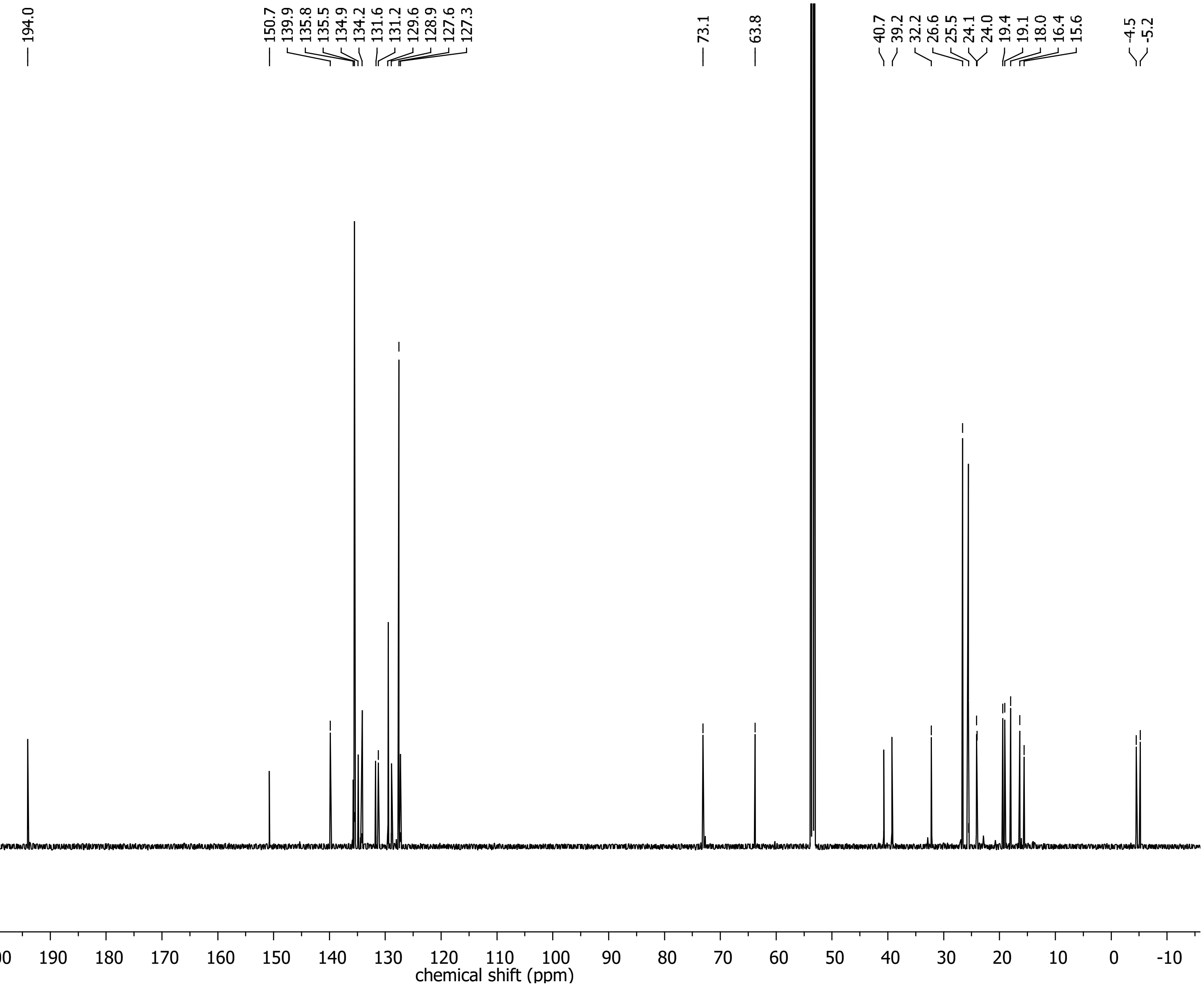

Nucleus: 1H
Frequency: 500.14 MHz
Solvent: CD2Cl2
Temperature: 298.0 K

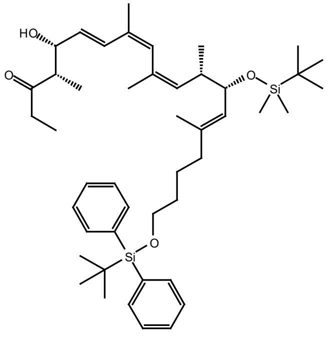

53

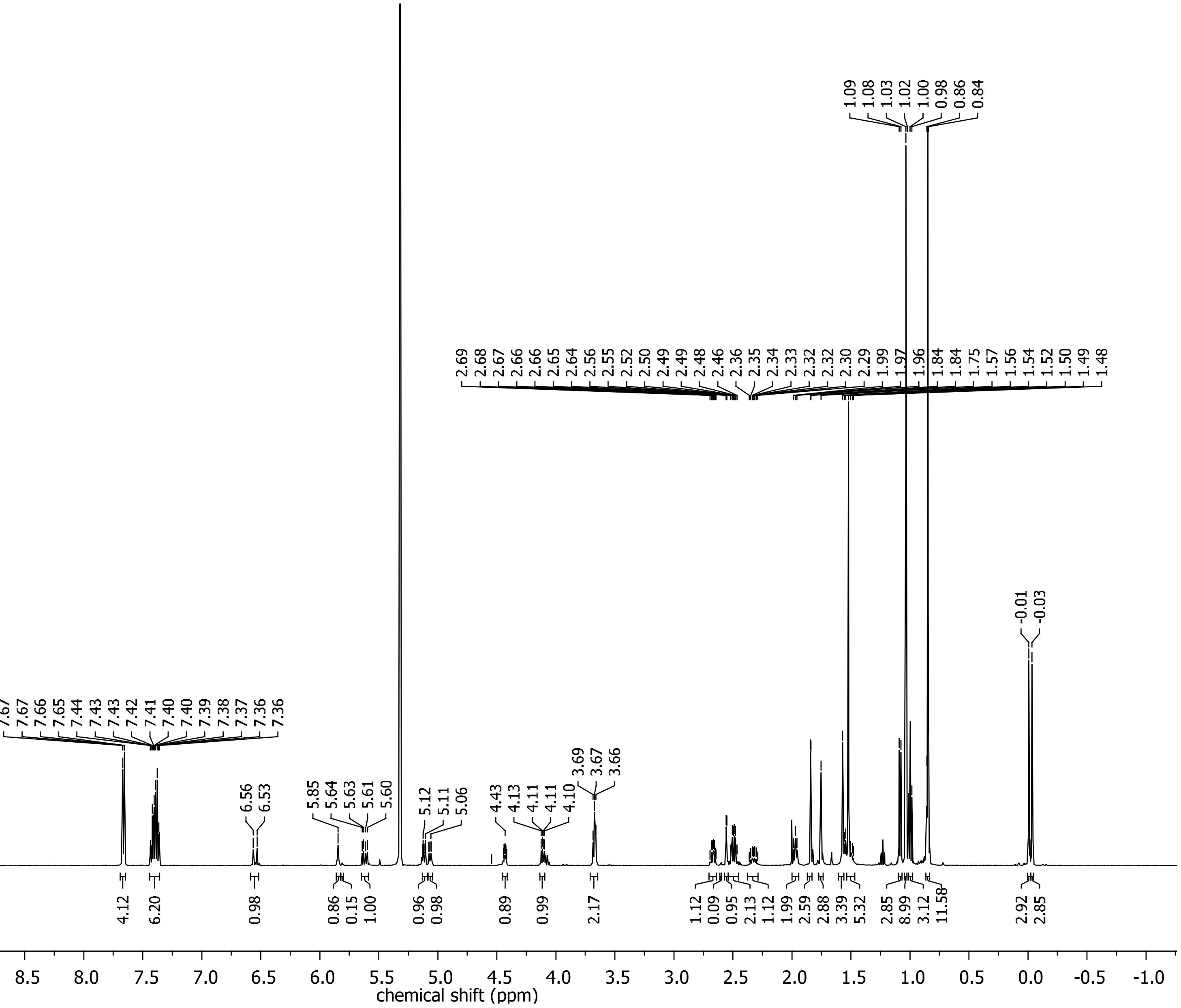

Nucleus: 13C
Frequency: 125.76 MHz
Solvent: CD2Cl2
Temperature: 298.0 K

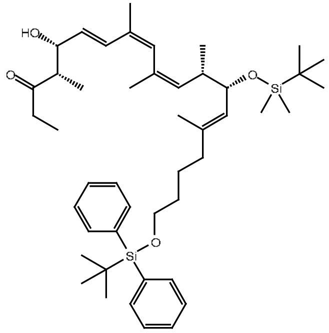

53

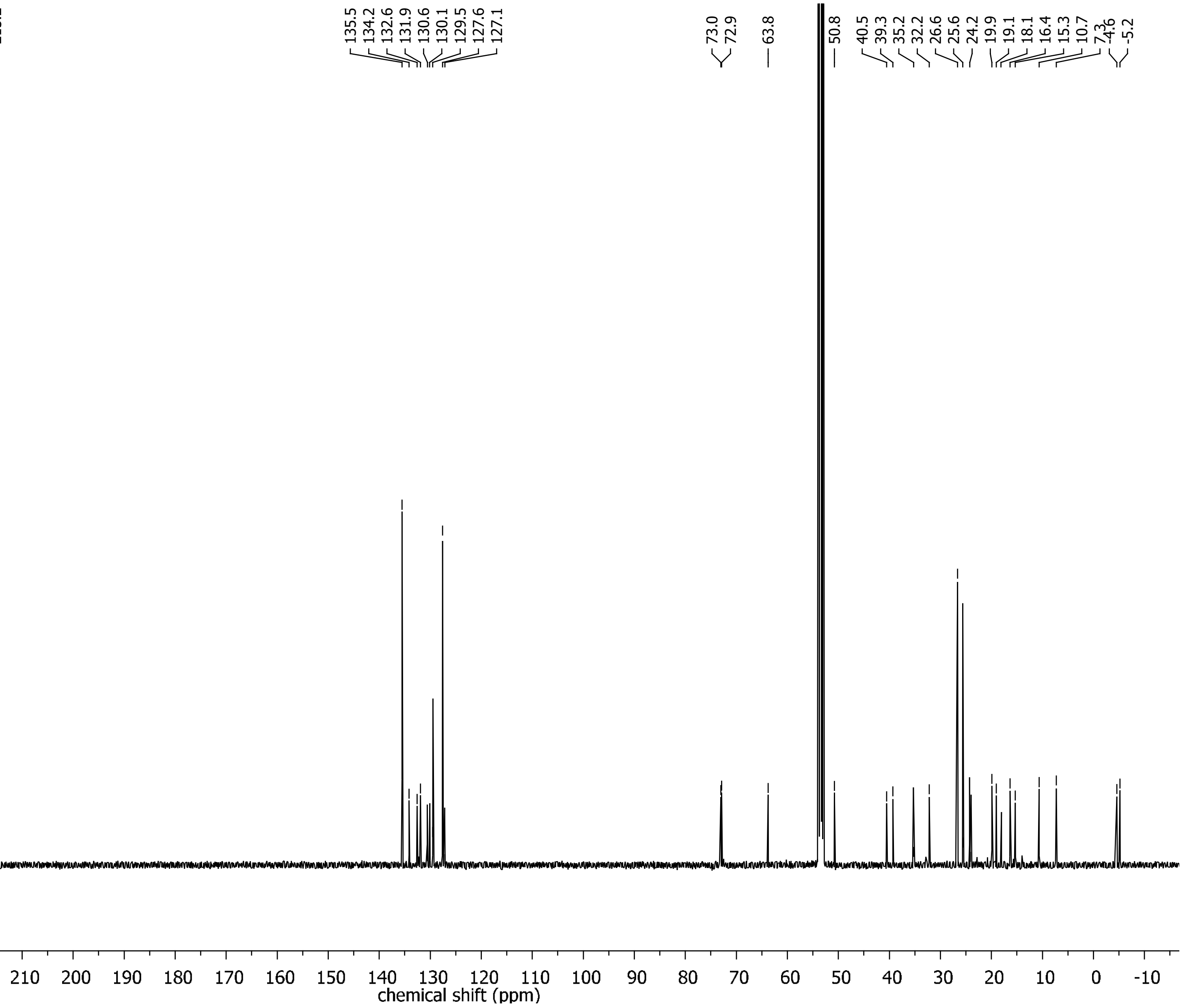

Nucleus: 1H
Frequency: 700.41 MHz
Solvent: CD2Cl2
Temperature: 298.0 K

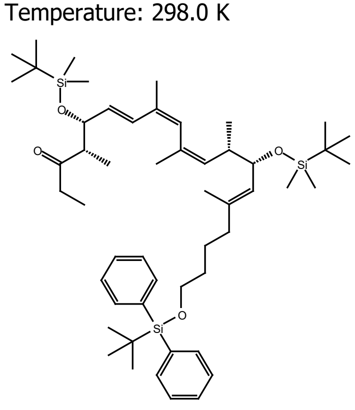

27

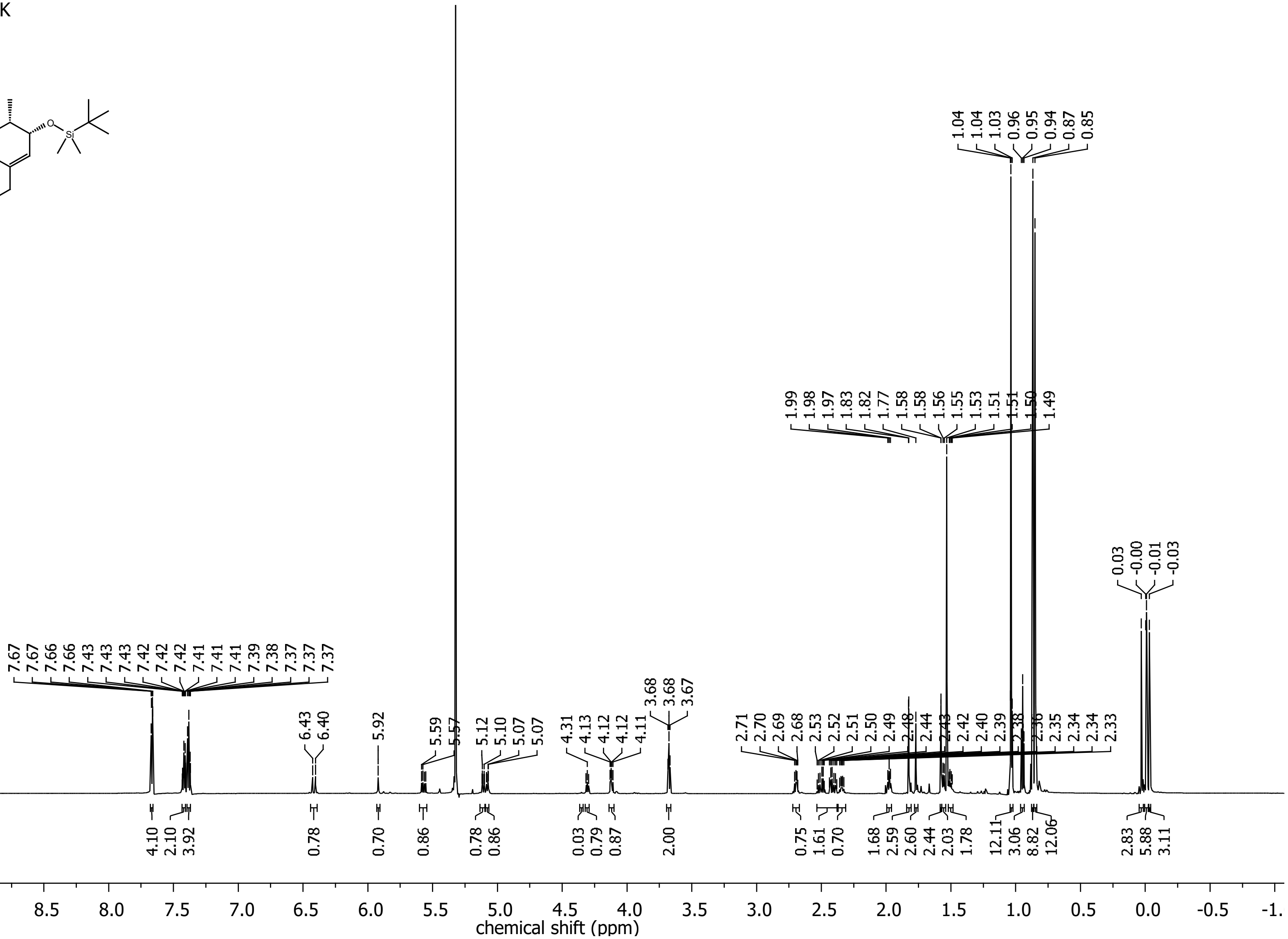

Nucleus: 13C
Frequency: 176.12 MHz
Solvent: CD2Cl2
Temperature: 298.0 K

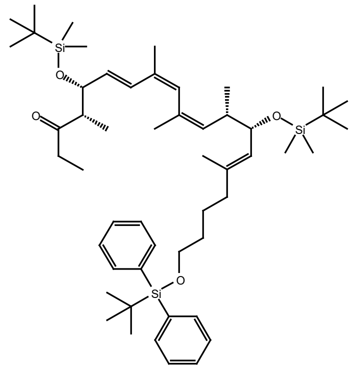

27

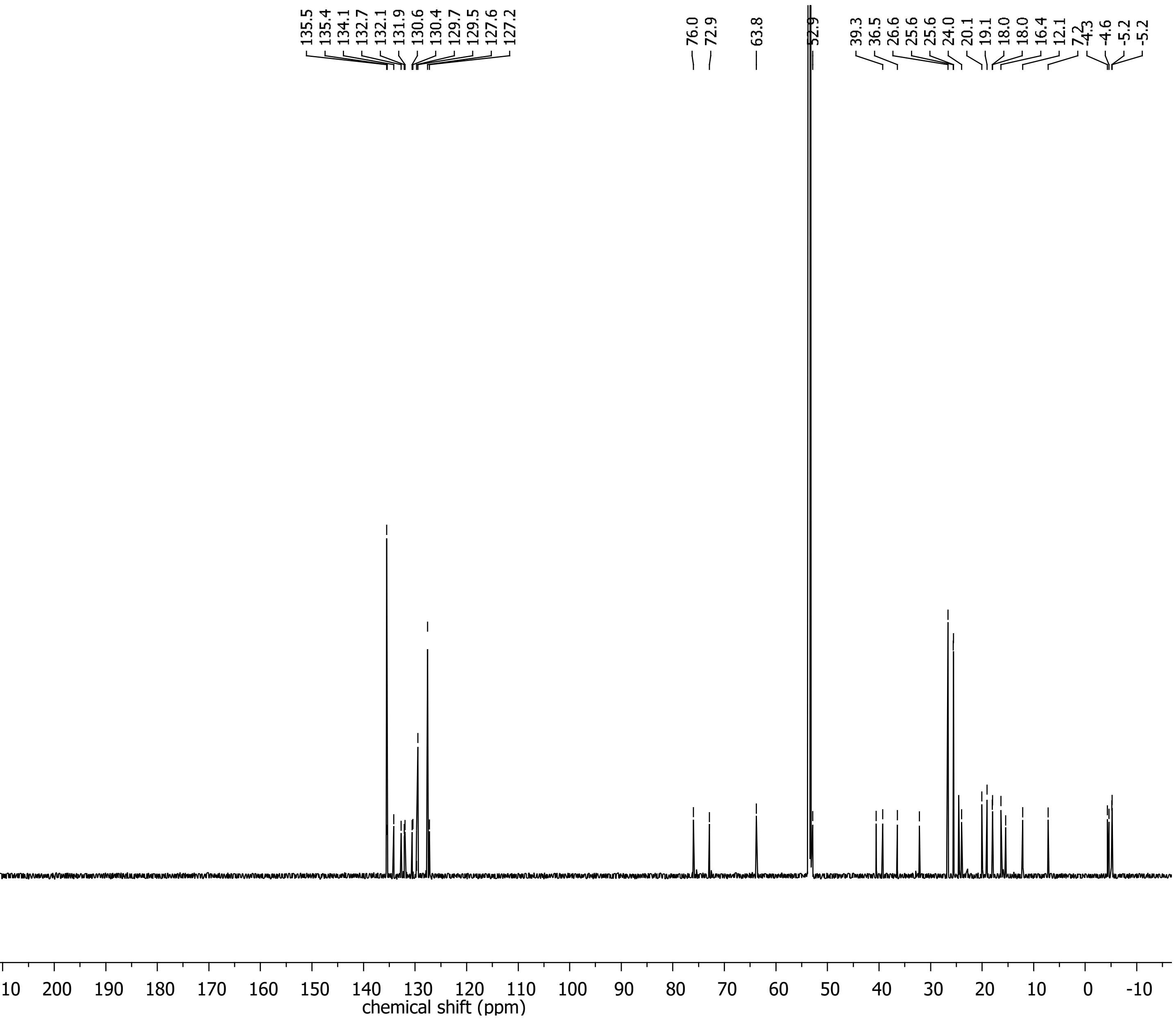

Nucleus: 1H
Frequency: 500.14 MHz
Solvent: CD2Cl2
Temperature: 298.0 K

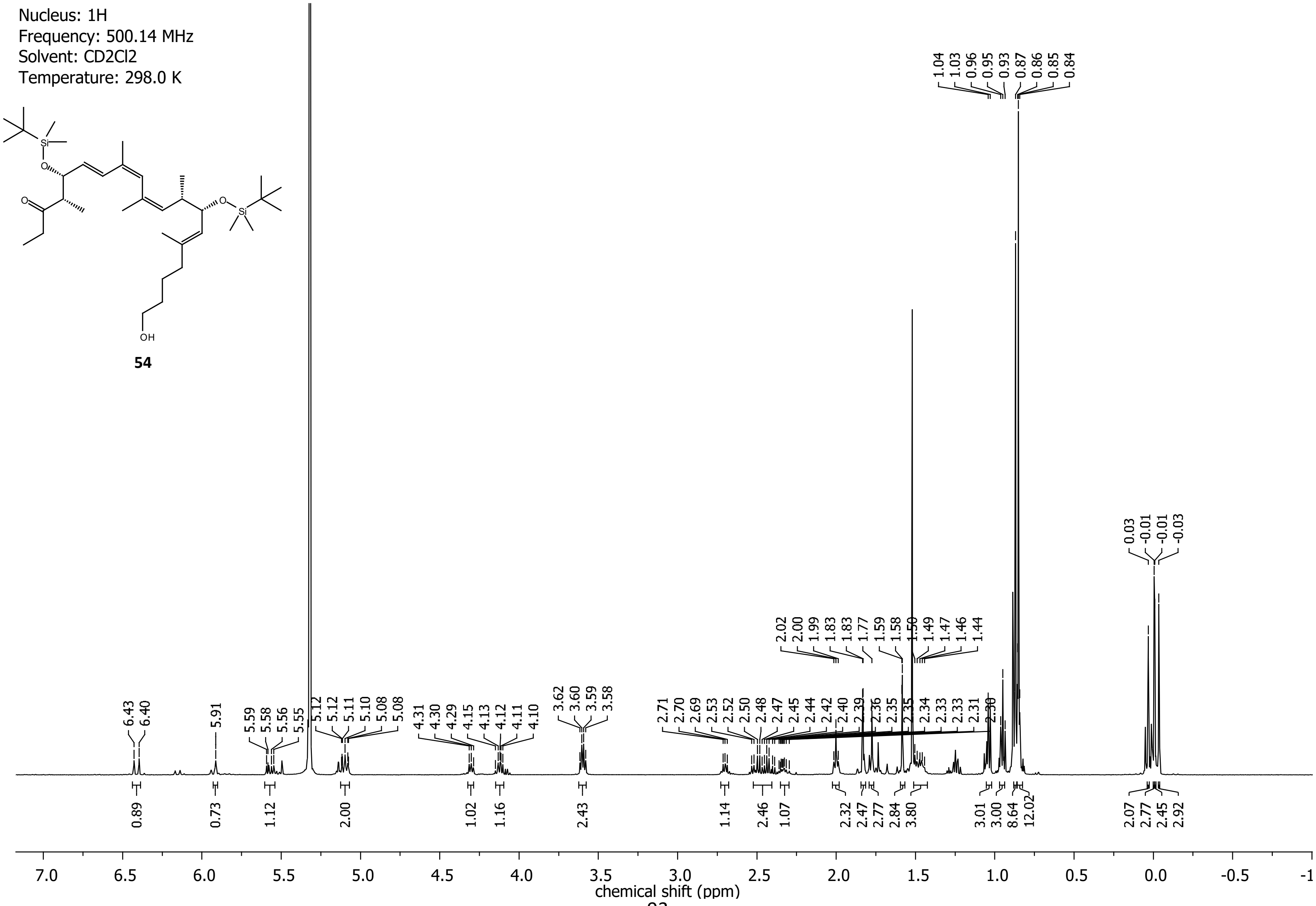

54

Nucleus: 13C
Frequency: 125.76 MHz
Solvent: CD2Cl2
Temperature: 298.0 K

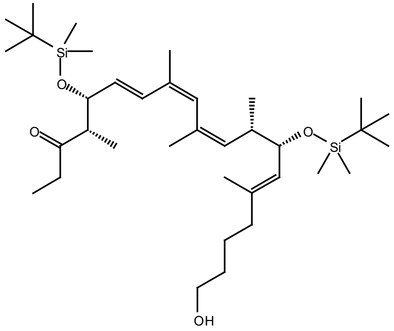

54

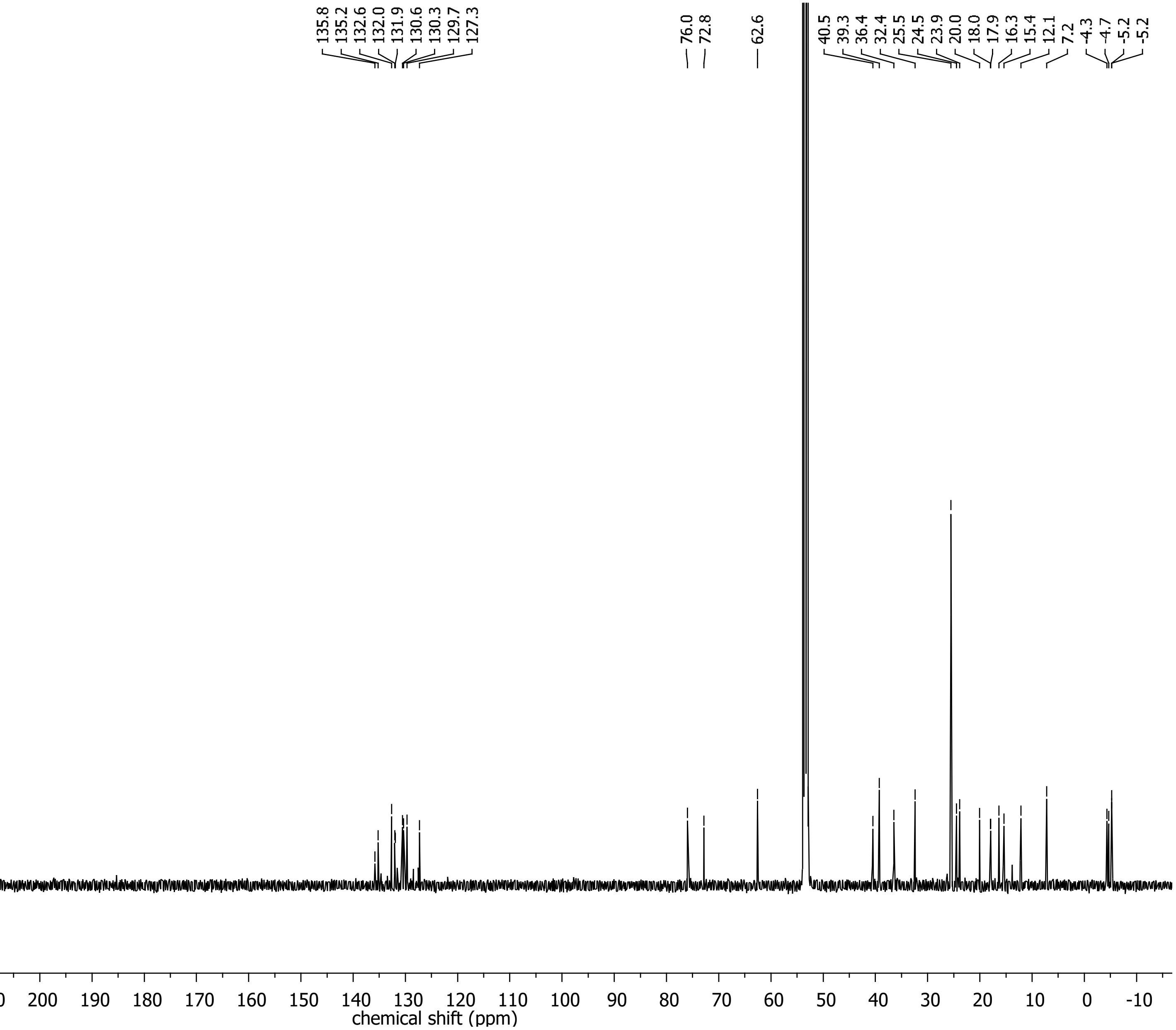

Nucleus: 1H
Frequency: 700.41 MHz
Solvent: CD2Cl2
Temperature: 298.0 K

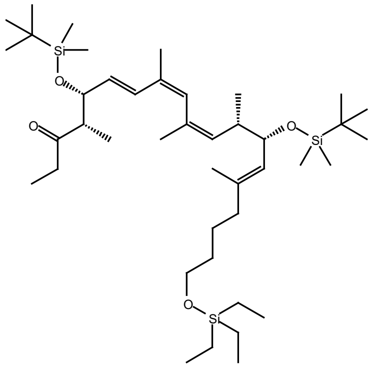

28

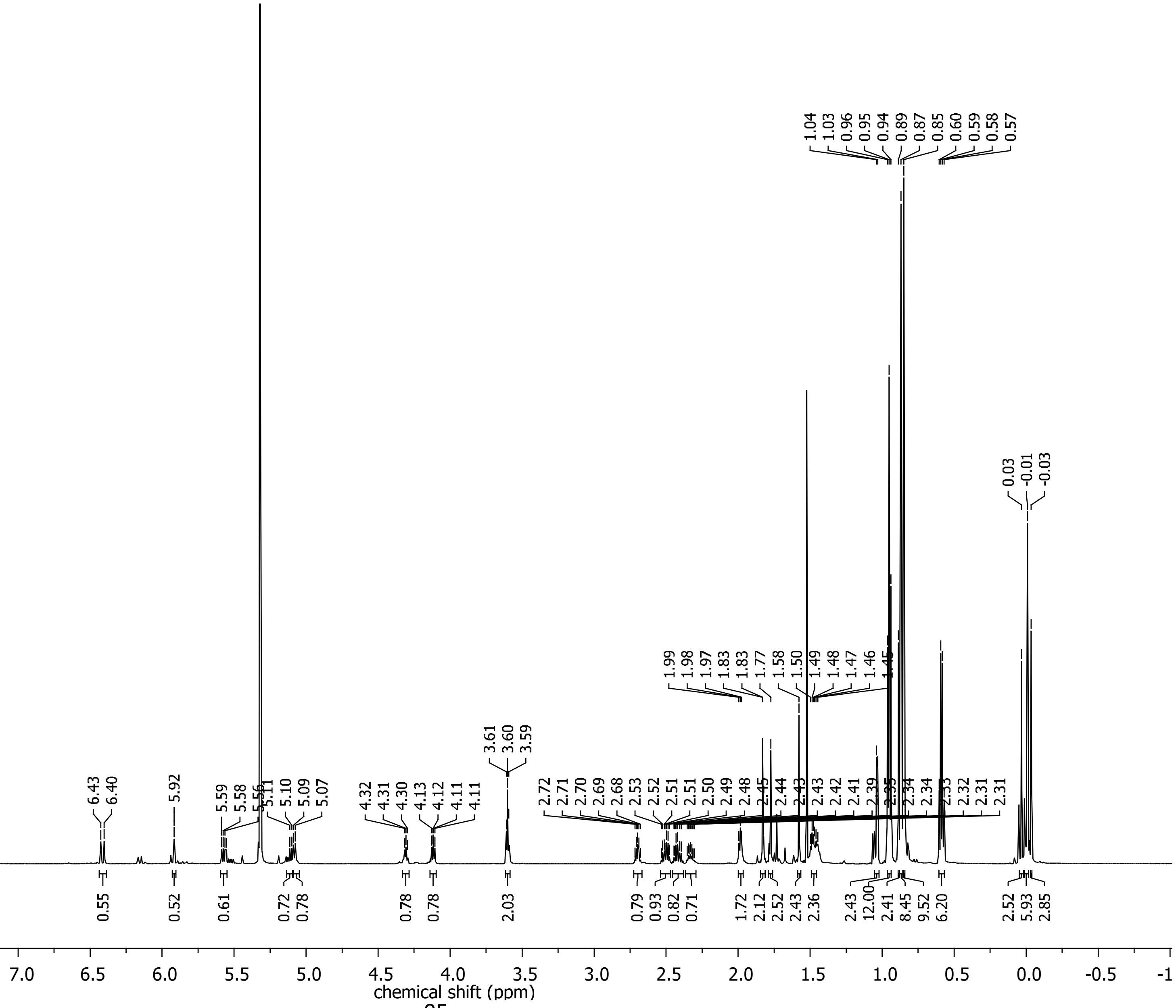

Nucleus: 13C
Frequency: 176.12 MHz
Solvent: CD2Cl2
Temperature: 298.0 K

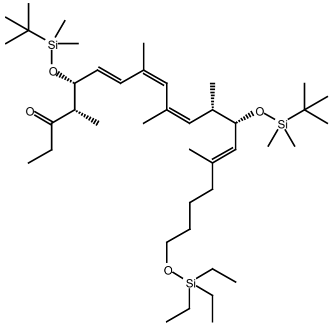

28

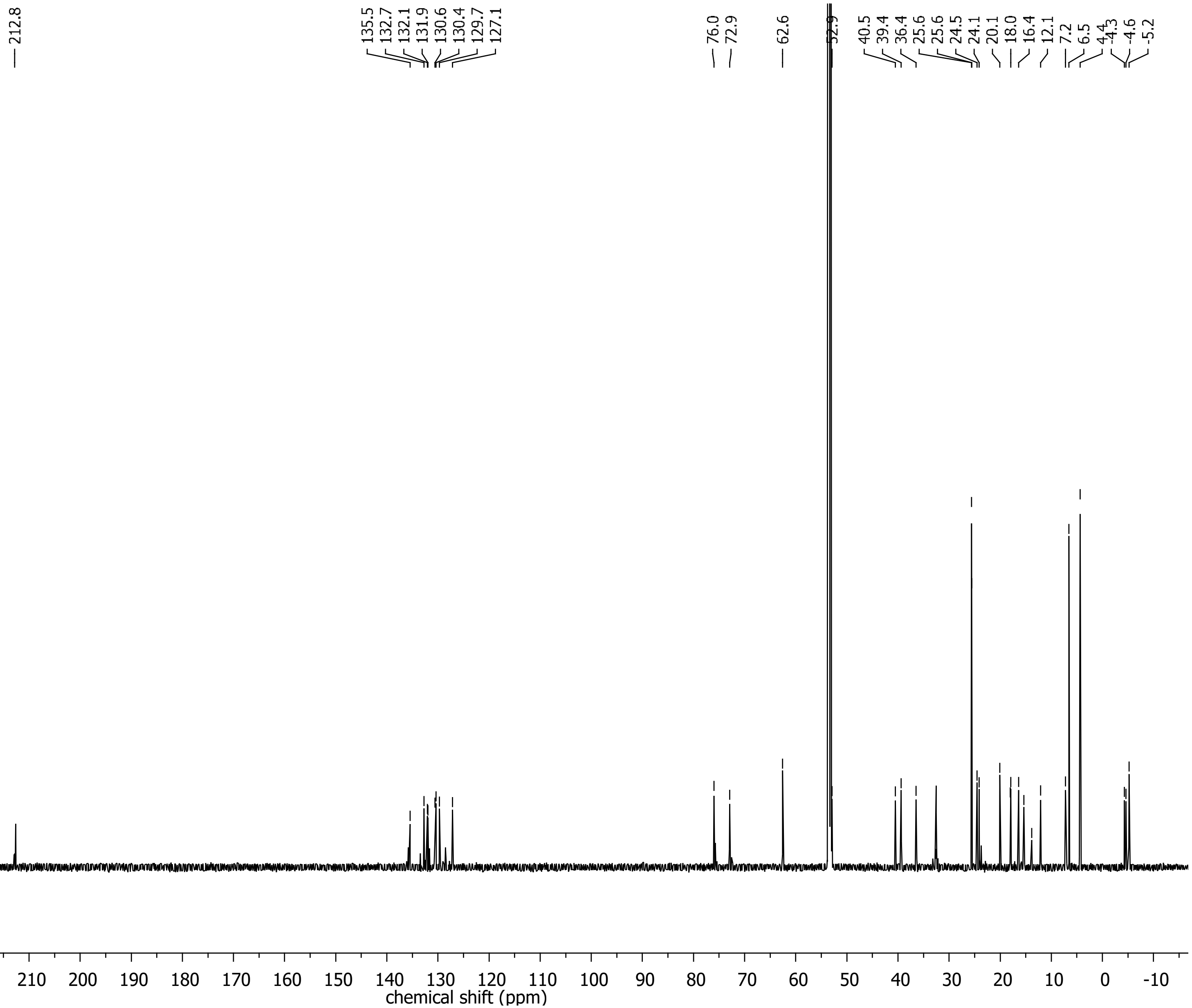

Nucleus: 1H
Frequency: 300.13 MHz
Solvent: CDCl3
Temperature: 298.0 K

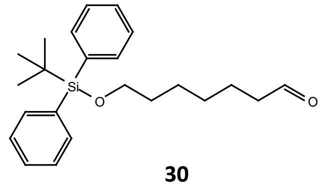

30

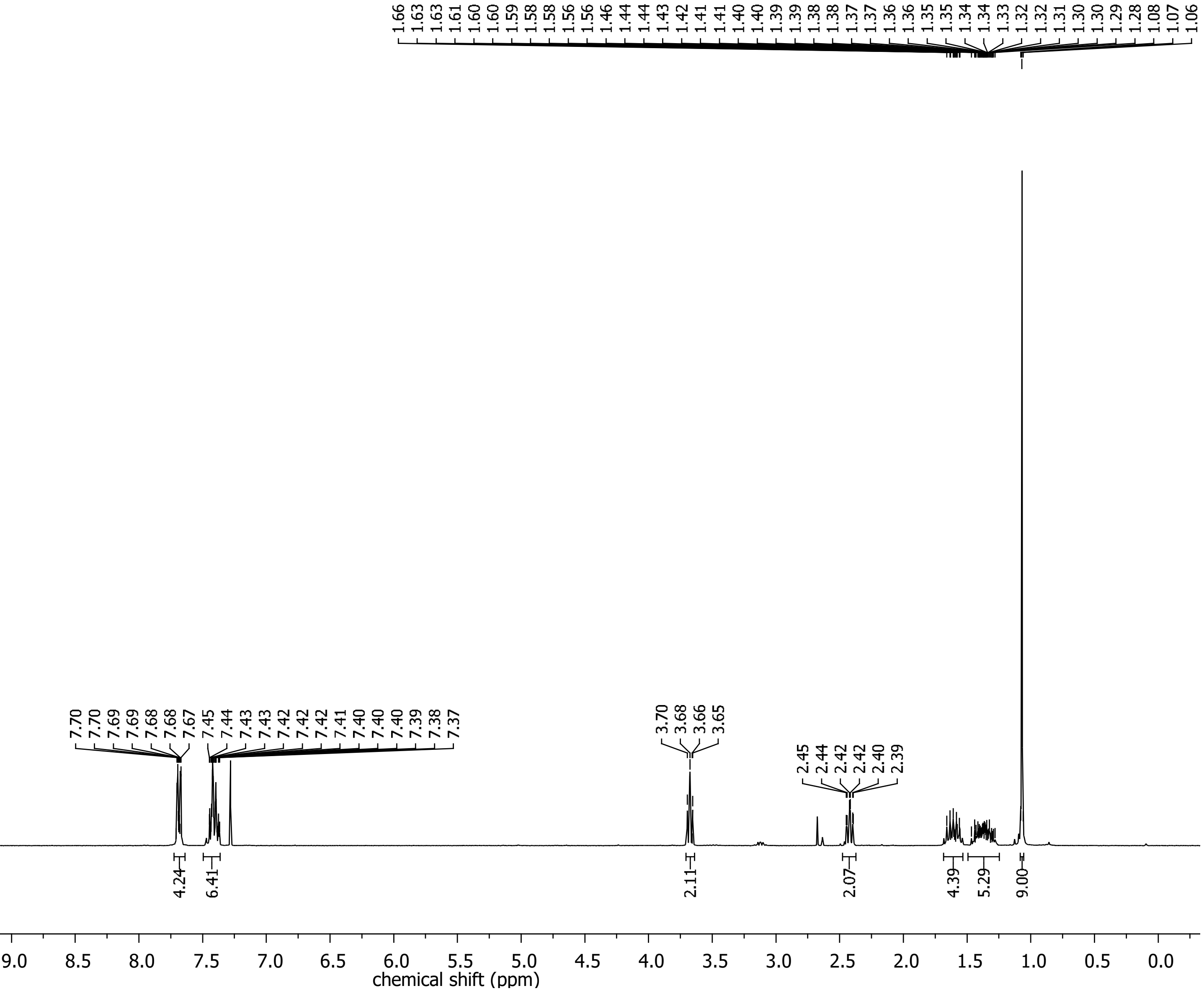

Nucleus: 13C
Frequency: 125.74 MHz
Solvent: CDCl3
Temperature: 298.0 K

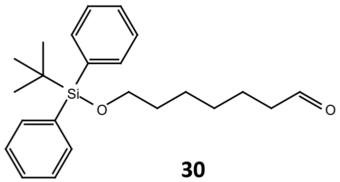

30

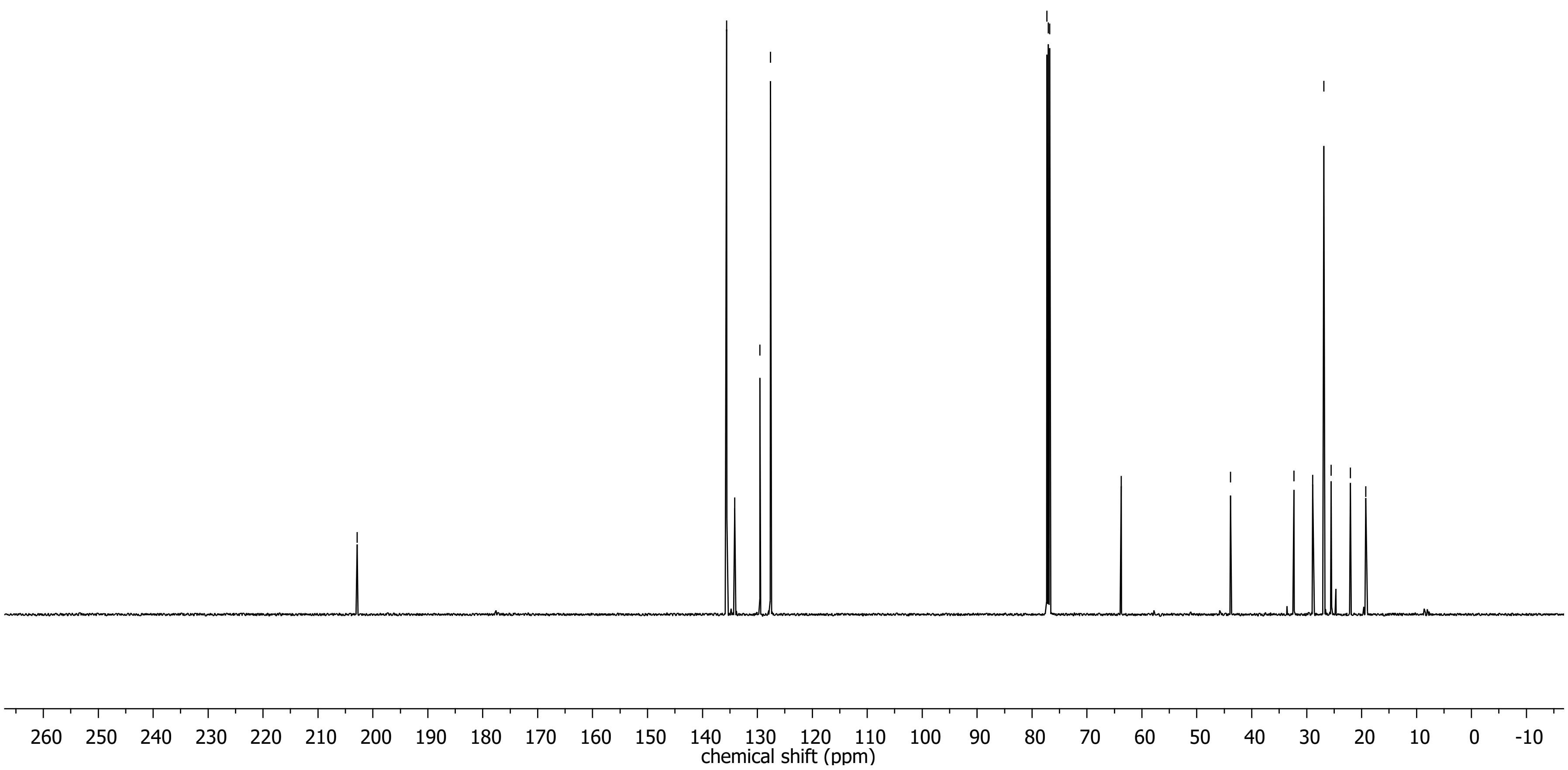

Nucleus: 1H
Frequency: 700.41 MHz
Solvent: CDCl3
Temperature: 298.0 K

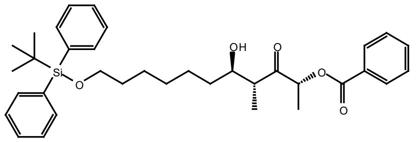

31

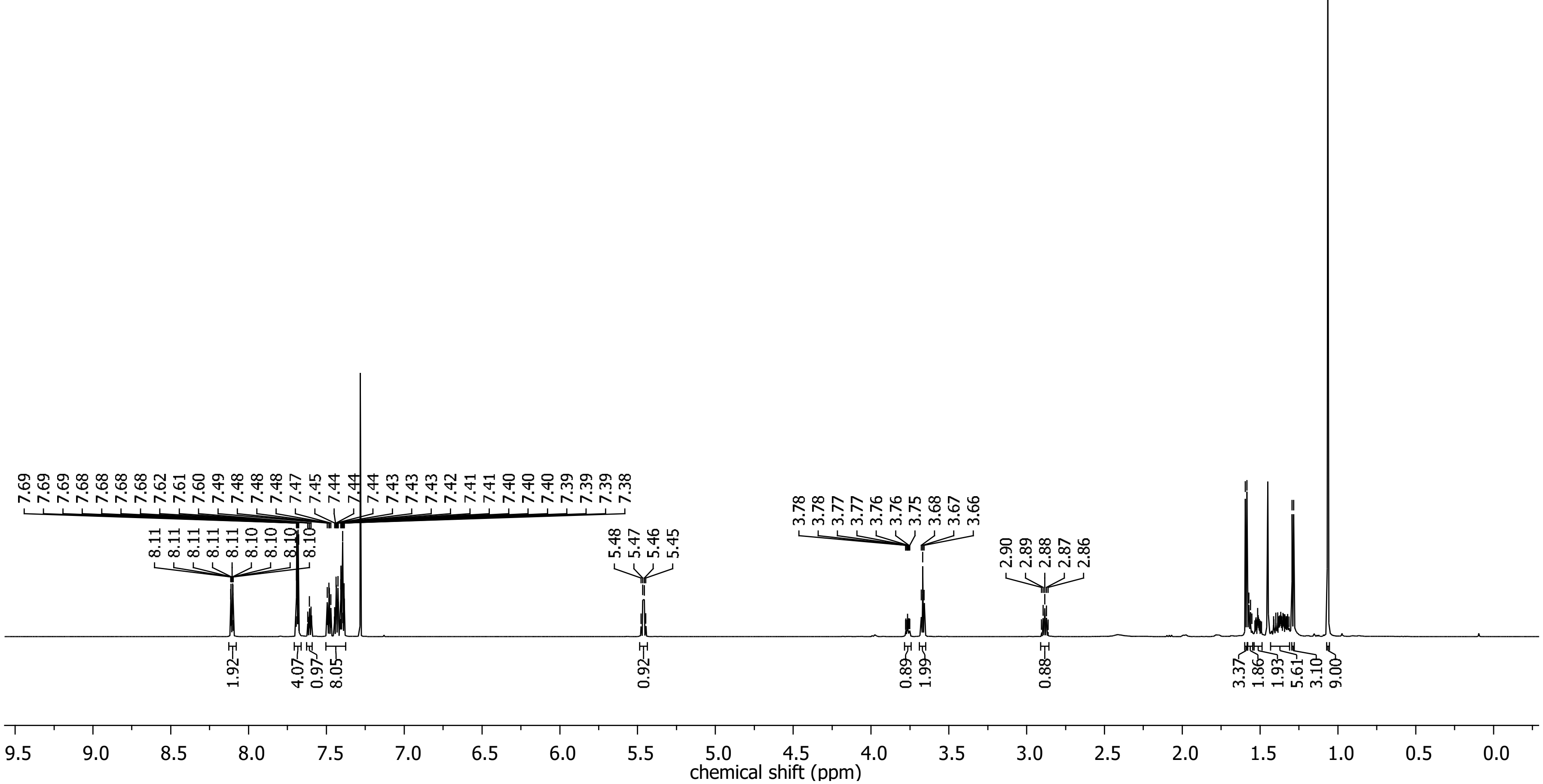

Nucleus: 13C
Frequency: 176.12 MHz
Solvent: CDCl3
Temperature: 298.0 K

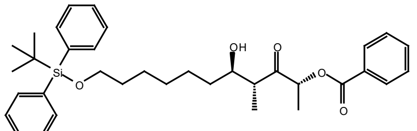

31

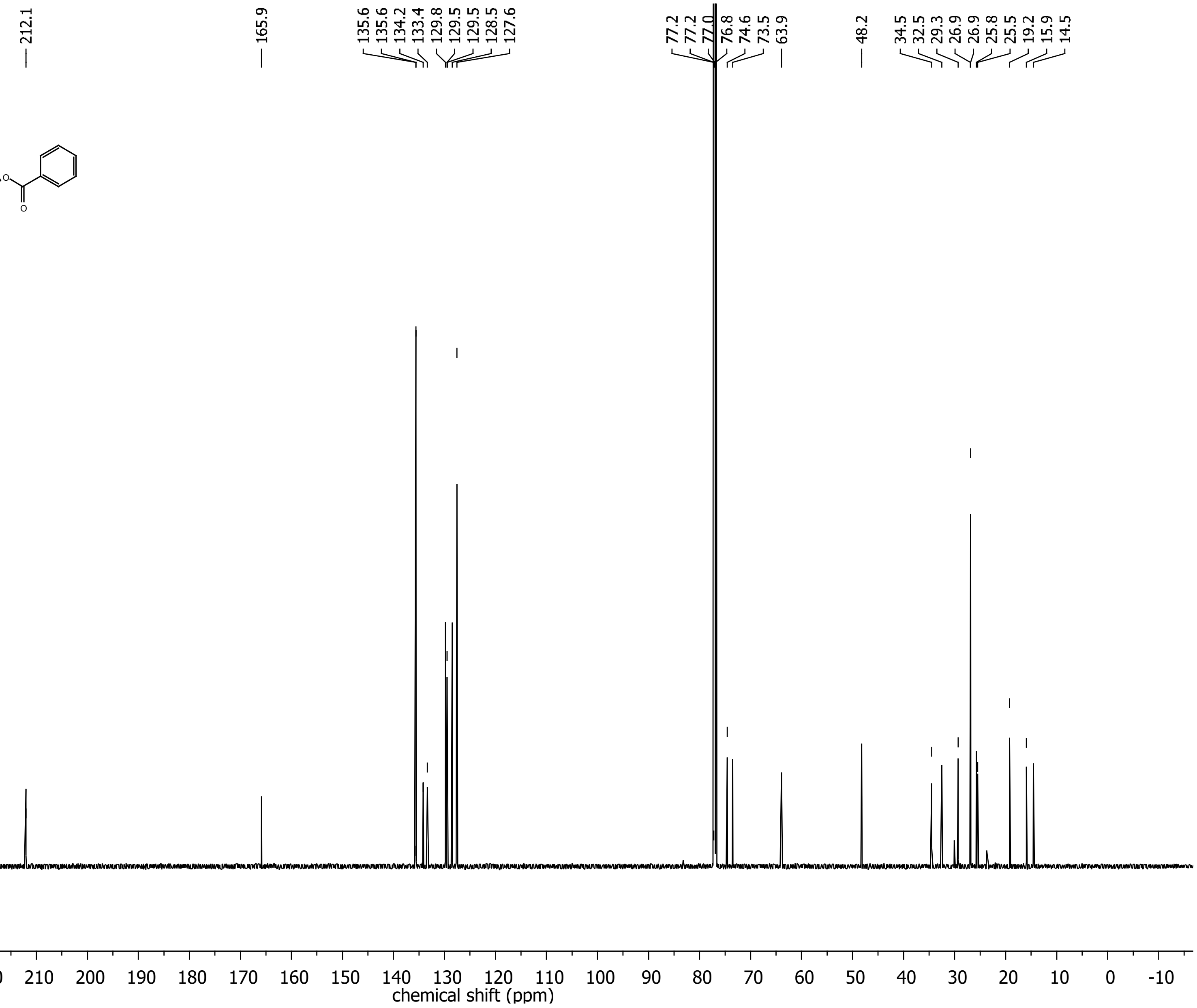

Nucleus: 1H
Frequency: 500.07 MHz
Solvent: CDCl3
Temperature: 298.0 K

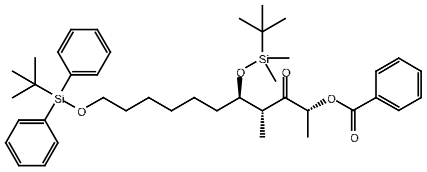

55

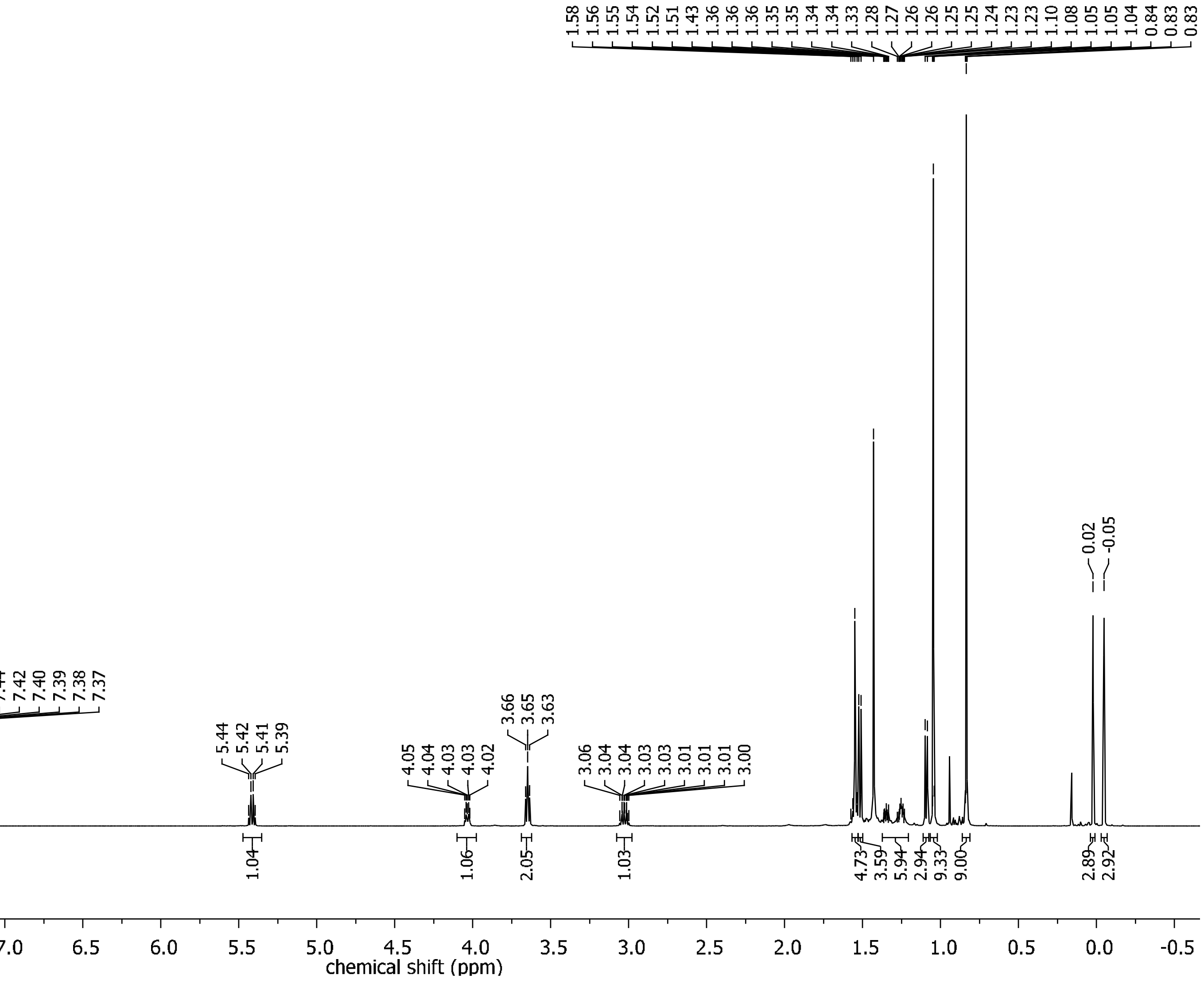

Nucleus: 13C
Frequency: 125.74 MHz
Solvent: CDCl3
Temperature: 298.0 K

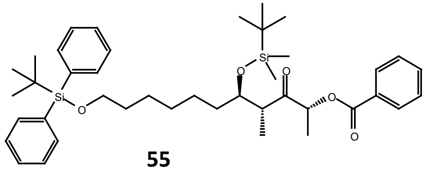

55

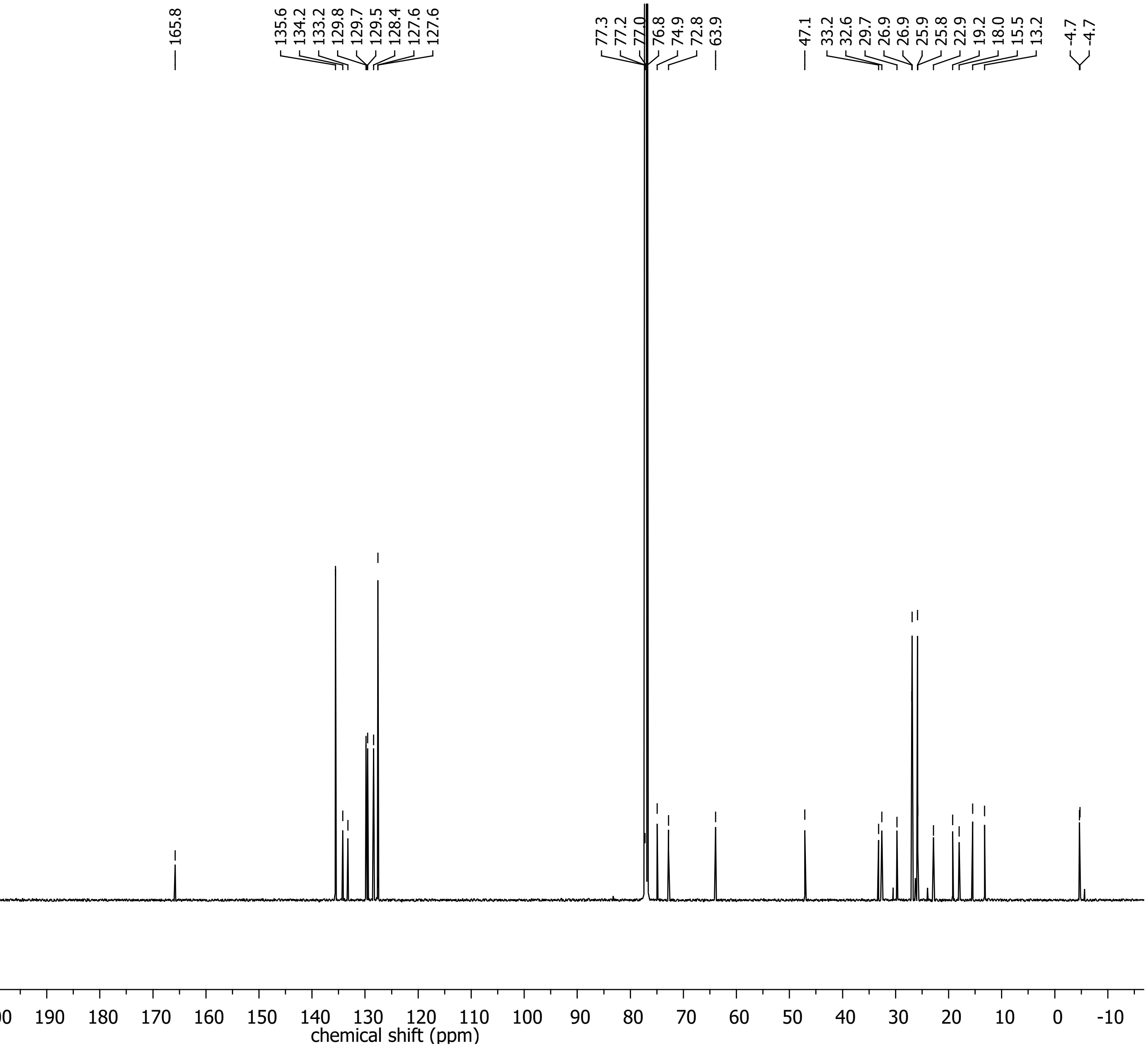

Nucleus: 1H
Frequency: 499.13 MHz
Solvent: CDCl3
Temperature: 298.0 K

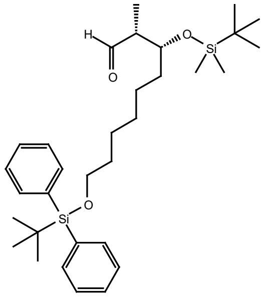

32

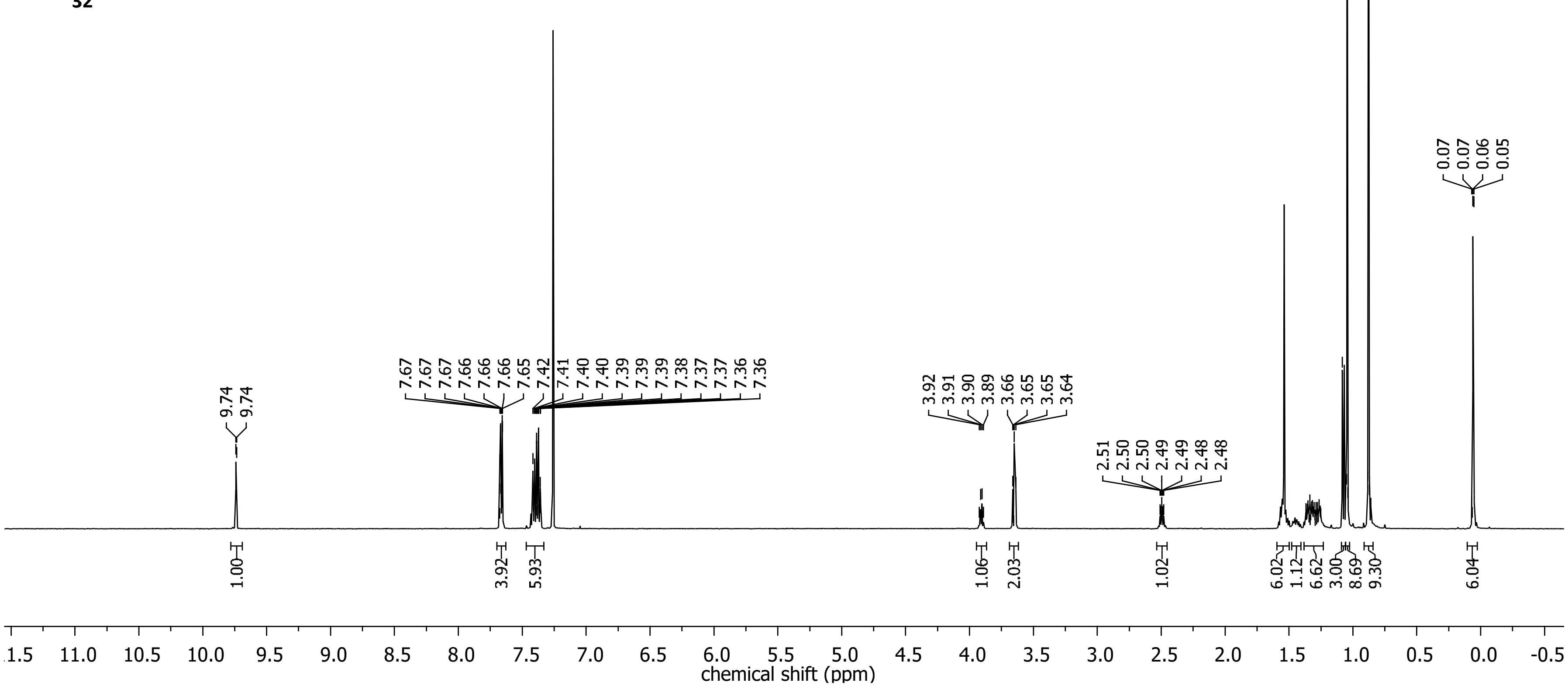

Nucleus: 13C
Frequency: 125.51 MHz
Solvent: CDCl3
Temperature: 298.0 K

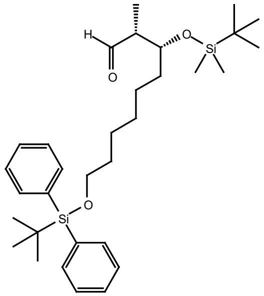

32

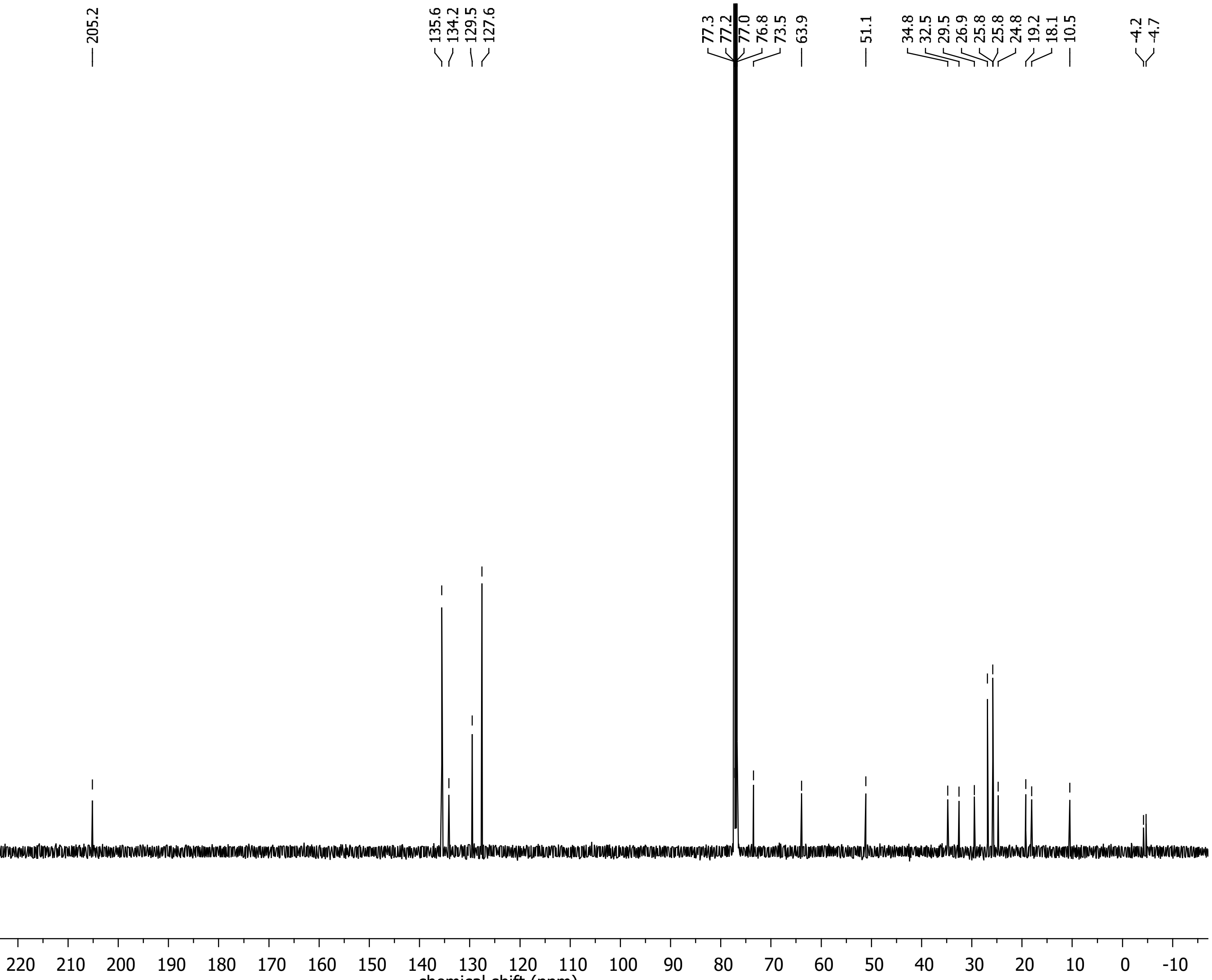

Nucleus: 1H
Frequency: 700.41 MHz
Solvent: CDCl3
Temperature: 298.0 K

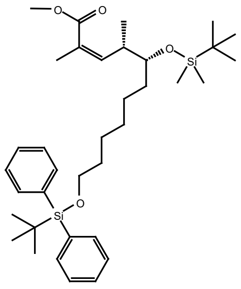

33

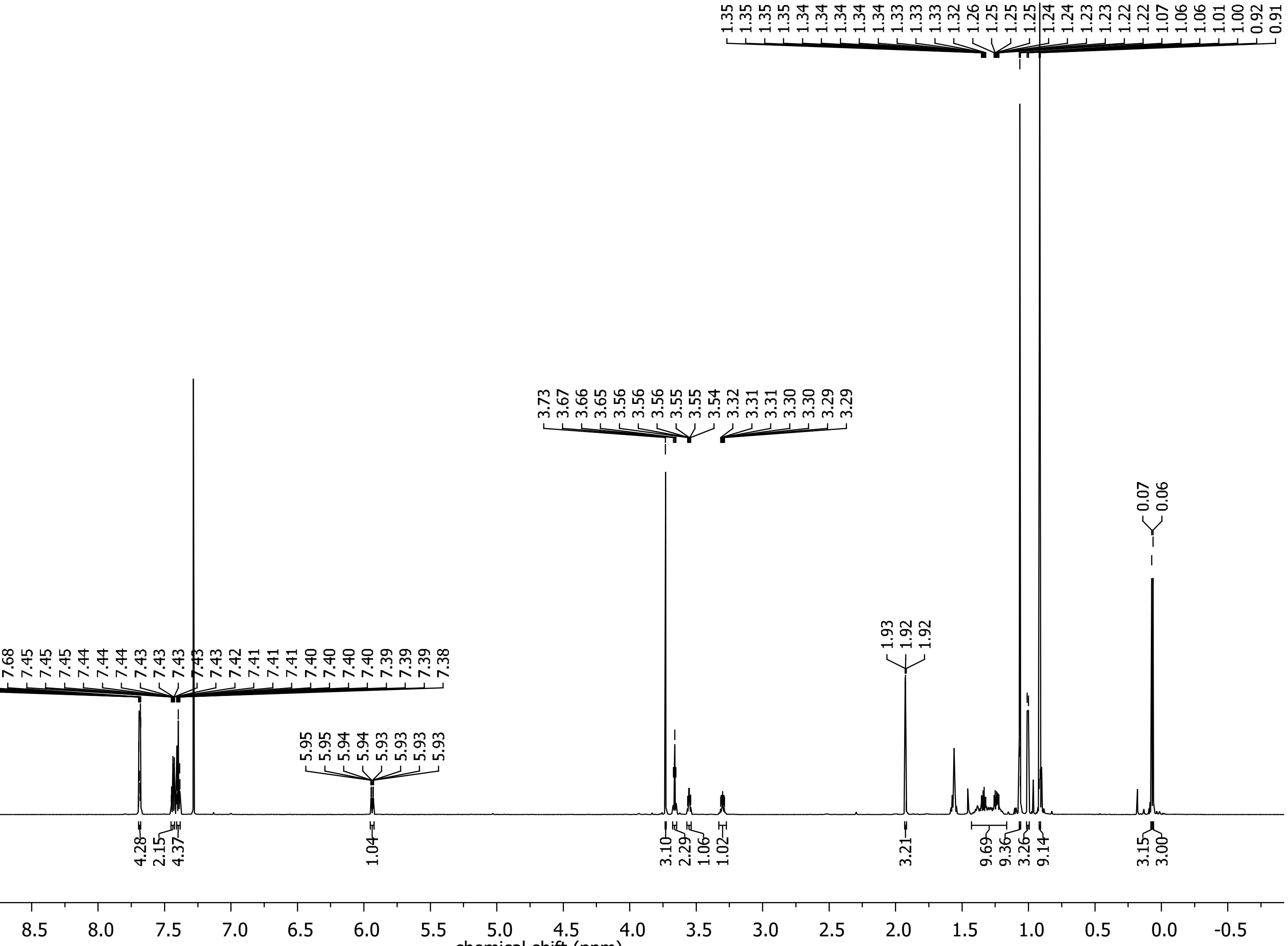

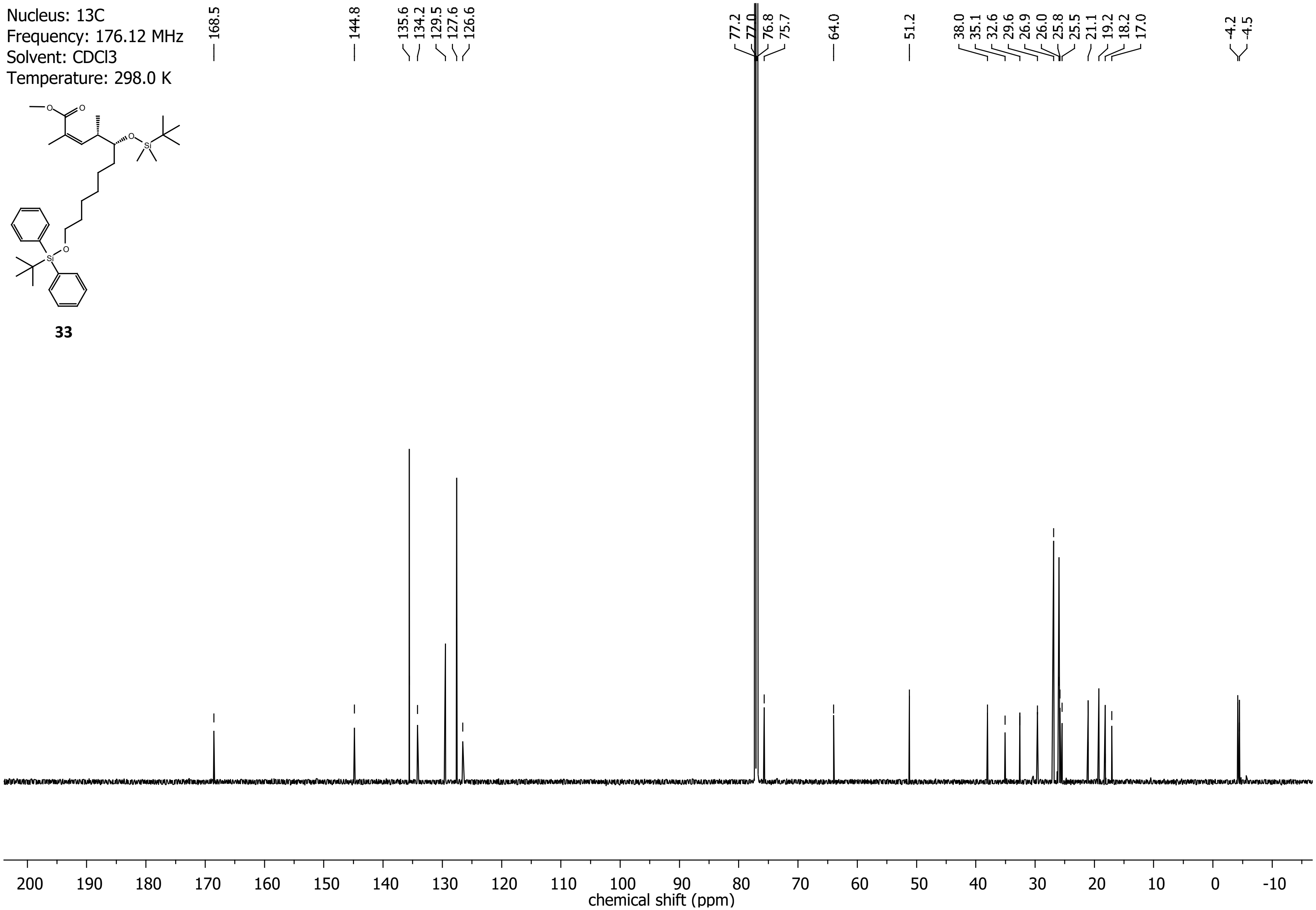
Nucleus: 13C
Frequency: 176.12 MHz
Solvent: CDCl3
Temperature: 298.0 K
168.5
144.8
135.6
134.2
129.5
127.6
126.6
77.2
77.0
76.8
75.7
64.0
51.2
38.0
35.1
32.6
29.6
26.9
26.0
25.8
25.5
21.1
19.2
18.2
17.0
-4.2
-4.5
200 190 180 170 160 150 140 130 120 110 100 90 80 70 60 50 40 30 20 10 0 -10
chemical shift (ppm)
O
O
O
Si
O
Si

33

Nucleus: 1H
Frequency: 700.41 MHz
Solvent: CDCl3
Temperature: 298.0 K

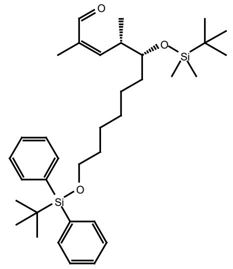

34

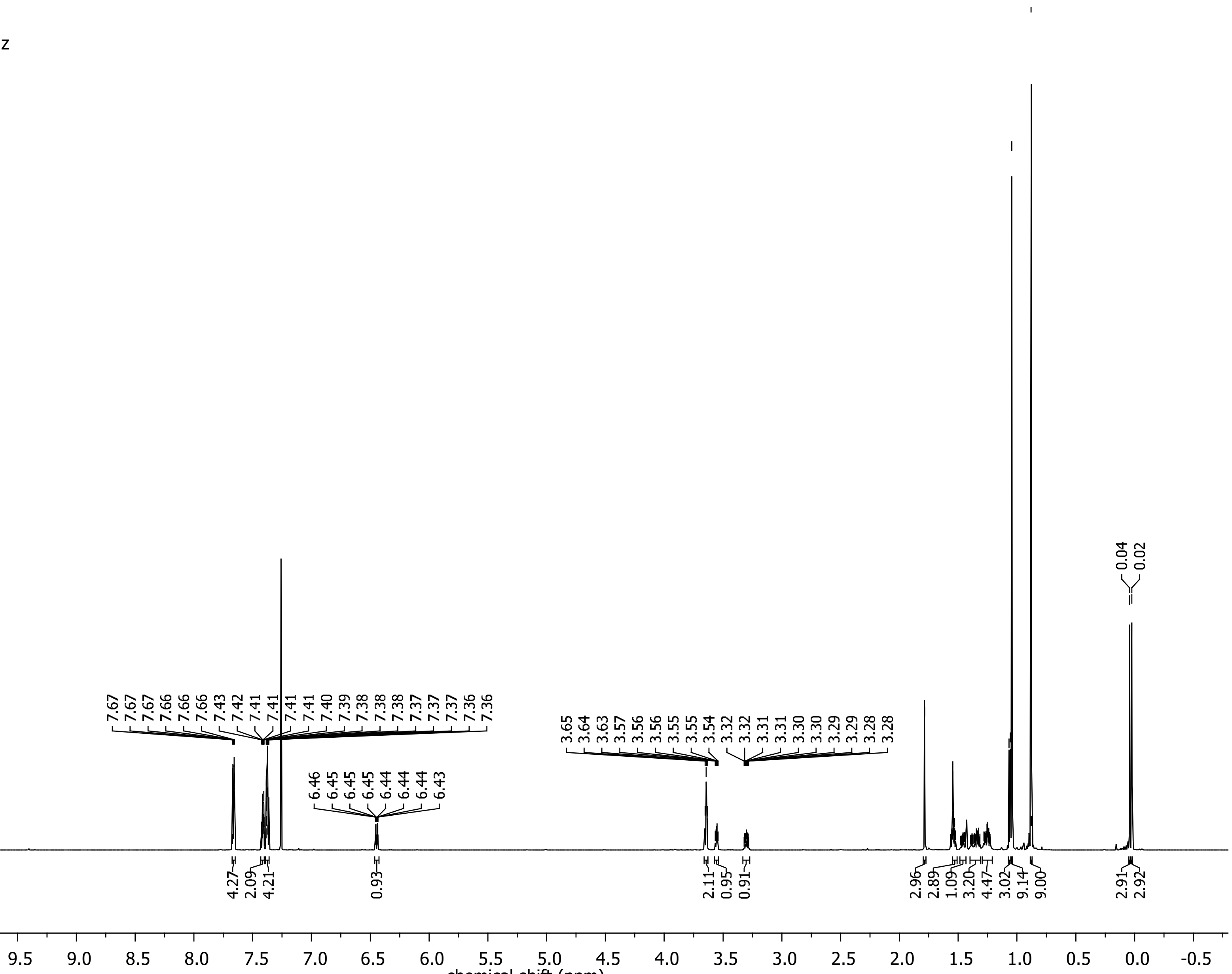

Nucleus: 13C
Frequency: 176.12 MHz
Solvent: CDCl3
Temperature: 298.0 K

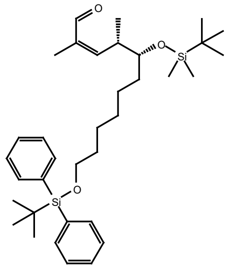

34

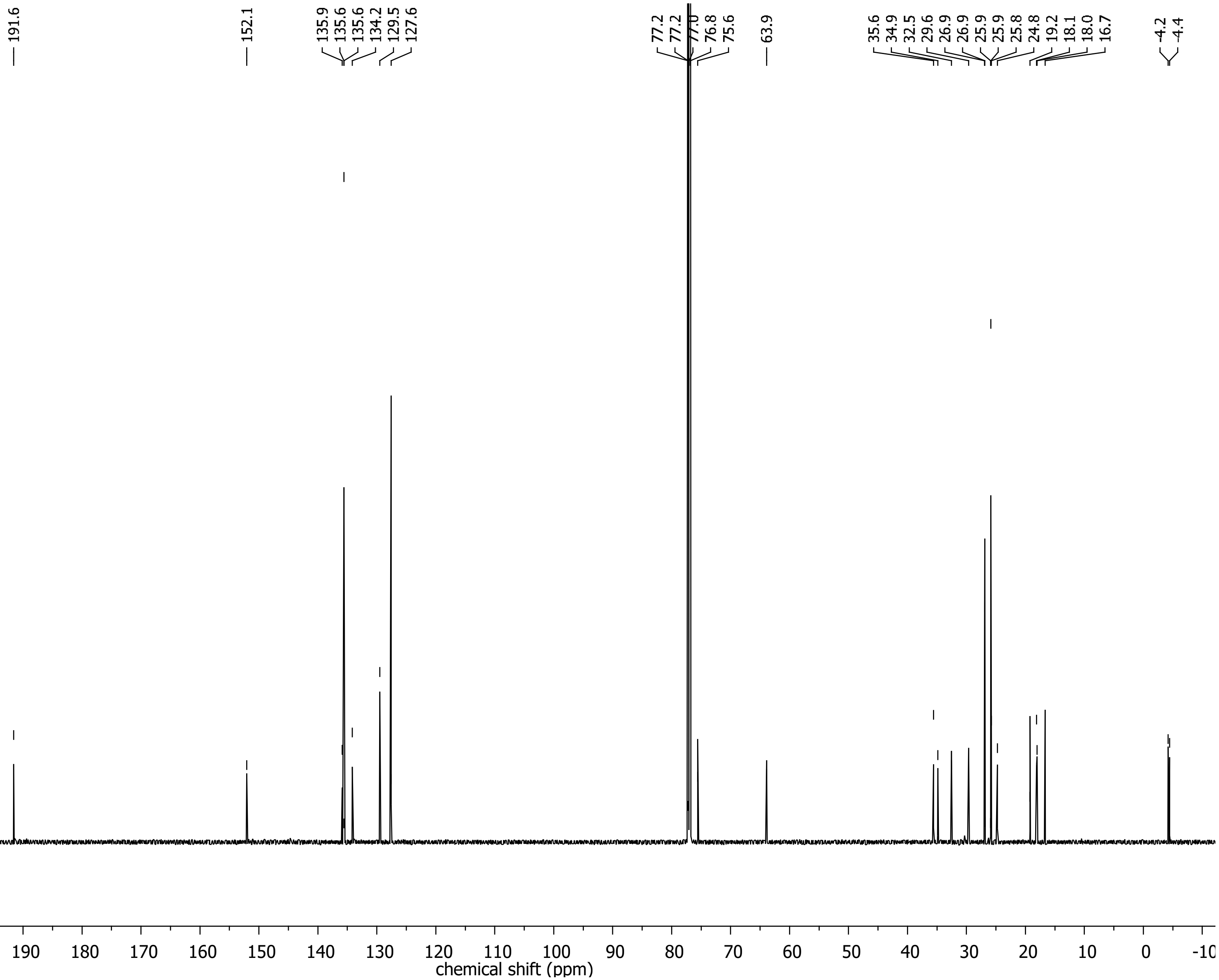

Nucleus: 1H
Frequency: 700.41 MHz
Solvent: CDCl3
Temperature: 298.0 K

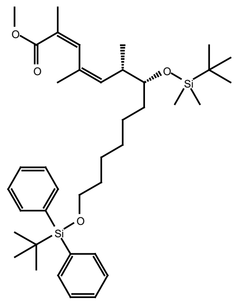

35

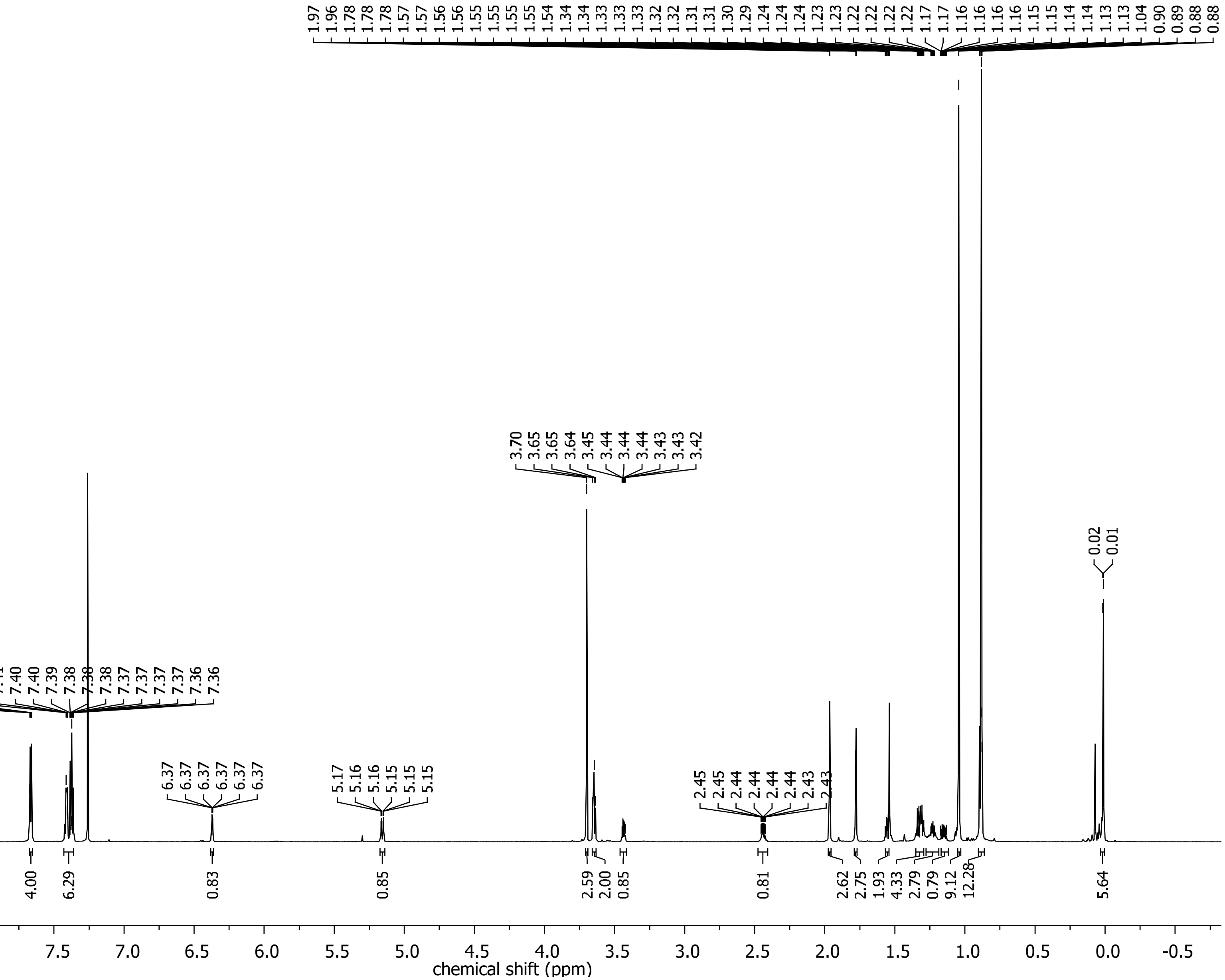

Nucleus: 13C
Frequency: 176.12 MHz
Solvent: CDCl3
Temperature: 298.0 K

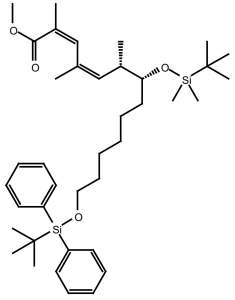

35

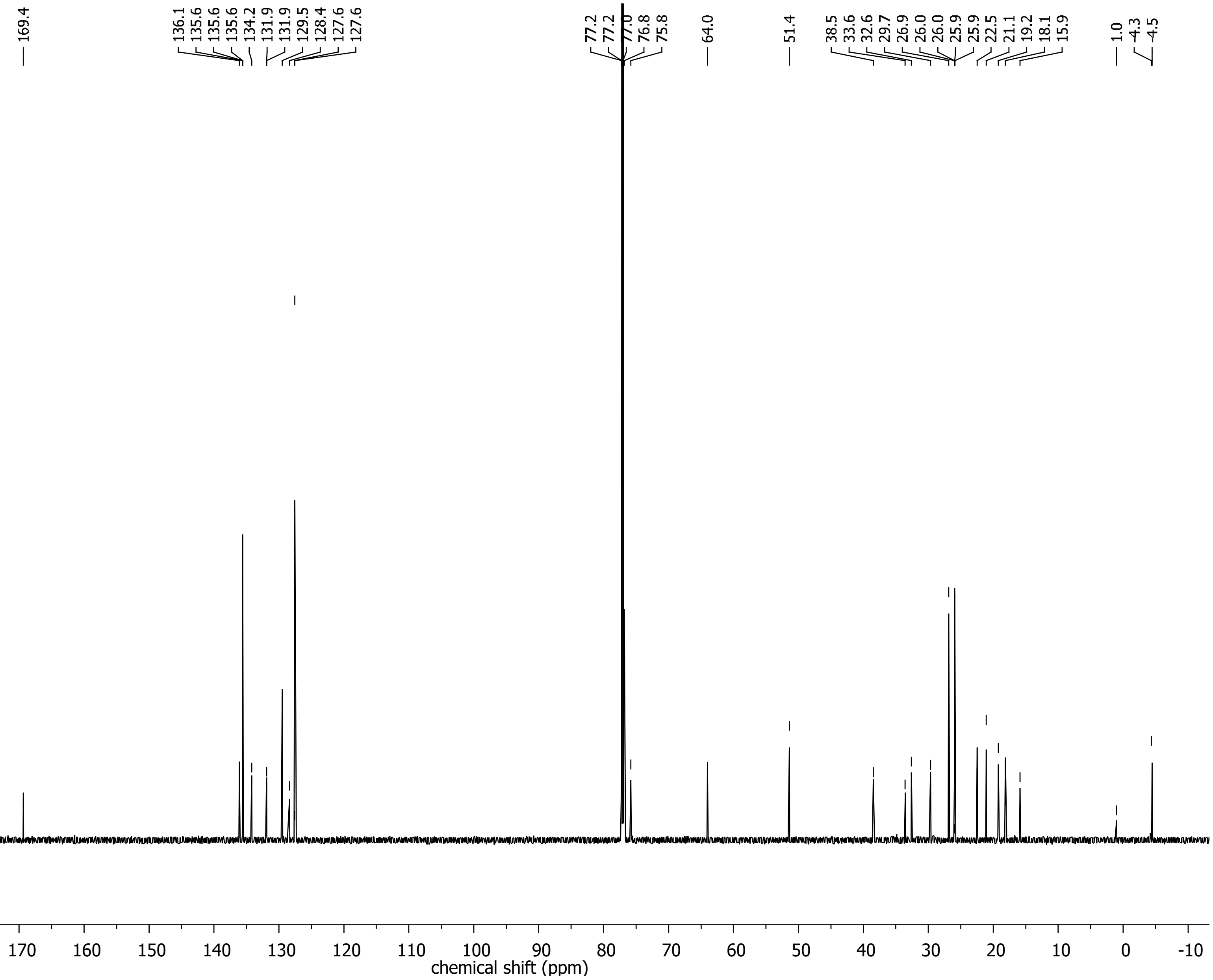

Nucleus: 1H
Frequency: 500.07 MHz
Solvent: CDCl3
Temperature: 298.0 K

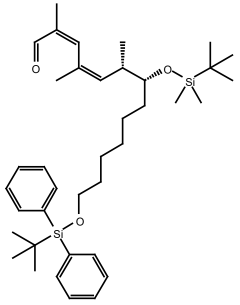

36

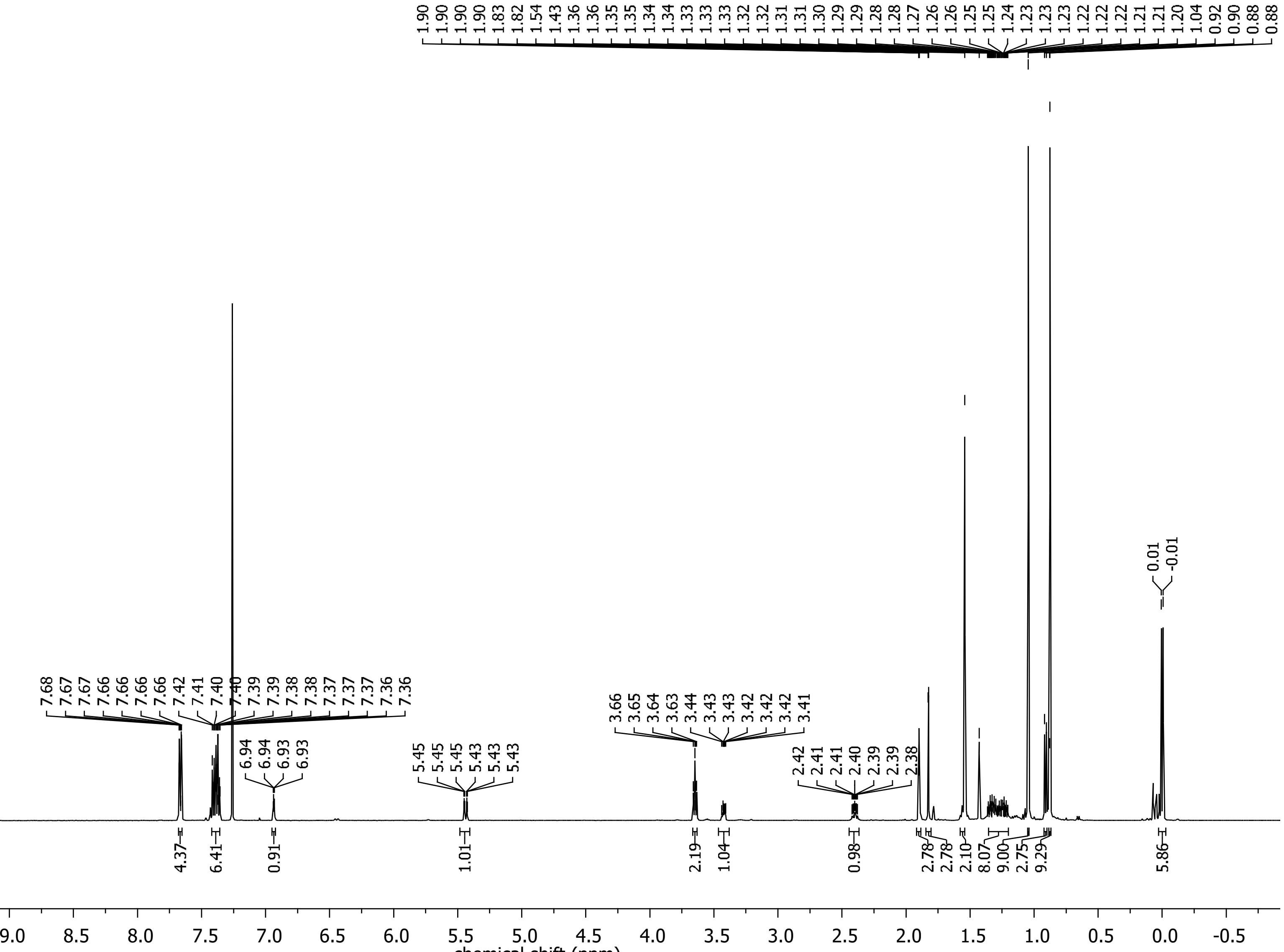

Nucleus: 13C
Frequency: 125.74 MHz
Solvent: CDCl3
Temperature: 298.0 K

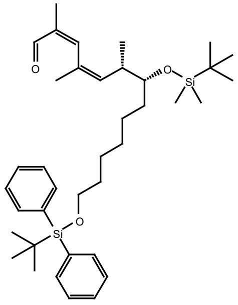

36

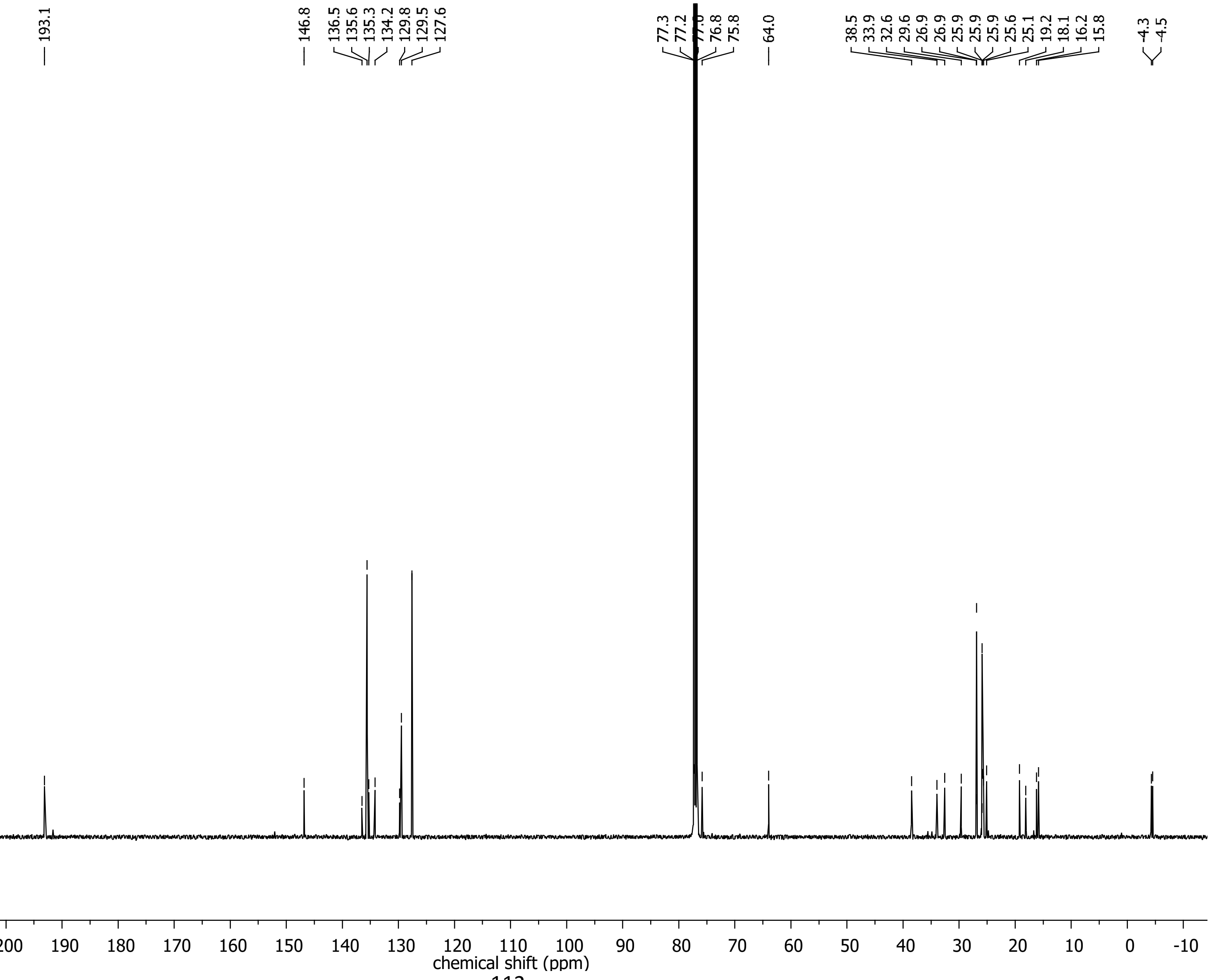

Nucleus: 1H
Frequency: 500.07 MHz
Solvent: CDCl3
Temperature: 298.0 K

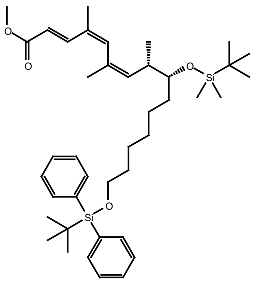

37

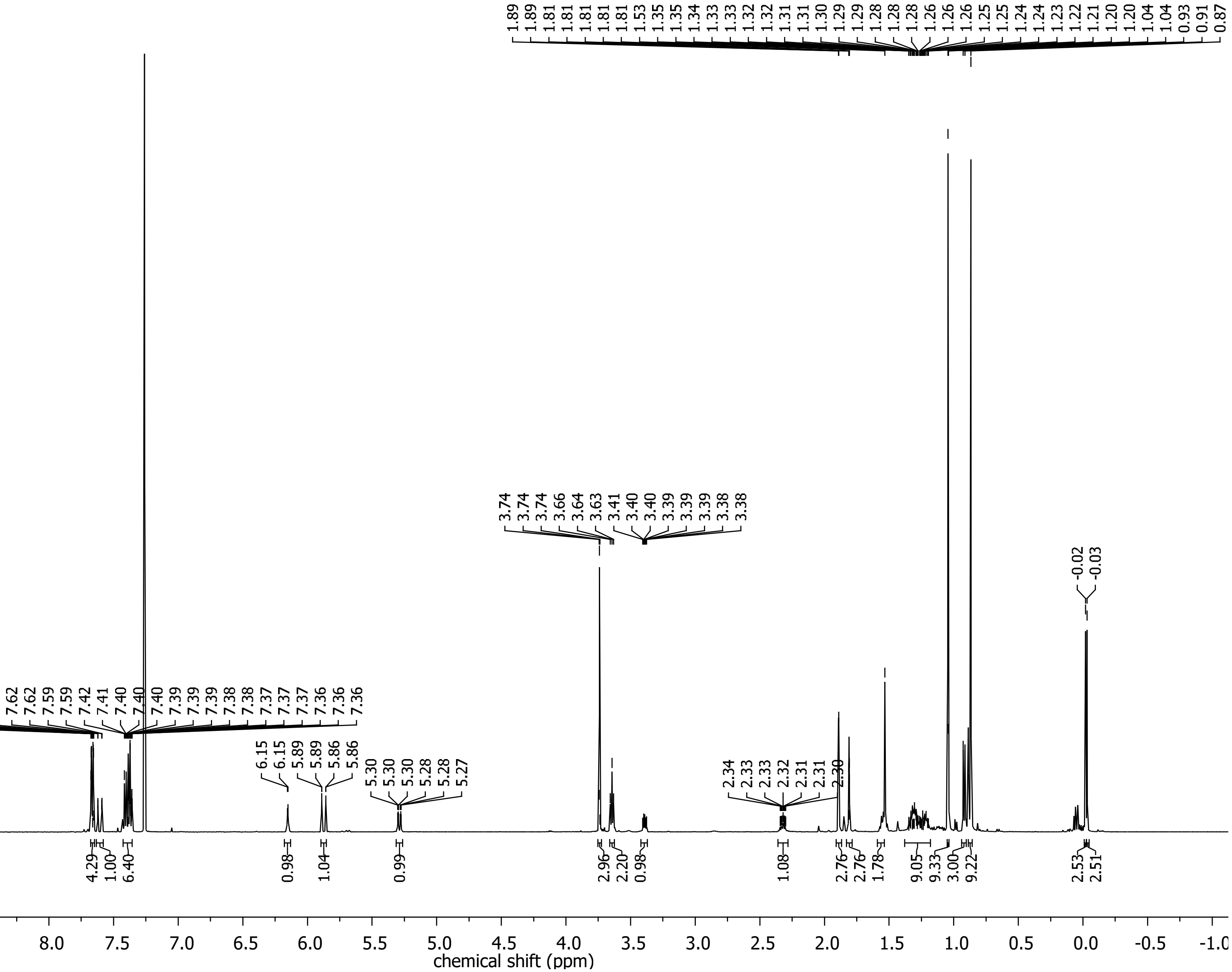

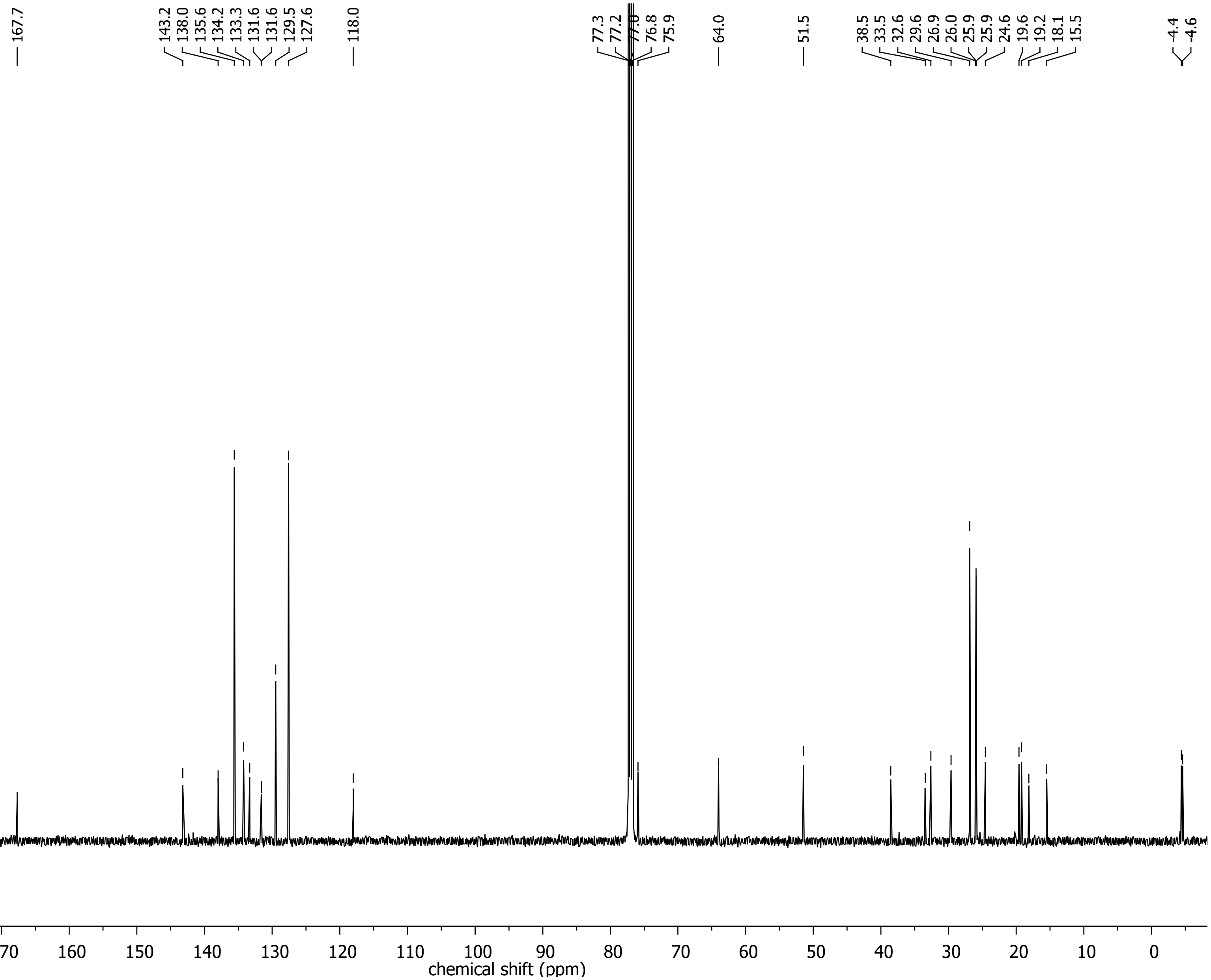
Nucleus: 13C
Frequency: 125.74 MHz
Solvent: CDCl3
Temperature: 298.0 K
167.7
143.2
138.0
135.6
134.2
133.3
131.6
131.6
129.5
127.6
118.0
77.3
77.2
77.0
76.8
75.9
64.0
51.5
38.5
33.5
32.6
29.6
26.9
26.0
25.9
25.9
24.6
19.6
19.2
18.1
15.5
-4.4
-4.6
190 180 170 160 150 140 130 120 110 100 90 80 70 60 50 40 30 20 10 0
chemical shift (ppm)

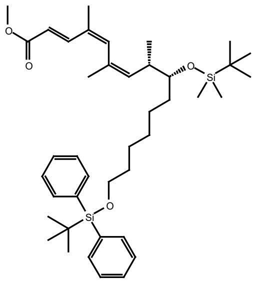

37

Nucleus: 1H
Frequency: 300.13 MHz
Solvent: CDCl3
Temperature: 298.0 K

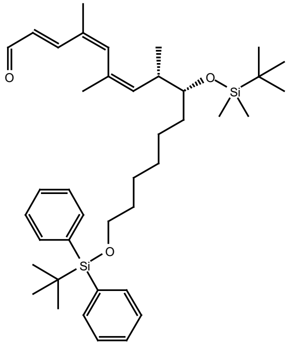

38

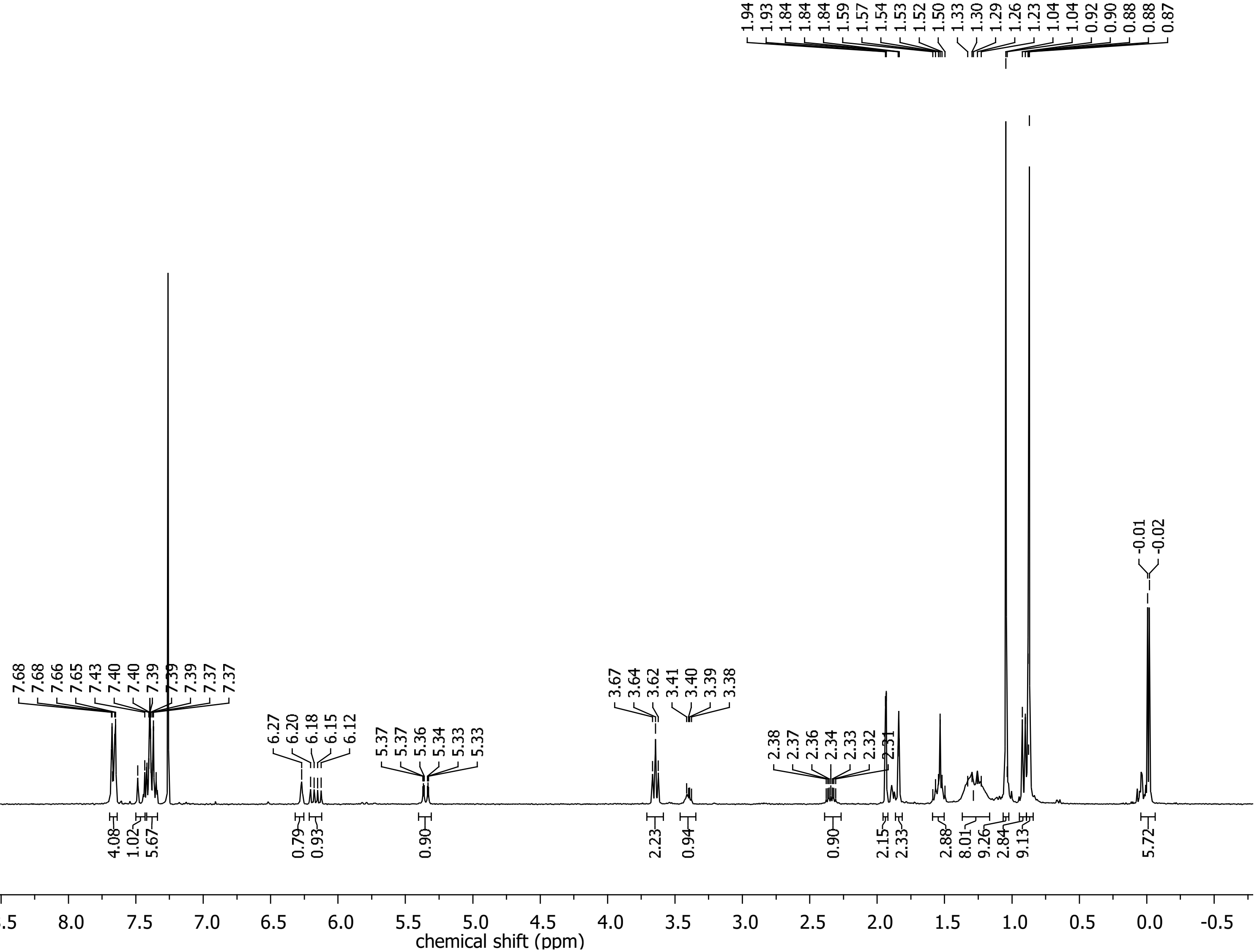

Nucleus: 13C
Frequency: 176.12 MHz
Solvent: CDCl3
Temperature: 298.0 K

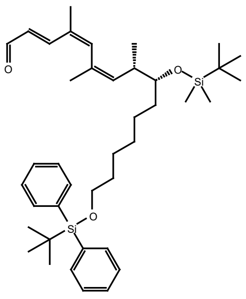

38

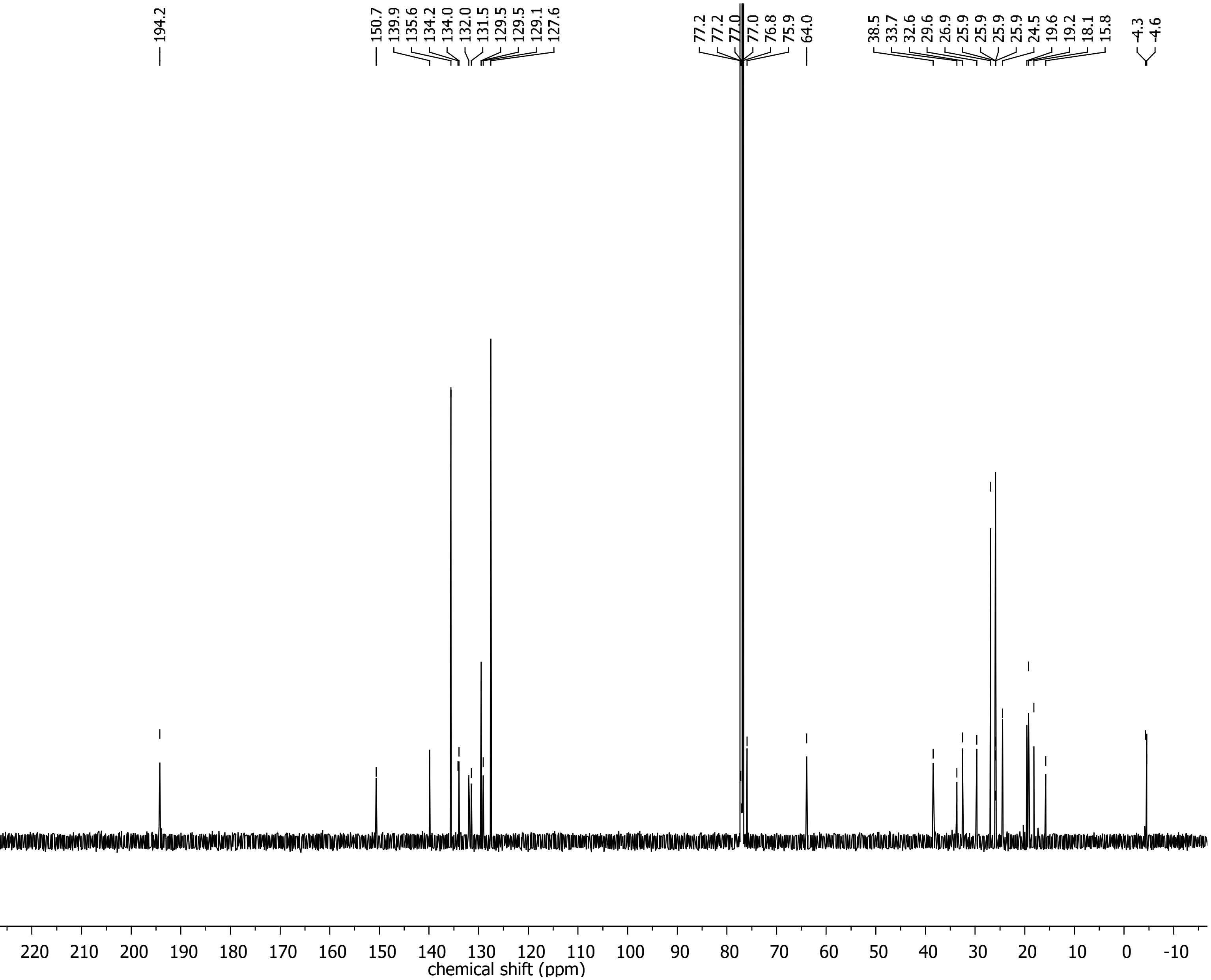

Nucleus: 1H
Frequency: 700.41 MHz
Solvent: CDCl3
Temperature: 298.0 K

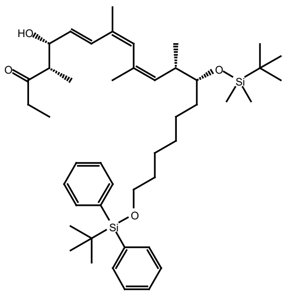

56

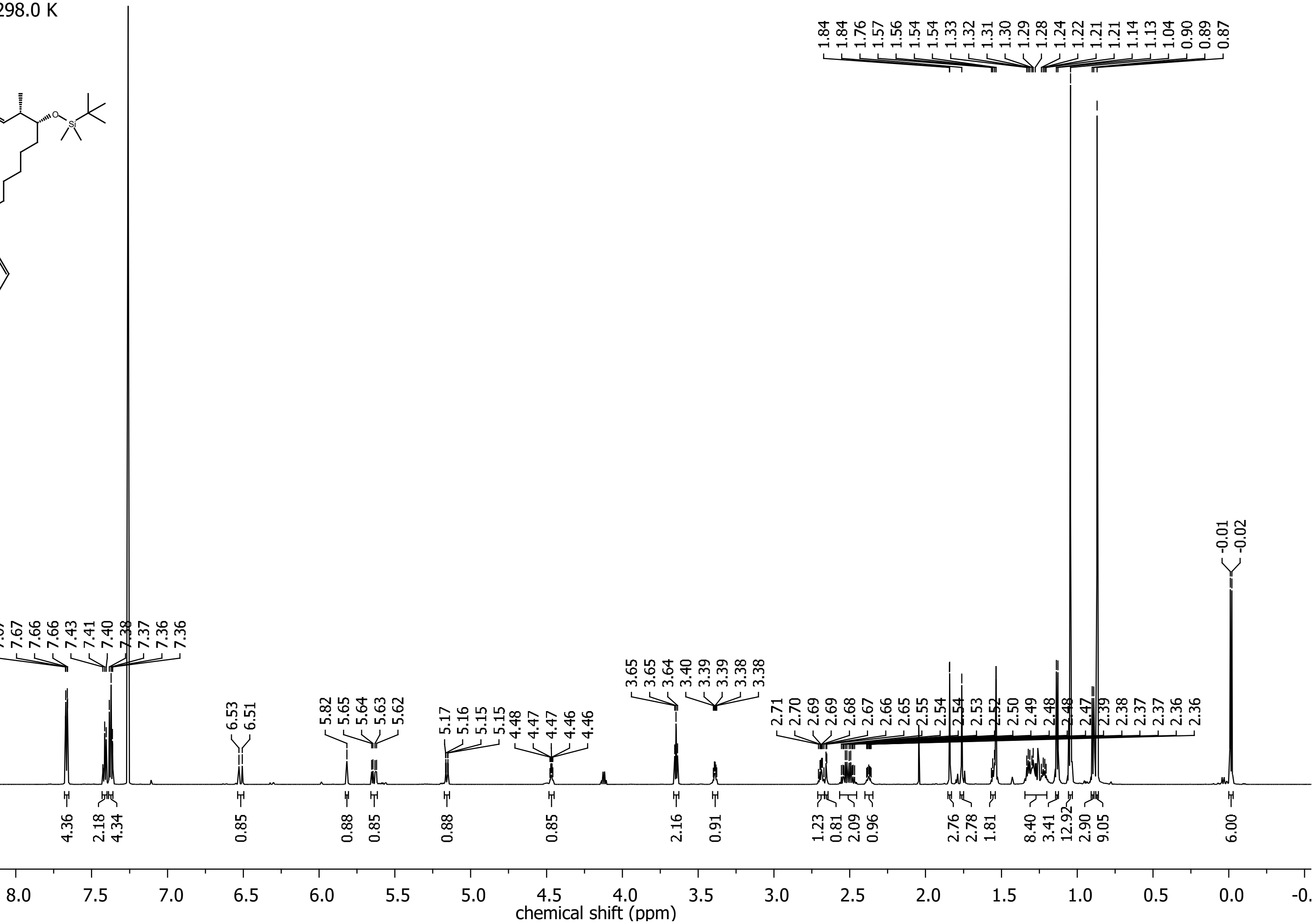

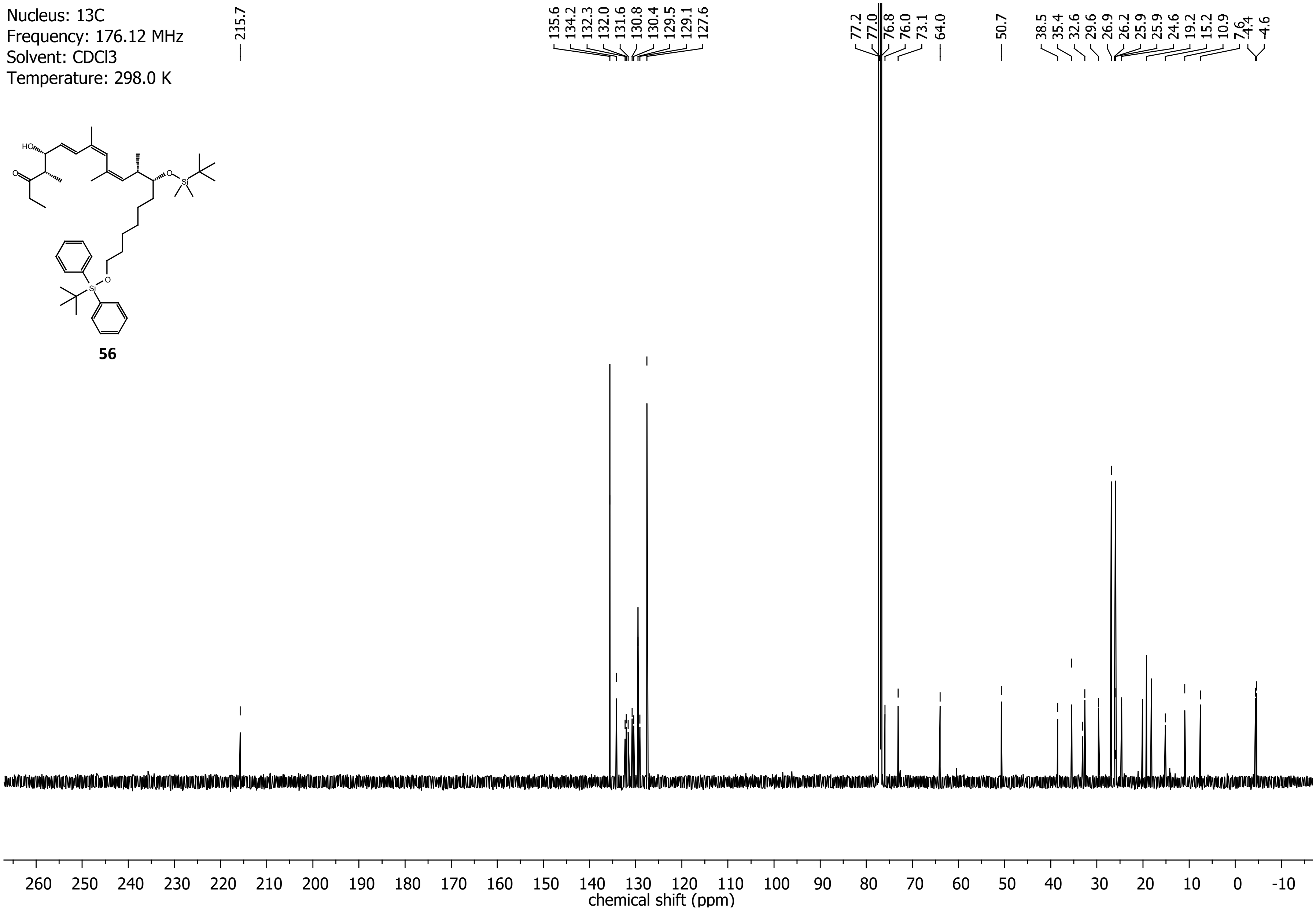
Nucleus: 13C
Frequency: 176.12 MHz
Solvent: CDCl3
Temperature: 298.0 K
HO
O
O
Si
O
Si
56
215.7
135.6
134.2
132.3
132.0
131.6
130.8
130.4
129.5
129.1
127.6
77.2
77.0
76.8
76.0
73.1
64.0
50.7
38.5
35.4
32.6
29.6
26.9
26.2
25.9
25.9
24.6
19.2
15.2
10.9
7.6
-4.4
-4.6
260 250 240 230 220 210 200 190 180 170 160 150 140 130 120 110 100 90 80 70 60 50 40 30 20 10 0 -10
chemical shift (ppm)

Nucleus: 1H
Frequency: 300.13 MHz
Solvent: CDCl3
Temperature: 298.0 K

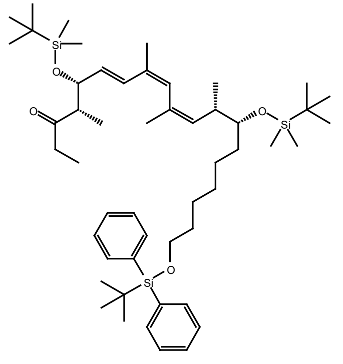

39

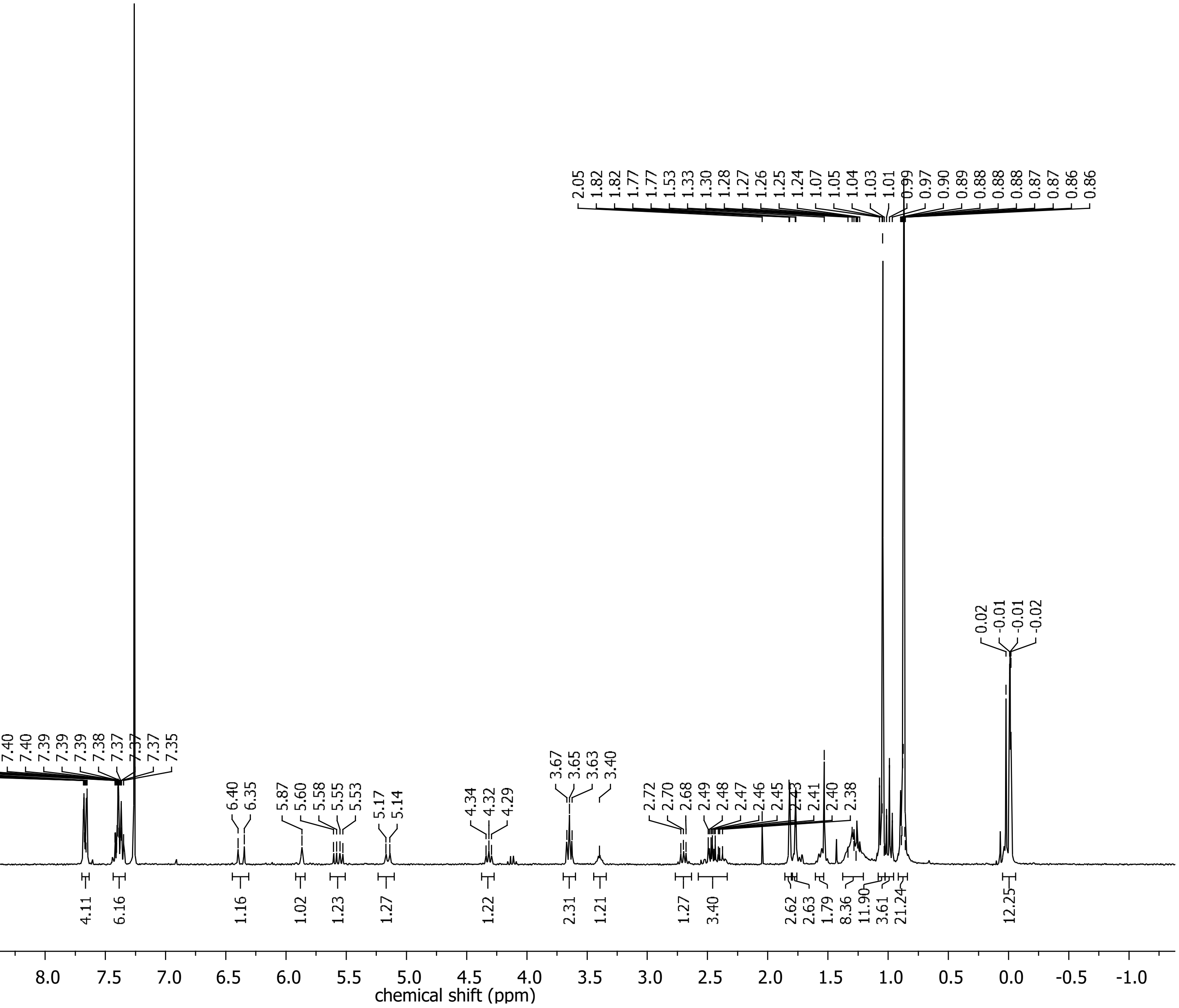

Nucleus: 13C
Frequency: 176.12 MHz
Solvent: CDCl3
Temperature: 298.0 K

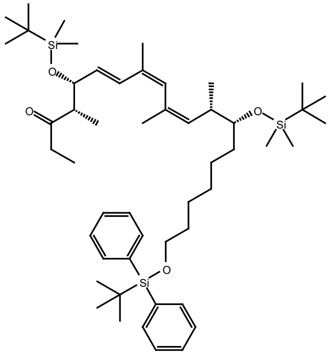

39

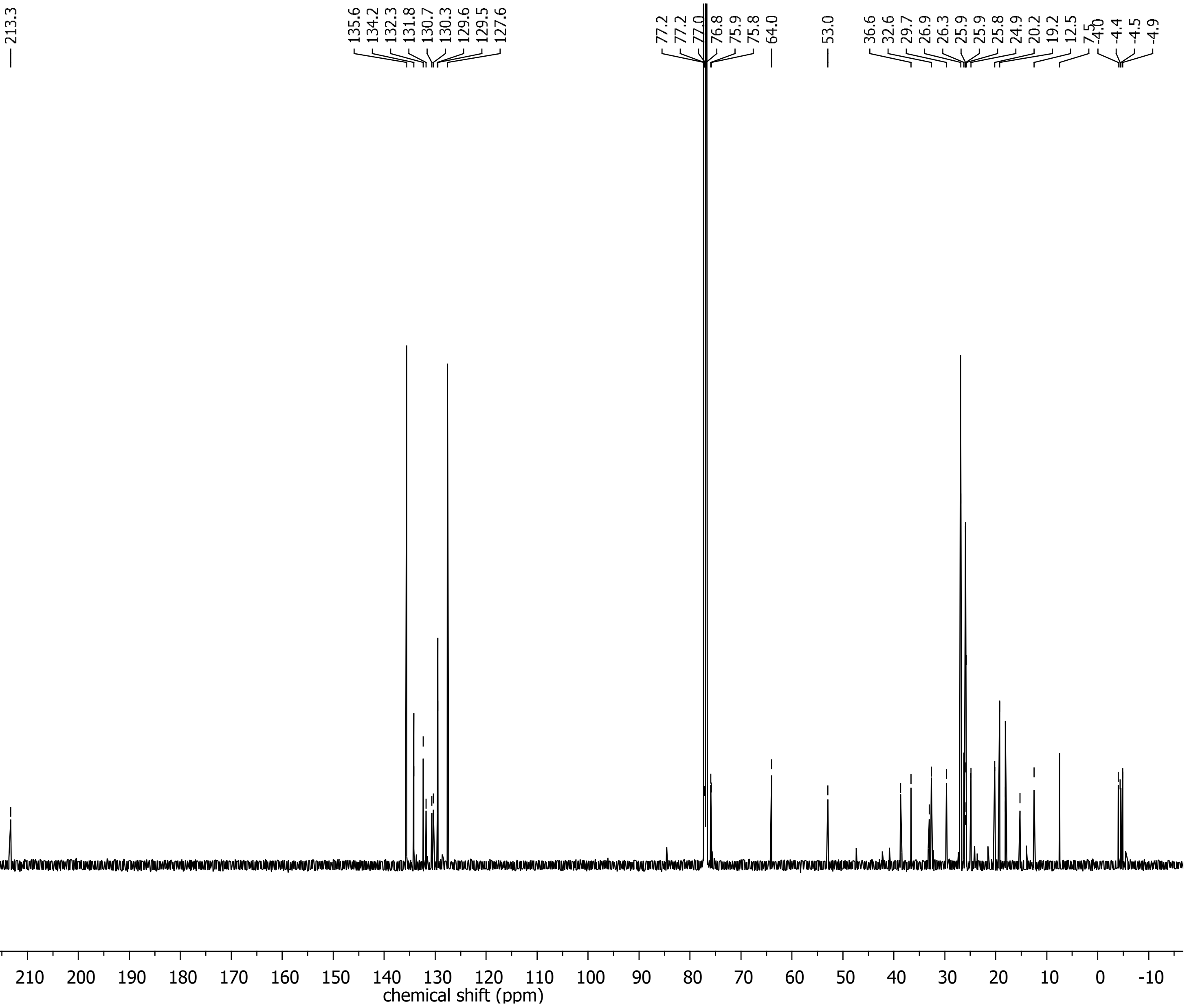

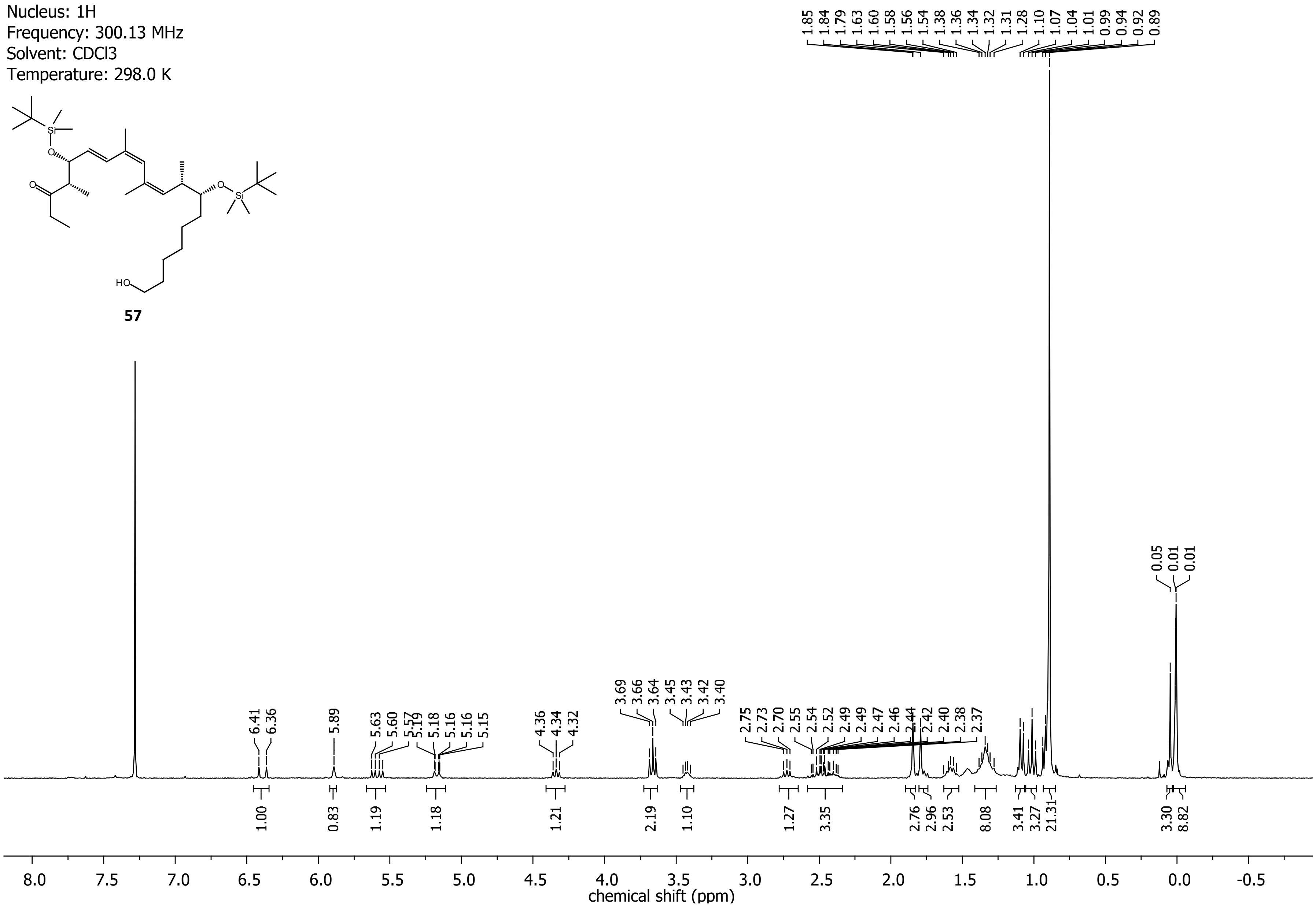
Nucleus: 1H
Frequency: 300.13 MHz
Solvent: CDCl3
Temperature: 298.0 K
57
8.0 7.5 7.0 6.5 6.0 5.5 5.0 4.5 4.0 3.5 3.0 2.5 2.0 1.5 1.0 0.5 0.0 -0.5
chemical shift (ppm)

Nucleus: 13C
Frequency: 176.12 MHz
Solvent: CDCl3
Temperature: 298.0 K

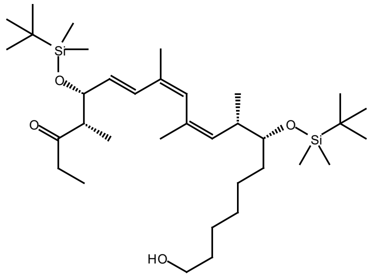

57

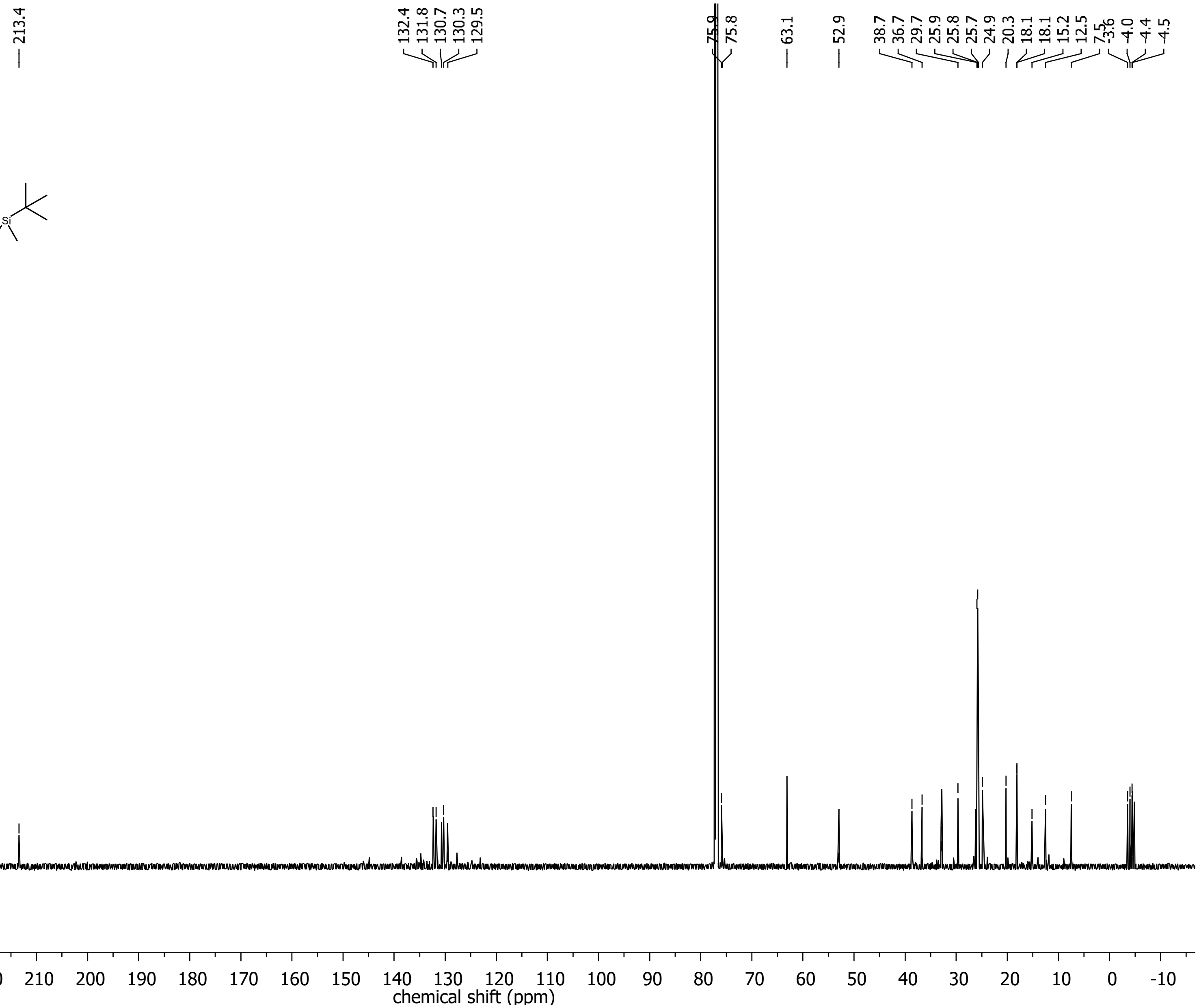

Nucleus: 1H
Frequency: 700.41 MHz
Solvent: CD2Cl2
Temperature: 298.0 K

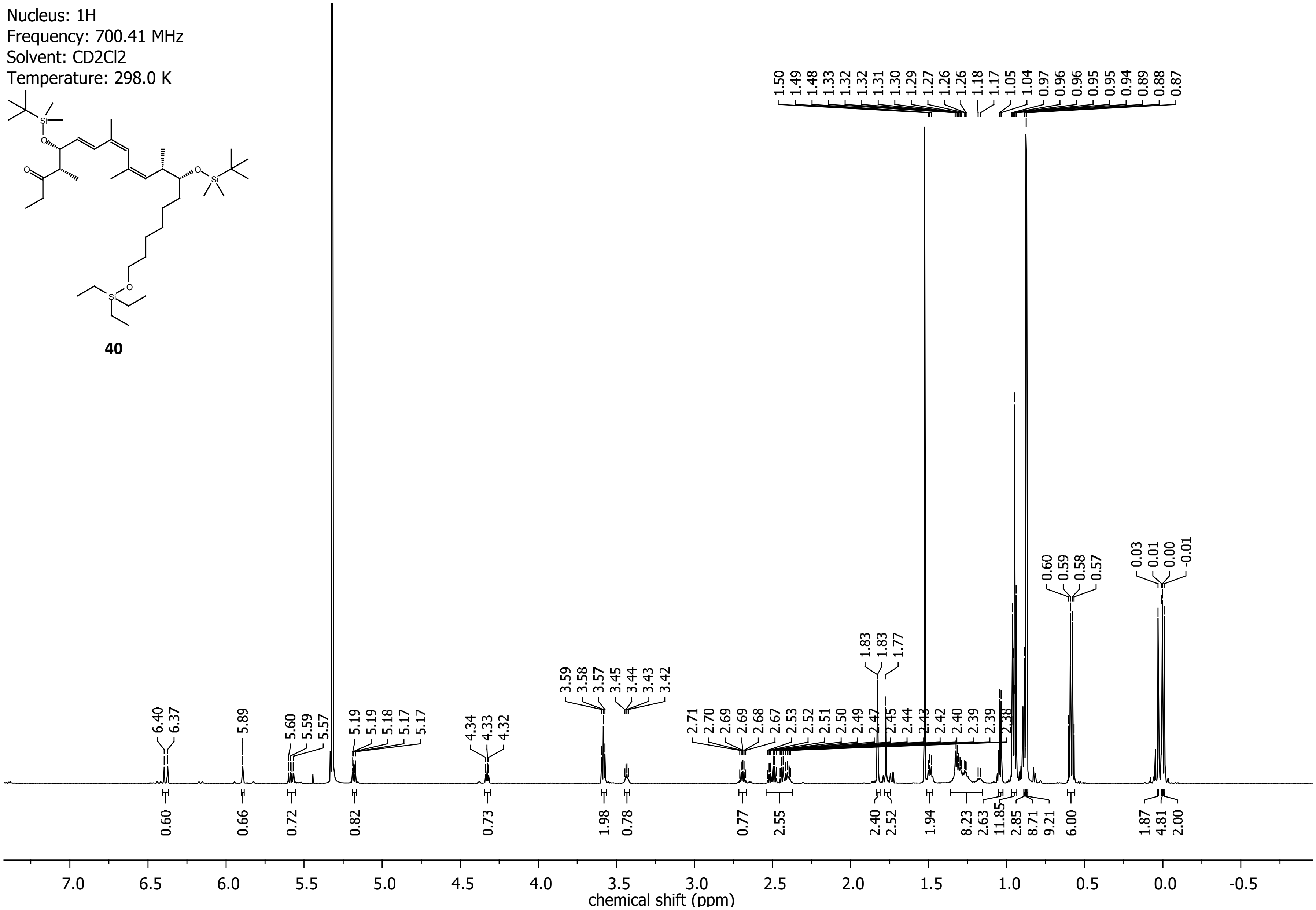

Nucleus: 13C
Frequency: 176.12 MHz
Solvent: CD2Cl2
Temperature: 298.0 K

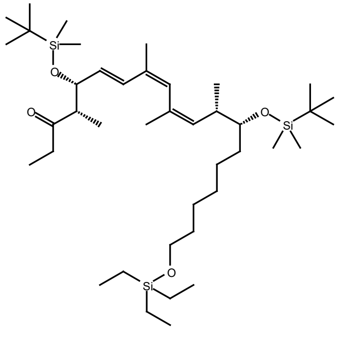

40

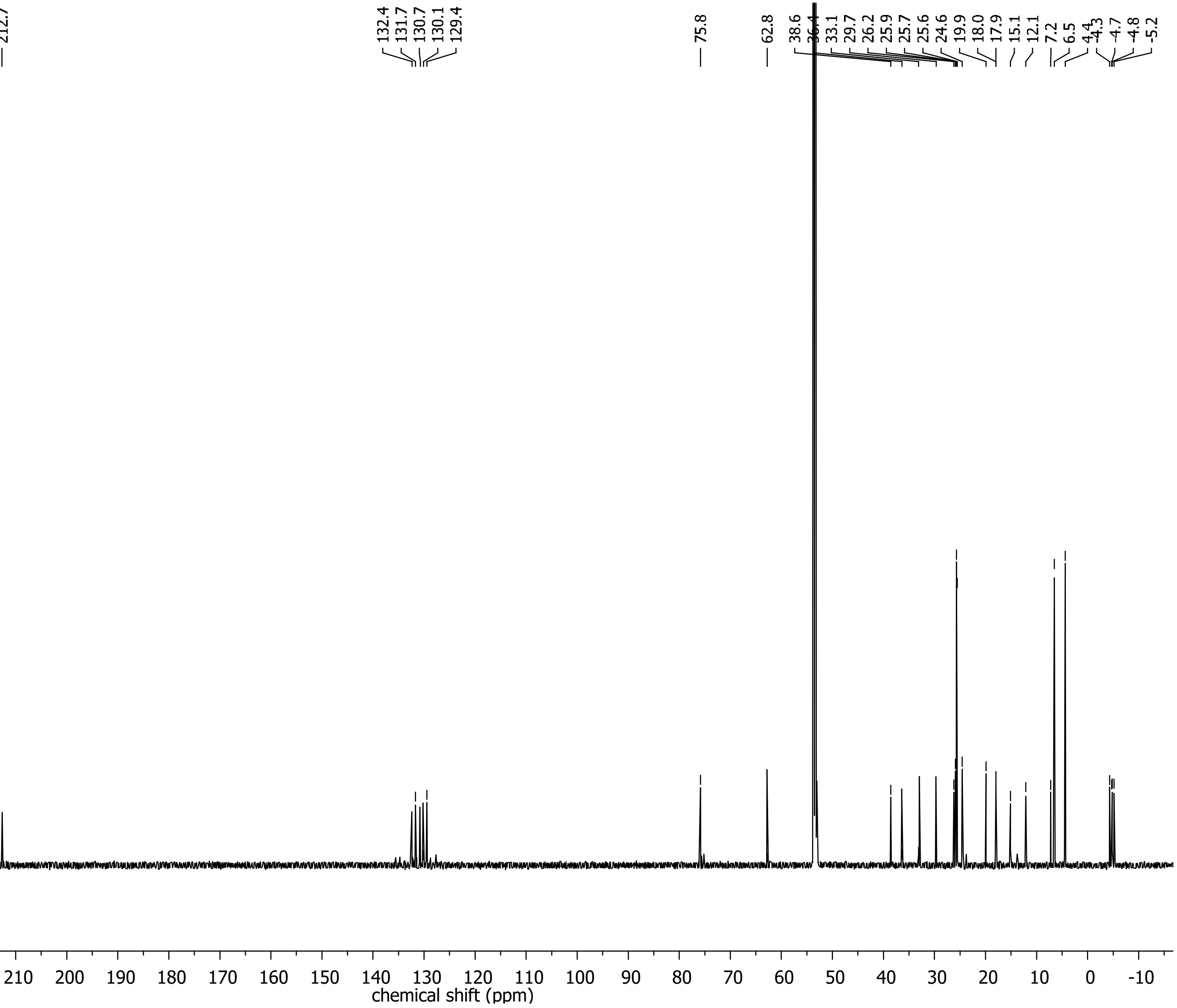

Nucleus: 1H
Frequency: 500.07 MHz
Solvent: CDCl3
Temperature: 298.0 K

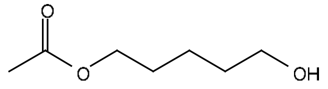

58

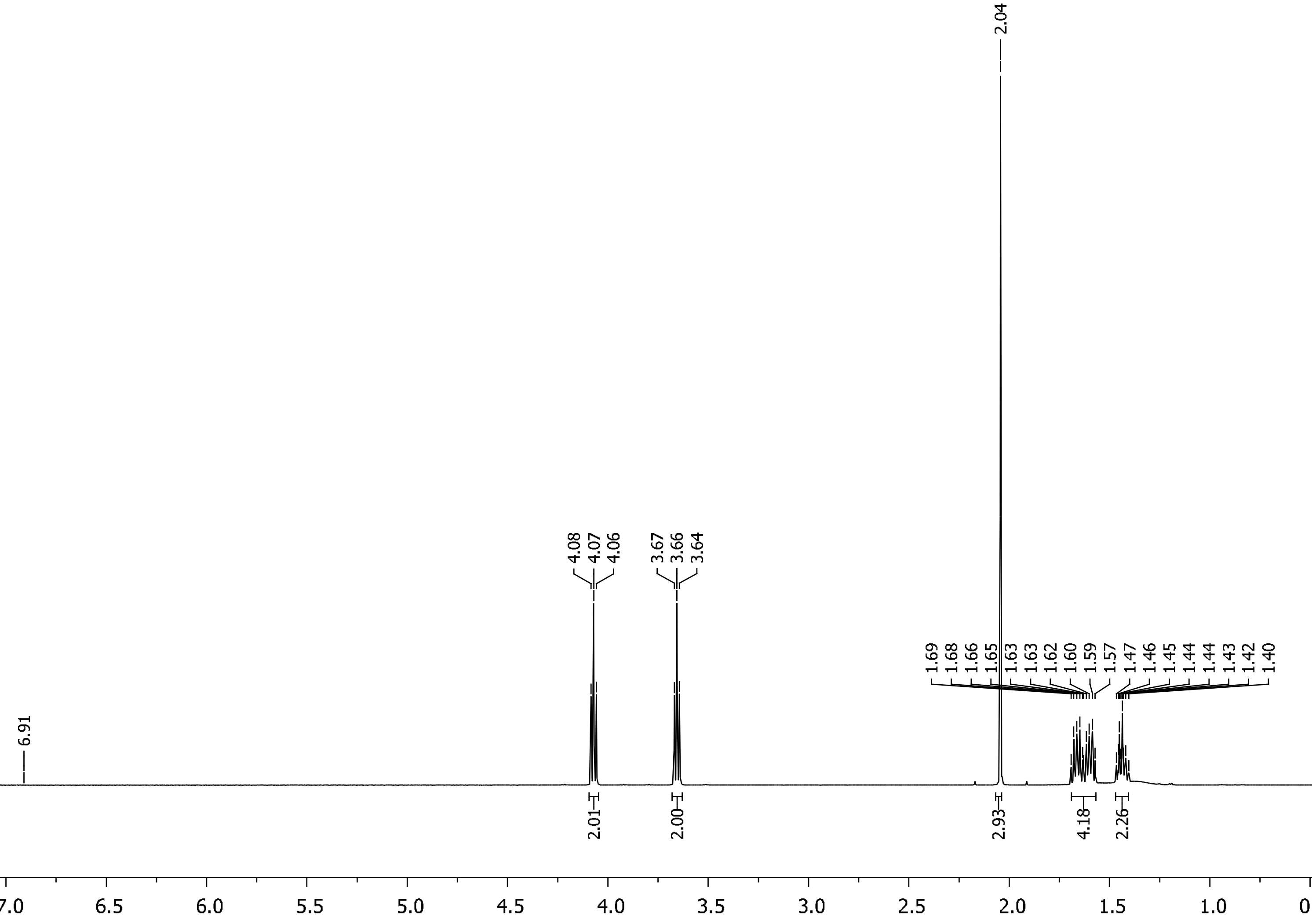

Nucleus: 13C
Frequency: 125.74 MHz
Solvent: CDCl3
Temperature: 298.0 K

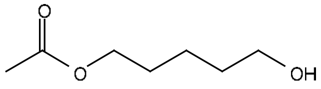

58

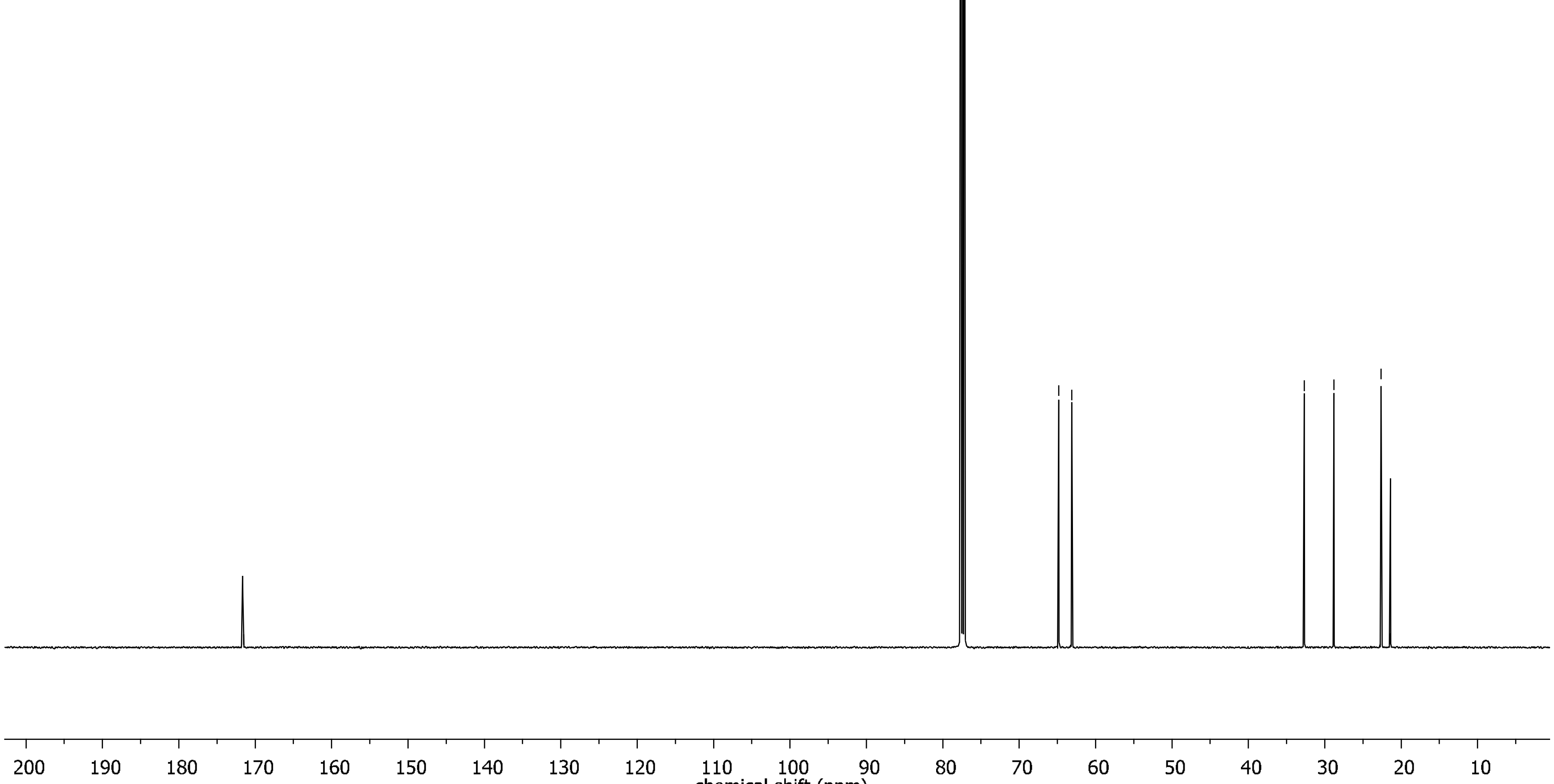

Nucleus: 1H
Frequency: 700.41 MHz
Solvent: CDCl3
Temperature: 298.0 K

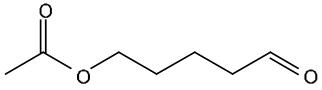

41

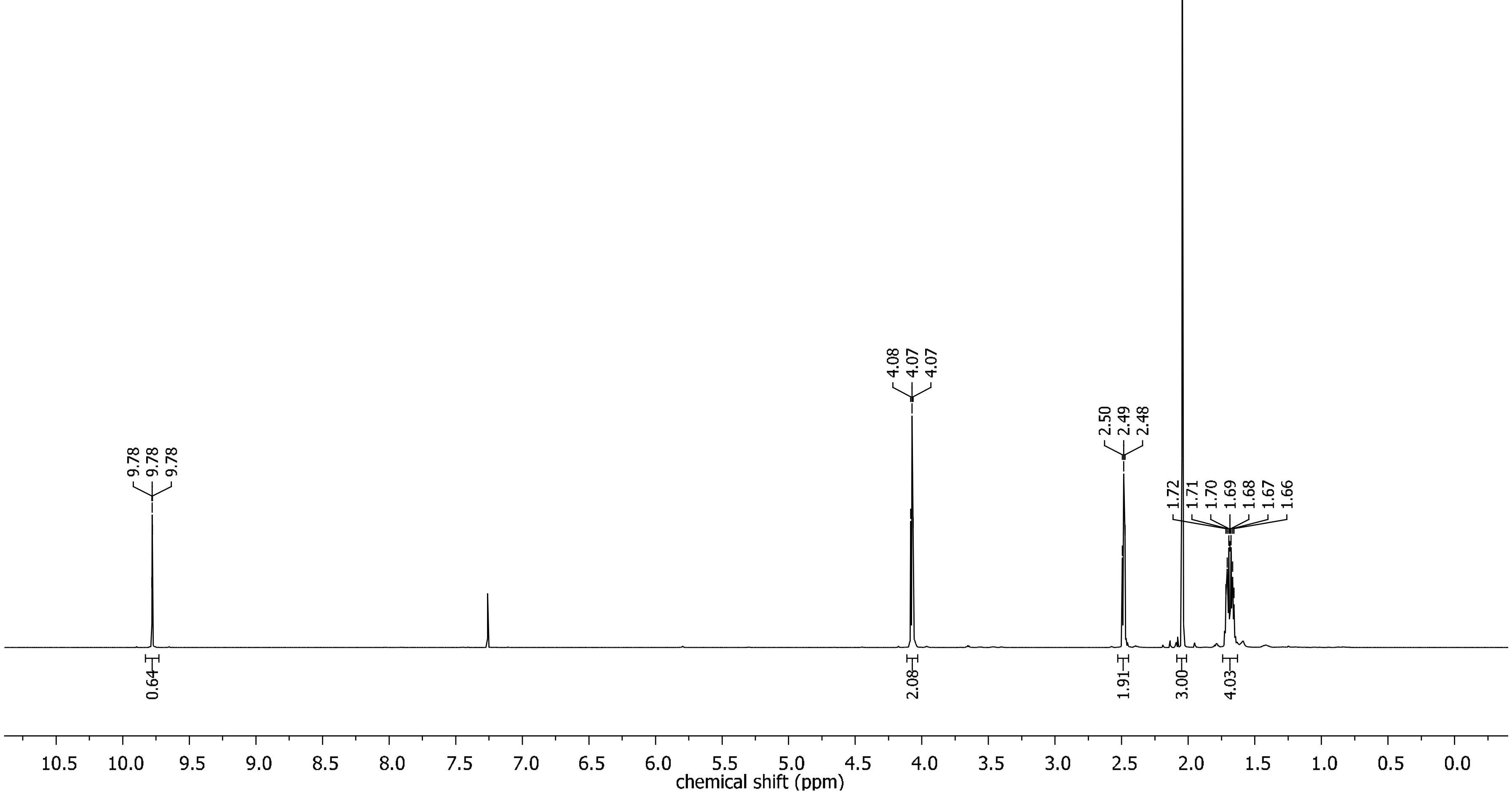

Nucleus: 13C
Frequency: 176.12 MHz
Solvent: CDCl3
Temperature: 298.0 K

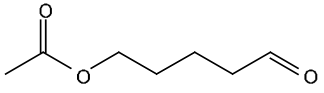

41

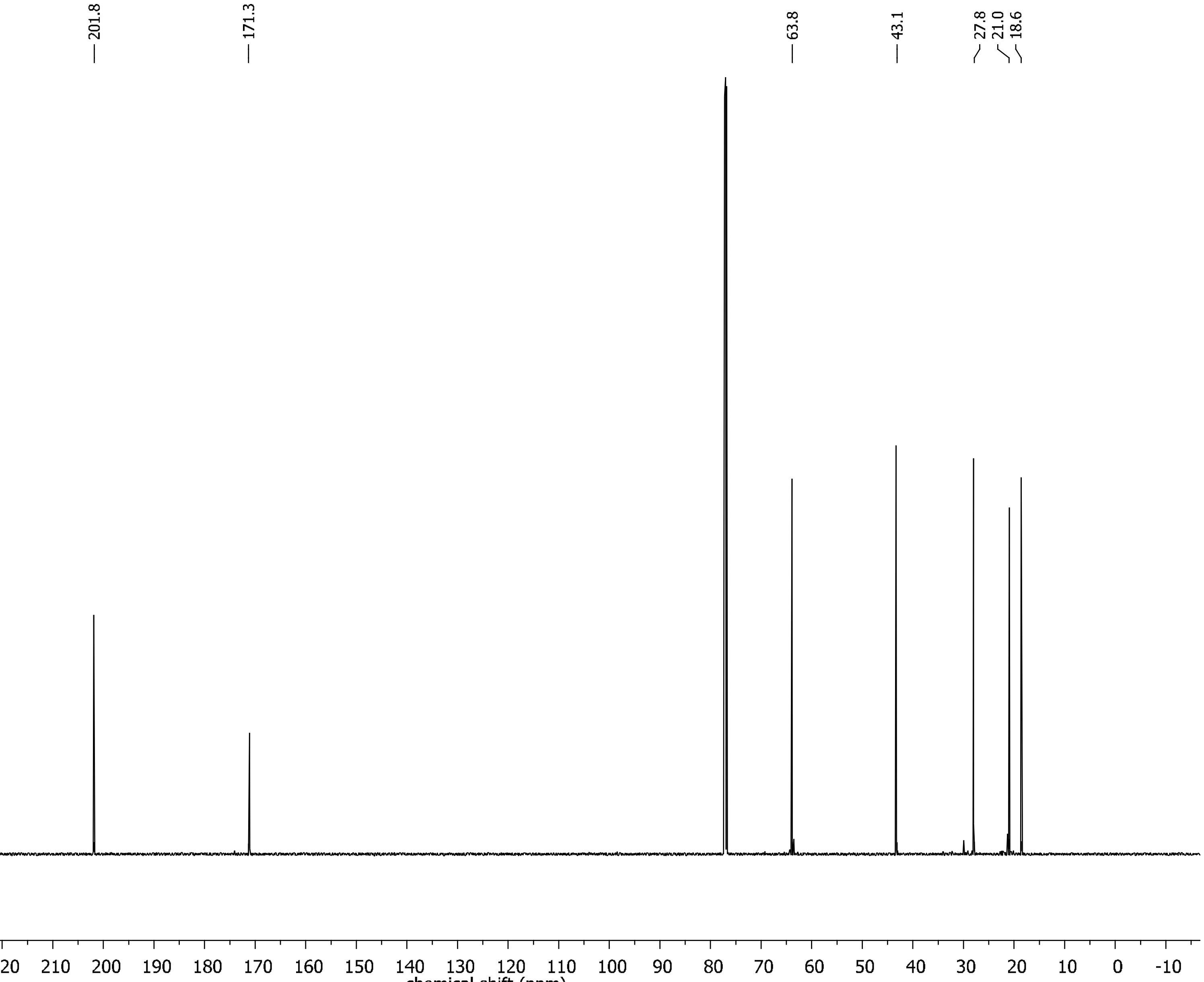

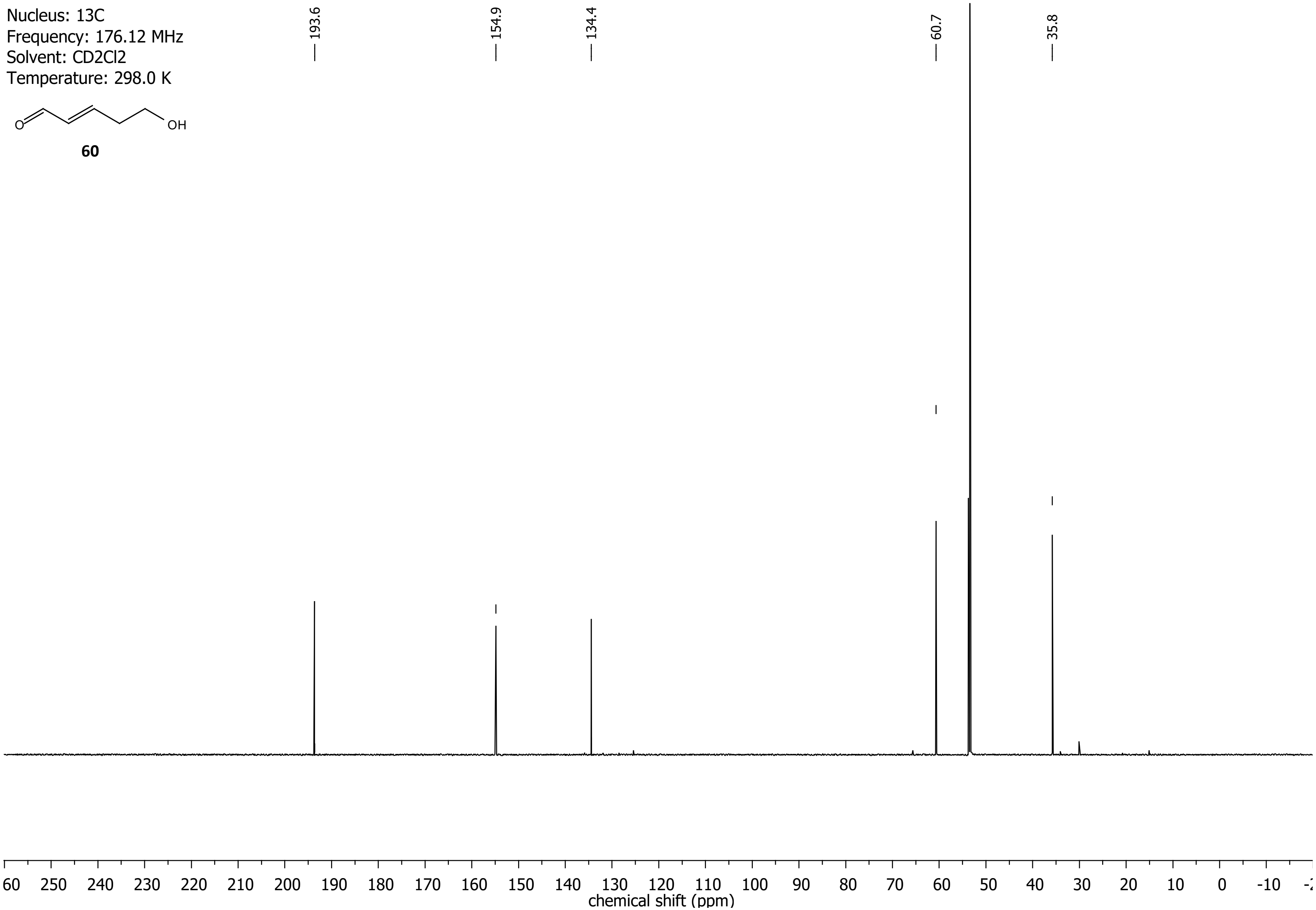
Nucleus: 13C
Frequency: 176.12 MHz
Solvent: CD2Cl2
Temperature: 298.0 K
OH
O
60
193.6
154.9
134.4
60.7
35.8
60 250 240 230 220 210 200 190 180 170 160 150 140 130 120 110 100 90 80 70 60 50 40 30 20 10 0 -10
chemical shift (ppm)

Nucleus: 13C
Frequency: 176.12 MHz
Solvent: CD2Cl2
Temperature: 298.0 K

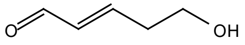

60

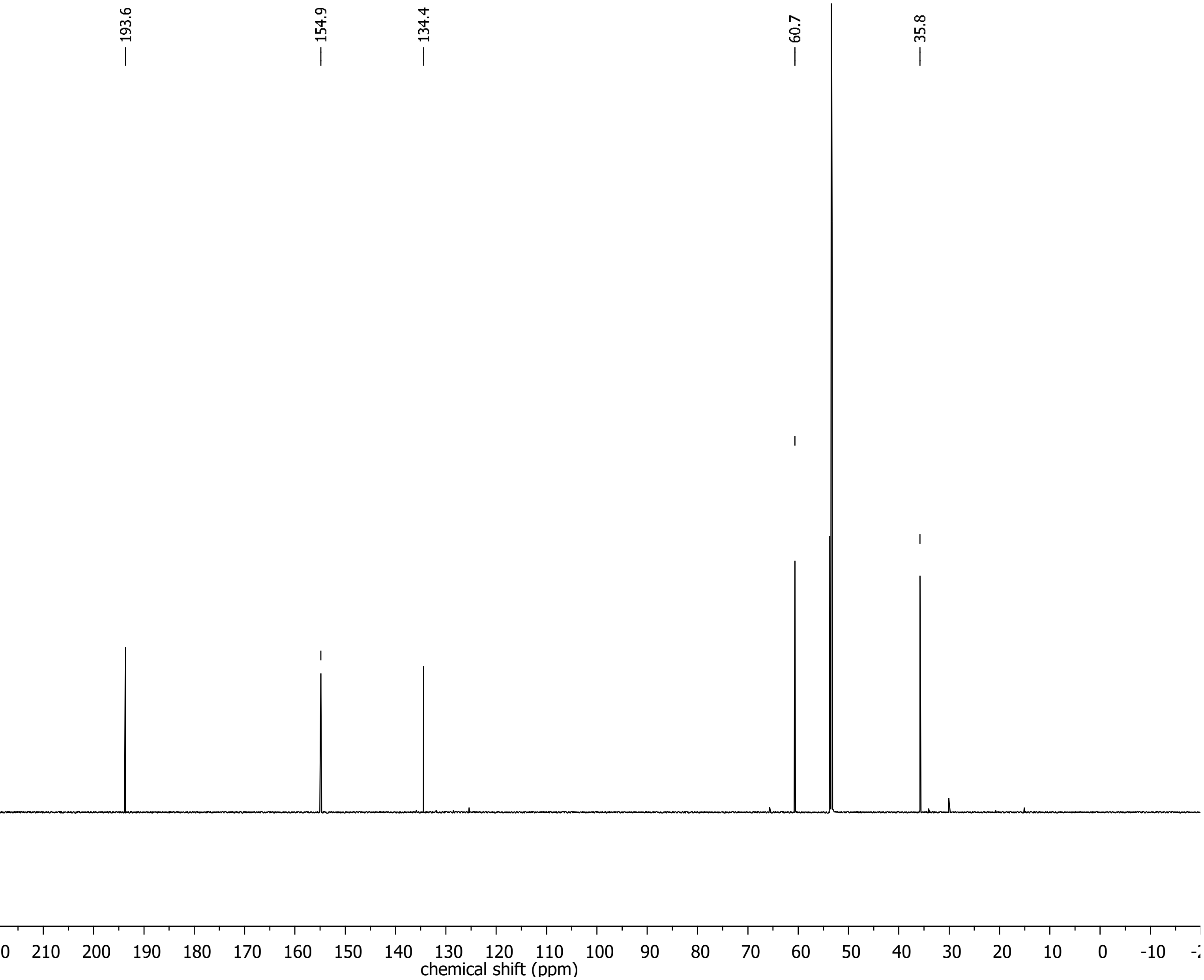

Nucleus: 1H
Frequency: 499.13 MHz
Solvent: CDCl3
Temperature: 297.9 K

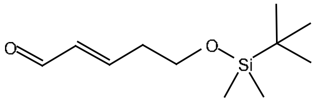

42

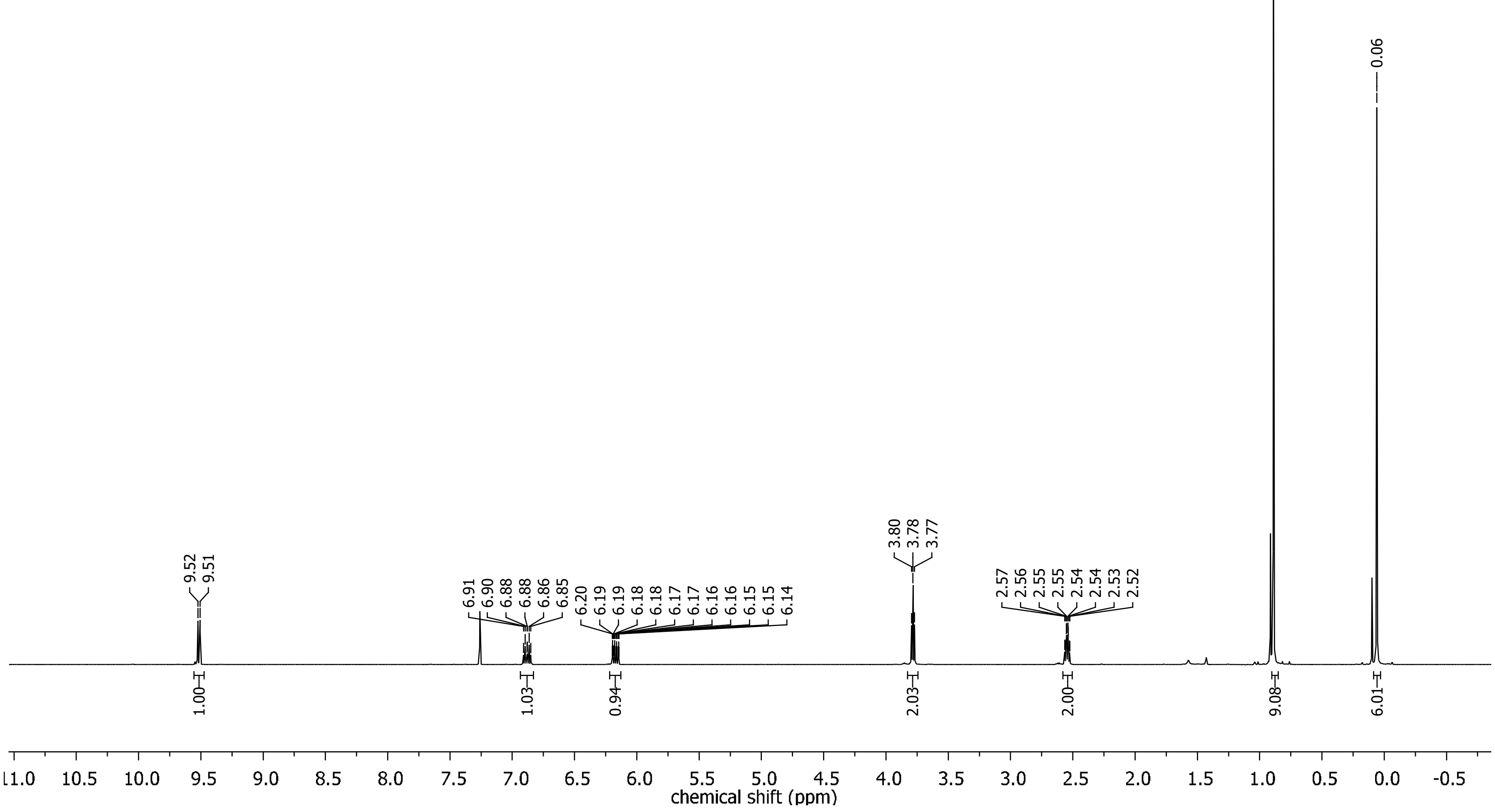

Nucleus: 13C
Frequency: 125.51 MHz
Solvent: CDCl3
Temperature: 298.0 K

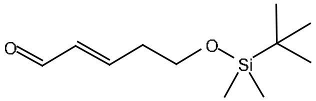

42

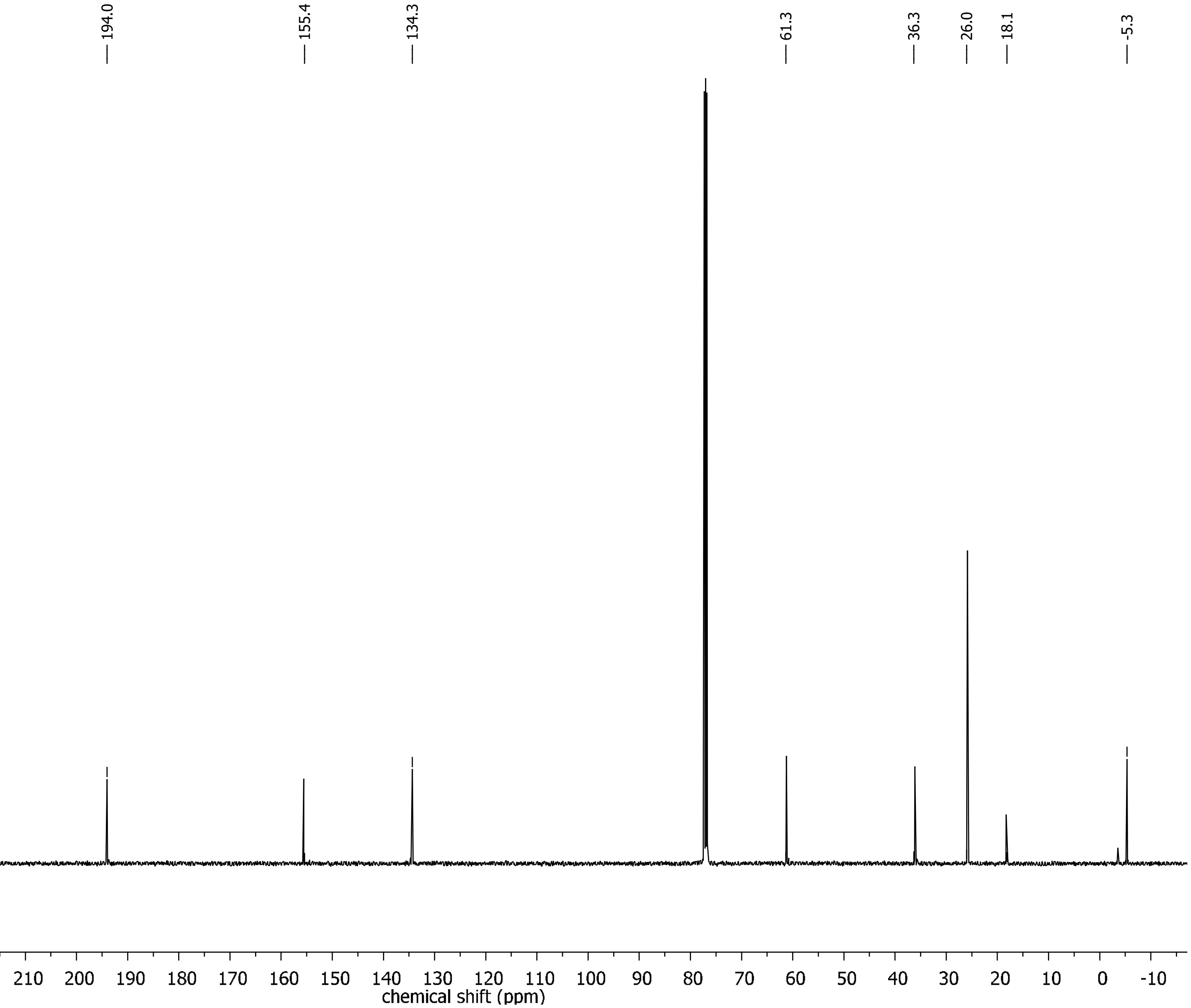

Nucleus: 1H
Frequency: 500.14 MHz
Solvent: CD2Cl2
Temperature: 298.0 K

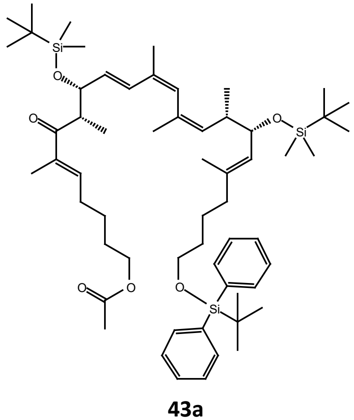

43a

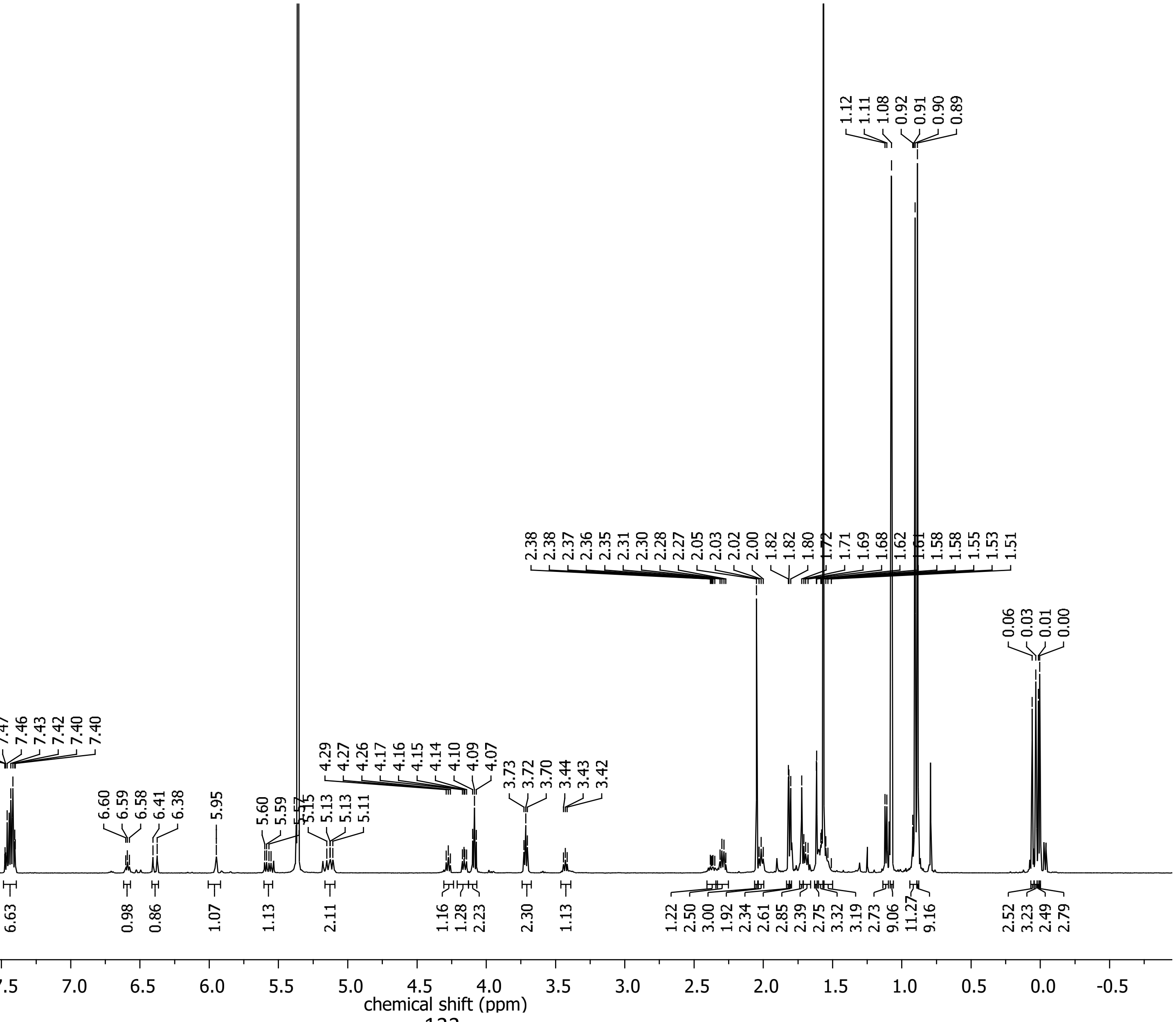

Nucleus: 13C
Frequency: 125.76 MHz
Solvent: CD2Cl2
Temperature: 298.0 K

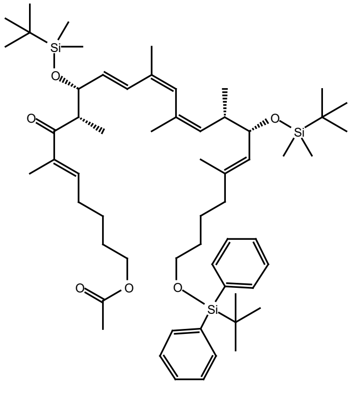

43a

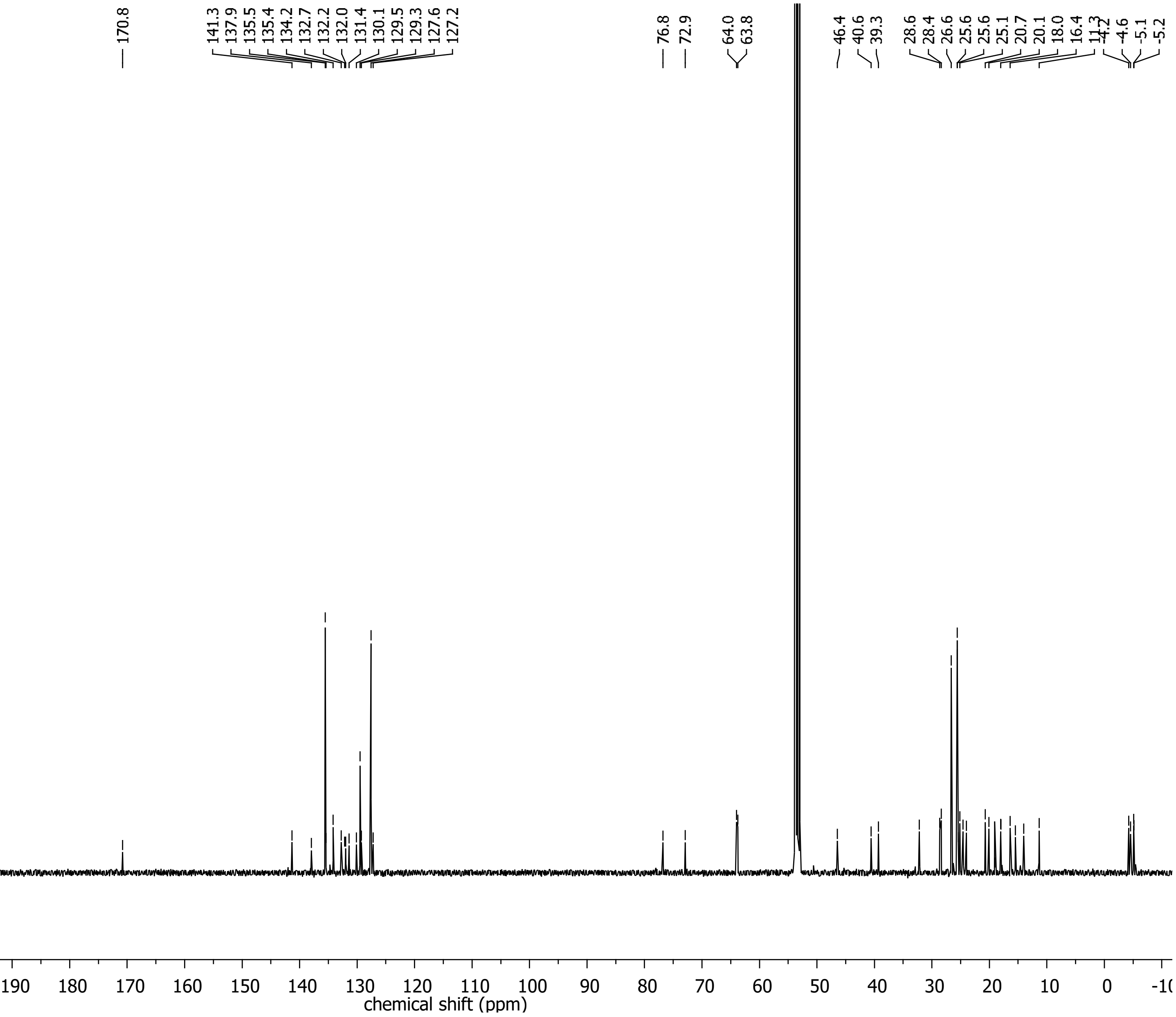

Nucleus: 1H
Frequency: 700.41 MHz
Solvent: CD2Cl2
Temperature: 298.0 K

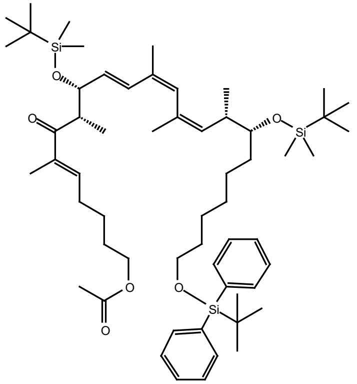

43b

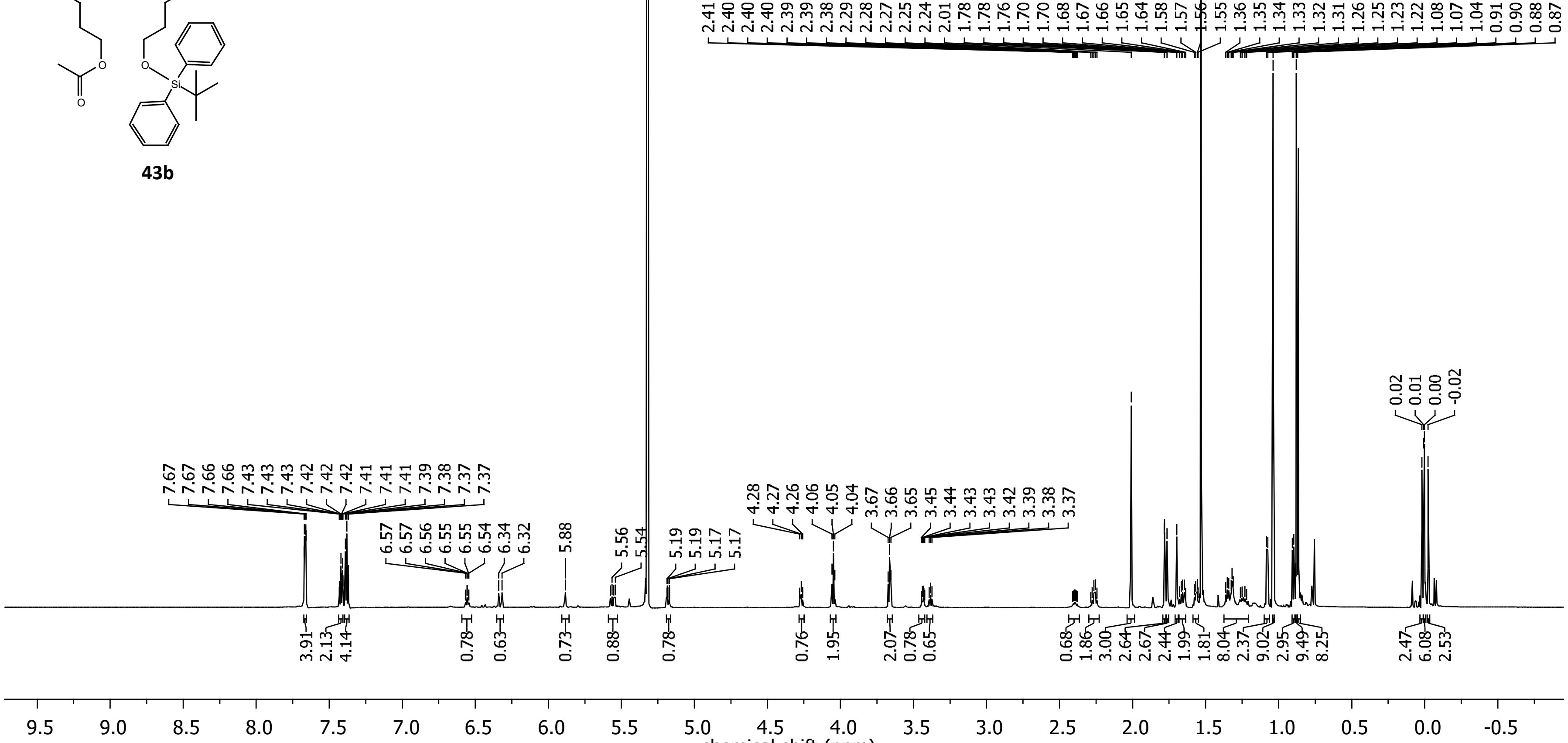

Nucleus: 13C
Frequency: 176.12 MHz
Solvent: CD2Cl2
Temperature: 298.0 K

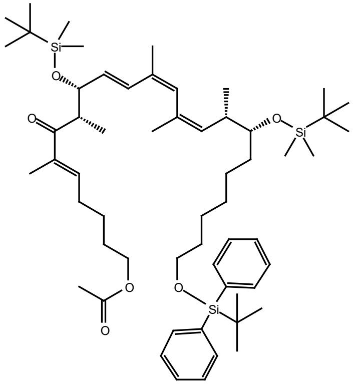

43b

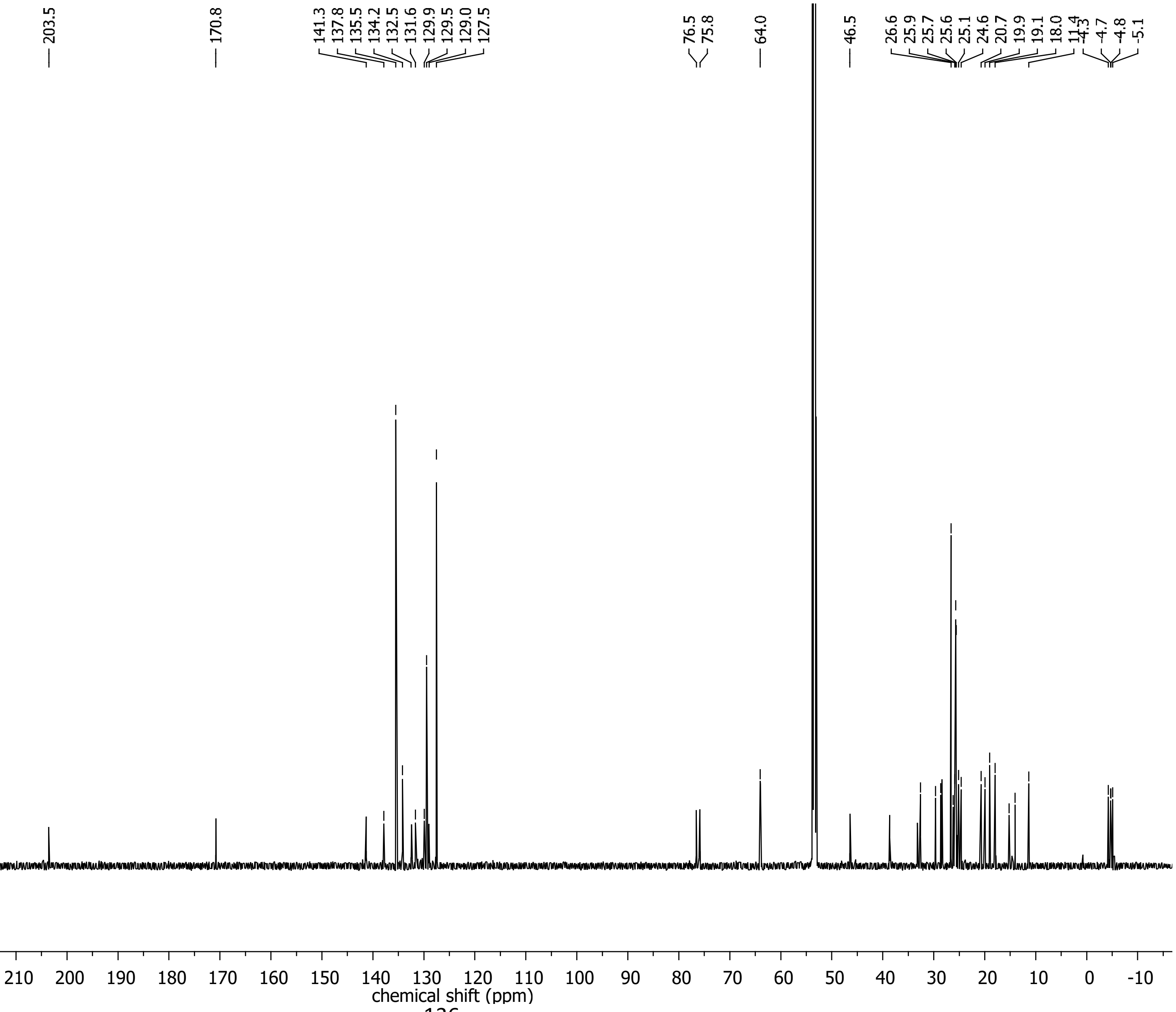

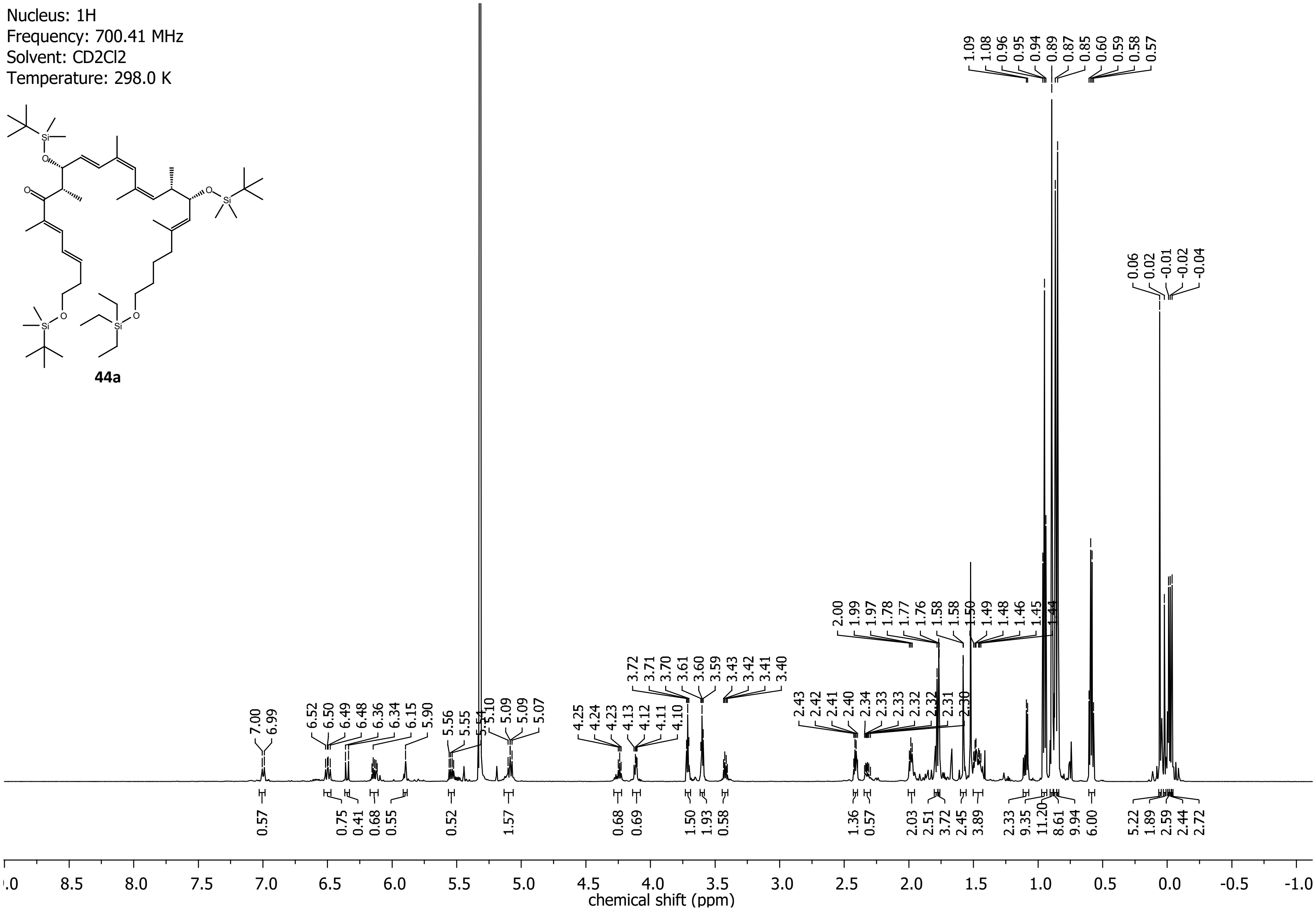
Nucleus: 1H
Frequency: 700.41 MHz
Solvent: CD2Cl2
Temperature: 298.0 K
44a
1.09 1.08 0.96 0.95 0.94 0.89 0.87 0.85 0.60 0.59 0.58 0.57
0.06 0.02 -0.01 -0.02 -0.04
2.00 1.99 1.97 1.78 1.77 1.76 1.58 1.58 1.50 1.49 1.48 1.46 1.45 1.44
3.72 3.71 3.70 3.61 3.60 3.59 3.43 3.42 3.41 3.40
2.43 2.42 2.41 2.40 2.34 2.33 2.33 2.32 2.32 2.31 2.30
7.00 6.99 6.52 6.50 6.49 6.48 6.36 6.34 6.15 5.90 5.56 5.55 5.54 5.10 5.09 5.09 5.07
4.25 4.24 4.23 4.13 4.12 4.11 4.10
0.57 0.75 0.41 0.68 0.55 0.52 1.57 0.68 0.69 1.50 1.93 0.58 1.36 0.57 2.03 2.51 3.72 2.45 3.89 2.33 9.35 11.20 8.61 9.94 6.00 5.22 1.89 2.59 2.44 2.72
.0 8.5 8.0 7.5 7.0 6.5 6.0 5.5 5.0 4.5 4.0 3.5 3.0 2.5 2.0 1.5 1.0 0.5 0.0 -0.5 -1.0
chemical shift (ppm)

Nucleus: 13C
Frequency: 176.12 MHz
Solvent: CD2Cl2
Temperature: 298.0 K

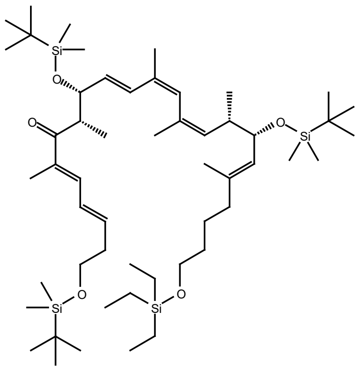

44a

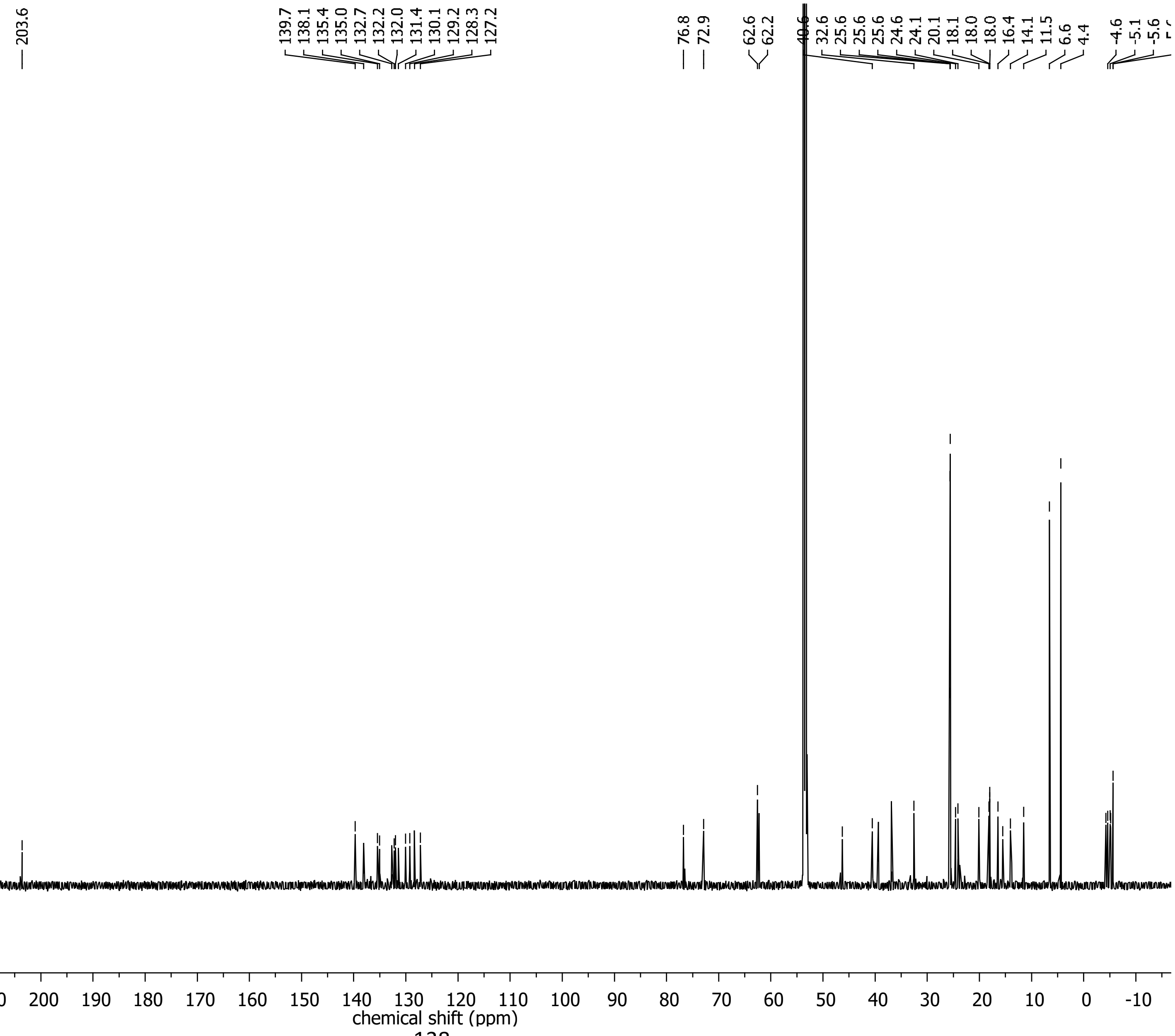

Nucleus: 1H
Frequency: 500.14 MHz
Solvent: CD2Cl2
Temperature: 298.0 K

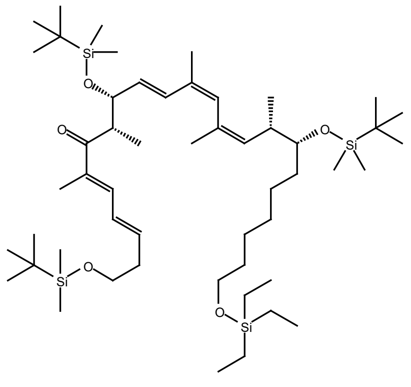

44b

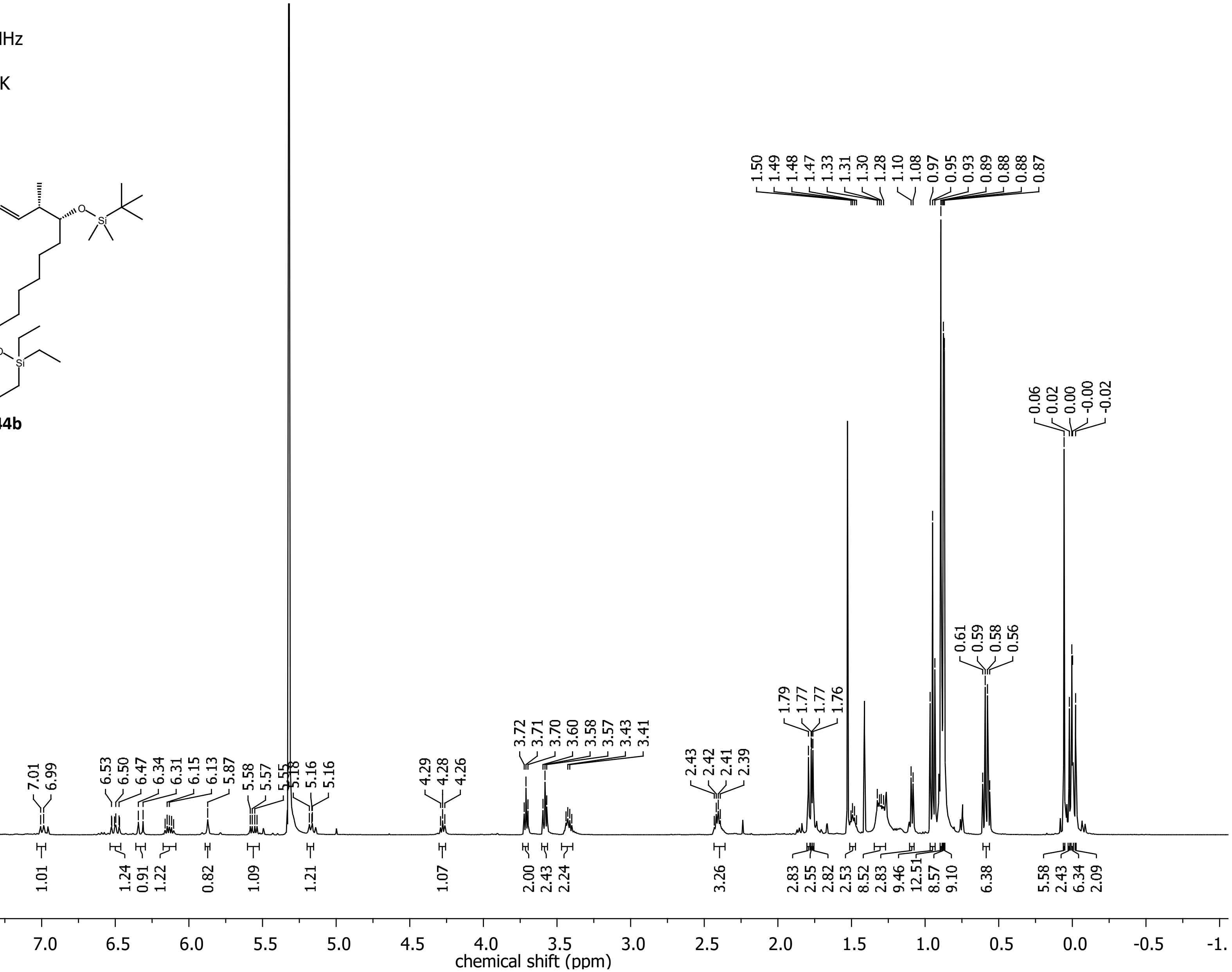

Nucleus: 13C
Frequency: 125.76 MHz
Solvent: CD2Cl2
Temperature: 298.0 K

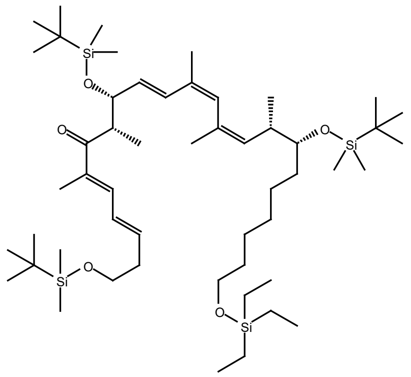

44b

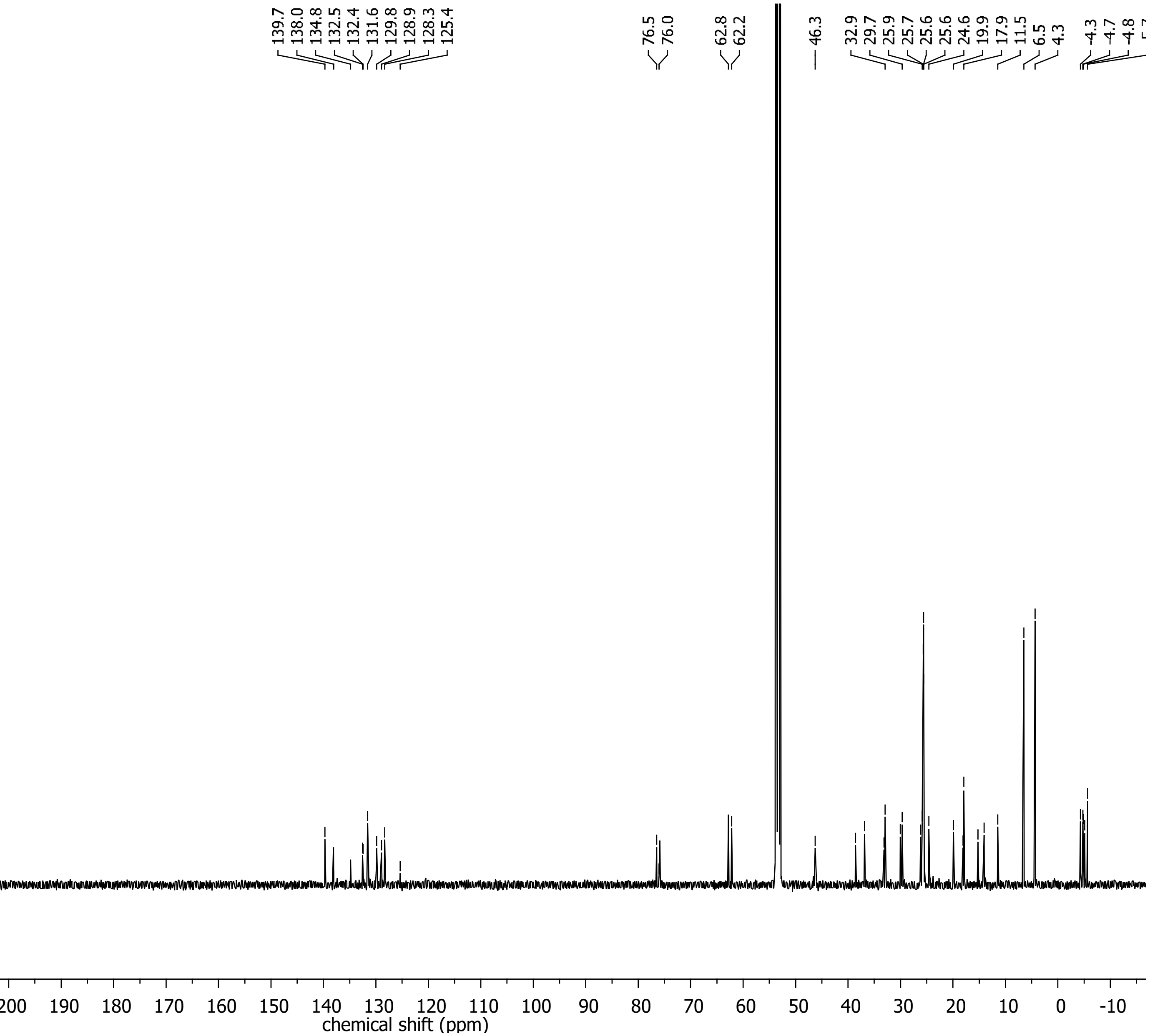

Nucleus: 1H
Frequency: 500.14 MHz
Solvent: CD2Cl2
Temperature: 298.0 K

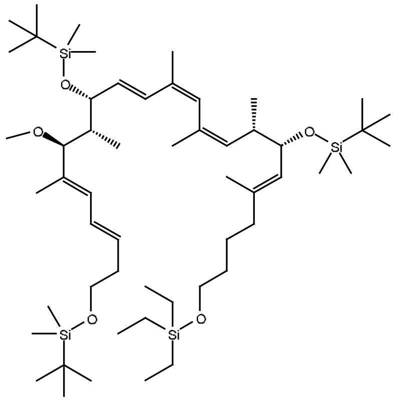

46a

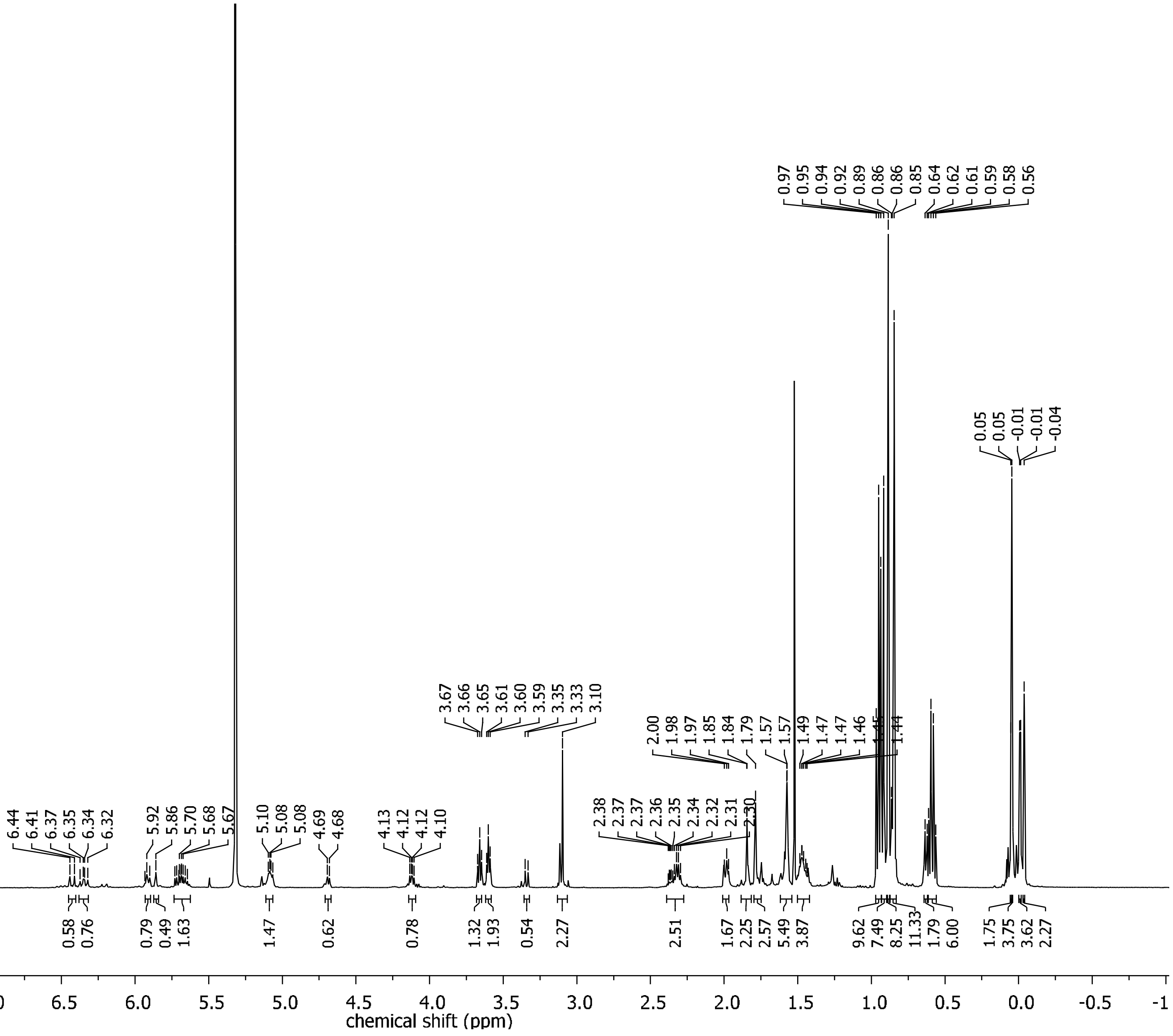

Nucleus: 13C
Frequency: 125.76 MHz
Solvent: CD2Cl2
Temperature: 298.0 K

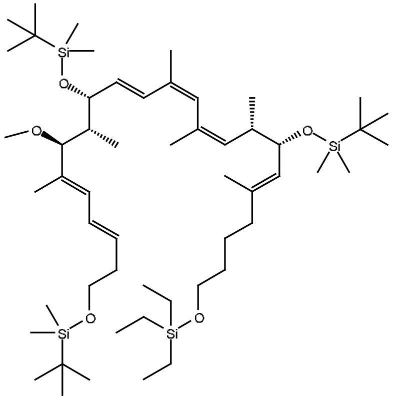

46a

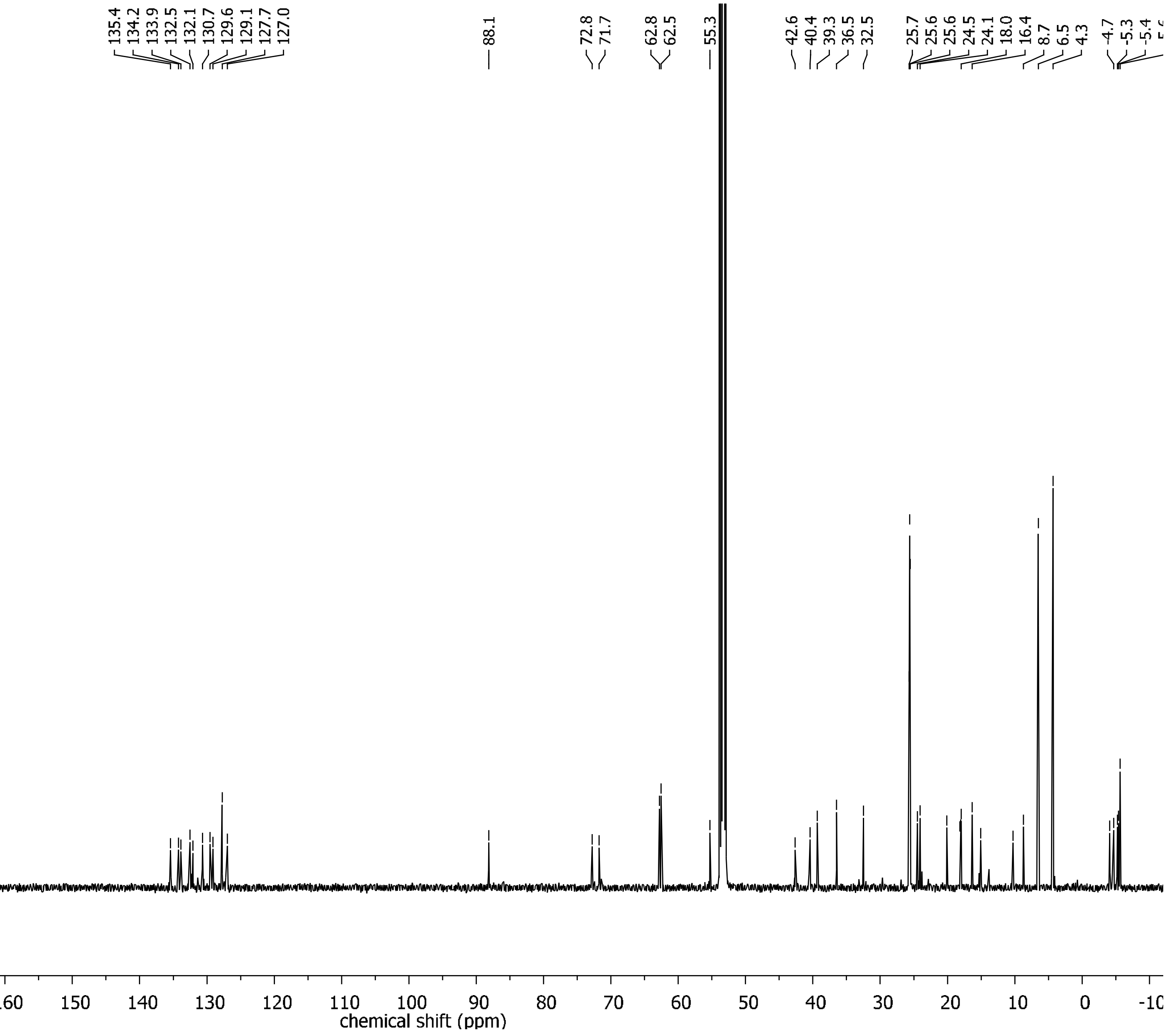

Nucleus: 1H
Frequency: 500.14 MHz
Solvent: CDCl3
Temperature: 298.0 K

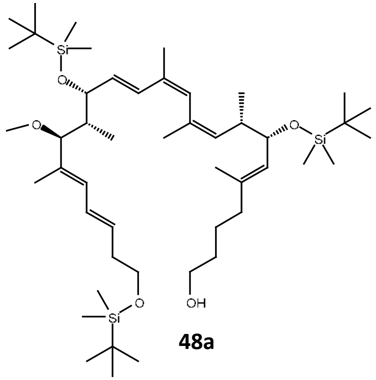

48a

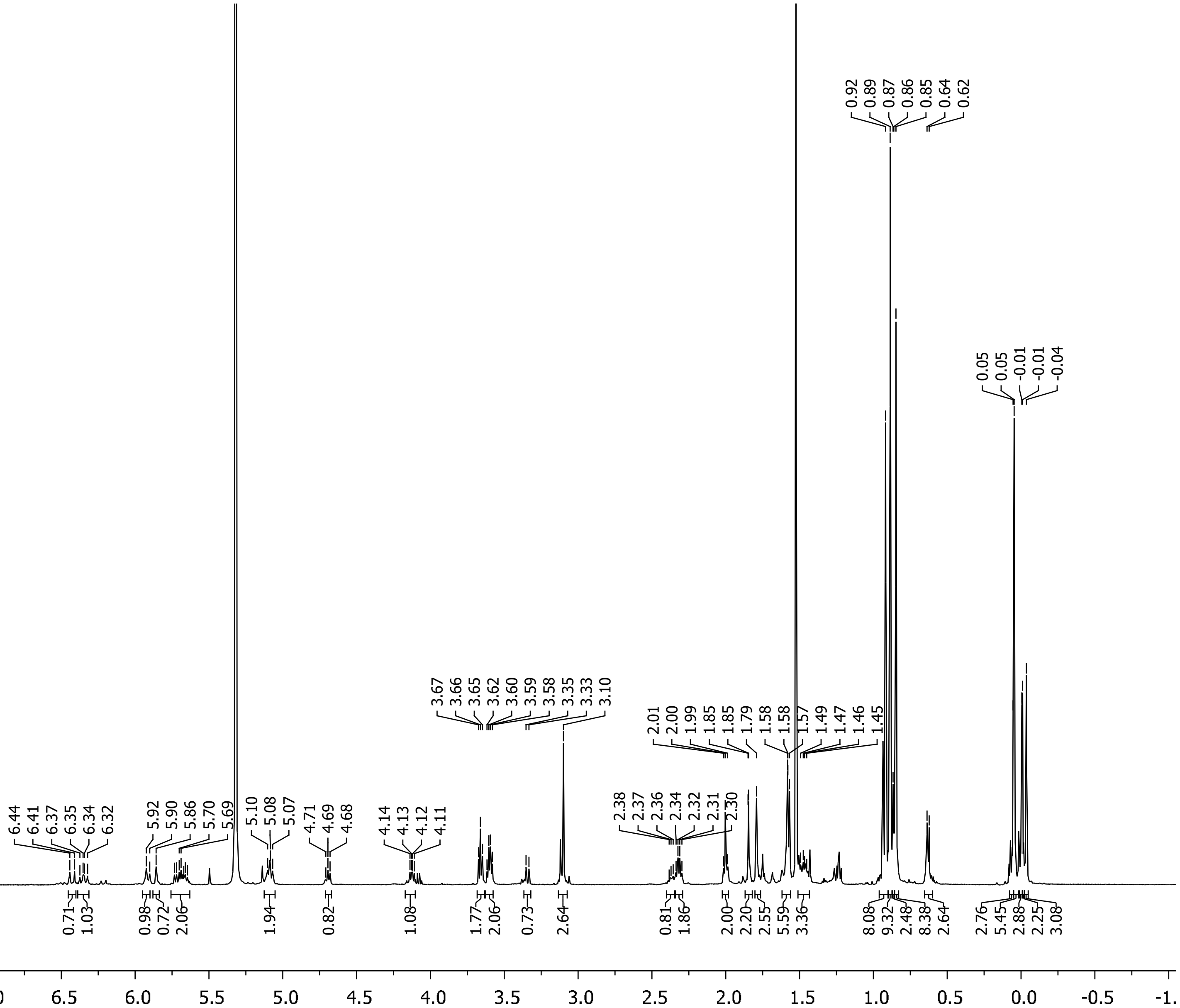

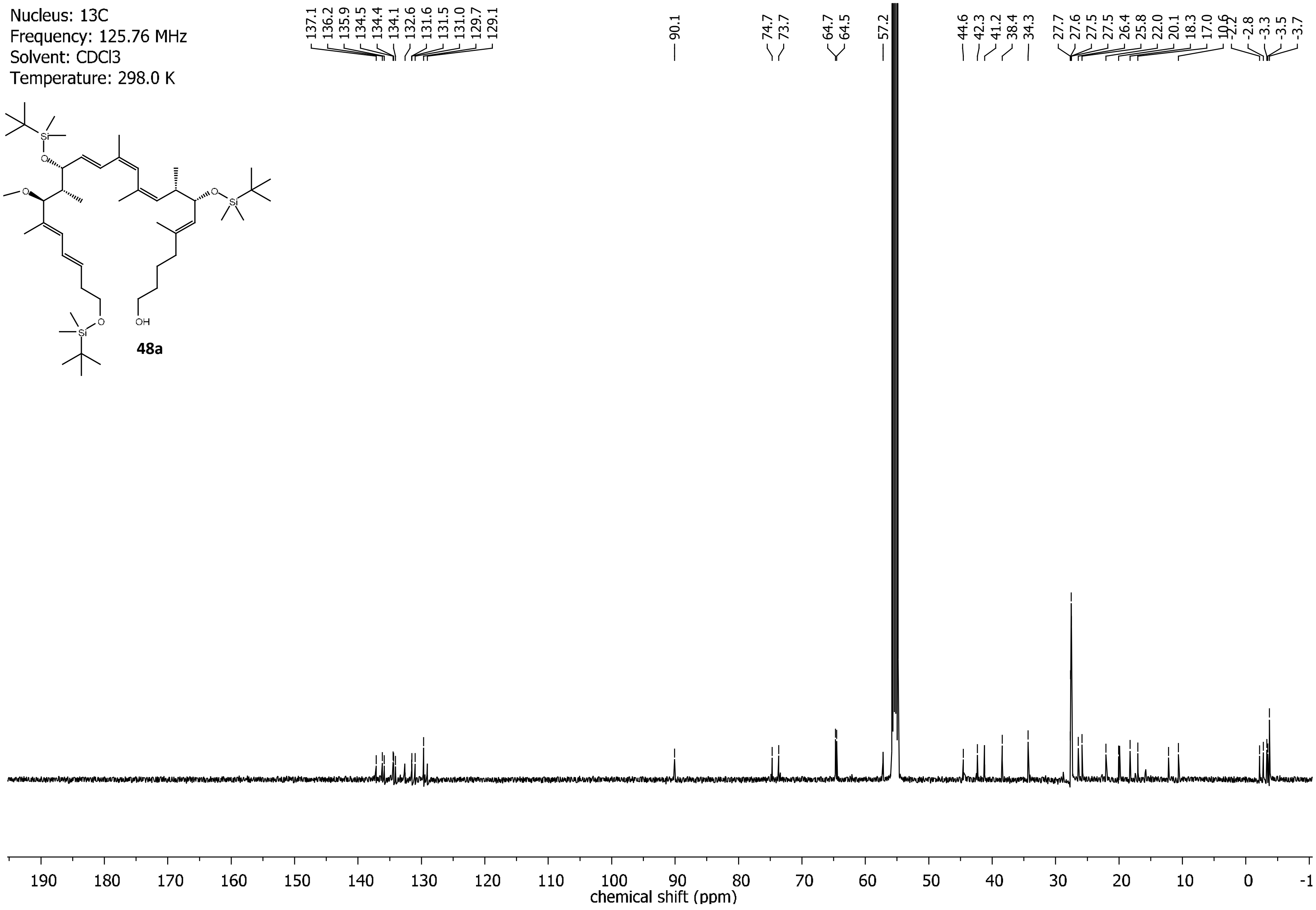
Nucleus: 13C
Frequency: 125.76 MHz
Solvent: CDCl3
Temperature: 298.0 K
137.1
136.2
135.9
134.5
134.4
134.1
132.6
131.6
131.5
131.0
129.7
129.1
90.1
74.7
73.7
64.7
64.5
57.2
44.6
42.3
41.2
38.4
34.3
27.7
27.6
27.5
27.5
26.4
25.8
22.0
20.1
18.3
17.0
10.6
-2.2
-2.8
-3.3
-3.5
-3.7
Si
O
O
O
Si
OH
O
Si
190 180 170 160 150 140 130 120 110 100 90 80 70 60 50 40 30 20 10 0 -1
chemical shift (ppm)

48a

Nucleus: 1H
Frequency: 700.41 MHz
Solvent: CD2Cl2
Temperature: 298.0 K

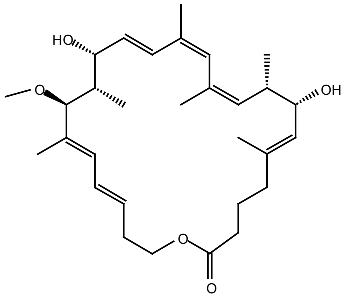

5

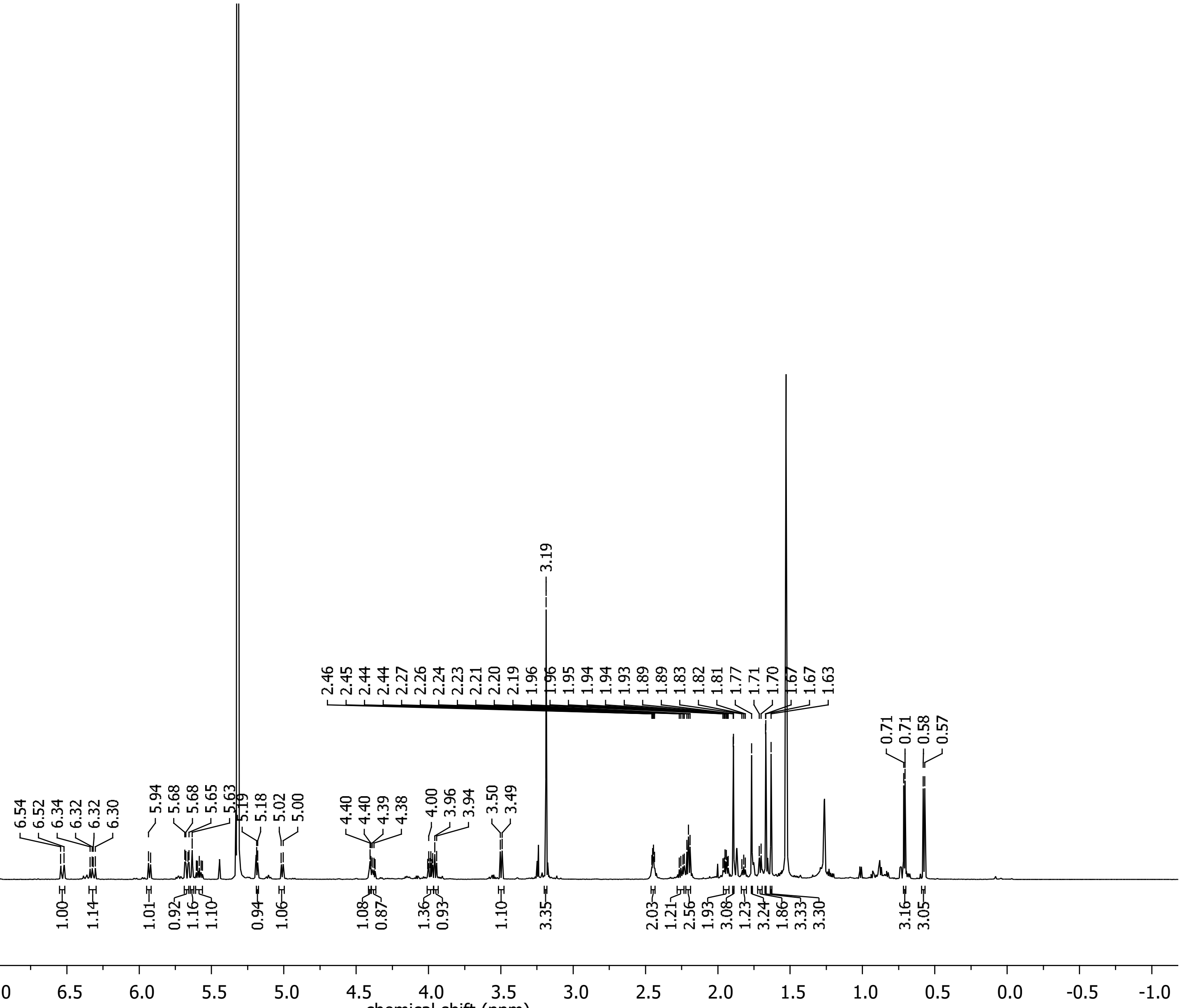

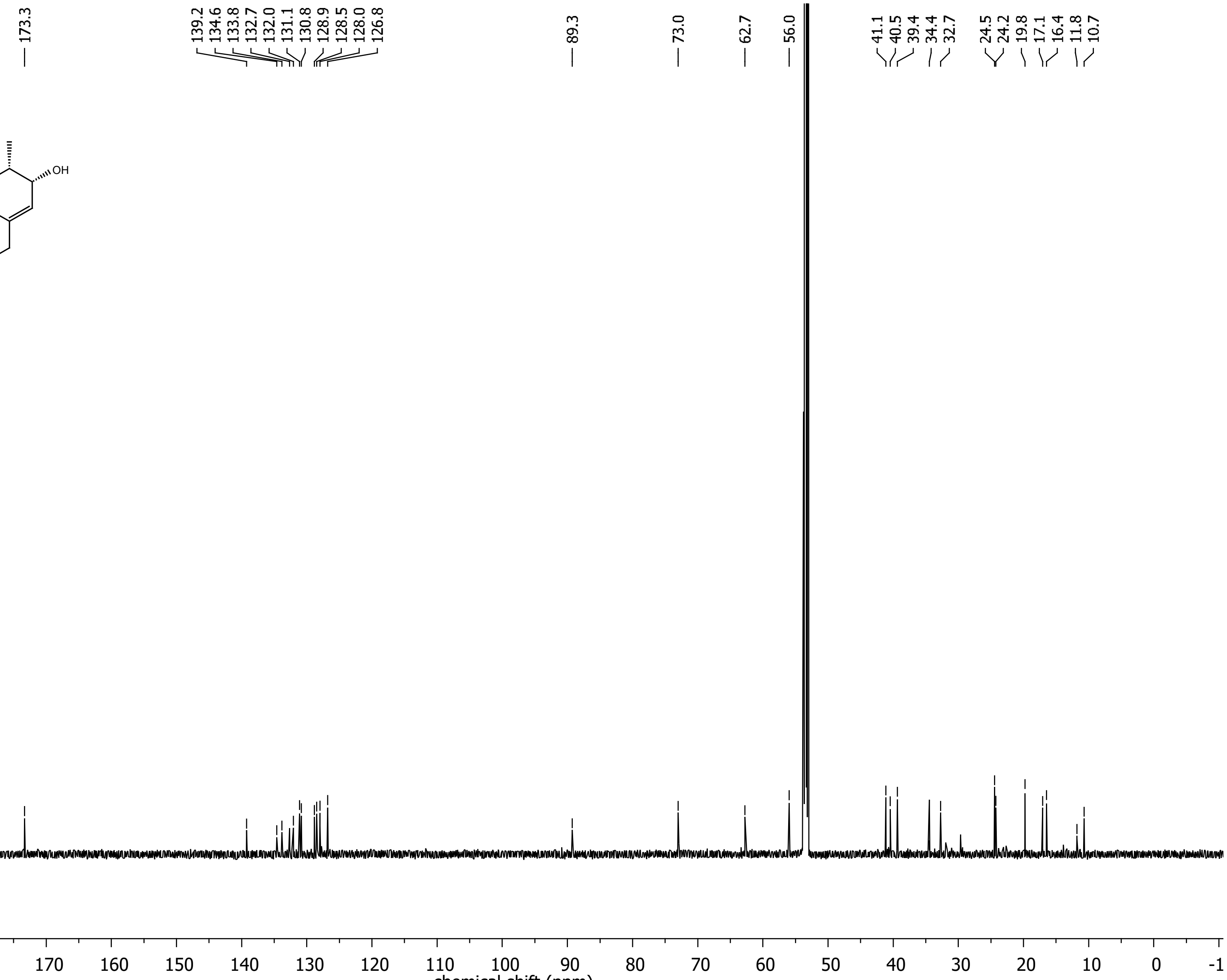
Nucleus: 13C
Frequency: 176.12 MHz
Solvent: CD2Cl2
Temperature: 298.0 K
173.3
139.2
134.6
133.8
132.7
132.0
131.1
130.8
128.9
128.5
128.0
126.8
89.3
73.0
62.7
56.0
41.1
40.5
39.4
34.4
32.7
24.5
24.2
19.8
17.1
16.4
11.8
10.7
200
190
180
170
160
150
140
130
120
110
100
90
80
70
60
50
40
30
20
10
0
-1
chemical shift (ppm)

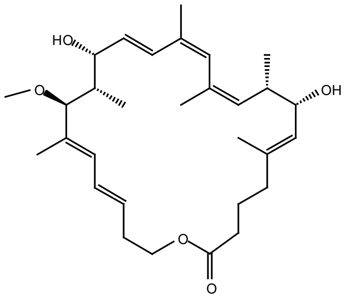
HO
O
OH
O
O

5

Nucleus: 1H
Frequency: 700.41 MHz
Solvent: CD2Cl2
Temperature: 298.0 K

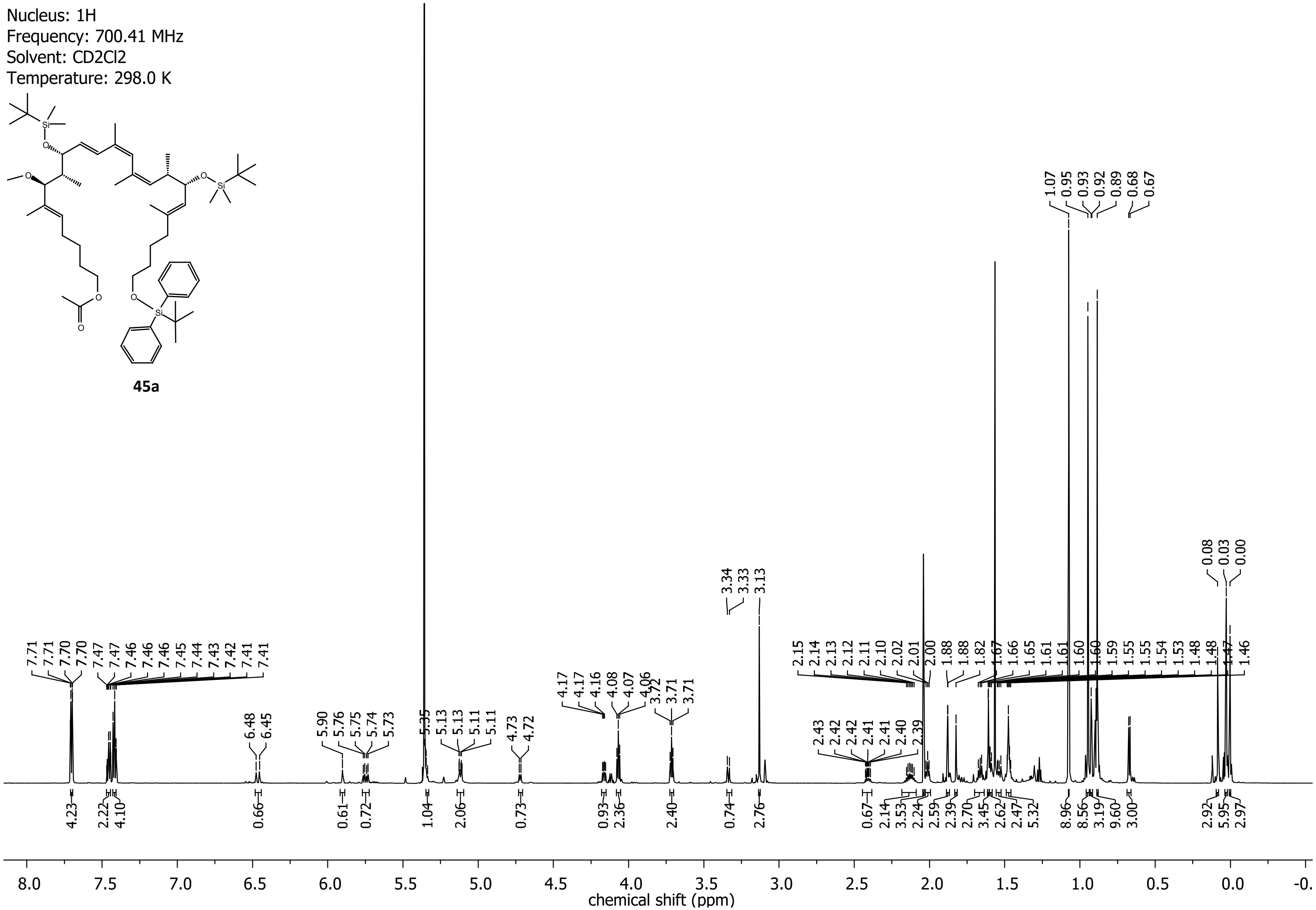

45a

Nucleus: 13C
Frequency: 176.12 MHz
Solvent: CD2Cl2
Temperature: 298.0 K

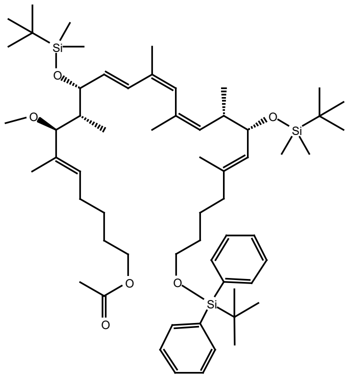

45a

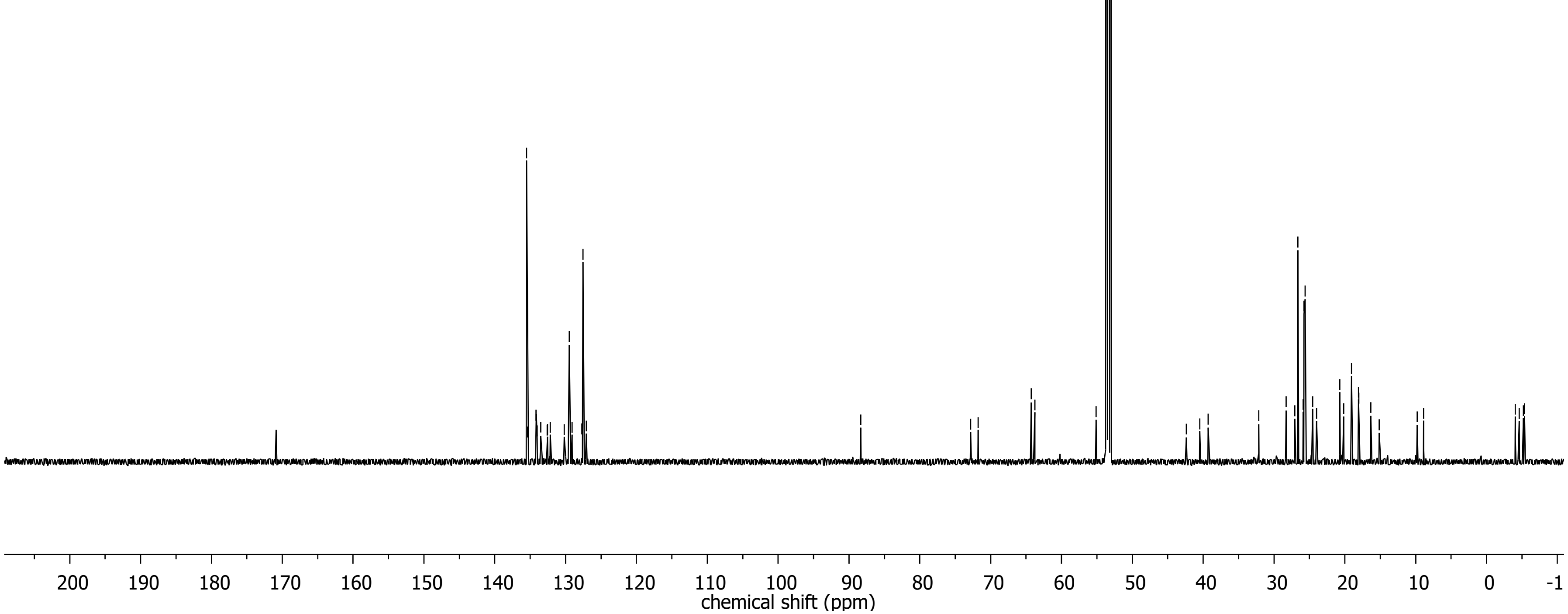

Nucleus: 1H
Frequency: 700.41 MHz
Solvent: CD2Cl2
Temperature: 298.0 K

47a

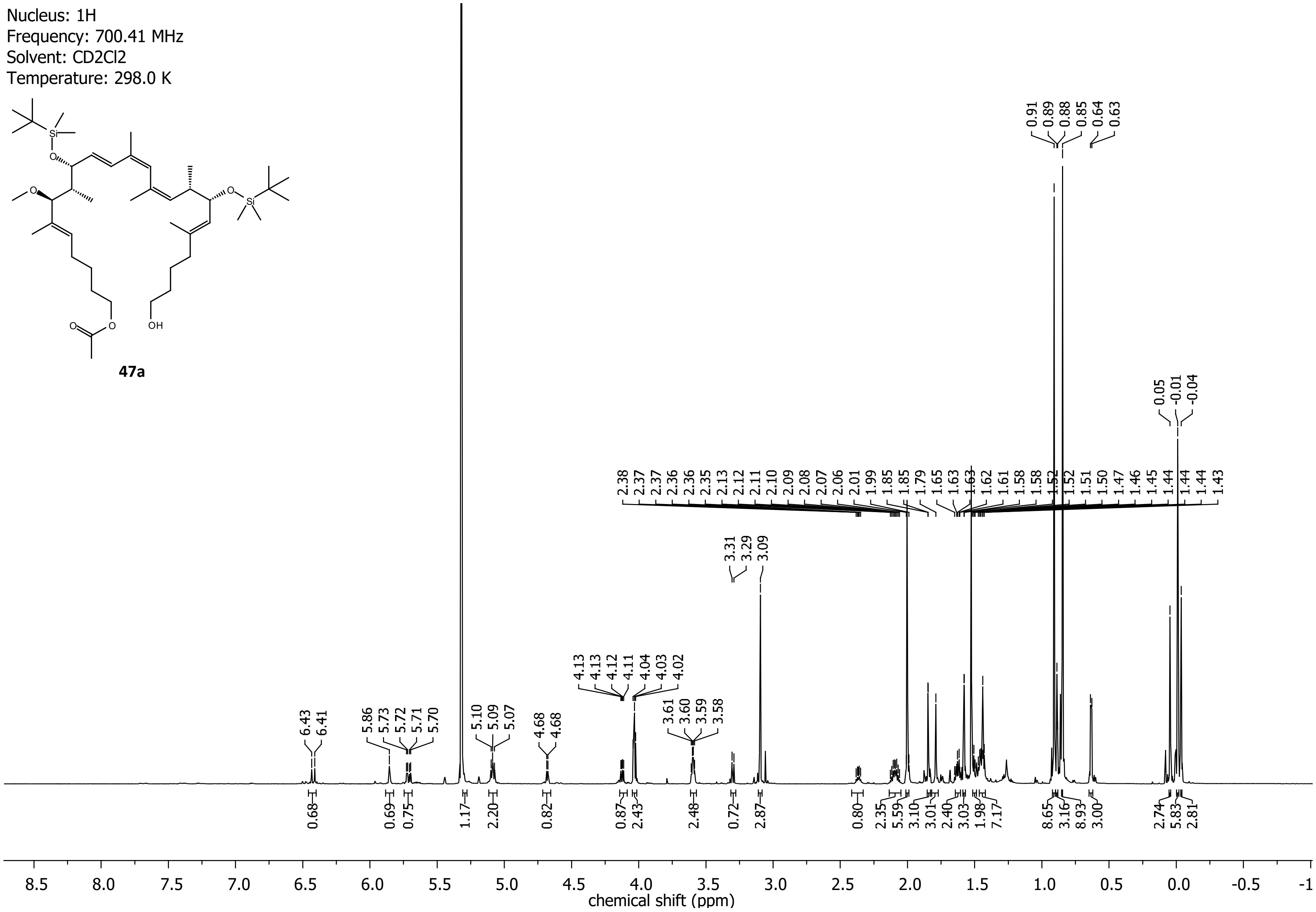

Nucleus: 13C
Frequency: 176.12 MHz
Solvent: CD2Cl2
Temperature: 298.0 K

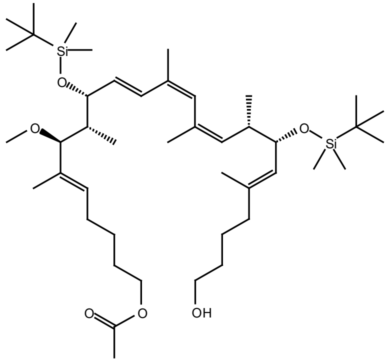

47a

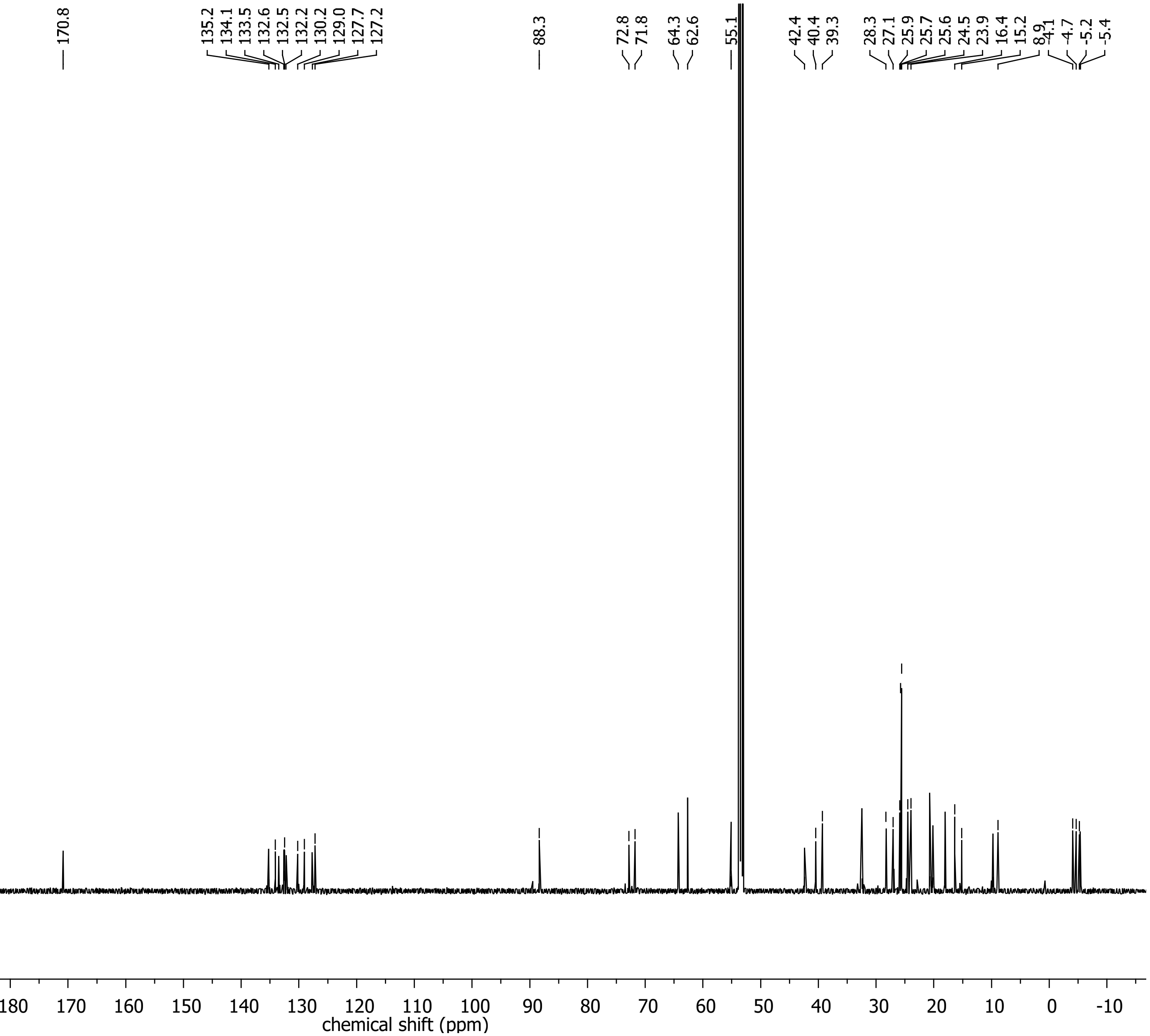

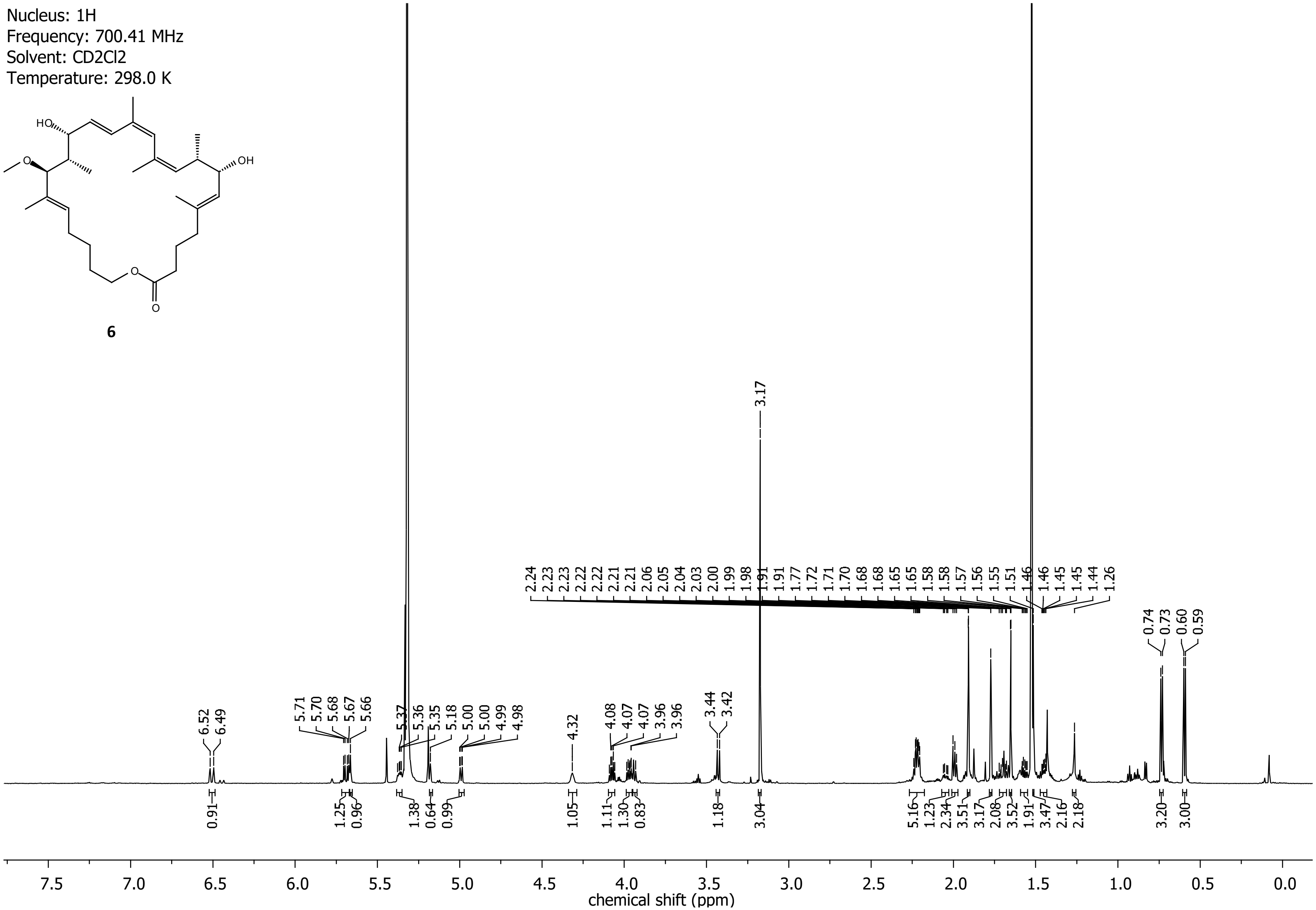
Nucleus: 1H
Frequency: 700.41 MHz
Solvent: CD2Cl2
Temperature: 298.0 K
HO
OH
O
O
O
6
6.52
6.49
5.71
5.70
5.68
5.67
5.66
5.37
5.36
5.35
5.18
5.00
5.00
4.99
4.98
4.32
4.08
4.07
4.07
3.96
3.96
3.44
3.42
3.17
2.24
2.23
2.23
2.22
2.22
2.21
2.21
2.06
2.05
2.04
2.03
2.00
1.99
1.98
1.91
1.91
1.77
1.72
1.71
1.70
1.68
1.68
1.65
1.65
1.58
1.58
1.57
1.56
1.55
1.51
1.46
1.46
1.45
1.45
1.44
1.26
0.74
0.73
0.60
0.59
0.91
1.25
0.96
1.38
0.64
0.99
1.05
1.11
1.30
0.83
1.18
3.04
5.16
1.23
2.34
3.51
3.17
2.08
3.52
1.91
3.47
2.16
2.18
3.20
3.00
7.5
7.0
6.5
6.0
5.5
5.0
4.5
4.0
3.5
3.0
2.5
2.0
1.5
1.0
0.5
0.0
chemical shift (ppm)

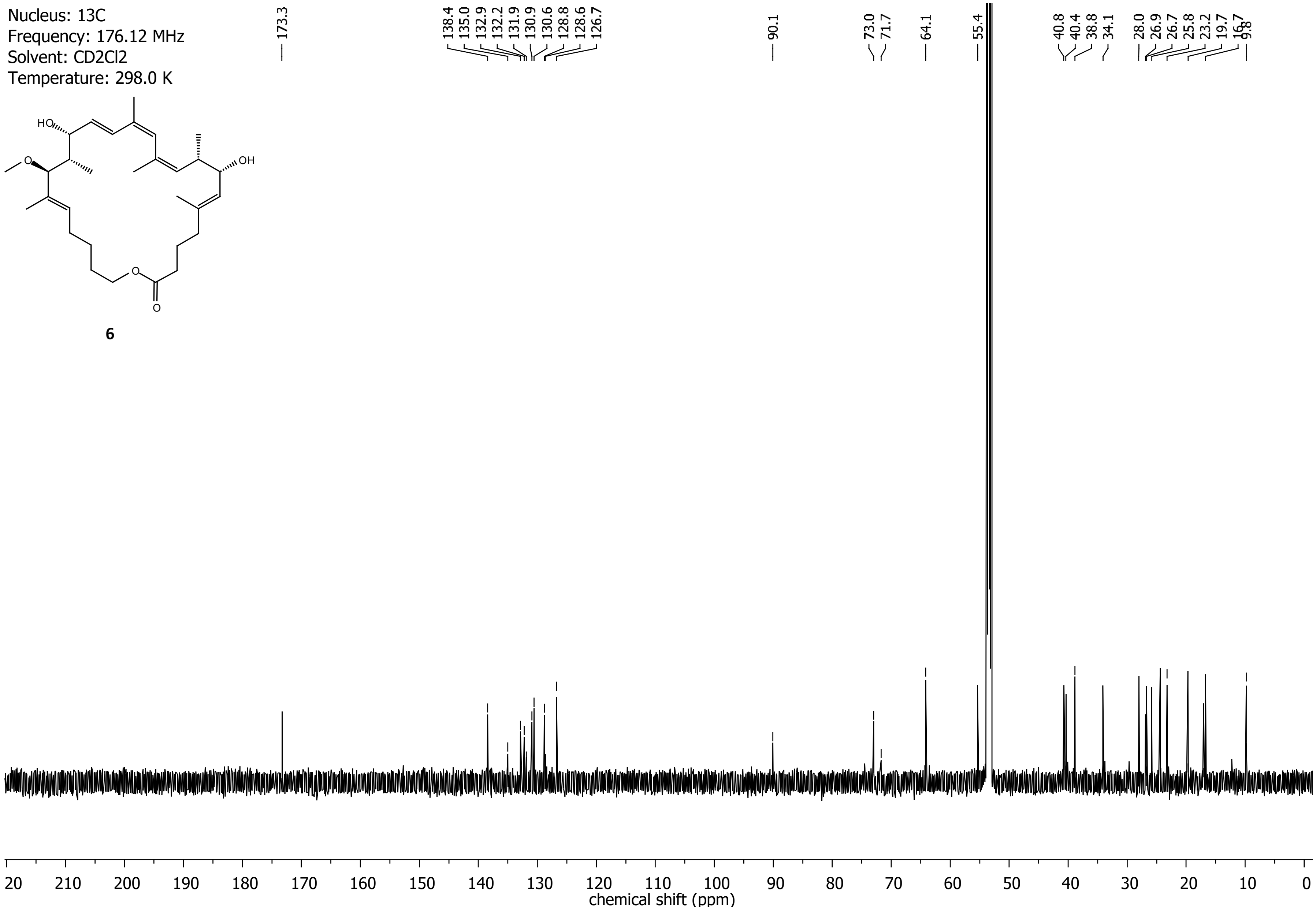
Nucleus: 13C
Frequency: 176.12 MHz
Solvent: CD2Cl2
Temperature: 298.0 K
HO
O
OH
O
O
6
173.3
138.4
135.0
132.9
132.2
131.9
130.9
130.6
128.8
128.6
126.7
90.1
73.0
71.7
64.1
55.4
40.8
40.4
38.8
34.1
28.0
26.9
26.7
25.8
23.2
19.7
16.7
9.8
20
210
200
190
180
170
160
150
140
130
120
110
100
90
80
70
60
50
40
30
20
10
0
chemical shift (ppm)

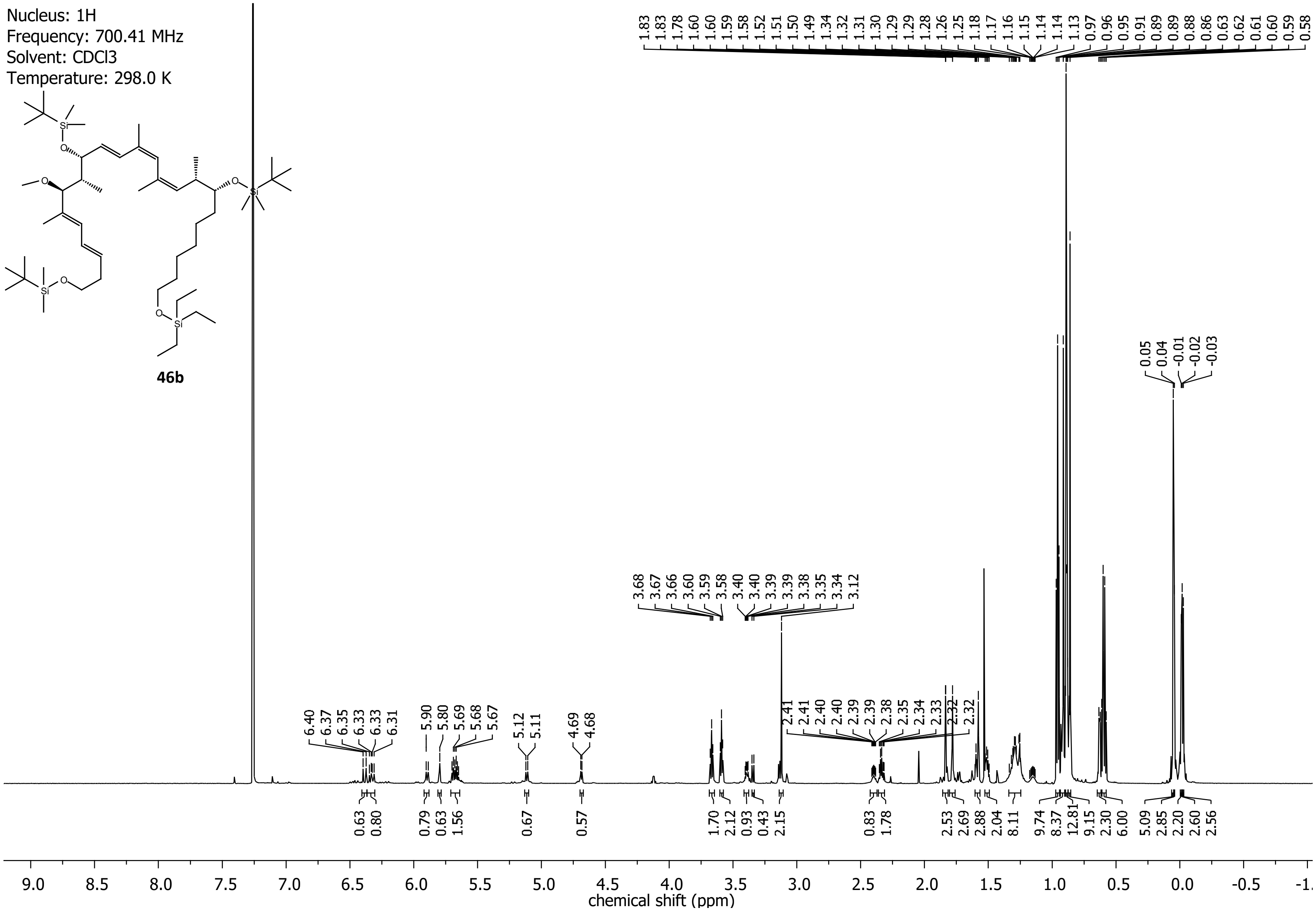
Nucleus: 1H
Frequency: 700.41 MHz
Solvent: CDCl3
Temperature: 298.0 K
46b
chemical shift (ppm)

Nucleus: 13C
Frequency: 176.12 MHz
Solvent: CDCl3
Temperature: 298.0 K

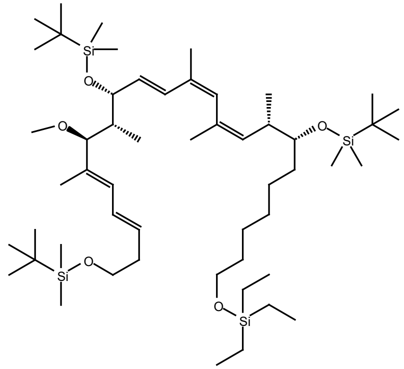

46b

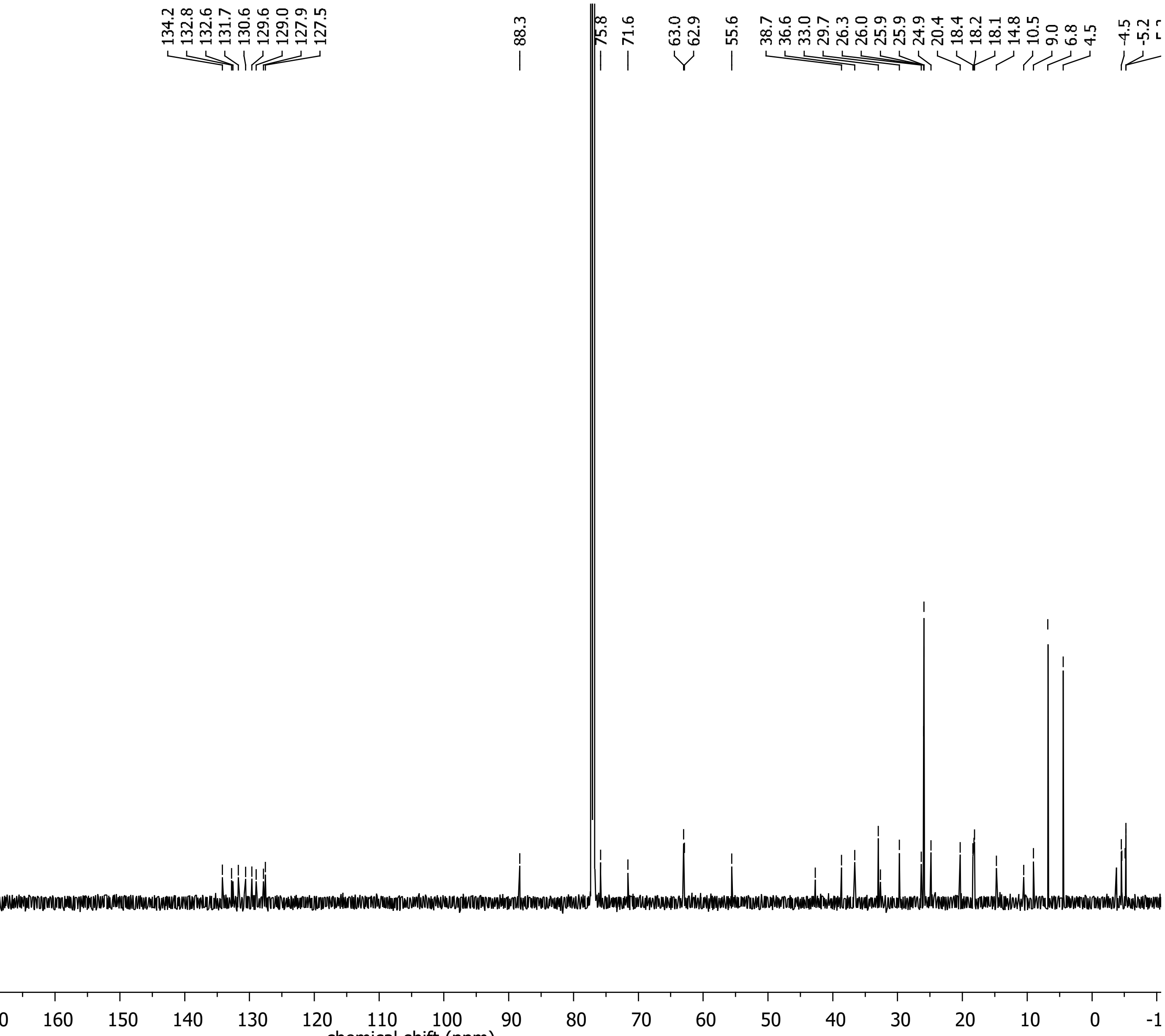

Nucleus: 1H
Frequency: 700.41 MHz
Solvent: CD2Cl2
Temperature: 298.0 K

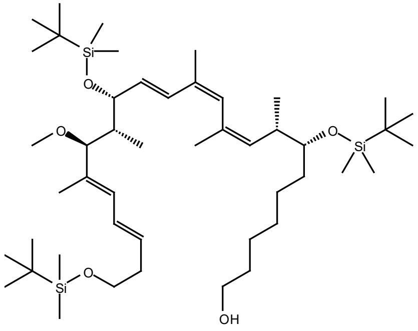

48b

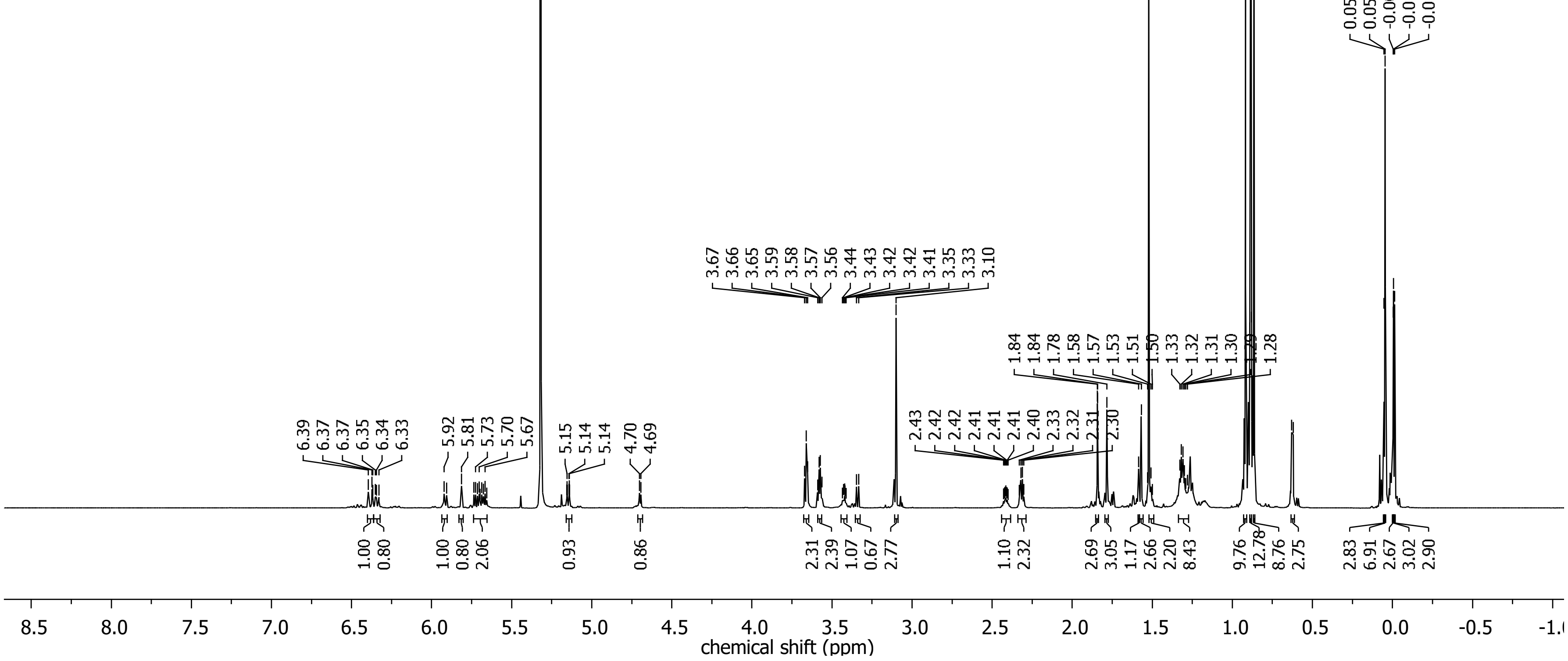

Nucleus: 13C
Frequency: 176.12 MHz
Solvent: CD2Cl2
Temperature: 298.0 K

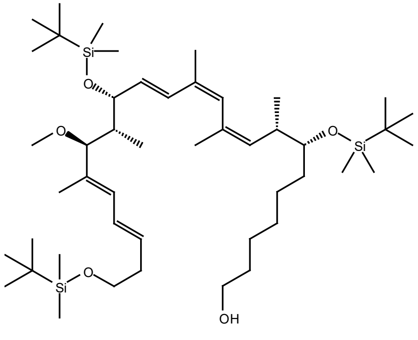

48b

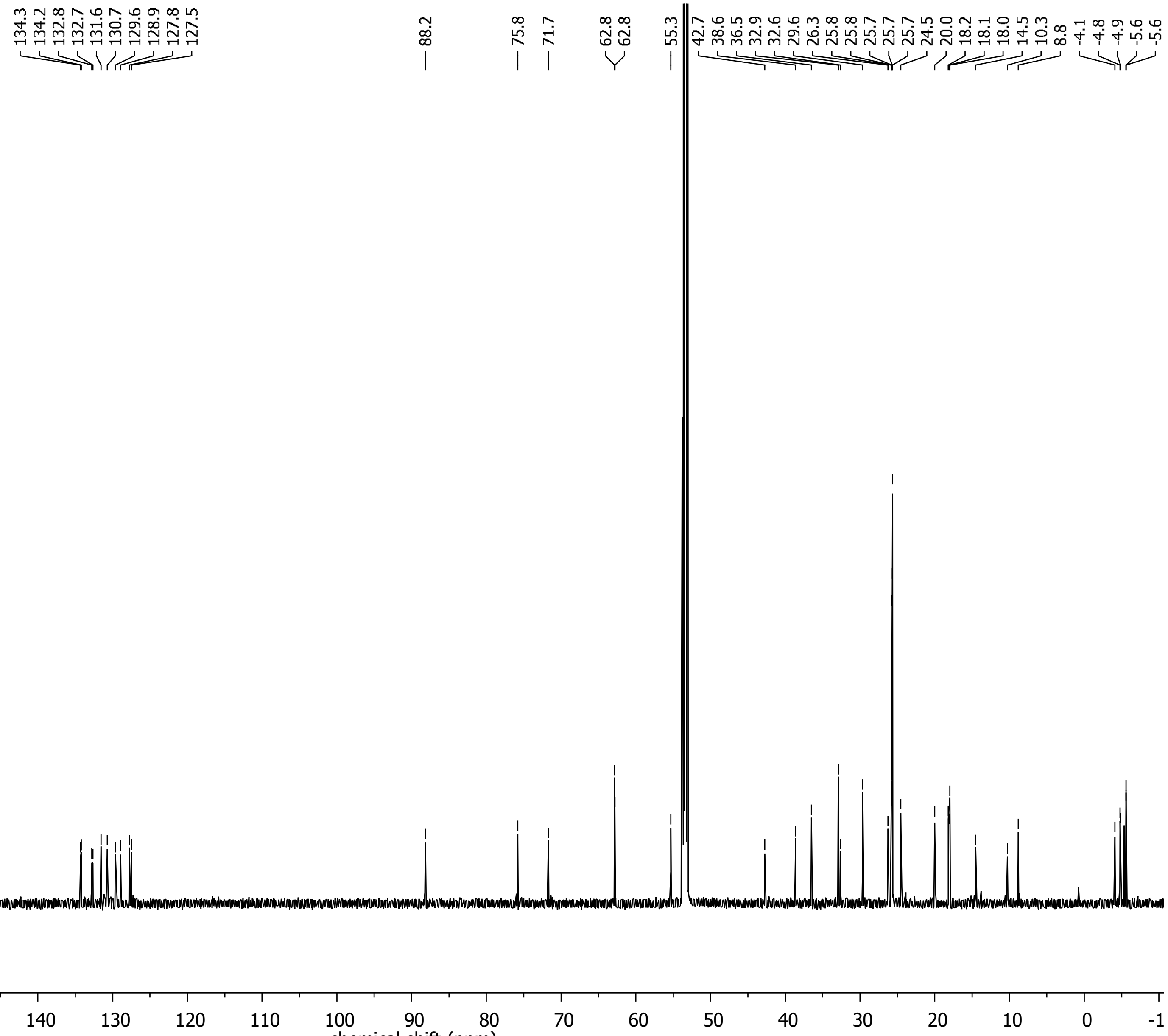

Nucleus: 1H
Frequency: 700.41 MHz
Solvent: CD2Cl2
Temperature: 298.0 K

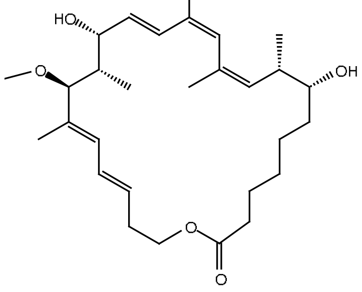

7

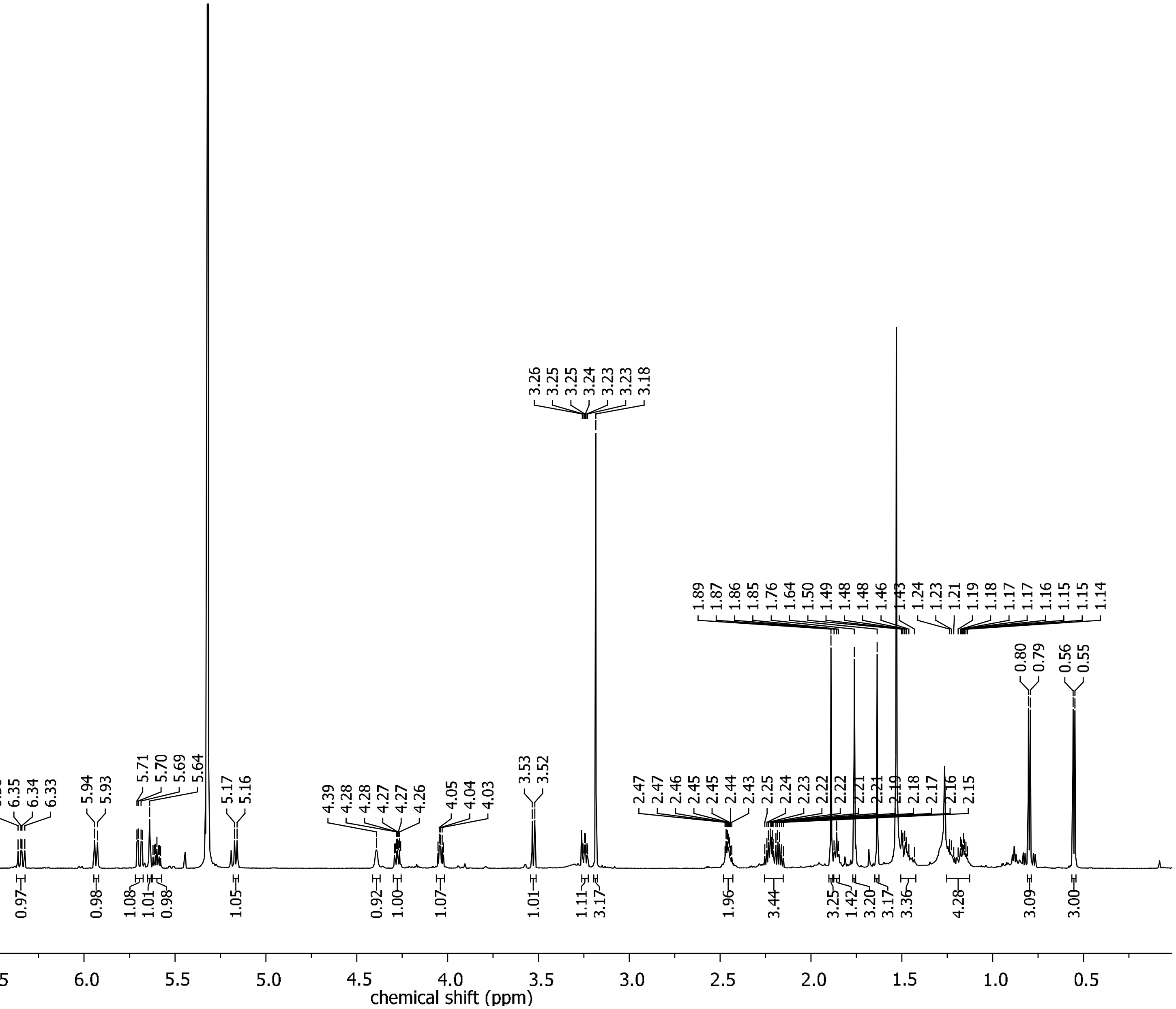

Nucleus: 13C
Frequency: 176.12 MHz
Solvent: CD2Cl2
Temperature: 298.0 K

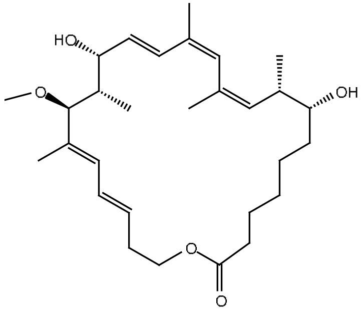

7

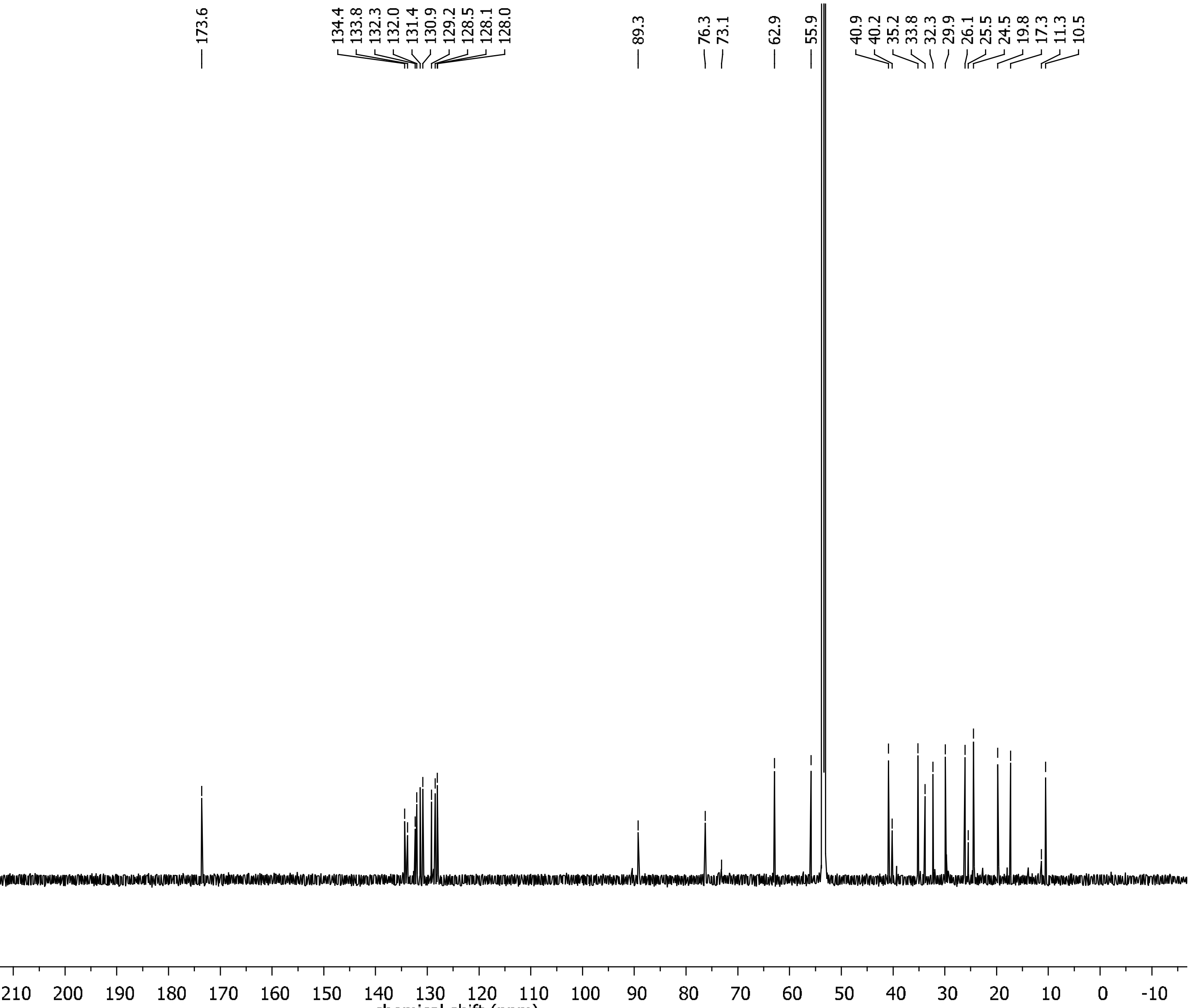

Nucleus: 1H
Frequency: 500.14 MHz
Solvent: CD2Cl2
Temperature: 298.0 K

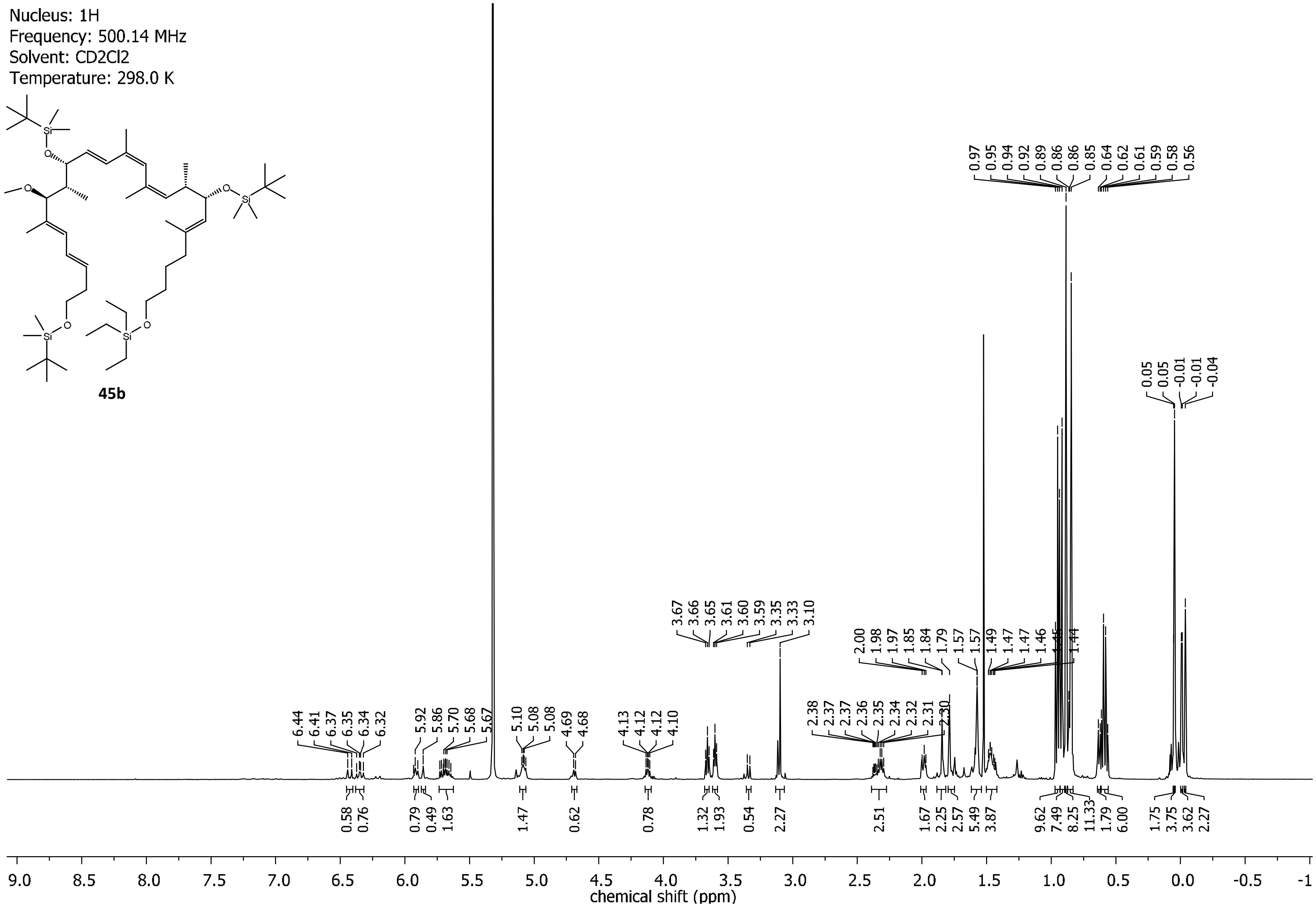

45b

Nucleus: 13C
Frequency: 125.76 MHz
Solvent: CDCl3
Temperature: 298.0 K

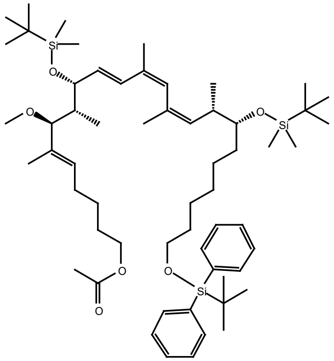

45b

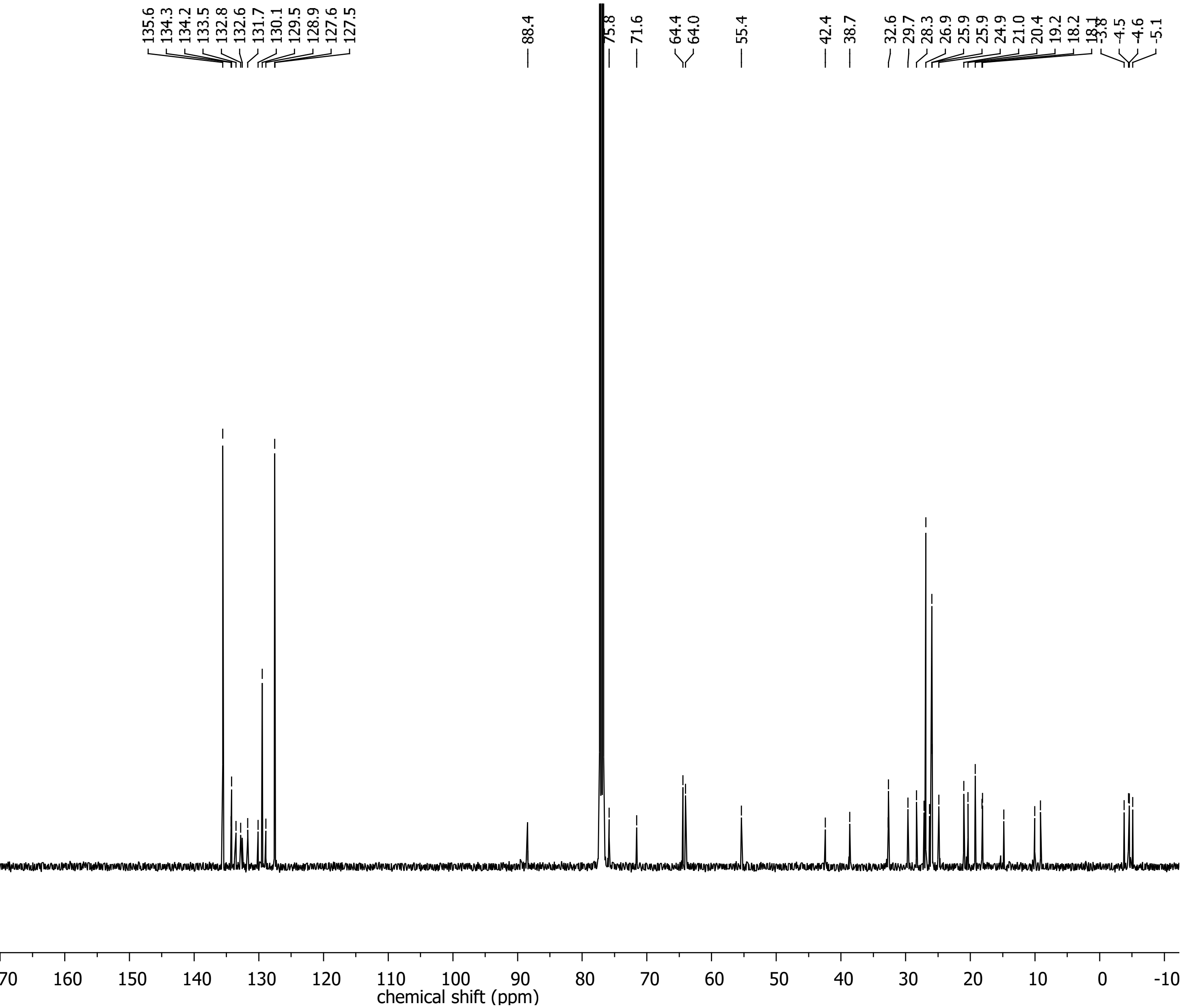

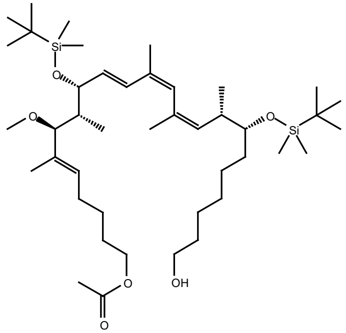

47b

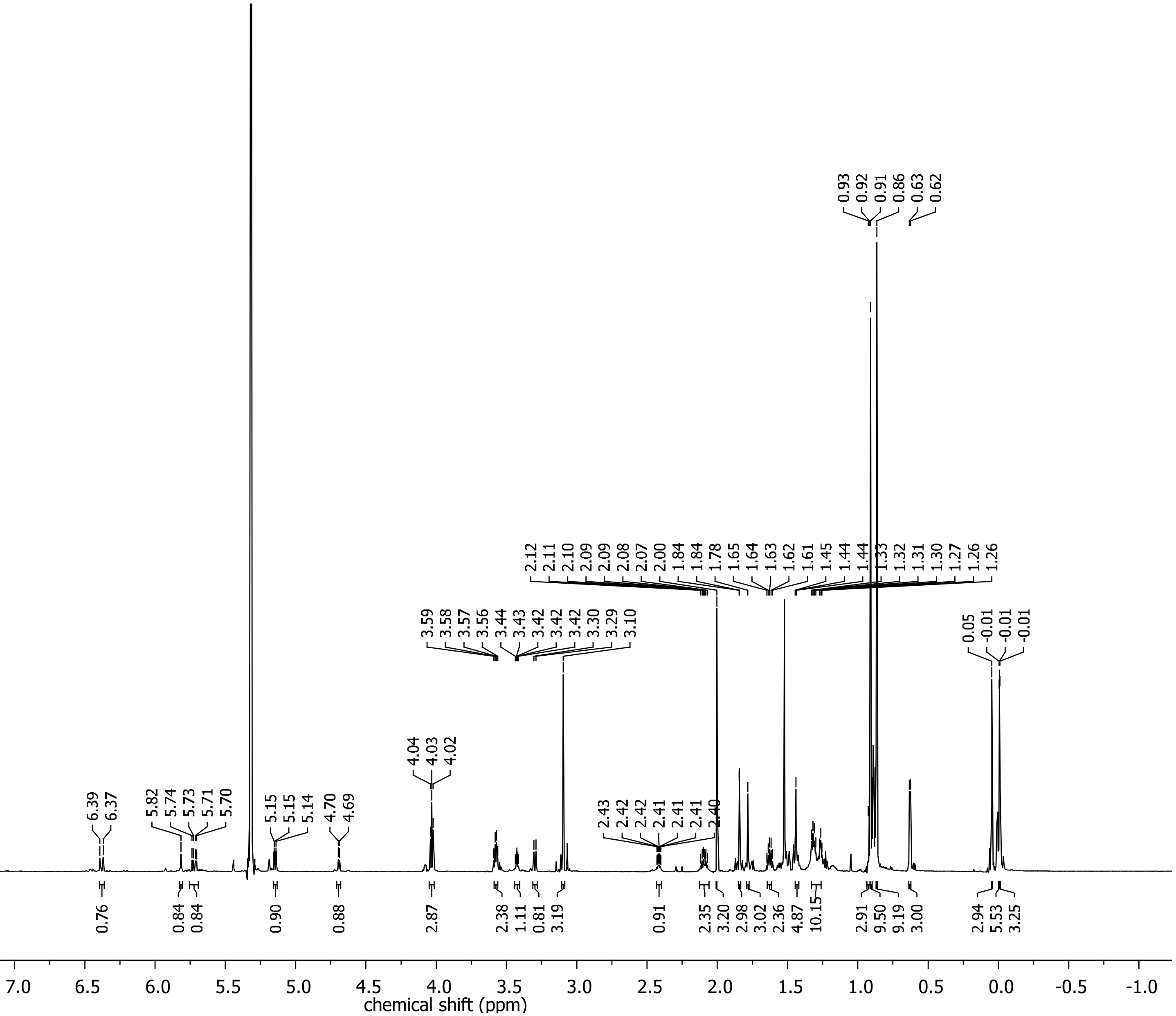

Nucleus: 1H
Frequency: 700.41 MHz
Solvent: CD2Cl2
Temperature: 298.0 K
6.39 6.37
5.82 5.74 5.73 5.71 5.70
5.15 5.15 5.14
4.70 4.69
4.04 4.03 4.02
3.59 3.58 3.57 3.56 3.44 3.43 3.42 3.42 3.42 3.30 3.29 3.10
2.43 2.42 2.42 2.41 2.41 2.41 2.40
2.12 2.11 2.10 2.09 2.09 2.08 2.07 2.00 1.84 1.84 1.78 1.65 1.64 1.63 1.62 1.61 1.45 1.44 1.44 1.33 1.32 1.31 1.30 1.27 1.26 1.26
0.93 0.92 0.91 0.86 0.63 0.62
0.05 -0.01 -0.01 -0.01
0.76 0.84 0.84 0.90 0.88 2.87 2.38 1.11 0.81 3.19 0.91 2.35 3.20 2.98 3.02 2.36 4.87 10.15 2.91 9.50 9.19 3.00 2.94 5.53 3.25
9.0 8.5 8.0 7.5 7.0 6.5 6.0 5.5 5.0 4.5 4.0 3.5 3.0 2.5 2.0 1.5 1.0 0.5 0.0 -0.5 -1.0
chemical shift (ppm)

Nucleus: 13C
Frequency: 176.12 MHz
Solvent: CD2Cl2
Temperature: 298.0 K

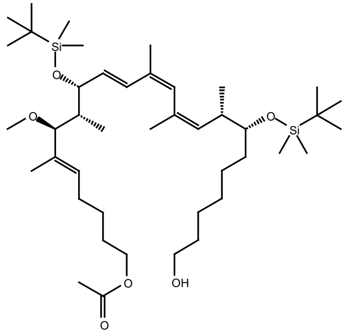

47b

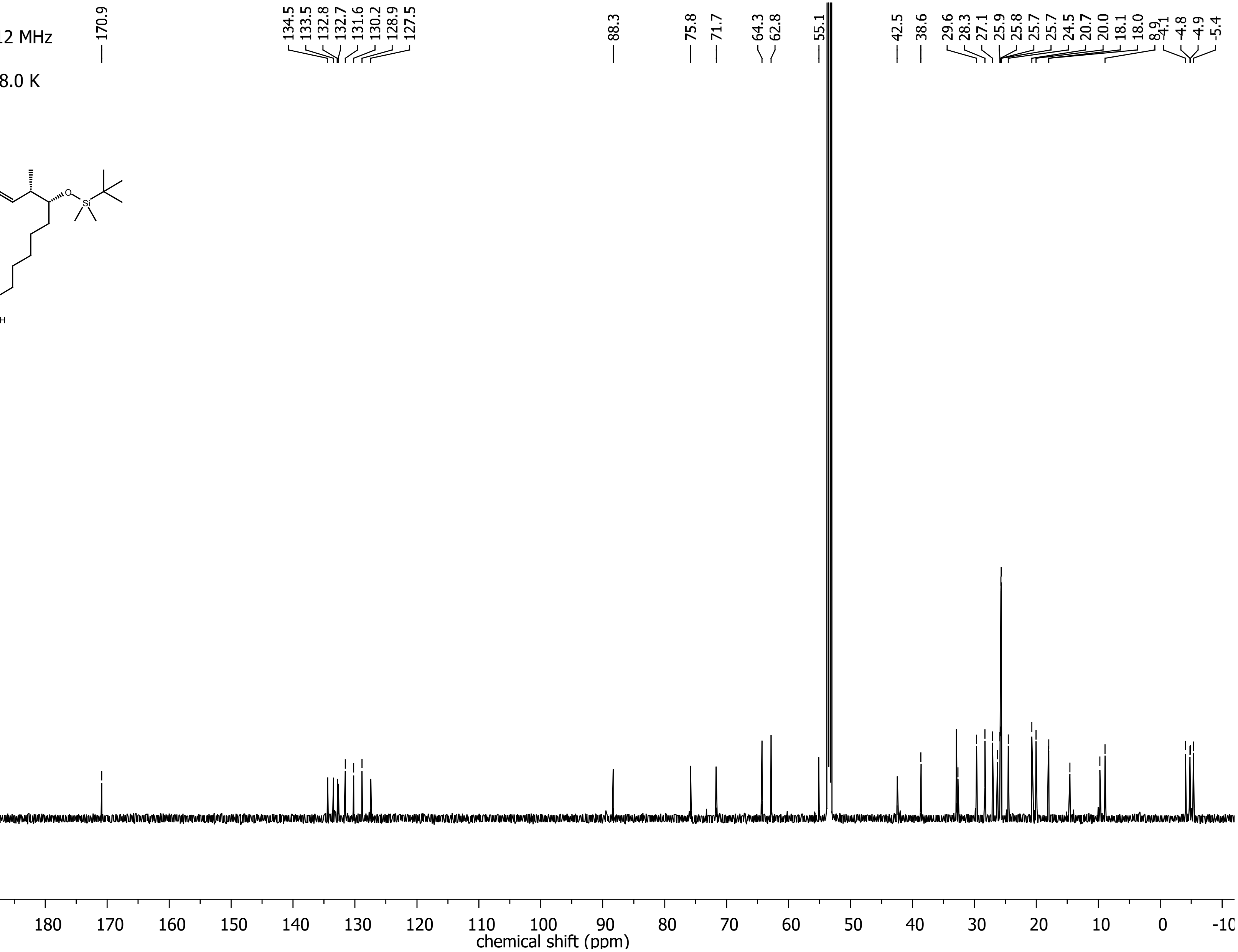

Nucleus: 1H
Frequency: 700.41 MHz
Solvent: CD2Cl2
Temperature: 298.0 K

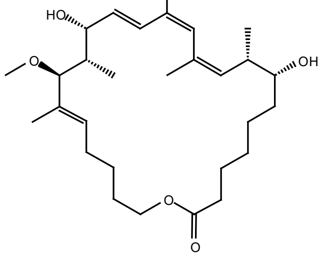

8

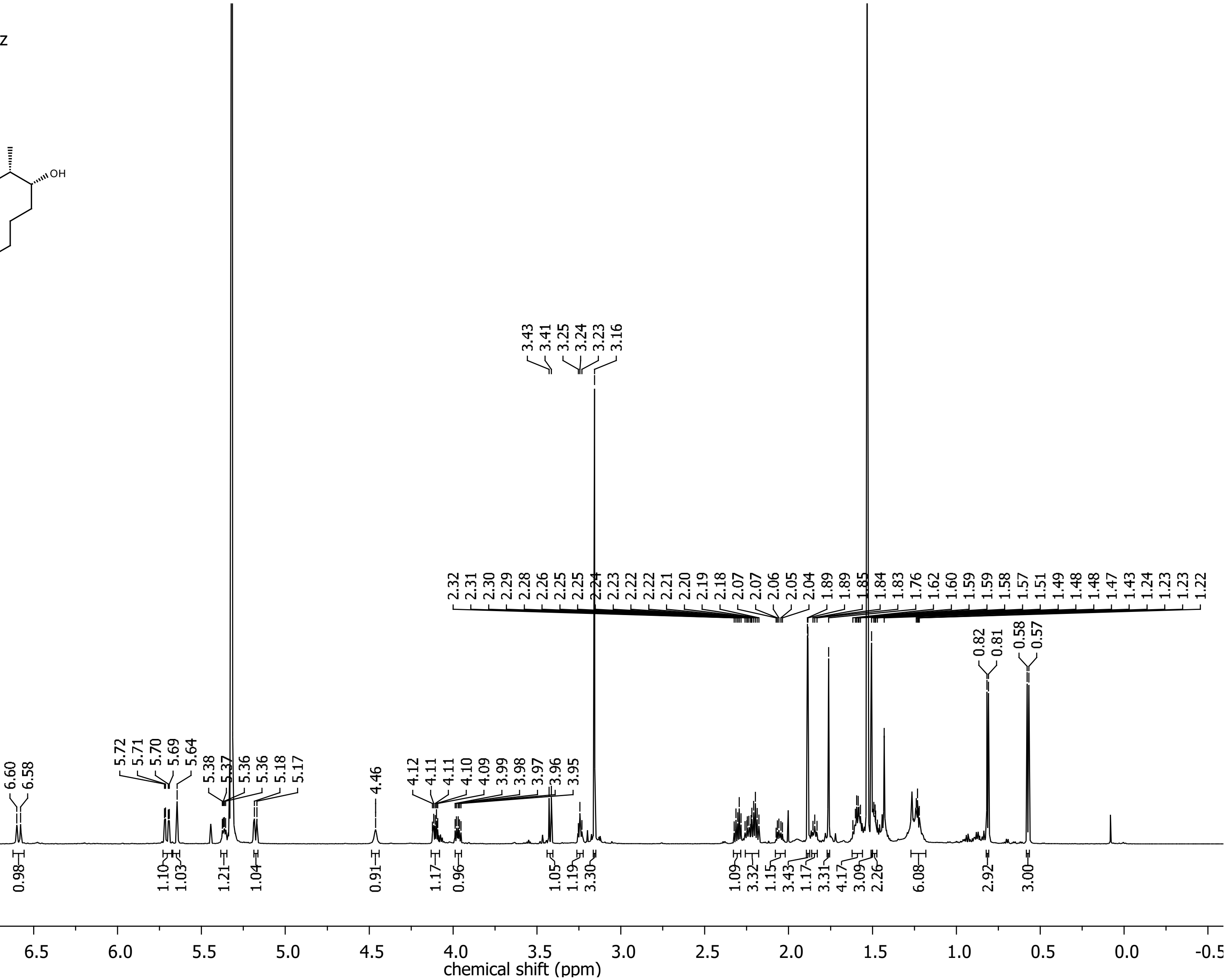

Nucleus: 13C
Frequency: 176.12 MHz
Solvent: CD2Cl2
Temperature: 298.0 K

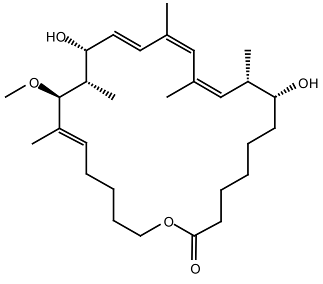

8

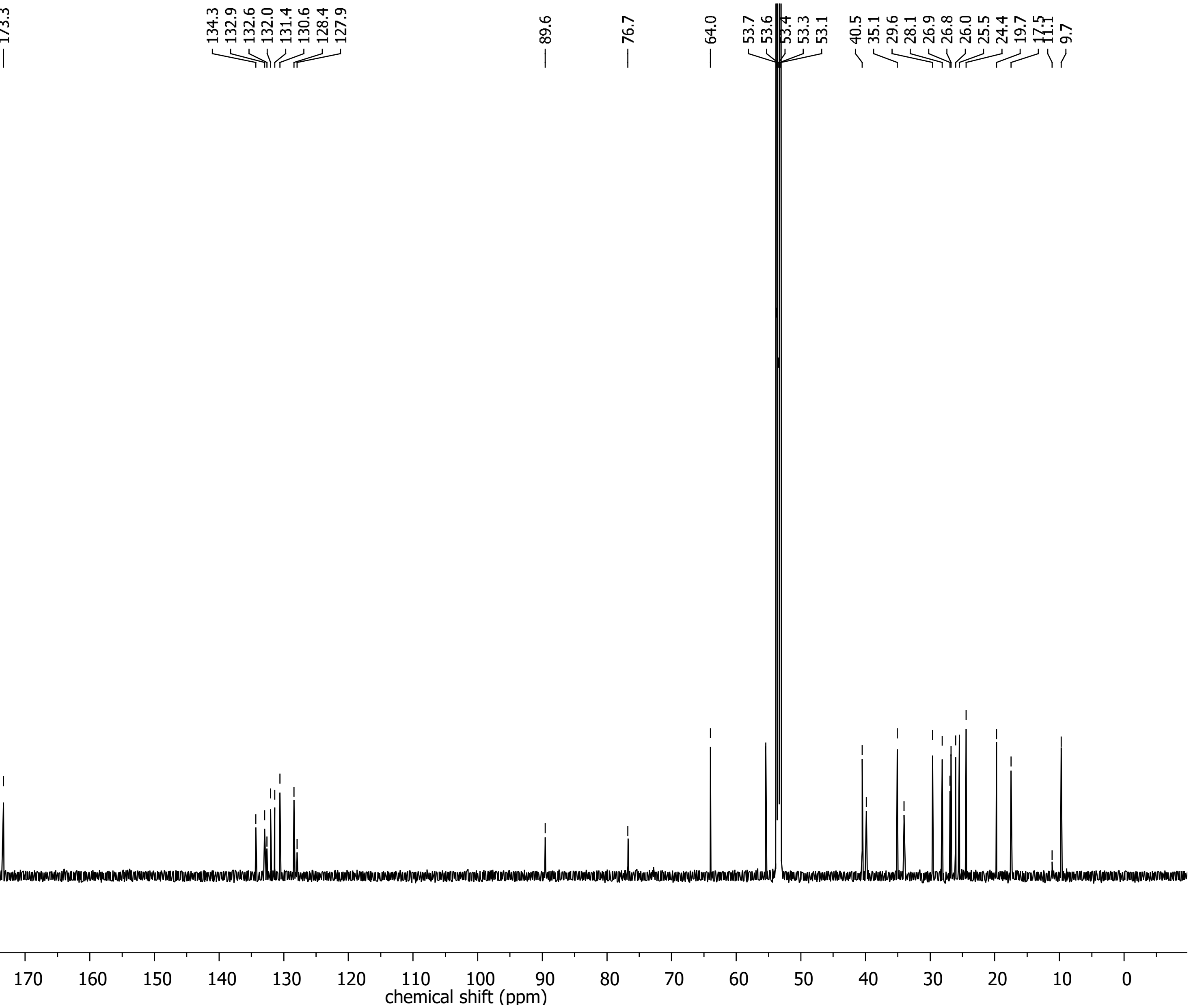

Nucleus: 1H
Frequency: 300.13 MHz
Solvent: CDCl3
Temperature: 298.0 K

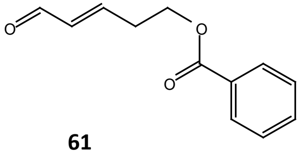

61

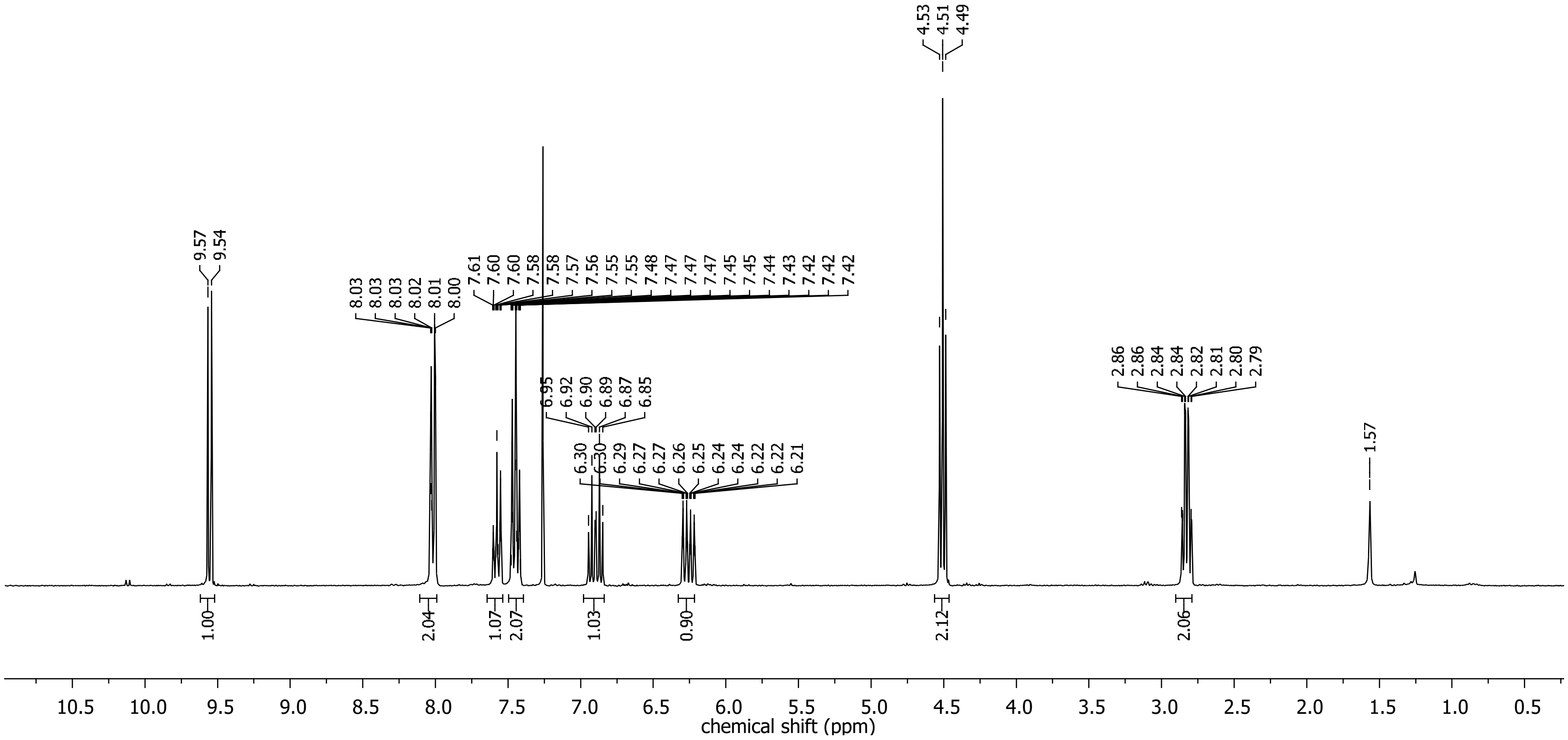

Nucleus: 13C
Frequency: 125.51 MHz
Solvent: CDCl3
Temperature: 298.0 K

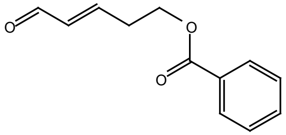

61

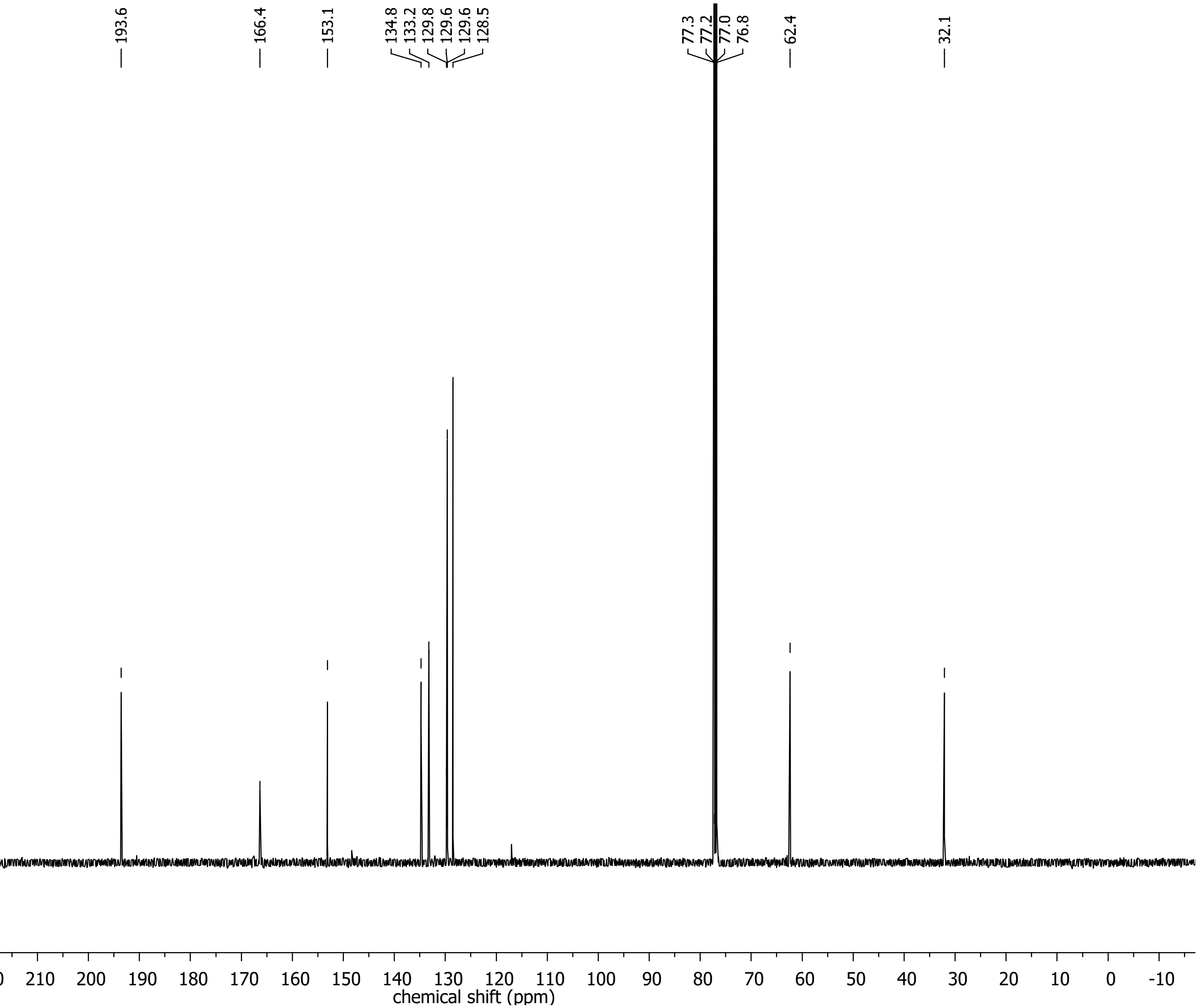

Nucleus: 1H
Frequency: 700.41 MHz
Solvent: CDCl3
Temperature: 298.0 K

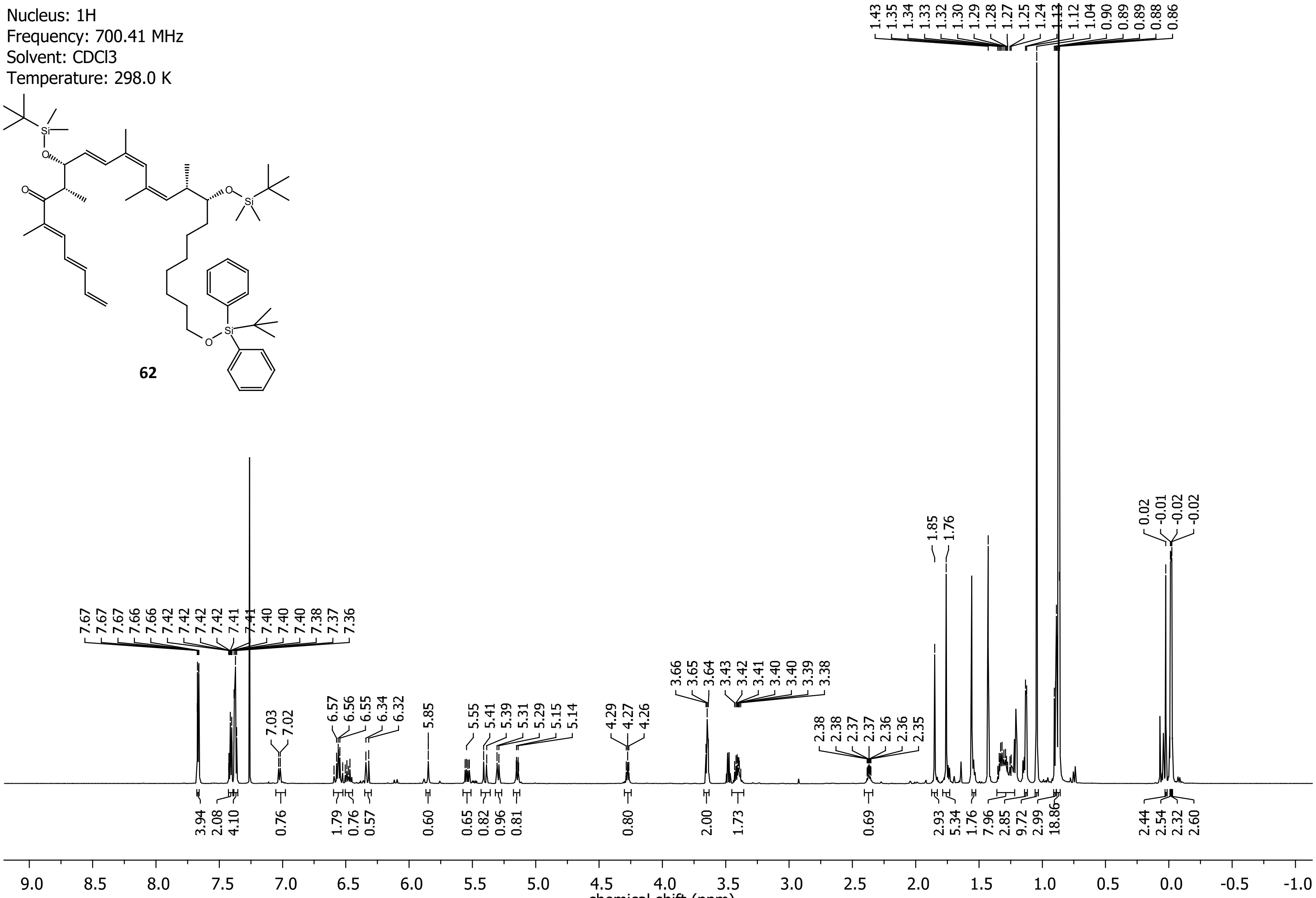

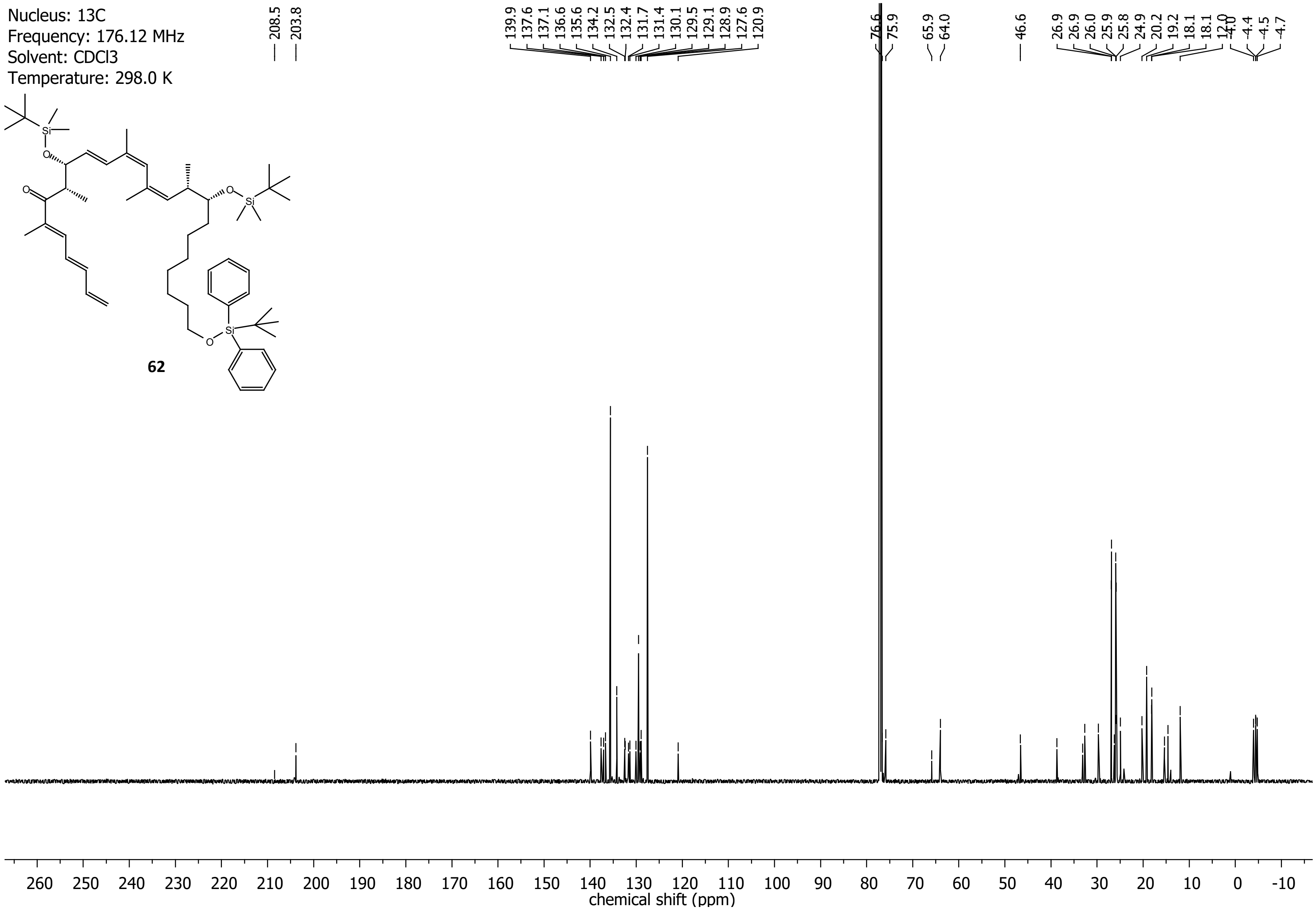
Nucleus: 13C
Frequency: 176.12 MHz
Solvent: CDCl3
Temperature: 298.0 K
208.5
203.8
139.9
137.6
137.1
136.6
135.6
134.2
132.5
132.4
131.7
131.4
130.1
129.5
129.1
128.9
127.6
120.9
76.6
75.9
65.9
64.0
46.6
26.9
26.9
26.0
25.9
25.8
24.9
20.2
19.2
18.1
18.1
12.0
-4.0
-4.4
-4.5
-4.7
O
Si
62
260 250 240 230 220 210 200 190 180 170 160 150 140 130 120 110 100 90 80 70 60 50 40 30 20 10 0 -10
chemical shift (ppm)

Nucleus: 1H
Frequency: 700.41 MHz
Solvent: CDCl3
Temperature: 298.0 K

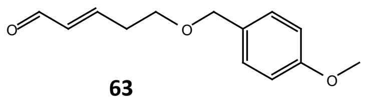

63

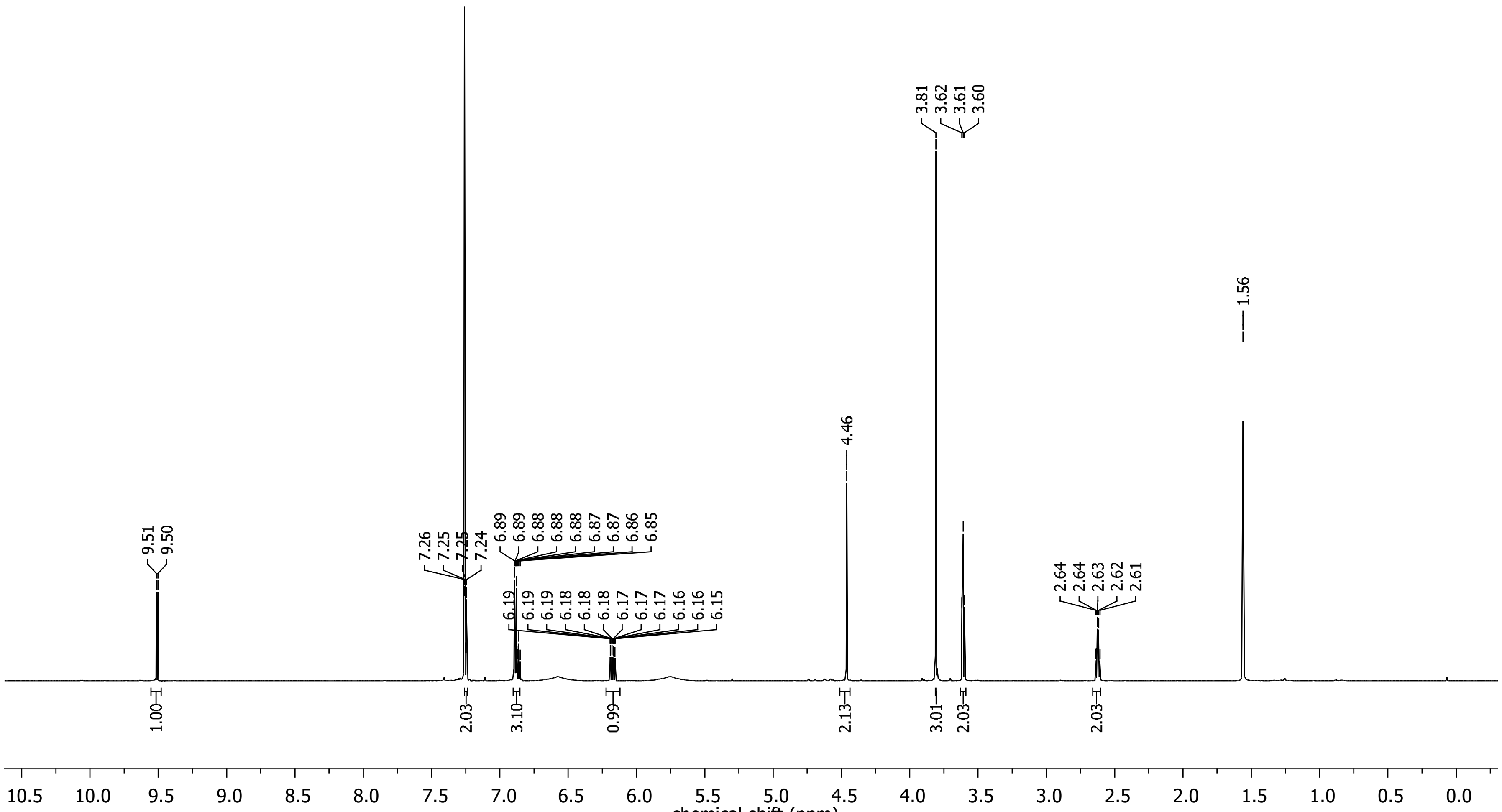

Nucleus: 13C
Frequency: 176.12 MHz
Solvent: CDCl3
Temperature: 298.0 K

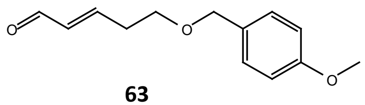

63

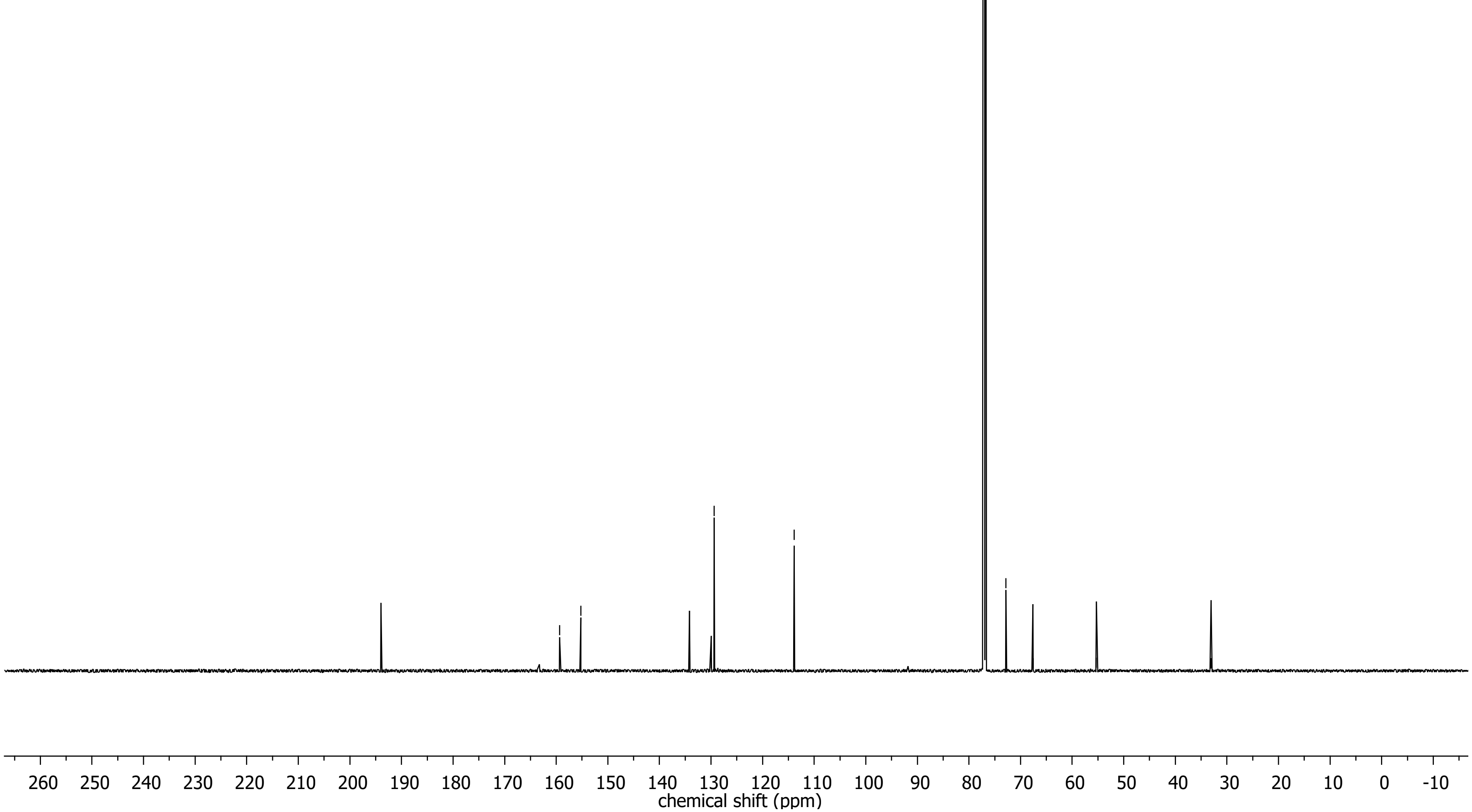

Nucleus: 1H
Frequency: 500.14 MHz
Solvent: CD2Cl2
Temperature: 298.0 K

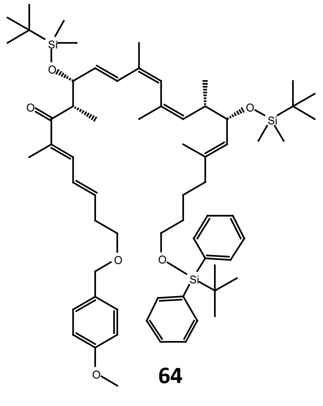

64

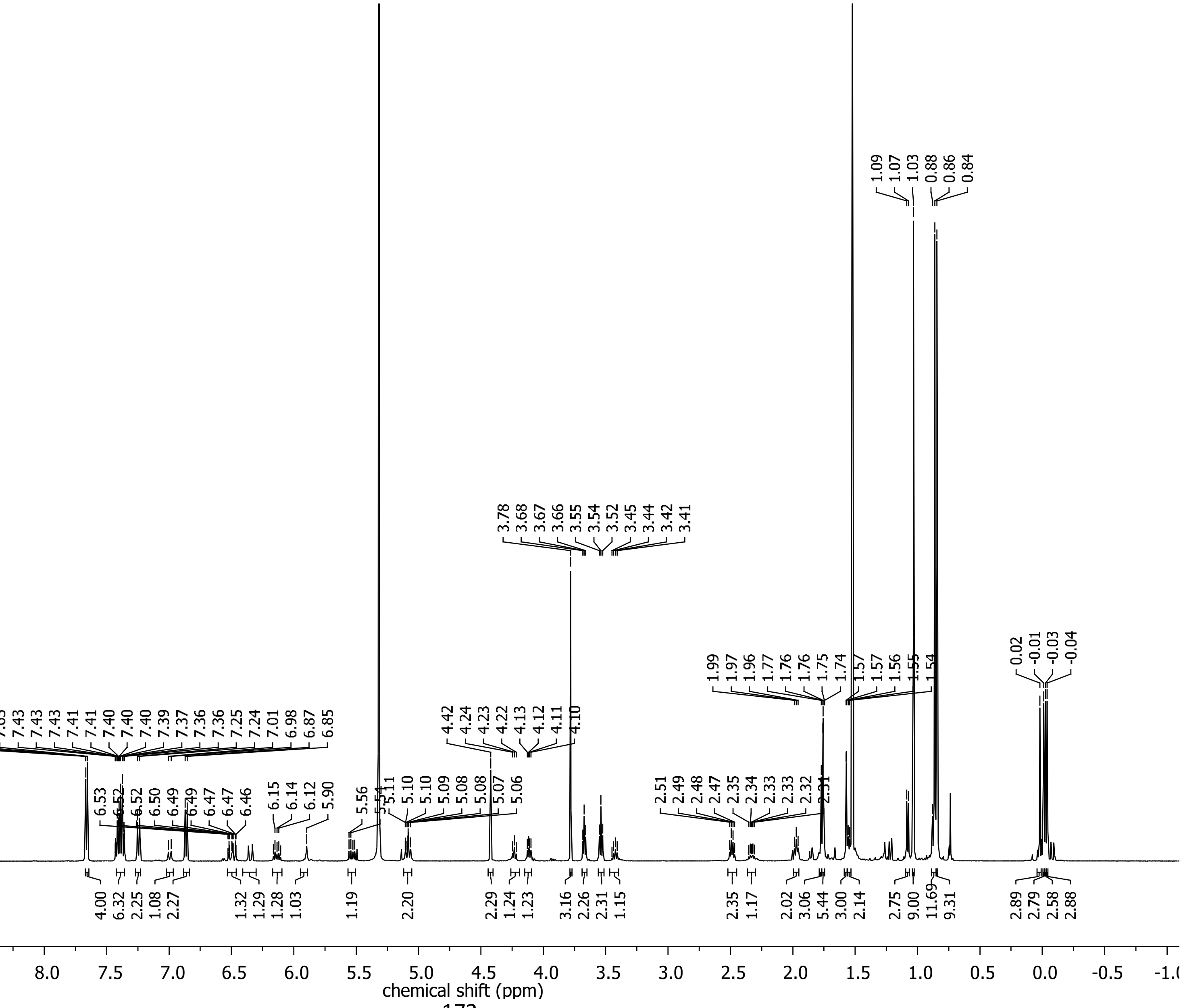

Nucleus: 13C
Frequency: 125.76 MHz
Solvent: CD2Cl2
Temperature: 298.0 K

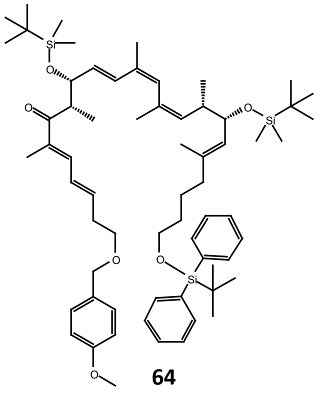

64

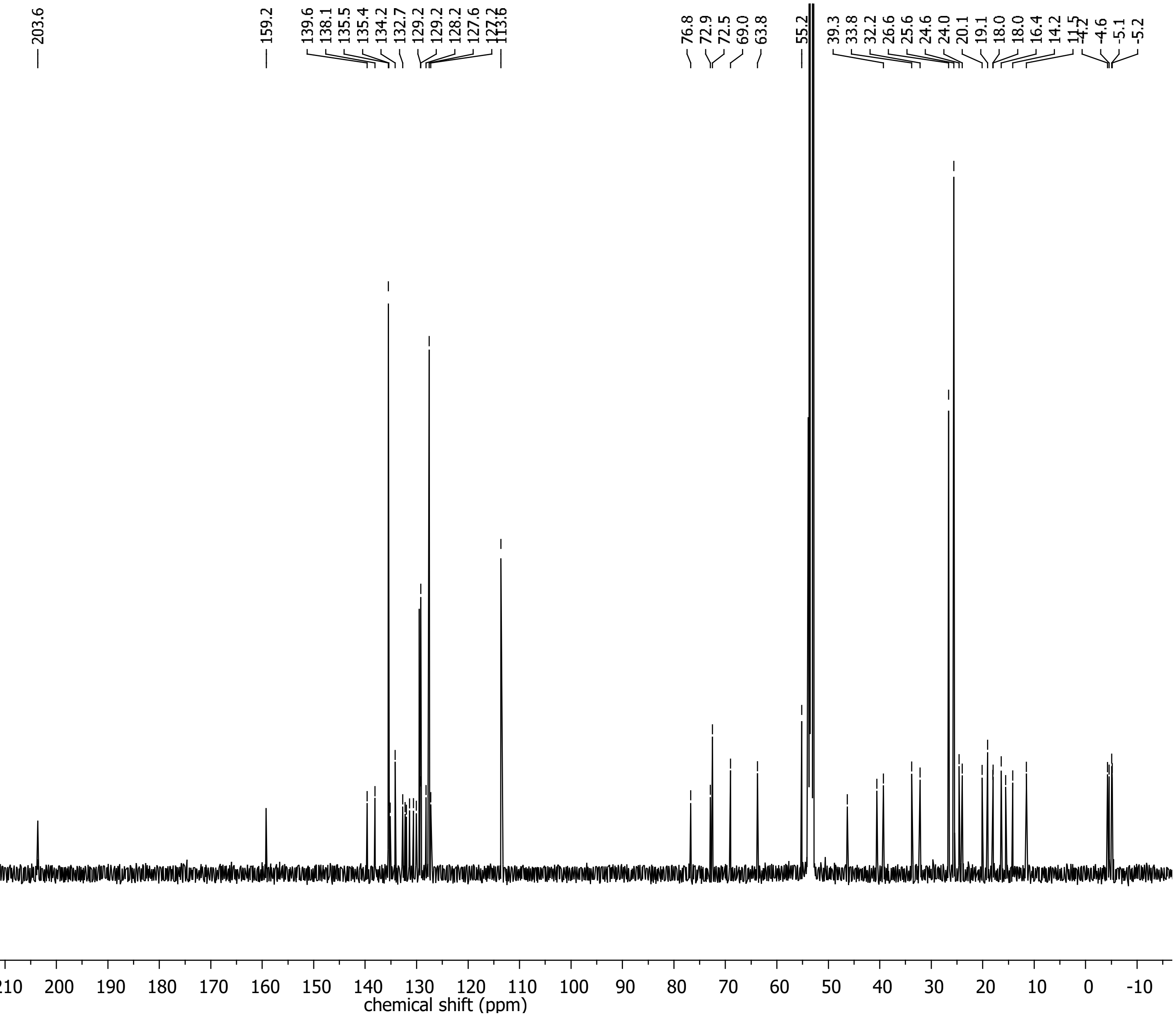

Nucleus: 1H
Frequency: 500.14 MHz
Solvent: CD2Cl2
Temperature: 298.0 K

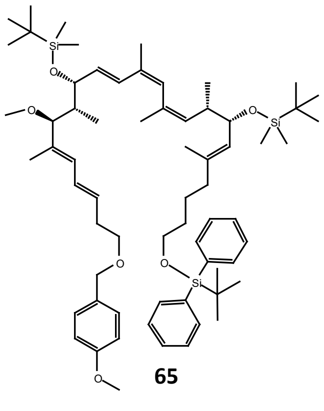

65

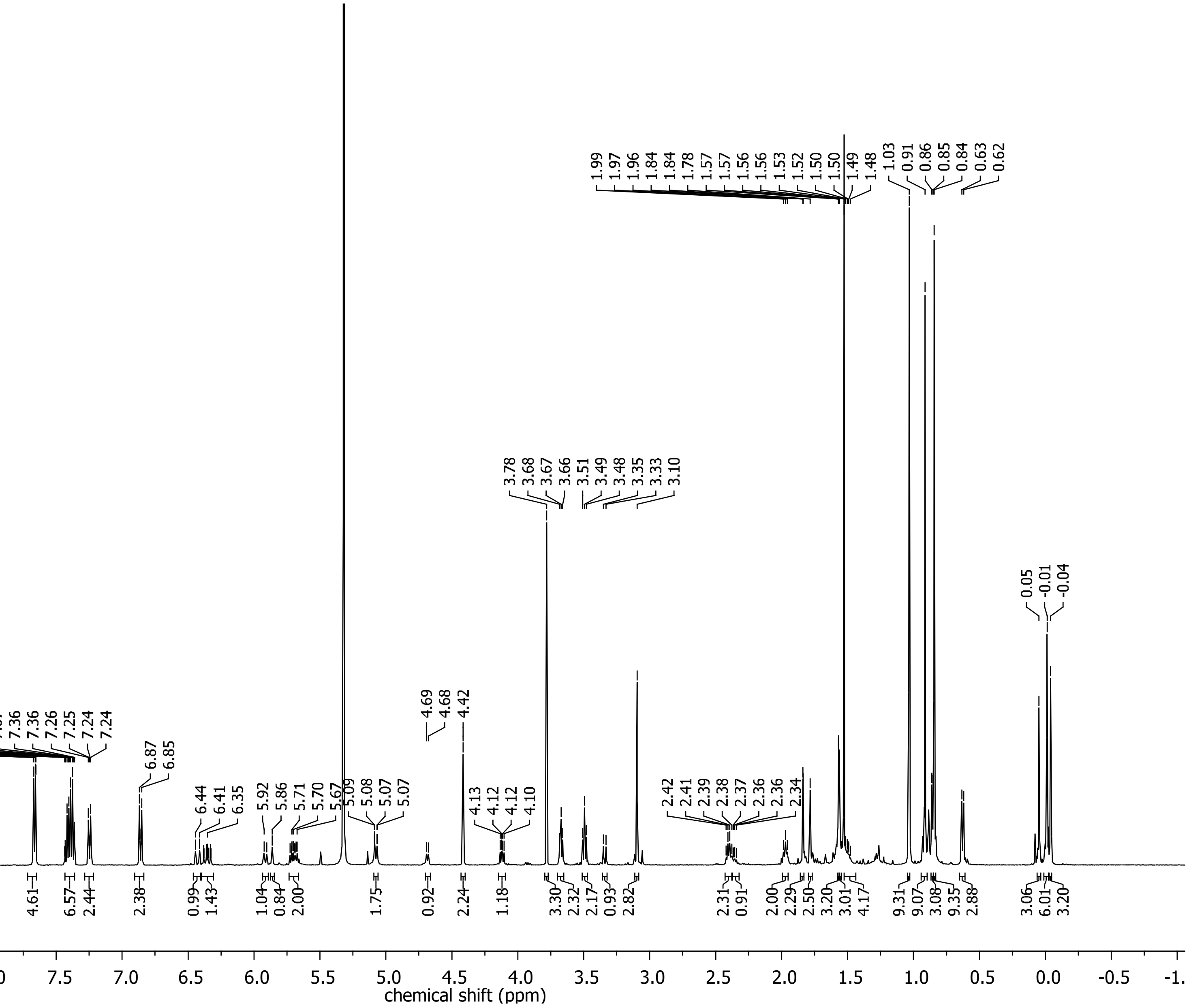

Nucleus: 13C
Frequency: 125.76 MHz
Solvent: CD2Cl2
Temperature: 298.0 K

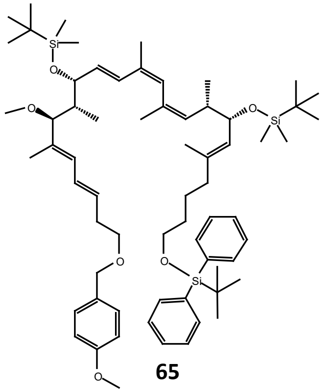

65

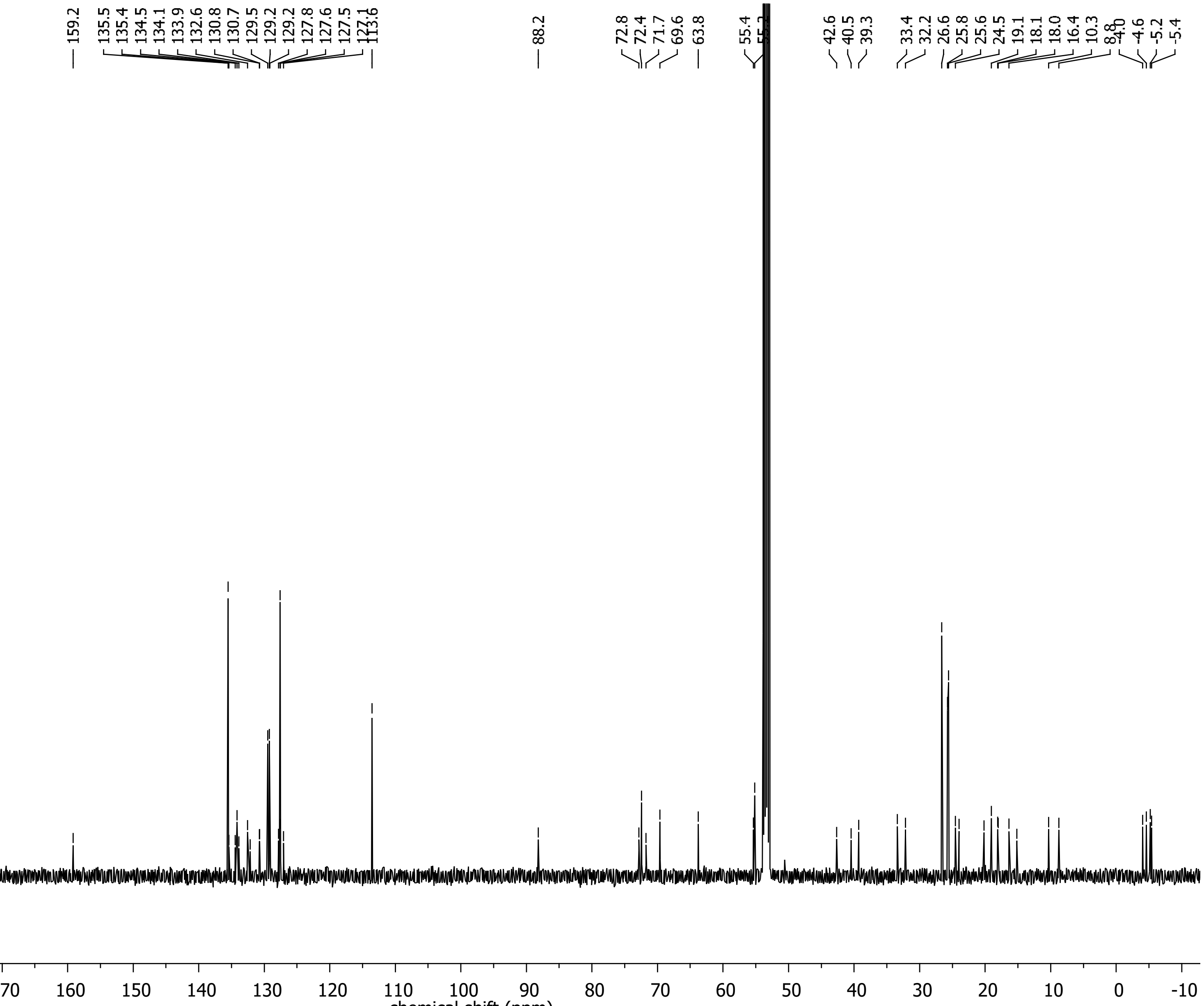

Nucleus: 1H
Frequency: 500.14 MHz
Solvent: CD2Cl2
Temperature: 298.0 K

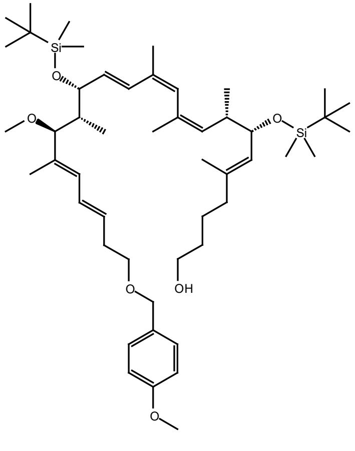

66

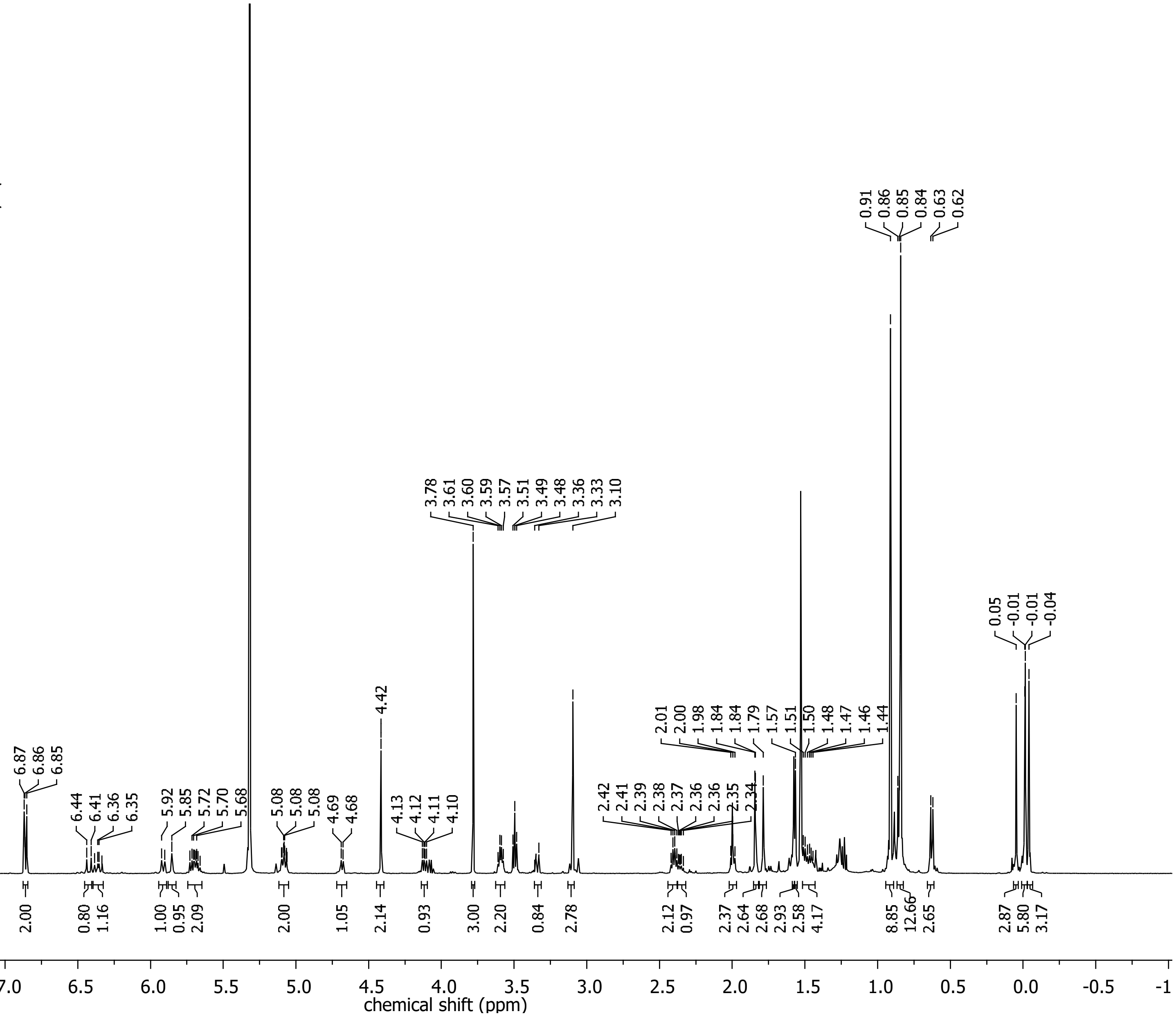

Nucleus: 13C
Frequency: 125.76 MHz
Solvent: CD2Cl2
Temperature: 298.0 K

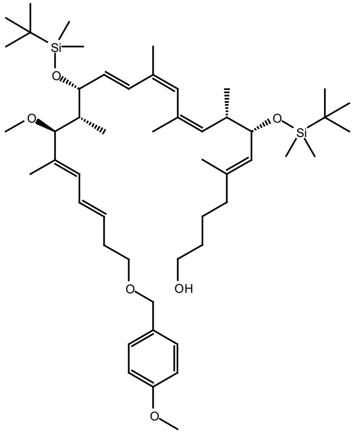

66

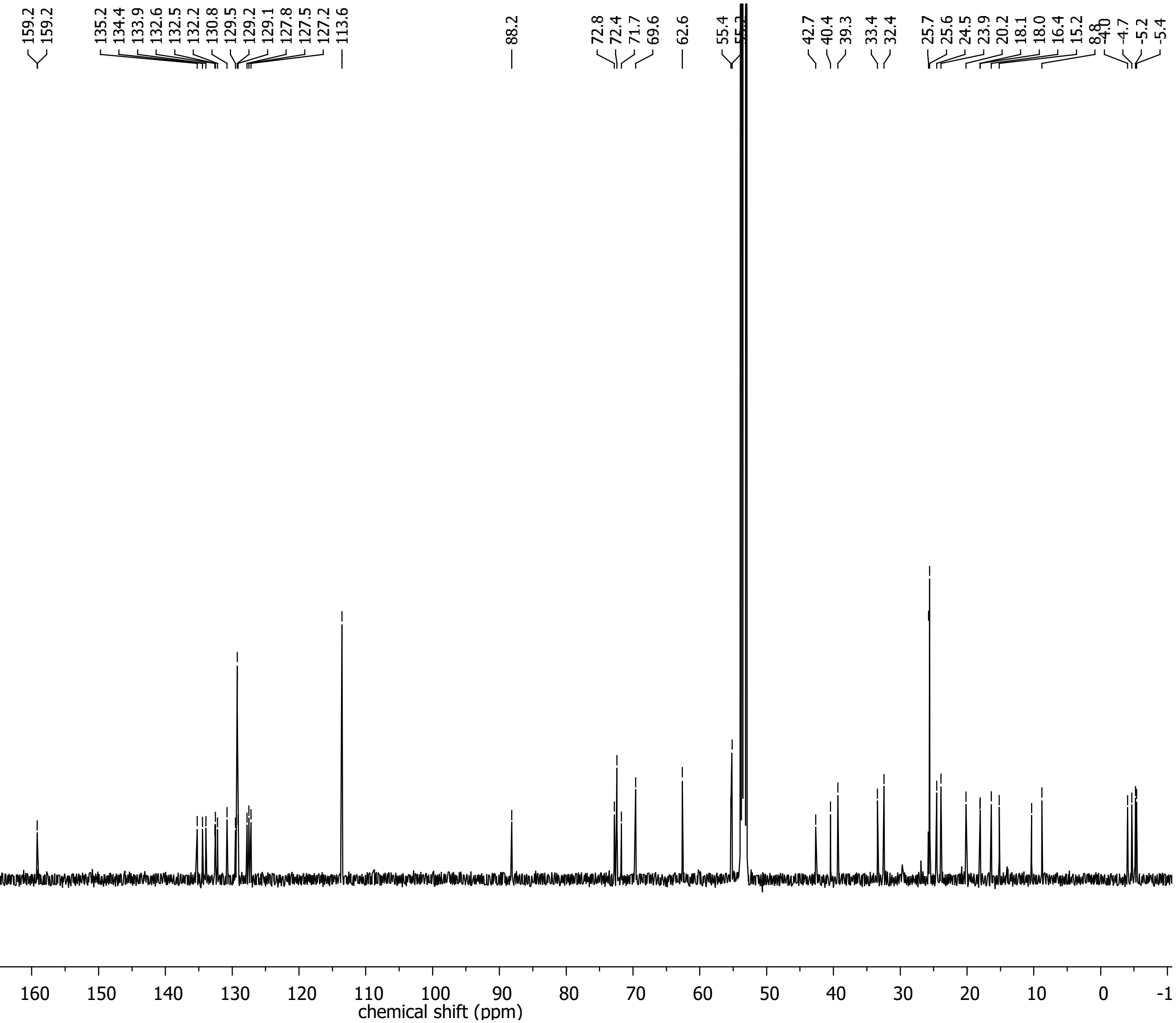

Nucleus: 1H
Frequency: 700.41 MHz
Solvent: CD2Cl2
Temperature: 298.0 K

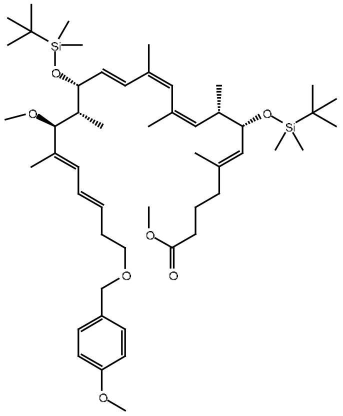

67

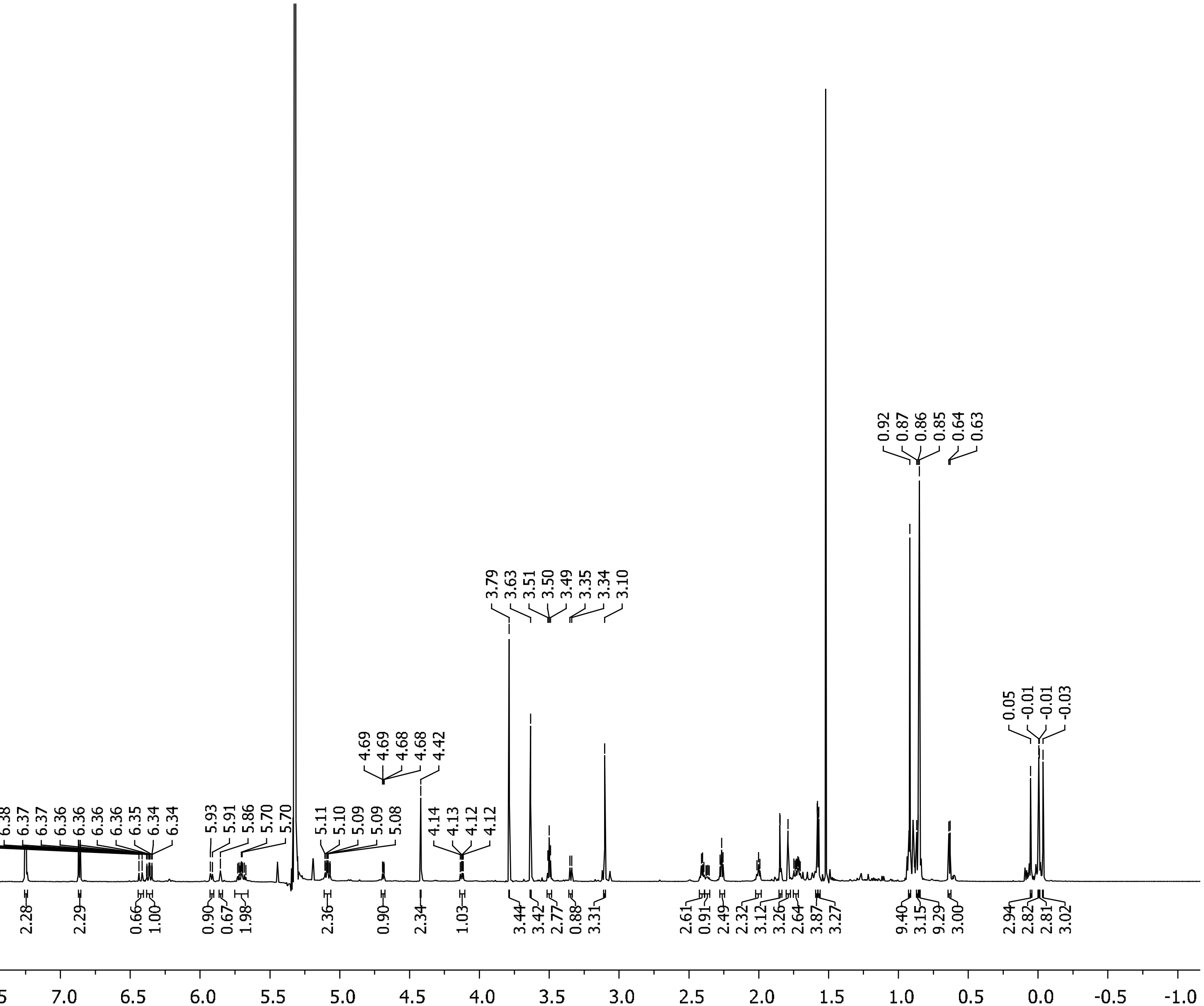

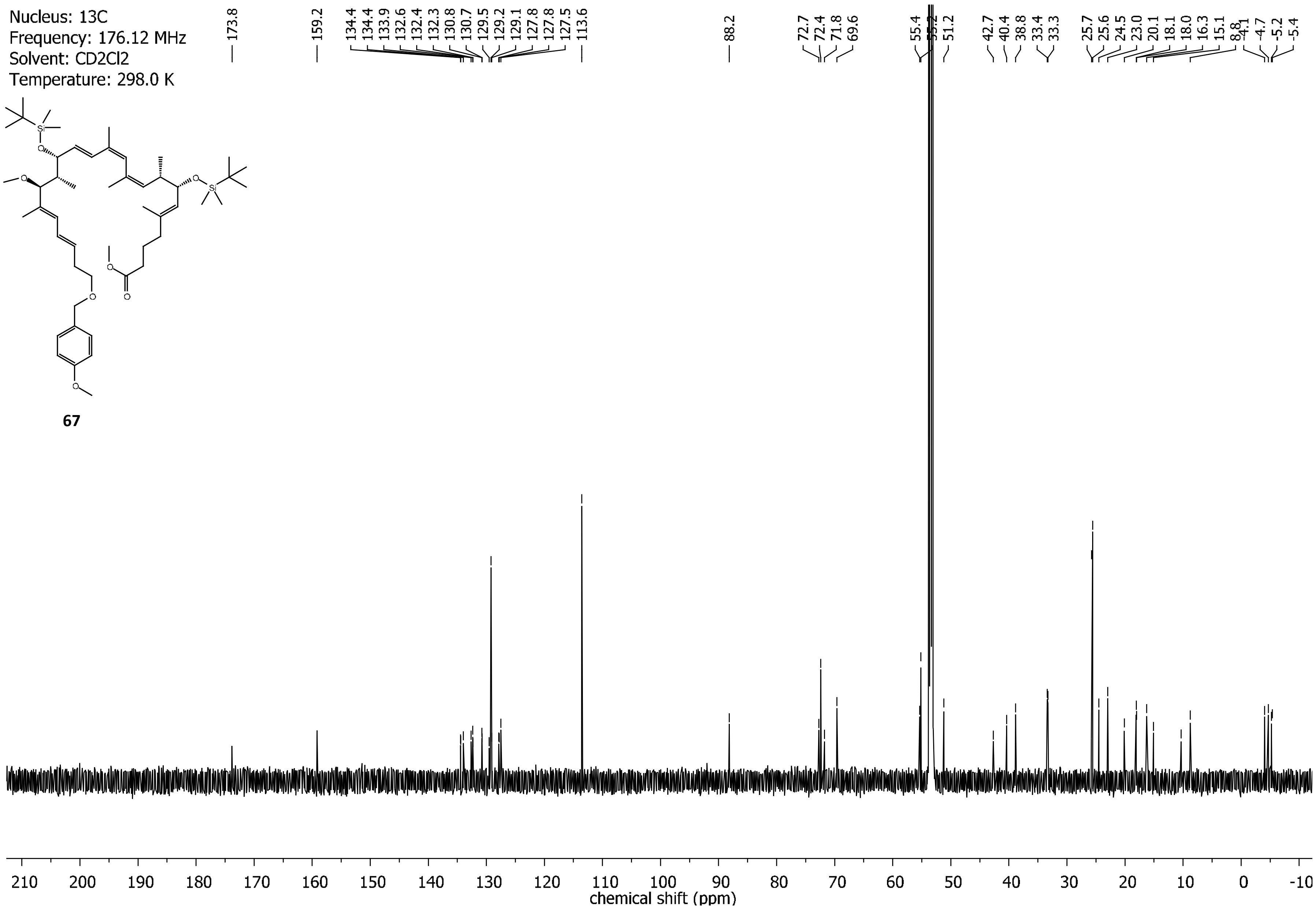
Nucleus: 13C
Frequency: 176.12 MHz
Solvent: CD2Cl2
Temperature: 298.0 K
173.8
159.2
134.4
134.4
133.9
132.6
132.4
132.3
130.8
130.7
129.5
129.2
129.1
127.8
127.8
127.5
113.6
88.2
72.7
72.4
71.8
69.6
55.4
51.2
42.7
40.4
38.8
33.4
33.3
25.7
25.6
24.5
23.0
20.1
18.1
18.0
16.3
15.1
8.8
-4.1
-4.7
-5.2
-5.4
Si
O
O
Si
O
O
O
O
O
67
210 200 190 180 170 160 150 140 130 120 110 100 90 80 70 60 50 40 30 20 10 0 -10
chemical shift (ppm)

Nucleus: 1H
Frequency: 500.14 MHz
Solvent: CD2Cl2
Temperature: 298.0 K

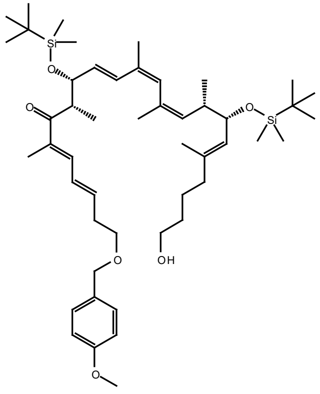

68

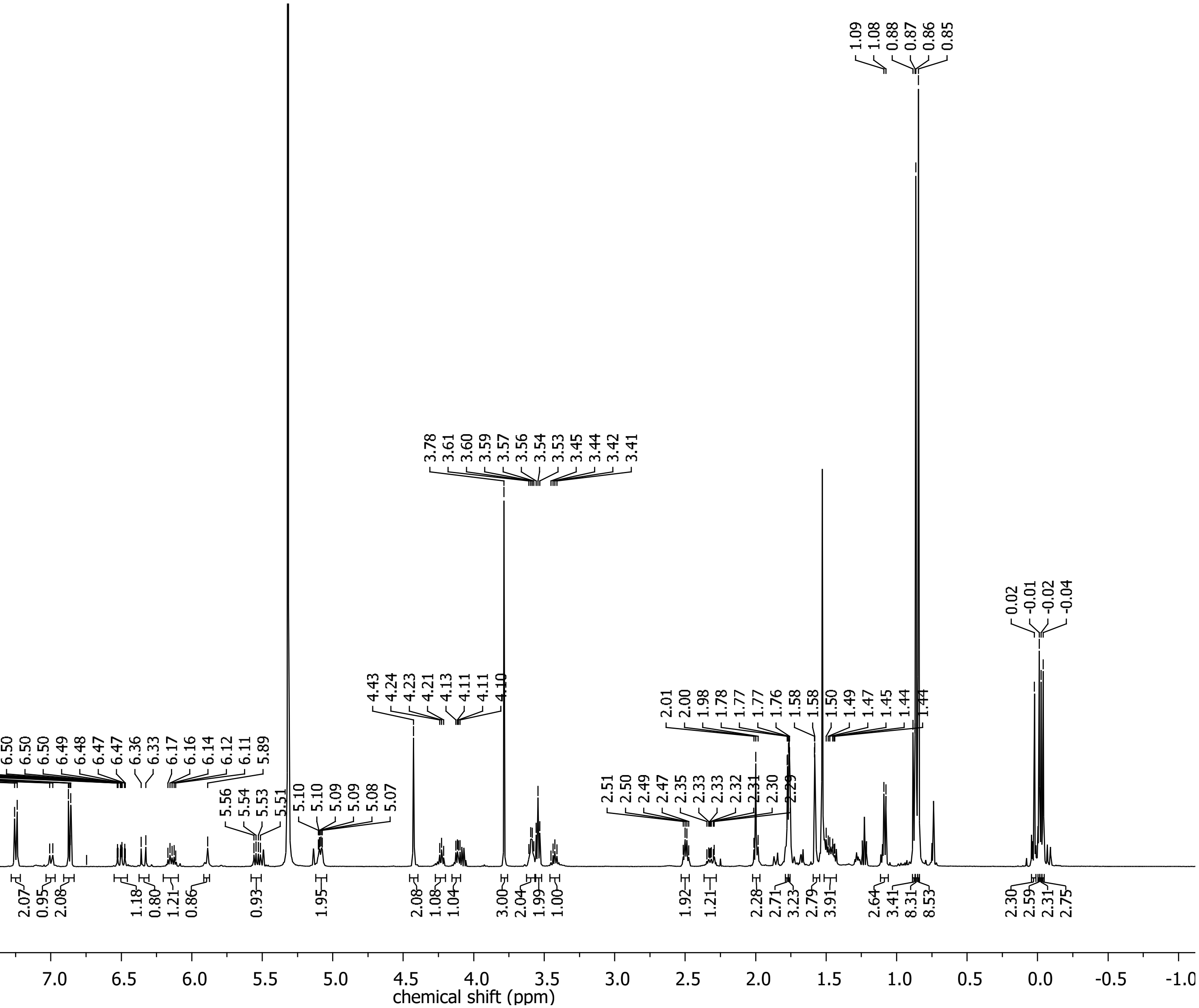

Nucleus: 13C
Frequency: 125.76 MHz
Solvent: CD2Cl2
Temperature: 298.0 K

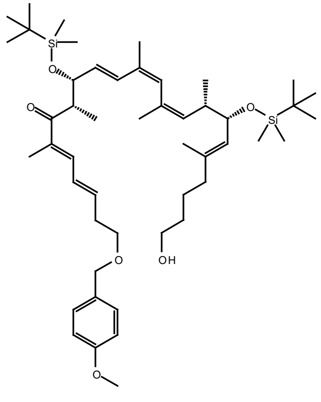

68

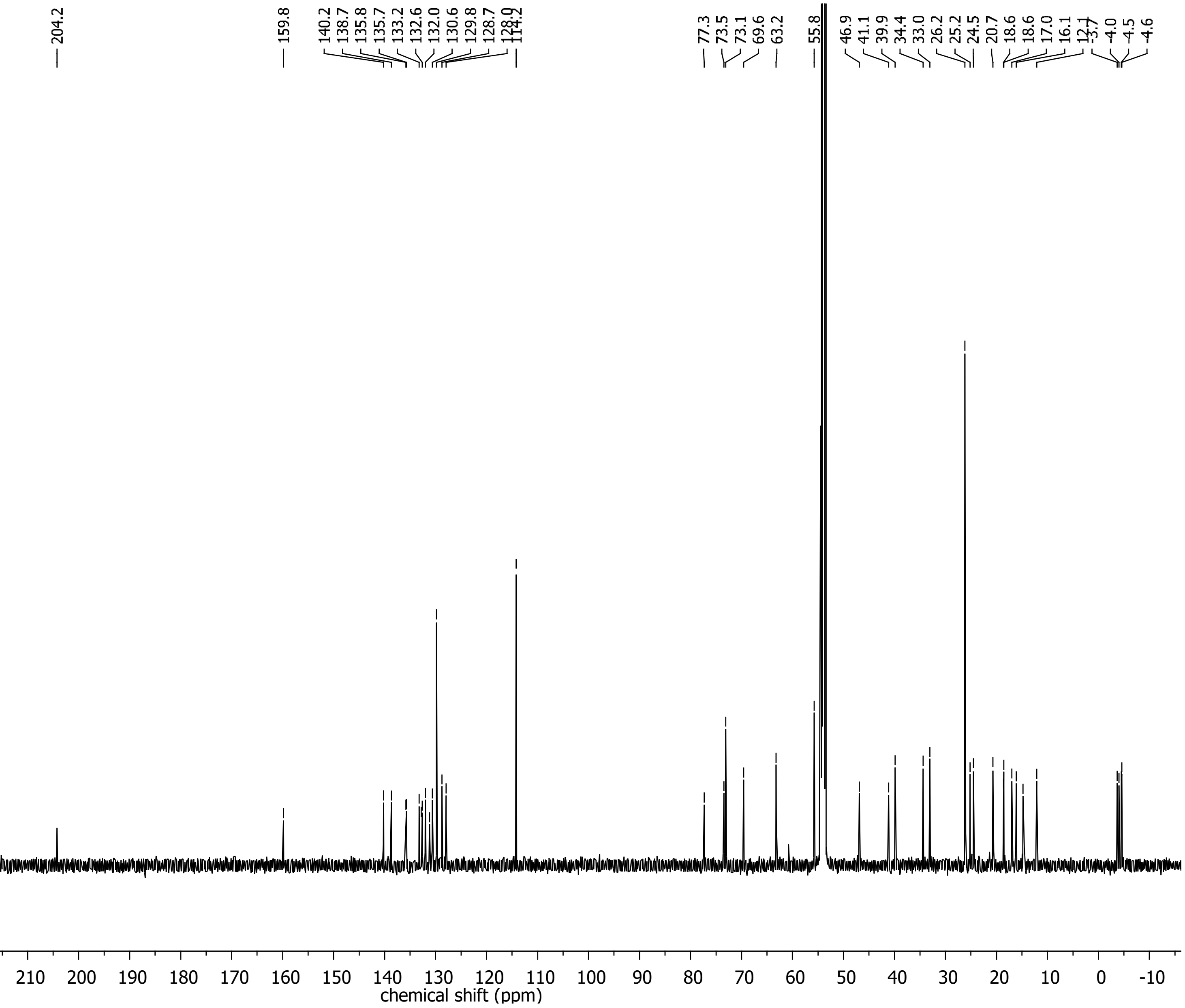

Nucleus: 1H
Frequency: 500.14 MHz
Solvent: CD2Cl2
Temperature: 298.0 K

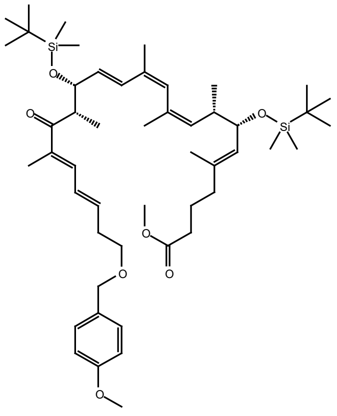

69

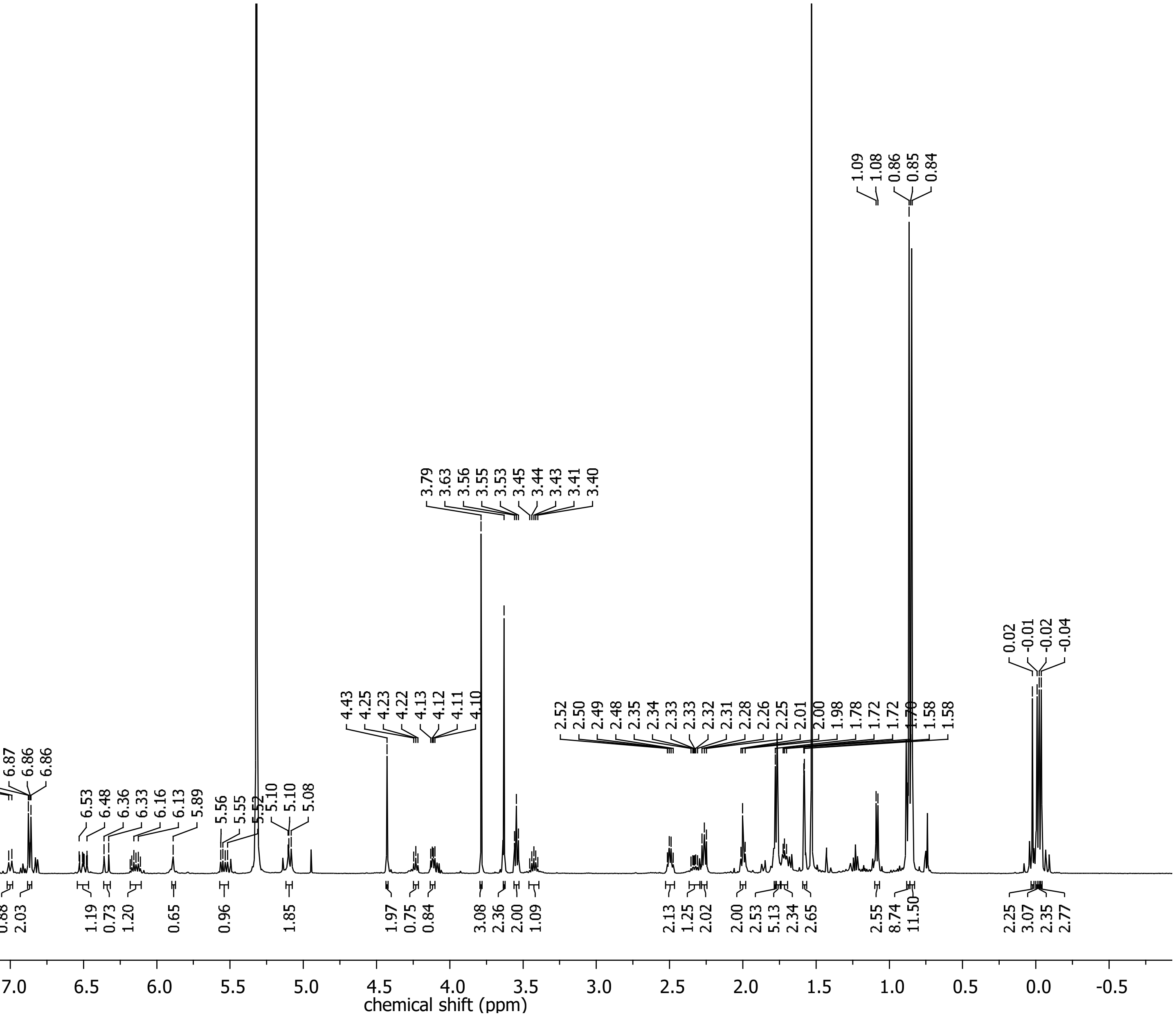

Nucleus: 13C
Frequency: 125.76 MHz
Solvent: CD2Cl2
Temperature: 298.0 K

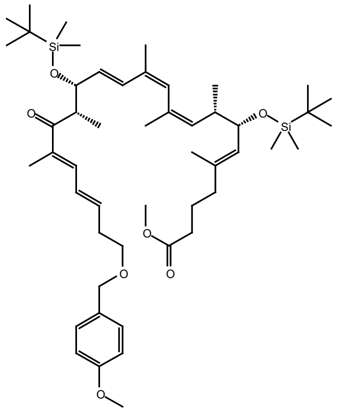

69

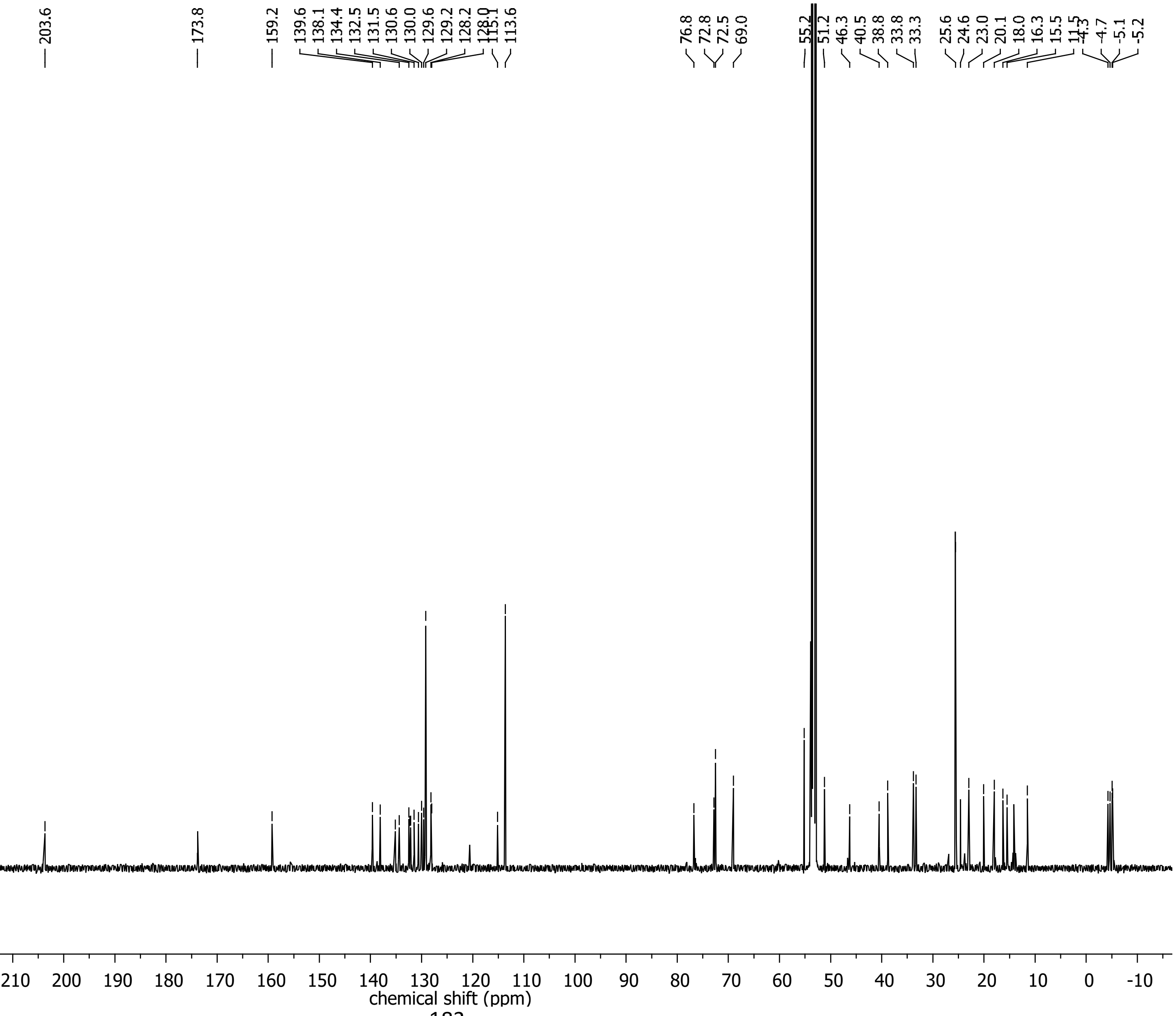

Nucleus: 1H
Frequency: 700.41 MHz
Solvent: CD2Cl2
Temperature: 298.0 K

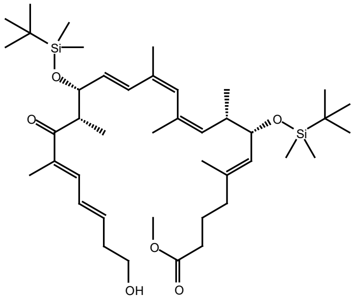

70

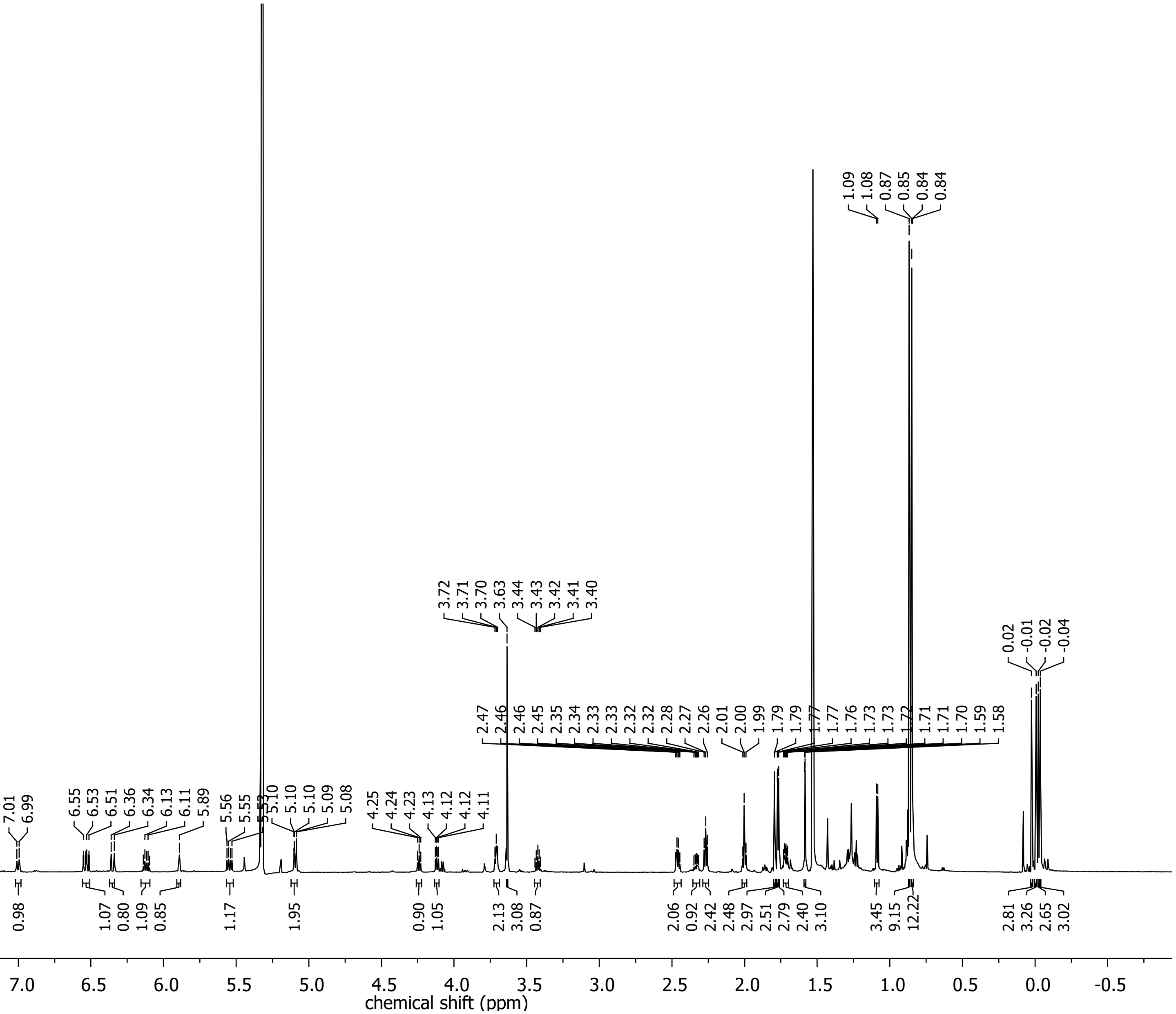

Nucleus: 13C
Frequency: 176.12 MHz
Solvent: CD2Cl2
Temperature: 298.0 K

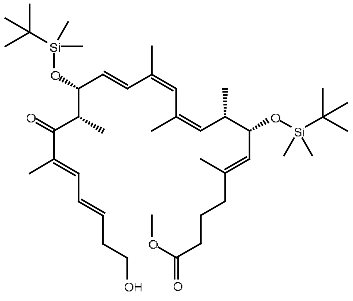

70

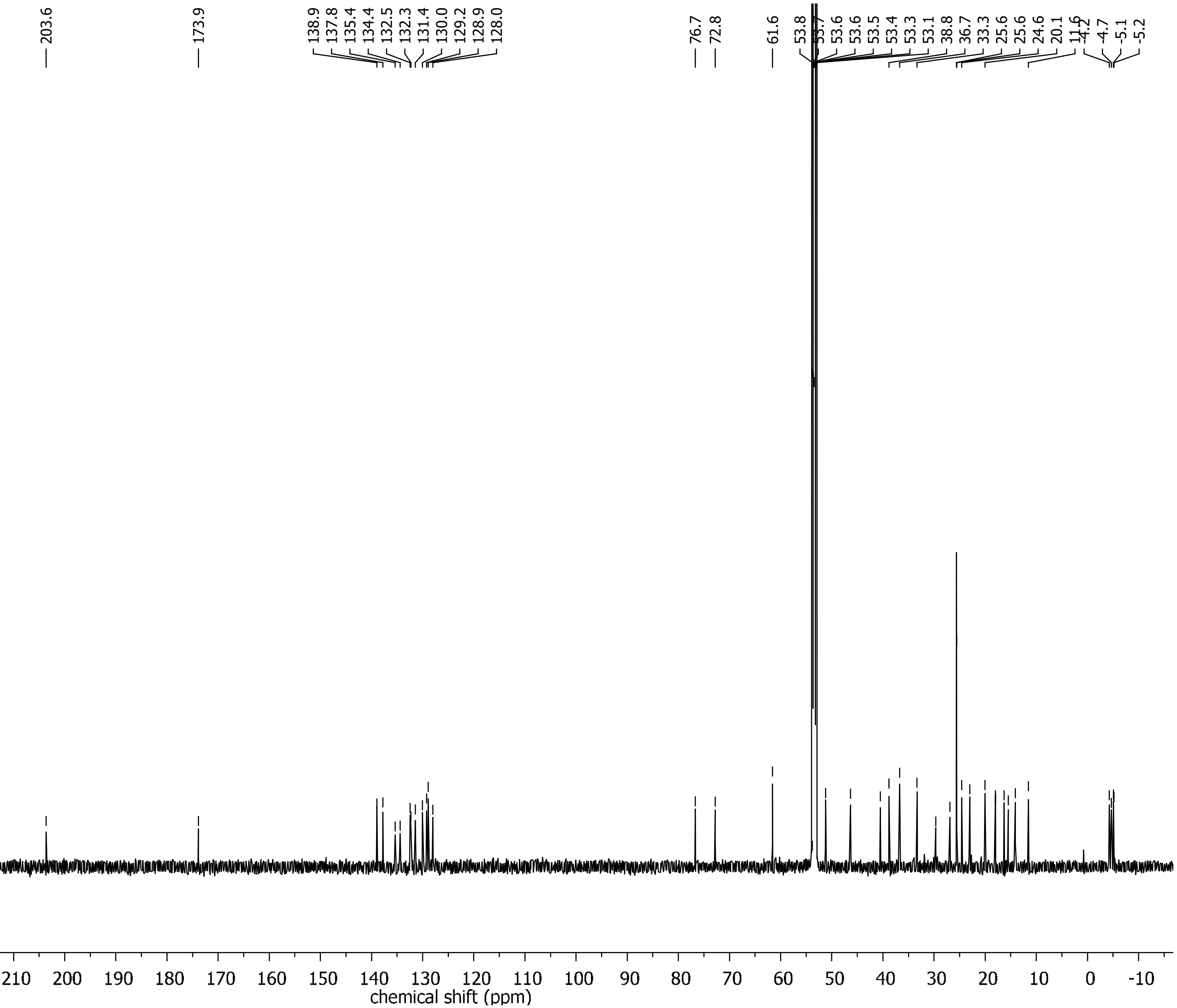

Nucleus: 1H
Frequency: 700.41 MHz
Solvent: CDCl3
Temperature: 298.0 K

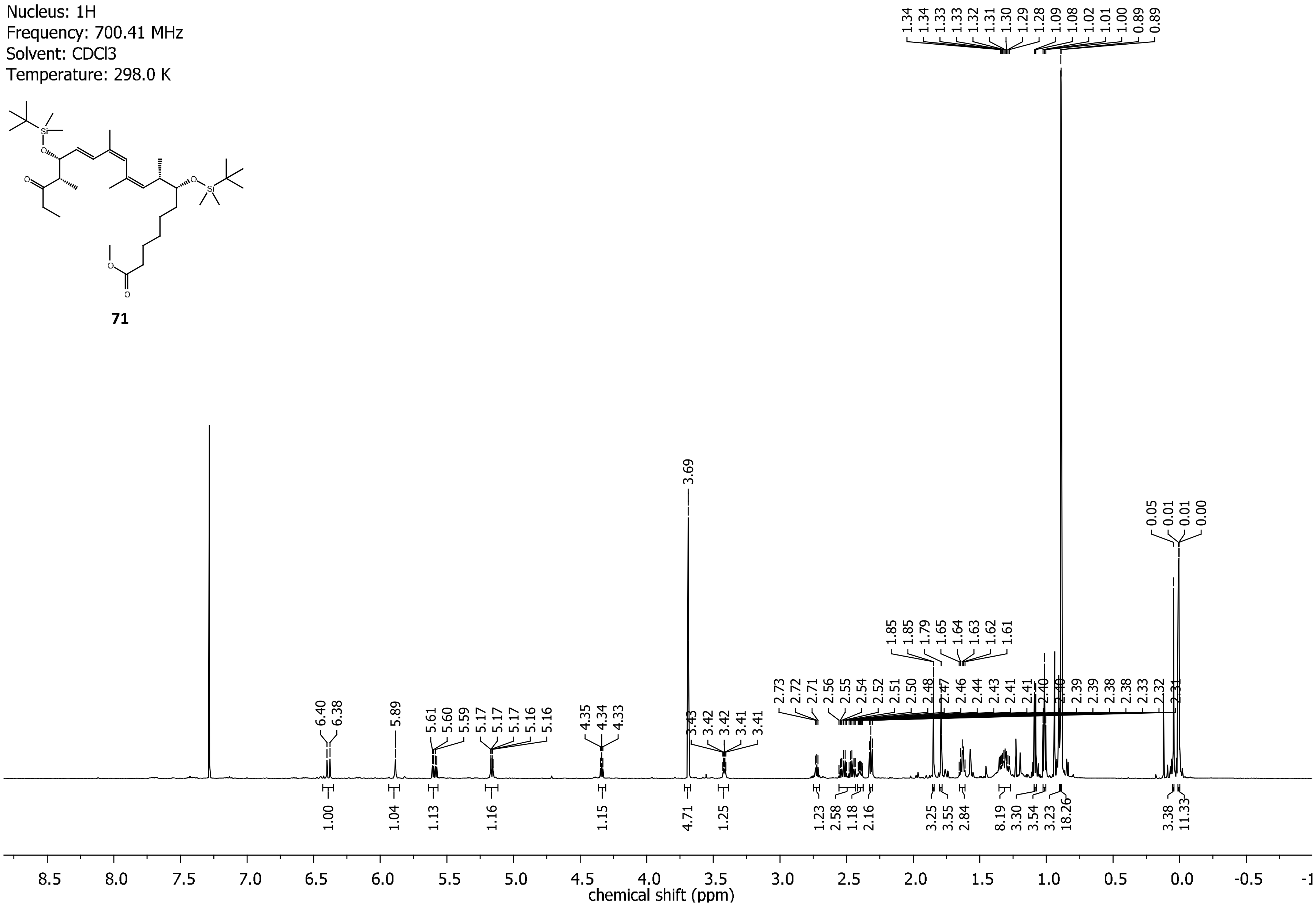

Nucleus: 13C
Frequency: 176.12 MHz
Solvent: CDCl3
Temperature: 298.0 K

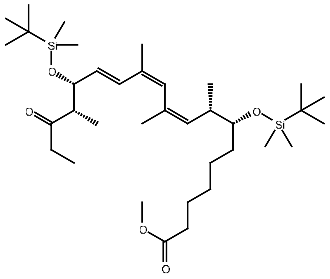

71

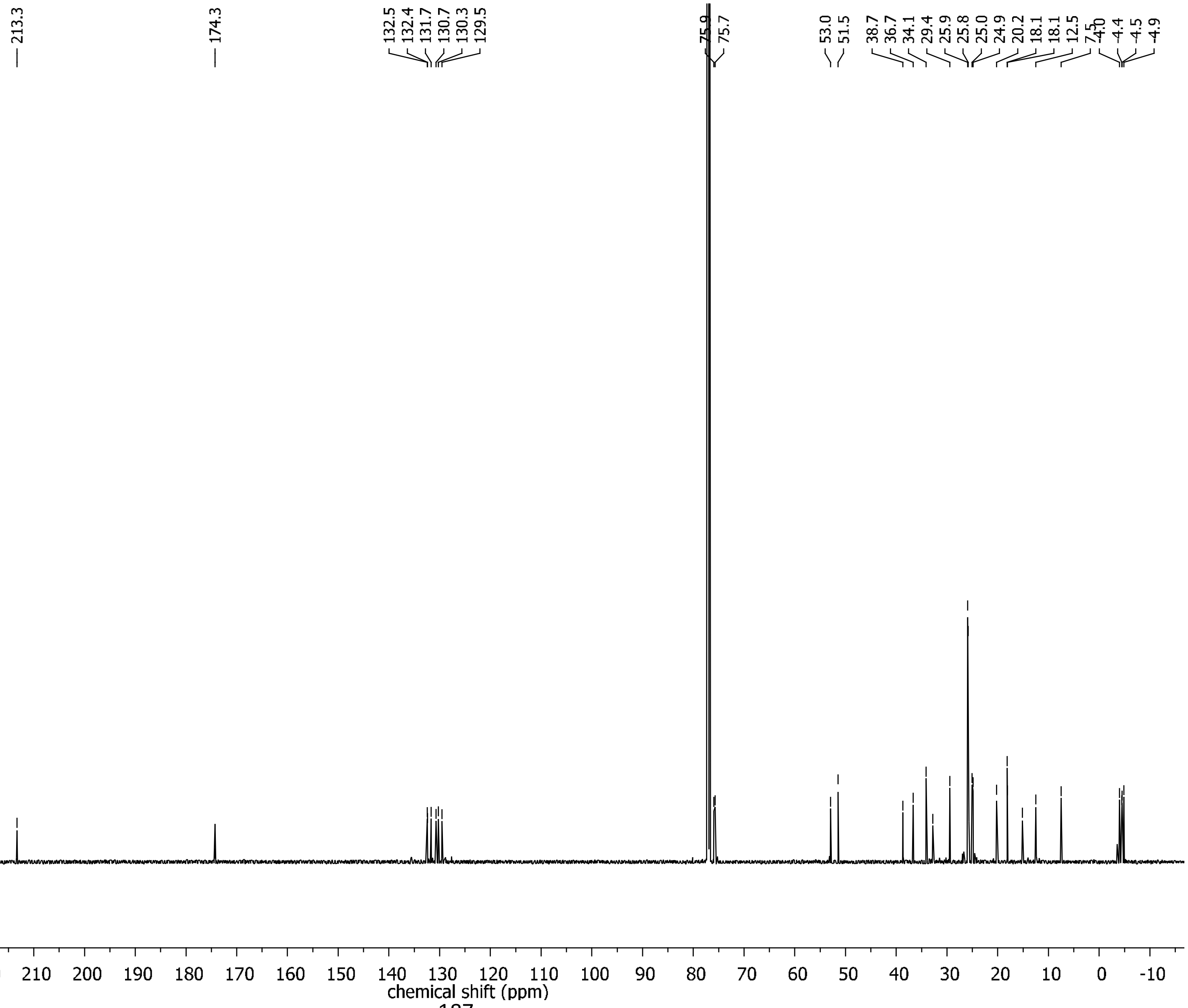

Nucleus: 1H
Frequency: 700.41 MHz
Solvent: CD2Cl2
Temperature: 298.0 K

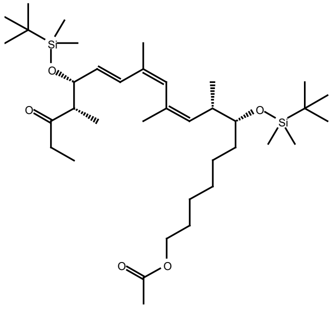

72

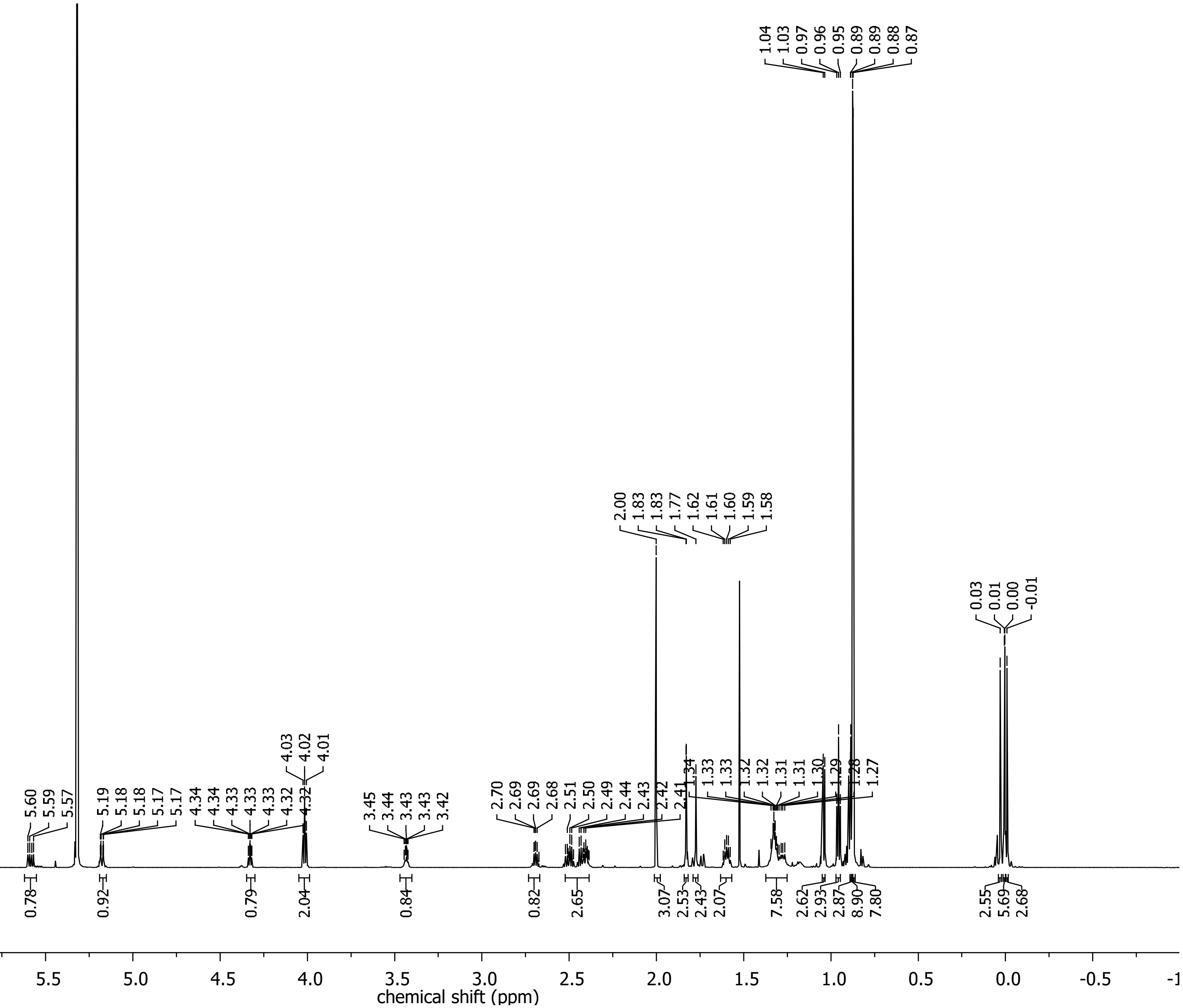

Nucleus: 13C
Frequency: 176.12 MHz
Solvent: CD2Cl2
Temperature: 298.0 K

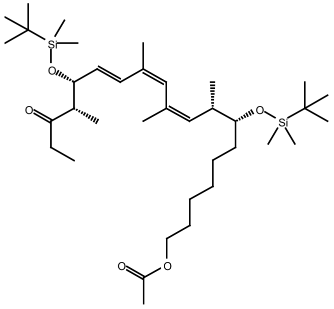

72

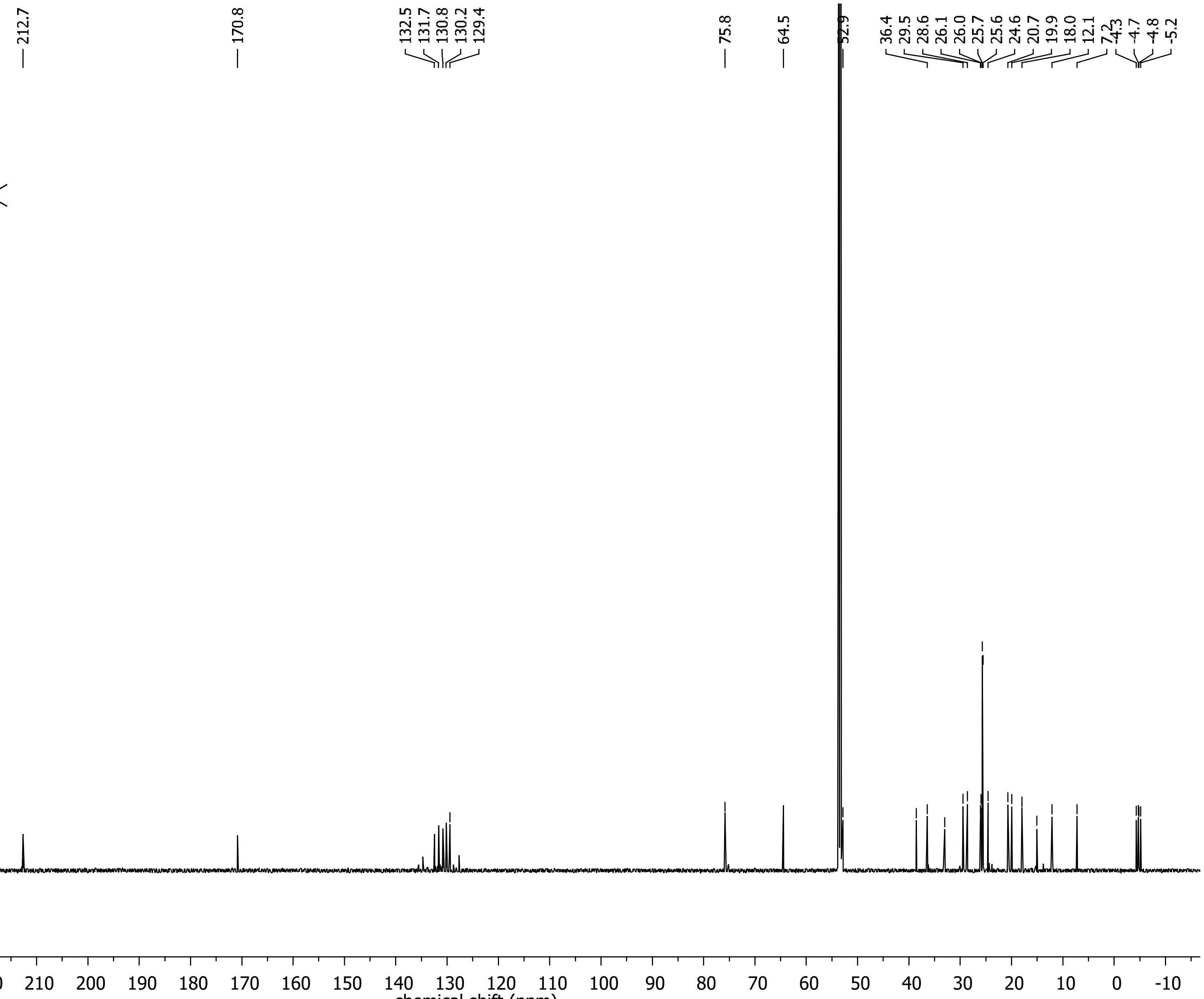

Supplement: Supplementary file 1 — Supplementary [file CMDC-15-1348-s001.pdf]
